# Ir-catalyzed enantioselective B−H alkenylation for asymmetric synthesis of chiral-at-cage *o*‑carboranes

**DOI:** 10.1038/s41467-021-27441-y

**Published:** 2021-12-08

**Authors:** Ruofei Cheng, Jie Zhang, Huifang Zhang, Zaozao Qiu, Zuowei Xie

**Affiliations:** 1 Shanghai-Hong Kong Joint Laboratory in Chemical Synthesis, Shanghai Institute of Organic Chemistry, University of Chinese Academy of Sciences, Chinese Academy of Sciences, 345 Lingling Rd, 200032 Shanghai, China; 2 Department of Chemistry and State Key Laboratory of Synthetic Chemistry, The Chinese University of Hong Kong, Shatin, New Territories, Hong Kong, China; 3 School of Chemistry and Chemical Engineering, Henan Normal University, 453007 Xinxiang, Henan China; 4 CAS Key Laboratory of Energy Regulation Materials, Shanghai Institute of Organic Chemistry, Chinese Academy of Sciences, 345 Lingling Rd, 200032 Shanghai, China

**Keywords:** Asymmetric catalysis, Asymmetric synthesis, Synthetic chemistry methodology

## Abstract

The asymmetric synthesis of chiral-at-cage *o*-carboranes, whose chirality is associated with the substitution patterns on the polyhedron, is of great interest as the icosahedral carboranes have wide applications in medicinal and materials chemistry. Herein we report an intermolecular Ir-catalyzed enantioselective B−H alkenylation for efficient and facile synthesis of chiral-at-cage *o*-carboranes with new skeletons under mild reaction conditions. Generally very good to excellent yields with up to 99% ee can be achieved in this Ir-catalyzed B−H alkenylation. The enantiocontrol model is proposed based on Density Functional Theory calculations in which the use of chiral phosphoramidite ligand is essential for such asymmetric *o*-carborane B−H alkenylation.

## Abstract (web summary)

Chiral-at-cage o-carboranes are clusters of carbon and boron atoms that, when functionalized, can lower the symmetry of the cluster. Here the authors present a method to alkenylate B–H bonds on ocarboranes enantioselectively via iridium catalysis.

## Introduction

Stereochemistry has been one of the most active research areas in modern chemistry. Asymmetric catalysis with chiral metal complexes, enzymes, and chiral organic molecules have emerged as successful and powerful tools in asymmetric synthesis to obtain enantiomerically enriched compounds^1–5^. Despite a great variety of chiral structures incorporating central, axial, planar, and helical chirality achieved by catalytic asymmetric synthesis, to obtain the inherent chirality of three-dimensional cage compounds such as carboranes is extremely challenging and the currently available methods to access such an enantioenriched skeleton are still rather limited^6–9^.

**Fig. 1:**
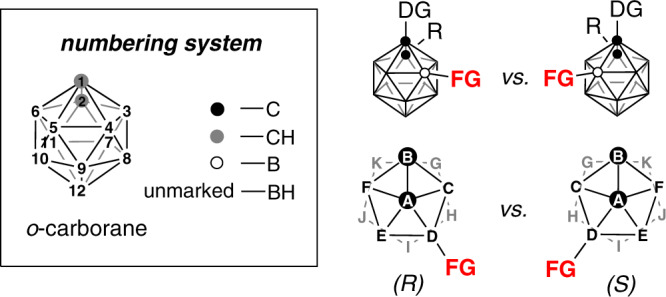
The inherent cage B(4/5) chirality of 1,2-substituted-*o*-carboranes. The presence of a substituent at the position 4/5 results in the chirality of the molecule. The observer looks onto the pentagonal plane of C(2)–B(3)–B(4)–B(5)–B(6) in *o*-carborane and then examines the position of the substituent according to the Cahn–Ingold–Prelog rule for the determination of the cage chirality.

Icosahedral carboranes are carbon–boron molecular clusters, often viewed as three-dimensional analogs to benzene, which are invaluable building blocks for applications ranging from functional materials to pharmaceuticals^10–18^. The most extensively studied *o*-carborane, which shows a highly symmetrical 3D structure and extraordinary versatility^19–22^, can be transformed to chiral-at-cage molecules with the addition of substituents to lower the symmetry of its icosahedral structure^23–27^. In view of the rapidly developed transition-metal-catalyzed regioselective *o*-carborane B–H bond activation methodologies^28–34^, controlled introduction of a substituent at either cage B(4) or B(5) of C(1)-substituted *o*-carboranes would be possible, resulting in the chirality of the resultant molecules (Fig. 1)^35^. Inspired by the recent reports on fullerene cage chirality^36–39^ and ferrocene planar chirality^40^, the transition-metal-catalyzed enantioselective B–H functionalization should realize the synthesis of optically pure chiral-at-cage *o*-carborane derivatives, which are important in the fields of asymmetric synthesis, materials science, and medicinal chemistry, where the chirality plays an important role in molecular design. We have very recently reported a proof-of-concept study on enantioselective synthesis of chiral-at-cage *o*-carborane derivatives via a Pd-catalyzed intramolecular B(5)–H arylation of *o*-carboranes in the presence of chiral phosphine ligands^41^.

Herein, we report an intermolecular catalytic asymmetric B−H functionalization of *o*-carboranes with the assistance of a directing group and chiral phosphoramidite ligand. This protocol allows easy access to chiral-at-cage *o*-carborane derivatives in high yields and excellent enantioselectivities via Ir-catalyzed enantioselective B−H alkenylation under mild reaction conditions. It illustrates an important application of asymmetric synthesis beyond conventional organic chemistry into the chemistry of chiral boron cages.

## Results and discussion

### Reaction development and optimization

We initially examined the regioselectivity of the reaction using 1-acetylamino-2-methyl-*o*-carborane (**1a**) and diphenylacetylene (**2a**) as model coupling partners and [Cp*IrCl_2_]_2_ as a precatalyst. Reaction of **1a** with 2 equiv of **2a** in toluene at 80 °C in the presence of 5 mol% [Cp*IrCl_2_]_2_, 25 mol% AgNTf_2_, 2 equiv of Cu(OAc)_2_, and 5 equiv of PhCO_2_H afforded the desired B(4/5)-alkenylation product **3aa** as a racemate in 83% isolated yield with complete regioselectivity (B(4/5) vs. B(3/6)) (Table 1, entry 1). Two enantiomers were able to be separated by HPLC on the Chiralpak IA column (see Supplementary Fig. 283). To evaluate the feasibility of asymmetric B−H alkenylation, we then evaluated the role of chiral ligands, and the results were compiled in Table 1. Bisphosphine ligand (*S*)-BINAP **L1** deactivated the catalyst, whereas monophosphine ligand (*R*)-MOP **L2** and (*S*)-BI-DIME **L3** accelerated the alkenylation to yield **3aa** quantitatively (Table 1, entries 2–4) with no enantioselectivity. Among a series of chiral phosphoramidite ligands **L4**–**L14** examined, reactions with dibenz[b,f]azepine containing **L8**–**L14** provided promising results (Table 1, entries 5–15). **L8**, introduced by the Carreira group^42^, proved to be the most efficient chiral ligand in terms of enantioselectivity and reactivity, giving (*S*)-**3aa** in 72% isolated yield with 80% ee (Table 1, entry 9). The use of a stronger acid, phenylsulfonic acid, as an additive, improved the reaction efficiency (Table 1, entry 16). It was later found that the cage C-benzylated substrate, 1-acetylamino-2-benzyl-*o*-carborane (**1b**), significantly enhanced the enantioselectivity to 93% ee (Table 1, entry 17).

**Table 1:** Screening of chiral ligands and additives^a^.


Entry	R	L*	Acid additive	(*S*)-3 (%)^b^	ee (%)
1	Me	–	PhCO_2_H	83	rac
2	Me	**L1**	PhCO_2_H	N.R.	–
3	Me	**L2**	PhCO_2_H	99	rac
4	Me	**L3**	PhCO_2_H	99	rac
5	Me	**L4**	PhCO_2_H	40	rac
6	Me	**L5**	PhCO_2_H	90	rac
7	Me	**L6**	PhCO_2_H	N.R.	–
8	Me	**L7**	PhCO_2_H	N.R.	–
9	Me	**L8**	PhCO_2_H	72	80
10	Me	**L9**	PhCO_2_H	77	77
11	Me	**L10**	PhCO_2_H	99	−24
12^c^	Me	**L11**	PhCO_2_H	42	6
13	Me	**L12**	PhCO_2_H	99	−3
14	Me	**L13**	PhCO_2_H	66	81
15	Me	**L14**	PhCO_2_H	79	−69
16	Me	**L8**	C_6_H_5_SO_3_H	99	82
17	Bn	**L8**	C_6_H_5_SO_3_H	99	93


*ee* enantiomeric excess.

^a^Reactions were conducted on 0.1 mmol scale in 2 mL of toluene. [Cp*IrCl_2_]_2_ and L* were dissolved in 1 mL of toluene and stirred at r.t. for 1 h in advance.

^b^Isolated yields.

^c^6 days.

After a systematic evaluation of reaction parameters such as solvents, acids, [Ag], and [Cu] additives (see Supplementary Tables 1–3 for detail), the asymmetric intermolecular alkenylation of **1b** proceeded smoothly in toluene under the “normal” conditions (Table 2, entry 1), giving (*S*)-**3ba** in 99% yield and 94% ee. Further screening of the catalysts indicated that Pd(OAc)_2_ provided the product in high yield with poor enantioselectivity, whereas [Cp*RhCl_2_]_2_ and IrCl_3_ showed no catalytic activity (Table 2, entries 2–4). To our surprise, improved enantioselectivity of 97% ee was observed with [Ir(COD)Cl]_2_ as an Ir precursor in the absence of Cu(OAc)_2_ (Table 2, entries 5–6). Lowering the reaction temperature to 50 °C resulted in an increased ee to 99% (Table 2, entry 7). The 2.5 mol% catalyst loading also worked very well (Table 2, entry 9). It was noted that a complete reversal of the enantioselectivity was observed with (*R*)-**L8** ligand (Table 2, entry 10).

**Table 2:** Optimization of Ir-catalyzed asymmetric B–H alkenylation^a^.


Entry	Variations from the “normal” conditions	(*S*)-3ba (%)^b^	ee (%)
1	–	99	94
2	5 mol% [Cp*RhCl_2_]_2_ as catalyst	N.R.	–
3	10 mol% IrCl_3_ as catalyst	N.R.	–
4	10 mol% Pd(OAc)_2_ as catalyst	93	4
5	5 mol% [Ir(COD)Cl]_2_ as catalyst	99	97
6	5 mol% [Ir(COD)Cl]_2_, without Cu(OAc)_2_	98	97
7	5 mol% [Ir(COD)Cl]_2_, without Cu(OAc)_2_, 50 °C	99	99
8	5 mol% [Ir(COD)Cl]_2_, without Cu(OAc)_2_, r.t.	N.R.	–
9	[Ir(COD)Cl]_2_ (2.5 mol%), (*S*)-**L8** (5 mol%), AgNTf_2_ (12.5 mol%), without Cu(OAc)_2_, 50 °C	99	99
10	[Ir(COD)Cl]_2_ (2.5 mol%), (*R*)-**L8** (5 mol%), AgNTf_2_ (12.5 mol%), without Cu(OAc)_2_, 50 °C	99	−99

*ee* enantiomeric excess.

^a^Reactions were conducted on 0.1 mmol scale in 2 mL of toluene. [Ir(COD)Cl]_2_ and **L8** were dissolved in 1 mL of toluene and stirred at r.t. for 1 h in advance. ^b^Isolated yields.

**Fig. 2:**
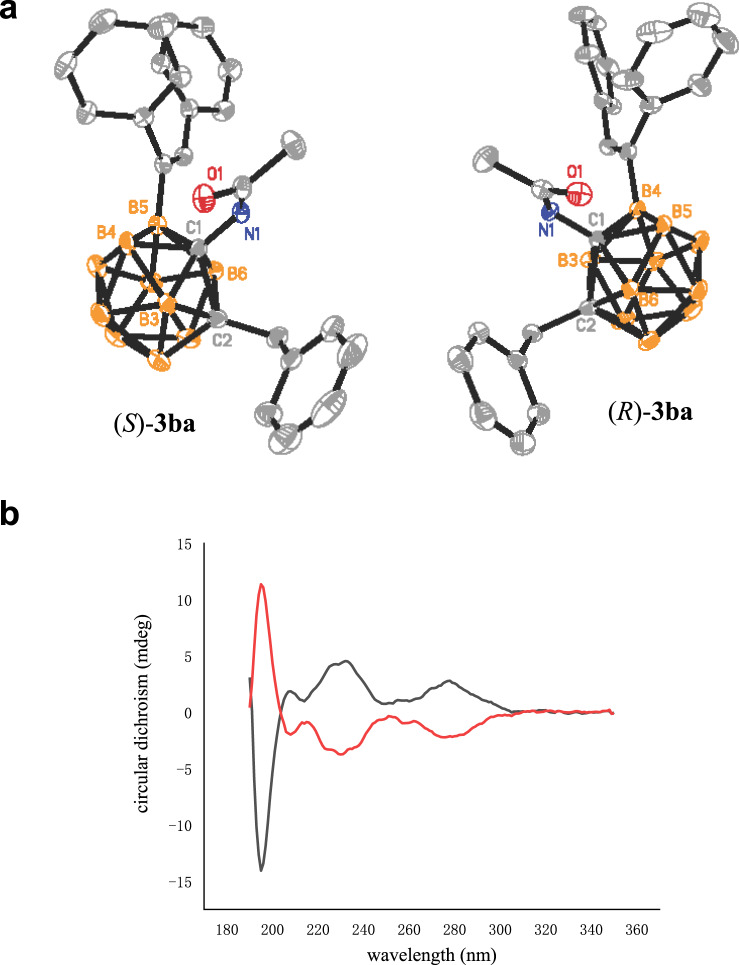
The chirality of B(4/5)-alkenylated *o*-carborane. **a** Molecular structures of (*S*)-**3ba** and (*R*)-**3ba**. **b** CD Spectra of (*S*)-**3ba** (black) and (*R*)-**3ba** (red) in MeCN (*c* = 0.1 mg/mL).

The circular dichroism (CD) spectra of (*R*)-**3ba** and (*S*)-**3ba** exhibited unambiguously mirror images to each other, indicating a pair of enantiomers. The absolute configurations of (*R*)-**3ba** and (*S*)-**3ba** were determined via single-crystal X-ray analyses (Fig. 2). In addition, the single-crystal X-ray structure and CD spectrum of (*S*)-**3bd** (see the Supplementary Information for detail) further confirm the assignment of the absolute configurations for (*R*)-**3ba** and (*S*)-**3ba**.

### Substrate scope

**Fig. 3:**
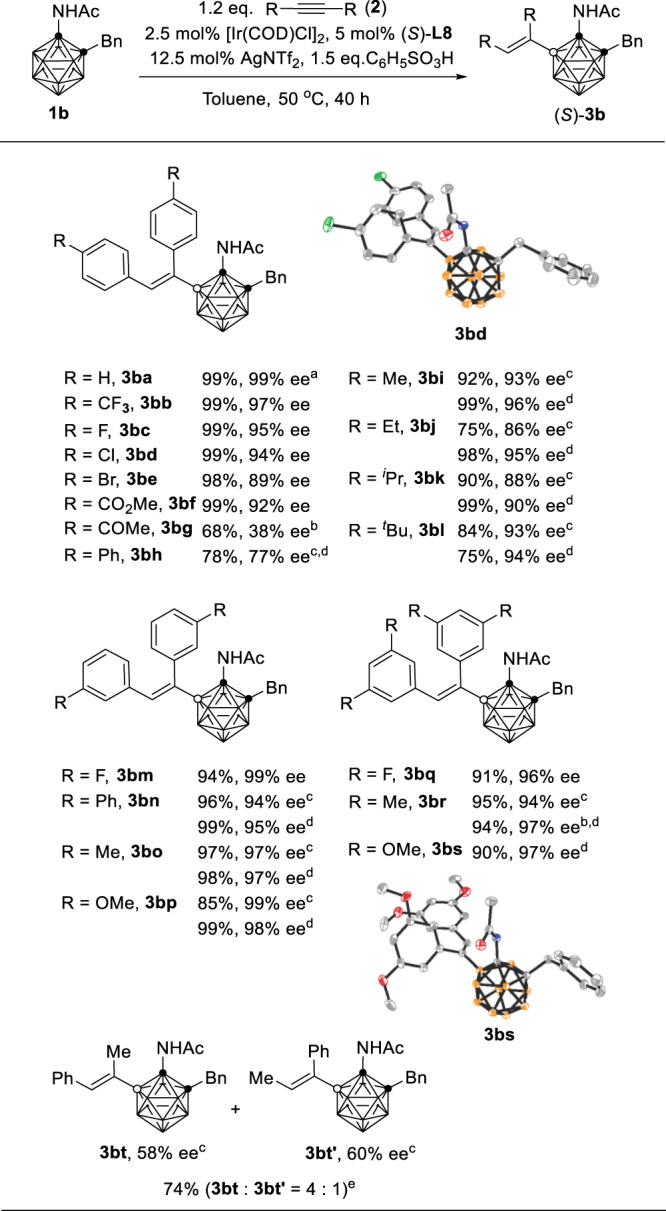
Alkyne substrates scope and molecular structures of (*S*)-3bd and (*S*)-3bs. General conditions: **1b** (0.1 mmol), **2** (0.12 mmol), 2.5 mol% [Ir(COD)Cl]_2_, 5 mol% (*S*)-**L8**, 12.5 mol% AgNTf_2_, 1.5 equiv of C_6_H_5_SO_3_H in 2 mL of toluene, 50 °C, 40 h. [Ir(COD)Cl]_2_ and (*S*)-**L8** was dissolved in 1 mL of toluene and stirred at r.t. for 1 h in advance; isolated yields. ee enantiomeric excess. ^a^12 h. ^b^4 days. ^c^80 °C. ^d^1.1 eq. of Cu(OAc)_2_ was added. ^e^The ratio was determined by ^1^H NMR.

Under the optimized reaction conditions (Table 2, entry 9), the alkyne substrate scope was then examined and the results were summarized in Fig. 3. Generally, diaryl acetylenes with electron-withdrawing groups such as –F, –Cl, –Br, –CF_3,_ and –CO_2_Me worked very well, affording (*S*)-**3bb**–**3bf**, **3bm,** and **3bq** in excellent yields with 89–99% ee. *p*-Acyl and *p*-phenyl were also tolerated with low conversions and enantioselectivities (**3bg**, **3bh**). For the electron-donating group containing substrates, a higher reaction temperature of 80 °C was required to promote the reaction, leading to slightly decreased enantioselectivity. It was found that the addition of 1.1 equiv of Cu(OAc)_2_ to the above reactions could not only accelerate the reaction but also improve the enantioselectivity (**3bi**–**3bk**, **3bn**–**3bp**, **3br**, **3bs**) with a lower reaction temperature probably due to the activation of alkynes via their complexation with Cu(II) salt. Steric factors also played a role as di-*o*-tolylacetylene was not compatible. Unsymmetrical alkyne MeC≡CPh gave two regioisomers of **3bt** and **3bt**′ in a 4:1 ratio with 58% and 60% ee, respectively.

**Fig. 4:**
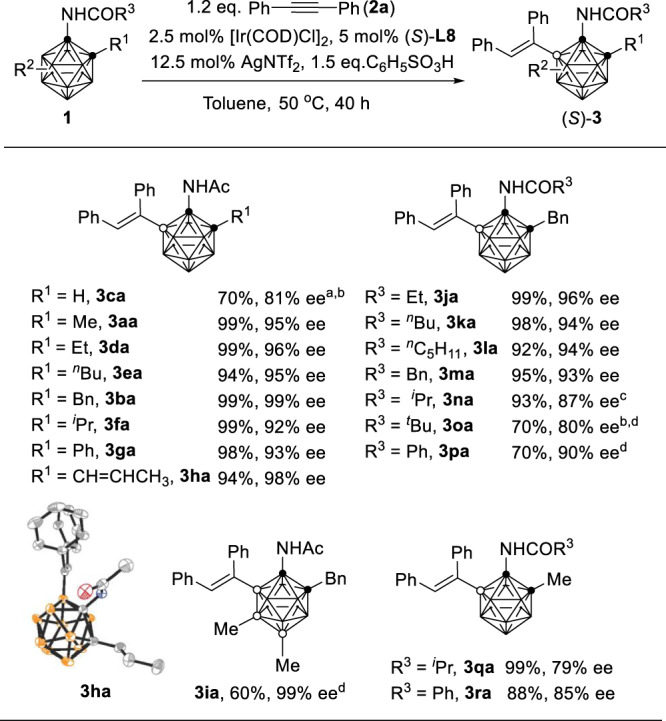
*o*-Carborane substrates scope and molecular structure of (*S*)-3ha. General conditions: **1** (0.1 mmol), **2** (0.12 mmol), 2.5 mol% [Ir(COD)Cl]_2_, 5 mol% (*S*)-**L8**, 12.5 mol% AgNTf_2_, 1.5 equiv of C_6_H_5_SO_3_H in 2 mL of toluene, 50 °C, 40 h. [Ir(COD)Cl]_2_ and (*S*)-**L8** was dissolved in 1 mL of toluene and stirred at r.t. for 1 h in advance; isolated yields. ee enantiomeric excess. ^a^12 h. ^b^80 °C. ^c^4 days. ^d^1 week.

For the scope of *o*-carboranes (Fig. 4), cage C substituent R^1^ does not have an obvious impact on the reactions except for R^1^ = H (**3ca**), affording the corresponding (*S*)-B(5)-alkenylated compounds (**3aa**, **3ba**, **3da**–**3ha**) in high yields (94−99%) with excellent enantioselectivity (92−99% ee). For B(9,12)-dimethylated *o*-carborane, a lower alkenylation efficiency was observed, but the enantioselectivity remained unchanged (**3ia**; 60% yield, 99% ee). On the other hand, bulky substituent R^3^ on the amide group resulted in slightly low yields and enantioselectivities (**3na**–**3ra**).

**Fig. 5:**
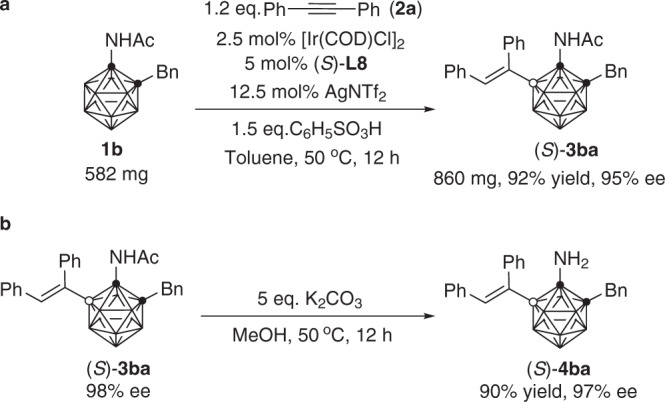
Large-scale synthesis of (*S*)-3ba and its transformation. **a** Large-scale synthesis of (*S*)-**3ba**. **b** Deacylation of (*S*)-**3ba**.

A large-scale synthesis of (*S*)-**3ba** was subsequently carried out (Fig. 5a). Under the optimal reaction conditions, treatment of **1b** (582 mg, 2.0 mmol) with 1.2 equiv of diphenylacetylene (428 mg, 2.4 mmol) in the presence of 2.5 mol% [Ir(COD)Cl]_2_, 5 mol% (*S*)-**L8**, 12.5 mol% AgNTf_2_ and 1.5 equiv of C_6_H_5_SO_3_H in toluene (20 mL) afforded (*S*)-**3ba** (860 mg, 92% isolated yield) with 95% ee. In addition, the product (*S*)-**3ba** (98% ee) was readily converted to synthetically valuable amine by treatment with K_2_CO_3_ in methanol, furnishing (*S*)-1-NH_2_-2-Bn-5-[C(Ph)=CH(Ph)]-*o*-C_2_B_10_H_9_ ((*S*)-**4ba**) in 90% yield with 97% ee (Fig. 5b).

Compounds **3** and **4** were fully characterized by ^1^H-, ^13^C- and ^11^B-NMR spectroscopy, as well as high-resolution mass spectrometry. The molecular structures of **3bd**, **3bs,** and **3ha** were further confirmed by single-crystal X-ray analyses.

### Mechanistic investigations

**Fig. 6:**
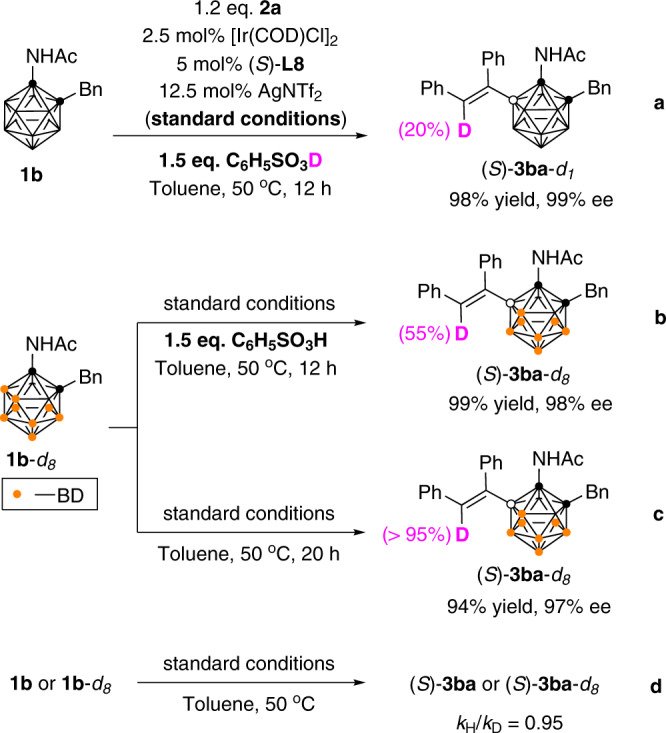
Mechanistic investigations. Control experiments. **a** Reaction of **1b** using C_6_H_5_SO_3_D as the acid additive. **b** Reaction of **1b**-*d*_8_ using C_6_H_5_SO_3_H as the acid additive. **c** Reaction of **1b** without acid additive. **d** Independent-rate KIE experiments.

To gain some insight into the reaction mechanism, several control experiments were conducted. Under standard reactions, using C_6_H_5_SO_3_D as the acid additive gave (*S*)-**3ba**-*d*_1_ in 98% yield and 99% ee with 20% D-incorporation (Fig. 6a). On the other hand, treatment of **1b**-*d*_8_ with C_6_H_5_SO_3_H resulted in a 55% D-incorporation, suggesting some D–H exchange with the acid (Fig. 6b). In the absence of C_6_H_5_SO_3_H additive, (*S*)-**3ba**-*d*_8_ with >95% D-incorporation was obtained, indicating that the deuterium was originated from B(5)-D, and no deuterium scrambling over carborane was observed (Fig. 6c). To gain additional information regarding the initial rate of the reaction, parallel reactions using substrate **1b** and **1b**-*d*_8_ were conducted, leading to the kinetic isotope effect of *k*_H_/*k*_D_ = 0.95 (Fig. 6d; see Supplementary Figs. 11 and 12 for detail), which indicates that B−H activation is not involved in the rate-determining step.

**Fig. 7:**
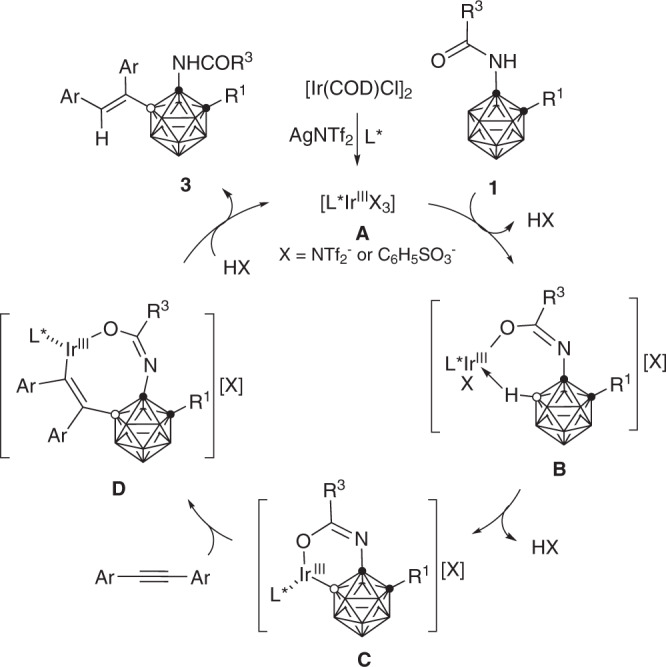
Proposed catalytic cycle. Possible reaction mechanism of the Ir-catalyzed enantioselective B−H alkenylation. The chiral ligand on Ir has been marked as L* for clarity.

As the Ir(I) can be oxidized by Ag(I) to generate in-situ active catalyst Ir(III)^43^ that performs even better than [Cp*IrCl_2_]_2_ (Table 2), a plausible reaction mechanism is proposed in Fig. 7. The catalysis is initiated by the Ir(III) generated in situ via the oxidation of [Ir(COD)Cl]_2_ with Ag^+^ in the presence of chiral phosphoramidite ligand, followed by the coordination with acetylamino-*o*-carborane **1** in an iminium form to offer the Ir(III) intermediate **B**. Subsequent selective electrophilic B(5)–H metalation^32,44^ (for C–H metalation, see ref. ^44^) and alkyne insertion afford the intermediate **D** that undergoes protonation to give the final product **3**.

**Fig. 8:**
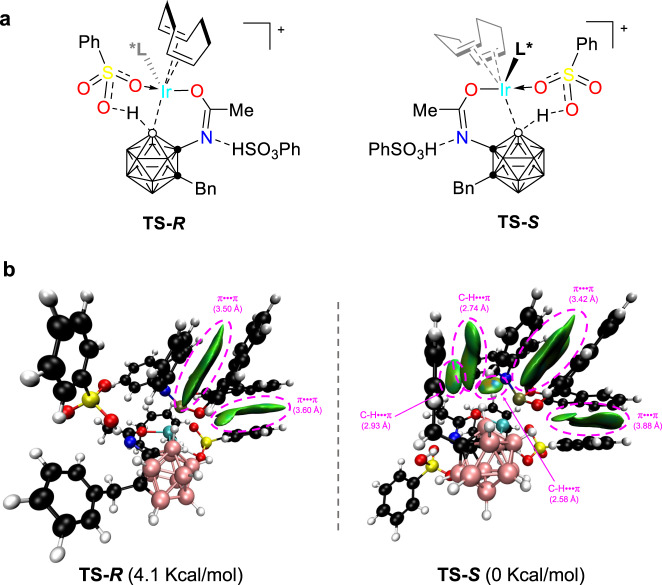
DFT calculated transition states. **a** Structures of the two transition states **TS-*R*** and **TS-*S*** from the intermediates **B** to **C**. **b** NCI analysis for the enantioselective transition states **TS-*R*** and **TS-*S***. Values in parentheses are distances between two interactive fragments in Å.

In the reaction, cage B(4/5) regioselectivity is dominated by the combination of the Ir(III) catalyst and the directing group^31,33^, whereas the enantioselectivity is controlled by the chiral phosphoramidite ligand^40^. (*S*)- or (*R*)-**L8** leads to (*S*)- or (*R*)-enantiomer, respectively. To shed some light on the enantioselectivity in the current asymmetric B−H functionalization, the transition states **TS-*R*** and **TS-*S*** leading to the final alkenylation products in *R* and *S* configuration, respectively, were located by DFT calculations on the basis of concerted metalation-deprotonation (CMD) mechanism (Fig. 8). The B−Ir bond-forming step (from the intermediates **B** to **C**) was identified as the stereoselectivity-determining step, leading to the preferentially generated (*S*)-B(5)-alkenylation product. The transition state **TS-*S*** was calculated to be more stable than its enantiomer **TS-*R*** by 4.1 kcal/mol. Non-covalent interactions (NCI) analysis, which has been successful to identify electrostatic interactions, was performed using Multiwfn software^45,46^ to gain further insight into the key factors that control stereoselectivity. The NCI pictures show that the π···π interactions exist in both isomers with nearly equal contributions (Fig. 8b). The C-H···π interactions only exist in **TS-*S***. These additional interactions would be responsible for stabilizing the transition state **TS-*S***.

In summary, the first intermolecular asymmetric B−H functionalization has been developed via Ir catalysis for the enantioselective synthesis of chiral-at-cage *o*-carboranes under mild reaction conditions. Generally very good to excellent yields with up to 99% ee can be achieved in this Ir-catalyzed B−H alkenylation. The enantiocontrol model is proposed based on DFT calculations in which the use of chiral phosphoramidite ligand is essential for such asymmetric *o*-carborane B−H functionalization. This work sets a good example for exploring the potential of asymmetric synthesis beyond conventional organic chemistry into the chemistry of chiral-at-cage *o*-carboranes.

## Methods

### A representative procedure for the synthesis of 3

An oven-dried Schlenk flask equipped with a stir bar was charged with [Ir(cod)Cl]_2_ (1.7 mg, 0. 0025 mmol) and (*S*)-**L8** (2.6 mg, 0.005 mmol), followed by dry toluene (1 mL). The mixture was stirred at room temperature for 1 h, to which was successively added **1** (0.10 mmol), **2** (0.12 mmol), benzenesulfonic acid (24 mg, 0.15 mmol), AgNTf_2_ (4.9 mg, 0.0125 mmol), and dry toluene (1 mL). The flask was closed under an atmosphere of nitrogen, then stirred at 50 °C for 40 h. After hydrolysis with water (5 mL) and extraction with diethyl ether (10 mL × 3), the ether solutions were combined, dried over anhydrous Na_2_SO_4,_ and concentrated to dryness in vacuo. The residue was subjected to flash column chromatography on silica gel (230–400 mesh) using *n*-hexane and ethyl acetate (4/1 in v/v) as eluent to give the product (*S*)-**3**. The synthetic protocol and the characterization of compounds **1**, **3**, and **4** can be found in the  Supplementary Information.

## Supplementary information

Supplementary Information

Description of Additional Supplementary Files

Dataset 1

Dataset 2

## Data Availability

CCDC 2081461-2081465 ((*S*)-**3ba**, (*R*)-**3ba**, (*S*)-**3bd**, (*S*)-**3bs** and (*S*)-**3ha**) contain the supplementary crystallographic data for this paper. These data can be obtained free of charge from The Cambridge Crystallographic Data Centre via www.ccdc.cam.ac.uk/data_request/cif.

## References

[1] Corey, E. J. & Kürti, L. *Enantioselective Chemical Synthesis* (Academic Press, 2010).

[2] Christmann, M. & Bräse, S. *Asymmetric Synthesis II* (Wiley-VCH, 2012).

[3] Zhou, Q.-L. *Privileged Chiral Ligands and Catalysts* (Wiley-VCH, 2011).

[4] Lin, G.-Q., Li, Y.-M. & Chan, A. S. C. *Principles and Applications of Asymmetric Synthesis* (John Wiley & Sons, 2002).

[5] Newton CG, Wang S-G, Oliveira CC, Cramer N (2017). Catalytic enantioselective transformations involving C–H bond cleavage by transition-metal complexes. Chem. Rev..

[6] Krasnov VP (2002). Enantiomers of 3-amino-1-methyl-1,2-dicarba-closo-dodecaborane. Tetrahedron: Asymmetry.

[7] Levit GL (2005). Acidic hydrolysis of N-acyl-1-substituted 3-amino-1,2-dicarba-closo-dodecaboranes. J. Organomet. Chem..

[8] Levit GL (2007). Synthesis of N-[(3-amino-1,2-dicarba-closo-dodecaboran-1-yl)acetyl] derivatives of α-amino acids. Collect. Czech. Chem. Commun..

[9] Krasnov VP (2008). Determination of enantiomeric purity of 1-substituted 3-amino-1,2-dicarba-closo-dodecaboranes by HPLC on chiral stationary phases. Russ. Chem. Bull..

[10] Fisher SP (2019). Nonclassical applications of closo-carborane anions: from main group chemistry and catalysis to energy storage. Chem. Rev..

[11] Núñez R, Tarrés M, Ferrer-Ugalde A, de Biani FF, Teixidor F (2016). Electrochemistry and photoluminescence of icosahedral carboranes, boranes, metallacarboranes, and their derivatives. Chem. Rev..

[12] Hawthorne MF, Maderna A (1999). Applications of radiolabeled boron clusters to the diagnosis and treatment of cancer. Chem. Rev..

[13] Scholz M, Hey-Hawkins E (2011). Carbaboranes as pharmacophores: properties, synthesis, and application strategies. Chem. Rev..

[14] Leśnikowski ZJ (2016). Challenges and opportunities for the application of boron clusters in drug design. J. Med. Chem..

[15] Xie Z (2002). Advances in the chemistry of metallacarboranes of *f*-block elements. Coord. Chem. Rev..

[16] Hosmane, N. S. & Maguire, J. A. in *Comprehensive Organometallic Chemistry III* (eds Crabtree, R. H. & Mingos, D. M. P.) Ch. 5 (Elsevier, Oxford, 2007).

[17] Yao Z-J, Jin G-X (2013). Transition metal complexes based on carboranyl ligands containing N, P, and S donors: synthesis, reactivity and applications. Coord. Chem. Rev..

[18] Fisher SP, Tomich AW, Guo J, Lavallo V (2019). Teaching an old dog new tricks: new directions in fundamental and applied closo-carborane anion chemistry. Chem. Commun..

[19] Grimes, R. N. *Carboranes* 3rd edn (Elsevier, 2016).

[20] Hosmane NS (2011). Boron Science: New Technologies and Applications.

[21] Grimes RN (2015). Carboranes in the chemist’s toolbox. Dalton Trans..

[22] Zheng F, Yui TH, Zhang J, Xie Z (2020). Synthesis and X-ray characterization of 15- and 16-vertex *closo*-carboranes. Nat. Commun..

[23] King RB (1987). Chemical applications of topology and group theory. XXII: lowest degree chirality polynomials for regular polyhedra. J. Math. Chem..

[24] Usatov, A. V. et al. Fullerene and carborane in one coordination sphere: synthesis and structure of a mixed η^2^-C_60_ and σ-carboranyl complex of iridium. *Eur. J. Inorg. Chem*. 2565–2567 (2002).

[25] Smolyakov, V. M., Sokolov, D. V., Nilov, D. Y., Grebeshkov, V. V. & Fedin, D. M. *Carbon Nanomaterials in Clean Energy Hydrogen Systems*—II Ch. 31 (Springer, 2011).

[26] Gona KB, Gomez-Vallejo V, Padro D, Llop J (2013). [^18^F]Fluorination of *o*-carborane via nucleophilic substitution: towards a versatile platform for the preparation of ^18^F-labelled BNCT drug candidates. Chem. Commun..

[27] Dziedzic RM (2017). Cage-walking: vertex differentiation by palladium-catalyzed isomerization of B(9)-bromo-meta-carborane. J. Am. Chem. Soc..

[28] Olid D, Nunez R, Vinas C, Teixidor F (2013). Methods to produce B–C, B–P, B–N and B–S bonds in boron clusters. Chem. Soc. Rev..

[29] Yu W-B, Cui P-F, Gao W-X, Jin G-X (2017). BH activation of carboranes induced by late transition metals. Coord. Chem. Rev..

[30] Quan Y, Qiu Z, Xie Z (2018). Transition-metal-catalyzed selective cage B−H functionalization of *o*-carboranes. Chem. Eur. J..

[31] Dziedzic RM, Spokoyny AM (2019). Metal-catalyzed cross-coupling chemistry with polyhedral boranes. Chem. Commun..

[32] Quan Y, Xie Z (2019). Controlled functionalization of o-carborane via transition metal catalyzed B–H activation. Chem. Soc. Rev..

[33] Zhang X, Yan H (2019). Transition metal-induced B–H functionalization of o-carborane. Coord. Chem. Rev..

[34] Au YK, Xie Z (2021). Recent advances in transition metal-catalyzed selective B–H functionalization of *o*-carboranes. Bull. Chem. Soc. Jpn..

[35] Plešek J, Gregor V, Heřmánek S (1970). Chemistry of boranes. XX. Optical isomerism in the o-carborane series. Collect. Czech. Chem. Commun..

[36] Thilgen C, Diederich F (2006). Structural aspects of fullerene chemistry a journey through fullerene chirality. Chem. Rev..

[37] Nambo M, Wakamiya A, Itami K (2012). Palladium-catalyzed tetraallylation of C_60_ with allyl chloride and allylstannane: mechanism, regioselectivity, and enantioselectivity. Chem. Sci..

[38] Maroto EE (2014). Chiral fullerenes from asymmetric catalysis. Acc. Chem. Res..

[39] Rickhaus M, Mayor M, Juricek M (2017). Chirality in curved polyaromatic systems. Chem. Soc. Rev..

[40] Gao D-W, Gu Q, Zheng C, You S-L (2017). Synthesis of planar chiral ferrocenes via transition-metal-catalyzed direct C–H bond functionalization. Acc. Chem. Res..

[41] Cheng R (2018). Enantioselective synthesis of chiral-at-cage *o*-carboranes via Pd-catalyzed asymmetric B–H substitution. J. Am. Chem. Soc..

[42] Rossler SL, Petrone DA, Carreira EM (2019). Iridium-catalyzed asymmetric synthesis of functionally rich molecules enabled by (phosphoramidite,olefin) ligands. Acc. Chem. Res..

[43] Fu R, Bercaw JE, Labinger JA (2011). Intra- and intermolecular C–H activation by bis(phenolate)pyridineiridium(III) complexes. Organometallics.

[44] Snieckus, V. Directed *ortho* metalation. Tertiary amide and *O*-carbamate directors in synthetic strategies for polysubstituted aromatics. *Chem. Rev*. **90**, 879–933 (1990).

[45] Lu T, Chen F (2012). Multiwfn: a multifunctional wavefunction analyzer. J. Comput. Chem..

[46] Wang Y, Qu L-B, Lan Y, Wei D (2020). Origin of regio- and stereoselectivity in the NHC-catalyzed reaction of alkyl pyridinium with aliphatic enal. ChemCatChem.

