# Supplementary material for: Ir-catalyzed enantioselective B−H alkenylation for asymmetric synthesis of chiral-at-cage o‑carboranes
Source: Nat Commun. 2021 Dec 8;12:7146. doi: 10.1038/s41467-021-27441-y (PMC8654863; doi:10.1038/s41467-021-27441-y)
Supplement: Supplementary file 1 — Supplementary Information [file 41467_2021_27441_MOESM1_ESM.pdf]

Supplementary Information

Ir-Catalyzed Enantioselective B−H Alkenylation for Asymmetric Synthesis of Chiral-at-Cage *o*-Carboranes

Ruofei Cheng,[1] Jie Zhang,[2] Huifang Zhang,[1,3] Zaozao Qiu,*[,1,4] and Zuowei Xie*[,1,2]

[1]Shanghai-Hong Kong Joint Laboratory in Chemical Synthesis, Shanghai Institute of Organic Chemistry, University of Chinese Academy of Sciences, Chinese Academy of Sciences, 345 Lingling Rd, Shanghai 200032, China

[2]Department of Chemistry and State Key Laboratory of Synthetic Chemistry, The Chinese University of Hong Kong, Shatin, N. T., Hong Kong, China

[3]School of Chemistry and Chemical Engineering, Henan Normal University, Xinxiang, Henan 453007, China

[4]CAS Key Laboratory of Energy Regulation Materials, Shanghai Institute of Organic Chemistry, Chinese Academy of Sciences, 345 Lingling Rd, Shanghai 200032, China

Table of Contents

Supplementary Methods S2

Mechanistic Study S36

Crystal Data and Summary of Data Collection and Refinements S43

Computational Details S46

NMR Spectra S47

Chiral HPLC Traces S264

Supplementary References S320

Supplementary Methods

General Procedures. All reactions were carried out in oven-dried glassware under an atmosphere of dry N_2 with the rigid exclusion of air and moisture using standard Schlenk techniques or in a glovebox. Organic solvents were freshly distilled from sodium benzophenone ketyl immediately prior to use. All other chemicals were purchased from either Aldrich or J&K Chemical Co. and used as received unless otherwise specified. ^{1}H, ^{13}C and ^{19}F NMR spectra were recorded on a Varian Inova or a Bruker 400 spectrometer at 400, 101 and 376 Hz, respectively. ^{11}B NMR spectra were recorded on a Bruker 400 spectrometer at 128 MHz. All chemical shifts were reported in ppm unit with references to the residual solvent resonances of the deuterated solvents for proton and carbon chemical shifts, to external $BF_3{\cdot}OEt_2$ (0.00) for boron chemical shifts and to external $CFCl_3$ (0.00) for fluorine chemical shifts. Mass spectra were obtained on a Thermo Fisher Scientific LTQ FTICR-MS spectrometer. Circular Dichroism spectra were recorded on a Chirascan cicular dichroism spectrometer (Applied Photophysics Ltd., Leatherhead, UK). Enantiomeric excess was determined by HPLC analysis using the corresponding commercial chiral column as stated in the experimental procedures at 25 ^{o}C with UV detector at 214 or 254 nm. Single crystal X-ray analyses were performed on SMART APEX CCD X-ray single crystal diffractometer. Specific rotation was measured on the Autopol I Polarimeter at 589 nm. The melting points of solid compounds were determined by the melting point apparatus (Shanghai INESA Physico-Optical Instrument Co., LTD). IR spectra were recorded on a Nicolet 380FT-IR infrared spectrophotometer.

Optimization of Enantioselective Reaction Conditions.

Supplementary Table 1 Optimization of Ir-Catalyzed Intermolecular Asymmetric Alkenylation Conditions Using $[Cp^*IrCl_2]_2$ as Precatalyst[a]

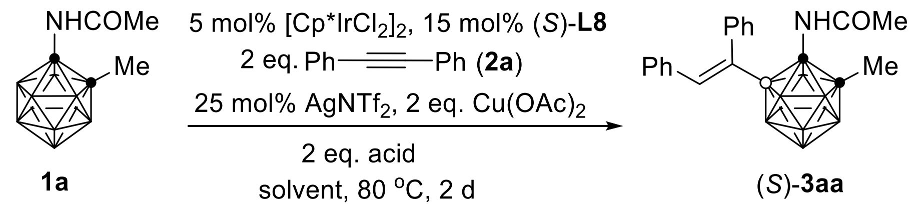

Entry	Acid	Solvent	(*S*)-**3aa** (%)[b]	ee (%)
1[c]	PhCOOH	HFIP	17	-
2	PhCOOH	C_6H_5Cl	90	71
3[d]	PhCOOH	C_6H_5F	90	80
4	PhCOOH	toluene	72	80
5[e]	PhCOOH	toluene	50	39
6[f]	PhCOOH	toluene	81	50
7	$C_6H_5SO_3H$	toluene	99	82
8	*p*-Me-$C_6H_4SO_3H$	toluene	99	84
9	*p*-F-$C_6H_4SO_3H$	toluene	99	82
10	CF_3SO_3H	toluene	N.P.[g]	-

[a]Reactions were conducted on 0.1 mmol scale in 2 mL of solvent. $[Cp^*IrCl_2]_2$ and (*S*)-**L8** were dissolved in 1 mL of solvent and stirred at r.t. for 1 h in advance. [b]Isolated yields. [c]4 days. [d]5 days. [e]$[Cp^*IrCl_2]_2$, (*S*)-**L8** and $AgNTf_2$ were dissolved in 1 mL of solvent and stirred at r.t. for 1 h in advance. [f]All reagents were dissolved in 2 mL of toluene together. [g]**1a** deacetylation occurred.

Supplementary Table 2 Optimization of Ir-Catalyzed Intermolecular Asymmetric Alkenylation Conditions Using $[Cp^*IrCl_2]_2$ as Precatalyst[a]

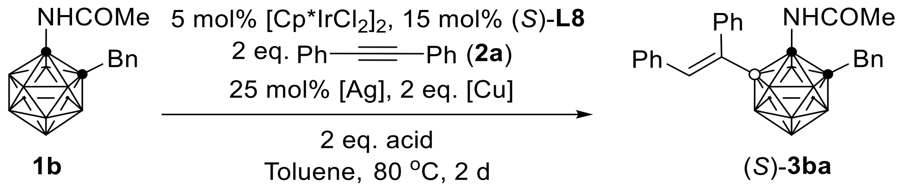

Entry	Acid	[Ag]	[Cu]	(*S*)-**3ba** (%)[b]	ee (%)
1	*p*-Me-$C_6H_4SO_3H$	$AgNTf_2$	$Cu(OAc)_2$	99	93
2	*p*-F-$C_6H_4SO_3H$	$AgNTf_2$	$Cu(OAc)_2$	99	93
3	$C_6H_5SO_3H$	$AgNTf_2$	$Cu(OAc)_2$	99	93
4	$C_6H_5SO_3H$	$AgNTf_2$	-	N.R.	-
5	$C_6H_5SO_3H$	$AgNTf_2$	$Cu(OTf)_2$	N.R.	-
6	$C_6H_5SO_3H$	AgOAc	$Cu(OAc)_2$	N.R.	-
7	$C_6H_5SO_3H$	AgOTf	$Cu(OAc)_2$	99	88
8	$C_6H_5SO_3H$	$AgSbF_6$	$Cu(OAc)_2$	78	41
9	$C_6H_5SO_3H$	$AgBF_4$	$Cu(OAc)_2$	99	87

[a]Reactions were conducted on 0.1 mmol scale in 2 mL of toluene. $[Cp^*IrCl_2]_2$ and (*S*)-**L8** were dissolved in 1 mL of toluene and stirred at r.t. for 1 h in advance. [b]Isolated yields.

Supplementary Table 3 Optimization of Transition Metal-Catalyzed Intermolecular Asymmetric Alkenylation Conditions[a]

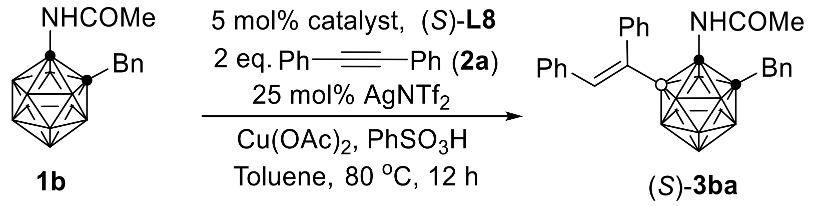

Entry[a]	catalyst	(*S*)-**L8** (mol%)	$Cu(OAc)_2$ (eq.)	$PhSO_3H$ (eq.)	(*S*)-**3ba** (%)[b]	ee (%)
1	$[Cp^*IrCl_2]_2$	15	2	2	99	93
2	$[Cp^*IrCl_2]_2$	10	2	2	99	95
3	$[Cp^*IrCl_2]_2$	10	2	4	N.R.	-
4	$[Cp^*IrCl_2]_2$	10	2	1.5	99	95
5	$[Cp^*IrCl_2]_2$	10	2	1.1	99	92
6	$[Cp^*IrCl_2]_2$	10	1.1	1.5	99	95
7	$[Cp^*IrCl_2]_2$	10	0.5	1.5	N.R.	-
8[c]	$[Cp^*IrCl_2]_2$	10	1.1	1.5	99	94
9[c,d]	$IrCl_3$	10	1.1	1.5	N.R.	-
10[c]	[Ir(COD)Cl]2	10	1.1	1.5	99	97
11[c]	$[Cp^*RhCl_2]_2$	10	1.1	1.5	N.R.	-
12[c,d]	$Pd(OAc)_2$	10	1.1	1.5	93	4

[a]Reactions were conducted on 0.1 mmol scale in 2 mL of toluene. $[Cp^*IrCl_2]_2$ and (*S*)-**L8** were dissolved in 1 mL of toluene and stirred at r.t. for 1 h in advance. [b]Isolated yields. [c]1.2 equiv. **2a**. [d]10 mol% catalyst.

Supplementary Table 4 Optimization of Ir-Catalyzed Intermolecular Asymmetric Alkenylation Conditions Using $[Ir(COD)Cl]_2$ as Precatalyst[a]

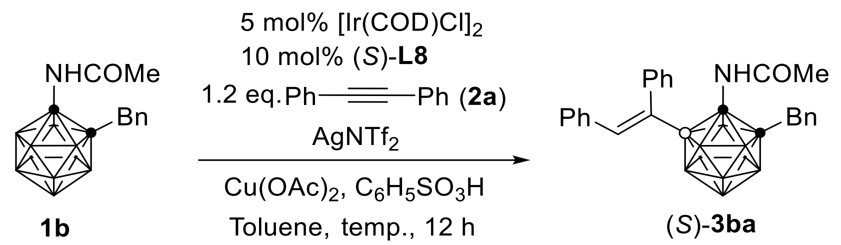

Entry	$AgNTf_2$ (mol%)	$Cu(OAc)_2$ (eq.)	$PhSO_3H$ (eq.)	temp (°C)	time (h)	(*S*)-**3ba** (%)[b]	ee (%)
1	25	1.1	1.5	80	12	99	97
2	-	1.1	1.5	80	12	74	95
3	25	-	1.5	80	12	98	97
4	25	1.1	-	80	12	97	96
5	25	-	-	80	12	98	96
6	25	-	-	50	18	98	97
7	25	-	-	r.t.	12	N.R.	-
8	25	-	1.5	50	12	99	99
9	25	-	1.5	r.t.	12	N.R.	-
10[c]	12.5	-	1.5	50	12	99	99
11[d]	12.5	-	1.5	50	12	N.R.	-

[a]Reactions were conducted on 0.1 mmol scale in 2 mL of toluene. $[Ir(COD)Cl]_2$ and (*S*)-**L8** were dissolved in 1 mL of toluene and stirred at r.t. for 1 h in advance. [b]Isolated yields. [c]2.5 mol% $[Ir(COD)Cl]_2$, 5 mol% (*S*)-**L8**. [d]2.5 mol% $[Ir(COD)Cl]_2$, 10 mol% (*S*)-**L8**.

A Representative Procedure for the Preparation of Starting Materials 1a-s.

According to the literature procedure,[1] to a toluene solution (15 mL) of 1-amino-2-substituted-*o*-carborane[2] (5.0 mmol) was added pyridine (1.2 mL, 15.0 mmol) and acyl chloride (15.0 mmol) successively under an atmosphere of dry nitrogen. The reaction flask was closed and the mixture was stirred at 80 °C for 12 h. After hydrolysis with water (20 mL) and extraction with diethyl ether (20 mL

x 3), the organic portions were combined, dried over anhydrous Na_2SO_4 and concentrated to dryness in vacuo. The residue was subjected to flash column chromatography on silica gel (230-400 mesh) using *n*-hexane and ethyl acetate (3/1 in v/v) as eluent to give the products **1a**-**s**.

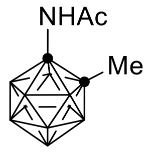

1a

1a: White solid. Yield: 92%. M.p. = 205-207 °C. TLC: R_f = 0.30 (*n*-hexane : ethyl acetate = 3 : 1). ^1H NMR (400 MHz, $CDCl_3$): δ 6.46 (s, 1H) (N*H*), 2.10 (s, 3H), 1.97 (s, 3H) (C*H*$_3$). $^{13}C\{^1H\}$ NMR (101 MHz, $CDCl_3$): δ 168.3 (*C*O), 81.9, 78.3 (cage *C*), 24.3, 22.4 (*C*H$_3$). $^{11}B\{^1H\}$ NMR (128 MHz, $CDCl_3$): δ -5.9 (1B), -6.4 (1B) -10.5 (4B), -11.3 (2B), -12.0 (2B). IR ν (film) cm^{-1}: 3284, 2581, 2555, 1698, 1524, 1268, 594, 526. HRMS (DART) Calcd for $C_5H_{18}{}^{10}B_2{}^{11}B_8NO^+$ [M+H$^+$]: 216.3286. Found: 216.3286.

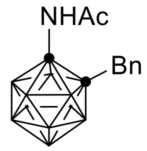

1b

1b: White solid. Yield: 86%. M.p. = 183-185 °C. TLC: R_f = 0.24 (*n*-hexane : ethyl acetate = 4 : 1). ^1H NMR (400 MHz, $CDCl_3$): δ 7.34 (m, 3H), 7.14 (m, 2H) (aromatic C*H*), 6.50 (s, 1H) (N*H*), 3.40 (s, 2H) (C*H*$_2$), 2.16 (s, 3H) (COC*H*$_3$). $^{13}C\{^1H\}$ NMR (101 MHz, $CDCl_3$): δ 168.7 (*C*O), 134.9, 130.3, 128.8, 128.3 (aromatic *C*H), 83.4, 83.1 (cage *C*), 40.5 (*C*H$_2$), 24.3 (CO*C*H$_3$). $^{11}B\{^1H\}$ NMR (128 MHz, $CDCl_3$): δ -5.1 (1B), -5.9 (1B), -10.6 (4B), -11.6 (4B). IR ν (film) cm^{-1}: 3011, 2574, 1688, 1540, 1282, 694, 520. HRMS (DART) Calcd for $C_{11}H_{22}{}^{10}B_2{}^{11}B_8NO^+$ [M+H$^+$]: 292.2699. Found: 292.2698.

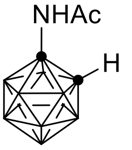

1c

1c: White solid. Yield: 90%. M.p. = 168-170 °C. TLC: R_f = 0.26 (*n*-hexane : ethyl acetate = 4 : 1). ^1H NMR (400 MHz, $CDCl_3$): δ 6.40 (s, 1H) (N*H*), 5.09 (s, 1H) (cage C*H*), 1.99 (s, 3H) (COC*H*$_3$). $^{13}C\{^1H\}$ NMR (101 MHz, $CDCl_3$): δ 168.9 (*C*O), 78.4 (cage *C*), 59.7 (cage *C*H), 23.9 (CO*C*H$_3$). $^{11}B\{^1H\}$ NMR (128 MHz, $CDCl_3$): δ -4.4 (1B), -7.3 (1B), -11.2 (4B), -11.8 (2B), -14.0 (2B). IR ν (film) cm^{-1}: 3239, 3027, 2568, 1667, 1532, 1291, 1010, 592, 520. HRMS (DART) Calcd for $C_4H_{16}{}^{10}B_2{}^{11}B_8NO^+$ [M+H$^+$]: 202.2230. Found: 202.2230.

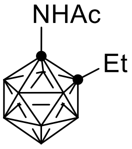

1d

1d: White solid. Yield: 88%. M.p. = 176-178 °C. TLC: R_f = 0.31 (*n*-hexane : ethyl acetate = 3 : 1). ^1H NMR (400 MHz, $CDCl_3$): δ 6.45 (s, 1H) (N*H*), 2.17 (q, *J* = 7.6 Hz, 3H) (C*H*$_2$CH$_3$), 2.09 (s, 3H) (COC*H*$_3$), 1.15 (t, *J* = 7.5 Hz, 3H) (CH$_2$C*H*$_3$). $^{13}C\{^1H\}$ NMR (101 MHz, $CDCl_3$): δ 165.2 (*C*O), 84.5, 83.5 (cage *C*), 27.8, 24.2 (*C*H$_2$ & CO*C*H$_3$) 13.8

(CH_2CH_3). $^{11}B\{^1H\}$ NMR (128 MHz, $CDCl_3$): δ -5.7 (1B), -6.2 (1B), -10.8 (4B), -12.0 (2B), -12.6 (2B). IR ν (film) cm^{-1}: 3273, 2986, 2579, 1688, 1527, 1276, 1038, 524. HRMS (DART) Calcd for $C_6H_{20}{}^{10}B_2{}^{11}B_8NO^+$ [M+H$^+$]: 230.2543. Found: 230.2542.

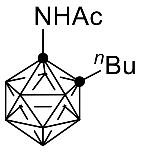

1e

1e: White solid. Yield: 84%. M.p. = 171-173 °C. TLC: R_f = 0.30 (*n*-hexane : ethyl acetate = 4 : 1). ^1H NMR (400 MHz, $CDCl_3$): δ 6.40 (s, 1H) (N*H*), 2.09 (s, 3H) ($COCH_3$), 2.05 (m, 2H), 1.50 (m, 2H), 1.32 (m, 2H) (CH_2), 0.92 (d, *J* = 7.3 Hz, 3H) (CH_2CH_3). $^{13}C\{^1H\}$ NMR (101 MHz, $CDCl_3$): δ 169.2 (*C*O), 83.8, 83.4 (cage *C*), 34.0, 31.5, 24.1, 22.5 (CH_2 & $COCH_3$), 13.8 (CH_3). $^{11}B\{^1H\}$ NMR (128 MHz, $CDCl_3$): δ -5.7 (1B), -6.0 (1B), -10.9 (4B), -12.0 (4B). IR ν (film) cm^{-1}: 3210, 2962, 2571, 1695, 1533, 1277, 1036, 726, 519. HRMS (DART) Calcd for $C_8H_{24}{}^{10}B_2{}^{11}B_8NO^+$ [M+H$^+$]: 258.2856. Found: 258.2855.

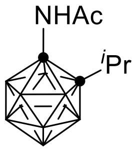

1f

1f: White solid. Yield: 85%. M.p. = 198-200 °C. TLC: R_f = 0.49 (*n*-hexane : ethyl acetate = 3 : 1). ^1H NMR (400 MHz, $CDCl_3$): δ 6.43 (s, 1H) (N*H*), 2.19 (m, 1H) ($CH(CH_3)_2$), 2.08 (s, 3H) ($COCH_3$), 1.21 (d, *J* = 5.5 Hz, 6H) ($CH(CH_3)_2$). $^{13}C\{^1H\}$ NMR (101 MHz, $CDCl_3$): δ 168.2 (*C*O), 89.9, 85.3 (cage *C*), 30.8 ($CH(CH_3)_2$), 24.3, 24.0 (CH_3). $^{11}B\{^1H\}$ NMR (128 MHz, $CDCl_3$): δ -4.8 (1B), -6.6 (1B), -10.9 (2B), -11.8 (4B), -13.3 (2B). IR ν (film) cm^{-1}: 3206, 3022, 2566, 1692, 1534, 1279, 728, 600, 526. HRMS (DART) Calcd for $C_7H_{22}{}^{10}B_2{}^{11}B_8NO^+$ [M+H$^+$]: 244.2699. Found: 244.2699.

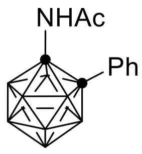

1g

1g: White solid. Yield: 80%. M.p. = 160-162 °C. TLC: R_f = 0.45 (*n*-hexane : ethyl acetate = 3 : 1). ^1H NMR (400 MHz, $CDCl_3$): δ 7.62 (d, *J* = 7.6 Hz, 2H), 7.46 (t, *J* = 7.6 Hz, 1H), 7.38 (t, *J* = 7.6 Hz, 2H) (aromatic C*H*), 6.17 (s, 1H) (N*H*), 1.75 (s, 3H) ($COCH_3$). $^{13}C\{^1H\}$ NMR (101 MHz, $CDCl_3$): δ 168.3 (*C*O), 131.1, 131.0, 130.3, 129.0 (aromatic *C*), 87.2, 85.2 (cage *C*), 23.7 ($COCH_3$). $^{11}B\{^1H\}$ NMR (128 MHz, $CDCl_3$): δ -4.1 (1B), -5.2 (1B) -10.3 (2B), -11.2 (4B), -12.1 (2B). IR ν (film) cm^{-1}: 3200, 3021, 2574, 1711, 1688, 1366, 1231, 1199, 1022, 690, 588, 526. HRMS (DART) Calcd for $C_{10}H_{20}{}^{10}B_2{}^{11}B_8NO^+$ [M+H$^+$]: 278.2543. Found: 278.2538.

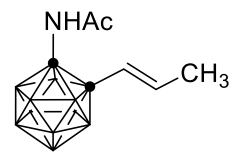

1h

1h: White solid. Yield: 85%. M.p. = 171-173 °C. TLC: R_f = 0.37 (*n*-hexane : ethyl acetate = 3 : 1). ^{1}H NMR (400 MHz, $CDCl_3$): δ 6.37 (s, 1H) (N*H*), 6.20 (m, 1H) (CH=C*H*CH_3), 5.64 (d, *J* = 13.2 Hz, 1H) (C*H*=CHCH_3), 2.03 (s, 3H) (COCH_3), 1.79 (dd, *J* = 6.8, 1.7 Hz, 3H) (CH=CHCH_3). $^{13}C\{^1H\}$ NMR (101 MHz, $CDCl_3$): δ 169.2 (*C*O), 139.5, 122.8 (alkenyl *C*), 83.4, 83.2 (cage *C*), 24.0, 16.2 (*C*H_3). $^{11}B\{^1H\}$ NMR (128 MHz, $CDCl_3$): δ -5.3 (1B), -5.8 (1B), -10.9 (3B), -11.6 (2B), -12.2 (3B). IR ν (film) cm^{-1}: 3207, 3026, 2572, 1693, 1539, 1277, 1038, 954, 725, 599, 521. HRMS (DART) Calcd for $C_7H_{20}{}^{10}B_2{}^{11}B_8NO^+$ [M+H$^+$]: 242.2543. Found: 242.2543.

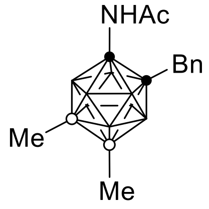

1i

1i: White solid. Yield: 83%. M.p. = 176-178 °C. TLC: R_f = 0.26 (*n*-hexane : ethyl acetate = 4 : 1). ^{1}H NMR (400 MHz, $CDCl_3$): δ 7.33 (m, 3H), 7.14 (m, 2H) (aromatic C*H*), 6.52 (s, 1H) (N*H*), 3.38 (s, 2H) (CH_2), 2.15 (s, 3H) (COCH_3), 0.16 (s, 3H), 0.12 (s, 3H) (B_{cage}-CH_3). $^{13}C\{^1H\}$ NMR (101 MHz, $CDCl_3$): δ 169.6 (*C*O), 135.3, 130.3, 128.7, 128.1 (phenyl *C*), 77.7, 75.5 (cage *C*), 39.8 (*C*H_2), 24.4 (CO*C*H_3), the B_{cage}-*C* was not observed. ^{11}B NMR (128 MHz, $CDCl_3$): δ 4.3 (s, 2B) (B_{cage}-CH_3), -9.7 (d, *J* = 84.1 Hz, 2B), -10.8 (m, 2B), -12.0 (m, 4B) (B_{cage}H). IR ν (film) cm^{-1}: 3213, 3027, 2597, 2544, 1694, 1536, 1279, 987, 698, 607, 535. HRMS (DART) Calcd for $C_{13}H_{26}{}^{10}B_2{}^{11}B_8NO^+$ [M+H$^+$]:320.3012. Found: 320.3011.

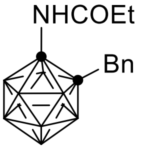

1j

1j: Colorless oil. Yield: 87%. TLC: R_f = 0.38 (*n*-hexane : ethyl acetate = 4 : 1). ^{1}H NMR (400 MHz, $CDCl_3$): δ 7.33 (m, 3H), 7.13 (m, 2H) (aromatic C*H*), 6.43 (s, 1H) (N*H*), 3.39 (s, 2H) (C_{cage}-CH_2), 2.36 (q, *J* = 7.6 Hz, 2H) (COCH_2), 1.23 (t, *J* = 7.6 Hz, 3H) (CH_3). $^{13}C\{^1H\}$ NMR (101 MHz, $CDCl_3$): δ 172.5 (*C*O), 134.9, 130.3, 128.8, 128.2 (aromatic *C*), 83.6, 83.4 (cage *C*), 40.4 (C_{cage}-*C*H_2), 30.4 (CO*C*H_2), 9.4 (*C*H_3). $^{11}B\{^1H\}$ NMR (128 MHz, $CDCl_3$): δ -5.5 (1B), -6.4 (1B), -10.9 (4B), -12.0 (4B). IR ν (film) cm^{-1}: 3207, 2933, 2574, 1689, 1526, 1275, 1076, 695, 522. HRMS (DART) Calcd for $C_{12}H_{24}{}^{10}B_2{}^{11}B_8NO^+$ [M+H$^+$]: 306.2856. Found: 306.2854.

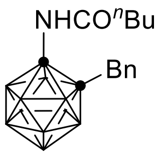

1k

1k: White solid. Yield: 81%. M.p. = 147-149 °C. TLC: R_f = 0.30 (*n*-hexane : ethyl acetate = 8 : 1). ^{1}H NMR (400 MHz, $CDCl_3$): δ 7.33 (m, 3H), 7.13 (m, 2H) (aromatic C*H*), 6.41 (s, 1H) (N*H*), 3.39 (s, 2H) (C_{cage}-CH_2), 2.32 (t, *J* = 7.2 Hz, 2H) (COCH_2), 1.68 (m, 2H), 1.39 (m, 2H) (CH_2), 0.95 (d, *J* = 7.2 Hz, 3H) (CH_3). ^{13}C{^{1}H} NMR (101 MHz, $CDCl_3$): δ 171.6 (*C*O), 134.9, 130.3, 128.8, 128.2 (aromatic *C*), 83.7, 83.0 (cage *C*), 40.4 (C_{cage}-*C*H_2), 37.0 (CO*C*H_2), 27.4, 22.4 (*C*H_2), 13.9 (*C*H_3). ^{11}B{^{1}H} NMR (128 MHz, $CDCl_3$): δ -5.3 (1B), -6.2 (1B), -10.7 (3B), -11.8 (5B). IR ν (film) cm^{-1}: 3287, 2965, 2575, 2551, 1688, 1507, 1257, 1171, 702, 549. HRMS (DART) Calcd for $C_{14}H_{28}{}^{10}B_2{}^{11}B_8NO^+$ [M+H$^+$]: 334.3169. Found: 334.3167.

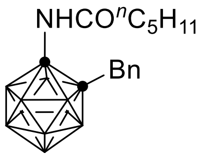

1l

1l: Colorless oil. Yield: 80%. TLC: R_f = 0.25 (*n*-hexane : ethyl acetate = 4 : 1). ^{1}H NMR (400 MHz, $CDCl_3$): δ 7.33 (m, 3H), 7.13 (m, 2H) (aromatic C*H*), 6.54 (s, 1H) (N*H*), 3.39 (s, 2H) (C_{cage}-CH_2), 2.34 (m, 2H) (COCH_2), 1.70 (m, 2H), 1.35 (m, 4H) (CH_2), 0.90 (t, *J* = 7.2 Hz, 3H) (CH_3). ^{13}C{^{1}H} NMR (101 MHz, $CDCl_3$): δ 171.7 (*C*O), 135.0, 130.3, 128.8, 128.2 (aromatic *C*), 83.8, 83.0 (cage *C*), 40.3 (C_{cage}-*C*H_2), 37.2, 31.3, 25.0, 22.5 (*C*H_2), 14.0 (*C*H_3). ^{11}B{^{1}H} NMR (128 MHz, $CDCl_3$): δ -5.3 (1B), -6.2 (1B), -10.7 (4B), -11.8 (4B). IR ν (film) cm^{-1}: 3283, 2929, 2585, 1690, 1516, 1260, 1170, 699, 549. HRMS (DART) Calcd for $C_{15}H_{30}{}^{10}B_2{}^{11}B_8NO^+$ [M+H$^+$]: 348.3325. Found: 348.3319.

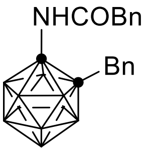

1m

1m: White solid. Yield: 80%. M.p. = 125-127 °C. TLC: R_f = 0.48 (*n*-hexane : ethyl acetate = 4 : 1). ^{1}H NMR (400 MHz, $CDCl_3$): δ 7.43 (t, *J* = 6.8 Hz, 2H), 7.37 (t, *J* = 6.8 Hz, 1H), 7.31 (d, *J* = 6.8 Hz, 2H), 7.28 (m, 3H), 6.86 (m, 2H) (aromatic C*H*), 6.48 (s, 1H) (N*H*), 3.71 (s, 2H), 3.14 (s, 2H) (CH_2). ^{13}C{^{1}H} NMR (101 MHz, $CDCl_3$): δ 169.3 (*C*O), 134.7, 133.3, 130.1, 129.7, 129.6, 129.2, 128.5, 128.3, 1128.0 (aromatic *C*), 83.4, 83.3 (cage *C*), 44.3, 39.8 (*C*H_2). ^{11}B{^{1}H} NMR (128 MHz, $CDCl_3$): δ -5.2 (1B), -6.2 (1B), -11.7 (8B). IR ν (film) cm^{-1}: 3293, 2988, 2575, 1689, 1510, 1263, 699, 547. HRMS (DART) Calcd for $C_{17}H_{26}{}^{10}B_2{}^{11}B_8NO^+$ [M+H$^+$]: 368.3012. Found: 368.3009.

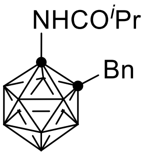

1n

1n: White solid. Yield: 78%. M.p. = 175-178 ºC. TLC: R_f = 0.54 (*n*-hexane : ethyl acetate = 4 : 1). ^{1}H NMR (400 MHz, $CDCl_3$): δ 7.33 (m, 3H), 7.13 (m, 2H) (aromatic C*H*), 6.45 (s, 1H) (N*H*), 3.37 (s, 2H) (C*H*$_2$), 2.44 (m, 1H) (C*H*), 1.25 (d, *J* = 6.9 Hz, 6H) (C*H*$_3$). ^{13}C{^{1}H} NMR (101 MHz, $CDCl_3$): δ 175.1 (*C*O), 134.9, 130.4, 128.8, 128.2 (aromatic *C*), 83.7, 82.1 (cage *C*), 40.3 (*C*H$_2$), 36.5 (*C*H), 19.5 (*C*H$_3$). ^{11}B{^{1}H} NMR (128 MHz, $CDCl_3$): δ -5.3 (1B), -6.3 (1B), -10.7 (2B), -11.0 (2B), -11.8 (4B). IR ν (film) cm^{-1}: 3270, 2972, 2569, 1694, 1520, 1257, 695, 643. HRMS (DART) Calcd for $C_{13}H_{26}{}^{10}B_2{}^{11}B_8NO^+$ [M+H$^+$]: 320.3012. Found: 320.3011.

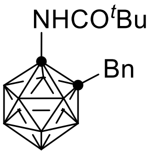

1o

1o: White solid. Yield: 75%. M.p. = 172-174 ºC. TLC: R_f = 0.39 (*n*-hexane : ethyl acetate = 8 : 1). ^{1}H NMR (400 MHz, $CDCl_3$): δ 7.33 (m, 3H), 7.13 (m, 2H) (aromatic C*H*), 6.58 (s, 1H) (N*H*), 3.35 (s, 2H) (C*H*$_2$), 1.29 (s, 9H) (C*H*$_3$). ^{13}C{^{1}H} NMR (101 MHz, $CDCl_3$): δ 176.4 (*C*O), 134.8, 130.3, 128.7, 128.2 (aromatic *C*), 84.0, 83.0 (cage *C*), 40.1 (*C*H$_2$), 40.0 (*C*(C*H*$_3$)$_3$), 27.4 (*C*H$_3$). ^{11}B{^{1}H} NMR (128 MHz, $CDCl_3$): δ -5.4 (1B), -6.4 (1B), -10.8 (4B), -11.8 (4B). IR ν (film) cm^{-1}: 3337, 2977, 2560, 1694, 1498, 1251, 1126, 698, 612. HRMS (DART) Calcd for $C_{14}H_{28}{}^{10}B_2{}^{11}B_8NO^+$ [M+H$^+$]: 334.3169. Found: 334.3167.

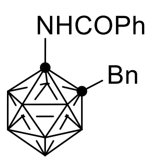

1p

1p: White solid. Yield: 81%. M.p. = 161-163 ºC. TLC: R_f = 0.30 (*n*-hexane : ethyl acetate = 8 : 1). ^{1}H NMR (400 MHz, $CDCl_3$): δ 7.80 (m, 2H), 7.64 (m, 1H), 7.54 (t, *J* = 7.6 Hz, 2H), 7.33 (m, 3H), 7.12 (m, 3H) (aromatic C*H* & N*H*), 3.49 (s, 2H) (C*H*$_2$). ^{13}C{^{1}H} NMR (101 MHz, $CDCl_3$): δ 165.4 (*C*O), 134.8, 133.3, 132.4, 130.2, 129.2, 128.7, 127.2 (aromatic *C*), 83.7, 83.3 (cage *C*), 40.5 (*C*H$_2$). ^{11}B{^{1}H} NMR (128 MHz, $CDCl_3$): δ -5.2 (1B), -6.0 (1B), -10.6 (4B), -11.7 (4B). IR ν (film) cm^{-1}: 2958, 2567, 1669, 1531, 1286, 699, 686, 666. HRMS (DART) Calcd for $C_{16}H_{24}{}^{10}B_2{}^{11}B_8NO^+$ [M+H$^+$]: 354.2856. Found: 354.2854.

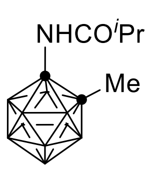

1q

1q: White solid. Yield: 82%. M.p. = 172-174 ºC. TLC: R_f = 0.43 (*n*-hexane : ethyl acetate = 4 : 1). ^{1}H NMR (400 MHz, $CDCl_3$): δ 6.42 (s, 1H) (N*H*), 2.38 (m, 1H) (C*H*(CH$_3$)$_2$),1.95 (s, 3H) (C*H*$_3$), 1.19 (d, *J* = 6,9 Hz, 6H) (CH(C*H*$_3$)$_2$). ^{13}C{^{1}H} NMR (101 MHz, $CDCl_3$): δ 175.4 (*C*O), 82.1, 78.4 (cage *C*), 28.3 (*C*H(CH$_3$)$_2$), 22.3, 19.4 (*C*H$_3$). ^{11}B{^{1}H} NMR (128 MHz, $CDCl_3$): δ -6.1 (1B), -6.4 (1B), -10.5 (4B), -11.3 (2B), -12.1 (2B).

IR ν (film) cm^{-1}: 3263, 2934, 2566, 1695, 1523, 1257, 726, 665. HRMS (DART) Calcd for $C_7H_{22}{}^{10}B_2{}^{11}B_8NO^+$ [M+H$^+$]: 244.2699. Found: 244.2700.

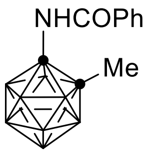

1r

1r: White solid. Yield: 80%. M.p. = 182-184 °C. TLC: R_f = 0.43 (*n*-hexane : ethyl acetate = 4 : 1). ^{1}H NMR (400 MHz, $CDCl_3$): *δ* 7.73 (d, *J* = 7.2 Hz, 2H), 7.61 (t, *J* = 7.2 Hz, 1H), 7.50 (t, *J* = 7.6 Hz, 2H) (aromatic C*H*), 7.04 (s, 1H) (N*H*), 2.04 (s, 3H) (C*H*$_3$). ^{13}C{^{1}H} NMR (101 MHz, $CDCl_3$): *δ* 165.7 (*C*O), 133.3, 132.7, 129.2, 127.3 (aromatic *C*), 82.3, 78.9 (cage *C*), 22.5 (*C*H$_3$). ^{11}B{^{1}H} NMR (128 MHz, $CDCl_3$): *δ* -5.8 (1B), -6.3 (1B) -10.3 (4B), -11.2 (2B), -11.8 (2B). IR ν (film) cm^{-1}: 3262, 2574, 1680, 1518, 1272, 719, 658, 640. HRMS (DART) Calcd for $C_{10}H_{20}{}^{10}B_2{}^{11}B_8NO^+$ [M+H$^+$]: 278.2543. Found: 278.2540.

General procedure for the synthesis of 3.

Method A: An oven-dried Schlenk flask equipped with a stir bar was charged with $[Ir(COD)Cl]_2$ (1.7 mg, 0. 0025 mmol) and (*S*)-**L8** (2.6 mg, 0.005 mmol), followed by dry toluene (1 mL). The mixture was stirred at room temperature for 1 h, to which was successively added **1** (0.10 mmol), **2** (0.12 mmol), benzenesulfonic acid (24 mg, 0.15 mmol), $AgNTf_2$ (4.9 mg, 0.0125 mmol) and dry toluene (1 mL). The flask was closed under an atmosphere of nitrogen, then stirred at 50 °C for 40 h. After hydrolysis with water (5 mL) and extraction with diethyl ether (10 mL x 3), the ether solutions were combined, dried over anhydrous Na_2SO_4 and concentrated to dryness in vacuo. The residue was subjected to flash column chromatography on silica gel (230-400 mesh) using *n*-hexane and ethyl acetate (4/1 in v/v) as eluent to give product (*S*)-**3**.

Method B: An oven-dried Schlenk flask equipped with a stir bar was charged with $[Ir(COD)Cl]_2$ (1.7 mg, 0. 0025 mmol) and (*S*)-**L8** (2.6 mg, 0.005 mmol), followed by dry toluene (1 mL). The mixture was stirred at room temperature for 1 h, to which was successively added **1** (0.1 mmol), **2** (0.12 mmol), benzenesulfonic acid (24 mg, 0.15 mmol), $AgNTf_2$ (4.9 mg, 0.0125 mmol) and another dry toluene (1 mL). The flask was closed under an atmosphere of nitrogen, then stirred at 80 °C for 40 h. After hydrolysis with water (5 mL) and extraction with diethyl ether (10 mL x 3), the ether solutions were combined, dried over anhydrous Na_2SO_4 and concentrated to dryness in vacuo. The residue was subjected to flash column chromatography on silica gel (230-400 mesh) using *n*-hexane and ethyl acetate (4/1 in v/v) as eluent to give product (*S*)-**3**.

Method C：An oven-dried Schlenk flask equipped with a stir bar was charged with $[Ir(COD)Cl]_2$ (1.7 mg, 0. 0025 mmol) and (*S*)-**L8** (2.6 mg, 0.005 mmol), followed by dry toluene (1 mL). The mixture was stirred at room temperature for 1 h, to which was successively added **1** (0.1 mmol), **2** (0.12 mmol), copper acetate (20 mg, 0.11 mmol), benzenesulfonic acid (24 mg, 0.15 mmol), $AgNTf_2$ (4.9 mg, 0.0125 mmol) and another dry toluene (1 mL). The flask was closed under an atmosphere of nitrogen, then stirred at 50 °C for 40 h. After hydrolysis with water (5 mL) and extraction with diethyl ether (10 mL x 3), the ether solutions were combined, dried over anhydrous Na_2SO_4 and concentrated to dryness in vacuo. The residue was subjected to flash column chromatography on silica gel (230-400 mesh) using *n*-hexane and ethyl acetate (4/1 in v/v) as eluent to give product (*S*)-**3**.

Method D：An oven-dried Schlenk flask equipped with a stir bar was charged with $[Ir(COD)Cl]_2$ (1.7 mg, 0. 0025 mmol) and (*S*)-**L8** (2.6 mg, 0.005 mmol), followed by dry toluene (1 mL). The mixture was stirred at room temperature for 1 h, to which was successively added **1** (0.1 mmol), **2** (0.12 mmol), copper acetate (20 mg, 0.11 mmol), benzenesulfonic acid (24 mg, 0.15 mmol), $AgNTf_2$ (4.9 mg, 0.0125 mmol) and another dry toluene (1 mL). The flask was closed under an atmosphere of nitrogen, then stirred at 80 °C for 40 h. After hydrolysis with water (5 mL) and extraction with diethyl ether (10 mL x 3), the ether solutions were combined, dried over anhydrous Na_2SO_4 and concentrated to dryness in vacuo. The residue was subjected to flash column chromatography on silica gel (230-400 mesh) using *n*-hexane and ethyl acetate (4/1 in v/v) as eluent to give product (*S*)-**3**.

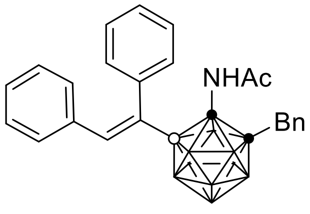

(*S*)-**3ba**

(*S*)-**3ba**: White solid. M.p. = 208-221 °C. TLC: R_f = 0.33 (*n*-hexane : ethyl acetate = 4 : 1). Method **A**: Yield: 99%. 99% ee, $[\alpha]_D^{30}$= -21.5 (*c* = 1.00, CH_2Cl_2). HPLC condition: Chiralpak IA (0.46 x 25 cm, 5 μm), acetonitrile/water = 99:1, 0.7 mL/min, 214 nm UV detector, t_R = 26.27 min (major) and t_R = 31.42 min (minor). ^{1}H NMR (400 MHz, $CDCl_3$): δ 7.36 (m, 2H), 7.31 (m, 3H), 7.28 (m, 1H), 7.14 (d, *J* = 7.2 Hz, 2H), 7.09 (m, 5H) (aromatic C*H*), 7.05 (s, 1H) (alkenyl C*H*), 6.91 (m, 2H) (aromatic C*H*), 6.08 (s, 1H) (N*H*), 3.32 (s, 2H) (CH_2), 1.96 (s, 3H) (COCH_3). ^{13}C{^{1}H} NMR (101 MHz, $CDCl_3$): δ 168.2 (*C*O), 142.7, 142.1, 137.1, 134.8, 130.2, 129.7, 129.1, 128.7, 128.4, 128.1, 128.0, 127.4, 126.8 (aromatic *C* & alkenyl *C*), 84.1, 82.7 (cage *C*), 40.3 (*C*H_2), 24.2 (CO*C*H_3), the B_{cage}-*C* was not observed. ^{11}B NMR (128 MHz, $CDCl_3$): δ -1.1 (s, 1B) (*B*$_{cage}$-C), -4.5 (d, *J* = 134.4 Hz, 1B), -5.6 (d, *J* = 108.8 Hz, 1B), -11.4 (d, *J* = 67.8 Hz, 4B), -12.5 (d, *J* = 137.0

Hz, 1B), -13.8 (d, J = 172.8 Hz, 2B) (B_{cage}H). IR ν (film) cm^{-1}: 3190, 3018, 2565, 1682, 1541, 1284, 751, 698, 595, 535. HRMS (DART) Calcd for $C_{25}H_{32}{}^{10}B_2{}^{11}B_8NO^+$ [M+H$^+$]: 470.3482. Found: 470.3480.

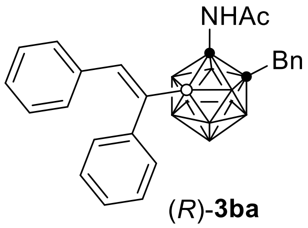

(R)-**3ba**

(R)-**3ba**: (R)-**L8** was used as ligand. White solid. Method **A**: Yield: 99%. -99% ee, $[\alpha]_D^{30}$ = 22.7 (c = 1.00, CH_2Cl_2). HPLC condition: Chiralpak IA (0.46 x 25 cm, 5 μm), acetonitrile/water = 99:1, 0.7 mL/min, 214 nm UV detector, t_R = 27.67 min (minor) and t_R = 32.19 min (major).

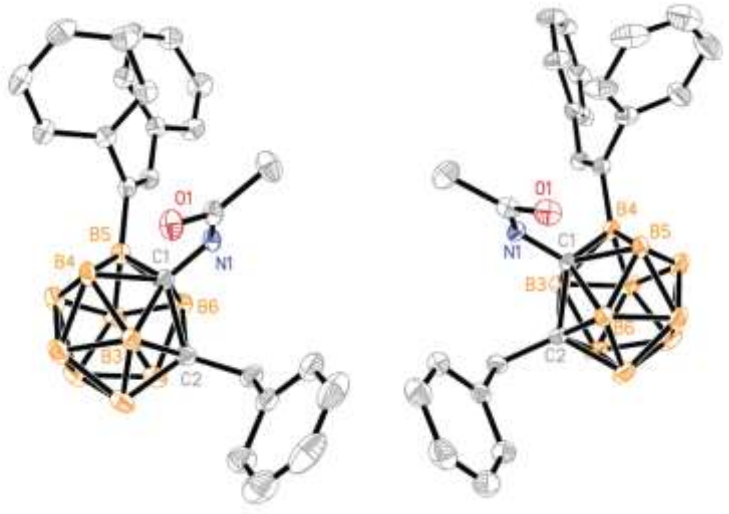

Supplementary Figure 1. Molecular structures of (S)-**3ba** (left) and (R)-**3ba** (right)

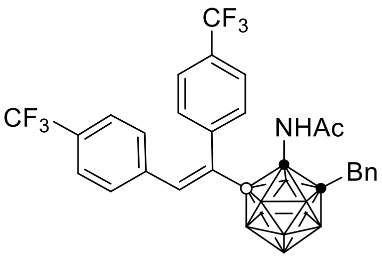

(S)-**3bb**

(S)-**3bb**: White solid. M.p. = 177-179 °C. TLC: R_f = 0.40 (n-hexane : ethyl acetate = 4 : 1). Method **A**: Yield: 99%. 97% ee, $[\alpha]_D^{28}$ = 19.7 (c = 1.00, CH_2Cl_2). HPLC condition: Chiralpak IA (0.46 x 25 cm, 5 μm), acetonitrile/water = 95:5, 0.7 mL/min, 214 nm UV detector, t_R = 9.81 min (major) and t_R = 12.09 min (minor). ^{1}H NMR (400 MHz, $CDCl_3$): δ 7.61 (d, J = 8.0 Hz, 2H), 7.37 (d, J = 8.4 Hz, 2H), 7.34 (d, J = 1.2 Hz, 1H), 7.33 (d, J = 2.0 Hz, 2H), 7.23 (d, J = 8.4 Hz, 2H), 7.12 (m, 2H), 7.10 (d, J = 1.6 Hz, 1H), 6.97 (d, J = 8.4 Hz, 2H) (aromatic CH & alkenyl CH), 6.13 (s, 1H) (NH), 3.38 (s, 2H) (CH_2), 2.00 (s, 3H) (COCH_3). ^{13}C{^{1}H} NMR (101 MHz, $CDCl_3$): δ 167.8 (CO), 146.2, 141.0, 140.2, 130.3, 129.8, 129.4 (q, $^2J_{CF}$ = 32.6 Hz), 129.3 (q, $^2J_{CF}$ = 32.7 Hz), 128.8, 128.3, 126.0 (q, $^3J_{CF}$ = 3.6 Hz), 125.2 (q, $^3J_{CF}$ = 3.6 Hz) (aromatic C & alkenyl C), 124.1 (q, $^1J_{CF}$ = 273.4 Hz), 124.2 (q, $^1J_{CF}$ = 273.4 Hz) (CF$_3$),

84.8, 82.9 (cage *C*), 40.6 (*C*H_2), 24.1 (CO*C*H_3), the B_{cage}-*C* was not observed. ^{11}B NMR (128 MHz, $CDCl_3$): *δ* -1.8 (s, 1B) (*B*$_{cage}$-C), -4.3 (d, *J* = 120.3 Hz, 1B), -5.5 (d, *J* = 103.7 Hz, 1B), -11.4 (d, *J* = 83.2 Hz, 3B), -12.2 (d, *J* = 108.8 Hz, 2B), -13.5 (d, *J* = 140.8 Hz, 2B) (*B*$_{cage}$H). ^{19}F NMR (376 MHz, $CDCl_3$): *δ* -62.4, -62.7. IR ν (film) cm^{-1}: 2568, 1696, 1322, 1163, 1125, 1064, 699, 540. HRMS (DART) Calcd for $C_{27}H_{30}{}^{10}B_2{}^{11}B_8F_6NO^+$ [$M+H^+$]: 606.3229. Found: 606.3222.

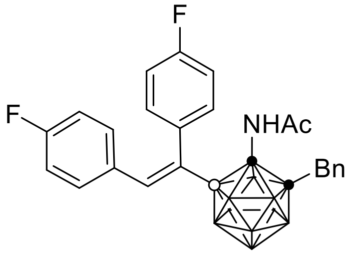

(*S*)-**3bc**

(*S*)-**3bc**: White solid. M.p. = 194-199 °C. TLC: R_f = 0.26 (*n*-hexane : ethyl acetate = 4 : 1). Method **A**: Yield: 99%. 95% ee, $[\alpha]_D^{28}$ = 5.0 (*c* = 0.50, CH_2Cl_2). HPLC condition: Chiralpak IA (0.46 x 25 cm, 5 μm), acetonitrile/water = 95:5, 0.7 mL/min, 214 nm UV detector, t_R = 11.40 min (major) and t_R = 14.27 min (minor). ^{1}H NMR (400 MHz, $CDCl_3$): *δ* 7.32 (m, 3H), 7.10 (m, 2H), 7.07 (m, 2H), 7.06 (m, 2H) (aromatic C*H*), 7.00 (s, 1H) (alkenyl C*H*), 6.87 (m, 2H), 6.80 (m, 2H) (aromatic C*H*), 6.11 (s, 1H) (N*H*), 3.35 (s, 2H) (C*H*$_2$), 2.00 (s, 3H) (COC*H*$_3$). ^{13}C{^{1}H} NMR (101 MHz, $CDCl_3$): *δ* 167.8 (*C*O), 161.9 (d, $^1J_{CF}$ = 199.4 Hz), 161.8 (d, $^1J_{CF}$ = 198.0 Hz), 141.2, 138.3 (d, $^4J_{CF}$ = 2.7 Hz), 134.9, 133.2 (d, $^4J_{CF}$ = 2.7 Hz), 131.4 (d, $^3J_{CF}$ = 6.4 Hz), 130.3, 130.2 (d, $^3J_{CF}$ = 5.9 Hz), 128.8, 128.3, 116.2 (d, $^2J_{CF}$ = 17.2 Hz), 115.1 (d, $^4J_{CF}$ = 17.2 Hz) (aromatic *C* & alkenyl *C*), 84.3, 82.9 (cage *C*), 40.5 (*C*H_2), 24.2 (CO*C*H_3), the B_{cage}-*C* was not observed. ^{11}B NMR (128 MHz, $CDCl_3$): *δ* -1.0 (s, 1B) (*B*$_{cage}$-C), -4.5 (d, *J* = 106.2 Hz, 1B), -5.4 (d, *J* = 128.0 Hz, 1B), -11.5 (d, *J* = 98.6 Hz, 3B), -12.4 (d, *J* = 137.0 Hz, 2B), -13.7 (d, *J* = 174.1 Hz, 2B) (*B*$_{cage}$H). ^{19}F NMR (376 MHz, $CDCl_3$): *δ* -113.6 (m, 1F), -115.3 (m, 1F). IR ν (film) cm^{-1}: 2562, 1700, 1598, 1504, 1223, 1114, 698, 521. HRMS (DART) Calcd for $C_{25}H_{30}{}^{10}B_2{}^{11}B_8F_2NO^+$ [$M+H^+$]: 506.3293. Found: 506.3293.

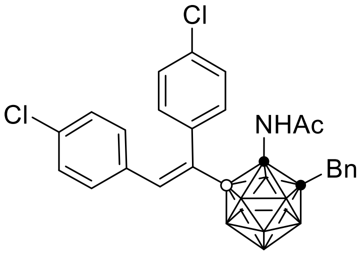

(*S*)-**3bd**

(*S*)-**3bd**: White solid. M.p. = 157-159 °C. TLC: R_f = 0.27 (*n*-hexane : ethyl acetate = 4 : 1). Method **A**: Yield: 99%. 94% ee, $[\alpha]_D^{26}$ = 8.7 (*c* = 1.00, CH_2Cl_2). HPLC condition: Chiralpak IA (0.46 x 25 cm, 5 μm), acetonitrile/water = 9:1, 0.7 mL/min, 214 nm UV detector, t_R = 11.34 min (major) and t_R = 15.38 min (minor).^{1}H NMR (400 MHz, $CDCl_3$): *δ* 7.33 (m, 5H), 7.09 (m, 4H), 7.04 (d, *J* = 8.0 Hz, 2H), 6.99 (s, 1H), 6.83 (d, *J* = 8.4 Hz, 2H) (aromatic C*H* & alkenyl C*H*), 6.13 (s, 1H) (N*H*), 3.35 (s, 2H) (C*H*$_2$), 1.99 (s, 3H)

(COCH_3). $^{13}C\{^1H\}$ NMR (101 MHz, $CDCl_3$): δ 167.9 (*C*O), 135.3, 134.7, 133.3, 132.9, 130.9, 130.3, 129.9, 129.3, 128.8, 128.4, 128.3 (aromatic *C* & alkenyl *C*), 84.5, 82.8 (cage *C*), 40.5 (*C*H_2), 24.2 (CO*C*H_3), the B_{cage}-*C* was not observed. ^{11}B NMR (128 MHz, $CDCl_3$): δ -1.3 (s, 1B) (B_{cage}-C), -4.2 (d, J = 108.8 Hz, 1B), -5.1 (d, J = 119.0 Hz, 1B), -11.3 (d, J = 71.7 Hz, 3B), -11.9 (d, J = 125.4 Hz, 2B), -13.4 (d, J = 139.5 Hz, 2B) (B_{cage}H). IR ν (film) cm^{-1}: 2573, 1687, 1486, 1272, 1089, 1012, 750, 699, 521. HRMS (DART) Calcd for $C_{25}H_{30}{}^{10}B_2{}^{11}B_8Cl_2NO^+$ [M+H$^+$]: 538.2702. Found: 538.2696

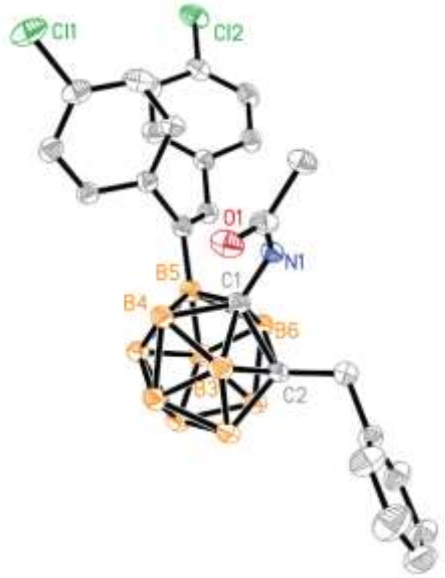

Supplementary Figure 2. Molecular structure of (*S*)-**3bd**

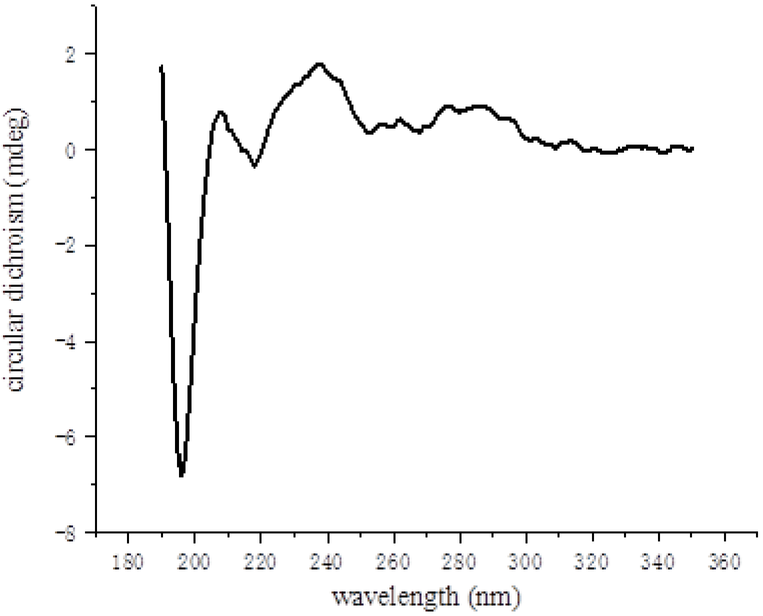

Supplementary Figure 3. CD Spectra of (*S*)-**3bd** in MeCN（c = 0.1 mg/mL）

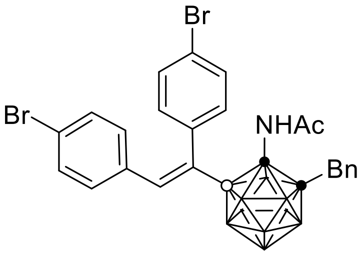

(S)-**3be**

(S)-**3be**: White solid. M.p. = 184-186 °C. TLC: R_f = 0.29 (*n*-hexane : ethyl acetate = 4 : 1). Method **A**: Yield: 98%. 89% ee, $[\alpha]_D^{28}$ = 16.4 (c = 1.00, CH_2Cl_2). HPLC condition: Chiralpak IA (0.46 x 25 cm, 5 μm), acetonitrile/water = 95:5, 0.7 mL/min, 214 nm UV detector, t_R = 12.03 min (major) and t_R = 17.23 min (minor). ^{1}H NMR (400 MHz, $CDCl_3$): δ 7.48 (d, J = 7.6 Hz, 2H), 7.33 (m, 3H), 7.24 (m, 2H), 7.10 (m, 2H), 6.99 (m, 3H), 6.77 (d, J = 8.4 Hz, 2H) (aromatic C*H* & alkenyl C*H*), 6.06 (s, 1H) (N*H*), 3.35 (s, 2H) (CH_2), 1.99 (s, 3H) ($COCH_3$). ^{13}C{^{1}H} NMR (101 MHz, $CDCl_3$): δ 167.7 (*C*O), 141.3, 141.2, 135.7, 134.7, 132.3, 131.4, 130.3, 130.2, 128.8, 128.3, 121.7, 121.0 (aromatic *C* & alkenyl *C*), 84.3, 82.8 (cage *C*), 40.5 (CH_2), 24.3 ($COCH_3$), the B_{cage}-*C* was not observed. ^{11}B NMR (128 MHz, $CDCl_3$): δ -1.5 (s, 1B) (B_{cage}-C), -4.3 (d, J = 111.4 Hz, 1B), -5.1 (d, J = 105.0 Hz, 1B), -11.5 (d, J = 88.3 Hz, 3B), -12.3 (d, J = 110.1 Hz, 2B), -12.7 (d, J = 157.4 Hz, 2B) (B_{cage}H). IR ν (film) cm^{-1}: 2568, 1691, 1483, 1264, 1070, 1008, 741, 699, 540. HRMS (DART) Calcd for $C_{25}H_{30}{}^{10}B_2{}^{11}B_8Br_2NO^+$ [M+H$^+$]: 628.1671. Found: 628.1663.

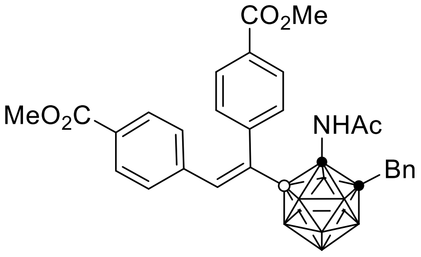

(S)-**3bf**

(S)-**3bf**: White solid. M.p. = 205-208 °C. TLC: R_f = 0.48 (*n*-hexane : ethyl acetate = 2 : 1). Method **A**: Yield: 99%. 92% ee, $[\alpha]_D^{23}$ = 17.3 (c = 1.00, CH_2Cl_2). HPLC condition: Chiralpak IA (0.46 x 25 cm, 5 μm), acetonitrile/water = 9:1, 0.7 mL/min, 214 nm UV detector, t_R = 12.90 min (major) and t_R = 15.63 min (minor). ^{1}H NMR (400 MHz, $CDCl_3$): δ 8.02 (d, J = 8.4 Hz, 2H), 7.76 (d, J = 8.4 Hz, 2H), 7.34 (m, 3H), 7.19 (d, J = 8.4 Hz, 2H), 7.12 (m, 3H), 6.94 (d, J = 8.4 Hz, 2H) (aromatic C*H* & alkenyl C*H*), 6.14 (s, 1H) (N*H*), 3.94 (s, 3H), 3.86 (s, 3H) (CO_2CH_3), 3.35 (s, 2H) (C_{cage}-CH_2), 1.99 (s, 3H) ($COCH_3$). ^{13}C{^{1}H} NMR (101 MHz, $CDCl_3$): δ 168.0, 167.0, 166.9 (NH*C*O & *C*O_2Me), 147.8, 141.4, 141.1, 134.7, 130.3, 130.0, 129.4, 128.8, 128.7, 128.6, 128.2 (aromatic *C* & alkenyl *C*), 84.9, 83.1 (cage *C*), 52.4, 52.3 (CO_2CH_3), 40.4 (CH_2), 24.2 ($COCH_3$), the B_{cage}-*C* was not observed. ^{11}B NMR (128 MHz, $CDCl_3$): δ -1.8 (s, 1B) (B_{cage}-C), -4.3 (d, J = 135.7 Hz, 1B), -5.3 (d, J = 90.9 Hz, 1B), -11.5 (m, 5B), -13.8 (m, 2B) (B_{cage}H). IR ν (film) cm^{-1}: 2918, 2570, 1695, 1434, 1272, 1102, 725, 700. HRMS (DART) Calcd for $C_{29}H_{36}{}^{10}B_2{}^{11}B_8NO_5^+$ [M+H$^+$]: 586.3591. Found: 586.3592.

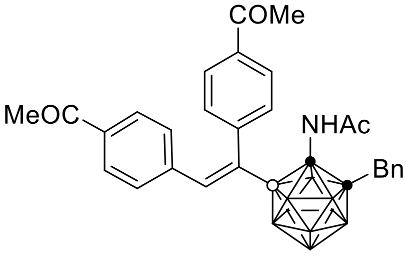

(*S*)-**3bg**

(*S*)-**3bg**: White solid. M.p. = 230-232 °C. TLC: R_f = 0.23 (*n*-hexane : ethyl acetate = 2 : 1). Method **A**: Yield: 68%. 38% ee, $[\alpha]_D^{29}$ = 15.6 (*c* = 0.30, CH_2Cl_2). HPLC condition: Chiralpak IE3 (0.46 x 25 cm, 3 μm), acetonitrile/water = 8:2, 0.7 mL/min, 214 nm UV detector, t_R = 26.99 min (minor) and t_R = 31.38 min (major). ^{1}H NMR (400 MHz, $CDCl_3$): *δ* 7.92 (d, *J* = 8.0 Hz, 2H), 7.67 (d, *J* = 8.0 Hz, 2H), 7.33 (m, 3H), 7.21 (d, *J* = 8.0 Hz, 2H), 7,12 (m, 3H), 6.95 (d, *J* = 8.4 Hz, 2H), (aromatic C*H* & alkenyl C*H*), 6.56 (s, 1H) (N*H*), 3.39 (s, 2H) (CH_2), 2.63 (s, 3H), 2.51 (s, 3H) (phenyl-COCH_3), 2.03 (s, 3H) (NHCOCH_3). ^{13}C{^{1}H} NMR (101 MHz, $CDCl_3$): *δ* 198.1, 198.0 (phenyl-*C*O), 168.1 (NH*C*O), 148.2, 141.7, 140.9, 135.6, 134.8, 130.3, 129.8, 129.1, 128.8, 128.7, 128.3 (aromatic *C* & alkenyl *C*), 85.1, 83.3 (cage *C*), 40.5 (*C*H_2), 26.8, 24.2 (CO*C*H_3), the B_{cage}-*C* was not observed. ^{11}B NMR (128 MHz, $CDCl_3$): *δ* -1.7 (s, 1B) (B_{cage}-C), -4.4 (d, *J* = 113.2 Hz, 1B), -5.3 (d, *J* = 111.1 Hz, 1B), -11.7 (m, 5B), -13.7 (m, 2B) (B_{cage}H). IR ν (film) cm^{-1}: 3220, 2995, 2575, 1660, 1599, 1263, 1015, 697, 602. HRMS (DART) Calcd for $C_{29}H_{36}{}^{10}B_2{}^{11}B_8NO_3{}^+$ [M+H$^+$]: 554.3693. Found: 554.3692.

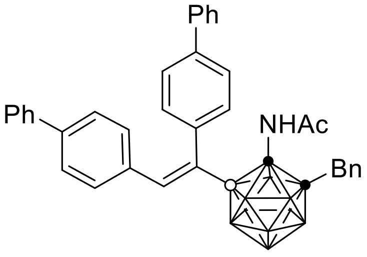

(*S*)-**3bh**

(*S*)-**3bh**: White solid. M.p. = 184-186 °C. Method **D**: Yield: 78%. 77% ee, $[\alpha]_D^{29}$ = -9.4 (*c* = 0.80, CH_2Cl_2). HPLC condition: Chiralpak IA (0.46 x 25 cm, 5 μm), acetonitrile/water = 95:5, 0.7 mL/min, 214 nm UV detector, t_R = 19.49 min (major) and t_R = 28.09 min (minor). ^{1}H NMR (400 MHz, $CDCl_3$): δ 7.68 (m, 4H), 7.53 (d, *J* = 7.7 Hz, 2H), 7.49 (t, *J* = 7.5 Hz, 2H), 7.40 (m, 5H), 7.33 (m, 4H), 7.29 (d, *J* = 8.0 Hz, 2H), 7.13 (m, 3H), 7.06 (d, *J* = 8.1 Hz, 2H) (aromatic C*H* & alkenyl C*H*), 6.40 (s, 1H) (N*H*), 3.37 (s, 2H) (CH_2), 1.99 (s, 3H) (COCH_3). ^{13}C{^{1}H} NMR (101 MHz, $CDCl_3$): *δ* 168.2 (*C*O), 141.9, 141.8, 140.4, 140.3, 140.0, 139.4, 136.2, 134.9, 130.3, 130.2, 129.0, 128.8, 128.7, 128.2, 127.7, 127.6, 127.5, 126.9, 126.7 (aromatic *C* & alkenyl *C*), 84.1, 82.9 (cage *C*), 40.4 (*C*H_2), 24.2 (CO*C*H_3), the B_{cage}-*C* was not observed. ^{11}B NMR (128 MHz, $CDCl_3$): *δ* -1.2 (s, 1B) (B_{cage}-C), -4.2 (d, *J* = 102.3 1B), -5.3 (d, *J* = 100.1, 1B), -11.4 (d, *J* = 74.5 Hz, 3B), -12.1 (d, *J* = 133.6 Hz, 2B), -13.7 (d, *J* = 165.2 Hz, 2B) (B_{cage}H). IR ν (film) cm^{-1}: 3640, 2696, 2577, 1696, 1557, 1287, 762, 700, 545. HRMS (DART) Calcd for $C_{37}H_{40}{}^{10}B_2{}^{11}B_8NO^+$ [M+H$^+$]: 622.4108. Found: 622.4101.

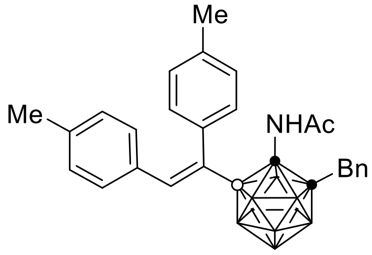

(S)-**3bi**

(S)-**3bi**: White solid. M.p. = 209-210 °C. TLC: R_f = 0.37 (*n*-hexane : ethyl acetate = 4 : 1). Method **B**: Yield: 92%. 93% ee, $[\alpha]_D^{27}$ = -24.4 (*c* = 1.17, CH_2Cl_2). Method **C**: Yield: 99%. 96% ee, $[\alpha]_D^{27}$ = -29.1 (*c* = 1.00, CH_2Cl_2). HPLC condition: Chiralpak IA (0.46 x 25 cm, 5 μm), acetonitrile/water = 95:5, 0.7 mL/min, 214 nm UV detector, t_R = 12.88 min (major) and t_R = 15.50 min (minor). ^1H NMR (400 MHz, $CDCl_3$): δ 7.31 (m, 3H), 7.17 (d, *J* = 7.6 Hz, 2H), 7.09 (m, 2H), 7.03 (d, *J* = 7.6 Hz, 2H) (aromatic C*H*), 6.98 (s, 1H) (alkenyl C*H*), 6.91 (d, *J* = 8.0 Hz, 2H), 6.81 (d, *J* = 8.0 Hz, 2H) (aromatic C*H*), 6.11 (s, 1H) (N*H*), 3.31 (s, 2H) (CH_2), 2.38 (s, 3H), 2.24 (s, 3H), 1.96 (s, 3H) (CH_3). $^{13}C\{^1H\}$ NMR (101 MHz, $CDCl_3$): δ 168.2 (*C*O), 141.9, 139.9, 137.3, 136.3, 134.5, 130.3, 129.7, 129.3, 128.8, 128.7, 128.4, 128.1 (aromatic *C* & alkenyl *C*), 83.7, 82.9 (cage *C*), 40.4 (CH_2), 24.2, 21.4, 21.3 (CH_3), the B_{cage}-*C* was not observed. ^{11}B NMR (128 MHz, $CDCl_3$): δ -0.7 (s, 1B) (B_{cage}-C), -4.3 (d, *J* = 108.8 Hz, 1B), -5.3 (d, *J* = 107.5 Hz, 1B), -11.4 (d, *J* = 85.8 Hz, 3B), -12.3 (d, *J* = 129.3 Hz, 2B), -13.9 (d, *J* = 275.2 Hz, 2B) (B_{cage}H). IR ν (film) cm^{-1}: 3258, 3003, 2572, 1679, 1599, 1313, 1029, 699, 508, 472. HRMS (DART) Calcd for $C_{27}H_{36}{}^{10}B_2{}^{11}B_8NO^+$ $[M+H^+]$: 498.3795. Found: 498.3793.

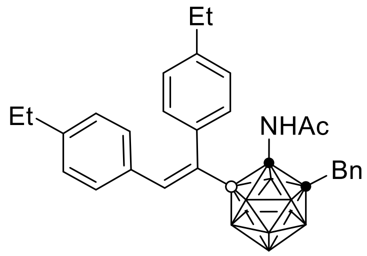

(S)-**3bj**

(S)-**3bj**: White solid. M.p. = 206-209 °C. TLC: R_f = 0.45 (*n*-hexane : ethyl acetate = 4 : 1). Method **B**: Yield: 75%. 86% ee, $[\alpha]_D^{28}$ = -33.3 (*c* = 1.00, CH_2Cl_2). Method **C**: Yield: 98%. 95% ee, $[\alpha]_D^{28}$ = -39.8 (*c* = 1.08, CH_2Cl_2). HPLC condition: Chiralpak IA (0.46 x 25 cm, 5 μm), acetonitrile/water = 99:1, 0.7 mL/min, 214 nm UV detector, t_R = 16.81 min (major) and t_R = 21.21 min (minor). ^1H NMR (400 MHz, $CDCl_3$): δ 7.31 (m, 3H), 7.20 (d, *J* = 7.6 Hz, 2H), 7.09 (m, 2H), 7.06 (d, *J* = 7.6 Hz, 2H) (aromatic C*H*), 6.98 (s, 1H) (alkenyl C*H*), 6.93 (d, *J* = 7.6 Hz, 2H), 6.82 (d, *J* = 7.6 Hz, 2H) (aromatic C*H*), 6.07 (s, 1H) (N*H*), 3.32 (s, 2H) (C_{cage}-CH_2), 2.69 (q, *J* = 7.6 Hz, 2H), 2.54 (q, *J* = 7.6 Hz, 2H) (CH_2CH_3), 1.95 (s, 3H) ($COCH_3$), 1.28 (t, *J* = 7.6 Hz, 3H), 1.15 (t, *J* = 7.6 Hz, 3H) (CH_2CH_3). $^{13}C\{^1H\}$ NMR (101 MHz, $CDCl_3$): δ 168.0 (*C*O), 143.7, 142.8, 142.0, 140.2, 135.0, 134.7, 130.3, 129.8, 128.7, 128.6, 128.4, 128.1, 127.6 (aromatic *C* & alkenyl *C*), 83.6, 82.9 (cage *C*), 40.5 (C_{cage}-CH_2), 28.7, 28.6 (CH_2CH_3), 24.3 ($COCH_3$), 15.6, 15.4 (CH_3), the B_{cage}-*C* was not observed. ^{11}B NMR (128 MHz, $CDCl_3$): δ -1.1

(s, 1B) (B_{cage}-C), -4.5 (d, J = 130.6 Hz, 1B), -5.6 (d, J = 110.1 Hz, 1B), -11.4 (d, J = 88.3 Hz, 4B), -12.7 (d, J = 143.4 Hz, 1B), -14.0 (d, J = 162.6 Hz, 2B) (B_{cage}H). IR ν (film) cm^{-1}: 3190, 2966, 2574, 1686, 1537, 1281, 757, 699, 600, 540. HRMS (DART) Calcd for $C_{29}H_{40}{}^{10}B_2{}^{11}B_8NO^+$ [$M+H^+$]: 526.4108. Found: 526.4102.

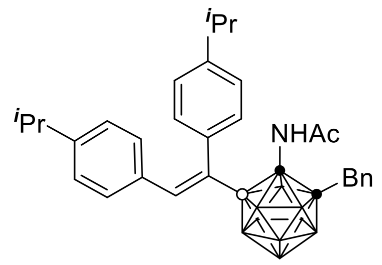
(*S*)-**3bk**

(*S*)-**3bk**: White solid. M.p. = 181-184 °C. TLC: R_f = 0.42 (*n*-hexane : ethyl acetate = 4 : 1). Method **B**: Yield: 90%. 88% ee, $[\alpha]_D^{29}$ = -27.2 (*c* = 1.00, CH_2Cl_2). Method **C**: Yield: 99%. 90% ee, $[\alpha]_D^{25}$ = -32.5 (*c* = 1.05, CH_2Cl_2). HPLC condition: Chiralpak IA (0.46 x 25 cm, 5 μm), acetonitrile/water = 95:5, 0.7 mL/min, 214 nm UV detector, t_R = 7.65 min (major) and t_R = 8.48 min (minor). ^{1}H NMR (400 MHz, $CDCl_3$): δ 7.31 (m, 3H), 7.23 (d, J = 7.6 Hz, 2H), 7.08 (m, 4H), 6.96 (m, 2H) (aromatic C*H*), 6.93 (s, 1H) (alkenyl C*H*), 6.81 (d, J = 8.0 Hz, 2H) (aromatic C*H*), 6.11 (s, 1H) (N*H*), 3.33 (s, 2H) (CH_2), 2.94 (m, 1H), 2.78 (m, 1H) ($CH(CH_3)_2$) 1.94 (s, 3H) ($COCH_3$), 1.29 (d, J = 6.8 Hz, 3H), 1.16 (d, J = 6.8 Hz, 3H) ($CH(CH_3)_2$). $^{13}C\{^1H\}$ NMR (101 MHz, $CDCl_3$): δ 168.1 (*C*O), 148.3, 147.4, 141.9, 140.4, 135.0, 134.8, 130.3, 129.8, 128.7, 128.2, 128.1, 127.3 (aromatic *C* & alkenyl *C*), 83.6, 82.8 (cage *C*), 40.4 (CH_2), 33.9 (*C*H), 24.3, 24.2, 23.9 (CH_3), the B_{cage}-*C* was not observed. ^{11}B NMR (128 MHz, $CDCl_3$): δ -1.1 (s, 1B) (B_{cage}-C), -4.6 (d, J = 129.3 Hz, 1B), -5.7 (d, J = 112.6 Hz, 1B), -11.4 (d, J = 85.8 Hz, 3B), -12.6 (d, J = 148.5 Hz, 2B), -13.9 (d, J = 163.8 Hz, 2B) (B_{cage}H). IR ν (film) cm^{-1}: 3272, 2953, 2554, 1692, 1516, 1265, 831, 699, 598, 547. HRMS (DART) Calcd for $C_{31}H_{44}{}^{10}B_2{}^{11}B_8NO^+$ [$M+H^+$]: 554.4421. Found: 554.4418.

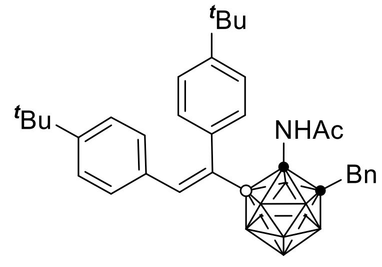
(*S*)-3bl

(*S*)-**3bl**: White solid. M.p. = 196-197 °C. TLC: R_f = 0.30 (*n*-hexane : ethyl acetate = 6 : 1). Method **B**: Yield: 75%. 94% ee, $[\alpha]_D^{29}$ = -23.8 (*c* = 0.90, CH_2Cl_2). Method **C**: Yield: 84%. 94% ee, $[\alpha]_D^{29}$ = -29.0 (*c* = 1.08, CH_2Cl_2). HPLC condition: Chiralpak IA (0.46 x 25 cm, 5 μm), acetonitrile/water = 99:1, 0.7 mL/min, 214 nm UV detector, t_R = 12.21 min (major) and t_R = 14.98 min (minor). ^{1}H NMR (400 MHz, $CDCl_3$): δ 7.39 (d, J = 8.0 Hz, 2H), 7.31 (m, 3H), 7.09 (m, 6H) (aromatic C*H*), 6.96 (s, 1H) (alkenyl C*H*), 6.82 (d, J = 8.0 Hz, 2H) (aromatic C*H*), 6.07 (s, 1H) (N*H*), 3.32 (s, 2H) (CH_2), 1.94 (s, 3H) ($COCH_3$), 1.36

(s, 9H), 1.23 (s, 9H) (C(C*H*$_3$)$_3$). ^{13}C{^{1}H} NMR (101 MHz, CDCl$_3$): δ 168.1 (*C*O), 150.5, 149.8, 141.8, 140.0, 135.0, 134.4, 130.3, 129.5, 128.7, 128.1, 127.9, 126.1, 125.0 (aromatic *C* & alkenyl *C*), 83.6, 82.8 (cage *C*), 40.4 (*C*H$_2$), 34.7, 34.6 (*C*(CH$_3$)$_3$), 31.6, 31.3 (C(*C*H$_3$)$_3$), 24.3 (CO*C*H$_3$), the B$_{cage}$-*C* was not observed. ^{11}B NMR (128 MHz, CDCl$_3$): δ -1.1 (s, 1B) (*B*$_{cage}$-C), -4.5 (d, *J* = 144.6 Hz, 1B), -5.7 (d, *J* = 74.2 Hz, 1B), -11.5 (d, *J* = 92.2 Hz, 4B), -12.5 (d, *J* = 157.4 Hz, 1B), -13.9 (d, *J* = 153.6 Hz, 2B) (*B*$_{cage}$H). IR ν (film) cm^{-1}: 3256, 2961, 2577, 1688, 1269, 701, 599, 555. HRMS (DART) Calcd for $C_{33}H_{48}{}^{10}B_2{}^{11}B_8NO^+$ [M+H$^+$]: 582.4734. Found: 582.4732.

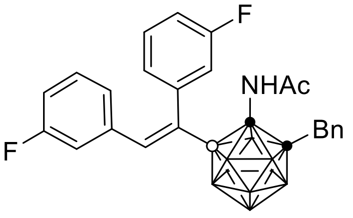

(*S*)-**3bm**

(*S*)-**3bm**: White solid. M.p. = 203-205 °C. TLC: R$_f$ = 0.48 (*n*-hexane : ethyl acetate = 4 : 1). Method **A**: Yield: 94%. 99% ee, $[\alpha]_D^{26}$ = 11.1 (*c* = 0.8, CH$_2$Cl$_2$). HPLC condition: Chiralpak IA (0.46 x 25 cm, 5 μm), acetonitrile/water = 99:1, 0.7 mL/min, 214 nm UV detector, t$_R$ = 31.71 min (major) and t$_R$ = 37.83 min (minor). ^{1}H NMR (400 MHz, CDCl$_3$): δ 7.33 (m, 4H), 7.10 (m, 3H), 6.99 (m, 2H), 6.90 (d, *J* = 7.6 Hz, 1H), 6.82 (m, 2H), 6.73 (d, *J* = 7.6 Hz, 1H), 6.55 (d, *J* = 10.4 Hz, 1H), (aromatic C*H* & alkenyl C*H*), 6.13 (s, 1H) (N*H*), 3.36 (s, 2H) (C*H*$_2$), 2.02 (s, 3H) (COC*H*$_3$). ^{13}C{^{1}H} NMR (101 MHz, CDCl$_3$): δ 168.0 (*C*O), 164.1 (d, $^1J_{CF}$ = 248.9 Hz), 164.1 (d, $^1J_{CF}$ = 246.1 Hz), 144.7 (d, $^3J_{CF}$ = 7.5 Hz), 141.1, 138.9 (d, $^3J_{CF}$ = 7.6 Hz), 134.7, 130.8 (d, $^3J_{CF}$ = 8.5 Hz), 130.3, 129.6 (d, $^3J_{CF}$ = 8.4 Hz), 128.8, 128.3, 125.6 (d, $^4J_{CF}$ = 2.4 Hz), 124.1 (d, $^4J_{CF}$ = 2.4 Hz), 116.0 (d, $^2J_{CF}$ = 22.2 Hz), 115.3 (d, $^2J_{CF}$ = 21.3 Hz), 114.6 (d, $^2J_{CF}$ = 21.6 Hz), 114.0 (d, $^2J_{CF}$ = 20.9 Hz) (aromatic *C* & alkenyl *C*), 84.5, 82.8 (cage *C*), 40.5 (*C*H$_2$), 24.2 (CO*C*H$_3$), the B$_{cage}$-*C* was not observed. ^{11}B NMR (128 MHz, CDCl$_3$): δ -1.6 (s, 1B) (*B*$_{cage}$-C), -4.4 (d, *J* = 119.0 Hz, 1B), -5.4 (d, *J* = 103.7 Hz, 1B), -11.6 (d, *J* = 74.2 Hz, 4B), -12.3 (d, *J* = 117.8 Hz, 1B), -13.9 (d, *J* = 166.4 Hz, 2B) (*B*$_{cage}$H). ^{19}F NMR (376 MHz, CDCl$_3$): δ -111.8 (m, 1F), -113.3 (m, 1F). IR ν (film) cm^{-1}: 3253, 3014, 2581, 1686, 1578, 1277, 781, 702, 593, 520. HRMS (DART) Calcd for $C_{25}H_{30}{}^{10}B_2{}^{11}B_8F_2NO^+$ [M+H$^+$]: 506.3293. Found: 506.3292.

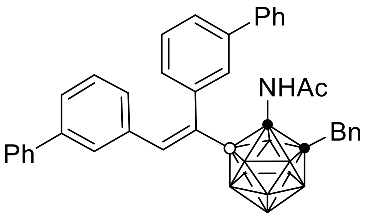
(S)-**3bn**

(S)-**3bn**: White solid. M.p. = 185-186 °C. TLC: R_f = 0.31 (*n*-hexane : ethyl acetate = 4 : 1). Method **B**: Yield: 96%. 94% ee, $[\alpha]_D^{28}$ = -12.0 (*c* = 1.30, CH_2Cl_2). Method **C**: Yield: 99%. 95% ee, $[\alpha]_D^{28}$ = -12.2 (*c* = 1.22, CH_2Cl_2). HPLC condition: Chiralpak IA (0.46 x 25 cm, 5 μm), acetonitrile/water = 95:5, 0.7 mL/min, 214 nm UV detector, t_R = 15.04 min (major) and t_R = 17.59 min (minor). ^{1}H NMR (400 MHz, $CDCl_3$): δ 7.53 (m, 3H), 7.44 (m, 4H), 7.34 (m, 6H), 7.27 (m, 5H), 7.21 (m, 2H), 7.15 (m, 1H), 7.10 (m, 2H), 6.99 (d, *J* = 7.2 Hz, 1H) (aromatic C*H* & alkenyl C*H*), 6.13 (s, 1H) (N*H*), 3.35 (s, 2H) (CH_2), 1.86 (s, 3H) (COCH_3). ^{13}C{^{1}H} NMR (101 MHz, $CDCl_3$): δ 168.0 (*C*O), 143.4, 142.2, 142.1, 140.9, 140.8, 140.7, 137.6, 134.9, 130.3, 130.0, 129.0, 128.8, 128.7, 128.6, 128.2, 127.7, 127.5, 127.4, 127.3, 127.2, 127.0, 126.1, 125.6 (aromatic *C* & alkenyl *C*), 84.2, 82.9 (cage *C*), 40.5 (*C*H_2), 24.1 (CO*C*H_3), the B_{cage}-*C* was not observed. ^{11}B NMR (128 MHz, $CDCl_3$): δ -1.3 (s, 1B) (B_{cage}-C), -4.3 (d, *J* = 140.8 Hz, 1B), -5.5 (d, *J* = 103.7 Hz, 1B), -11.4 (d, *J* = 79.4 Hz, 4B), -12.3 (d, *J* = 113.4 Hz, 1B), -13.7 (d, *J* = 166.4 Hz, 2B) (B_{cage}H). IR ν (film) cm^{-1}: 3199, 3026, 2556, 1690, 1530, 1271, 751, 697. HRMS (DART) Calcd for $C_{37}H_{40}{}^{10}B_2{}^{11}B_8NO^+$ [M+H$^+$]: 622.4108. Found: 622.4097.

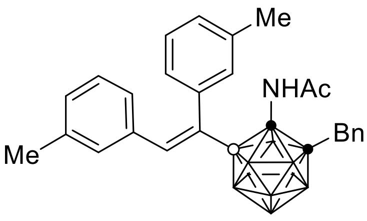
(S)-**3bo**

(S)-**3bo**: White solid. M.p. = 180-184 °C. TLC: R_f = 0.42 (*n*-hexane : ethyl acetate = 4 : 1). Method **B**: Yield: 97%. 97% ee, $[\alpha]_D^{27}$ = -32.6 (*c* = 1.15, CH_2Cl_2). Method **C**: Yield: 98%. 97% ee, $[\alpha]_D^{27}$ = -34.4 (*c* = 1.16, CH_2Cl_2). HPLC condition: IC, acetonitrile/water = 98:2, 0.7 mL/min, 214 nm UV detector, t_R = 12.57 min (minor) and t_R = 14.65 min (major). ^{1}H NMR (400 MHz, $CDCl_3$): δ 7.31 (m, 3H), 7.24 (m, 1H), 7.09 (m, 3H), 6.97 (m, 3H), 6.93 (m, 2H) (aromatic C*H*), 6.77 (s, 1H) (alkenyl C*H*), 6.65 (d, *J* = 7.2 Hz, 1H) (aromatic C*H*), 6.12 (s, 1H) (N*H*), 3.31 (s, 2H) (CH_2), 2.34 (s, 3H), 2.16 (s, 3H), 1.95 (s, 3H) (CH_3). ^{13}C{^{1}H} NMR (101 MHz, $CDCl_3$): δ 168.1 (*C*O), 142.9, 142.1, 138.7, 137.5, 137.1, 130.3, 129.0, 128.7, 128.2, 128.1, 127.9, 127.6, 126.6, 125.5 (aromatic *C* & alkenyl *C*), 83.7, 82.8 (cage *C*), 40.4 (*C*H_2), 24.2, 21.7, 21.4 (*C*H_3), the B_{cage}-*C* was not observed. ^{11}B NMR (128 MHz, $CDCl_3$): δ -1.2 (s, 1B) (B_{cage}-C), -4.6 (d, *J* = 117.8 Hz, 1B), -5.7 (d, *J* = 105.0 Hz, 1B), -11.5 (d, *J* = 85.8 Hz, 3B), -12.6 (d, *J* = 133.1 Hz, 2B), -13.8 (d, *J* = 174.1 Hz, 2B) (B_{cage}H). IR ν (film) cm^{-1}: 3190, 3022, 2562, 1683, 1494, 1280, 699, 665, 607. HRMS (DART) Calcd for $C_{27}H_{36}{}^{10}B_2{}^{11}B_8NO^+$ [M+H$^+$]: 498.3795. Found: 498.3795.

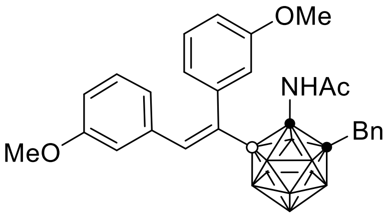

(*S*)-**3bp**

(*S*)-**3bp**: White solid. M.p. = 190-192 °C. TLC: R_f = 0.20 (*n*-hexane : ethyl acetate = 4 : 1). Method **B**: Yield: 85%. 99% ee, $[\alpha]_D^{28}$ = -16.9 (*c* = 1.00, CH_2Cl_2). Method **C**: Yield: 99%. 98% ee, $[\alpha]_D^{28}$ = -18.5 (*c* = 0.35, CH_2Cl_2). HPLC condition: Chiralpak IA (0.46 x 25 cm, 5 μm), acetonitrile/water = 98:2, 0.7 mL/min, 214 nm UV detector, t_R = 33.01 min (major) and t_R = 38.28 min (minor). ^{1}H NMR (400 MHz, $CDCl_3$): *δ* 7.32 (m, 3H), 7.29 (m, 1H), 7.11 (m, 2H), 7.05 (t, *J* = 8.0 Hz, 1H), 6.99 (s, 1H), 6.81 (d, *J* = 8.0 Hz, 1H), 6.73 (m, 2H), 6.67 (d, *J* = 8.4 Hz, 1H), 6.61 (d, *J* = 7.6 Hz, 1H), 6.45 (s, 1H) (aromatic C*H* & alkenyl C*H*), 6.14 (s, 1H) (N*H*), 3.79 (s, 3H), 3.49 (s, 3H) (OCH_3), 3.33 (s, 2H) (CH_2), 1.98 (s, 3H) (COCH_3). ^{13}C{^{1}H} NMR (101 MHz, $CDCl_3$): *δ* 168.2 (*C*O), 160.4, 159.1, 144.5, 141.8, 138.3, 134.9, 130.3, 130.2, 129.0, 128.7, 128.2, 122.7, 120.5, 114.5, 114.2, 113.7, 112.2 (aromatic *C* & alkenyl *C*), 84.0, 82.8 (cage *C*), 55.4, 54.5 (O*C*H_3), 40.4 (*C*H_2), 24.2 (CO*C*H_3), the B_{cage}-*C* was not observed. ^{11}B NMR (128 MHz, $CDCl_3$): *δ* -1.4 (s, 1B) (*B*$_{cage}$-C), -4.5 (d, *J* = 125.4 Hz, 1B), -5.6 (d, *J* = 117.8 Hz, 1B), -11.5 (d, *J* = 85.8 Hz, 3B), -12.4 (d, *J* = 119.0 Hz, 2B), -13.8 (d, *J* = 140.8 Hz, 2B) (*B*$_{cage}$H). IR ν (film) cm^{-1}: 3275, 2952, 2577, 1693, 1519, 1265, 919, 701, 525. HRMS (DART) Calcd for $C_{27}H_{36}{}^{10}B_2{}^{11}B_8NO_3^+$ [M+H$^+$]: 530.3693. Found: 530.3692.

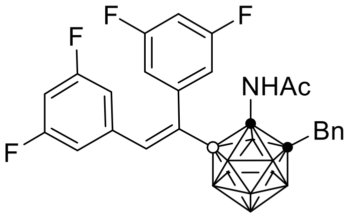

(*S*)-**3bq**

(*S*)-**3bq**: White solid. M.p. = 191-193 °C. TLC: R_f = 0.43 (*n*-hexane : ethyl acetate = 4 : 1). Method **A**: Yield: 91%. 96% ee, $[\alpha]_D^{28}$ = 50.5 (*c* = 1.16, CH_2Cl_2). HPLC condition: Chiralpak IE3 (0.46 x 25 cm, 3 μm), acetonitrile/water = 9:1, 0.7 mL/min, 214 nm UV detector, t_R = 6.13 min (minor) and t_R = 6.51 min (major). ^{1}H NMR (400 MHz, $CDCl_3$): *δ* 7.34 (m, 3H), 7.12 (m, 2H) (aromatic C*H*), 6.94 (s, 1H) (alkenyl C*H*), 6.76 (t, *J* = 8.4 Hz, 1H), 6.61 (m, 3H), 6.43 (d, *J* = 7.2 Hz, 2H) (aromatic C*H*), 6.23 (s, 1H) (N*H*), 3.39 (s, 2H) (CH_2), 2.07 (s, 3H) (COCH_3). ^{13}C{^{1}H} NMR (101 MHz, $CDCl_3$): *δ* 168.0 (*C*O), 163.4 (dd, $^1J_{CF}$ = 251.7 Hz, $^3J_{CF}$ = 13.0 Hz), 162.8 (dd, $^1J_{CF}$ = 249.3 Hz, $^3J_{CF}$ = 12.9 Hz), 145.4 (t, $^3J_{CF}$ = 9.6 Hz), 140.1, 139.5 (t, $^3J_{CF}$ = 9.3 Hz), 134.6, 130.3, 128.9, 128.3, 112.2 (m), 111.1 (m), 103.4 (t, $^2J_{CF}$ = 25.8 Hz), 102.7 (t, $^2J_{CF}$ = 25.4 Hz) (aromatic *C* & alkenyl *C*), 85.2, 82.8 (cage *C*), 40.6 (*C*H_2), 24.1 (CO*C*H_3), the B_{cage}-*C* was not observed. ^{11}B NMR (128 MHz, $CDCl_3$): *δ* -2.1 (s, 1B) (*B*$_{cage}$-C), -4.3 (d, *J* = 120.3 Hz, 1B), -5.1 (d, *J*

=111.4 Hz, 1B), -11.5 (m, 5B), -13.2 (m, 2B) (B_{cage}H). ^{19}F NMR (376 MHz, $CDCl_3$): δ -108.2 (s, 2F), -109.8 (t, J = 8.3 Hz, 2F). IR ν (film) cm^{-1}: 3305, 2594, 1695, 1618, 1583, 1229, 912, 751, 677, 570. HRMS (DART) Calcd for $C_{25}H_{28}{}^{10}B_2{}^{11}B_8F_4NO^+$ [M+H$^+$]: 542.3105. Found: 542.3099.

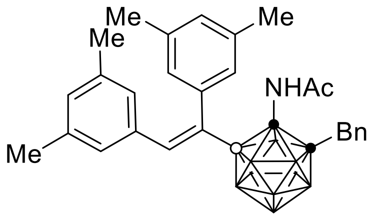

(S)-**3br**

(S)-**3br**: White solid. M.p. = 170-172 °C. TLC: R_f = 0.42 (n-hexane : ethyl acetate = 4 : 1). Method **B**: Yield: 95%. 94% ee, $[\alpha]_D^{28}$ = -38.7 (c = 0.60, CH_2Cl_2). Method **C**: Yield: 94%. 97% ee, $[\alpha]_D^{28}$ = -41.4 (c = 0.60, CH_2Cl_2). HPLC condition: Chiralpak IA (0.46 x 25 cm, 5 μm), acetonitrile/water = 99:1, 0.7 mL/min, 214 nm UV detector, t_R = 11.89 min (minor) and t_R = 13.01 min (major). ^{1}H NMR (400 MHz, $CDCl_3$): δ 7.31 (m, 2H), 7.26 (m, 2H), 7.09 (m, 2H), 6.92 (s, 2H), 6.76 (m, 2H), 6.53 (s, 2H) (aromatic CH & alkenyl CH), 6.10 (s, 1H) (NH), 3.31 (s, 2H) (CH_2), 2.30 (s, 6H), 2.16 (s, 6H) (phenyl-CH_3), 1.94 (s, 3H) (COCH_3). ^{13}C{^{1}H} NMR (101 MHz, $CDCl_3$): δ 168.0 (CO), 142.9, 142.1, 138.6, 137.3, 137.1, 135.0, 130.4, 129.1, 128.7, 128.3, 128.1, 127.8, 126.1 (aromatic C & alkenyl C), 83.5, 82.8 (cage C), 40.4 (CH$_2$), 24.1 (COCH$_3$), 21.5, 21.3 (phenyl-CH$_3$), the B_{cage}-C was not observed. ^{11}B NMR (128 MHz, $CDCl_3$): δ -1.1 (s, 1B) (B_{cage}-C), -4.6 (d, J = 116.5 Hz, 1B), -5.7 (d, J = 112.6 Hz, 1B), -11.4 (d, J = 81.9 Hz, 4B), -12.7 (d, J = 170.2 Hz, 1B), -13.9 (d, J = 133.1 Hz, 2B) (B_{cage}H). IR ν (film) cm^{-1}: 2918, 2573, 1711,1486, 1255, 693, 555. HRMS (DART) Calcd for $C_{29}H_{40}{}^{10}B_2{}^{11}B_8NO^+$ [M+H$^+$]: 526.4108. Found: 526.4101.

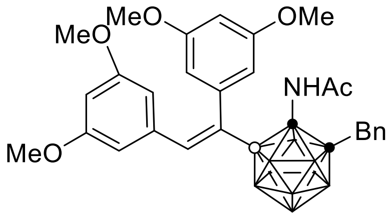

(S)-**3bs**

(S)-**3bs**: White solid. M.p. = 181-184 °C. TLC: R_f = 0.21 (n-hexane : ethyl acetate = 3 : 1). Method **C**: Yield: 90%. 97% ee, $[\alpha]_D^{29}$ = -6.2 (c = 1.03, CH_2Cl_2). HPLC condition: Chiralpak IA (0.46 x 25 cm, 5 μm), acetonitrile/water = 9:1, 0.7 mL/min, 214 nm UV detector, t_R = 12.53 min (major) and t_R = 18.35 min (minor). ^{1}H NMR (400 MHz, $CDCl_3$): δ 7.32 (m, 3H), 7.10 (m, 2H), 6.91 (s, 1H), 6.36 (m, 1H), 6.32 (m, 2H), 6.25 (m, 1H), 6.20 (m, 3H) (aromatic CH, alkenyl CH & NH), 3.76 (s, 6H), 3.55 (s, 6H) (OCH_3), 3.34 (s, 2H) (CH_2), 2.00 (s, 3H) (COCH_3). ^{13}C{^{1}H} NMR (101 MHz, $CDCl_3$): δ 168.3 (CO), 161.6, 160.2, 145.2, 141.6, 138.7, 134.9, 130.3, 128.7, 128.1, 107.3, 128.1, 106.2, 101.0, 98.5 (aromatic C & alkenyl C), 84.0, 82.8 (cage C), 55.6, 55.2 (OCH$_3$), 40.4 (CH$_2$), 24.2 (COCH$_3$), the B_{cage}-C was not observed. ^{11}B NMR (128 MHz, $CDCl_3$): δ -1.4 (s, 1B) (B_{cage}-C), -4.3 (d, J = 124.2 Hz, 1B), -5.7 (d, J = 108.8 Hz, 1B), -11.4 (d, J =

80.6 Hz, 4B), -12.4 (d, J = 80.6 Hz, 1B), -13.6 (d, J = 172.8 Hz, 2B) (B_{cage}H). IR ν (film) cm^{-1}: 2998, 2557, 1703, 1581, 1421, 1277, 1198, 1152, 1062, 699, 523. HRMS (DART) Calcd for $C_{29}H_{40}{}^{10}B_2{}^{11}B_8NO_5^+$ [M+H$^+$]: 590.3904. Found: 590.3890.

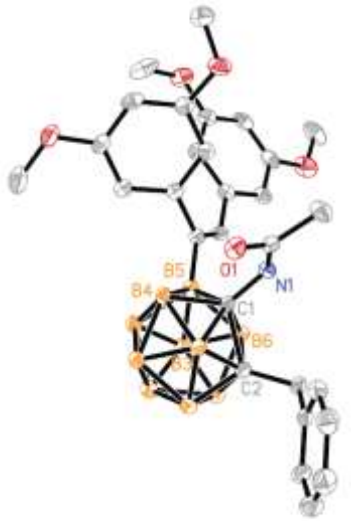

Supplementary Figure 4. Molecular structure of (*S*)-**3bs**

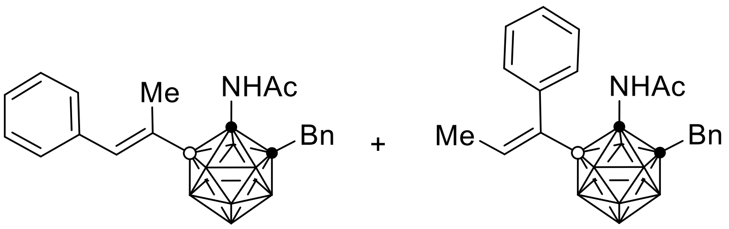

(*S*)-**3bt** (*S*)-**3bt'**

Method **B**: Yield: 74% ((*S*)-**3ft**:(*S*)-**3ft'** = 81:19 determined by ^{1}H NMR spectrum of the crude mixture) TLC: R_f = 0.45 (*n*-hexane : ethyl acetate = 4 : 1).

(*S*)-**3bt**: White solid. M.p. = 151-153 °C. 58% ee, $[\alpha]_D^{29}$ = 17.0 (c = 1.18, CH_2Cl_2). HPLC condition: Chiralpak IB (0.46 x 25 cm, 5 μm), acetonitrile/water = 95:5, 0.7 mL/min, 214 nm UV detector, t_R = 16.07 min (minor) and t_R = 19.09 min (major). ^{1}H NMR (400 MHz, $CDCl_3$): δ 7.34 (m, 5H), 7.28 (m, 2H), 7.24 (m, 1H), 7.16 (m, 2H) (aromatic C*H*), 6.88 (s, 1H) (alkenyl C*H*), 6.46 (s, 1H) (N*H*), 3.44 (m, 2H) (CH_2), 2.12 (s, 3H) ($COCH_3$), 2.03 (s, 3H) (alkenyl C-CH_3). $^{13}C\{^1H\}$ NMR (101 MHz, $CDCl_3$): δ 168.3 (*C*O), 139.7, 138.3, 135.0, 130.4, 129.2, 128.8, 128.3, 128.2, 127.0 (aromatic *C* & alkenyl *C*), 84.1, 83.4 (cage *C*), 40.6 (C_{cage}-CH_2), 24.3 ($COCH_3$), 20.1 (alkenyl C-CH_3), the B_{cage}-*C* was not observed. ^{11}B NMR (128 MHz, $CDCl_3$): δ -0.5 (s, 1B) (B_{cage}-C), -4.4 (d, J = 107.8 Hz, 1B), -5.8 (d, J = 90.8 Hz, 1B), -11.7 (d, J = 81.9 Hz, 4B), -12.1 (d, J = 78.2 Hz, 1B), -13.9 (d, J = 87.4 Hz, 2B) (B_{cage}H). IR ν (film) cm^{-1}: 3256, 2922, 2559, 1688, 1528, 1267, 1028, 751, 701, 589. HRMS (DART) Calcd for $C_{20}H_{30}{}^{10}B_2{}^{11}B_8NO^+$ [M+H$^+$]: 408.3325. Found: 408.3322.

(*S*)-**3bt'**: White solid. M.p. = 144-146 °C. 60% ee, $[\alpha]_D^{29}$ = -16.9 (*c* = 0.50, CH_2Cl_2). HPLC condition: Chiralpak IG (0.46 x 25 cm, 5 μm), acetonitrile/water = 8:2, 0.7 mL/min, 214 nm UV detector, t_R = 5.70 min (major) and t_R = 6.31 min (minor). ^{1}H NMR (400 MHz, $CDCl_3$): δ 7.37 (t, *J* = 7.6 Hz, 2H), 7.30 (m, 3H), 7.24 (m, 1H), 7.08 (m, 4H) (aromatic C*H*), 6.32 (q, *J* = 6.8 Hz, 1H) (alkenyl C*H*), 6.04 (s, 1H) (N*H*), 3.29 (m, 2H) (CH_2), 1.96 (s, 3H) ($COCH_3$), 1.59 (d, *J* = 6.8 Hz, 3H) (alkenyl C-CH_3). ^{13}C{^{1}H} NMR (101 MHz, $CDCl_3$): δ 168.0 (*C*O), 142.4, 140.3, 135.0, 130.3, 128.7, 128.6, 128.1, 126.4 (aromatic *C* & alkenyl *C*), 83.4, 82.7 (cage *C*), 40.4 (C_{cage}-CH_2), 24.2 ($COCH_3$), 16.7 (alkenyl C-CH_3), the B_{cage}-*C* was not observed. ^{11}B NMR (128 MHz, $CDCl_3$): δ -1.4 (s, 1B) (B_{cage}-C), -4.6 (d, *J* = 109.7 Hz, 1B), -5.8 (d, *J* = 103.2 Hz, 1B), -11.5 (m, 4B), -12.5 (d, *J* = 132.5 Hz, 1B), -13.9 (d, *J* = 102.4 Hz, 1B), -13.6 (d, *J* = 93.4 Hz, 1B) (B_{cage}H). IR ν (film) cm^{-1}: 3258, 2923, 2573, 1682, 1537, 1279, 1046, 748, 698, 590. HRMS (DART) Calcd for $C_{20}H_{30}{}^{10}B_2{}^{11}B_8NO^+$ [M+H$^+$]: 408.3325. Found: 408.3322.

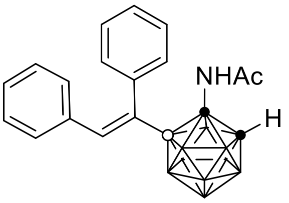

(*S*)-**3ca**

(*S*)-**3ca**: White solid. m,p. = 182-183 °C. TLC: R_f = 0.28 (*n*-hexane : ethyl acetate = 4 : 1). Method **B**: Yield: 70%. 80% ee, $[\alpha]_D^{28}$ = -46.8 (*c* = 1.10, CH_2Cl_2). HPLC condition: Chiralpak IA (0.46 x 25 cm, 5 μm), acetonitrile/water = 95:5, 0.7 mL/min, 214 nm UV detector, t_R = 8.77 min (minor) and t_R = 9.63 min (major). ^{1}H NMR (400 MHz, $CDCl_3$): δ 7.38 (m, 2H), 7.30 (d, *J* = 7.2 Hz, 1H), 7.16 (d, *J* = 7.2 Hz, 2H), 7.10 (m, 4H), 6,91 (m, 2H) (aromatic C*H* & alkenyl C*H*), 5.90 (s, 1H) (N*H*), 5.24 (cage C*H*), 1.74 (s, 1H) ($COCH_3$). ^{13}C{^{1}H} NMR (101 MHz, $CDCl_3$): δ 168.1 (*C*O), 142.6, 1423, 136.9, 129.6, 129.2, 128.3, 128.0, 127.4, 126.9 (aromatic *C* & alkenyl *C*), 59.5 (cage *C*H), 23.8 ($COCH_3$), the C_{cage}-NHAc and B_{cage}-*C* was not observed. ^{11}B NMR (128 MHz, $CDCl_3$): δ -2.1 (s, 1B) (B_{cage}-C), -3.5 (d, *J* = 98.6 Hz, 1B), -6.7 (d, *J* = 105.0 Hz, 1B), -10.5 (d, *J* = 99.8 Hz, 1B), -11.4 (d, *J* = 124.2 Hz, 2B), -12.3 (d, *J* = 99.8 Hz, 2B), -15.2 (d, *J* = 115.2 Hz, 1B), -16.7 (d, *J* = 158.7 Hz, 1B) (B_{cage}H). IR ν (film) cm^{-1}: 3244, 3017, 2587, 1664, 1530, 1273, 998, 752, 690, 524. HRMS (DART) Calcd for $C_{18}H_{26}{}^{10}B_2{}^{11}B_8NO^+$ [M+H$^+$]: 380.3012. Found: 380.3010.

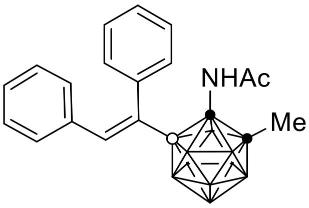

(*S*)-**3aa**

(*S*)-**3aa**: White solid. M.p. = 218-220 ºC. TLC: R_f = 0.20 (*n*-hexane : ethyl acetate = 4 : 1). Method **A**: Yield: 99%. 95% ee, $[\alpha]_D^{25}$ = 17.3 (*c* = 1.15, CH_2Cl_2). HPLC condition: Chiralpak IA (0.46 x 25 cm, 5 μm), acetonitrile/water = 99:1, 0.7 mL/min, 214 nm UV detector, t_R = 17.55 min (major) and t_R = 19.60 min (minor). ^{1}H NMR (400 MHz, $CDCl_3$): δ 7.35 (t, *J* = 7.6 Hz, 2H), 7.28 (m, 1H), 7.14 (d, *J* = 7.2 Hz, 2H), 7.09 (m, 3H) (aromatic C*H*), 7.04 (s, 1H) (alkenyl C*H*), 6.90 (m, 2H) (aromatic C*H*), 6.06 (s, 1H) (N*H*), 1.90 (s, 3H), 1.89 (s, 3H) (C*H*3). ^{13}C{^{1}H} NMR (101 MHz, $CDCl_3$): δ 170.0 (*C*O), 142.9, 142.1, 137.2, 129.7, 129.1, 128.5, 128.1, 127.4, 126.9 (aromatic *C* & alkenyl *C*), 81.4, 78.9 (cage *C*), 24.1, 22.4 (*C*H3), the B_{cage}-*C* was not observed. ^{11}B NMR (128 MHz, $CDCl_3$): δ -1.1 (s, 1B) (*B*cage-C), -5.5 (d, *J* = 78.1 Hz, 2B), -10.6 (d, *J* = 110.1 Hz, 3B), -11.7 (d, *J* = 147.2 Hz, 1B), -12.7 (d, *J* = 98.6 Hz, 3B) (*B*cageH). IR ν (film) cm^{-1}: 3254, 2959, 2580, 1692, 1530, 1271, 728, 693. HRMS (DART) Calcd for $C_{19}H_{28}{}^{10}B_2{}^{11}B_8NO^+$ [M+H$^+$]: 394.3169. Found: 394.3170.

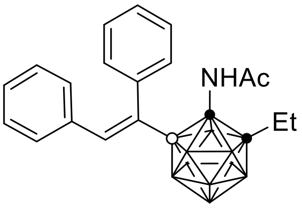

(*S*)-**3da**

(*S*)-**3da**: White solid. M.p. = 196-197 ºC. TLC: R_f = 0.24 (*n*-hexane : ethyl acetate = 4 : 1). Method **A**: Yield: 99%. 96% ee, $[\alpha]_D^{25}$ = 16.7 (*c* = 1.12, CH_2Cl_2). HPLC condition: Chiralpak IA (0.46 x 25 cm, 5 μm), acetonitrile/water = 99:1, 0.7 mL/min, 214 nm UV detector, t_R = 15.77 min (major) and t_R = 17.51 min (minor). ^{1}H NMR (400 MHz, $CDCl_3$): δ 7.35 (m, 2H), 7.29 (m, 1H), 7.14 (d, *J* = 7.6 Hz, 2H), 7.09 (m, 3H) (aromatic C*H*), 7.05 (s, 1H) (alkenyl C*H*), 6.89 (m, 2H) (aromatic C*H*), 5.92 (s, 1H) (N*H*), 2.09 (m, 2H) (C*H*2), 1.88 (s, 3H) (COC*H*3), 1.09 (t, *J* = 7.6 Hz, 3H) (CH2C*H*3). ^{13}C{^{1}H} NMR (101 MHz, $CDCl_3$): δ 167.9 (*C*O), 142.9, 142.1, 137.2, 129.7, 129.1, 128.6, 128.0, 127.4, 126.8 (aromatic *C* & alkenyl *C*), 85.1, 82.9 (cage *C*), 27.8, 27.1, 13.6 (*C*H2 & *C*H3), the B_{cage}-*C* was not observed. ^{11}B NMR (128 MHz, $CDCl_3$): δ -1.3 (s, 1B) (*B*cage-C), -4.8 (d, *J* = 99.8 Hz, 1B), -5.6 (d, *J* = 103.7 Hz, 1B), -11.1 (d, *J* = 87.0 Hz, 2B), -11.7 (d, *J* = 111.4 Hz, 2B), -12.7 (d, *J* = 110.1 Hz, 1B), -14.2 (d, *J* = 108.8 Hz, 1B), -14.7 (d, *J* = 92.2 Hz, 1B) (*B*cageH). IR ν (film) cm^{-1}: 3277, 2982, 2570, 1695, 1513, 1264, 1014, 694, 582. HRMS (DART) Calcd for $C_{20}H_{30}{}^{10}B_2{}^{11}B_8NO^+$ [M+H$^+$]: 408.3325. Found: 408.3321.

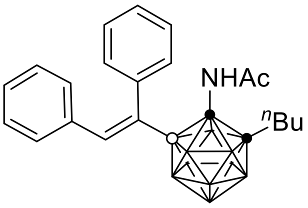

(*S*)-**3ea**

(*S*)-**3ea**: White solid. M.p. = 210-212 °C. TLC: R_f = 0.46 (*n*-hexane : ethyl acetate = 4 : 1). Method **A**: Yield: 94%. 95% ee, $[\alpha]_D^{29}$ = 6.6 (*c* = 1.00, CH_2Cl_2). HPLC condition: Chiralpak IB (0.46 x 25 cm, 5 μm), acetonitrile/water = 9:1, 0.7 mL/min, 214 nm UV detector, t_R = 7.17 min (minor) and t_R = 7.54 min (major). ^{1}H NMR (400 MHz, $CDCl_3$): δ 7.35 (t, *J* = 7.6 Hz, 2H), 7.29 (m, 1H), 7.13 (d, *J* = 6.8 Hz, 2H), 7.08 (m, 3H) (aromatic C*H*), 7.04 (s, 1H) (alkenyl C*H*), 6.89 (m, 2H) (aromatic C*H*), 5.92 (s, 1H) (N*H*), 2.00 (m, 2H) (C_{cage}-CH_2), 1.89 (s, 3H) (COCH_3), 1.45 (m, 2H), 1.25 (m, 2H) (CH_2), 0.88 (t, *J* = 7.2 Hz, 3H) (CH_2CH_3). ^{13}C{^{1}H} NMR (101 MHz, $CDCl_3$): δ 168.0 (*C*O), 142.9, 142.1, 137.2, 129.7, 129.1, 128.5, 128.0, 127.4, 126.8 (aromatic *C* & alkenyl *C*), 84.5, 82.9 (cage *C*), 34.0, 31.4, 24.1, 22.5, 13.8 (*C*H_2 & *C*H_3), the B_{cage}-*C* was not observed. ^{11}B NMR (128 MHz, $CDCl_3$): δ -1.4 (s, 1B) (*B*$_{cage}$-C), -4.8 (d, *J* = 107.5 Hz, 1B), -5.6 (d, *J* = 92.2 Hz, 1B), -11.0 (d, *J* = 119.0 Hz, 2B), -11.8 (d, *J* = 97.3 Hz, 2B), -12.8 (d, *J* = 115.2 Hz, 1B), -14.1 (d, *J* = 156.2 Hz, 2B) (*B*$_{cage}$H). IR ν (film) cm^{-1}: 3254, 2963, 2575, 1690, 1529, 1272,1028, 693, 521. HRMS (DART) Calcd for $C_{22}H_{34}{}^{10}B_2{}^{11}B_8NO^+$ [M+H$^+$]: 436.3638. Found: 436.3637.

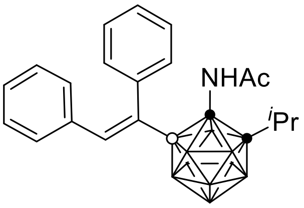

(*S*)-**3fa**

(*S*)-**3fa**: White solid. M.p. = 236-238 °C. TLC: R_f = 0.24 (*n*-hexane : ethyl acetate = 4 : 1). Method **A**: Yield: 99%. 92% ee, $[\alpha]_D^{28}$ = 36.8 (*c* = 1.19, CH_2Cl_2). HPLC condition: Chiralpak IA (0.46 x 25 cm, 5 μm), acetonitrile/water = 99:1, 0.7 mL/min, 214 nm UV detector, t_R = 15.54 min (major) and t_R = 17.70 min (minor). ^{1}H NMR (400 MHz, $CDCl_3$): δ 7.34 (t, *J* = 8.0 Hz, 2H), 7.28 (m, 1H), 7.13 (d, *J* = 7.6 Hz, 2H), 7.08 (m, 3H) (aromatic C*H*), 7.05 (s, 1H) (alkenyl C*H*), 6.89 (m, 2H) (aromatic C*H*), 5.94 (s, 1H) (N*H*), 2.13 (m, 1H) (C*H*($CH_3)_2$), 1.88 (s, 3H) (COCH_3), 1.15 (m, 6H) (CH(C$H_3)_2$). ^{13}C{^{1}H} NMR (101 MHz, $CDCl_3$): δ 168.0 (*C*O), 142.8, 142.0, 137.2, 129.7, 129.1, 128.5, 128.0, 127.4, 126.8 (aromatic *C* & alkenyl *C*), 91.0, 84.8 (cage *C*), 30.7 (*C*H), 24.2, 24.0, 23.8 (*C*H_3), the B_{cage}-*C* was not observed. ^{11}B NMR (128 MHz, $CDCl_3$): δ -1.3 (s, 1B) (*B*$_{cage}$-C), -3.9 (d, *J* = 93.4 Hz, 1B), -6.0 (d, *J* = 94.7 Hz, 1B), -11.7 (d, *J* = 79.4 Hz, 3B), -12.3 (d, *J* = 78.1 Hz, 2B), -14.7 (d, *J* = 106.2 Hz, 1B), -15.5 (d, *J* = 97.3 Hz, 1B) (*B*$_{cage}$H). IR ν (film) cm^{-1}: 3309, 2976, 2558, 1700, 1504, 1262, 1010, 760, 696, 575. HRMS (DART) Calcd for $C_{21}H_{32}{}^{10}B_2{}^{11}B_8NO^+$ [M+H$^+$]: 422.3482. Found: 422.3480.

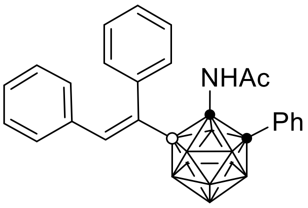

(S)-**3ga**

(S)-**3ga**: White solid. M.p. = 180-182 °C. TLC: R_f = 0.36 (*n*-hexane : ethyl acetate = 6 : 1). Method **A**: Yield: 98%. 93% ee, $[\alpha]_D^{23}$ = -7.5 (*c* = 1.10, CH_2Cl_2). HPLC condition: Chiralpak IA (0.46 x 25 cm, 5 μm), acetonitrile/water = 9:1, 0.7 mL/min, 214 nm UV detector, t_R = 6.61 min (minor) and t_R = 7.63 min (major). ^1H NMR (400 MHz, $CDCl_3$): δ 7.56 (d, *J* = 7.6 Hz, 2H), 7.42 (d, *J* = 7.6 Hz, 1H), 7.33 (m, 4H), 7.23 (t, *J* = 7.6 Hz, 2H), 7.12 (m, 1H), 7.08 (m, 4H), 6.89 (m, 2H) (aromatic & alkenyl C*H*), 5.17 (s, 1H) (N*H*), 1.55 (s, 3H) (COCH_3). $^{13}C\{^1H\}$ NMR (101 MHz, $CDCl_3$): δ 168.0 (*C*O), 142.7, 141.9, 137.2, 131.0, 130.9, 130.4, 129.7, 129.0, 128.9, 128.4, 128.0, 127.4, 126.7 (aromatic *C* & alkenyl *C*), 88.2, 84.5 (cage *C*), 23.6 (CO*C*H_3), the B_{cage}-*C* was not observed. ^{11}B NMR (128 MHz, $CDCl_3$): δ -0.2 (s, 1B) (B_{cage}-C), -3.2 (d, *J* = 97.5 Hz, 1B), -4.7 (d, *J* = 99.3 Hz, 1B), -12.0 (m, 7B) (B_{cage}H). IR ν (film) cm^{-1}: 3384, 2561, 1705, 1475, 1261, 761, 703, 549. HRMS (DART) Calcd for $C_{24}H_{30}{}^{10}B_2{}^{11}B_8NO^+$ [M+H$^+$]: 456.3325. Found: 456.3319.

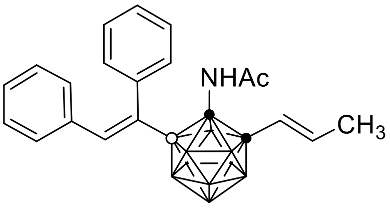

(S)-**3ha**

(S)-**3ha**: White solid. M.p. = 195-196 °C. TLC: R_f = 0.28 (*n*-hexane : ethyl acetate = 4 : 1). Method **A**: Yield: 94%. 98% ee, $[\alpha]_D^{28}$ = -27.5 (*c* = 1.05, CH_2Cl_2). HPLC condition: Chiralpak IE3 (0.46 x 25 cm, 5 μm), acetonitrile/water = 9:1, 0.7 mL/min, 214 nm UV detector, t_R = 10.09 min (minor) and t_R = 10.71 min (major). ^1H NMR (400 MHz, $CDCl_3$): δ 7.32 (m, 2H), 7.25 (m, 1H), 7.10 (d, *J* = 7.2 Hz, 2H), 7.06 (m, 3H) (aromatic C*H*), 7.01 (s, 1H) (PhC=C*H*Ph), 6.87 (m, 2H) (aromatic C*H*), 6.09 (m, 1H) (CH=C*H*CH_3), 5.86 (s, 1H) (N*H*), 5.55 (d, *J* = 15.2 Hz, 1H) (C*H*=CHCH_3), 1.81 (s, 3H) (COCH_3), 1.72 (d, *J* = 6.4 Hz, 3H) (CH=CHCH_3). $^{13}C\{^1H\}$ NMR (101 MHz, $CDCl_3$): δ 167.8 (*C*O), 142.3, 141.9, 139.4, 137.2, 129.7, 129.1, 128.5, 128.0, 127.4, 126.8, 123.1 (aromatic *C* & alkenyl *C*), 83.6, 82.8 (cage *C*), 24.0 (CO*C*H_3), 18.2 (CH=CH*C*H_3), the B_{cage}-*C* was not observed. ^{11}B NMR (128 MHz, $CDCl_3$): δ -0.9 (s, 1B) (B_{cage}-C), -4.3 (d, *J* = 121.6 Hz, 1B), -5.3 (d, *J* = 115.2 Hz, 1B), -11.2 (d, *J* = 143.4 Hz, 2B), -12.2 (d, *J* = 110.1 Hz, 2B), -13.6 (m, 3B) (B_{cage}H). IR ν (film) cm^{-1}: 3205, 3023, 2597, 1689, 1532, 1276, 954, 751, 694, 598. HRMS (DART) Calcd for $C_{21}H_{30}{}^{10}B_2{}^{11}B_8NO^+$ [M+H$^+$]: 420.3325. Found: 420.3326

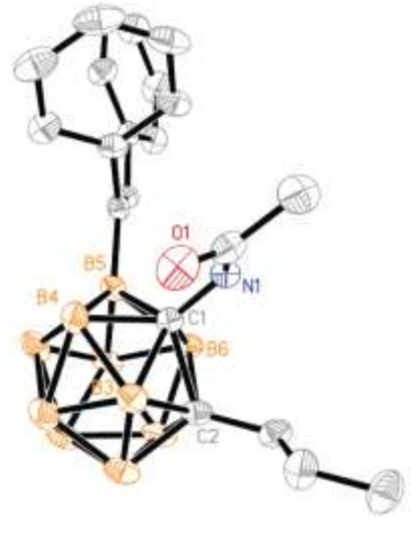

Supplementary Figure 5. Molecular structure of (*S*)-**3ha**

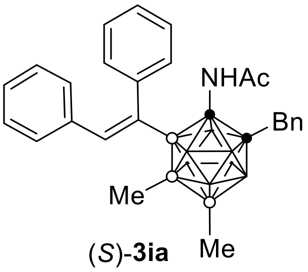

(*S*)-**3ia**: White solid. M.p. = 218-221 °C. TLC: R_f = 0.49 (*n*-hexane : ethyl acetate = 4 : 1). Method **A**: Yield: 60%. 99% ee, $[\alpha]_D^{29}$ = 54.2 (*c* = 0.70, CH_2Cl_2). HPLC condition: Chiralpak IA (0.46 x 25 cm, 5 μm), acetonitrile/water = 9:1, 0.7 mL/min, 214 nm UV detector, t_R= 6.83 min (major) and t_R= 8.07 min (minor). ^{1}H NMR (400 MHz, $CDCl_3$): *δ* 7.31 (m, 5H), 7.27 (m, 1H), 7.10 (m, 7H) (aromatic C*H*), 7.00 (s, 1H) (alkenyl C*H*), 6.90 (m, 2H) (aromatic C*H*), 6.12 (s, 1H) (N*H*), 3.33 (s, 2H) (CH_2), 1.95 (s, 3H) (COCH_3), 0.32 (s, 3H), 0.15 (s, 3H) (B_{cage}CH_3). ^{13}C{^{1}H} NMR (101 MHz, $CDCl_3$): *δ* 168.3 (*C*O), 143.1, 141.9, 137.4, 135.3, 130.3, 129.8, 129.0, 128.7, 128.1, 128.0, 127.3 (aromatic *C* & alkenyl *C*), 77.7 (cage *C*), 39.9 (*C*H_2), 24.2 (CO*C*H_3), the B_{cage}-*C* and B_{cage}-*C*H_3 were not observed. ^{11}B NMR (128 MHz, $CDCl_3$): *δ* 5.4 (s, 1B) 4.6 (s, 1B) (*B*$_{cage}$-CH_3), 2.0 (s, 1B) (*B*$_{cage}$-C), -9.8 (d, *J* = 101.4 Hz, 3B), -11.7 (d, *J* = 116.9 Hz, 2B), -13.8 (d, *J* = 90.5 Hz, 2B) (*B*$_{cage}$H). IR ν (film) cm^{-1}: 3260, 2904, 2550, 1691, 1529, 1271, 746, 701, 537. HRMS (DART) Calcd for $C_{27}H_{36}{}^{10}B_2{}^{11}B_8NO^+$ [M+H$^+$]: 498.3795. Found: 498.3792.

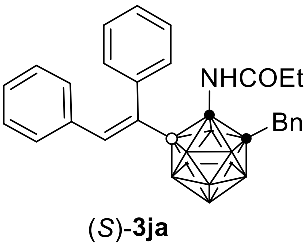

(*S*)-**3ja**: White solid. M.p. = 165-167 °C. TLC: R_f = 0.49 (*n*-hexane : ethyl acetate = 4 : 1). Method **A**: Yield: 99%. 96% ee, $[\alpha]_D^{28}$ = -19.8 (*c* = 1.00, CH_2Cl_2). HPLC condition: Chiralpak IA (0.46 x 25 cm, 5 μm), acetonitrile/water = 95:5, 0.7 mL/min, 214 nm UV detector, t_R = 9.31 min (major) and t_R = 10.84 min (minor). ^{1}H NMR (400 MHz, $CDCl_3$): *δ* 7.32 (m,

5H), 7.27 (m, 1H), 7.13 (d, *J* = 7.2 Hz, 2H), 7.09 (m, 5H) (aromatic C*H*), 7.04 (s, 1H) (alkenyl C*H*), 6.90 (m, 2H) (aromatic C*H*), 6.06 (s, 1H) (N*H*), 3.31 (s, 2H) (C_{cage}-CH_2), 2.15 (m, 2H) (COCH_2), 1.12 (t, *J* = 7.6 Hz, 3H) (CH_3). ^{13}C{^{1}H} NMR (101 MHz, $CDCl_3$): δ 171.9 (*C*O), 142.8, 142.0, 137.1, 134.9, 130.3, 129.7, 129.1, 128.7, 128.5, 128.1, 128.0, 127.4, 126.8 (aromatic *C* & alkenyl *C*), 84.0, 83.0 (cage *C*), 40.3 (C_{cage}-*C*H_2), 30.2 (CO*C*H_2), 9.1 (*C*H_3), the B_{cage}-*C* was not observed. ^{11}B NMR (128 MHz, $CDCl_3$): δ -1.3 (s, 1B) (*B*$_{cage}$-C), -4.5 (d, *J* = 124.2 Hz, 1B), -5.8 (d, *J* = 89.6 Hz, 1B), -11.4 (d, *J* = 83.2 Hz, 4B), -12.5 (d, *J* = 137.0 Hz, 1B), 13.9 (d, *J* = 149.8 Hz, 2B) (*B*$_{cage}$H). IR ν (film) cm^{-1}: 3270, 2569, 1693, 1507, 1073, 754, 695, 534. HRMS (DART) Calcd for $C_{26}H_{34}{}^{10}B_2{}^{11}B_8NO^+$ [M+H$^+$]: 484.3638. Found: 484.3637.

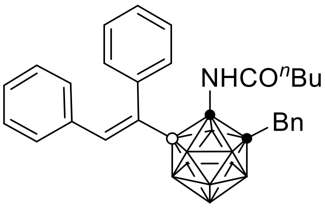

(*S*)-**3ka**

(*S*)-**3ka**: White solid. M.p. = 165-167 °C. TLC: R_f = 0.48 (*n*-hexane : ethyl acetate = 4 : 1). Method **A**: Yield: 98%. 94% ee, $[\alpha]_D^{25}$ = -26.2 (*c* = 1.59, CH_2Cl_2). HPLC condition: Chiralpak IG (0.46 x 25 cm, 5 μm), acetonitrile/water = 97:3, 0.7 mL/min, 214 nm UV detector, t_R = 13.08 min (major) and t_R = 14.99 min (minor). ^{1}H NMR (400 MHz, $CDCl_3$): δ 7.32 (m, 6H), 7.14 (d, *J* = 7.2 Hz, 2H), 7.09 (m, 5H) (aromatic C*H*), 7.04 (s, 1H) (alkenyl C*H*), 6.90 (m, 2H) (aromatic C*H*), 6.10 (s, 1H) (N*H*), 3.30 (s, 2H) (C_{cage}-CH_2), 2.12 (t, *J* = 7.6 Hz, 2H) (COCH_2), 1.59 (m, 2H), 1.31 (m, 2H) (other CH_2), 0.88 (t, *J* = 7.2 Hz, 3H) (CH_3). ^{13}C{^{1}H} NMR (101 MHz, $CDCl_3$): δ 171.2 (*C*O), 142.9, 142.0, 137.2, 134.9, 130.3, 129.7, 129.1, 128.7, 128.5, 128.1, 128.0, 127.4, 126.8 (aromatic *C* & alkenyl *C*), 83.8, 83.2 (cage *C*), 40.3 (C_{cage}-*C*H_2), 36.9 (CO*C*H_2), 27.1, 22.4 (*C*H_2), 13.8 (*C*H_3), the B_{cage}-*C* was not observed. ^{11}B NMR (128 MHz, $CDCl_3$): δ -1.2 (s, 1B) (*B*$_{cage}$-C), -4.4 (d, *J* = 113.2 Hz, 1B), -5.8 (d, *J* = 103.2 Hz, 1B), -11.5 (d, *J* = 106.6 Hz, 4B), -12.5 (d, *J* = 123.6 Hz, 1B), -13.9 (d, *J* = 123.4 Hz, 2B) (*B*$_{cage}$H). IR ν (film) cm^{-1}: 2958, 2565, 1693, 1495, 1260, 753, 698, 535. HRMS (DART) Calcd for $C_{28}H_{38}{}^{10}B_2{}^{11}B_8NO^+$ [M+H$^+$]: 512.3951. Found: 512.3948.

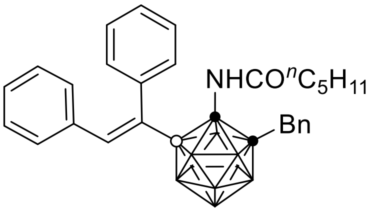

(S)-**3la**

(S)-**3la**: White solid. M.p. = 131.0- 133 °C. TLC: R_f = 0.49 (n-hexane : ethyl acetate = 6 : 1). Method **A**: Yield: 92%. 94% ee, $[\alpha]_D^{29}$ = -22.9 (c = 0.48, CH_2Cl_2). HPLC condition: Chiralpak IA (0.46 x 25 cm, 5 μm), acetonitrile/water = 99:1, 0.7 mL/min, 214 nm UV detector, t_R = 13.24 min (major) and t_R = 15.36 min (minor). ^{1}H NMR (400 MHz, $CDCl_3$): δ 7.32 (m, 5H), 7.28 (m, 1H), 7.14 (d, J = 7.2 Hz, 2H), 7.09 (m, 5H) (aromatic C*H*), 7,04 (s, 1H) (alkenyl C*H*), 6.90 (m, 2H) (aromatic C*H*), 6.08 (s, 1H) (N*H*), 3.30 (s, 2H) (C_{cage}-CH_2), 2.12 (t, J = 7.6 Hz, 2H) (COCH_2), 1.59 (m, 2H), 1.27 (m, 4H) (CH_2), 0.85 (t, J = 6.8 Hz, 3H) (CH_3). ^{13}C{^{1}H} NMR (101 MHz, $CDCl_3$): δ 171.2 (*C*O), 142.9, 142.1, 137.2, 134.9, 130.3, 129.8, 128.7, 128.6, 128.2, 128.1, 127.4, 126.8 (aromatic *C* & alkenyl *C*), 83.8, 83.2 (cage *C*), 40.2 (C_{cage}-*C*H_2), 37.1, 31.4, 24.7, 22.5 (*C*H_2), 14.0 (*C*H_3), the B_{cage}-*C* was not observed. ^{11}B NMR (128 MHz, $CDCl_3$): δ -1.1 (s, 1B) (*B*$_{cage}$-C), -4.5 (d, J = 113.9 Hz, 1B), -5.5 (d, J = 115.2 Hz, 1B), -11.5 (d, J = 107.5 Hz, 3B), -12.6 (d, J = 115.2 Hz, 2B), 14.0 (d, J = 165.1 Hz, 2B) (*B*$_{cage}$H). IR ν (film) cm^{-1}: 3307, 2956, 2560, 1693, 1456, 1256, 753, 698, 535. HRMS (DART) Calcd for $C_{29}H_{40}{}^{10}B_2{}^{11}B_8NO^+$ [M+H$^+$]: 526.4108. Found: 526.4099.

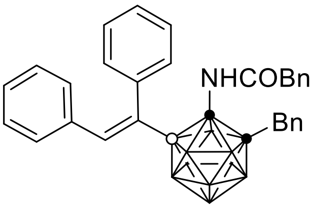

(S)-**3ma**

(S)-**3ma**: White solid. M.p. = 118-120 °C. TLC: R_f = 0.27 (n-hexane : ethyl acetate = 8 : 1). Method **A**: Yield: 95%. 93% ee, $[\alpha]_D^{26}$ = -24.2 (c = 0.79, CH_2Cl_2). HPLC condition: Chiralpak IA (0.46 x 25 cm, 5 μm), acetonitrile/water = 99:1, 0.7 mL/min, 214 nm UV detector, t_R= 25.71 min (major) and t_R= 29.71 min (minor). ^{1}H NMR (400 MHz, $CDCl_3$): δ 7.35 (m, 2H), 7.27 (m, 5H), 7.25 (m, 2H), 7.16 (m, 2H), 7.12 (m, 3H), 7.00 (d, J = 6.8 Hz, 2H) (aromatic C*H*), 6.96 (s, 1H) (alkenyl C*H*), 6.89 (m, 2H), 6.85 (m, 2H) (aromatic C*H*), 6.19 (s, 1H) (N*H*), 3.63 (d, J = 15.6 Hz, 1H), 3.47 (d, J = 15.6 Hz, 1H), 3.13 (d, J = 15.2 Hz, 1H), 3.01 (d, J = 15.2 Hz, 1H) (CH_2). ^{13}C{^{1}H} NMR (101 MHz, $CDCl_3$): δ 169.1 (*C*O), 143.0, 141.5, 137.2, 134.8, 133.3, 130.3, 129.8, 129.3, 129.0, 128.6, 128.4, 128.3, 128.0, 127.5, 126.7 (aromatic *C* & alkenyl *C*), 84.5, 83.2 (cage *C*), 44.6, 40.0 (*C*H_2), the B_{cage}-*C* was not observed. ^{11}B NMR (128 MHz, $CDCl_3$): δ -1.2 (s, 1B) (*B*$_{cage}$-C), -4.2 (d, J = 89.6 Hz, 1B), -5.8 (d, J = 106.2 Hz, 1B), -11.4 (d, J = 91.5 Hz, 4B), -12.1 (d, J = 96.4 Hz, 1B), -13.9 (d, J = 82.9 Hz, 2B) (*B*$_{cage}$H). IR ν (film) cm^{-1}: 3024, 2924, 2567, 1692, 1445, 1212, 1116, 724, 693, 537. HRMS (DART) Calcd for $C_{31}H_{36}{}^{10}B_2{}^{11}B_8NO^+$ [M+H$^+$]: 546.3795. Found: 546.3789.

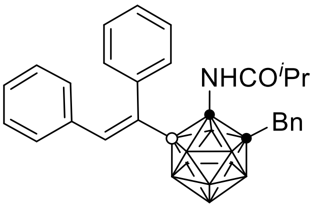

(S)-**3na**

(S)-**3na**: White solid. M.p. = 196-198 °C. TLC: R_f = 0.34 (n-hexane : ethyl acetate = 8 : 1). Method **A**: Yield: 93%. 87% ee, $[\alpha]_D^{29}$ = -33.2 (c = 0.90, CH_2Cl_2). HPLC condition: Chiralpak IA (0.46 x 25 cm, 5 μm), acetonitrile/water = 99:1, 0.7 mL/min, 214 nm UV detector, t_R = 12.60 min (major) and t_R = 14.93 min (minor). ^{1}H NMR (400 MHz, $CDCl_3$): δ 7.31 (m, 5H), 7.27 (m, 1H), 7.14 (d, J = 7.2 Hz, 2H), 7.09 (m, 5H) (aromatic CH), 7.04 (s, 1H) (alkenyl CH), 6.90 (m, 2H) (aromatic CH), 6.19 (s, 1H) (NH), 3.30 (s, 2H) (C_{cage}-CH_2), 2.22 (m, 1H) (CH), 1.17 (d, J = 6.8 Hz, 3H), 1.14 (d, J = 6.8 Hz, 3H) (CH_3). ^{13}C{^{1}H} NMR (101 MHz, $CDCl_3$): δ 175.3 (CO), 142.9, 141.8, 137.2, 134.9, 130.4, 129.7, 129.1, 128.7, 128.5, 128.1, 128.0, 127.4, 126.8 (aromatic C & alkenyl C), 84.1, 83.3 (cage C), 40.2 (CH$_2$), 36.5 (CH), 20.3, 18.9 (CH$_3$), the B_{cage}-C was not observed. ^{11}B NMR (128 MHz, $CDCl_3$): δ -0.9 (s, 1B) (B_{cage}-C), -4.3 (d, J = 107.5 Hz, 1B), -5.6 (d, J = 98.6 Hz, 1B), -11.3 (d, J = 84.5 Hz, 4B), -12.4 (d, J = 121.6 Hz, 1B), 13.8 (d, J = 180.5 Hz, 2B) (B_{cage}H). IR ν (film) cm^{-1}: 2929, 2569, 1700, 1492, 1253, 754, 696, 535. HRMS (DART) Calcd for $C_{27}H_{36}{}^{10}B_2{}^{11}B_8NO^+$ [M+H$^+$]: 498.3795. Found: 498.3790.

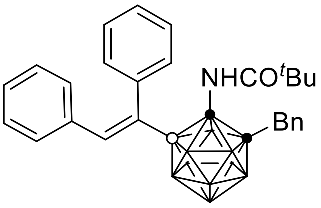

(S)-**3oa**

(S)-**3oa**: White solid. M.p. = 211-213 °C. TLC: R_f = 0.34 (n-hexane : ethyl acetate = 4 : 1). Method **A**: Yield: 70%. 80% ee, $[\alpha]_D^{27}$ = -46.5 (c = 1.48, CH_2Cl_2). HPLC condition: Chiralpak ODH (0.46 x 25 cm, 5 μm), acetonitrile/water = 98:2, 0.7 mL/min, 214 nm UV detector, t_R = 33.51 min (minor) and t_R = 38.19 min (major). ^{1}H NMR (400 MHz, $CDCl_3$): δ 7.33 (m, 5H), 7.27 (m, 1H), 7.11 (m, 7H) (aromatic CH), 7.02 (s, 1H) (alkenyl CH), 6.91 (m, 2H) (aromatic CH), 6.60 (s, 1H) (NH), 3.33 (s, 2H) (C_{cage}-CH_2), 1.24 (s, 9H) (C(CH_3)$_3$). ^{13}C{^{1}H} NMR (101 MHz, $CDCl_3$): δ 176.6 (CO), 143.0, 141.1, 137.2, 134.9, 130.4, 129.7, 128.9, 128.8, 128.6, 128.2, 128.1, 127.4, 126.7 (aromatic C & alkenyl C), 84.4, 83.8 (cage C), 40.3 (C_{cage}-CH$_2$), 29.8 (C(CH$_3$)$_3$), 27.7 (C(CH$_3$)$_3$). ^{11}B NMR (128 MHz, $CDCl_3$): δ -1.0 (s, 1B) (B_{cage}-C), -4.2 (d, J = 97.3 Hz, 1B), -5.7 (d, J = 92.2 Hz, 1B), -11.4 (d, J = 85.8 Hz, 3B), -12.2 (d, J = 105.0 Hz, 2B), -13.5 (d, J = 162.6 Hz, 2B) (B_{cage}H). IR ν (film) cm^{-1}: 3253, 2966, 2582, 1688, 1528, 1264, 1074, 748, 692, 537. HRMS (DART) Calcd for $C_{28}H_{38}{}^{10}B_2{}^{11}B_8NO^+$ [M+H$^+$]: 512.3951. Found: 512.3947.

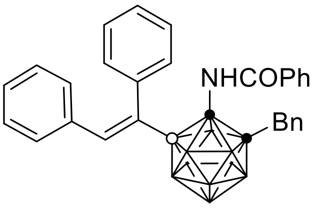

(*S*)-**3pa**

(*S*)-**3pa**: White solid. M.p. = 199-201 °C. TLC: R_f = 0.40 (*n*-hexane : ethyl acetate = 6 : 1). Method **A**: Yield: 70%. 90% ee, $[\alpha]_D^{29}$ = -34.1 (*c* = 0.50, CH_2Cl_2). HPLC condition: Chiralpak IA (0.46 x 25 cm, 5 μm), acetonitrile/water = 99:1, 0.7 mL/min, 214 nm UV detector, t_R = 23.01 min (minor) and t_R = 25.87 min (major). ^{1}H NMR (400 MHz, $CDCl_3$): δ 7.64 (m, 2H), 7.60 (t, *J* = 7.2 Hz, 1H), 7.46 (t, *J* = 7.6 Hz, 2H), 7.29 (m, 3H), 7.16 (m, 3H), 7.09 (m, 2H), 7.04 (m, 4H), 6.98 (m, 2H), 6.84 (m, 3H) (aromatic C*H*, alkenyl C*H* & N*H*), 3.41 (s, 2H) (C_{cage}-CH_2). ^{13}C{^{1}H} NMR (101 MHz, $CDCl_3$): δ 165.2 (*C*O), 142.7, 142.0, 137.1, 134.9, 133.4, 132.3, 130.4, 129.7, 129.2, 129.1, 128.7, 128.5, 128.2, 128.0, 127.4, 126.7 (aromatic *C* & alkenyl *C*), 84.2, 83.2 (cage *C*), 40.6 (*C*H_2), the B_{cage}-*C* was not observed. ^{11}B NMR (128 MHz, $CDCl_3$): δ -0.9 (s, 1B) (*B*$_{cage}$-C), -4.3 (d, *J* = 100.7 Hz, 1B), -5.3 (d, *J* = 108.3 Hz, 1B), -11.3 (d, *J* = 102.5 Hz, 4B), -12.4 (d, *J* = 113.5 Hz, 1B), -13.7 (d, *J* = 149.0 Hz, 2B) (*B*$_{cage}$H). IR ν (film) cm^{-1}: 3026, 2571, 1477, 1192, 753, 695, 546. HRMS (DART) Calcd for $C_{30}H_{34}{}^{10}B_2{}^{11}B_8NO^+$ [M+H$^+$]: 532.3638. Found: 532.3632.

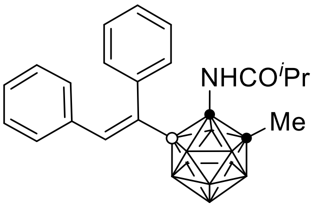

(*S*)-**3qa**

(*S*)-**3qa**: White solid. M.p. = 164-166 °C. TLC: R_f = 0.48 (*n*-hexane : ethyl acetate = 4 : 1). Method **A**: Yield: 99%. 79% ee, $[\alpha]_D^{30}$ = 3.7 (*c* = 1.26, CH_2Cl_2). HPLC condition: Chiralpak ADH (0.46 x 25 cm, 5 μm), acetonitrile/water = 99:1, 0.7 mL/min, 214 nm UV detector, t_R = 10.15 min (major) and t_R = 11.97 min (minor). ^{1}H NMR (400 MHz, $CDCl_3$): δ 7.34 (t, *J* = 7.2 Hz, 2H), 7.28 (m, 1H), 7.12 (d, *J* = 7.6 Hz, 2H), 7.08 (m, 3H) (aromatic C*H*), 7.03 (s, 1H) (alkenyl C*H*), 6.89 (m, 2H) (aromatic C*H*), 6.01 (s, 1H) (N*H*), 2.15 (m, 1H) (C*H*(CH_3)$_2$), 1.89 (s, 3H) (C_{cage}-CH_3), 1.09 (d, *J* = 6.8 Hz, 6H) (CH(CH_3)$_2$). ^{13}C{^{1}H} NMR (101 MHz, $CDCl_3$): δ 175.2 (*C*O), 143.0, 141.7, 137.1, 130.0, 129.0, 128.4, 128.0, 127.4, 126.7 (aromatic *C* & alkenyl *C*), 81.5, 79.1 (cage *C*), 36.4 (*C*H), 22.3, 20.2, 18.8 (*C*H_3), the B_{cage}-*C* was not observed. ^{11}B NMR (128 MHz, $CDCl_3$): δ -1.0 (s, 1B) (*B*$_{cage}$-C), -5.4 (d, *J* = 87.0 Hz, 2B), -10.6 (d, *J* = 96.0 Hz, 3B), -11.7 (d, *J* = 139.5 Hz, 1B), -12.7 (d, *J* = 90.9 Hz, 3B) (*B*$_{cage}$H). IR ν (film) cm^{-1}: 3337, 2973, 2588, 1700, 1496, 1259, 1101, 752, 692, 571. HRMS (DART) Calcd for $C_{21}H_{32}{}^{10}B_2{}^{11}B_8NO^+$ [M+H$^+$]: 422.3482. Found: 422.3481.

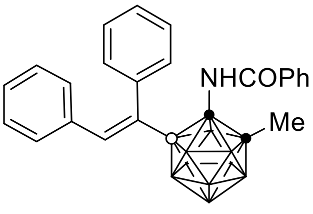

(*S*)-**3ra**

(*S*)-**3ra**: White solid. M.p. = 200-202 °C. TLC: R_f = 0.34 (*n*-hexane : ethyl acetate = 8 : 1). Method **A**: Yield: 88%. 85% ee, $[\alpha]_D^{31}$ = -32.3 (*c* = 1.10, CH_2Cl_2). HPLC condition: Chiralpak IB (0.46 x 25 cm, 5 μm), acetonitrile/water = 9:1, 0.7 mL/min, 214 nm UV detector, t_R = 8.33 min (major) and t_R = 9.50 min (minor). ^{1}H NMR (400 MHz, $CDCl_3$): δ 7.59 (m, 3H), 7.43 (t, *J* = 7.6 Hz, 2H), 7.15 (m, 3H), 7.03 (m, 6H), 6.85 (m, 2H) (aromatic C*H* & alkenyl C*H*), 6.69 (s, 1H) (N*H*), 1.97 (s, 3H) (C*H*$_3$). ^{13}C{^{1}H} NMR (101 MHz, $CDCl_3$): δ 165.2 (*C*O), 142.8, 141.9, 137.1, 133.2, 132.3, 129.1, 128.4, 128.0, 127.4, 127.3, 126.7 (aromatic *C* & alkenyl *C*), 81.7, 79.3 (cage *C*), 22.5 (*C*H$_3$), the B_{cage}-*C* was not observed. ^{11}B NMR (128 MHz, $CDCl_3$): δ -0.8 (s, 1B) (B_{cage}-C), -5.3 (d, *J* = 96.0 Hz, 2B), -10.5 (d, *J* = 183.0 Hz, 1B), -11.4 (d, *J* = 126.7 Hz, 2B), -12.6 (d, *J* = 143.4 Hz, 4B) (B_{cage}H). IR ν (film) cm^{-1}: 2573, 1677, 1518, 1276, 1025, 752, 690. HRMS (DART) Calcd for $C_{24}H_{30}{}^{10}B_2{}^{11}B_8NO^+$ [M+H$^+$]: 456.3325. Found: 456.3325.

Large-scale synthesis of (*S*)-3ba.

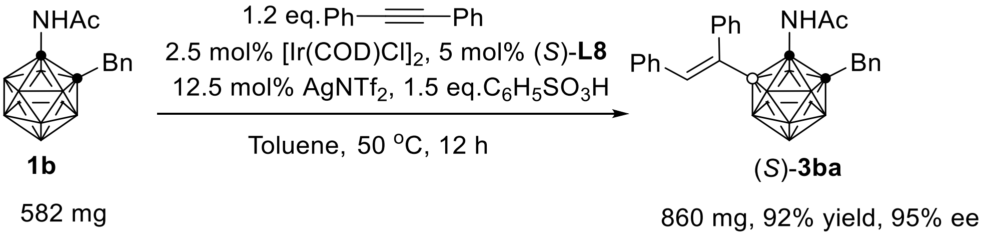

An oven-dried Schlenk flask equipped with a stir bar was charged with $[Ir(COD)Cl]_2$ (34 mg, 0.05 mmol) and (*S*)-**L8** (52 mg, 0.1 mmol), followed by dry toluene (10 mL). The mixture was stirred at room temperature for 1 h, to which was successively added **1b** (582 mg, 2.0 mmol), diphenyl acetylene (428 mg, 2.4 mmol), benzenesulfonic acid (474 mg, 0.3 mmol), $AgNTf_2$ (97 mg, 0.25 mmol) and dry toluene (10 mL). The flask was closed under an atmosphere of nitrogen, then stirred at 50 °C for 12 h. After hydrolysis with water (30 mL) and extraction with diethyl ether (30 mL x 3), the ether solutions were combined, dried over anhydrous Na_2SO_4 and concentrated to dryness in vacuo. The residue was subjected to flash column chromatography on silica gel (230-400 mesh) using *n*-hexane and ethyl acetate (4/1 in v/v) as eluent to give product (*S*)-**3ba** (860 mg, 92% yield, 95% ee).

Deacylation of (*S*)-3ba.

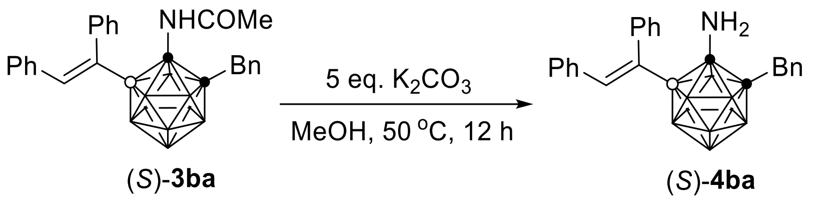

A MeOH (2 mL) suspension of (*S*)-**3ba** (47 mg, 0.1 mmol) and K_2CO_3 (69 mg, 0.5 mmol) was stirred at 50 °C for 12 h. After hydrolysis with water (5 mL) and extraction with diethyl ether (10 mL x 3), the ether solutions were combined, dried over anhydrous Na_2SO_4 and concentrated to dryness in vacuo. The residue was subjected to flash column chromatography on silica gel (230-400 mesh) using *n*-hexane and ethyl acetate (10/1 in v/v) as eluent to give (*S*)-**4ba** (38 mg, 90%).

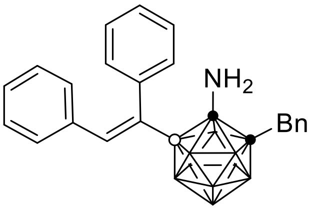

(*S*)-**4ba**

(*S*)-**4ba**: White solid. M.p. = 178-180 °C. TLC: R_f = 0.49 (*n*-hexane : ethyl acetate = 8 : 1). Yield: 90%. 97% ee, $[\alpha]_D^{28}$ = -50.6 (*c* = 1.20, CH_2Cl_2). HPLC condition: Chiralpak IG (0.46 x 25 cm, 5 μm), acetonitrile/water = 99:1, 0.7 mL/min, 214 nm UV detector, t_R = 8.41 min (major) and t_R = 8.96 min (minor). ^{1}H NMR (400 MHz, $CDCl_3$): δ 7.33 (m, 6H), 7.17 (m, 4H), 7.10 (m, 3H) (aromatic C*H*), 7.07 (s, 1H) (alkenyl C*H*), 6.94 (m, 2H) (aromatic C*H*), 3.59 (d, *J* = 14.8 Hz, 1H), 3.53 (d, *J* = 14.8 Hz, 1H) (CH_2), 2.97 (s, 2H) (NH_2) (the NH_2 signal peak was proved by H-D exchange experiment). $^{13}C\{^1H\}$ NMR (101 MHz, $CDCl_3$): δ 143.2, 141.2, 137.3, 136.2, 130.6, 129.7, 129.0, 128.5, 128.4, 128.0, 127.7, 127.4, 126.6 (aromatic *C* & alkenyl *C*), 92.9, 85.3 (cage *C*), 38.9 (CH_2), the B_{cage}-*C* was not observed. ^{11}B NMR (128 MHz, $CDCl_3$): δ -1.3 (s, 1B) (B_{cage}-C), -3.6 (d, *J* = 101.8 Hz, 1B), -8.9 (d, *J* = 104.7 Hz, 1B), -10.0 (d, *J* = 140.3 Hz, 1B), -11.4 (d, *J* = 96.1 Hz, 3B), -13.0 (m, 1B), -13.5 (d, *J* = 97.3 Hz, 2B) (B_{cage}H). IR ν (film) cm^{-1}: 3428, 3346, 2963, 2565, 1604, 1492, 1259, 1070, 1014, 752, 698, 553. HRMS (DART) Calcd for $C_{23}H_{30}{}^{10}B_2{}^{11}B_8NO^+$ $[M+H^+]$: 428.3376. Found: 428.3373.

Mechanistic study.

Deuterium labelling experiment.

Synthesis of deuterated *o*-carborane-d_8.[3] An oven-dried Schlenk flask equipped with a stir bar was charged with *o*-$C_2B_{10}H_{12}$ (1.44 g, 10.0 mmol) and aluminum chloride (665 mg, 5.0 mmol), followed by dry benezene-d_6 (5 mL). The flask was closed under an atmosphere of nitrogen, then stirred at 80 °C for 2 weeks. After cooling to room temperature, hydrolysis with water (5 mL) and extraction with

diethyl ether (10 mL x 3), the ether solutions were combined, dried over anhydrous Na_2SO_4 and concentrated to dryness in vacuo. The residue was subjected to flash column chromatography on silica gel (230-400 mesh) using *n*-hexane as eluent to give the product *o*-carborane-d_8 (1.06 g, 70%).

o-Carborane-d_8: $^1H\{^{11}B\}$ NMR ($CDCl_3$, 500 MHz): δ 3.54 (s, 2H) (cage C*H*), 2.31 (s, 1.54H) (B(3,6) *H*), 2.29 (s, 0.16H), 2.19 (s, 0.16H) (B(4,5,7,11) *H*), 2.11 (s, 0.32H) (B(8,10,9,12) *H*). $^2H\{^{11}B\}$ NMR ($CDCl_3$, 77 MHz): 2.29 (s, 2.30D), 2.20 (s, 1.84D) (B(3,6,4,5,7,11) *D*), 2.11 (s, 3.68D) ((B(8,10,9,12) *D*)).

Synthesis of 1b-*d*$_8$. 1-Acetylamino-2-benzyl-4,5,7,8,9,10,11,12-d_8-*o*-$C_2B_{10}H_2$ (**1b**-d_8) with ca. 92% deuteration on B(4,5,7,8,9,10,11,12) vertexes was prepared in 80% yield from *o*-carborane-d_8 using the same procedures for the preparation of **1b**.

1b-d_8: $^1H\{^{11}B\}$ NMR ($CDCl_3$, 500 MHz): δ 7.30 (m, 3H), 7.13 (m, 2H) (aromatic C*H*), 6.74 (m, 1H) (N*H*), 3.41 (s, 2H) (C_{cage}-CH_2), 2.52 (s, 1.54H) (B(3,6) *H*), 2.47 (s, 0.16H), 2.20 (s, 0.08H), 2.17 (s, 3.08H) (CH_3 & B(4,5,7,11) *H*), 2.06 (s, 0.16H) (B(8,10) *H*), 2.01 (s, 0.16H) (B(9,12) *H*). $^2H\{^{11}B\}$ NMR ($CDCl_3$, 77 MHz): δ 2.49 (s, 2.30D), 2.22 (s, 0.92D) (B(3,6,4,5,7,11) *D*), 2.16 (s, 0.92D), 2.08 (s, 1.84D) (B(8,10) *D*), 2.02 (s, 1.84D) (B(9,12) *D*).

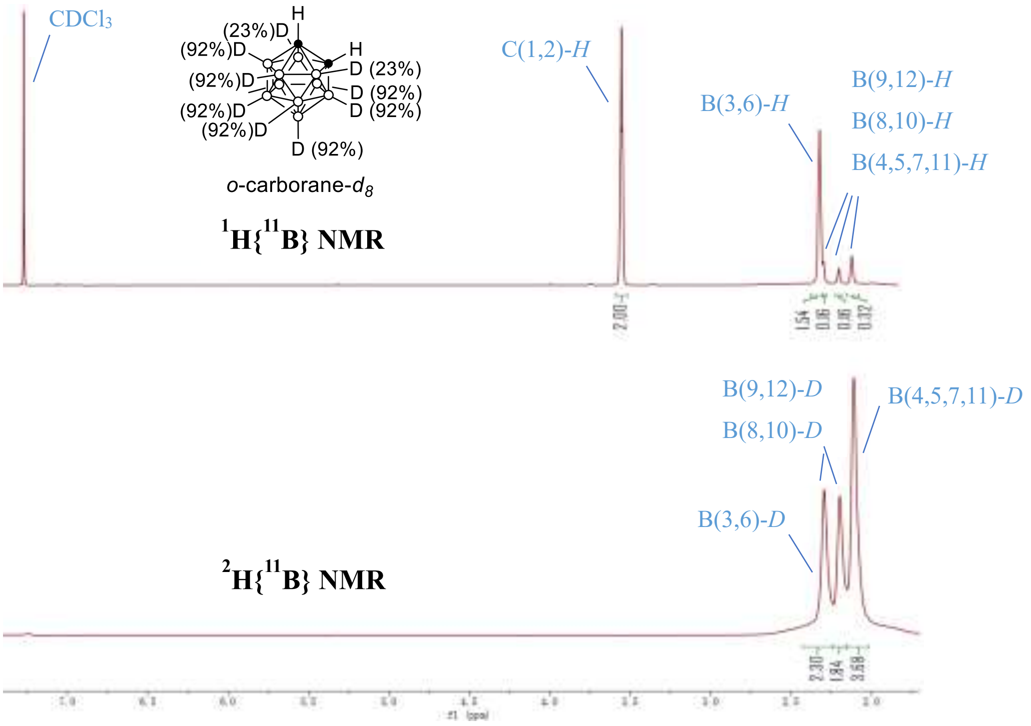

Supplementary Figure 6. Comparison of $^1H\{^{11}B\}$ (up) with $^2H\{^{11}B\}$ (bottom) NMR spectra of *o*-carborane-d_8.

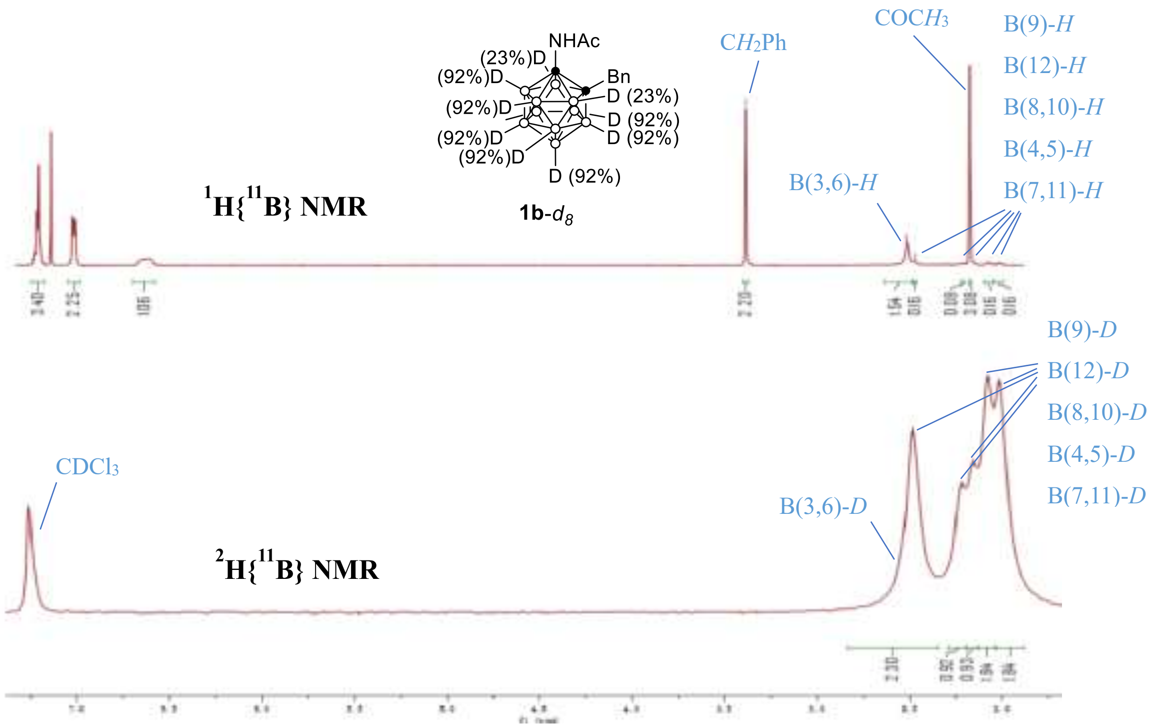

Supplementary Figure 7. Comparison of $^1H\{^{11}B\}$ (up) with $^2H\{^{11}B\}$ (bottom) NMR spectra of **1b**-d_8.

Control experiments.

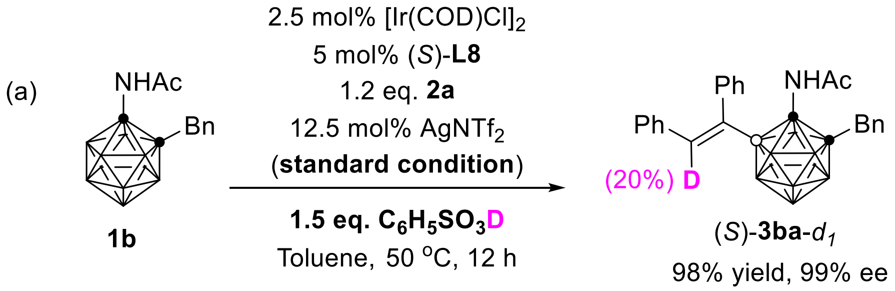

An oven-dried Schlenk flask equipped with a stir bar was charged with $[Ir(COD)Cl]_2$ (1.7 mg, 0. 0025 mmol) and (*S*)-**L8** (2.6 mg, 0.005 mmol), followed by dry toluene (1 mL). The mixture was stirred at room temperature for 1 h, to which was added successively **1b** (29.1 mg, 0.10 mmol), **2a** (21.4 mg, 0.12 mmol), $C_6H_5SO_3D$ (24 mg, 0.15 mmol), $AgNTf_2$ (4.9 mg, 0.0125 mmol) and dry toluene (1 mL). The flask was closed under an atmosphere of nitrogen, then stirred at 50 °C for 12 h. After hydrolysis with water (5 mL) and extraction with diethyl ether (10 mL x 3), the ether solutions were combined, dried over anhydrous Na_2SO_4 and concentrated to dryness in vacuo. The residue was subjected to flash

column chromatography on silica gel (230-400 mesh) using *n*-hexane and ethyl acetate (4/1 in v/v) as eluent to give (*S*)-**3ba**-d_1 (46 mg, 98%).

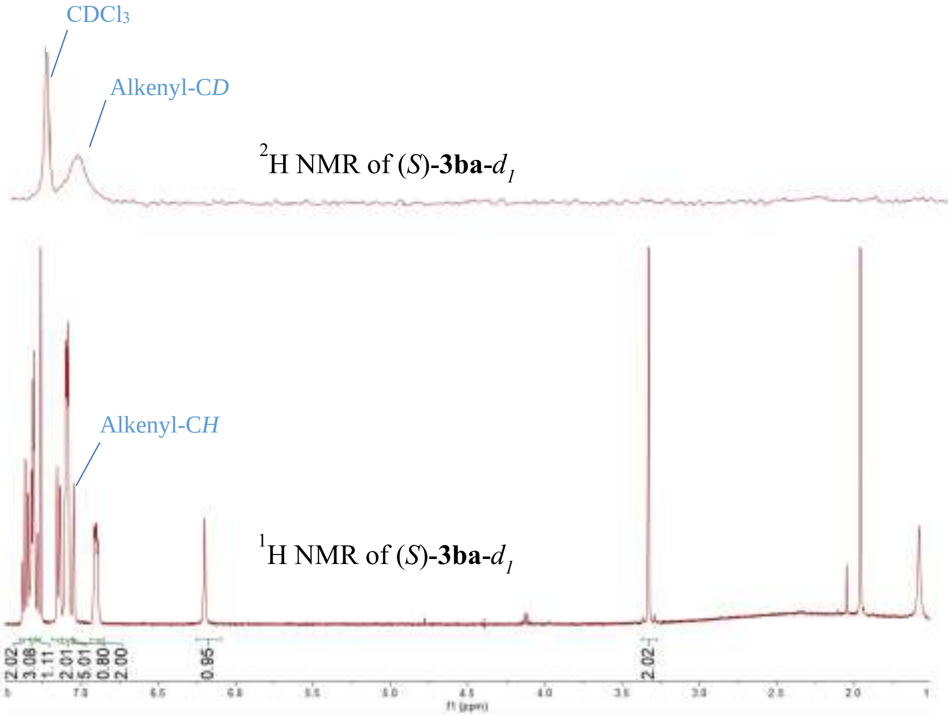

Supplementary Figure 8. Comparison of ^{2}H (up) with ^{1}H (bottom) NMR spectra of (*S*)-**3ba**-d_1.

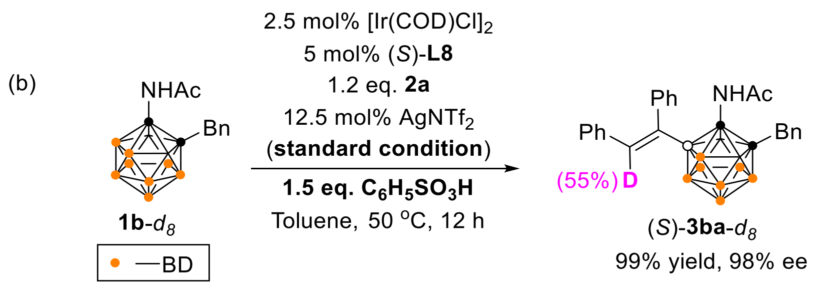

An oven-dried Schlenk flask equipped with a stir bar was charged with $[Ir(COD)Cl]_2$ (1.7 mg, 0.0025 mmol) and (*S*)-**L8** (2.6 mg, 0.005 mmol), followed by dry toluene (1 mL). The mixture was stirred at room temperature for 1 h, to which was added successively **1b**-d_8 (29.1 mg, 0.10 mmol), **2a** (21.4 mg, 0.12 mmol), $C_6H_5SO_3H$ (24 mg, 0.15 mmol), $AgNTf_2$ (4.9 mg, 0.0125 mmol) and dry toluene (1 mL). The flask was closed under an atmosphere of nitrogen, then stirred at 50 °C for 12 h. After hydrolysis with water (5 mL) and extraction with diethyl ether (10 mL x 3), the ether solutions were combined, dried over anhydrous Na_2SO_4 and concentrated to dryness in vacuo. The residue was subjected to flash column chromatography on silica gel (230-400 mesh) using *n*-hexane and ethyl acetate (4/1 in v/v) as eluent to give (*S*)-**3ba**-d_8 (47 mg, 99%).

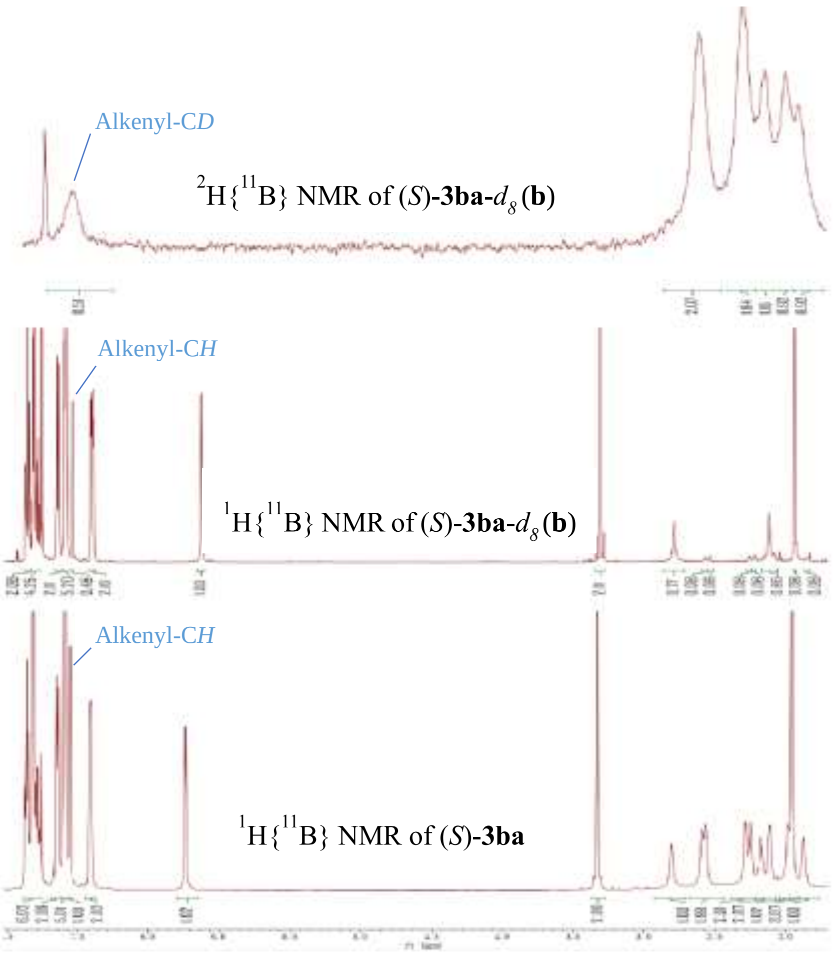

Supplementary Figure 9. Comparison of ^{2}H{^{11}B} with ^{1}H{^{11}B} NMR spectra of (*S*)-**3ba**-*d*$_8$ (**b**) and (*S*)-**3ba**.

(c)

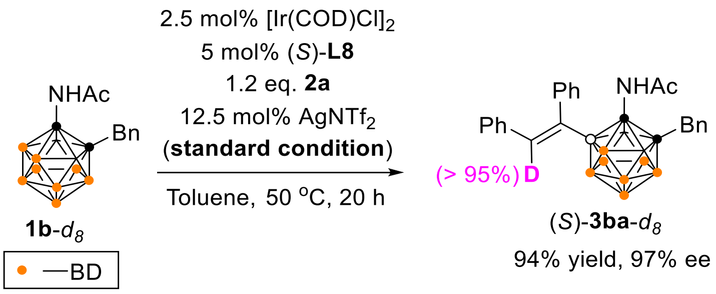

An oven-dried Schlenk flask equipped with a stir bar was charged with $[Ir(COD)Cl]_2$ (1.7 mg, 0.0025 mmol mmol) and (*S*)-**L8** (2.6 mg, 0.005 mmol), followed by dry toluene (1 mL). The mixture was stirred at room temperature for 1 h, to which was added successively **1b**-*d*$_8$ (29.1 mg, 0.1 mmol), **2a** (21.4 mg, 0.12 mmol), $AgNTf_2$ (4.9 mg, 0.0125 mmol) and dry toluene (1 mL). The flask was closed under an atmosphere of nitrogen, then stirred at 50 °C for 20 h. After hydrolysis with water (5 mL) and

extraction with diethyl ether (10 mL x 3), the ether solutions were combined, dried over anhydrous Na_2SO_4 and concentrated to dryness in vacuo. The residue was subjected to flash column chromatography on silica gel (230-400 mesh) using *n*-hexane and ethyl acetate (4/1 in v/v) as eluent to give (*S*)-**3ba**-d_8 (45 mg, 94%).

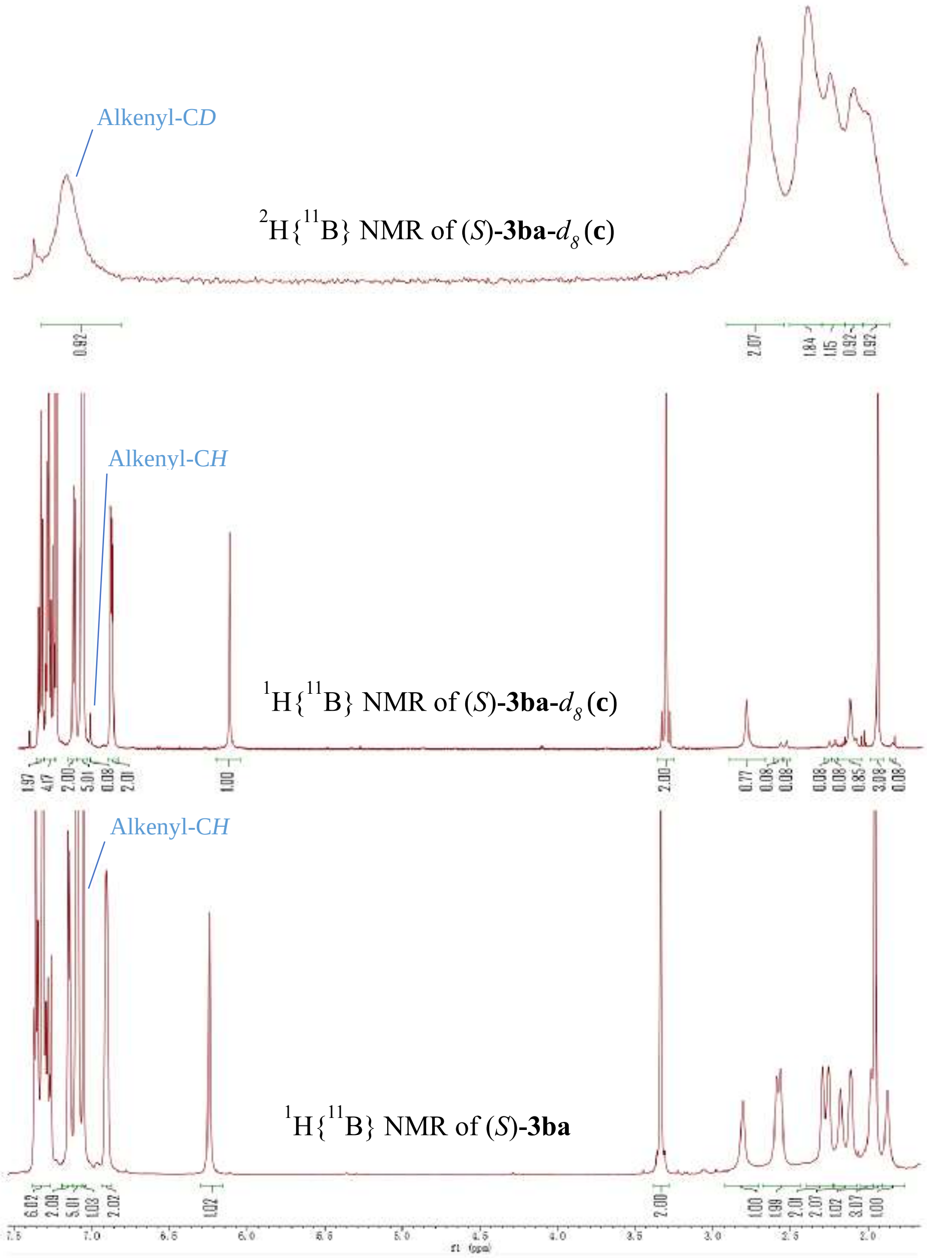

Supplementary Figure 10. Comparison of ^{2}H{^{11}B} with ^{1}H{^{11}B} NMR spectra of (*S*)-**3ba**-d_8 (**c**) and (*S*)-**3ba**.

Independent-Rate KIE Experiments

Two parallel experiments were conducted. An oven-dried Schlenk flask equipped with a stir bar was charged with $[Ir(COD)Cl]_2$ (5.1 mg, 0.0075 mmol) and (*S*)-**L8** (7.6 mg, 0.015 mmol), followed by dry toluene (3 mL). The mixture was stirred at room temperature for 1 h, to which was successively added **1b**/**1b**-d_8 (0.3 mmol), **2a** (64.1 mg, 0.36 mmol), $AgNTf_2$ (14.6 mg, 0.0375 mmol), internal standard 1-bromo-2-methoxynaphthalene (7.1 mg, 0.03 mmol) and dry toluene (3 mL). The flask was closed under an atmosphere of nitrogen, then stirred at 50 °C. An aliquot (0.8 mL) was taken at indicated time points, extracted with diethyl ether and water and then analyzed by ^{1}H NMR. These studies led to a KIE of 0.95, suggesting that the B–H bond cleavage may not be involved in the rate-determining step.

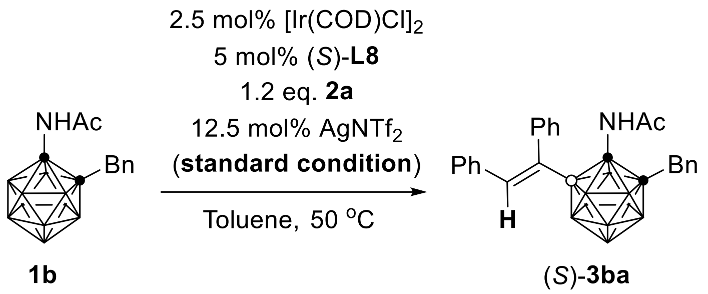

Supplementary Table 5. Yields of (*S*)-**3ba** at different reaction times

Time (min)	10	25	40	55	70
Yield (%)	4	7	13	18	23

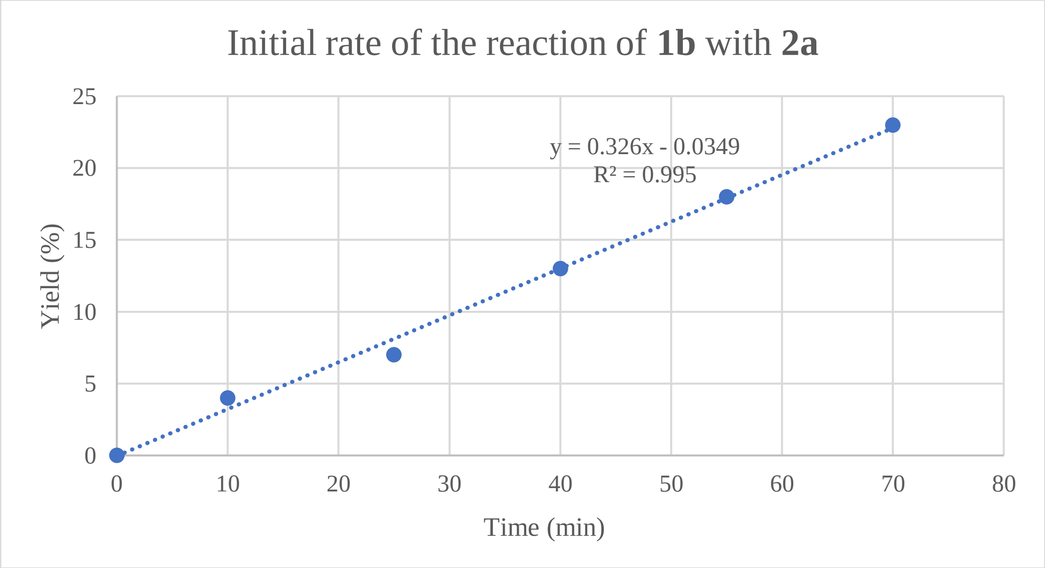

Supplementary Figure 11. Time dependent formation of (*S*)-**3ba** in the reaction of **1b** with **2a**.

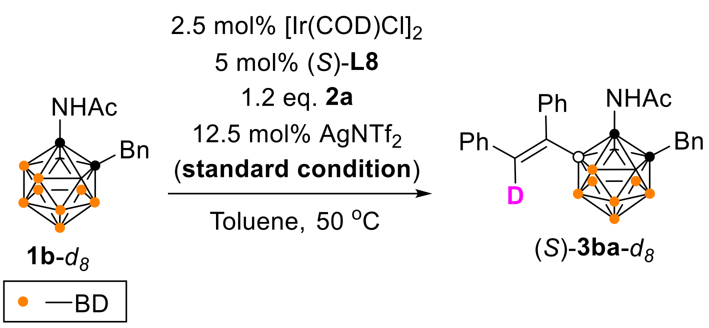

Supplementary Table 6. Yields of (S)-**3ba**-d_8 at different reaction times

Time (min)	15	20	25	30	35	40
Yield (%)	5	6	8	11	12	14

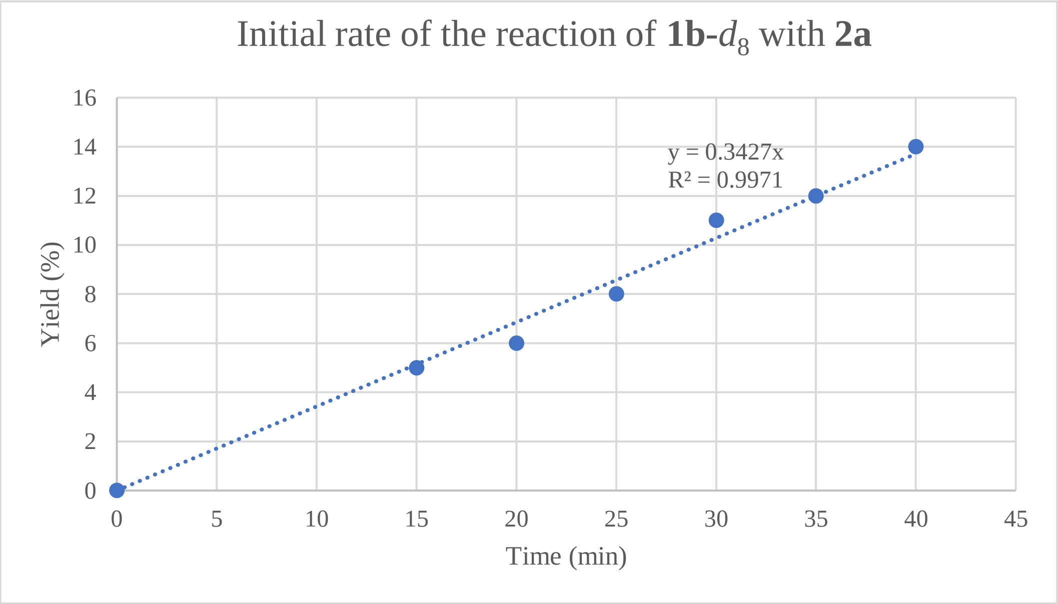

Supplementary Figure 12. Time dependent formation of (S)-**3ba**/(S)-**3ba**-d_8 in the reaction of **1b**/**1b**-d_8 with **2a**.

$$\text{KIE} = k_H/k_D = 0.3260/0.3427 = 0.95$$

X-ray Structure Determination. The data of (S)-**3ba** (170 K), (R)-**3ba** (170 K), (S)-**3bd** (170 K), (S)-**3bs** (170 K) and (S)-**3ha** (170 K) were collected on a Bruker APEX DUO diffractometer. An empirical absorption correction was applied using the SADABS program.[4] All structures were solved by direct

methods and subsequent Fourier difference techniques and refined anisotropically for all non-hydrogen atoms by full-matrix least-squares on F^2 using the SHELXTL program package.[5] All hydrogen atoms were geometrically fixed using the riding model. Crystal data and details of data collection and structure refinements were given in Supplementary Table 7.

CCDC 2081461-2081465 ((*S*)-**3ba**, (*R*)-**3ba**, (*S*)-**3bd**, (*S*)-**3bs** and (*S*)-**3ha**) contains the supplementary crystallographic data for this paper. These data can be obtained free of charge from The Cambridge Crystallographic Data Centre via www.ccdc.cam.ac.uk/data_request/cif.

Supplementary Table 7. Crystal Data and Summary of Data Collection and Refinement for (*S*)-**3ba**, (*R*)-**3ba**, (*S*)-**3bd**, (*S*)-**3bs** and (*S*)-**3ha.**

compound	(*S*)-**3ba**	(*R*)-**3ba**	(*S*)-**3bd**·EtOH	(*S*)-**3bs**	(*S*)-**3ha**·EtOH
formula	$C_{25}H_{31}B_{10}NO$	$C_{25}H_{31}B_{10}NO$	$C_{27}H_{35}B_{10}Cl_2NO_2$	$C_{29}H_{39}B_{10}NO_5$	$C_{23}H_{35}B_{10}NO_2$
crystal size (mm)	0.08x0.06x0.06	0.1x0.06x0.05	0.28x0.25x0.20	0.1x0.08x0.05	0.1x0.05x0.002
fw	469.61	469.61	584.56	589.71	465.62
crystal system	Orthorhombic	Orthorhombic	Monoclinic	Tetragonal	Orthorhombic
space group	$P2_12_12_1$	$P2_12_12_1$	$P2_1$	$P4_3$	$P2_12_12_1$
a, Å	13.800(1)	13.789 (1)	13.260(1)	19.438(1)	10.473(1)
b, Å	16.788(1)	16.780(1)	10.679(1)	19.438(1)	12.558(1)
c, Å	23.492(1)	23.470(1)	22.220(2)	18.088 (1)	20.954 (2)
α, deg	90	90	90	90	90
β, deg	90	90	96.082(6)	90	90
γ, deg	90	90	90	90	90
V, Å^3	5442.2(3)	5430.4(2)	3128.6(6)	6834.2(4)	2755.8(4)
Z	8	8	4	8	4
D_{calcd}, Mg/m^3	1.146	1.149	1.241	1.146	1.122
radiation (λ) Å	1.34139	1.34139	1.34139	1.34139	1.34139
2θ range, deg	5.6 to 109.8	5.6 to 110.1	6.5 to 109.9	5.8 to 109.9	7.1 to 110.0
μ, mm^{-1}	0.300	0.301	1.345	0.357	0.308
F(000)	648	1968	1216	2480	984
no. of obsd reflns	10291	10326	11401	12973	4898
no. of params refnd	669	669	751	822	329
goodness of fit	1.039	0.870	1.029	1.039	1.024
R1	0.0515	0.0522	0.0711	0.0516	0.0565
wR2	0.1231	0.1107	0.1907	0.1162	0.1390
absolute structure parameter	0.03(14)	0.05(13)	0.054(7)	0.01(11)	0.00(18)

Computational Details. Geometry optimizations were carried out with the Gaussian09 program, Revision D.01[6] at the B3LYP[7]-D3[8] level of density functional theory. The Los Alamos National Laboratory (LANL) effective core potentials (ECP) with the appropriate valence basis set of double- ζ quality (denoted LANL2DZ)[9] was used for Ir while the 6-31g(d,p) basis set was used for all other atoms. Frequency calculations were made to determine the characteristics of all stationary points as transition states. NCI analysis was performed using Multiwfn software.[10] The graphics of the NCI interactions were produced by using the visualizing software VMD.[11]

Supplementary Figure 13. ^{1}H NMR of **1a**.

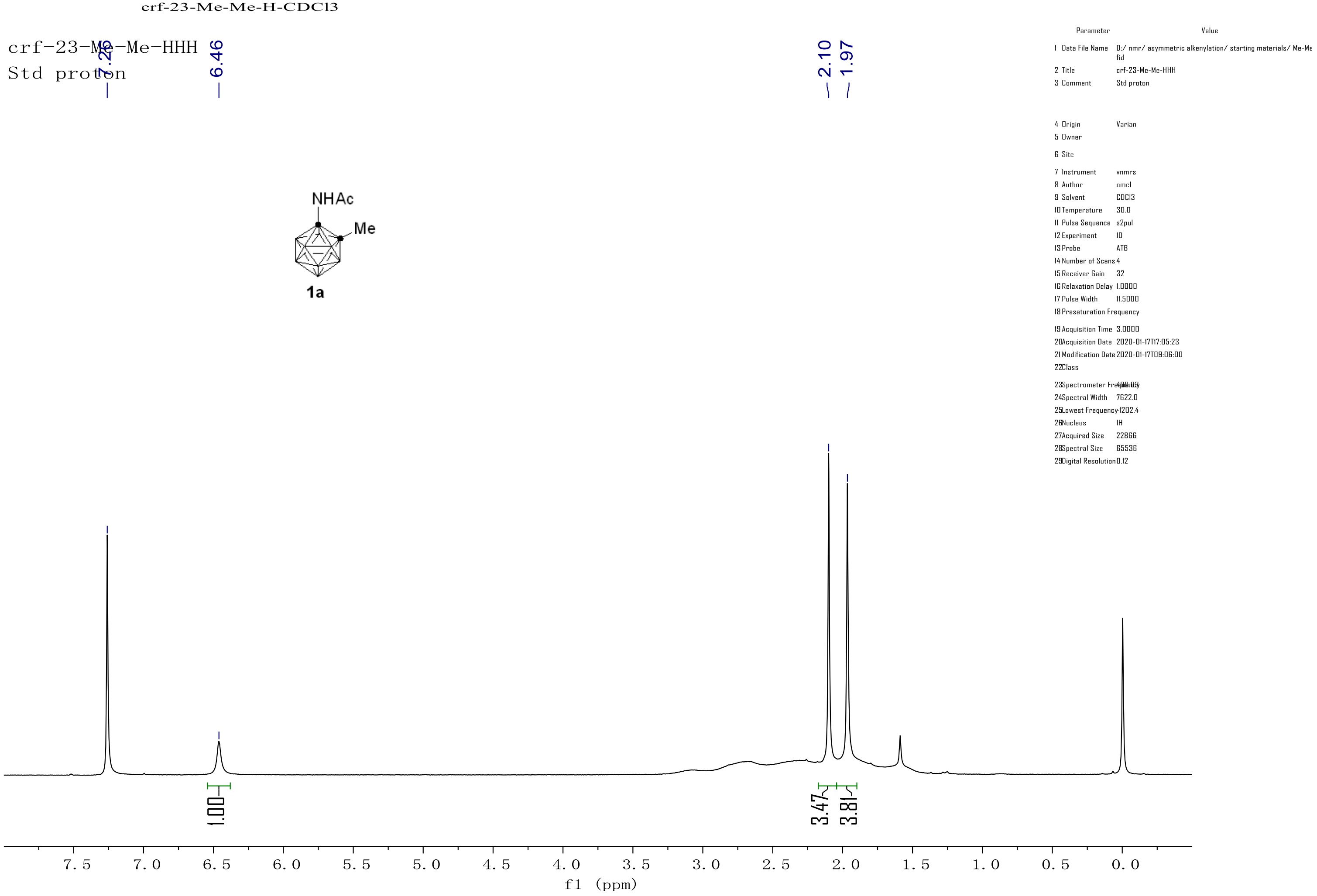

Supplementary Figure 14. $^{13}C\{^{1}H\}$ NMR of **1a**.

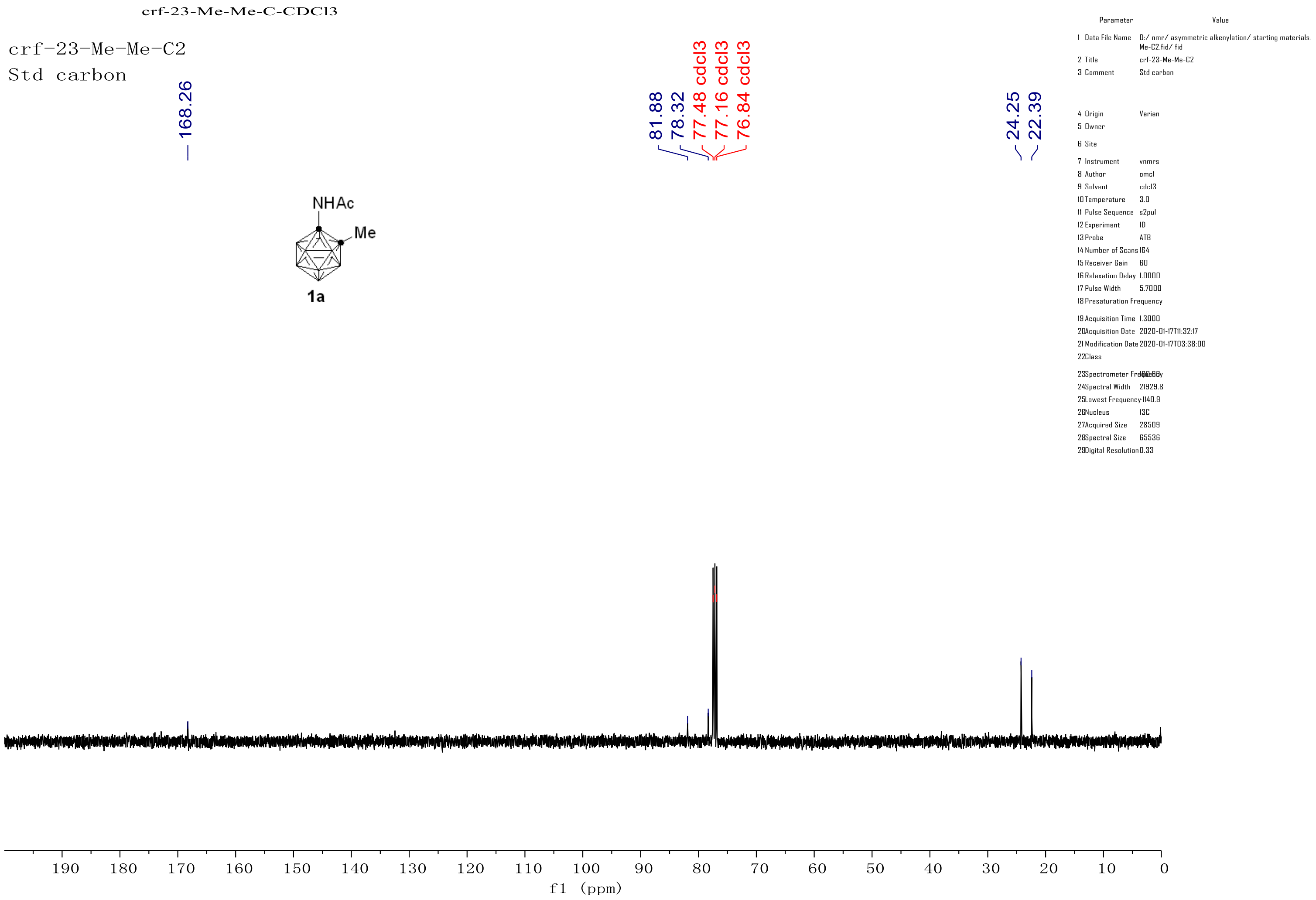

Supplementary Figure 15. $^{11}B\{^1H\}$ NMR of **1a**.

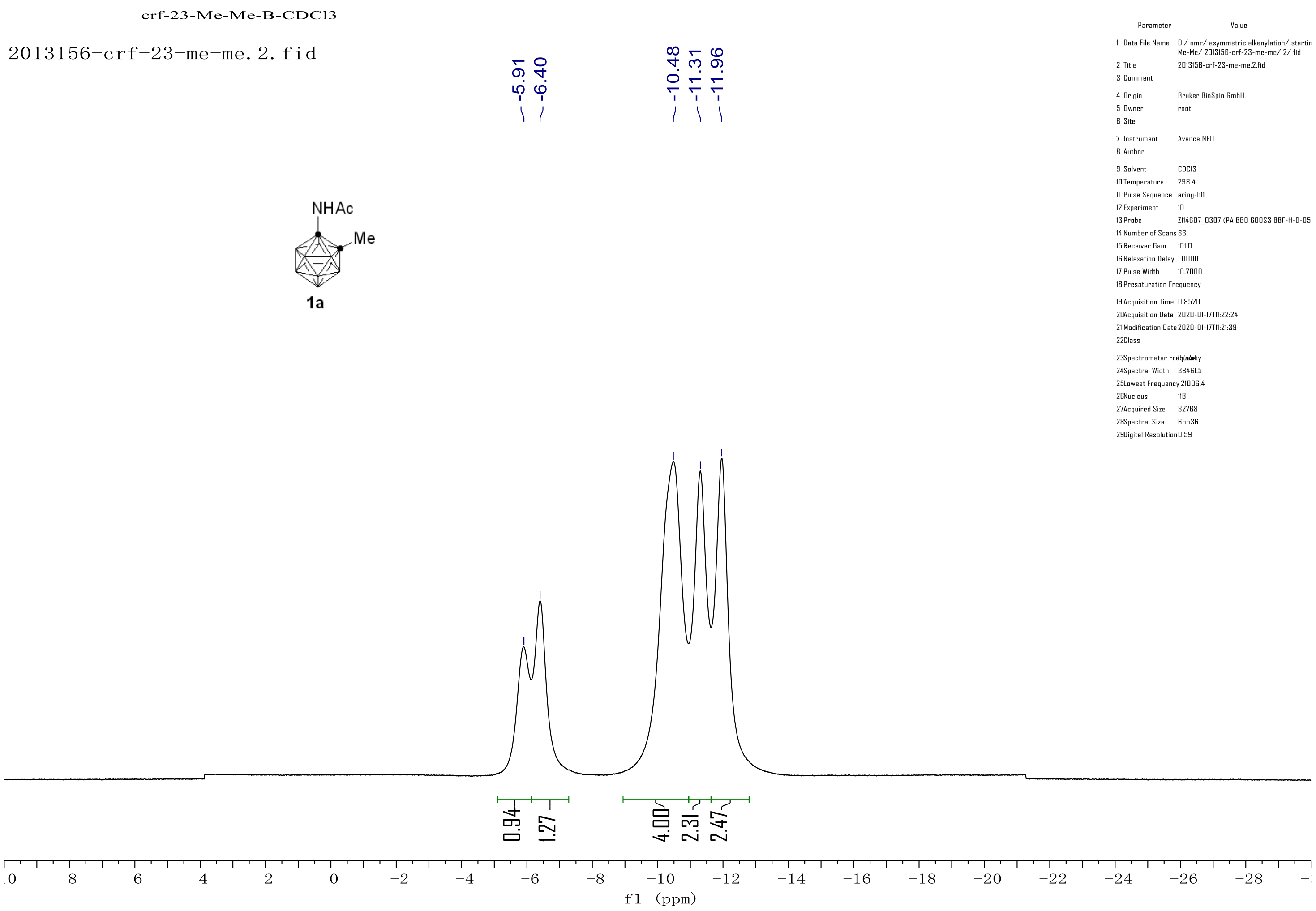

Supplementary Figure 16. ^{1}H NMR of **1b**.

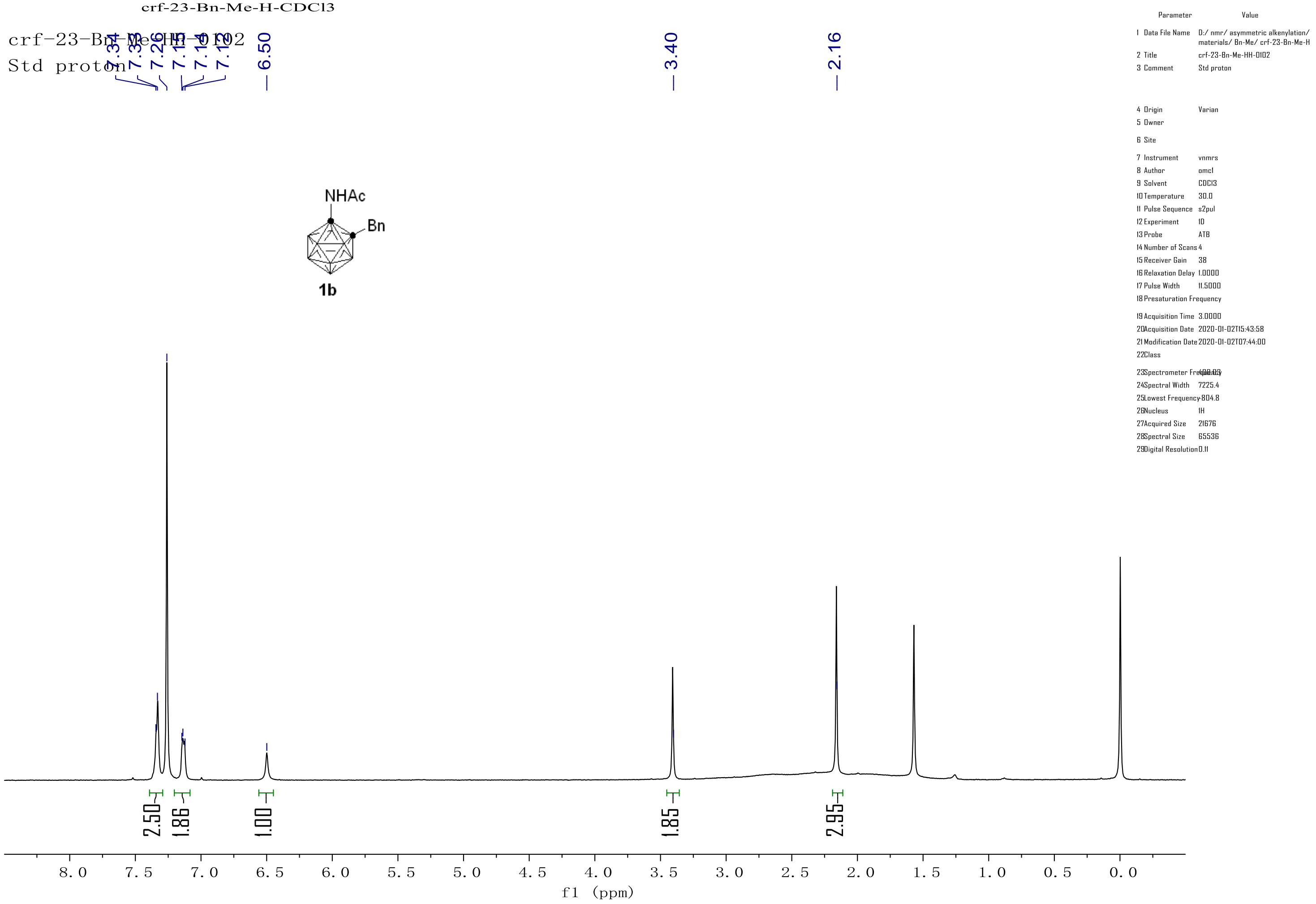

Supplementary Figure 17. $^{13}C\{^{1}H\}$ NMR of **1b**.

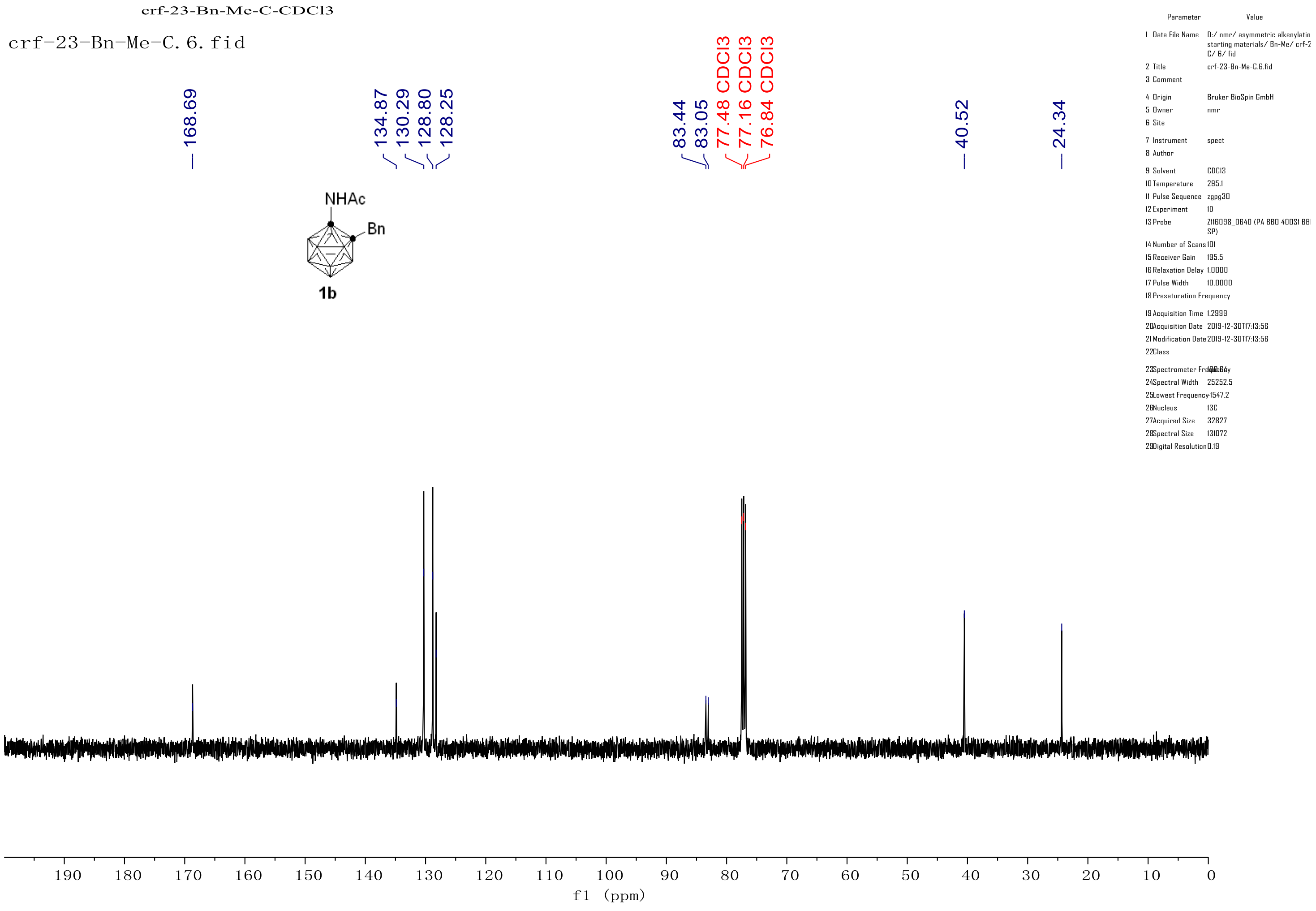

Supplementary Figure 18. $^{11}B\{^{1}H\}$ NMR of **1b**.

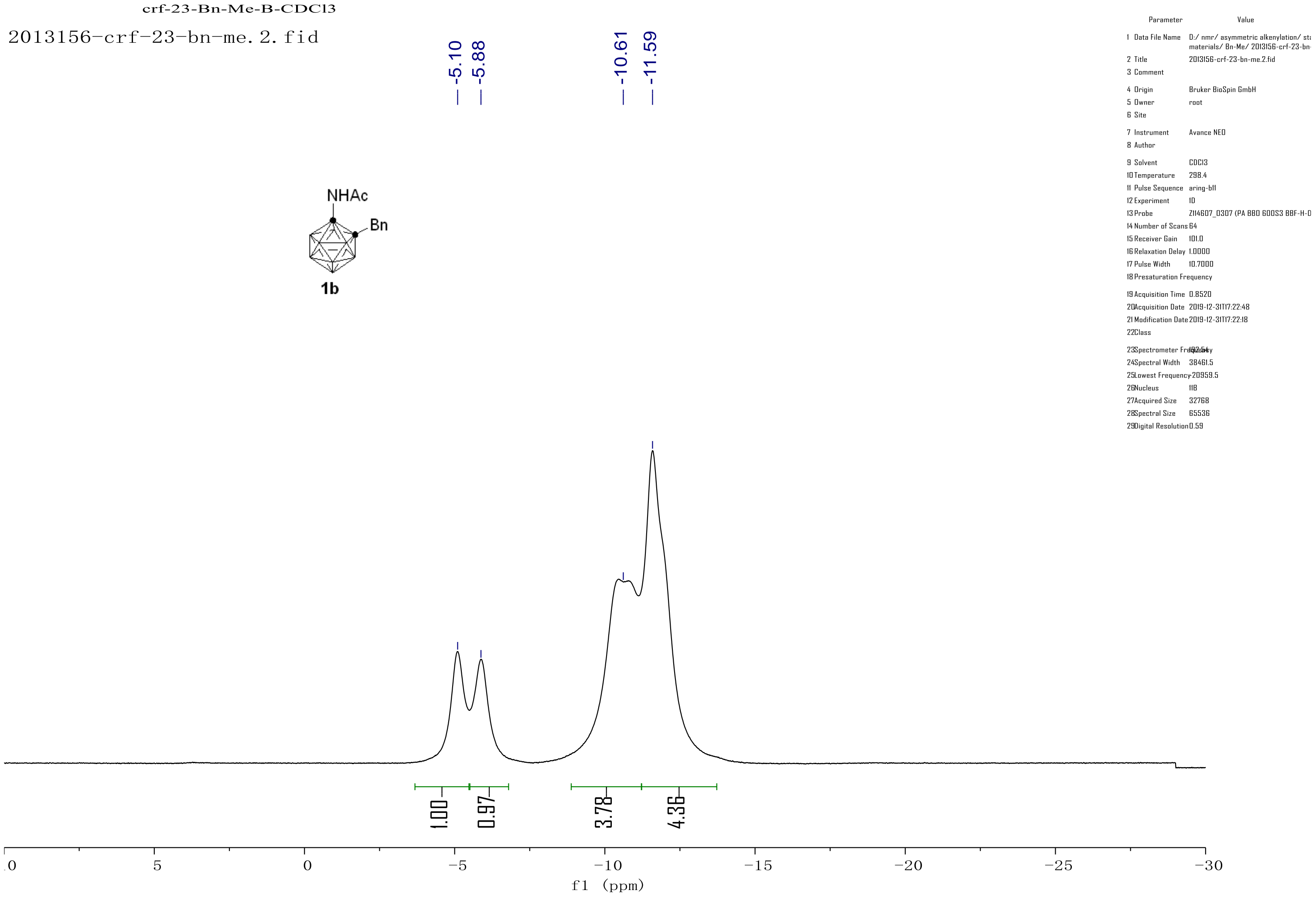

Supplementary Figure 19. ^{1}H NMR of **1c**.

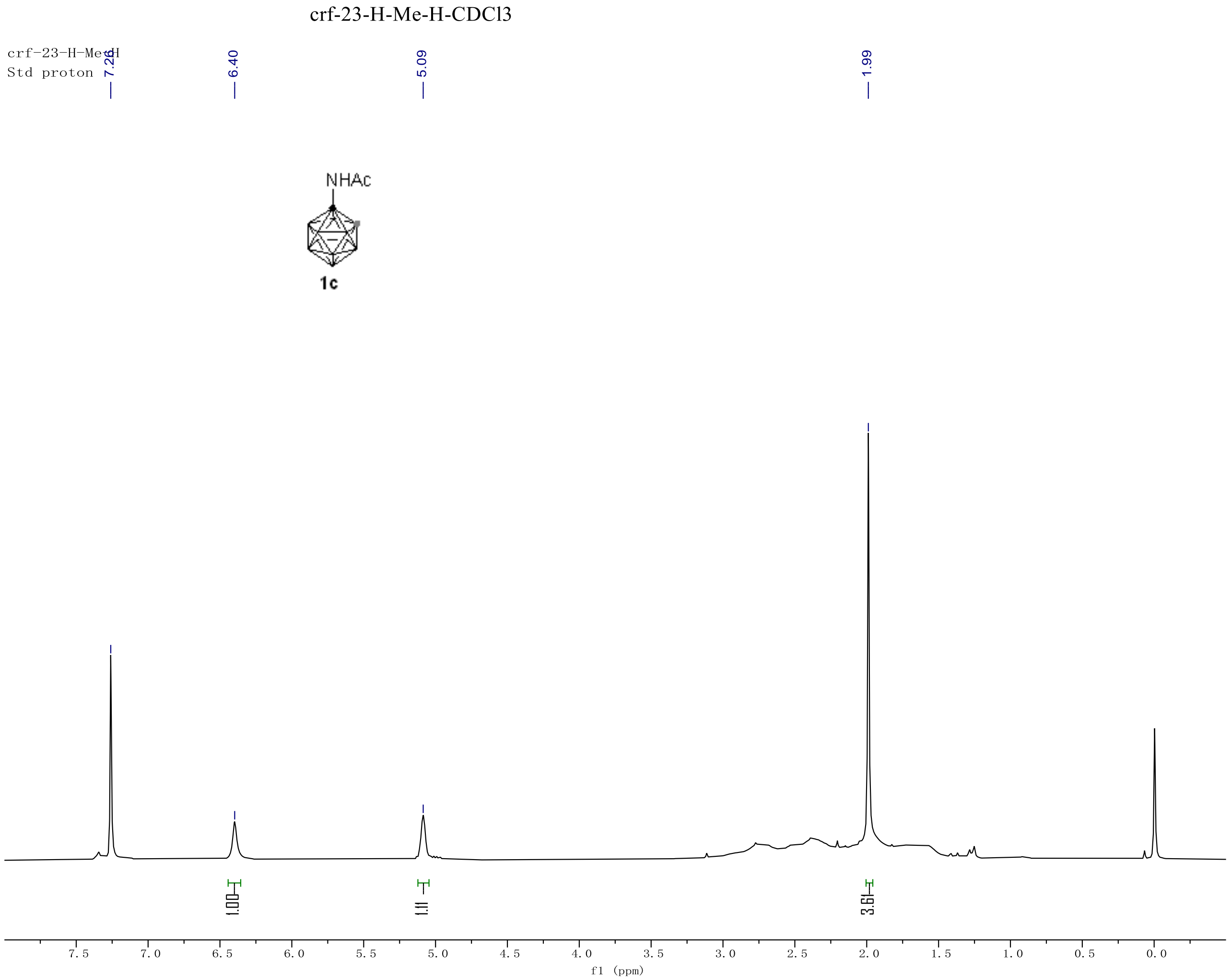

	Parameter	Value
1	Data File Name	D:/ nmr/ crf-23-H-Me-H.fid/ fid
2	Title	crf-23-H-Me-H
3	Comment	Std proton
4	Origin	Varian
5	Owner	
6	Site	
7	Instrument	vnmrs
8	Author	omcl
9	Solvent	CDCl3
10	Temperature	30.0
11	Pulse Sequence	s2pul
12	Experiment	1D
13	Probe	ATB
14	Number of Scans	8
15	Receiver Gain	32
16	Relaxation Delay	1.0000
17	Pulse Width	11.5000
18	Presaturation Frequency	
19	Acquisition Time	3.0000
20	Acquisition Date	2019-12-18T15:37:55
21	Modification Date	2019-12-18T07:38:00
22	Class	
23	Spectrometer Frequency	400.03
24	Spectral Width	7622.0
25	Lowest Frequency	-1203.0
26	Nucleus	1H
27	Acquired Size	22866
28	Spectral Size	65536
29	Digital Resolution	0.12

Supplementary Figure 20. $^{13}C\{^1H\}$ NMR of **1c**.

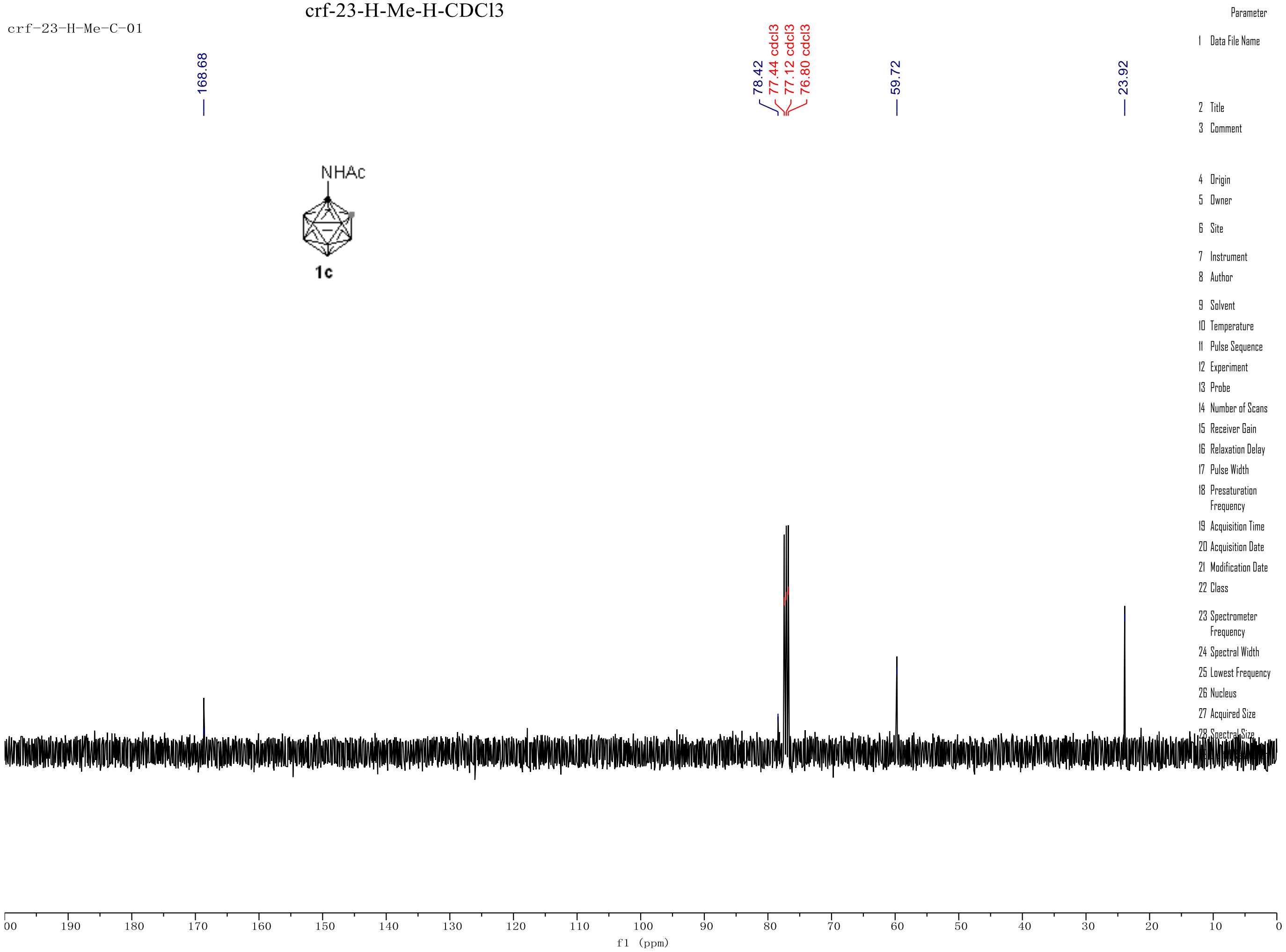

	Parameter	Value
1	Data File Name	D:/ nmr/ asymmetric alkenylation/ starting materials/ H-Me/ crf-23-H-Me-C-01.fid/ fid
2	Title	crf-23-H-Me-C-01
3	Comment	
4	Origin	Varian
5	Owner	
6	Site	
7	Instrument	vnmrs
8	Author	
9	Solvent	cdcl3
10	Temperature	25.0
11	Pulse Sequence	s2pul
12	Experiment	1D
13	Probe	4nuc
14	Number of Scans	48
15	Receiver Gain	60
16	Relaxation Delay	1.0000
17	Pulse Width	5.2000
18	Presaturation Frequency	
19	Acquisition Time	1.3000
20	Acquisition Date	2019-12-19T15:42:57
21	Modification Date	2019-12-19T07:45:00
22	Class	
23	Spectrometer Frequency	100.52
24	Spectral Width	25000.0
25	Lowest Frequency	-1433.5
26	Nucleus	13C
27	Acquired Size	32500
28	Spectral Size	65536
[illegible]	[illegible]	0.38

Supplementary Figure 21. $^{11}B\{^{1}H\}$ NMR of **1c**.

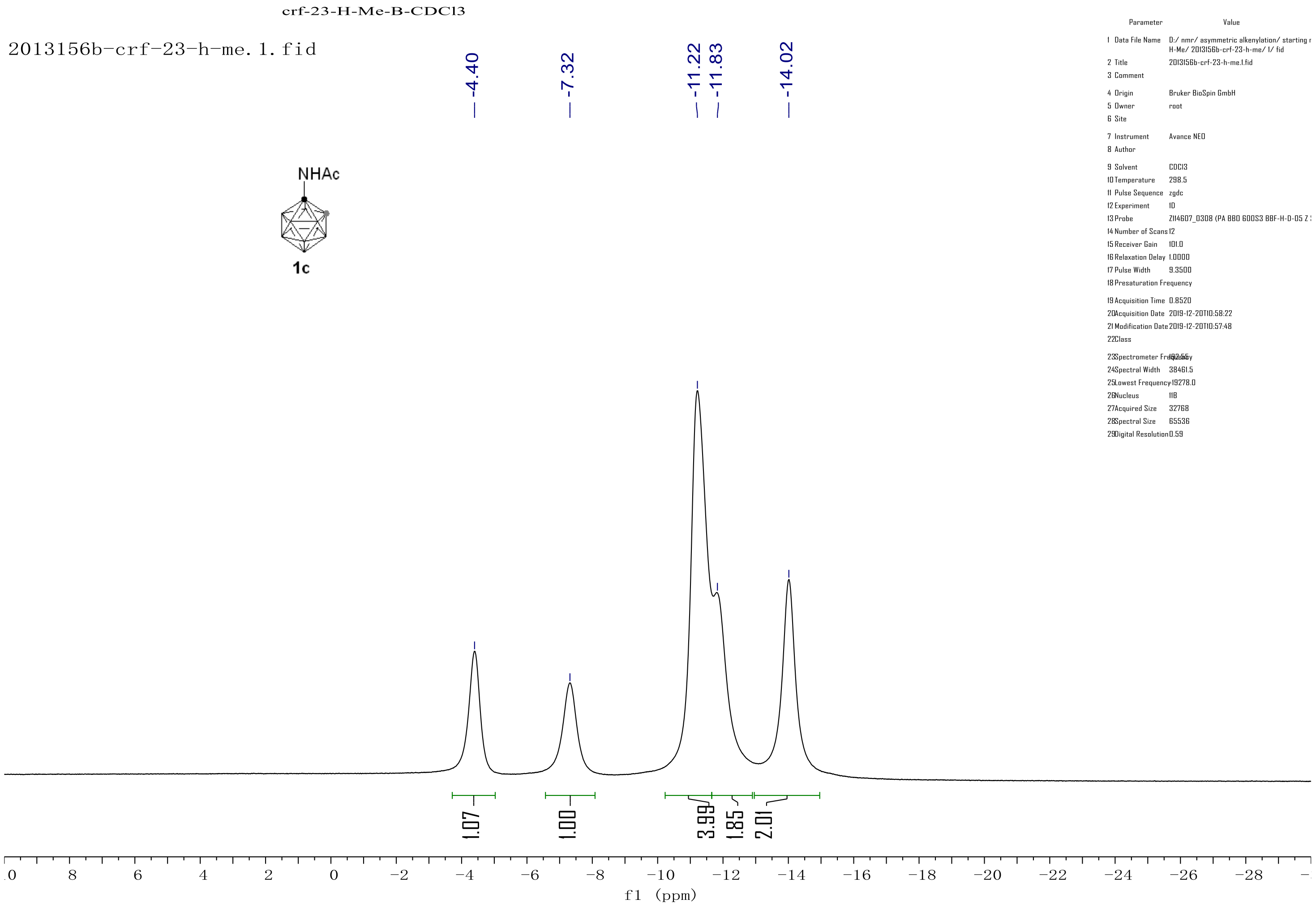

Supplementary Figure 22. ^{1}H NMR of **1d**.

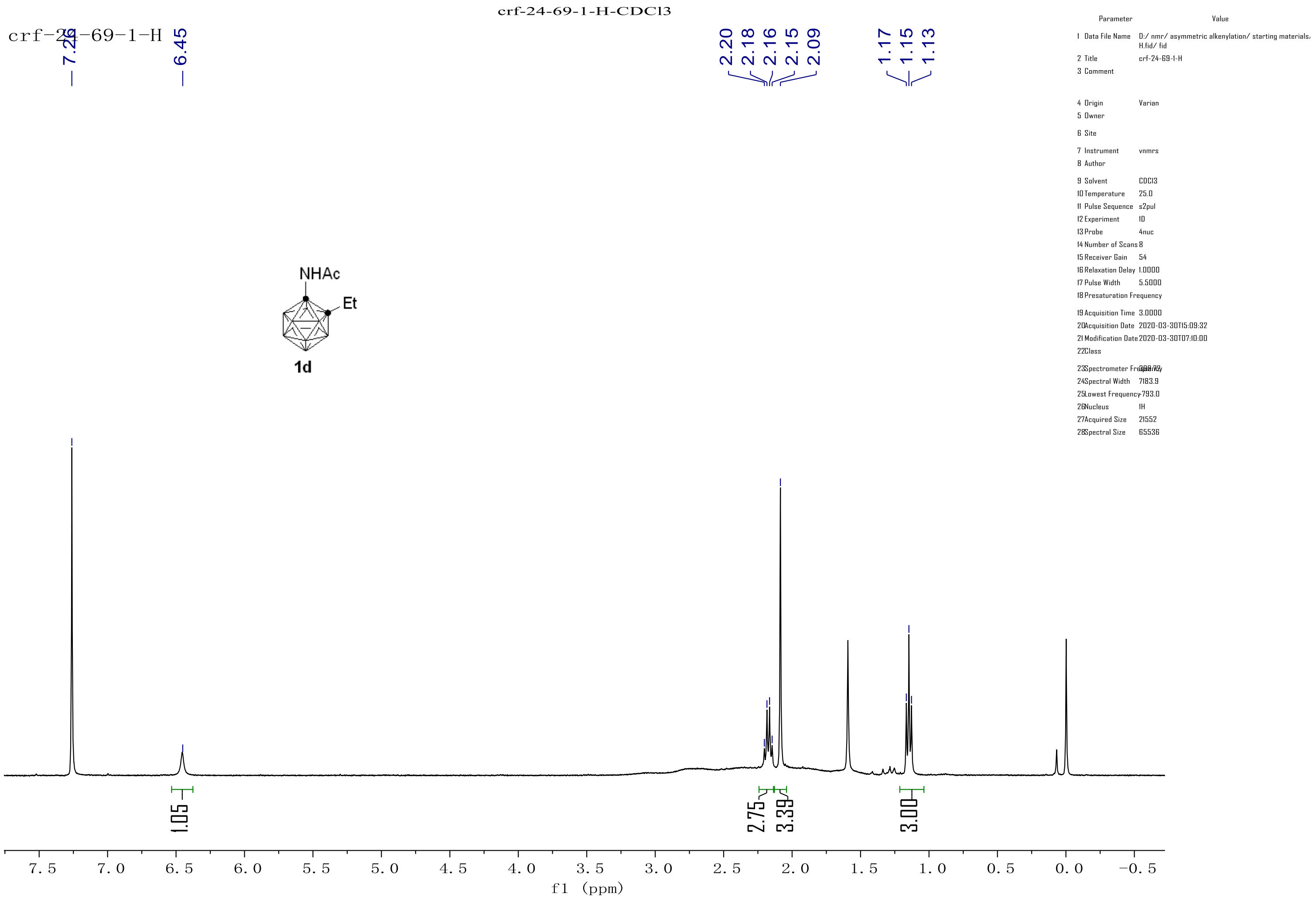

Supplementary Figure 23. $^{13}C\{^{1}H\}$ NMR of **1d**.

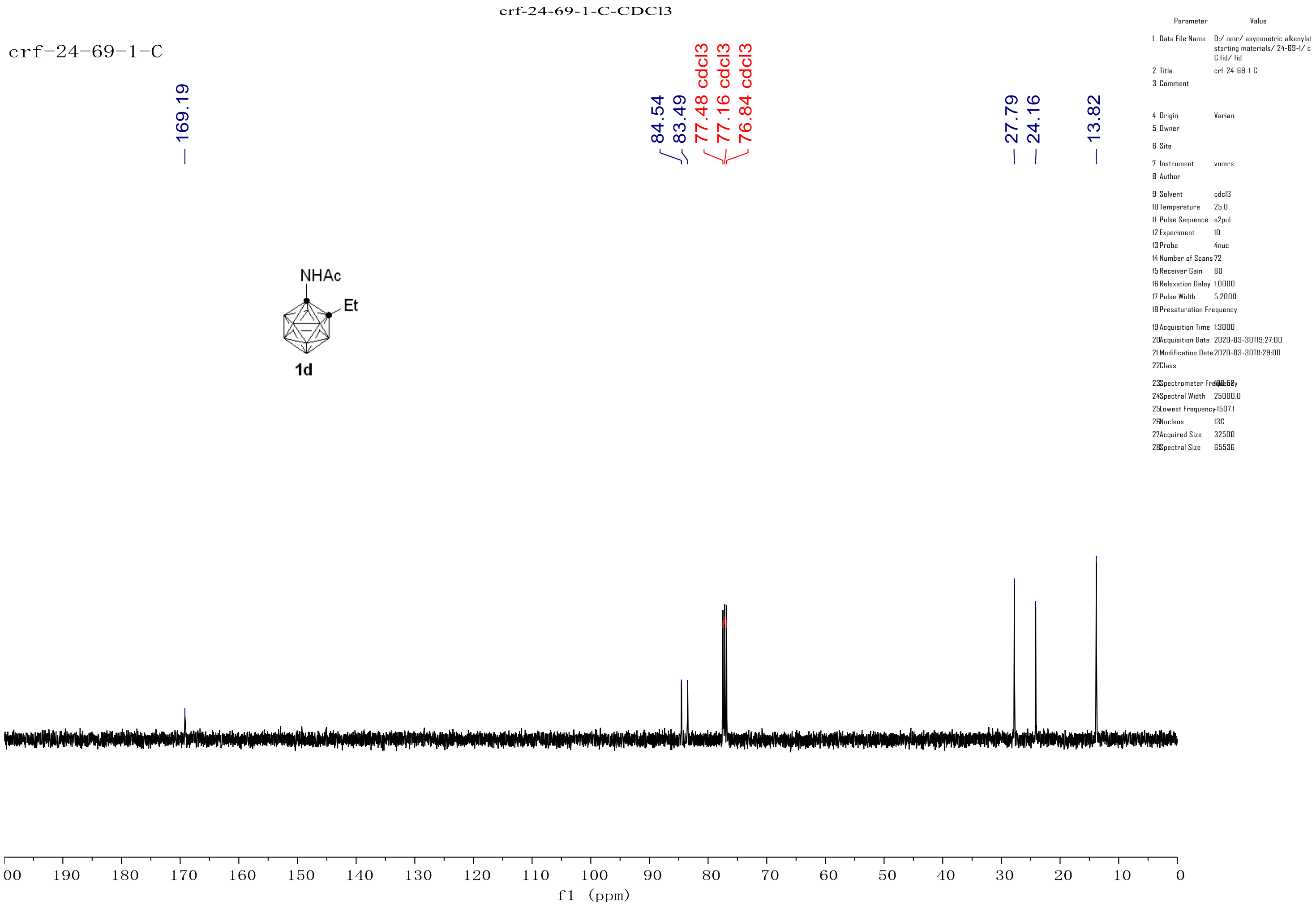

Supplementary Figure 24. $^{11}B\{^{1}H\}$ NMR of **1d**.

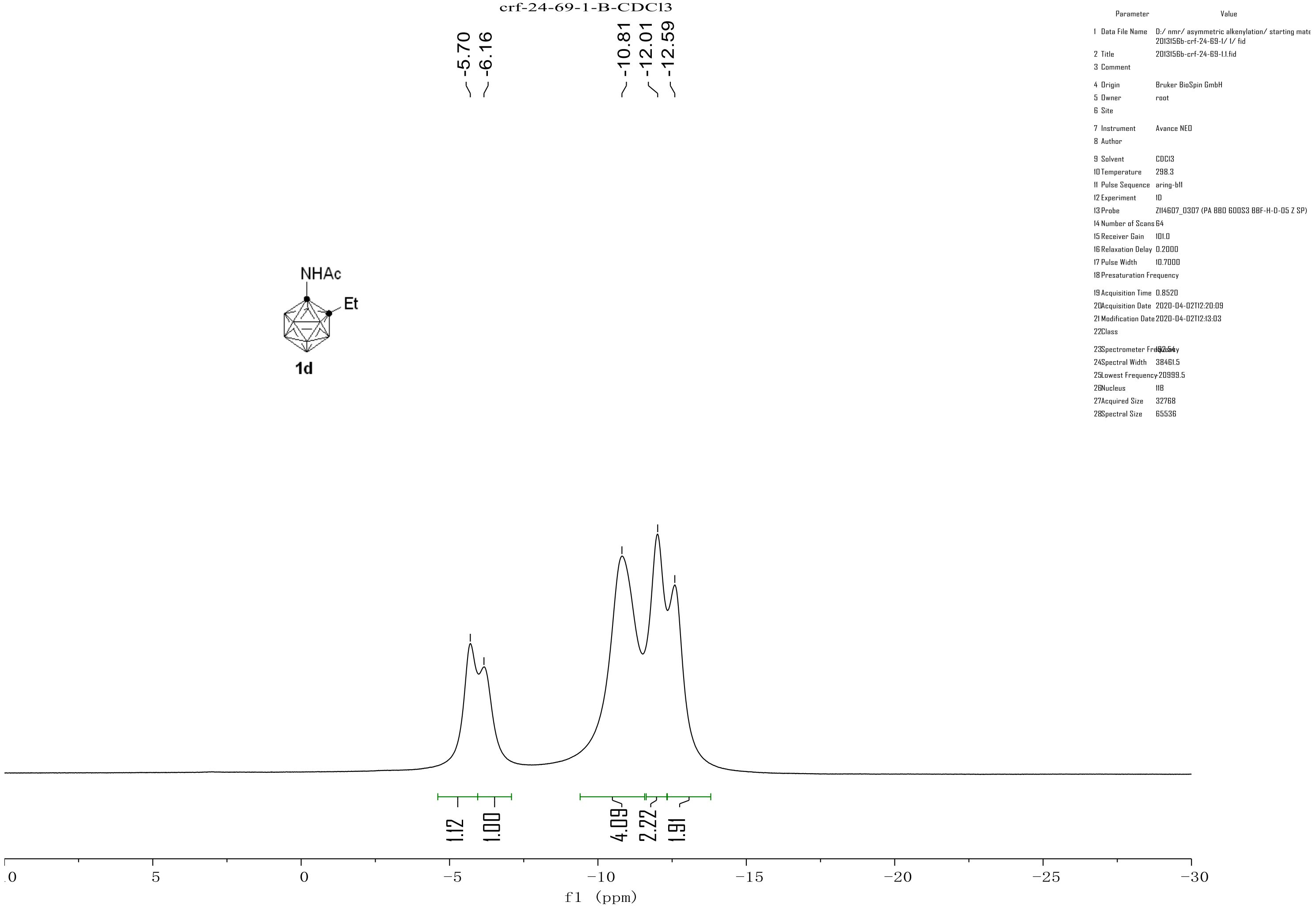

Supplementary Figure 25. ^{1}H NMR of **1e**.

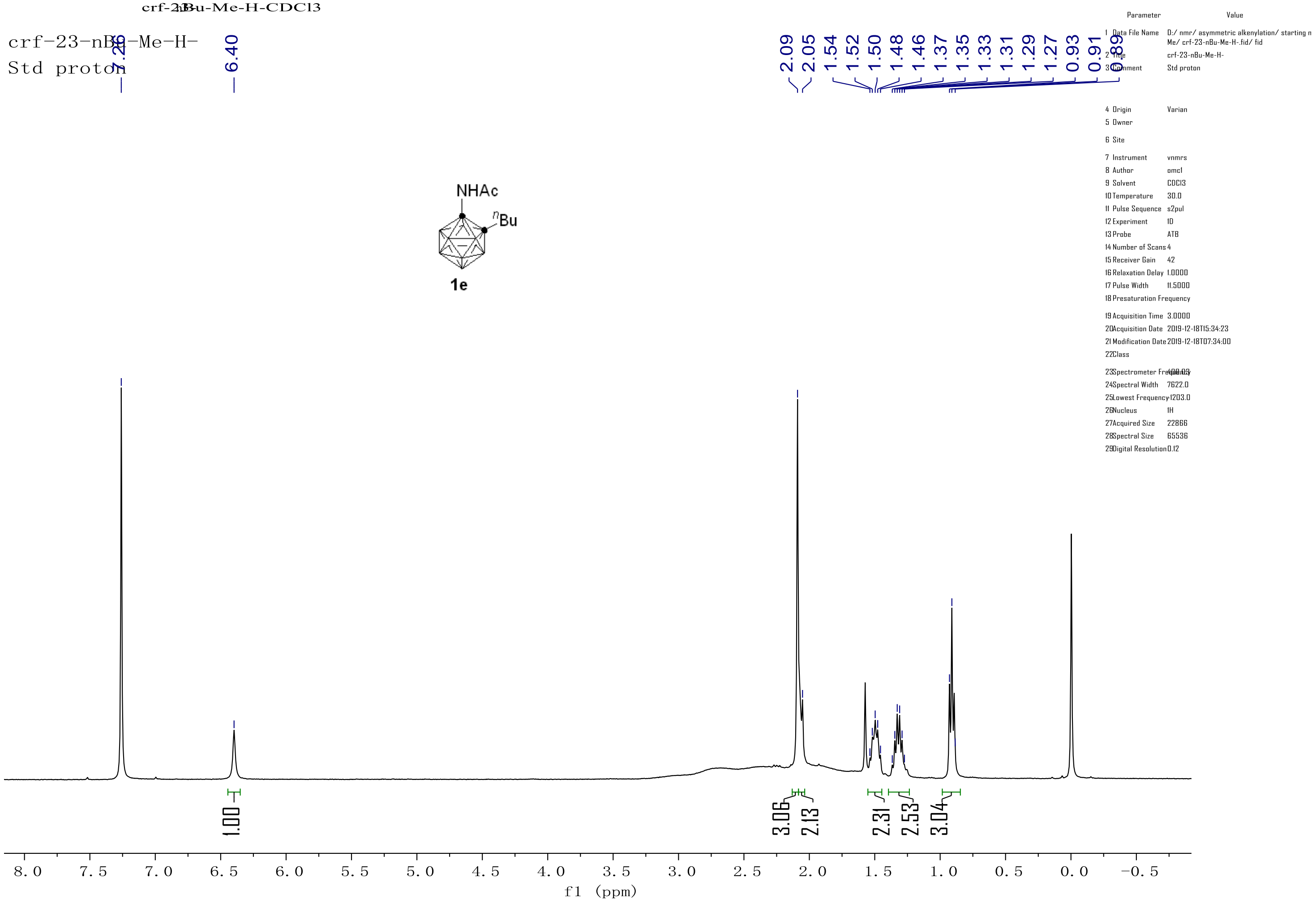

Supplementary Figure 26. $^{13}C\{^{1}H\}$ NMR of **1e**.

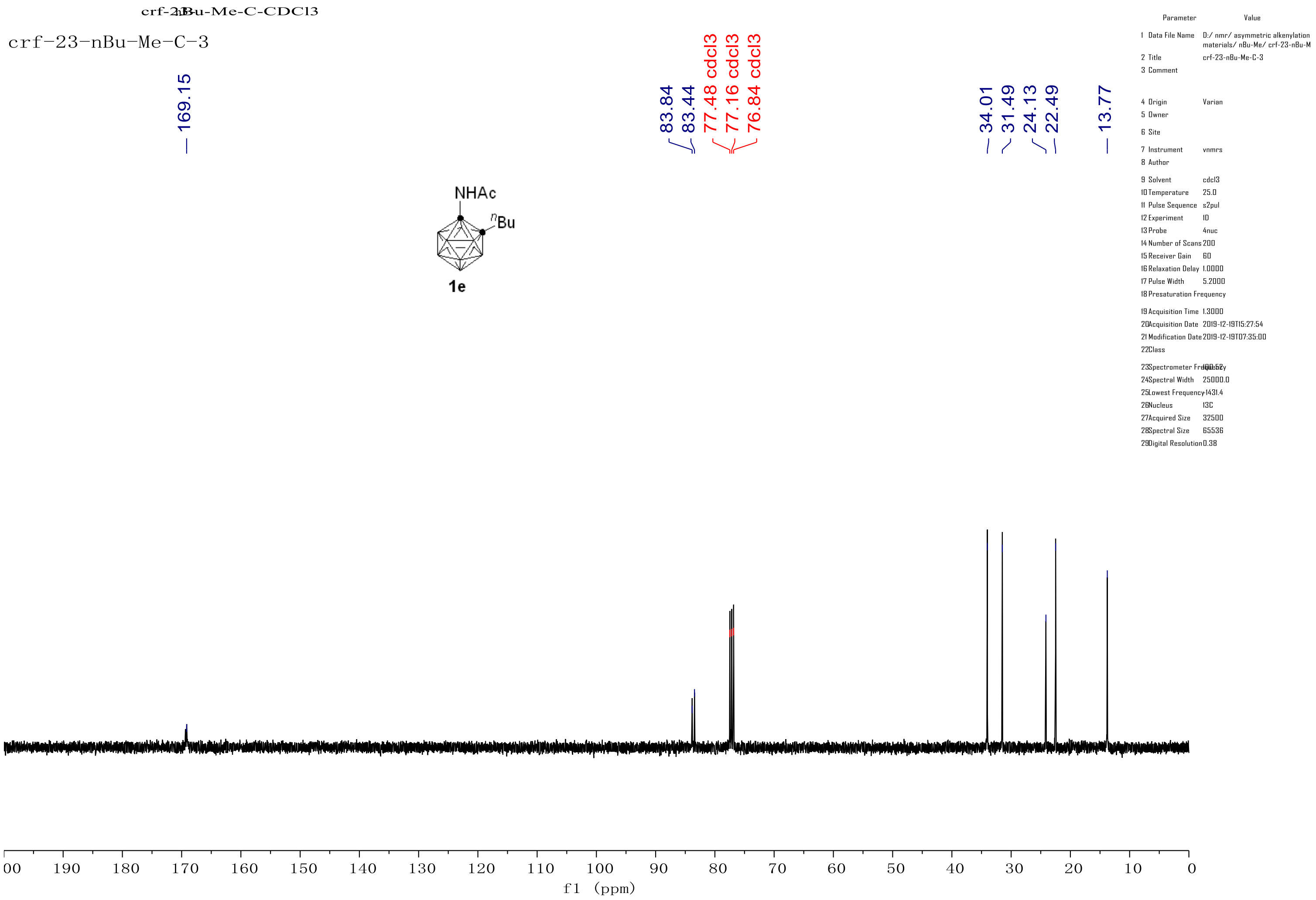

Supplementary Figure 27. $^{11}B\{^1H\}$ NMR of **1e**.

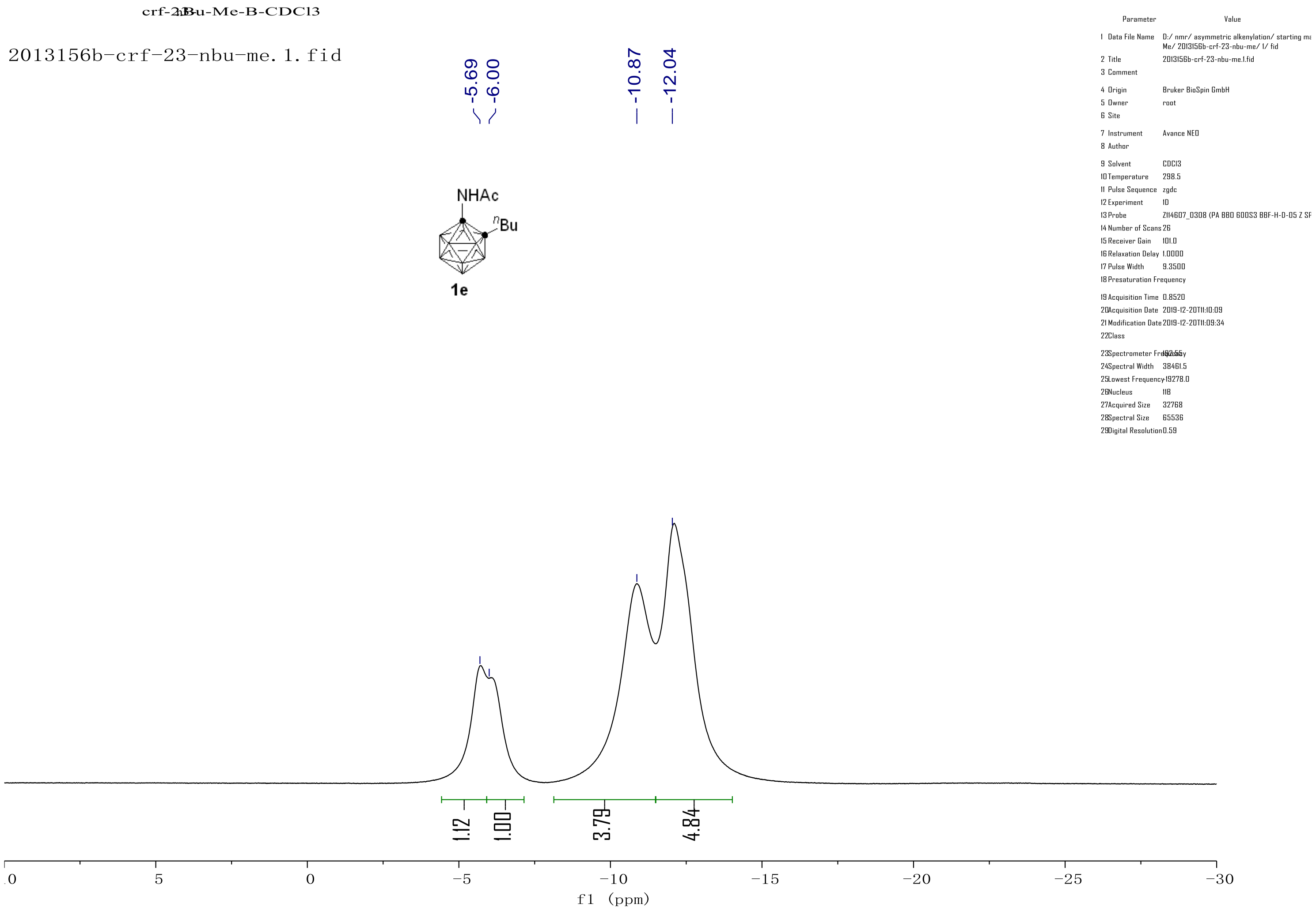

Supplementary Figure 28. ^{1}H NMR of **1f**.

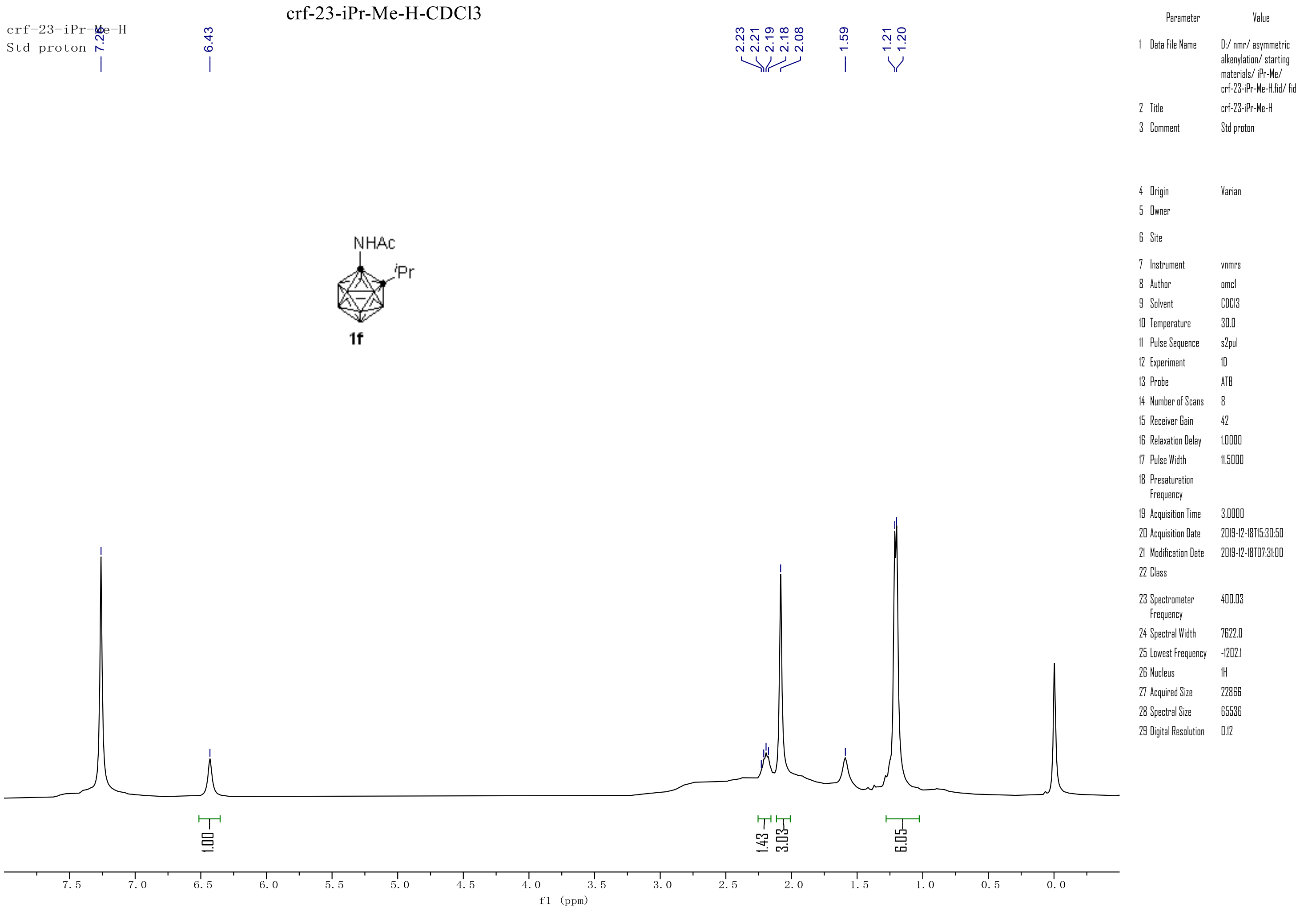

Supplementary Figure 29. $^{13}C\{^1H\}$ NMR of **1f**.

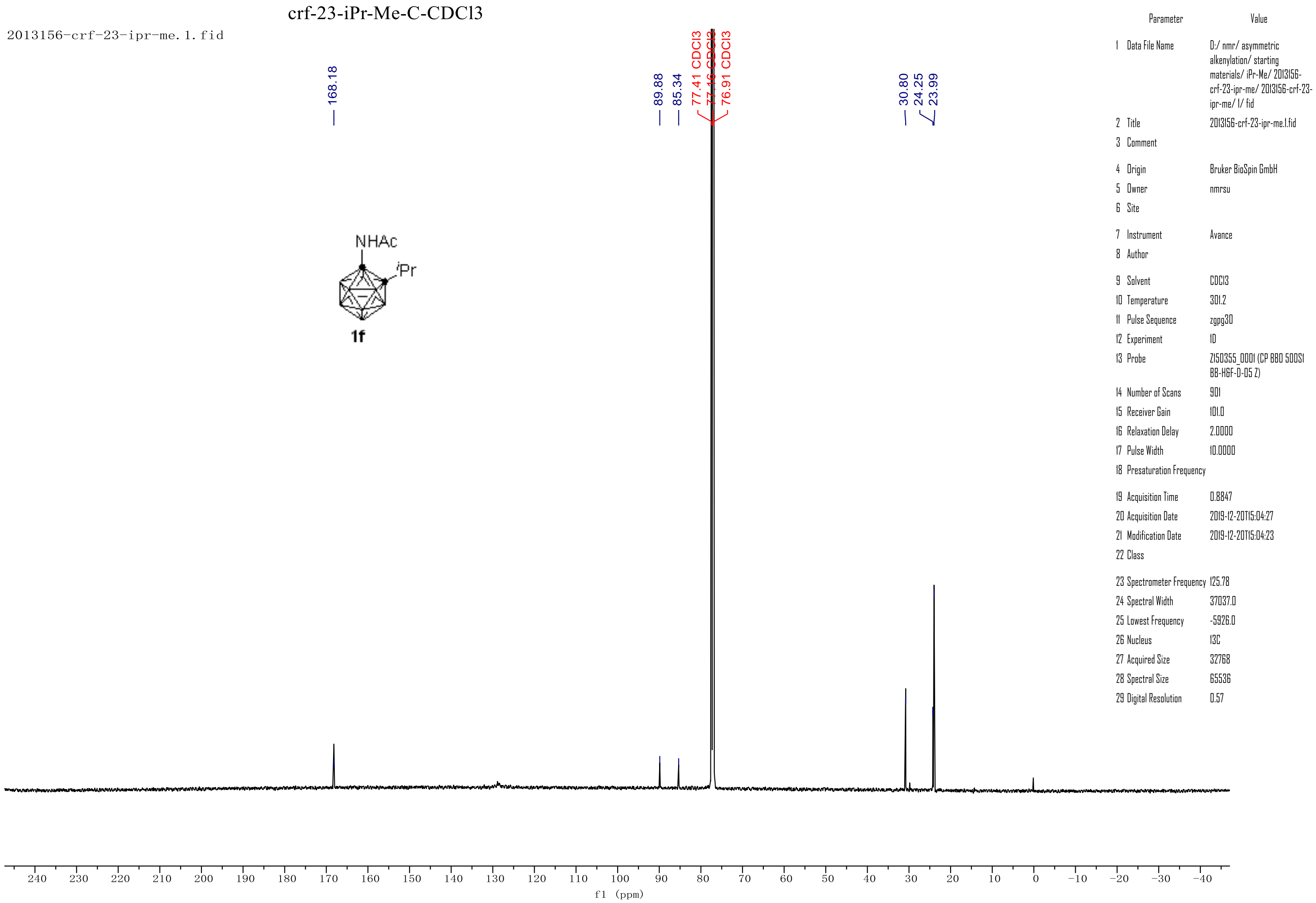

	Parameter	Value
1	Data File Name	D:/ nmr/ asymmetric alkenylation/ starting materials/ iPr-Me/ 2013156-crf-23-ipr-me/ 2013156-crf-23-ipr-me/ 1/ fid
2	Title	2013156-crf-23-ipr-me.1.fid
3	Comment	
4	Origin	Bruker BioSpin GmbH
5	Owner	nmrsu
6	Site	
7	Instrument	Avance
8	Author	
9	Solvent	CDCl3
10	Temperature	301.2
11	Pulse Sequence	zgpg30
12	Experiment	1D
13	Probe	Z150355_0001 (CP BBO 500S1 BB-H&F-D-05 Z)
14	Number of Scans	901
15	Receiver Gain	101.0
16	Relaxation Delay	2.0000
17	Pulse Width	10.0000
18	Presaturation Frequency	
19	Acquisition Time	0.8847
20	Acquisition Date	2019-12-20T15:04:27
21	Modification Date	2019-12-20T15:04:23
22	Class	
23	Spectrometer Frequency	125.78
24	Spectral Width	37037.0
25	Lowest Frequency	-5926.0
26	Nucleus	13C
27	Acquired Size	32768
28	Spectral Size	65536
29	Digital Resolution	0.57

Supplementary Figure 30. $^{11}B\{^{1}H\}$ NMR of **1f**.

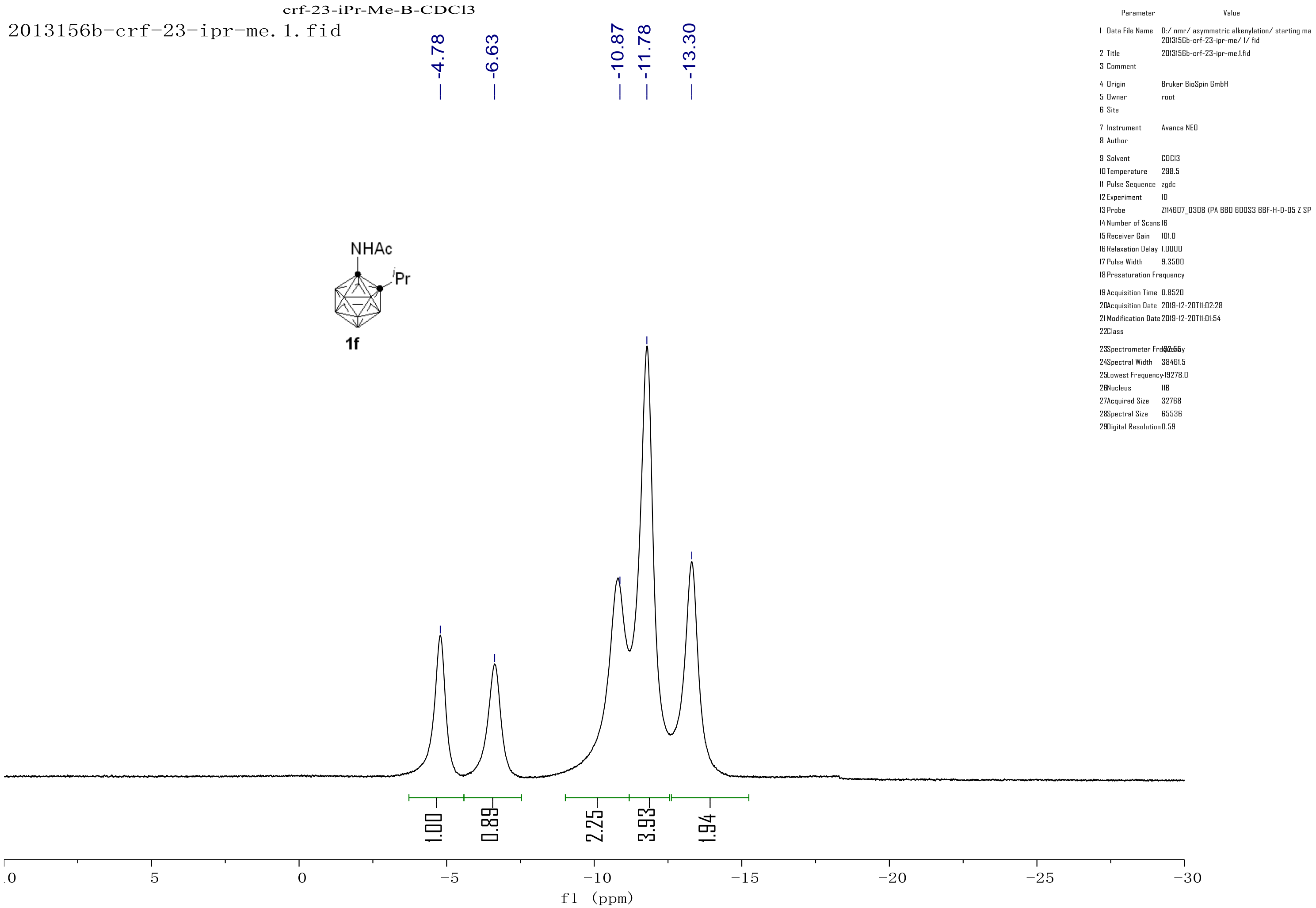

Supplementary Figure 31. ^{1}H NMR of **1g**.

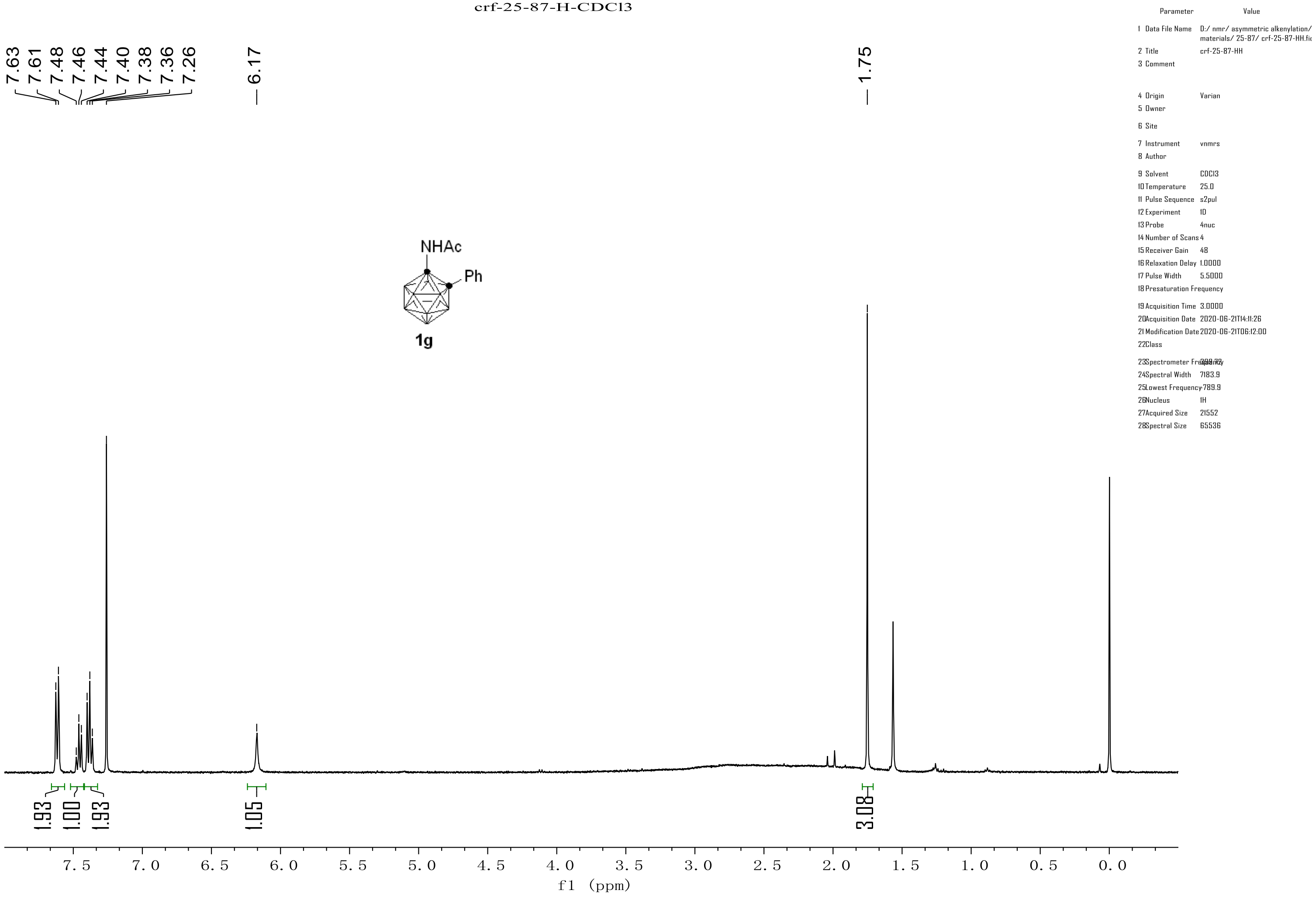

	Parameter	Value
1	Data File Name	D:/ nmr/ asymmetric alkenylation/ materials/ 25-87/ crf-25-87-HH.fid
2	Title	crf-25-87-HH
3	Comment	
4	Origin	Varian
5	Owner	
6	Site	
7	Instrument	vnmrs
8	Author	
9	Solvent	CDCl3
10	Temperature	25.0
11	Pulse Sequence	s2pul
12	Experiment	1D
13	Probe	4nuc
14	Number of Scans	4
15	Receiver Gain	48
16	Relaxation Delay	1.0000
17	Pulse Width	5.5000
18	Presaturation Frequency	
19	Acquisition Time	3.0000
20	Acquisition Date	2020-06-21T14:11:26
21	Modification Date	2020-06-21T06:12:00
22	Class	
23	Spectrometer Frequency	[illegible]
24	Spectral Width	7183.9
25	Lowest Frequency	-789.9
26	Nucleus	1H
27	Acquired Size	21552
28	Spectral Size	65536

Supplementary Figure 32. $^{13}C\{^{1}H\}$ NMR of **1g**.

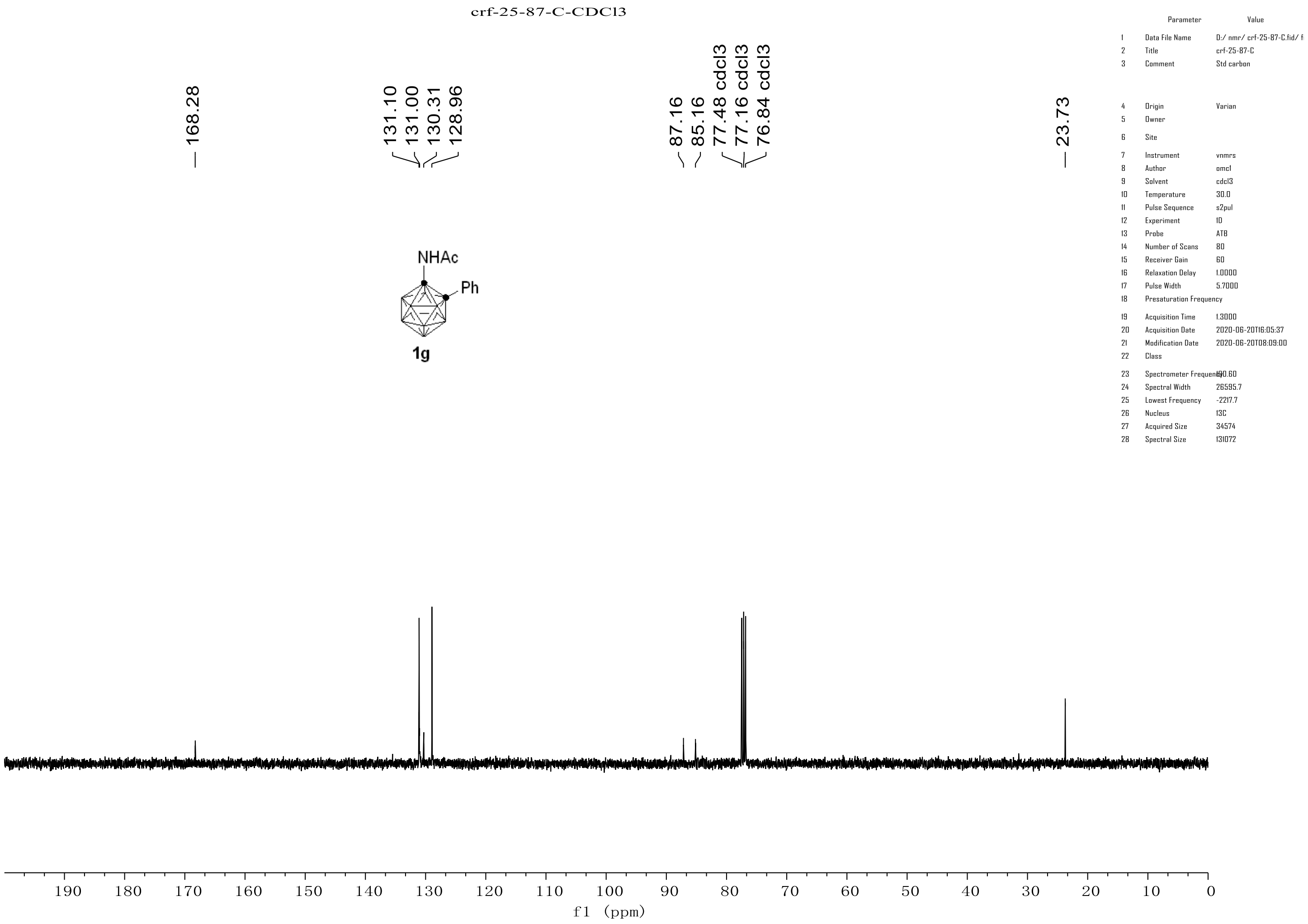

	Parameter	Value
1	Data File Name	D:/ nmr/ crf-25-87-C.fid/ f
2	Title	crf-25-87-C
3	Comment	Std carbon
4	Origin	Varian
5	Owner	
6	Site	
7	Instrument	vnmrs
8	Author	omcl
9	Solvent	cdcl3
10	Temperature	30.0
11	Pulse Sequence	s2pul
12	Experiment	1D
13	Probe	ATB
14	Number of Scans	80
15	Receiver Gain	60
16	Relaxation Delay	1.0000
17	Pulse Width	5.7000
18	Presaturation Frequency	
19	Acquisition Time	1.3000
20	Acquisition Date	2020-06-20T16:05:37
21	Modification Date	2020-06-20T08:09:00
22	Class	
23	Spectrometer Frequency	150.60
24	Spectral Width	26595.7
25	Lowest Frequency	-2217.7
26	Nucleus	13C
27	Acquired Size	34574
28	Spectral Size	131072

Supplementary Figure 33. $^{11}B\{^{1}H\}$ NMR of **1g**.

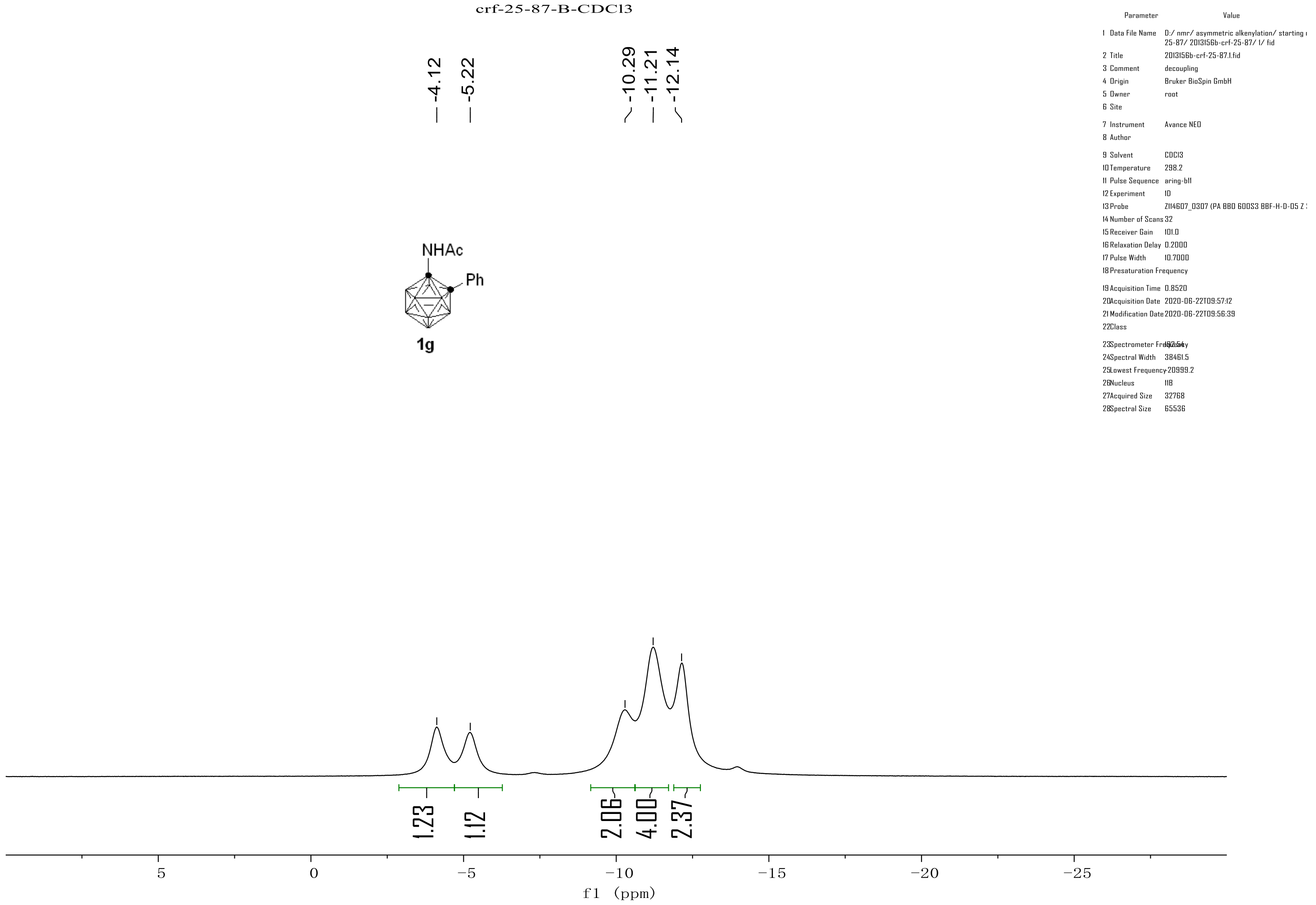

Supplementary Figure 34. ^{1}H NMR of **1h**.

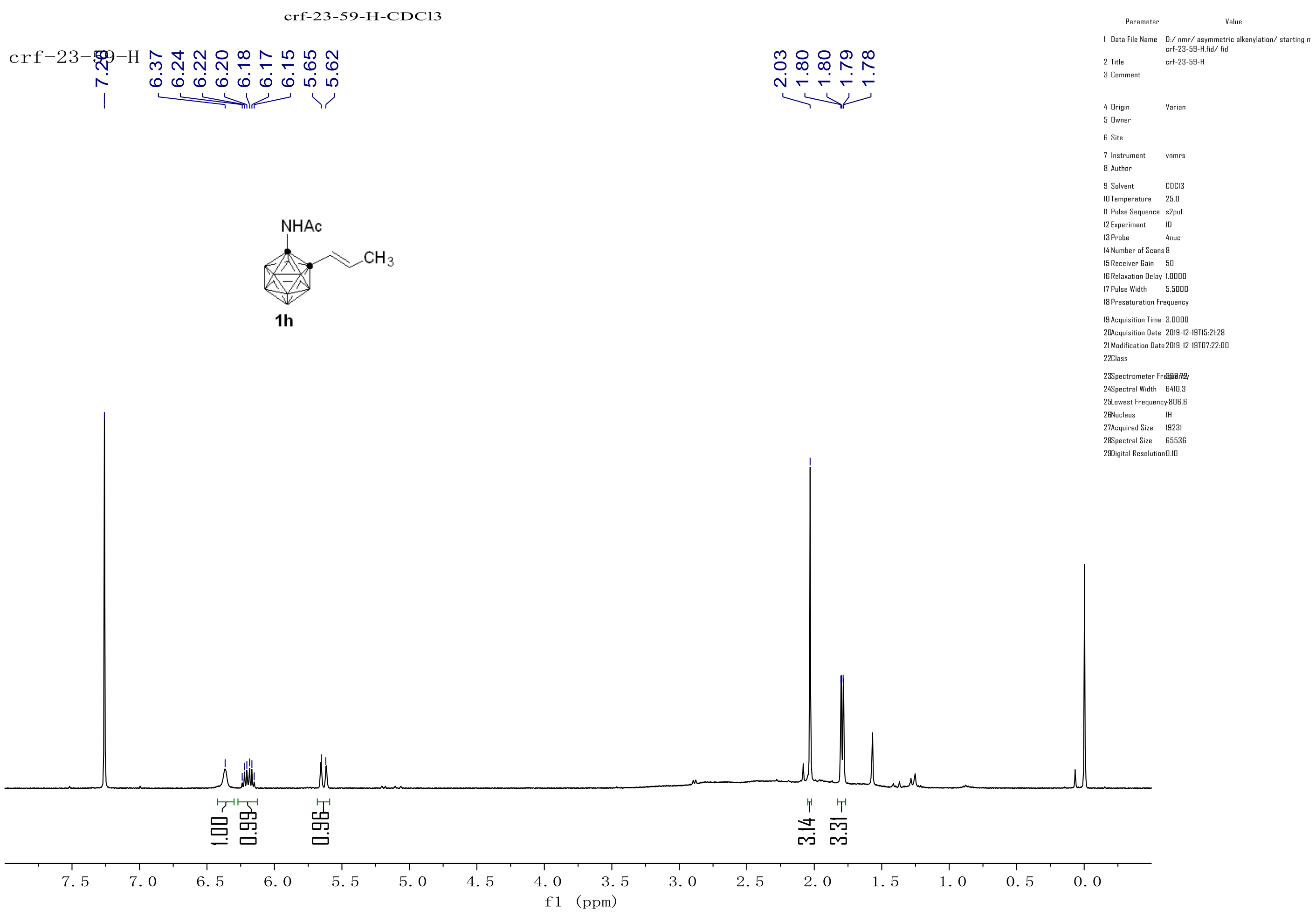

Supplementary Figure 35. $^{13}C\{^{1}H\}$ NMR of **1h**.

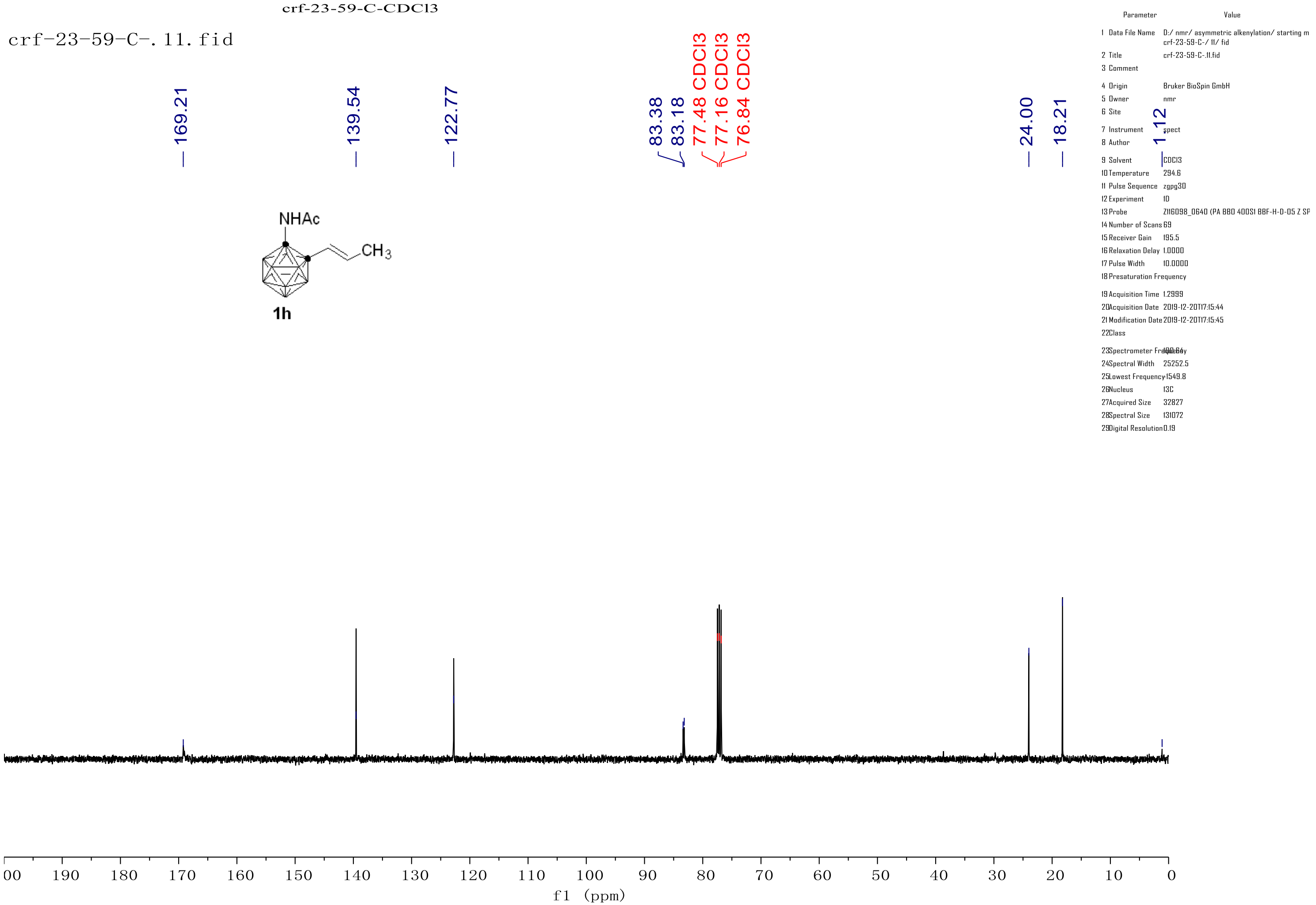

Supplementary Figure 36. $^{11}B\{^{1}H\}$ NMR of **1h**.

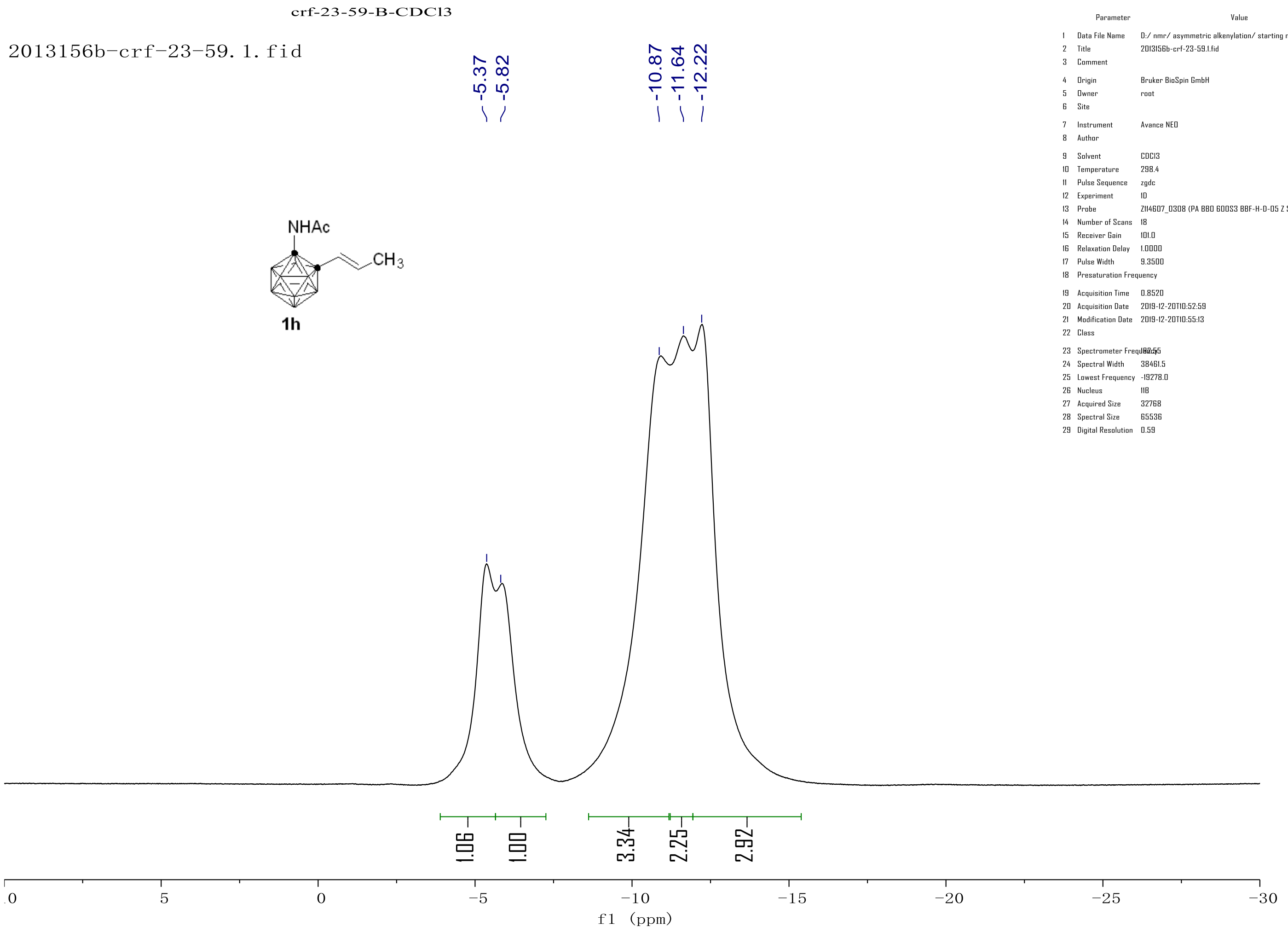

	Parameter	Value
1	Data File Name	D:/ nmr/ asymmetric alkenylation/ starting materials/ 23-59/
2	Title	2013156b-crf-23-59.1.fid
3	Comment	
4	Origin	Bruker BioSpin GmbH
5	Owner	root
6	Site	
7	Instrument	Avance NEO
8	Author	
9	Solvent	CDCl3
10	Temperature	298.4
11	Pulse Sequence	zgdc
12	Experiment	1D
13	Probe	Z114607_0308 (PA BBO 600S3 BBF-H-D-05 Z SP)
14	Number of Scans	18
15	Receiver Gain	101.0
16	Relaxation Delay	1.0000
17	Pulse Width	9.3500
18	Presaturation Frequency	
19	Acquisition Time	0.8520
20	Acquisition Date	2019-12-20T10:52:59
21	Modification Date	2019-12-20T10:55:13
22	Class	
23	Spectrometer Frequency	192.55
24	Spectral Width	38461.5
25	Lowest Frequency	-19278.0
26	Nucleus	11B
27	Acquired Size	32768
28	Spectral Size	65536
29	Digital Resolution	0.59

Supplementary Figure 37. ^{1}H NMR of **1i**.

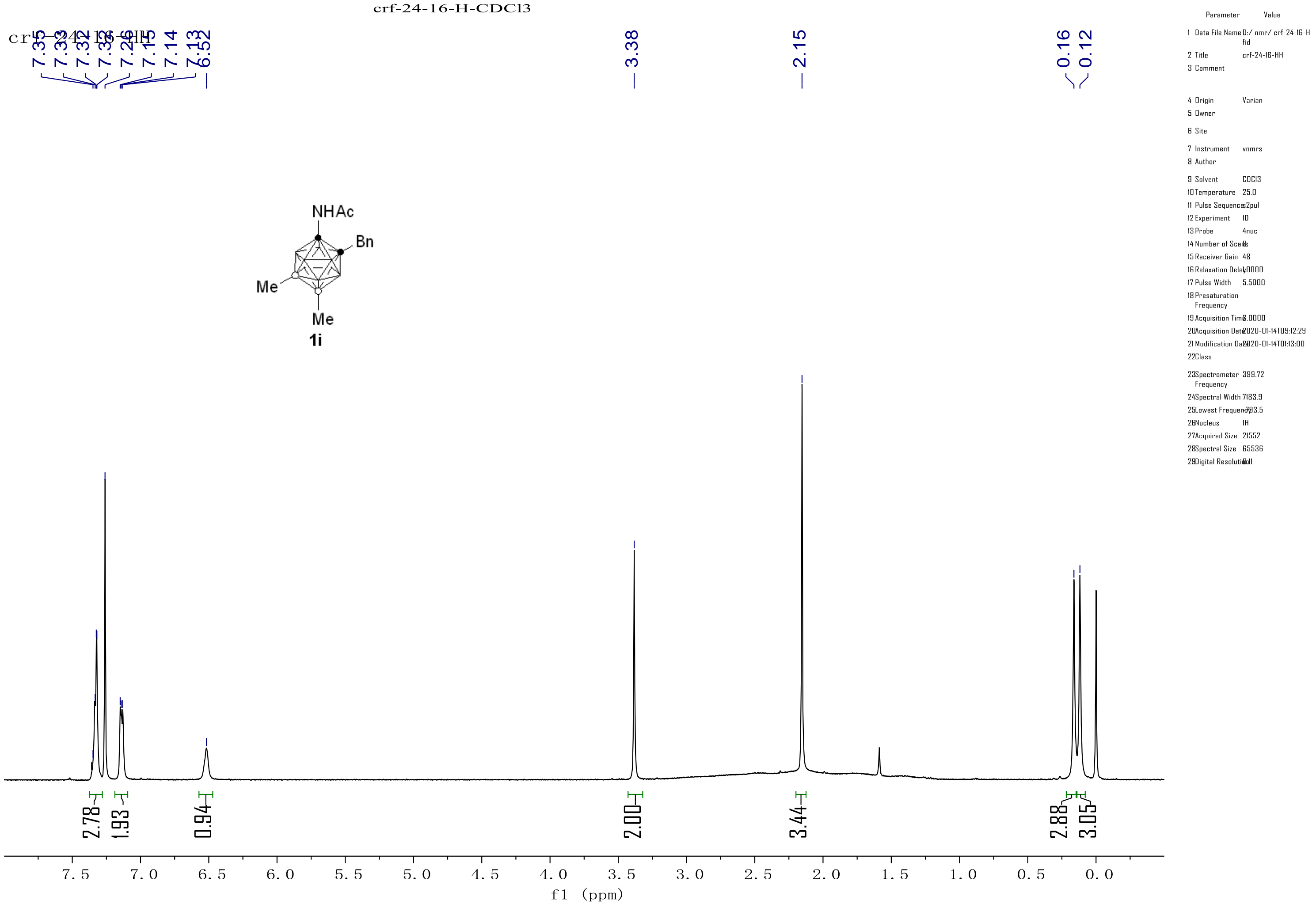

Supplementary Figure 38. $^{13}C\{^{1}H\}$ NMR of **1i**.

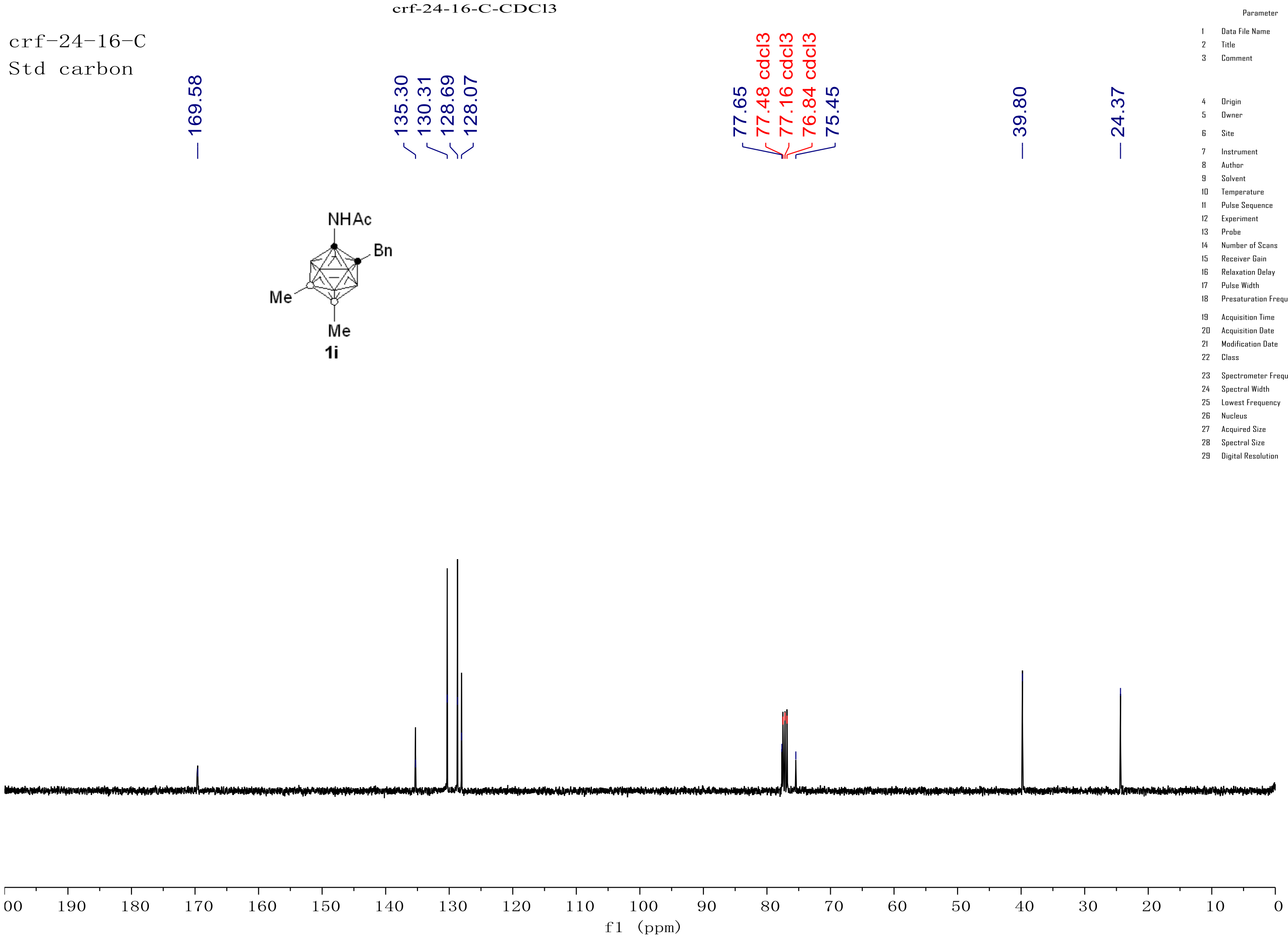

	Parameter	Value
1	Data File Name	D:/ nmr/ crf-24-16-C.fid/
2	Title	crf-24-16-C
3	Comment	Std carbon
4	Origin	Varian
5	Owner	
6	Site	
7	Instrument	vnmrs
8	Author	omcl
9	Solvent	cdcl3
10	Temperature	3.0
11	Pulse Sequence	s2pul
12	Experiment	1D
13	Probe	ATB
14	Number of Scans	40
15	Receiver Gain	28
16	Relaxation Delay	1.0000
17	Pulse Width	5.7000
18	Presaturation Frequency	
19	Acquisition Time	1.3000
20	Acquisition Date	2020-01-14T10:53:42
21	Modification Date	2020-01-14T02:55:00
22	Class	
23	Spectrometer Frequency	100.60
24	Spectral Width	24509.8
25	Lowest Frequency	-1682.2
26	Nucleus	13C
27	Acquired Size	31863
28	Spectral Size	65536
29	Digital Resolution	0.37

Supplementary Figure 39. $^{11}B\{^{1}H\}$ NMR of **1i**.

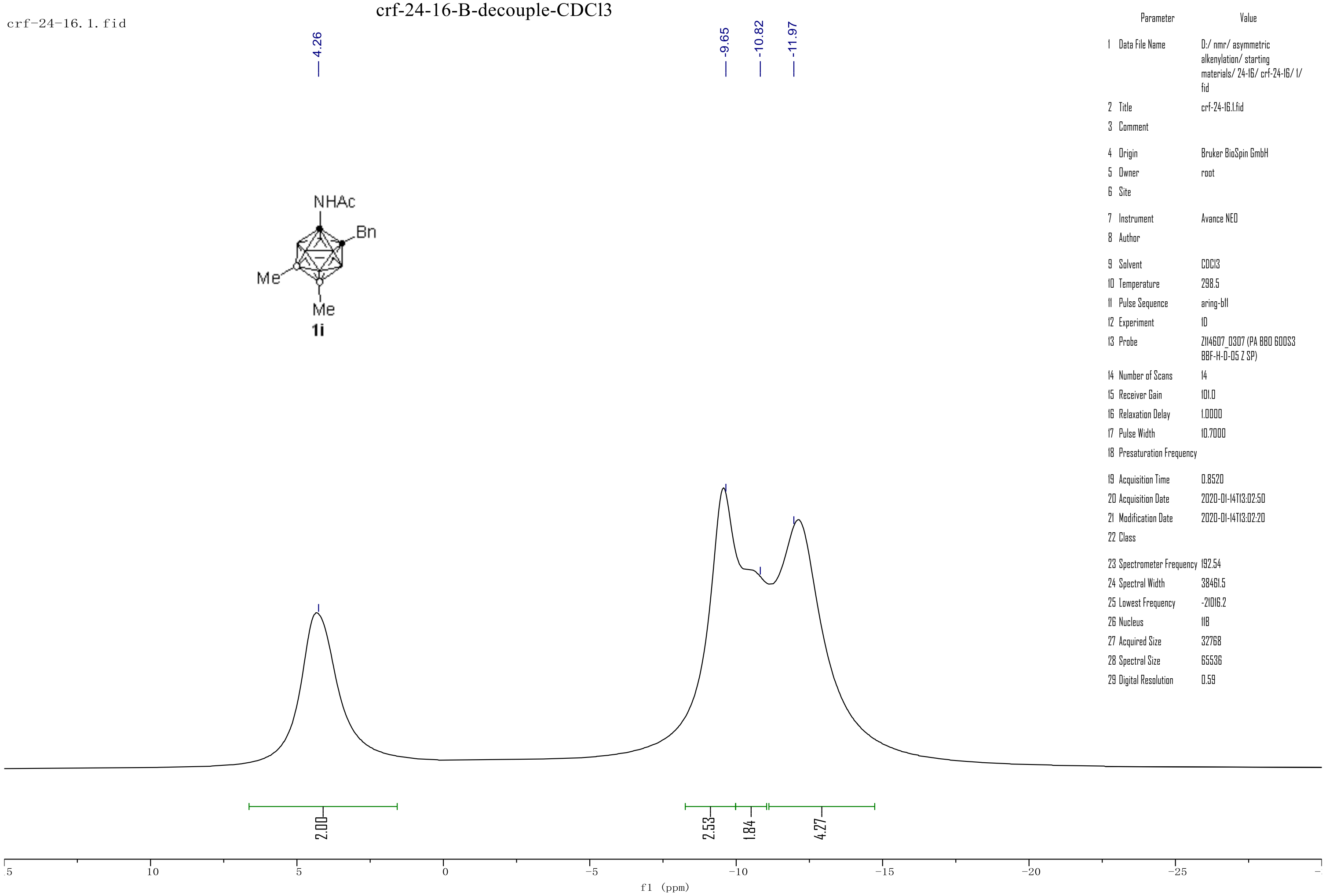

	Parameter	Value
1	Data File Name	D:/ nmr/ asymmetric alkenylation/ starting materials/ 24-16/ crf-24-16/ 1/ fid
2	Title	crf-24-16.1.fid
3	Comment	
4	Origin	Bruker BioSpin GmbH
5	Owner	root
6	Site	
7	Instrument	Avance NEO
8	Author	
9	Solvent	CDCl3
10	Temperature	298.5
11	Pulse Sequence	aring-b11
12	Experiment	1D
13	Probe	Z114607_0307 (PA BBO 600S3 BBF-H-D-05 Z SP)
14	Number of Scans	14
15	Receiver Gain	101.0
16	Relaxation Delay	1.0000
17	Pulse Width	10.7000
18	Presaturation Frequency	
19	Acquisition Time	0.8520
20	Acquisition Date	2020-01-14T13:02:50
21	Modification Date	2020-01-14T13:02:20
22	Class	
23	Spectrometer Frequency	192.54
24	Spectral Width	38461.5
25	Lowest Frequency	-21016.2
26	Nucleus	11B
27	Acquired Size	32768
28	Spectral Size	65536
29	Digital Resolution	0.59

Supplementary Figure 40. ^{11}B NMR of **1i**.

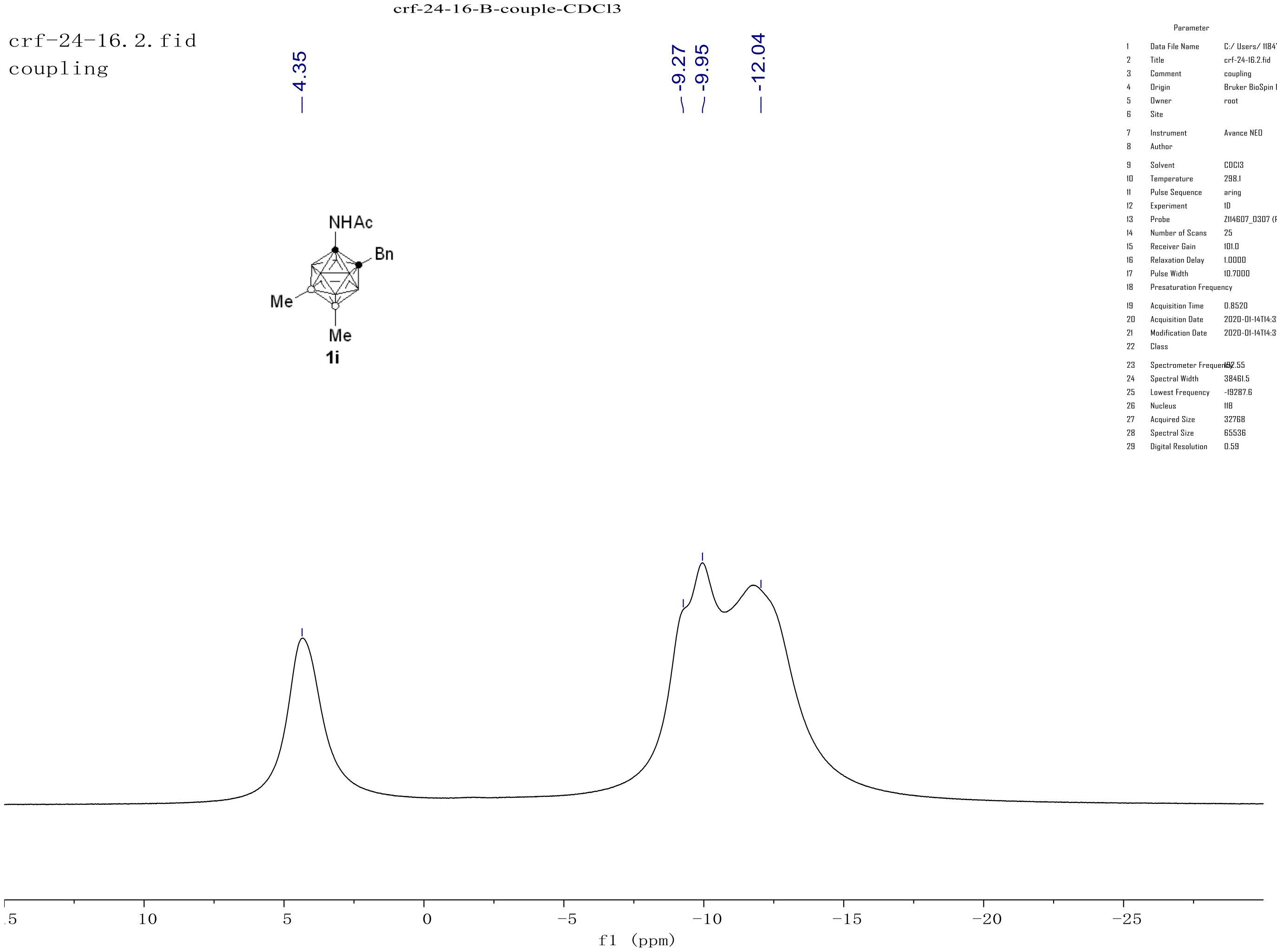

	Parameter	Value
1	Data File Name	C:/ Users/ 11847/ Downloads/ crf-24-16/ crf
2	Title	crf-24-16.2.fid
3	Comment	coupling
4	Origin	Bruker BioSpin GmbH
5	Owner	root
6	Site	
7	Instrument	Avance NEO
8	Author	
9	Solvent	CDCl3
10	Temperature	298.1
11	Pulse Sequence	aring
12	Experiment	1D
13	Probe	Z114607_0307 (PA BBO 600S3 BBF-H-D-05 Z S
14	Number of Scans	25
15	Receiver Gain	101.0
16	Relaxation Delay	1.0000
17	Pulse Width	10.7000
18	Presaturation Frequency	
19	Acquisition Time	0.8520
20	Acquisition Date	2020-01-14T14:32:17
21	Modification Date	2020-01-14T14:31:46
22	Class	
23	Spectrometer Frequency	1[illegible]2.55
24	Spectral Width	38461.5
25	Lowest Frequency	-19287.6
26	Nucleus	11B
27	Acquired Size	32768
28	Spectral Size	65536
29	Digital Resolution	0.59

Supplementary Figure 41. ^{1}H NMR of **1j**.

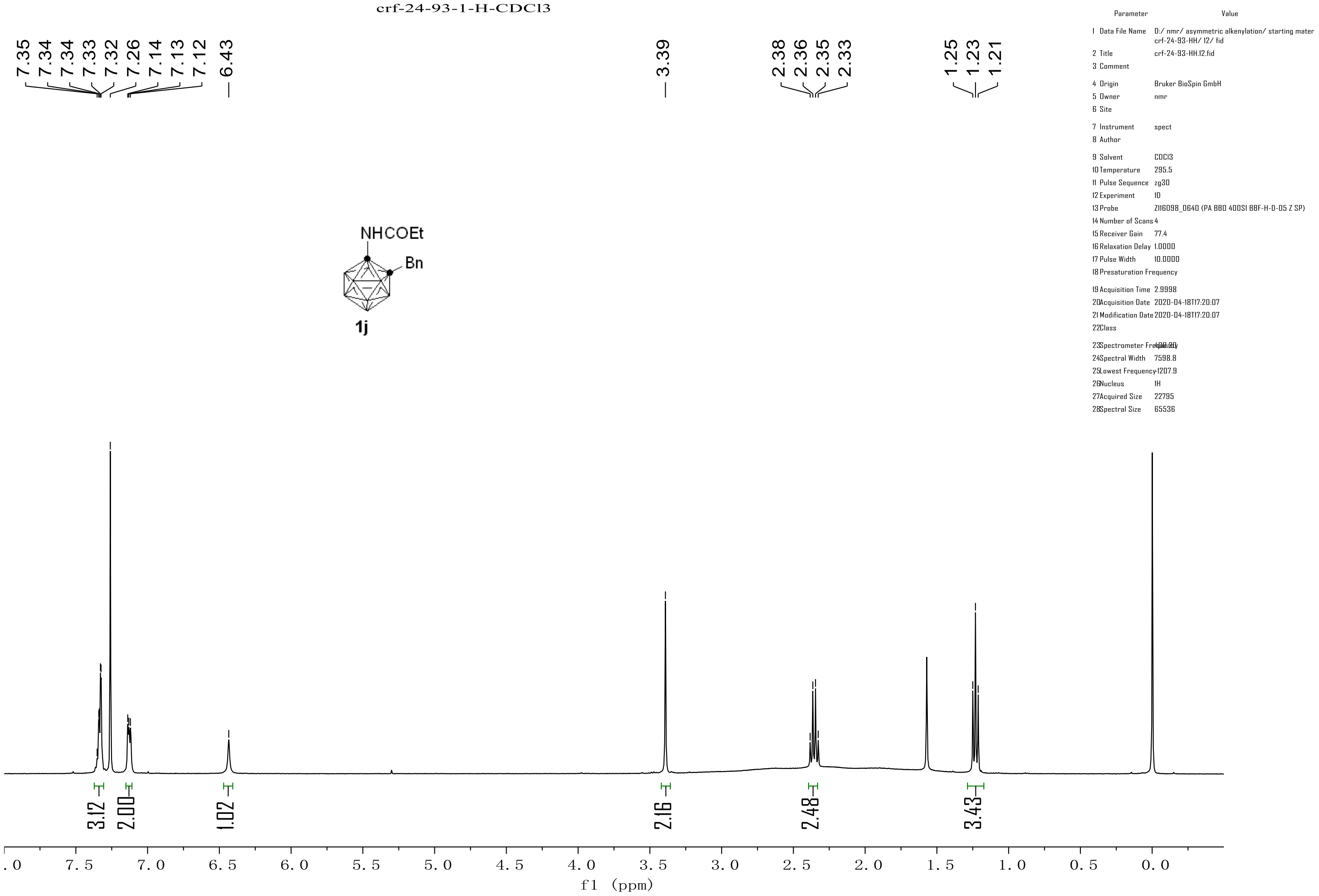

Supplementary Figure 42. $^{13}C\{^1H\}$ NMR of **1j**.

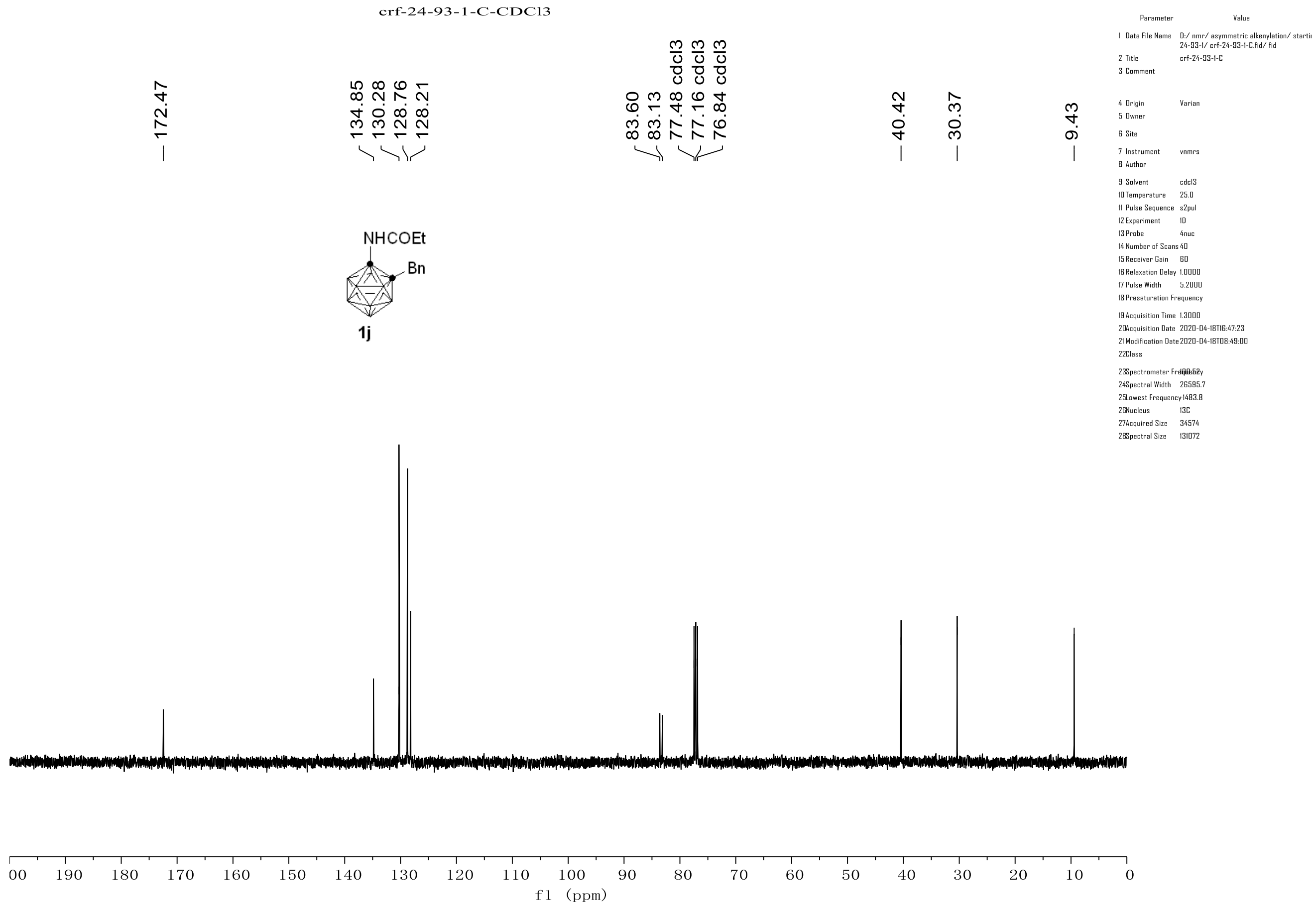

	Parameter	Value
1	Data File Name	D:/ nmr/ asymmetric alkenylation/ startir 24-93-1/ crf-24-93-1-C.fid/ fid
2	Title	crf-24-93-1-C
3	Comment	
4	Origin	Varian
5	Owner	
6	Site	
7	Instrument	vnmrs
8	Author	
9	Solvent	cdcl3
10	Temperature	25.0
11	Pulse Sequence	s2pul
12	Experiment	1D
13	Probe	4nuc
14	Number of Scans	40
15	Receiver Gain	60
16	Relaxation Delay	1.0000
17	Pulse Width	5.2000
18	Presaturation Frequency	
19	Acquisition Time	1.3000
20	Acquisition Date	2020-04-18T16:47:23
21	Modification Date	2020-04-18T08:49:00
22	Class	
23	Spectrometer Frequency	[illegible]
24	Spectral Width	26595.7
25	Lowest Frequency	-1483.8
26	Nucleus	13C
27	Acquired Size	34574
28	Spectral Size	131072

Supplementary Figure 43. $^{11}B\{^{1}H\}$ NMR of **1j**.

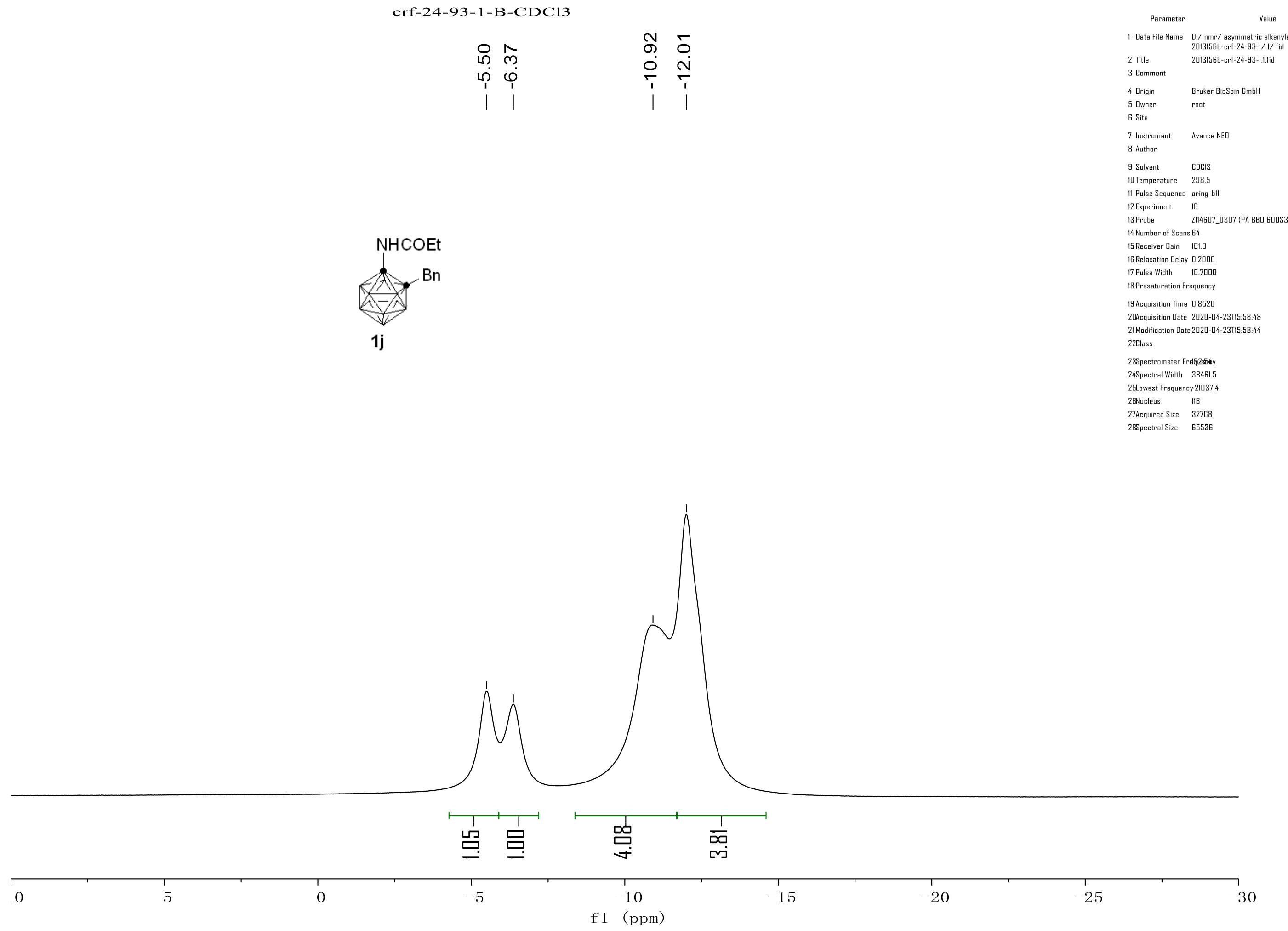

	Parameter	Value
1	Data File Name	D:/ nmr/ asymmetric alkenylation/ starting mate 2013156b-crf-24-93-1/ 1/ fid
2	Title	2013156b-crf-24-93-1.1.fid
3	Comment	
4	Origin	Bruker BioSpin GmbH
5	Owner	root
6	Site	
7	Instrument	Avance NEO
8	Author	
9	Solvent	CDCl3
10	Temperature	298.5
11	Pulse Sequence	aring-b11
12	Experiment	1D
13	Probe	Z114607_0307 (PA BBO 600S3 BBF-H-D-05 Z SP)
14	Number of Scans	64
15	Receiver Gain	101.0
16	Relaxation Delay	0.2000
17	Pulse Width	10.7000
18	Presaturation Frequency	
19	Acquisition Time	0.8520
20	Acquisition Date	2020-04-23T15:58:48
21	Modification Date	2020-04-23T15:58:44
22	Class	
23	Spectrometer Frequency	192.54
24	Spectral Width	38461.5
25	Lowest Frequency	-21037.4
26	Nucleus	11B
27	Acquired Size	32768
28	Spectral Size	65536

Supplementary Figure 44. ^{1}H NMR of **1k**.

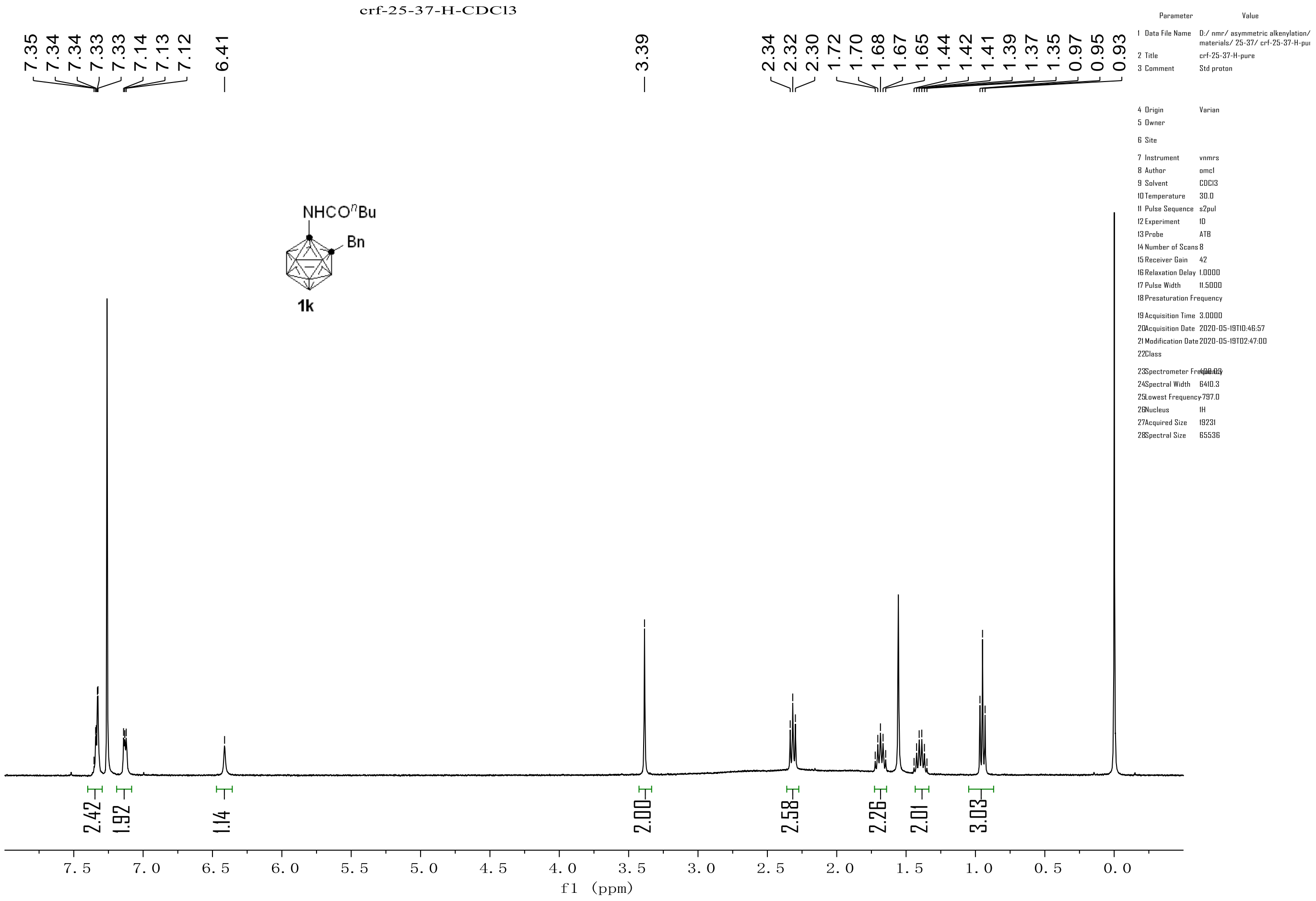

Supplementary Figure 45. $^{13}C\{^{1}H\}$ NMR of **1k**.

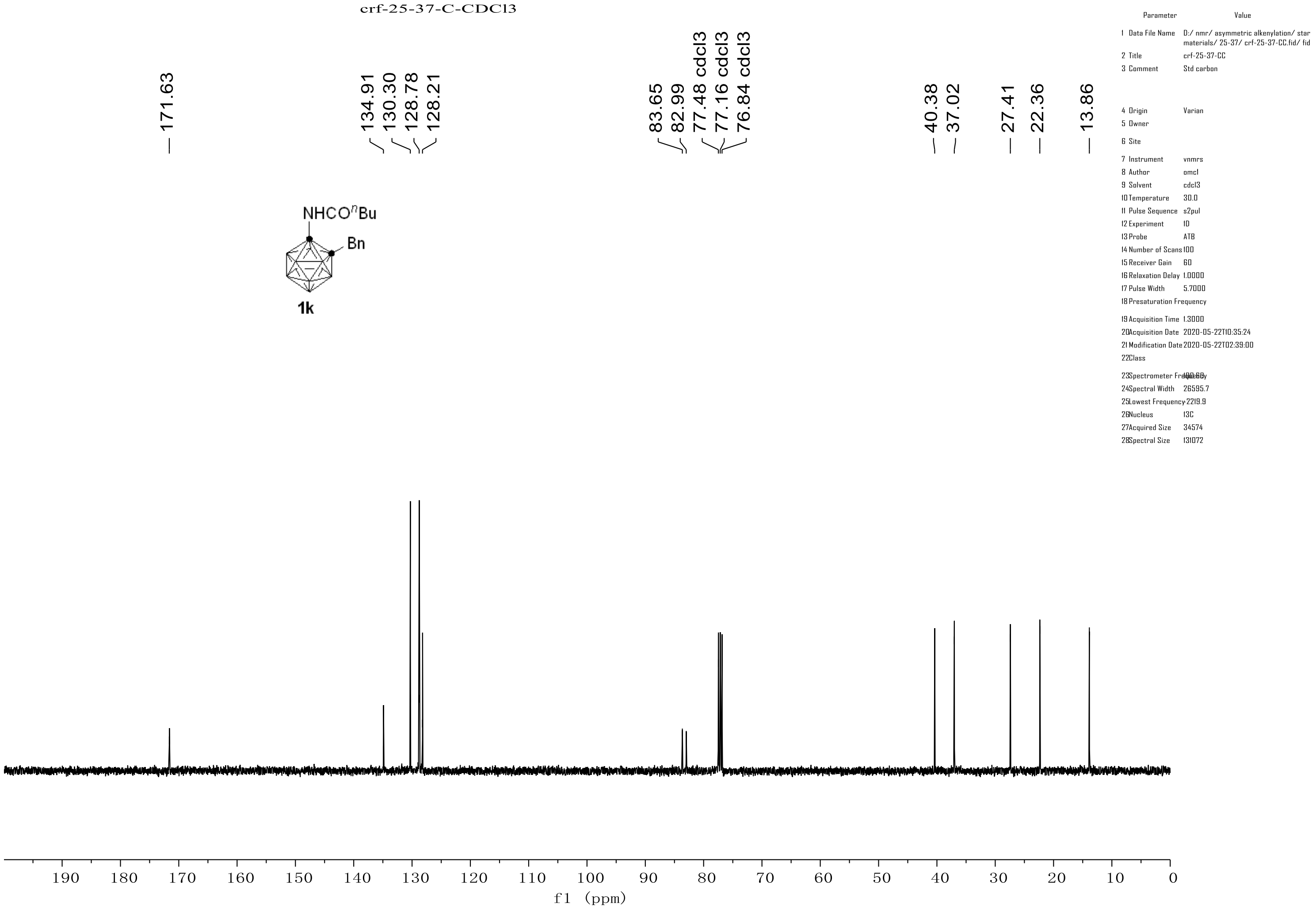

Supplementary Figure 46. $^{11}B\{^{1}H\}$ NMR of **1k**.

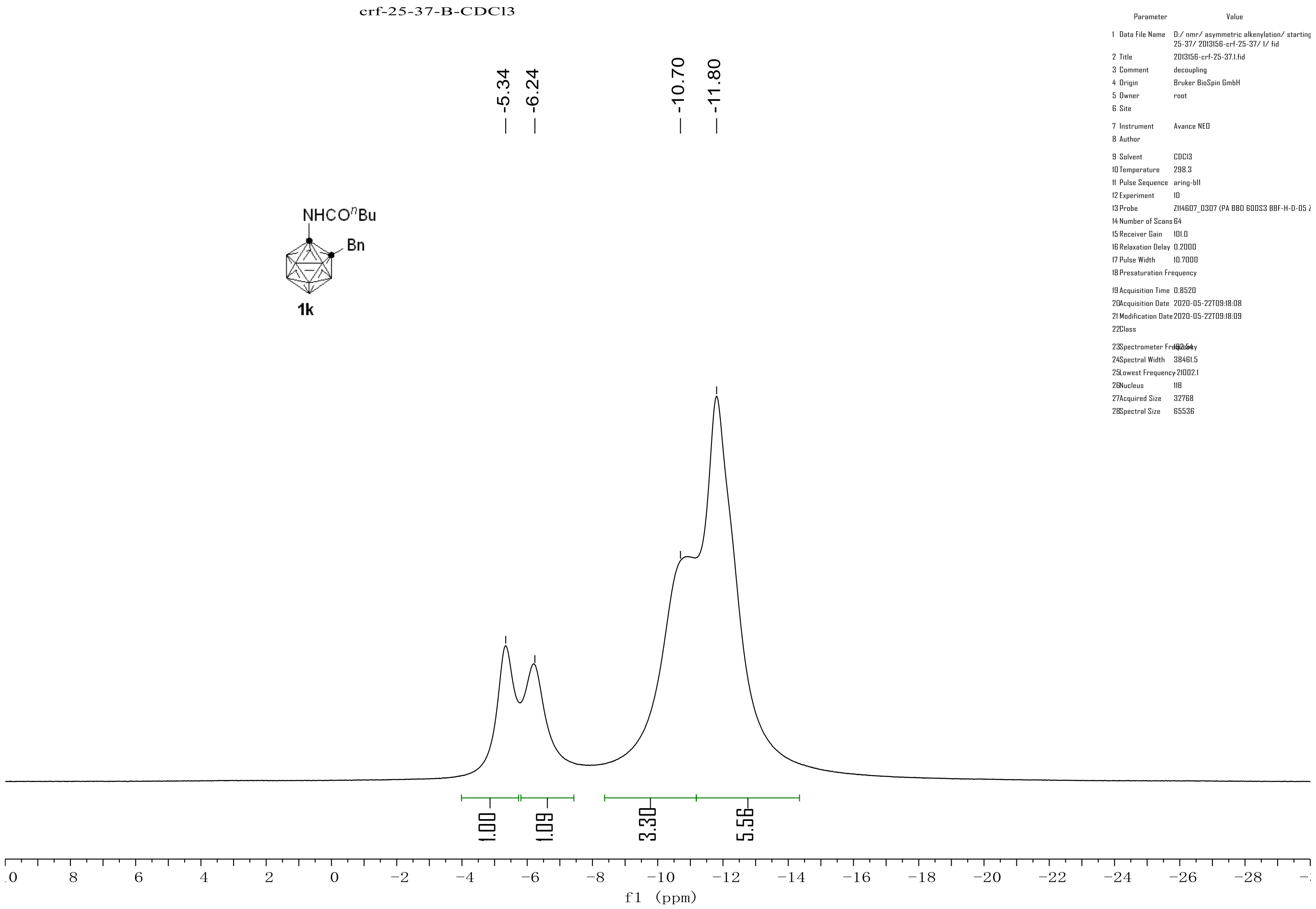

Supplementary Figure 47. ^{1}H NMR of **1l**.

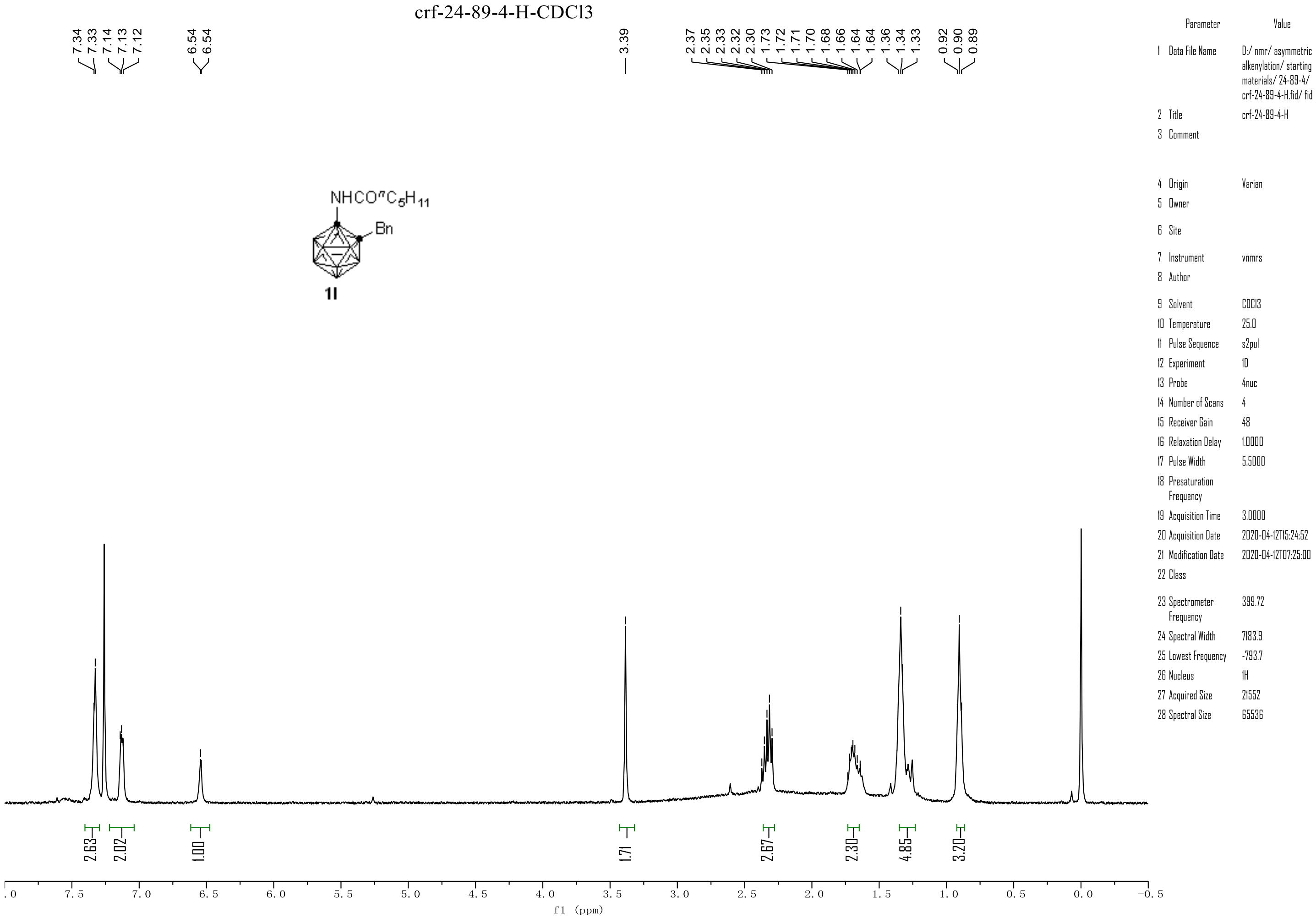

Parameter		Value
1	Data File Name	D:/ nmr/ asymmetric alkenylation/ starting materials/ 24-89-4/ crf-24-89-4-H.fid/ fid
2	Title	crf-24-89-4-H
3	Comment	
4	Origin	Varian
5	Owner	
6	Site	
7	Instrument	vnmrs
8	Author	
9	Solvent	CDCl3
10	Temperature	25.0
11	Pulse Sequence	s2pul
12	Experiment	1D
13	Probe	4nuc
14	Number of Scans	4
15	Receiver Gain	48
16	Relaxation Delay	1.0000
17	Pulse Width	5.5000
18	Presaturation Frequency	
19	Acquisition Time	3.0000
20	Acquisition Date	2020-04-12T15:24:52
21	Modification Date	2020-04-12T07:25:00
22	Class	
23	Spectrometer Frequency	399.72
24	Spectral Width	7183.9
25	Lowest Frequency	-793.7
26	Nucleus	1H
27	Acquired Size	21552
28	Spectral Size	65536

Supplementary Figure 48. $^{13}C\{^{1}H\}$ NMR of **1l**.

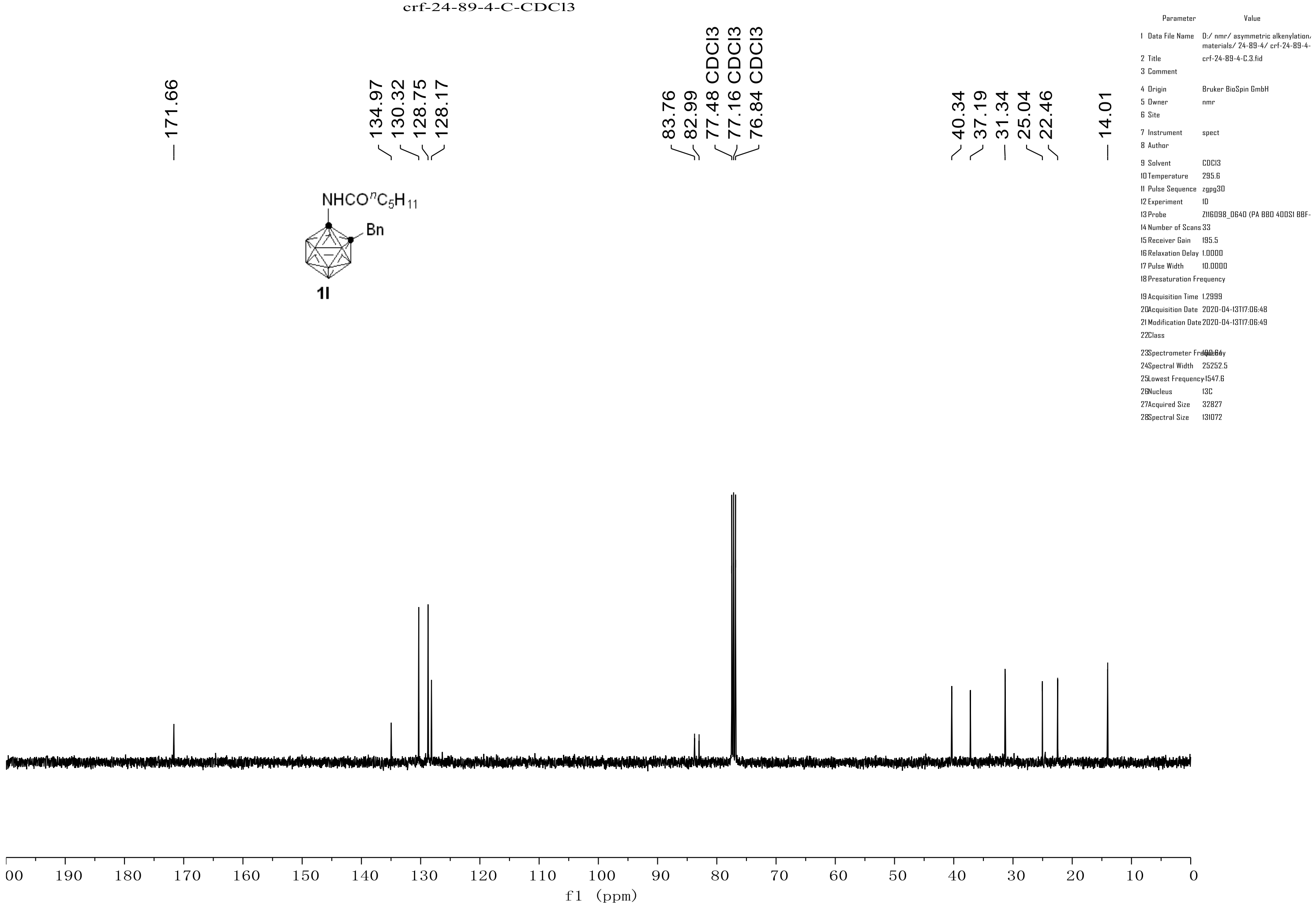

Supplementary Figure 49. $^{11}B\{^{1}H\}$ NMR of **1l**.

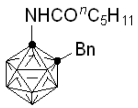

1l

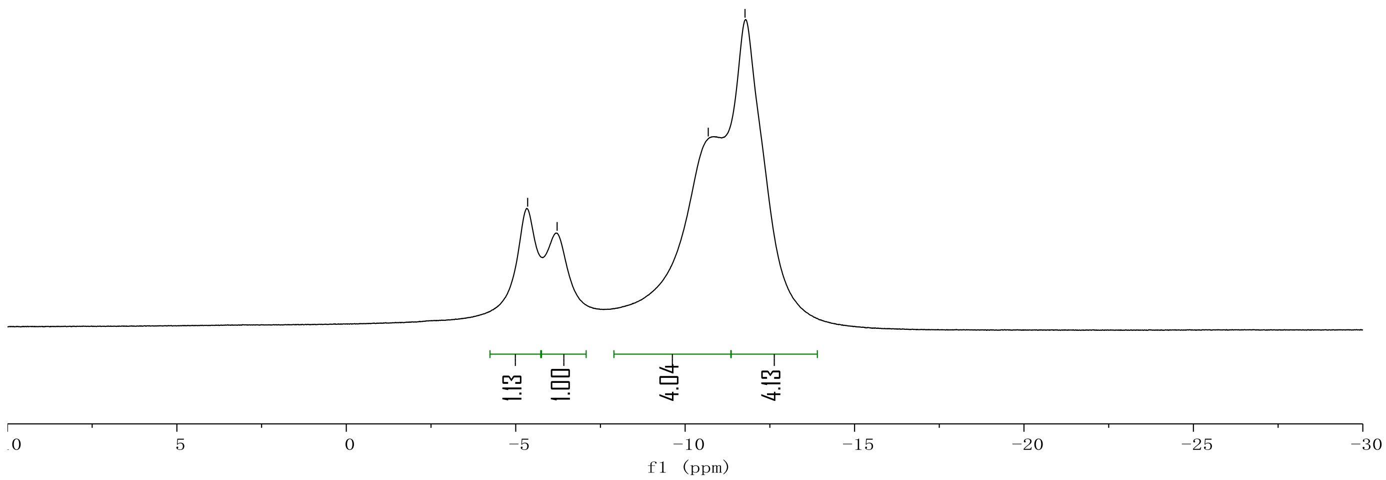

Supplementary Figure 50. ^{1}H NMR of **1m**.

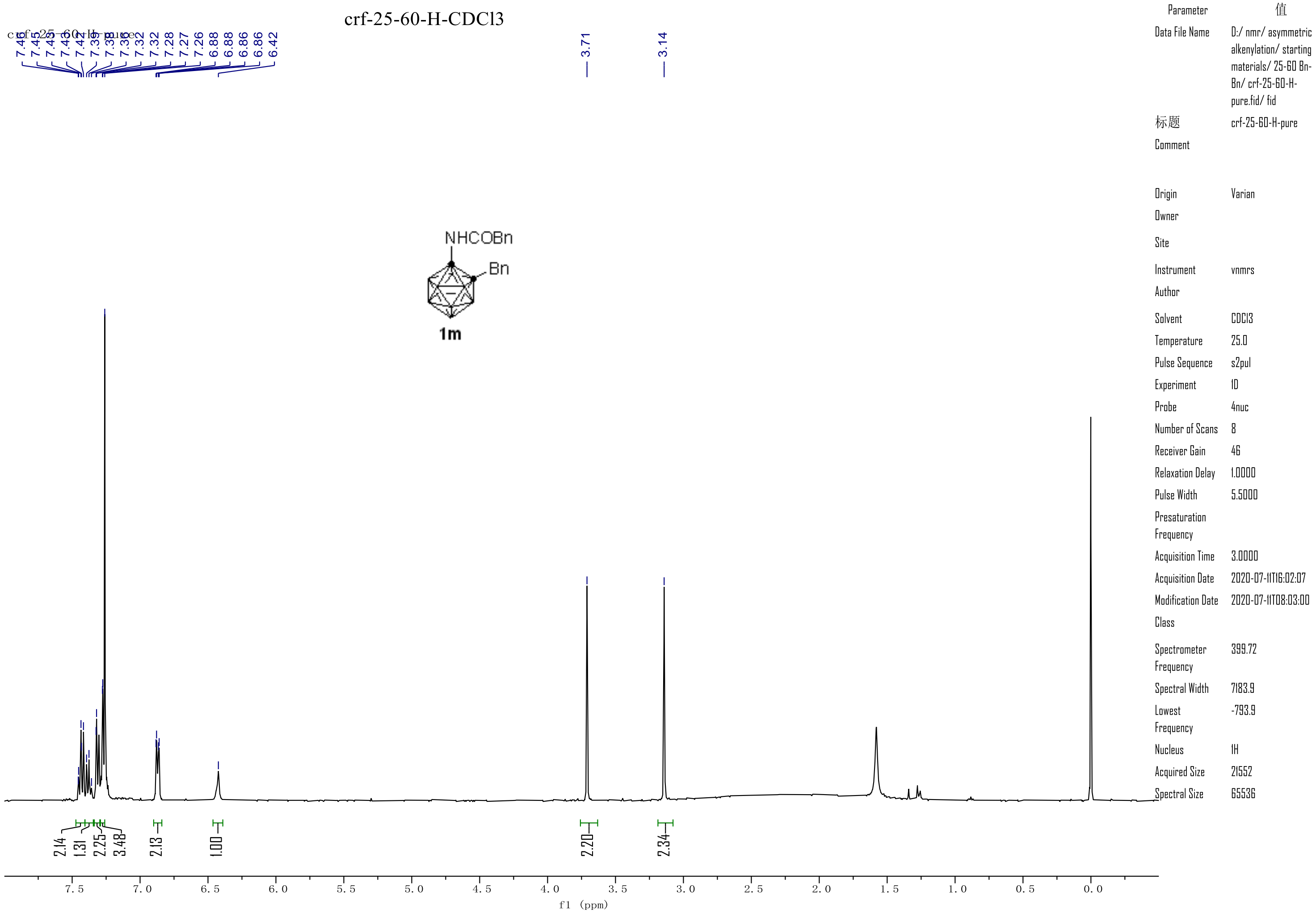

Supplementary Figure 51. $^{13}C\{^{1}H\}$ NMR of **1m**.

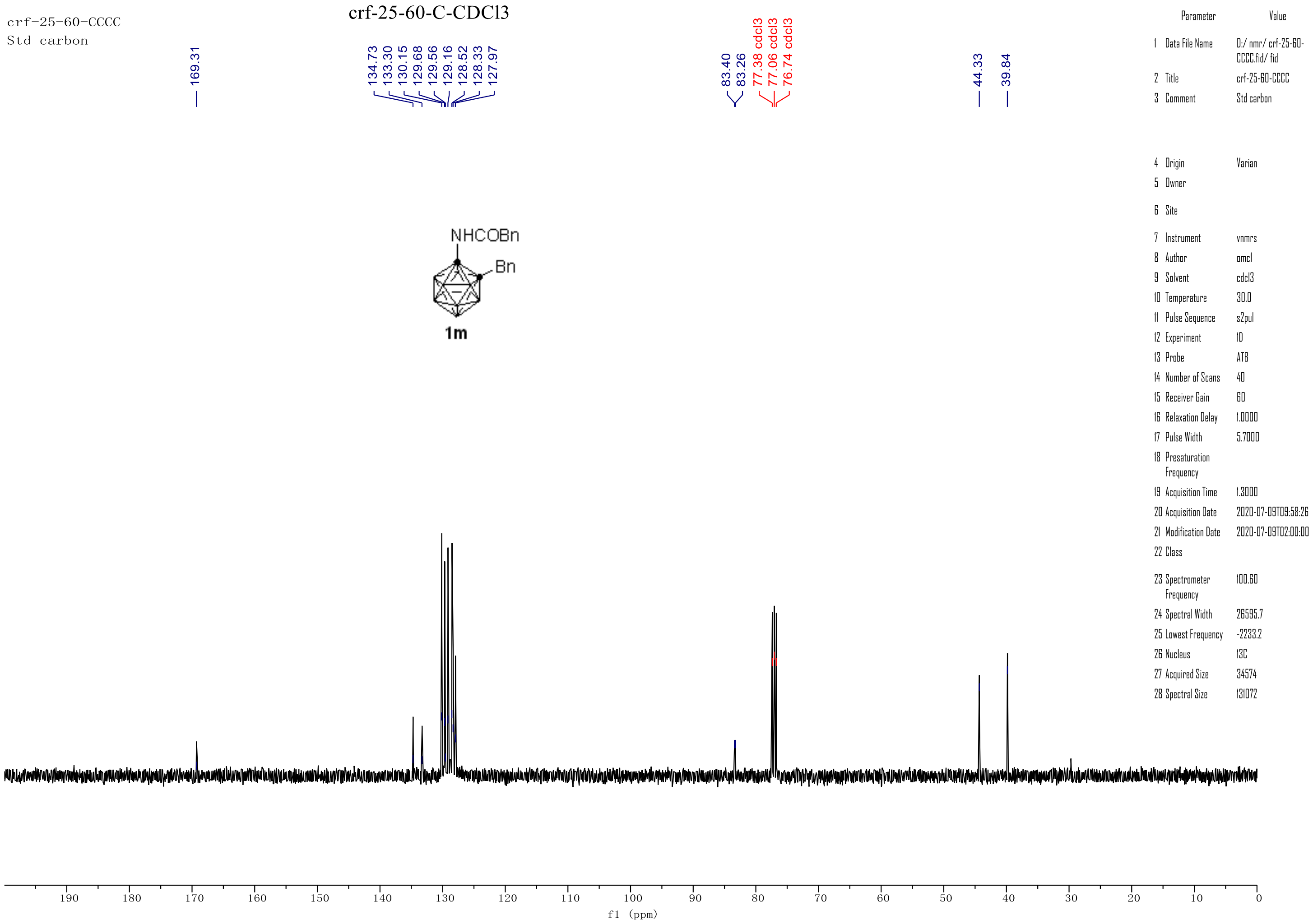

	Parameter	Value
1	Data File Name	D:/ nmr/ crf-25-60-CCCC.fid/ fid
2	Title	crf-25-60-CCCC
3	Comment	Std carbon
4	Origin	Varian
5	Owner	
6	Site	
7	Instrument	vnmrs
8	Author	omcl
9	Solvent	cdcl3
10	Temperature	30.0
11	Pulse Sequence	s2pul
12	Experiment	1D
13	Probe	ATB
14	Number of Scans	40
15	Receiver Gain	60
16	Relaxation Delay	1.0000
17	Pulse Width	5.7000
18	Presaturation Frequency	
19	Acquisition Time	1.3000
20	Acquisition Date	2020-07-09T09:58:26
21	Modification Date	2020-07-09T02:00:00
22	Class	
23	Spectrometer Frequency	100.60
24	Spectral Width	26595.7
25	Lowest Frequency	-2233.2
26	Nucleus	13C
27	Acquired Size	34574
28	Spectral Size	131072

Supplementary Figure 52. $^{11}B\{^{1}H\}$ NMR of **1m**.

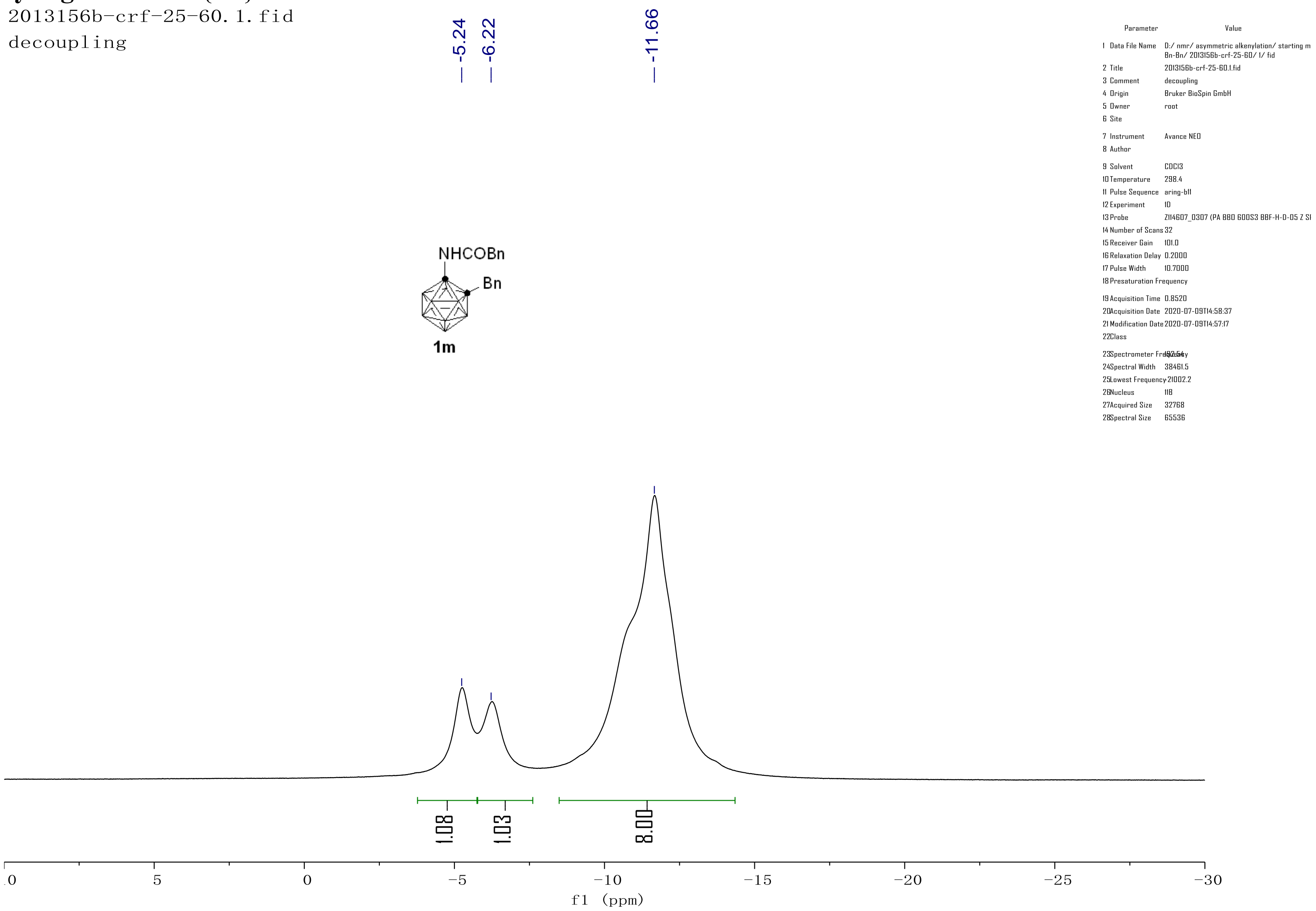

Supplementary Figure 53. ^{1}H NMR of **1n**.

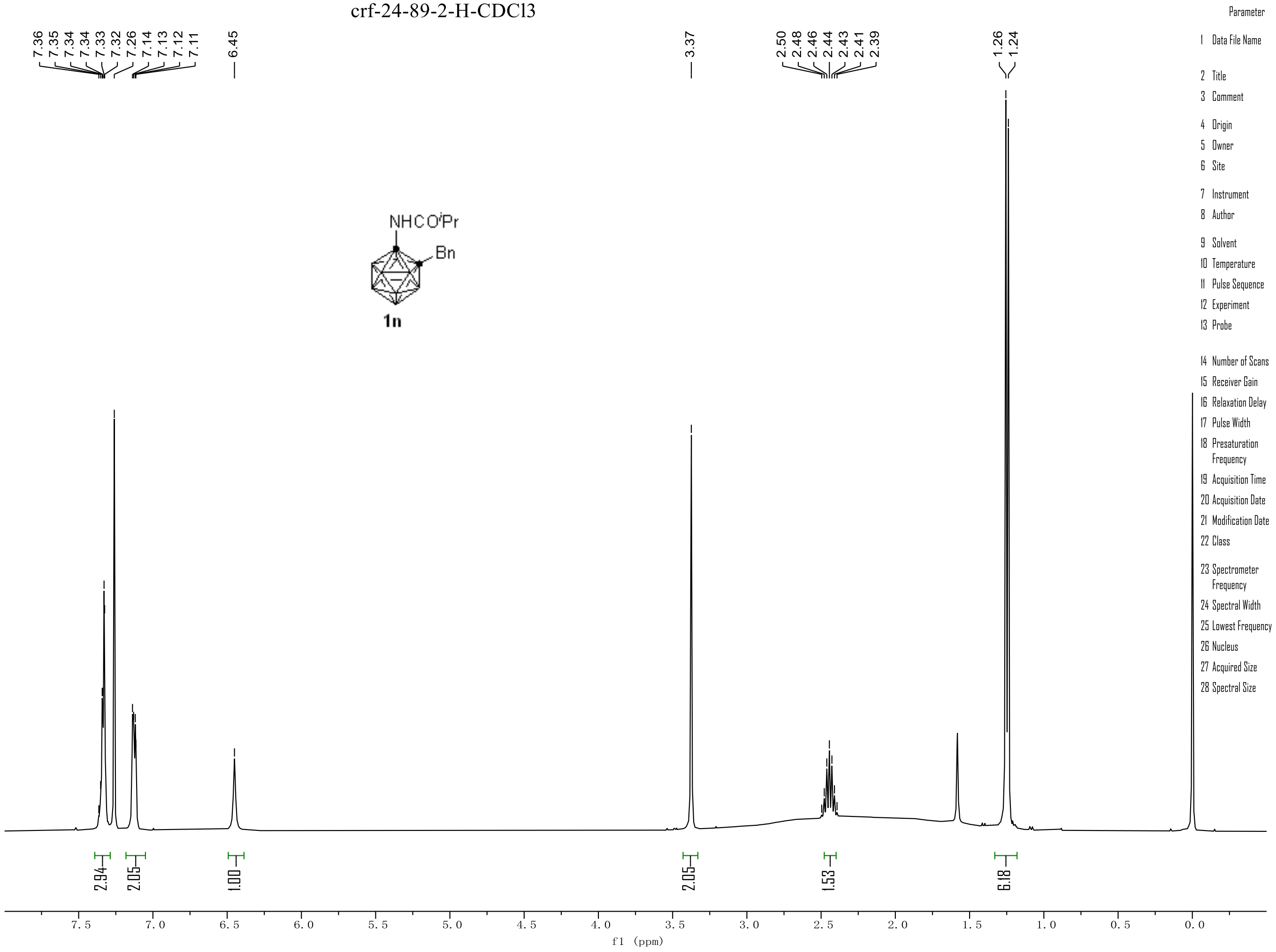

	Parameter	Value
1	Data File Name	D:/ nmr/ crf-24-89-3-H/ 4/ fid
2	Title	crf-24-89-3-H.4.fid
3	Comment	
4	Origin	Bruker BioSpin GmbH
5	Owner	nmr
6	Site	
7	Instrument	spect
8	Author	
9	Solvent	CDCl3
10	Temperature	294.6
11	Pulse Sequence	zg30
12	Experiment	1D
13	Probe	Z116098_0640 (PA BBO 400S1 BBF-H-D-05 Z SP)
14	Number of Scans	8
15	Receiver Gain	77.4
16	Relaxation Delay	1.0000
17	Pulse Width	10.0000
18	Presaturation Frequency	
19	Acquisition Time	2.9998
20	Acquisition Date	2020-04-11T17:06:44
21	Modification Date	2020-04-11T17:06:46
22	Class	
23	Spectrometer Frequency	400.20
24	Spectral Width	7598.8
25	Lowest Frequency	-1207.7
26	Nucleus	1H
27	Acquired Size	22795
28	Spectral Size	65536

Supplementary Figure 54. $^{13}C\{^1H\}$ NMR of **1n**.

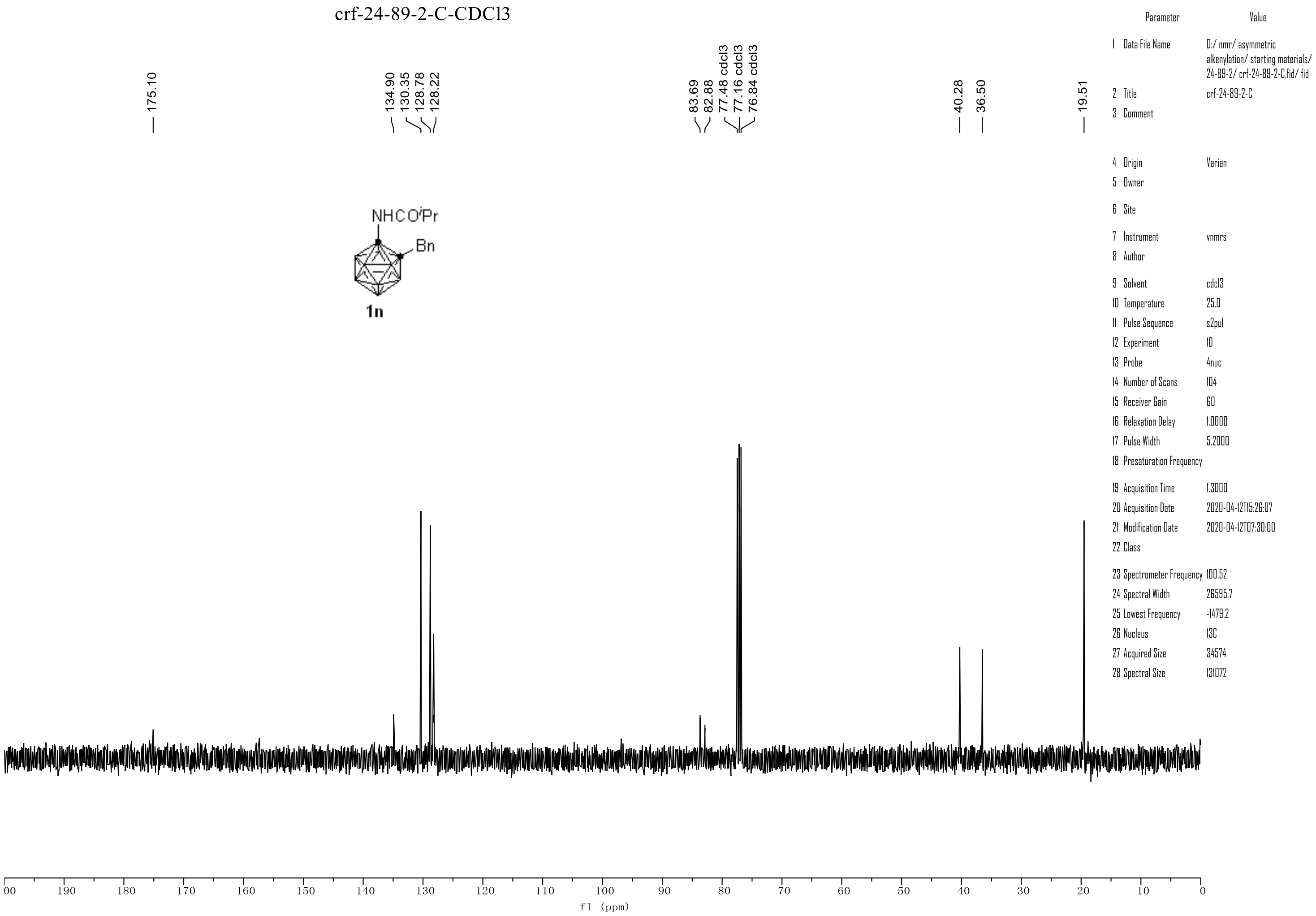

Parameter	Value
1 Data File Name	D:/ nmr/ asymmetric alkenylation/ starting materials/ 24-89-2/ crf-24-89-2-C.fid/ fid
2 Title	crf-24-89-2-C
3 Comment	
4 Origin	Varian
5 Owner	
6 Site	
7 Instrument	vnmrs
8 Author	
9 Solvent	cdcl3
10 Temperature	25.0
11 Pulse Sequence	s2pul
12 Experiment	1D
13 Probe	4nuc
14 Number of Scans	104
15 Receiver Gain	60
16 Relaxation Delay	1.0000
17 Pulse Width	5.2000
18 Presaturation Frequency	
19 Acquisition Time	1.3000
20 Acquisition Date	2020-04-12T15:26:07
21 Modification Date	2020-04-12T07:30:00
22 Class	
23 Spectrometer Frequency	100.52
24 Spectral Width	26595.7
25 Lowest Frequency	-1479.2
26 Nucleus	13C
27 Acquired Size	34574
28 Spectral Size	131072

Supplementary Figure 55. $^{11}B\{^1H\}$ NMR of **1n**.

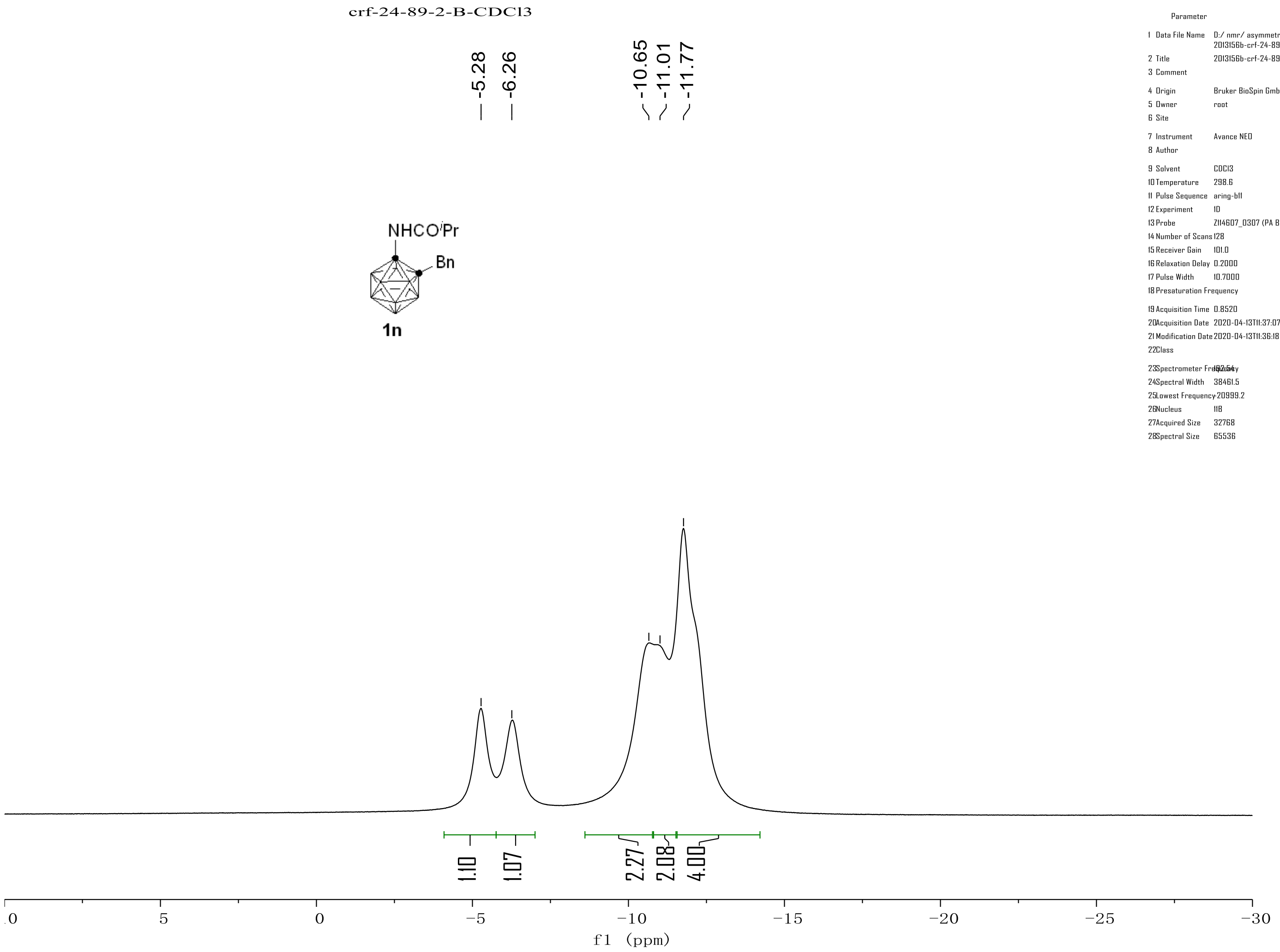

	Parameter	Value
1	Data File Name	D:/ nmr/ asymmetric alkenylation/ starting materia 2013156b-crf-24-89-2/ 1/ fid
2	Title	2013156b-crf-24-89-2.1.fid
3	Comment	
4	Origin	Bruker BioSpin GmbH
5	Owner	root
6	Site	
7	Instrument	Avance NEO
8	Author	
9	Solvent	CDCl3
10	Temperature	298.6
11	Pulse Sequence	aring-b11
12	Experiment	1D
13	Probe	Z114607_0307 (PA BBO 600S3 BBF-H-D-05 Z SP)
14	Number of Scans	128
15	Receiver Gain	101.0
16	Relaxation Delay	0.2000
17	Pulse Width	10.7000
18	Presaturation Frequency	
19	Acquisition Time	0.8520
20	Acquisition Date	2020-04-13T11:37:07
21	Modification Date	2020-04-13T11:36:18
22	Class	
23	Spectrometer Frequency	192.54
24	Spectral Width	38461.5
25	Lowest Frequency	-20999.2
26	Nucleus	11B
27	Acquired Size	32768
28	Spectral Size	65536

Supplementary Figure 56. ^{1}H NMR of **1o**.

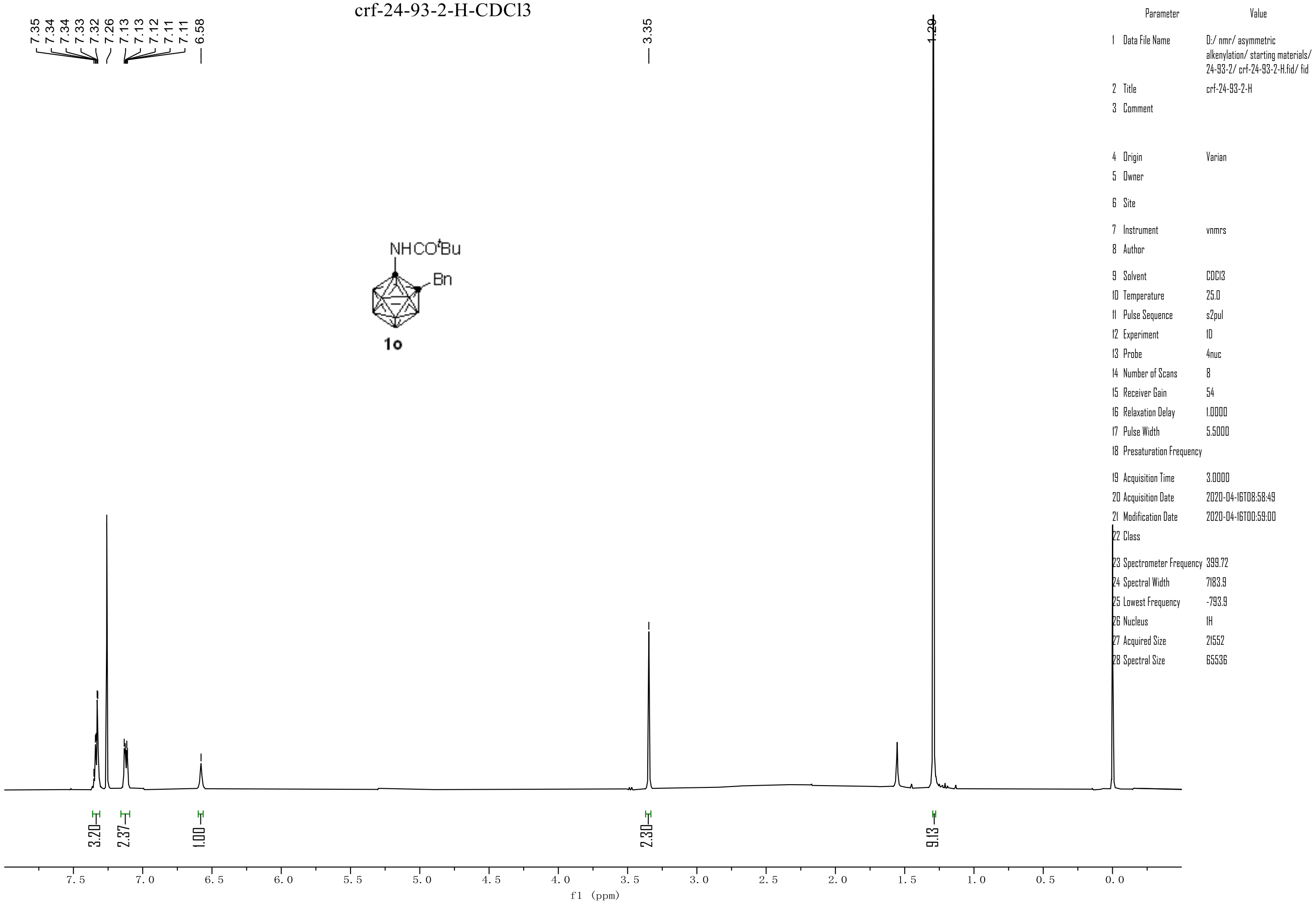

	Parameter	Value
1	Data File Name	D:/ nmr/ asymmetric alkenylation/ starting materials/ 24-93-2/ crf-24-93-2-H.fid/ fid
2	Title	crf-24-93-2-H
3	Comment	
4	Origin	Varian
5	Owner	
6	Site	
7	Instrument	vnmrs
8	Author	
9	Solvent	CDCl3
10	Temperature	25.0
11	Pulse Sequence	s2pul
12	Experiment	1D
13	Probe	4nuc
14	Number of Scans	8
15	Receiver Gain	54
16	Relaxation Delay	1.0000
17	Pulse Width	5.5000
18	Presaturation Frequency	
19	Acquisition Time	3.0000
20	Acquisition Date	2020-04-16T08:58:49
21	Modification Date	2020-04-16T00:59:00
22	Class	
23	Spectrometer Frequency	399.72
24	Spectral Width	7183.9
25	Lowest Frequency	-793.9
26	Nucleus	1H
27	Acquired Size	21552
28	Spectral Size	65536

Supplementary Figure 57. $^{13}C\{^{1}H\}$ NMR of **1o**.

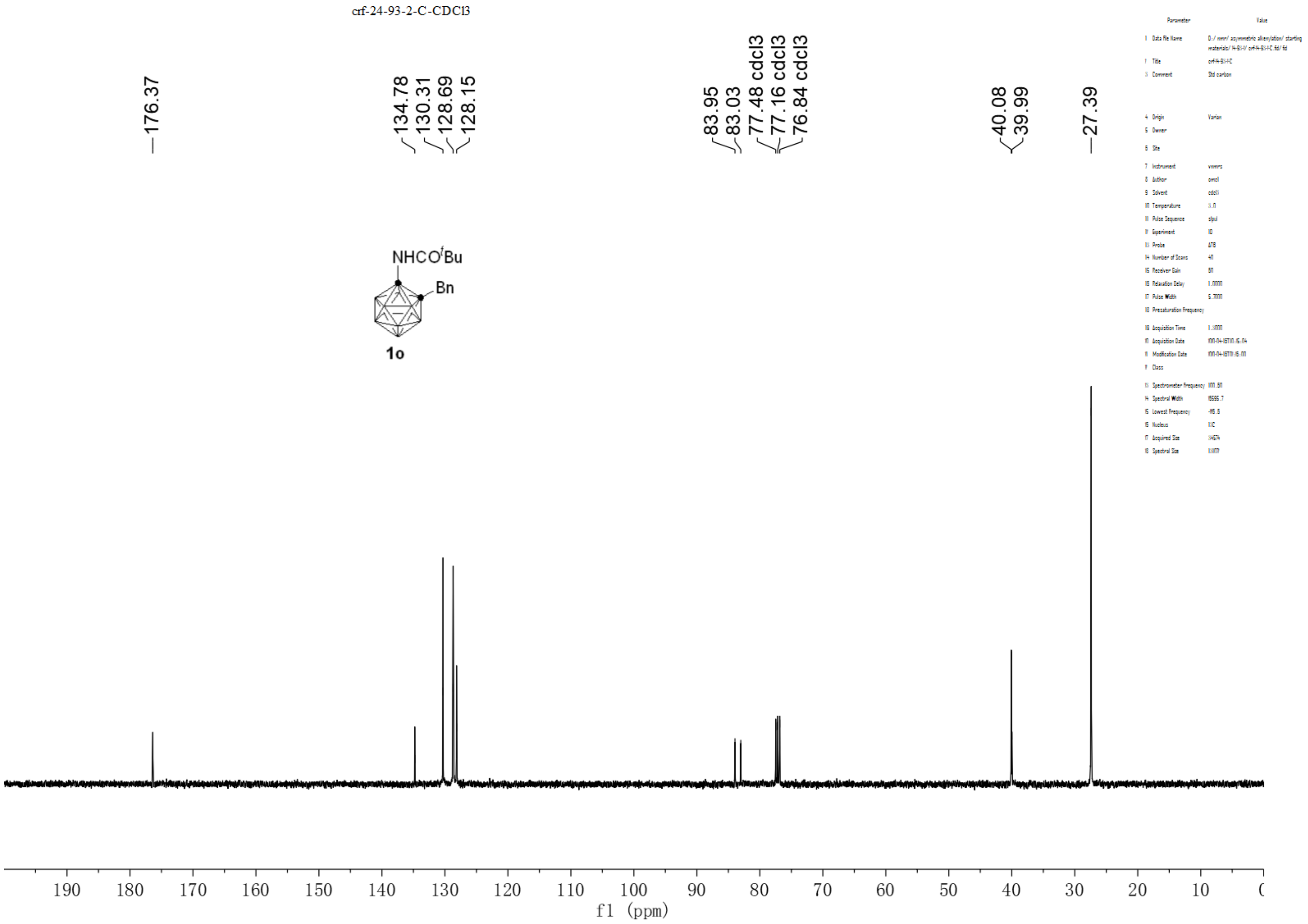

Supplementary Figure 58. $^{11}B\{^{1}H\}$ NMR of **1o**.

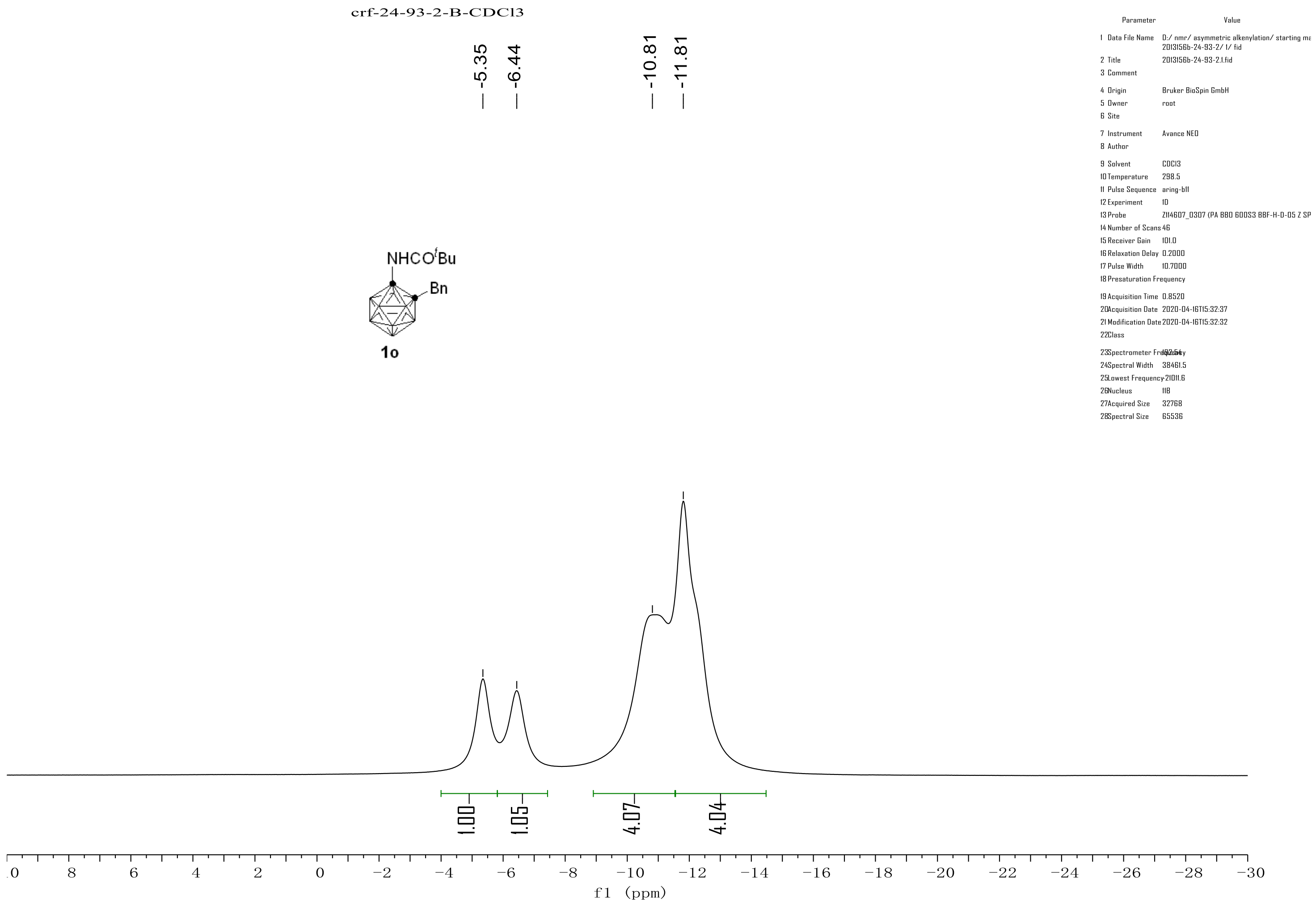

	Parameter	Value
1	Data File Name	D:/ nmr/ asymmetric alkenylation/ starting ma 2013156b-24-93-2/ 1/ fid
2	Title	2013156b-24-93-2.1.fid
3	Comment	
4	Origin	Bruker BioSpin GmbH
5	Owner	root
6	Site	
7	Instrument	Avance NEO
8	Author	
9	Solvent	CDCl3
10	Temperature	298.5
11	Pulse Sequence	aring-b11
12	Experiment	1D
13	Probe	Z114607_0307 (PA BBO 600S3 BBF-H-D-05 Z SP
14	Number of Scans	46
15	Receiver Gain	101.0
16	Relaxation Delay	0.2000
17	Pulse Width	10.7000
18	Presaturation Frequency	
19	Acquisition Time	0.8520
20	Acquisition Date	2020-04-16T15:32:37
21	Modification Date	2020-04-16T15:32:32
22	Class	
23	Spectrometer Frequency	192.54
24	Spectral Width	38461.5
25	Lowest Frequency	-21011.6
26	Nucleus	11B
27	Acquired Size	32768
28	Spectral Size	65536

Supplementary Figure 59. ^{1}H NMR of **1p**.

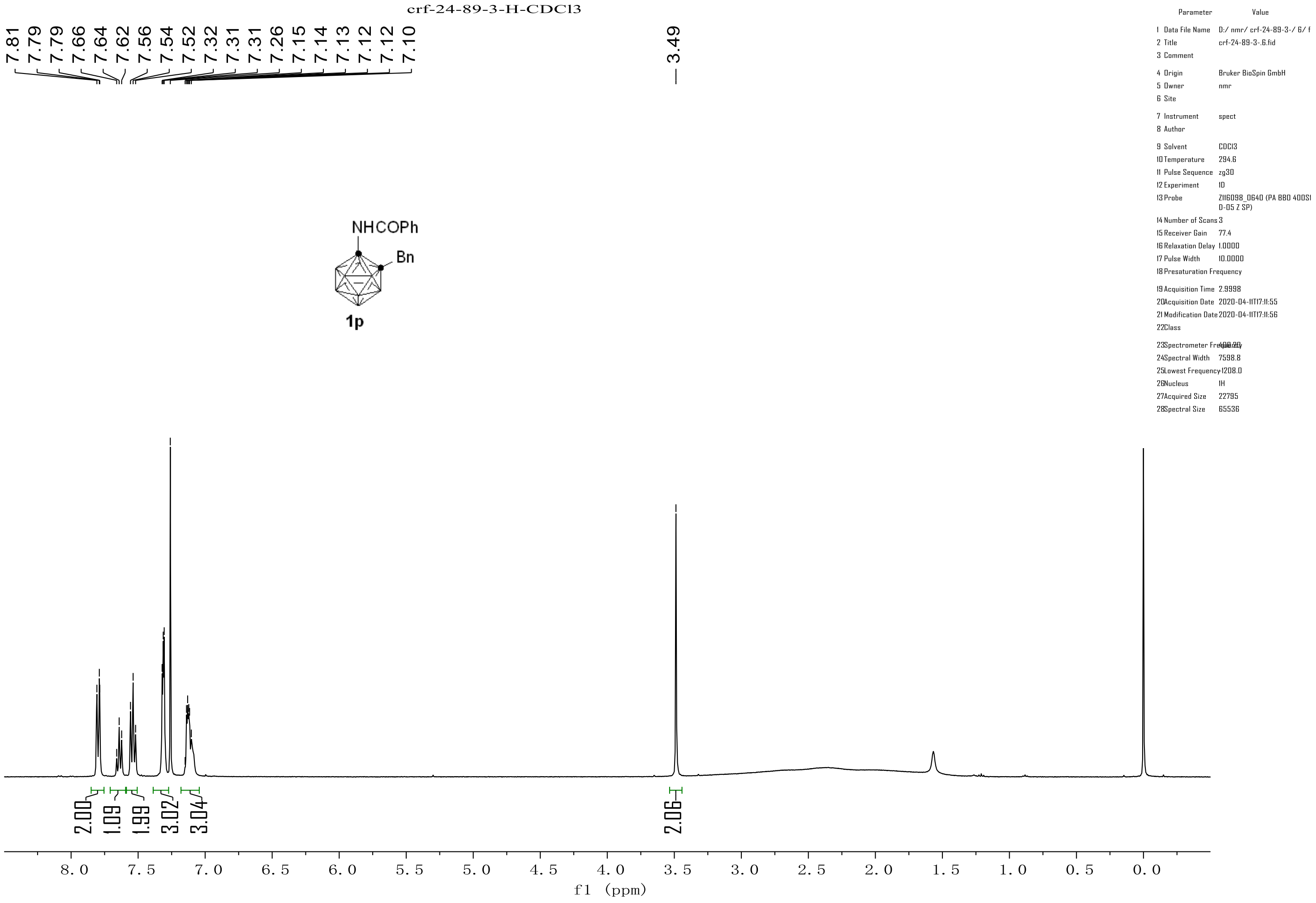

Supplementary Figure 60. $^{13}C\{^{1}H\}$ NMR of **1p**.

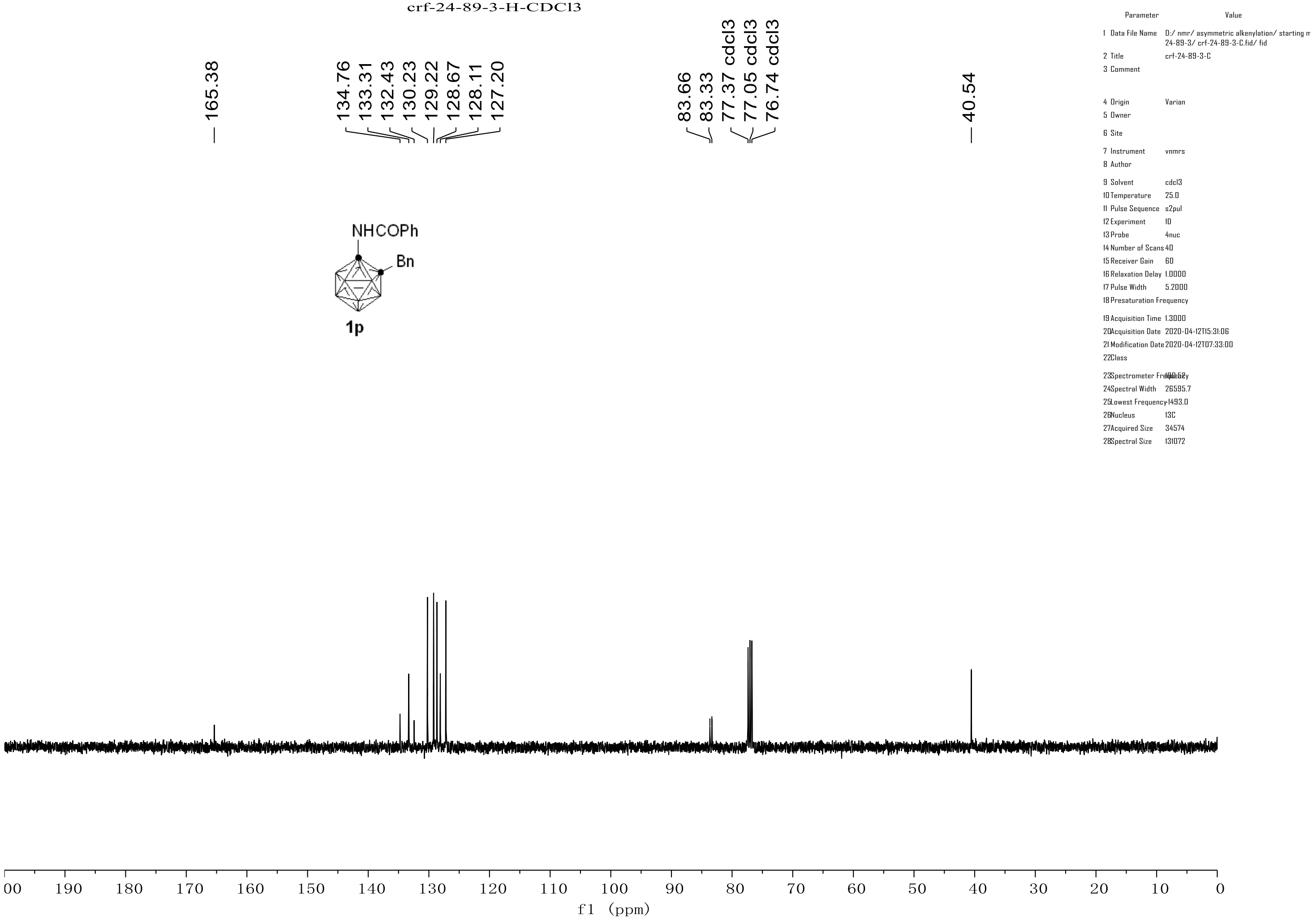

Supplementary Figure 61. $^{11}B\{^{1}H\}$ NMR of **1p**.

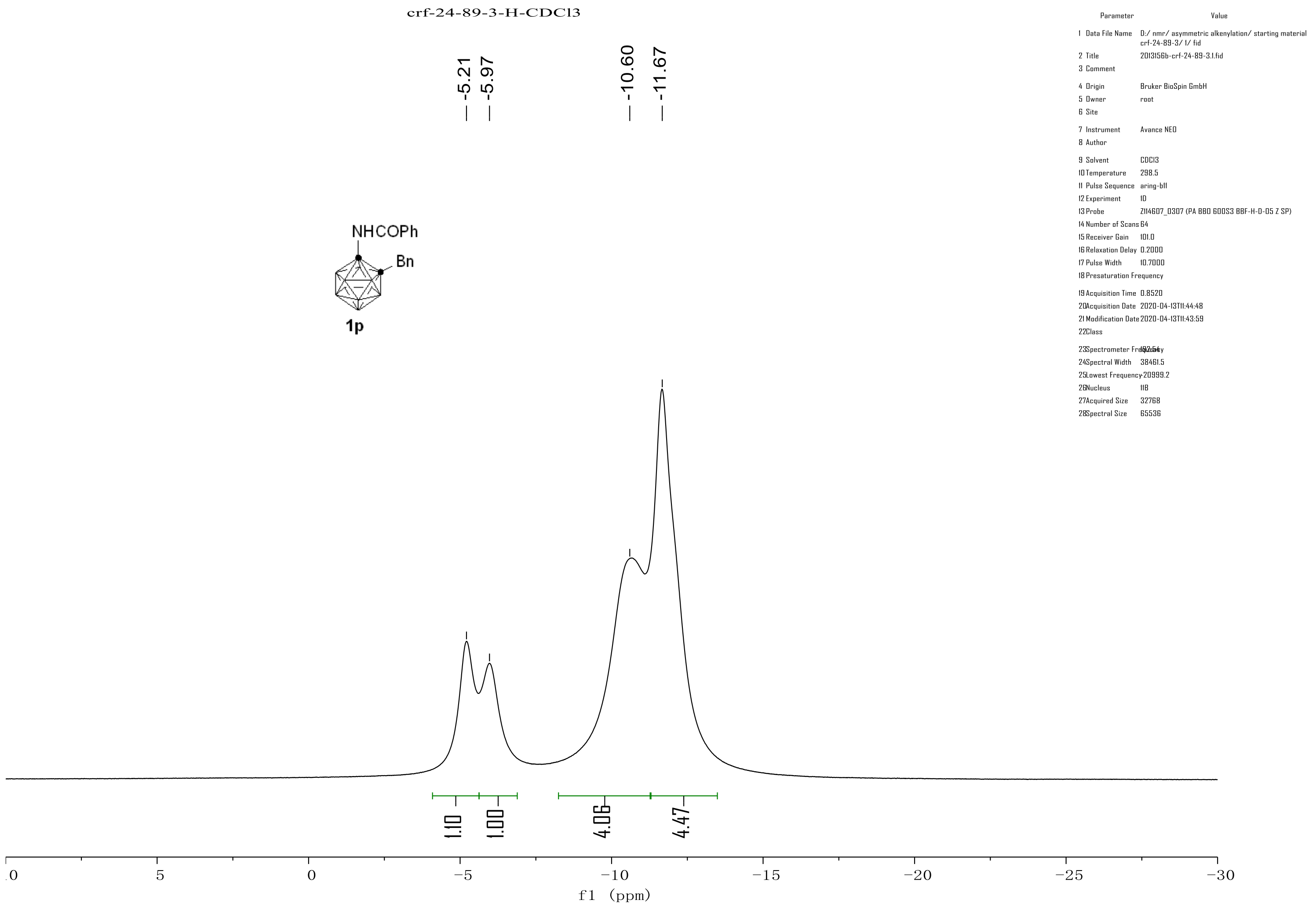

Supplementary Figure 62. ^{1}H NMR of **1q**.

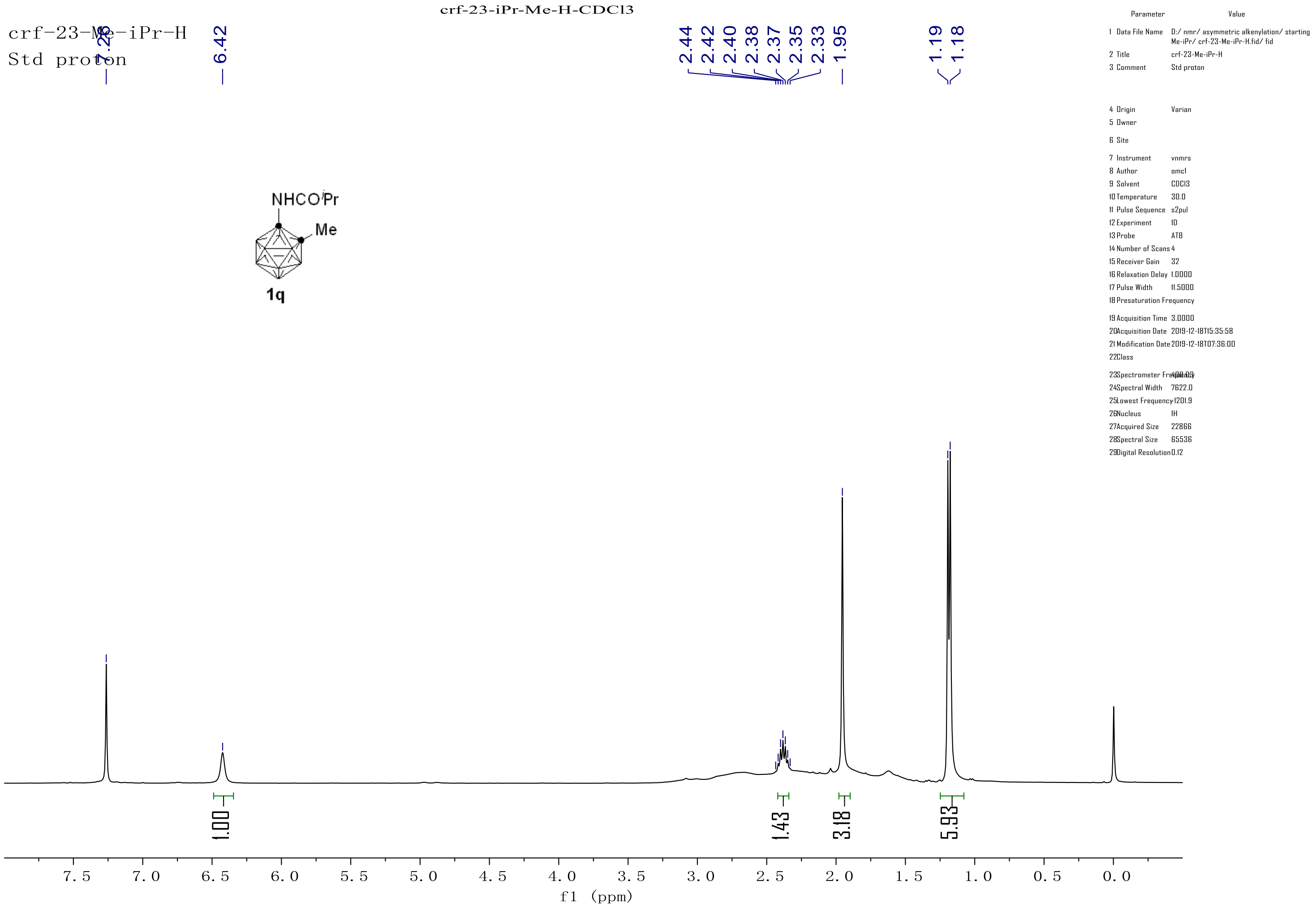

Supplementary Figure 63. $^{13}C\{^1H\}$ NMR of **1q**.

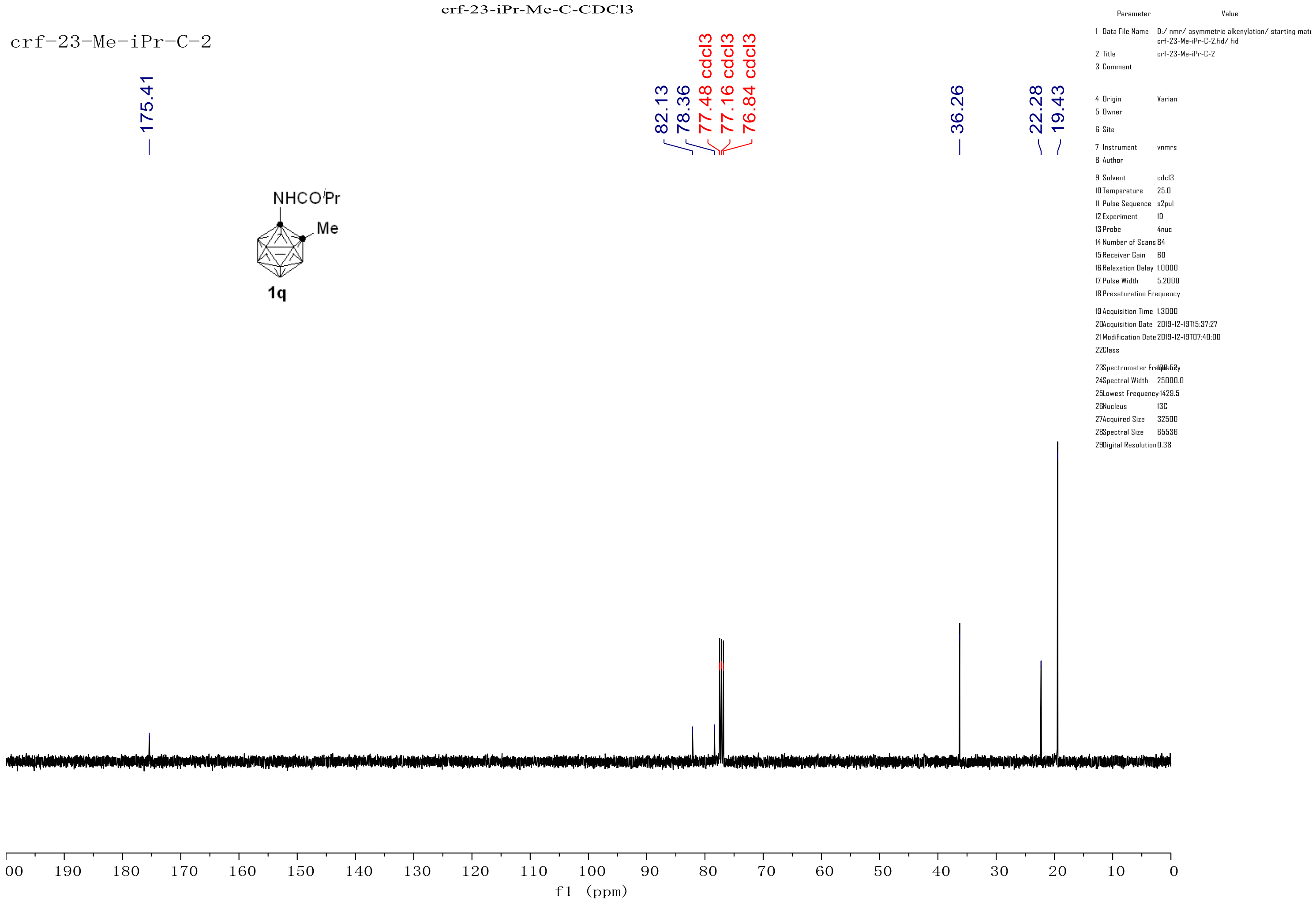

Supplementary Figure 64. $^{11}B\{^1H\}$ NMR of **1q**.

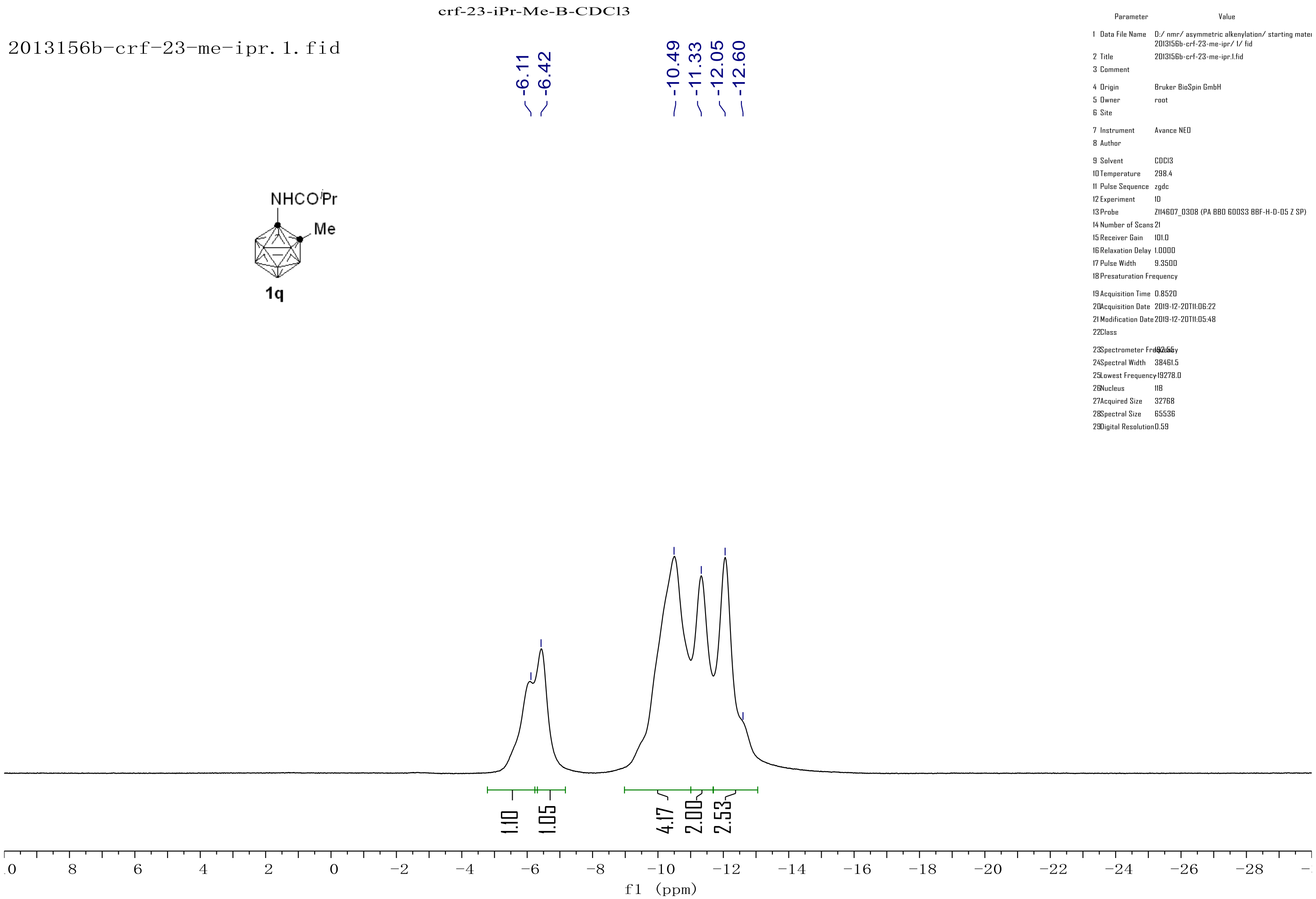

1q

	Parameter	Value
1	Data File Name	D:/ nmr/ asymmetric alkenylation/ starting mater 2013156b-crf-23-me-ipr/ 1/ fid
2	Title	2013156b-crf-23-me-ipr.1.fid
3	Comment	
4	Origin	Bruker BioSpin GmbH
5	Owner	root
6	Site	
7	Instrument	Avance NEO
8	Author	
9	Solvent	CDCl3
10	Temperature	298.4
11	Pulse Sequence	zgdc
12	Experiment	1D
13	Probe	Z114607_0308 (PA BBO 600S3 BBF-H-D-05 Z SP)
14	Number of Scans	21
15	Receiver Gain	101.0
16	Relaxation Delay	1.0000
17	Pulse Width	9.3500
18	Presaturation Frequency	
19	Acquisition Time	0.8520
20	Acquisition Date	2019-12-20T11:06:22
21	Modification Date	2019-12-20T11:05:48
22	Class	
23	Spectrometer Frequency	192.55
24	Spectral Width	38461.5
25	Lowest Frequency	-19278.0
26	Nucleus	11B
27	Acquired Size	32768
28	Spectral Size	65536
29	Digital Resolution	0.59

Supplementary Figure 65. ^{1}H NMR of **1r**.

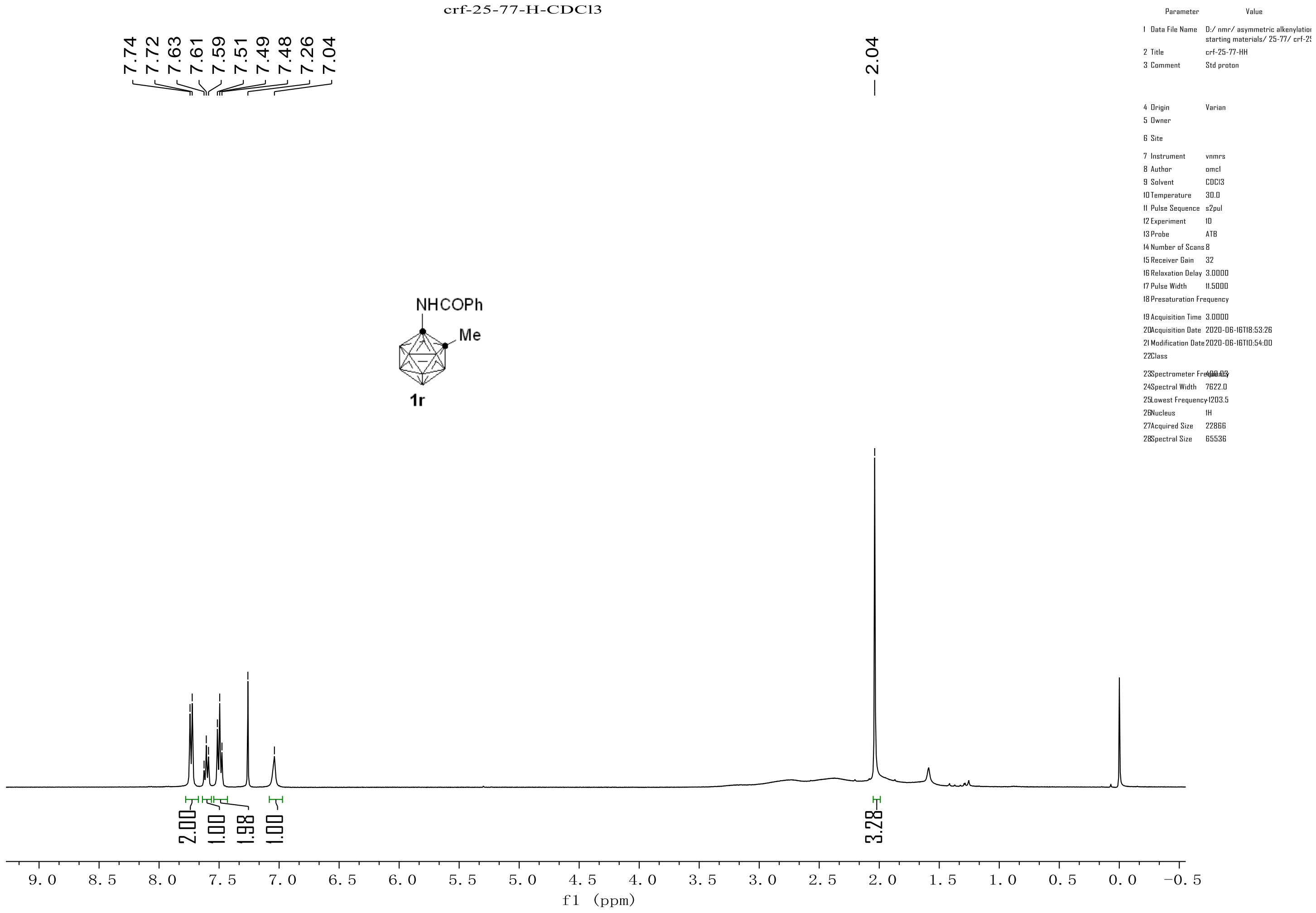

Supplementary Figure 66. $^{13}C\{^{1}H\}$ NMR of **1r**.

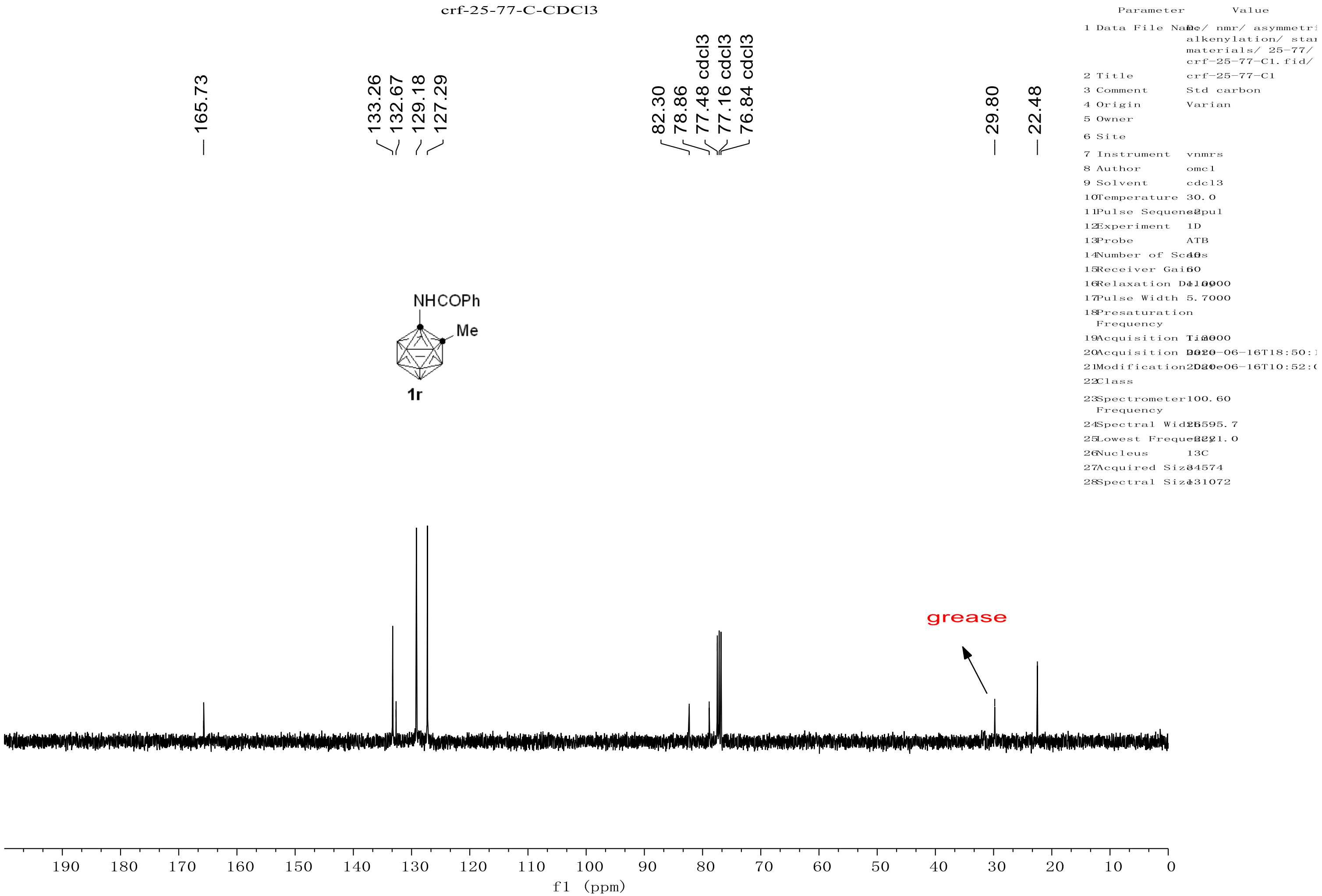

Supplementary Figure 67. $^{11}B\{^1H\}$ NMR of **1r**.

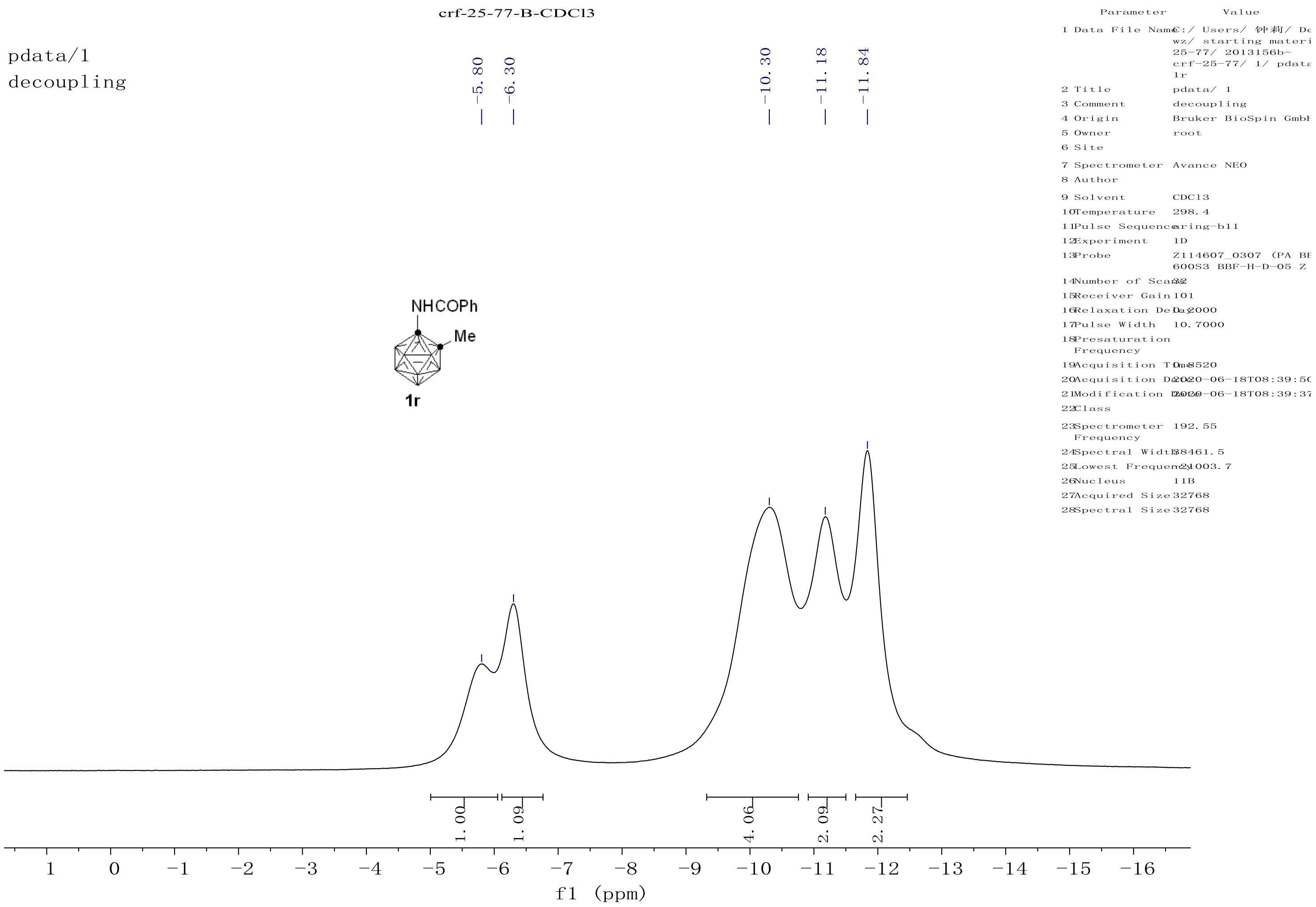

Supplementary Figure 68. ^{1}H NMR of (*S*)-**3ba**.

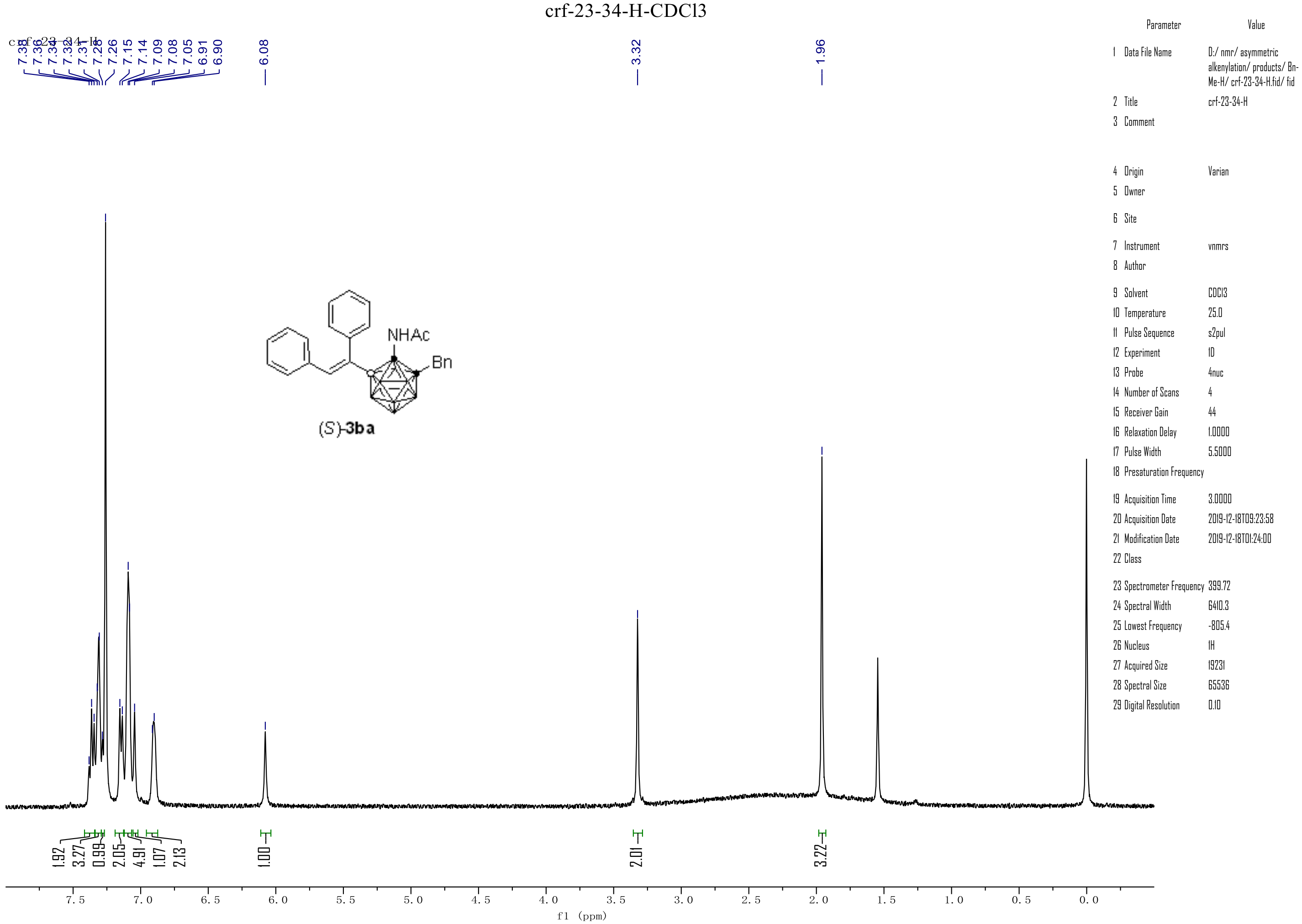

Parameter		Value
1	Data File Name	D:/ nmr/ asymmetric alkenylation/ products/ Bn-Me-H/ crf-23-34-H.fid/ fid
2	Title	crf-23-34-H
3	Comment	
4	Origin	Varian
5	Owner	
6	Site	
7	Instrument	vnmrs
8	Author	
9	Solvent	CDCl3
10	Temperature	25.0
11	Pulse Sequence	s2pul
12	Experiment	1D
13	Probe	4nuc
14	Number of Scans	4
15	Receiver Gain	44
16	Relaxation Delay	1.0000
17	Pulse Width	5.5000
18	Presaturation Frequency	
19	Acquisition Time	3.0000
20	Acquisition Date	2019-12-18T09:23:58
21	Modification Date	2019-12-18T01:24:00
22	Class	
23	Spectrometer Frequency	399.72
24	Spectral Width	6410.3
25	Lowest Frequency	-805.4
26	Nucleus	1H
27	Acquired Size	19231
28	Spectral Size	65536
29	Digital Resolution	0.10

Supplementary Figure 69. $^{13}C\{^{1}H\}$ NMR of (*S*)-**3ba**.

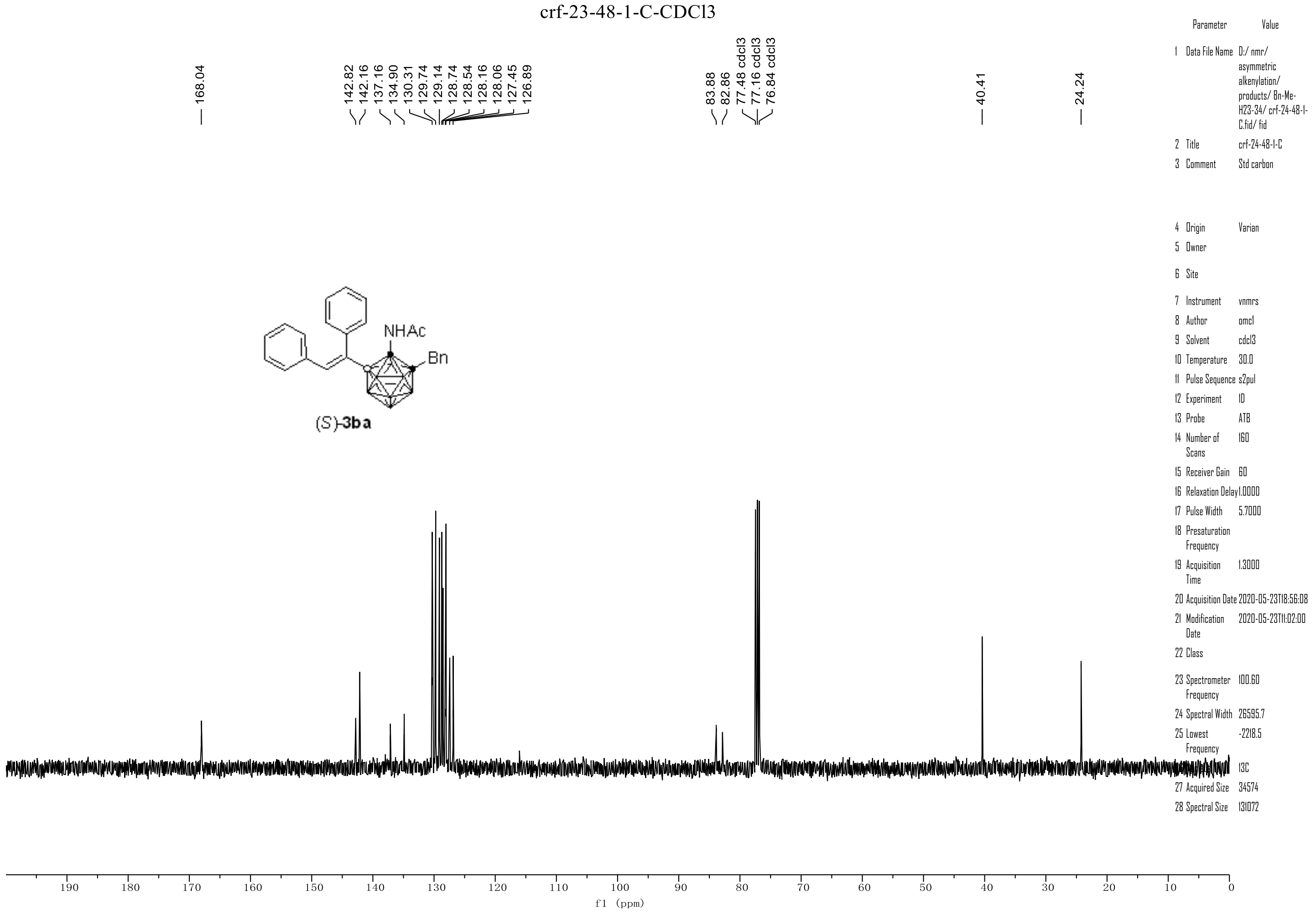

Supplementary Figure 70. $^{11}B\{^{1}H\}$ NMR of (*S*)-**3ba**.

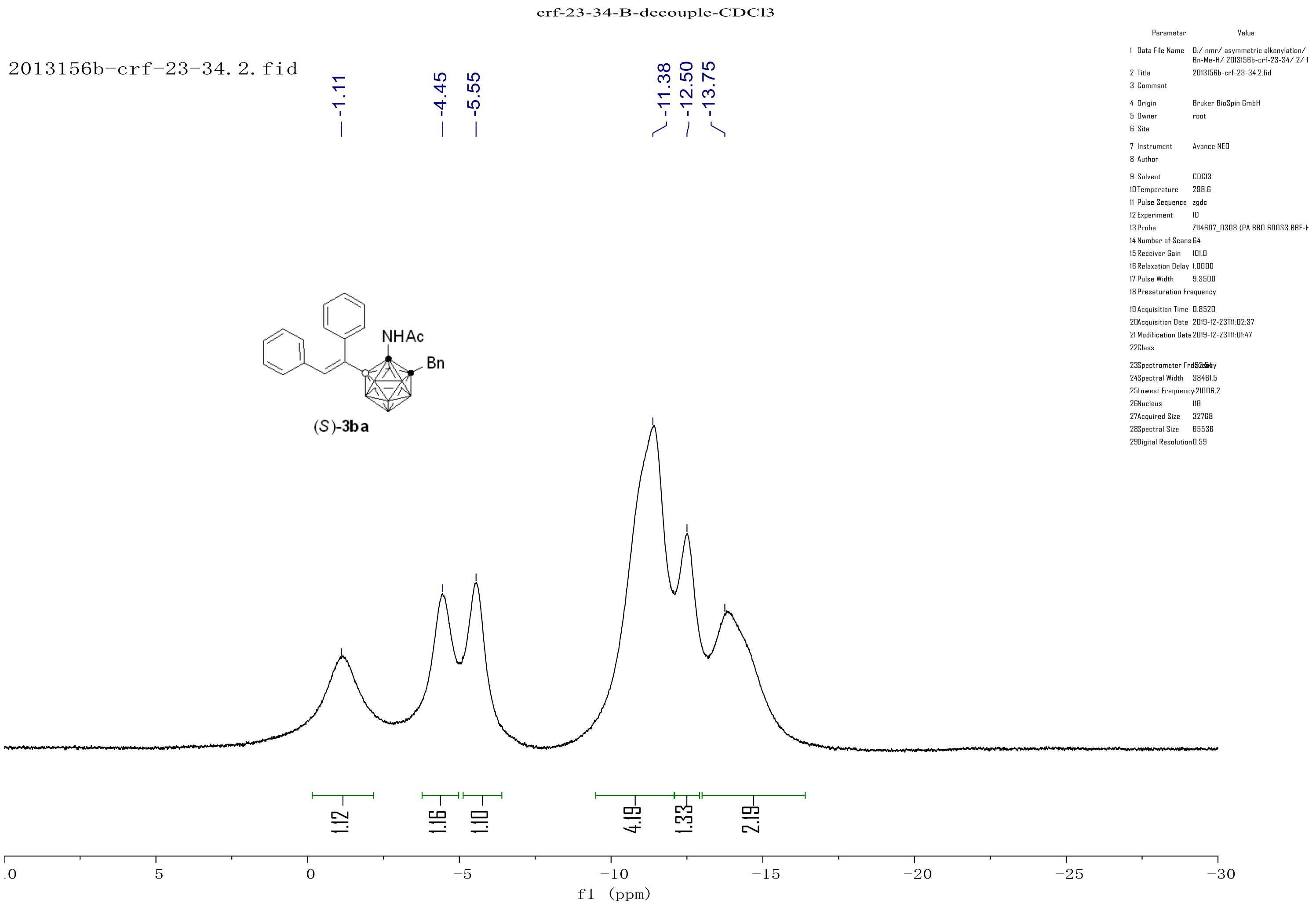

Supplementary Figure 71. ^{11}B NMR of (*S*)-**3ba**.

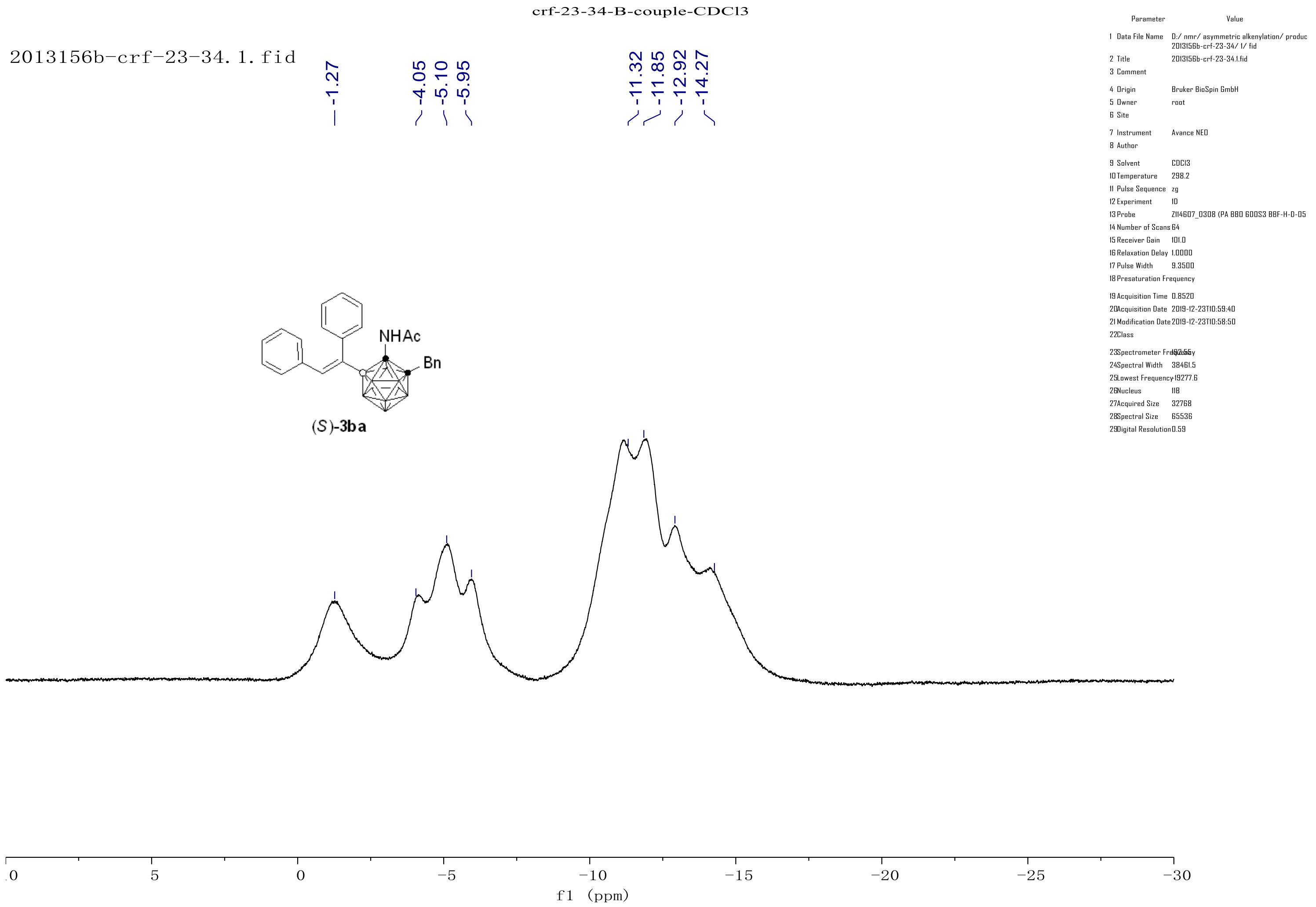

Supplementary Figure 72. ^{1}H NMR of (*S*)-**3bb**.

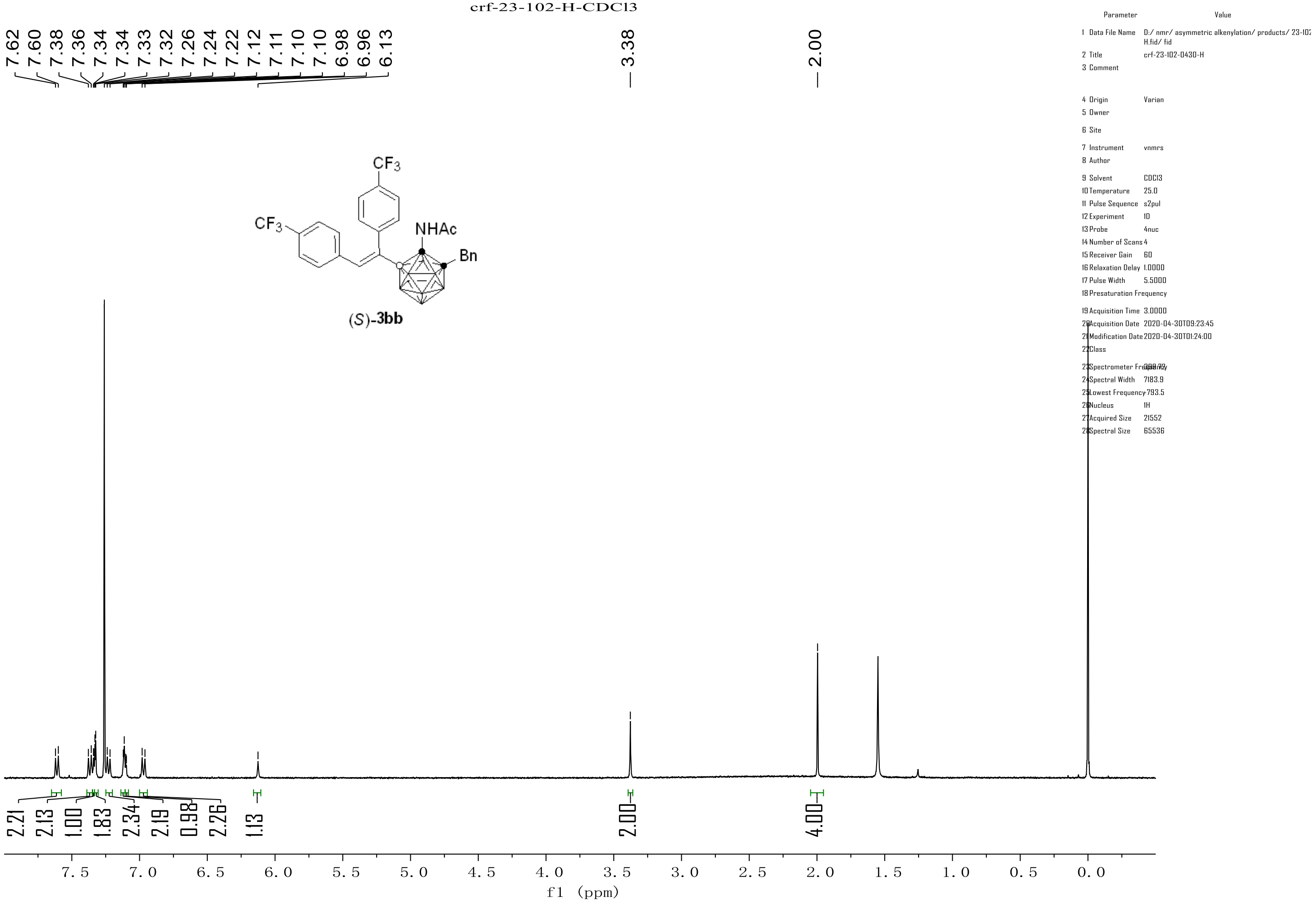

Supplementary Figure 73. $^{13}C\{^{1}H\}$ NMR of (*S*)-**3bb**.

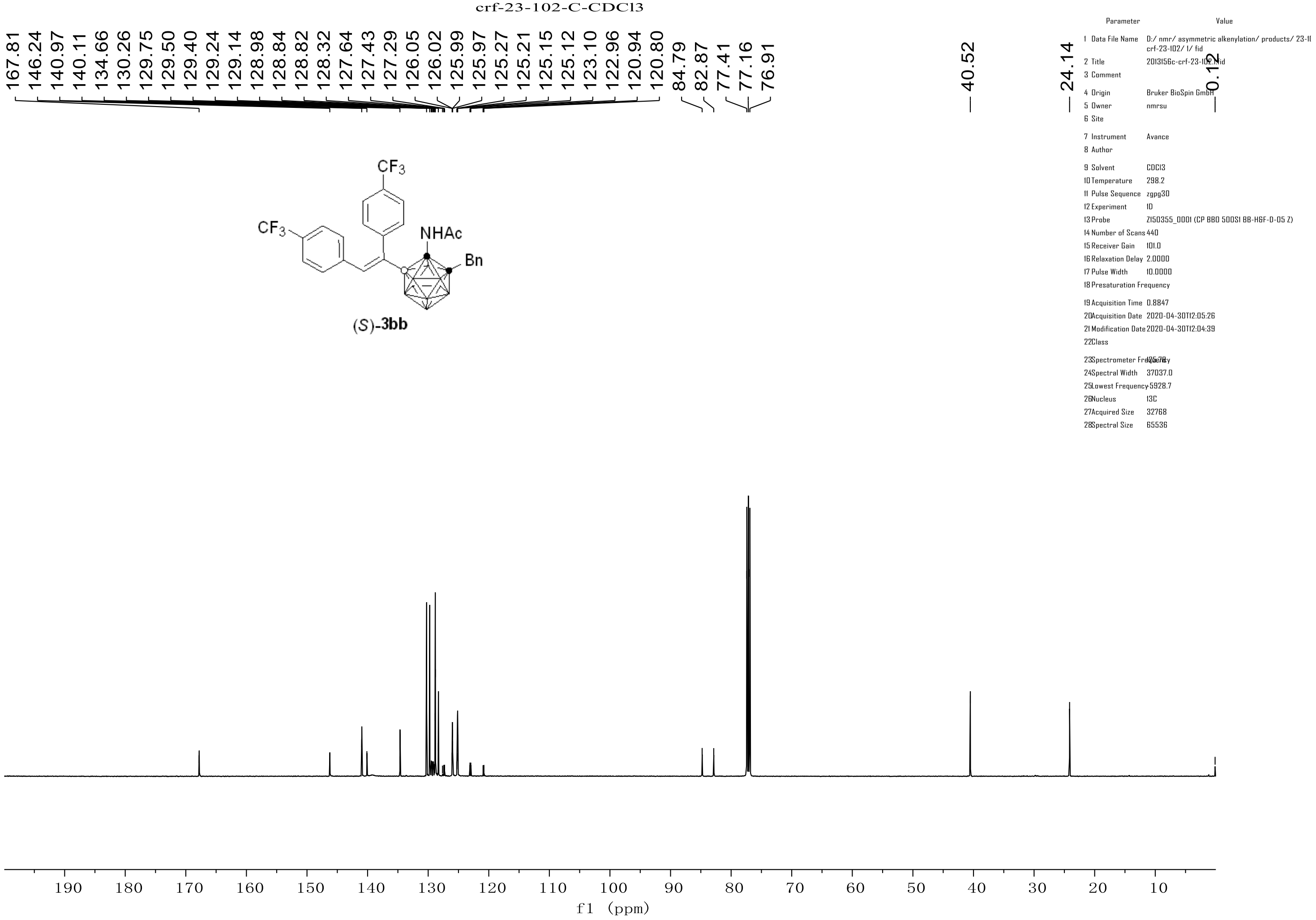

Supplementary Figure 74. $^{11}B\{^{1}H\}$ NMR of (*S*)-**3bb**.

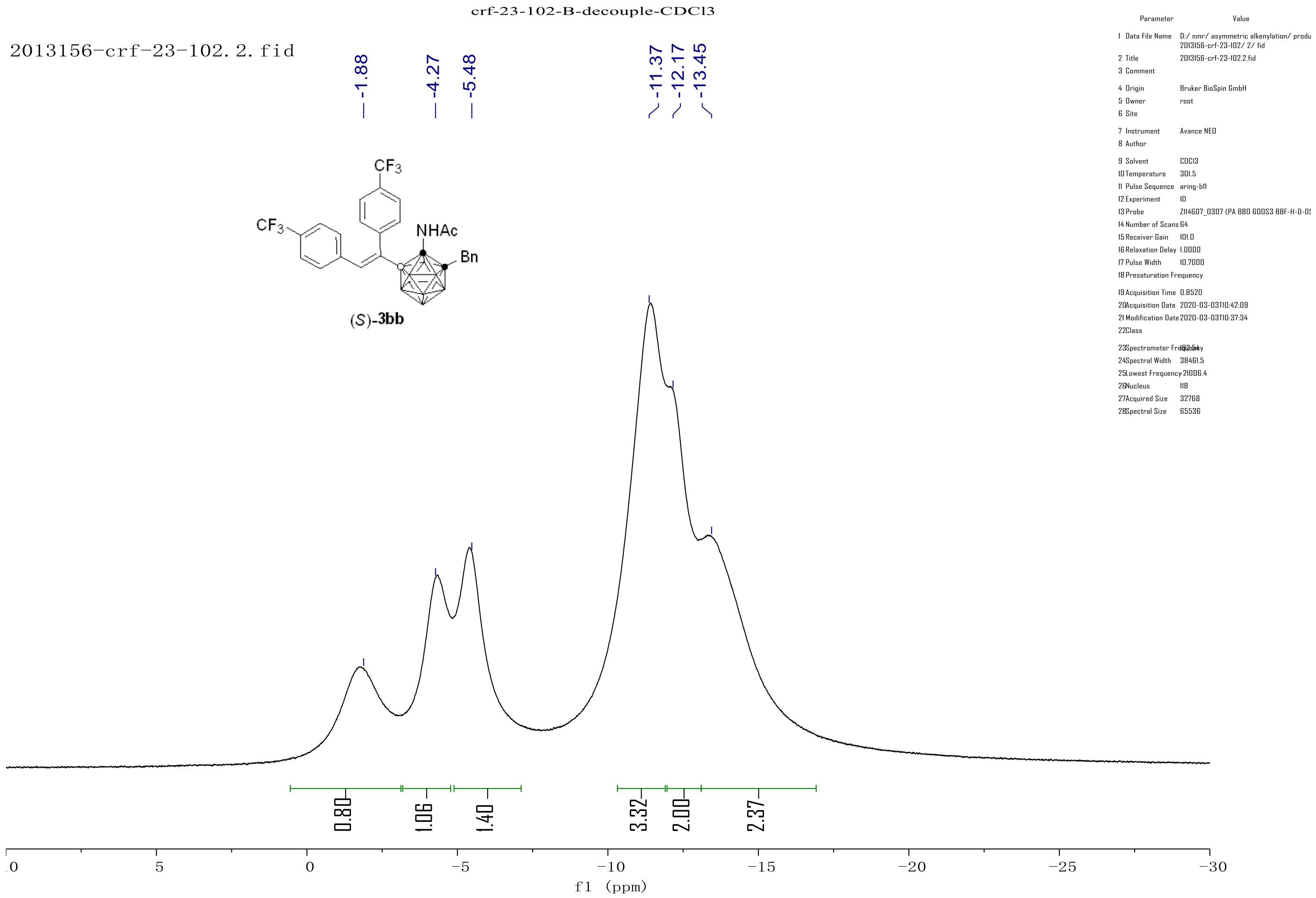

Supplementary Figure 75. ^{11}B NMR of (*S*)-**3bb**.

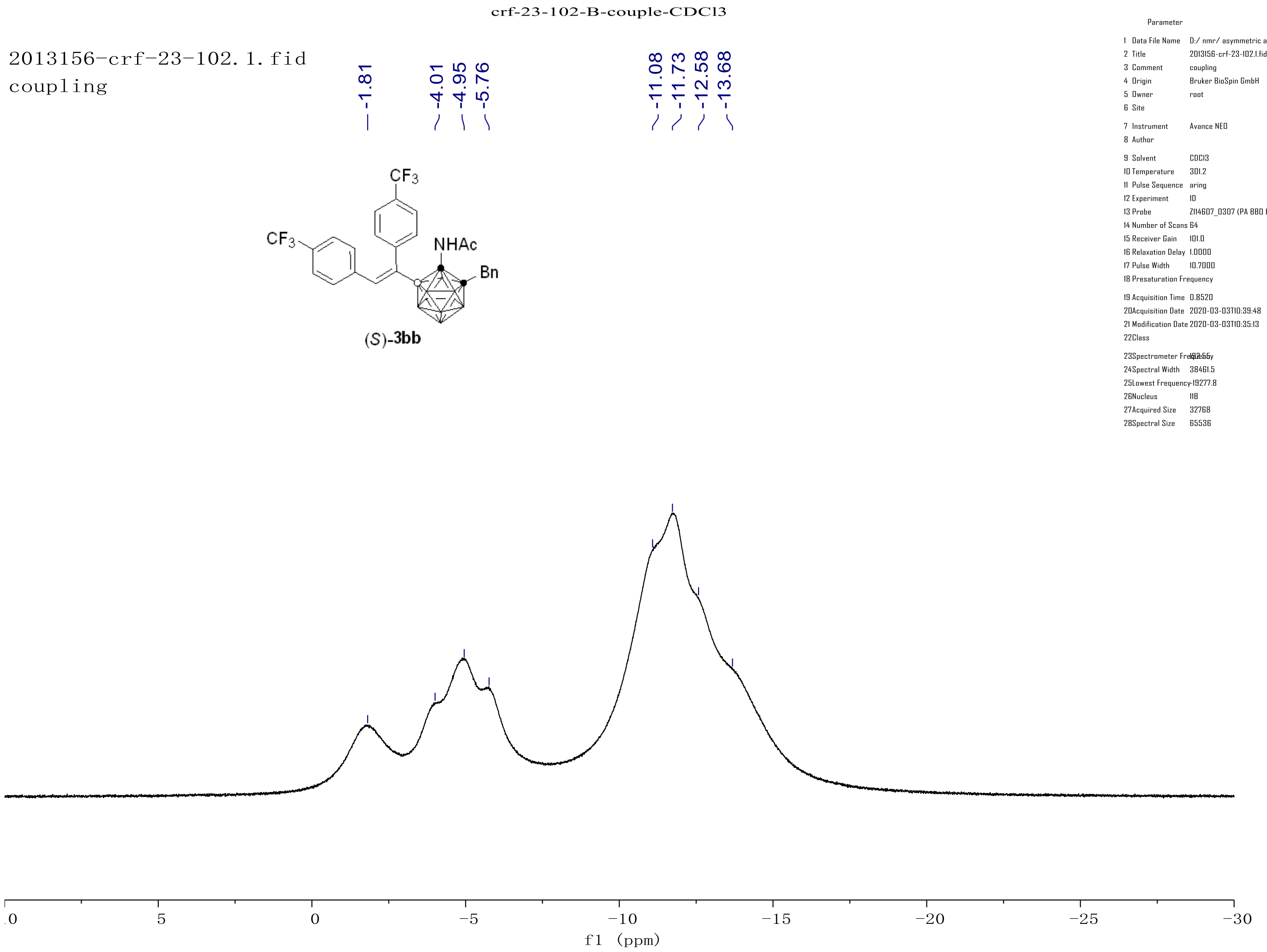

	Parameter	Value
1	Data File Name	D:/ nmr/ asymmetric alkenylation/ products/ 23-102/
2	Title	2013156-crf-23-102.1.fid
3	Comment	coupling
4	Origin	Bruker BioSpin GmbH
5	Owner	root
6	Site	
7	Instrument	Avance NEO
8	Author	
9	Solvent	CDCl3
10	Temperature	301.2
11	Pulse Sequence	aring
12	Experiment	1D
13	Probe	Z114607_0307 (PA BBO 600S3 BBF-H-D-05 Z SP)
14	Number of Scans	64
15	Receiver Gain	101.0
16	Relaxation Delay	1.0000
17	Pulse Width	10.7000
18	Presaturation Frequency	
19	Acquisition Time	0.8520
20	Acquisition Date	2020-03-03T10:39:48
21	Modification Date	2020-03-03T10:35:13
22	Class	
23	Spectrometer Frequency	192.55
24	Spectral Width	38461.5
25	Lowest Frequency	-19277.8
26	Nucleus	11B
27	Acquired Size	32768
28	Spectral Size	65536

Supplementary Figure 76. ^{19}F NMR of (*S*)-**3bb**.

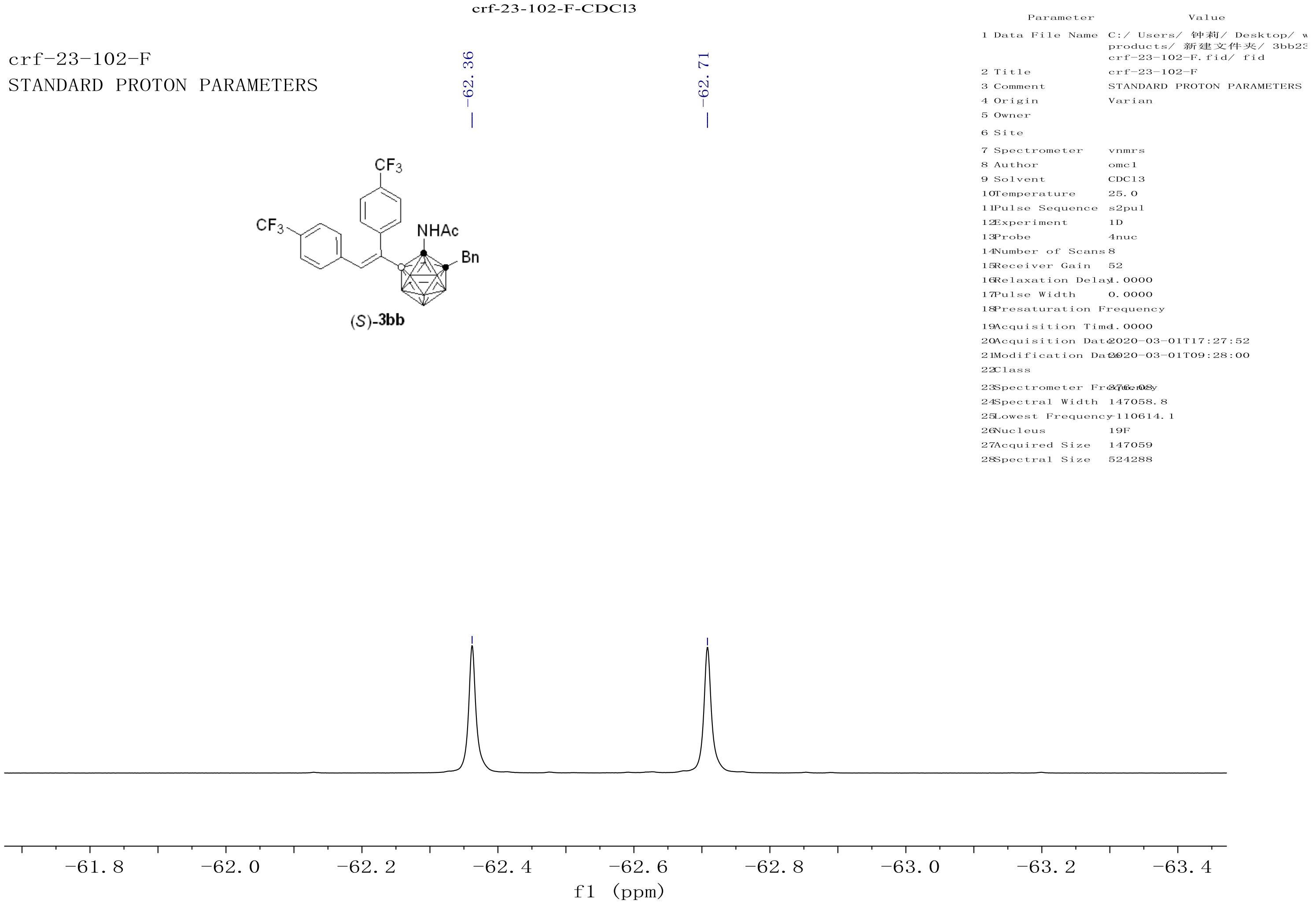

Supplementary Figure 77. ^{1}H NMR of (*S*)-**3bc**.

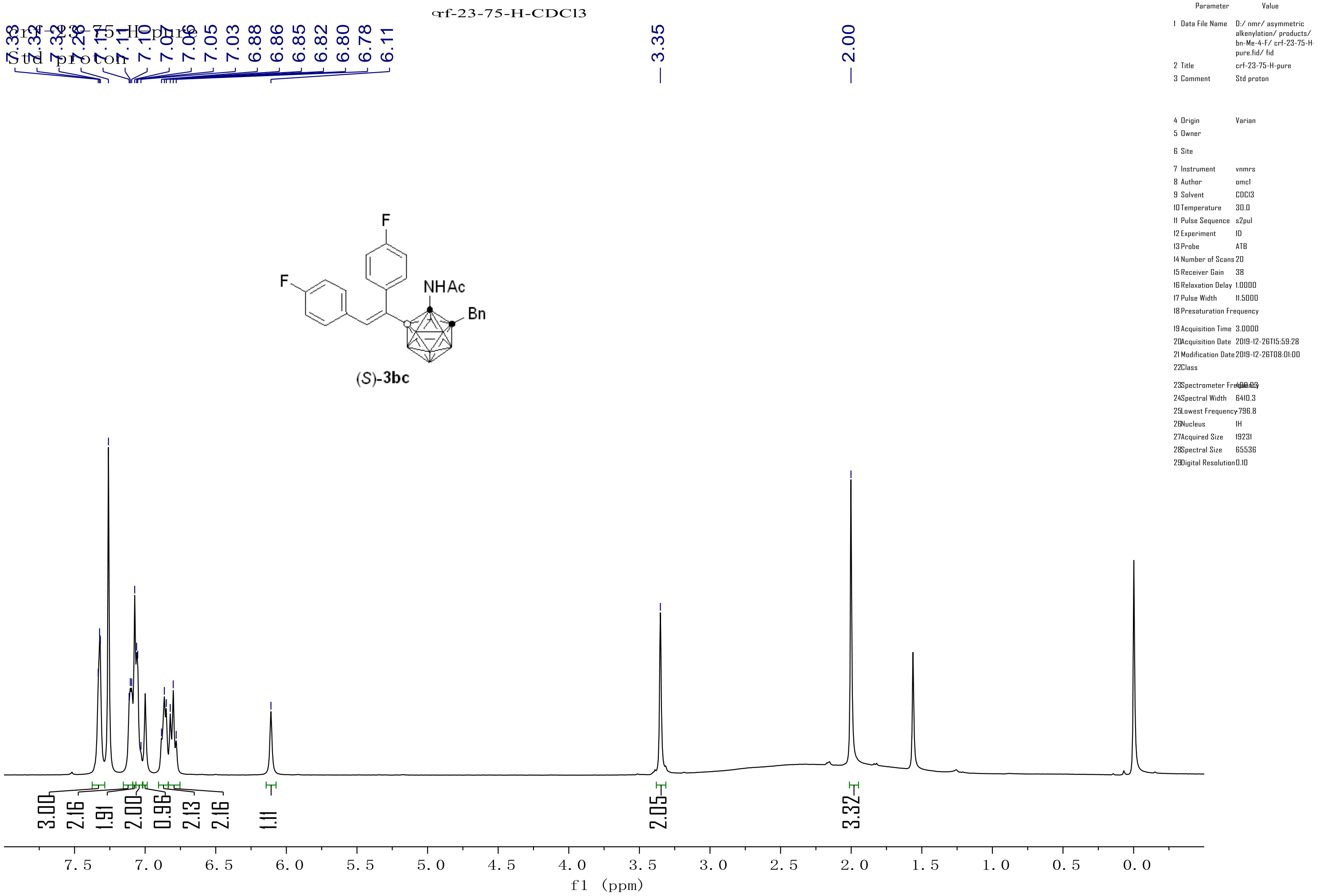

Supplementary Figure 78. $^{13}C\{^{1}H\}$ NMR of (*S*)-**3bc**.

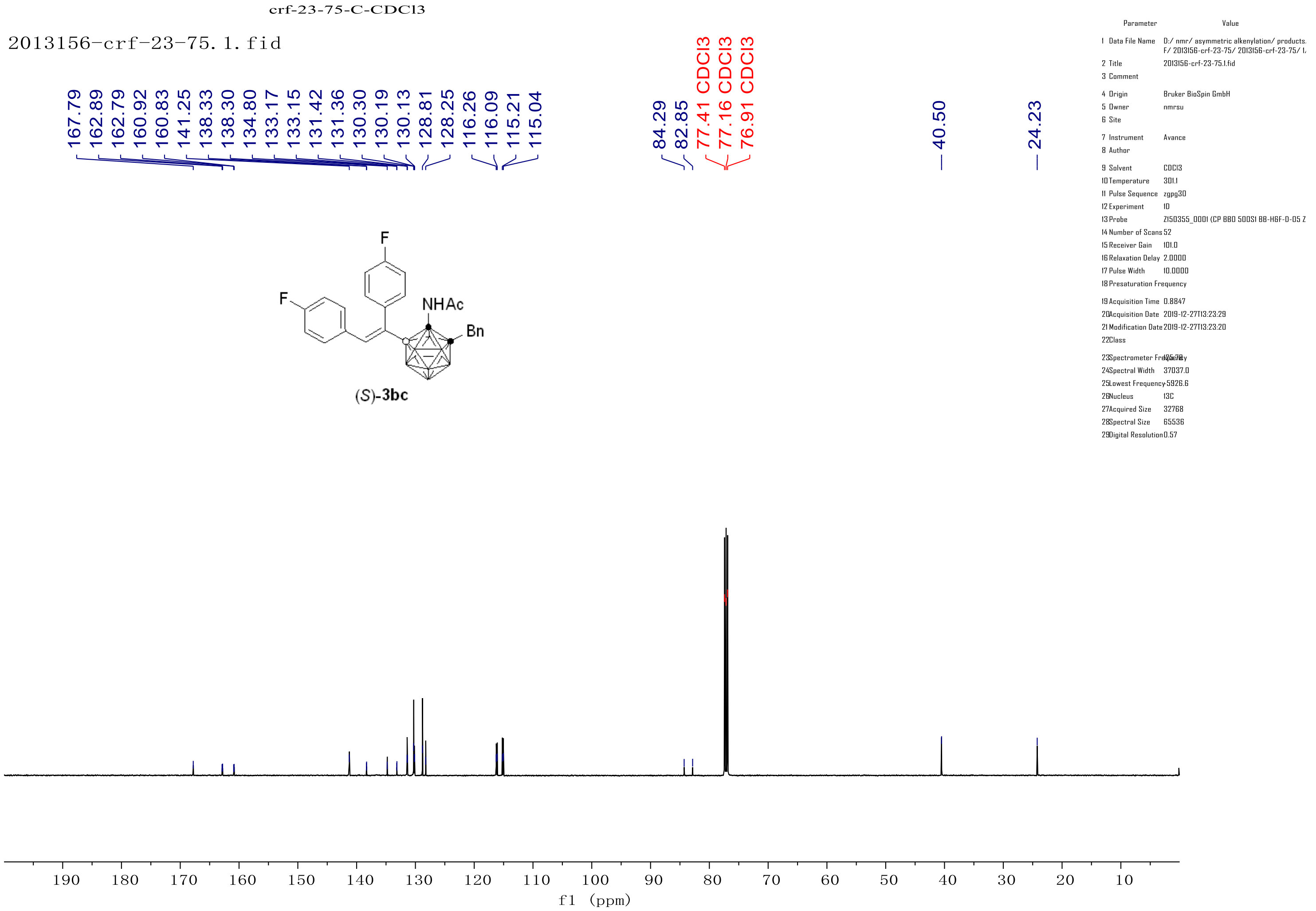

Supplementary Figure 79. $^{11}B\{^{1}H\}$ NMR of (*S*)-**3bc**.

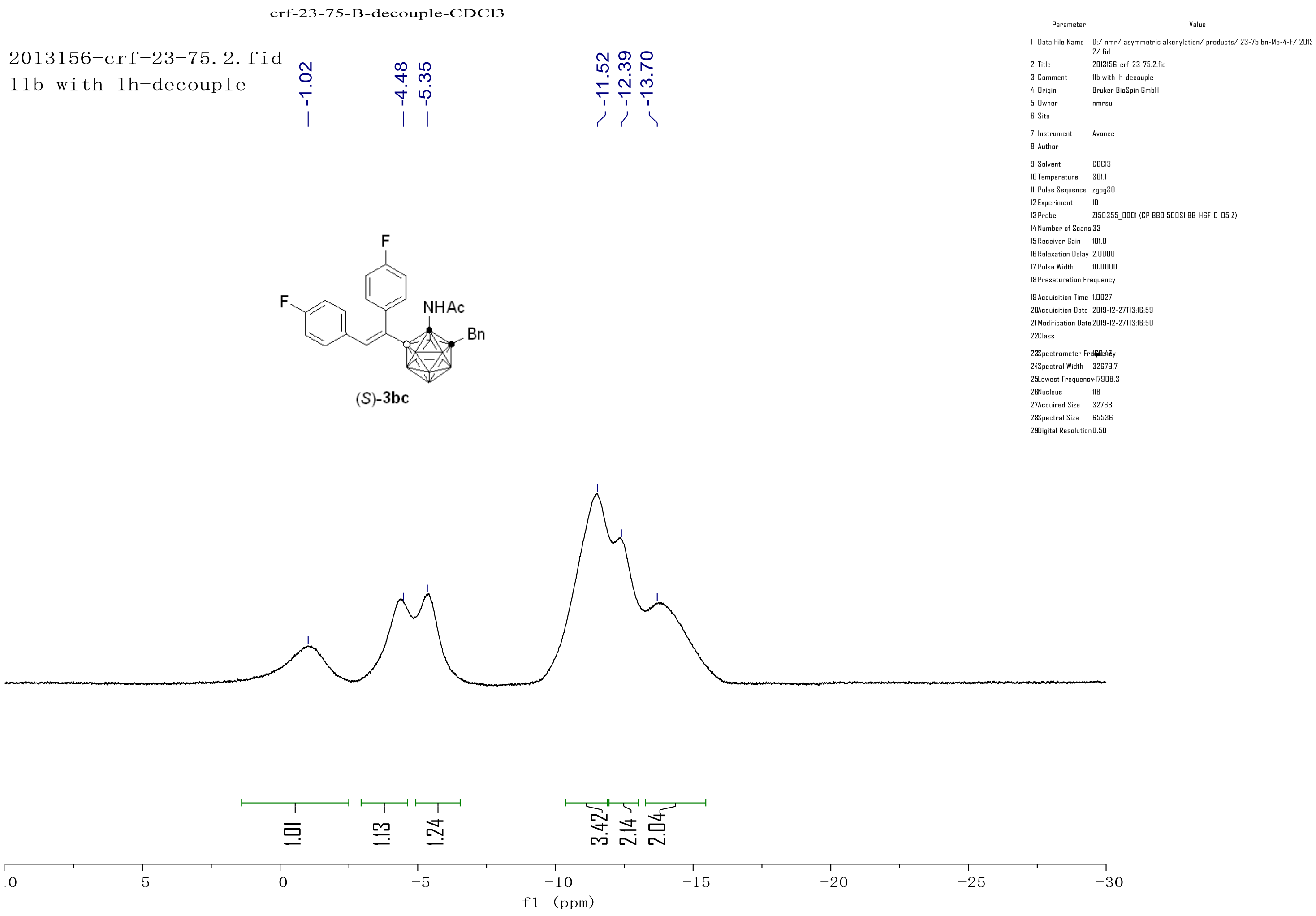

	Parameter	Value
1	Data File Name	D:/ nmr/ asymmetric alkenylation/ products/ 23-75 bn-Me-4-F/ 2013 2/ fid
2	Title	2013156-crf-23-75.2.fid
3	Comment	11b with 1h-decouple
4	Origin	Bruker BioSpin GmbH
5	Owner	nmrsu
6	Site	
7	Instrument	Avance
8	Author	
9	Solvent	CDCl3
10	Temperature	301.1
11	Pulse Sequence	zgpg30
12	Experiment	1D
13	Probe	Z150355_0001 (CP BBO 500S1 BB-H&F-D-05 Z)
14	Number of Scans	33
15	Receiver Gain	101.0
16	Relaxation Delay	2.0000
17	Pulse Width	10.0000
18	Presaturation Frequency	
19	Acquisition Time	1.0027
20	Acquisition Date	2019-12-27T13:16:59
21	Modification Date	2019-12-27T13:16:50
22	Class	
23	Spectrometer Frequency	160.47
24	Spectral Width	32679.7
25	Lowest Frequency	-17908.3
26	Nucleus	11B
27	Acquired Size	32768
28	Spectral Size	65536
29	Digital Resolution	0.50

Supplementary Figure 80. $^{11}B\{^{1}H\}$ NMR of (*S*)-**3bc**.

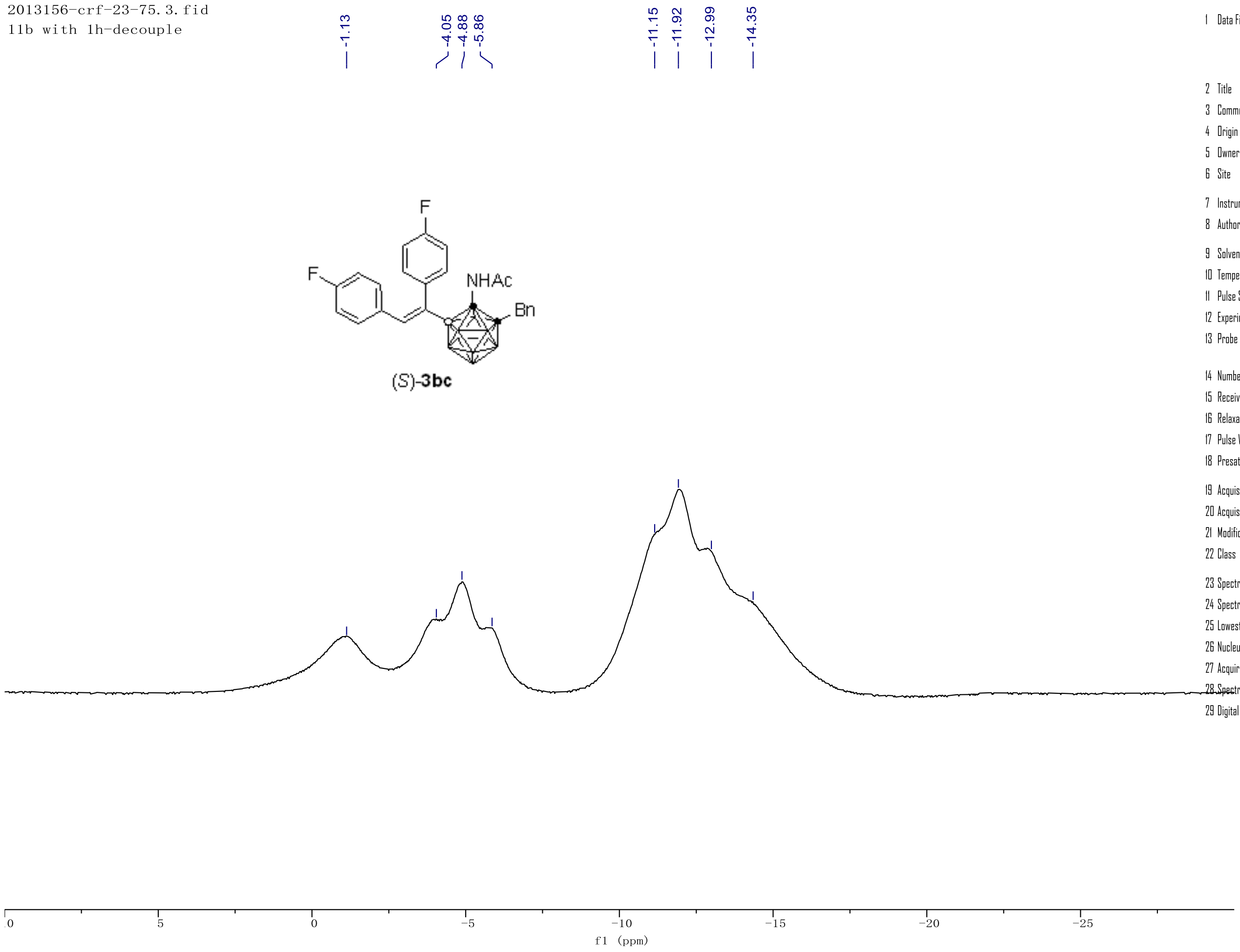

	Parameter	Value
1	Data File Name	D:/ nmr/ asymmetric alkenylation/ products/ 23-75 bn-Me-4-F/ 2013156-crf-23-75/ 2013156-crf-23-75/ 3/ fid
2	Title	2013156-crf-23-75.3.fid
3	Comment	11b with 1h-decouple
4	Origin	Bruker BioSpin GmbH
5	Owner	nmrsu
6	Site	
7	Instrument	Avance
8	Author	
9	Solvent	CDCl3
10	Temperature	301.1
11	Pulse Sequence	zg30
12	Experiment	1D
13	Probe	Z150355_0001 (CP BBO 500S1 BB-H&F-D-05 Z)
14	Number of Scans	72
15	Receiver Gain	101.0
16	Relaxation Delay	0.5000
17	Pulse Width	10.0000
18	Presaturation Frequency	
19	Acquisition Time	1.0027
20	Acquisition Date	2019-12-27T13:19:12
21	Modification Date	2019-12-27T13:19:04
22	Class	
23	Spectrometer Frequency	160.47
24	Spectral Width	32679.7
25	Lowest Frequency	-17908.3
26	Nucleus	11B
27	Acquired Size	32768
28	Spectral Size	65536
29	Digital Resolution	0.50

Supplementary Figure 81. ^{19}F NMR of (*S*)-**3bc**.

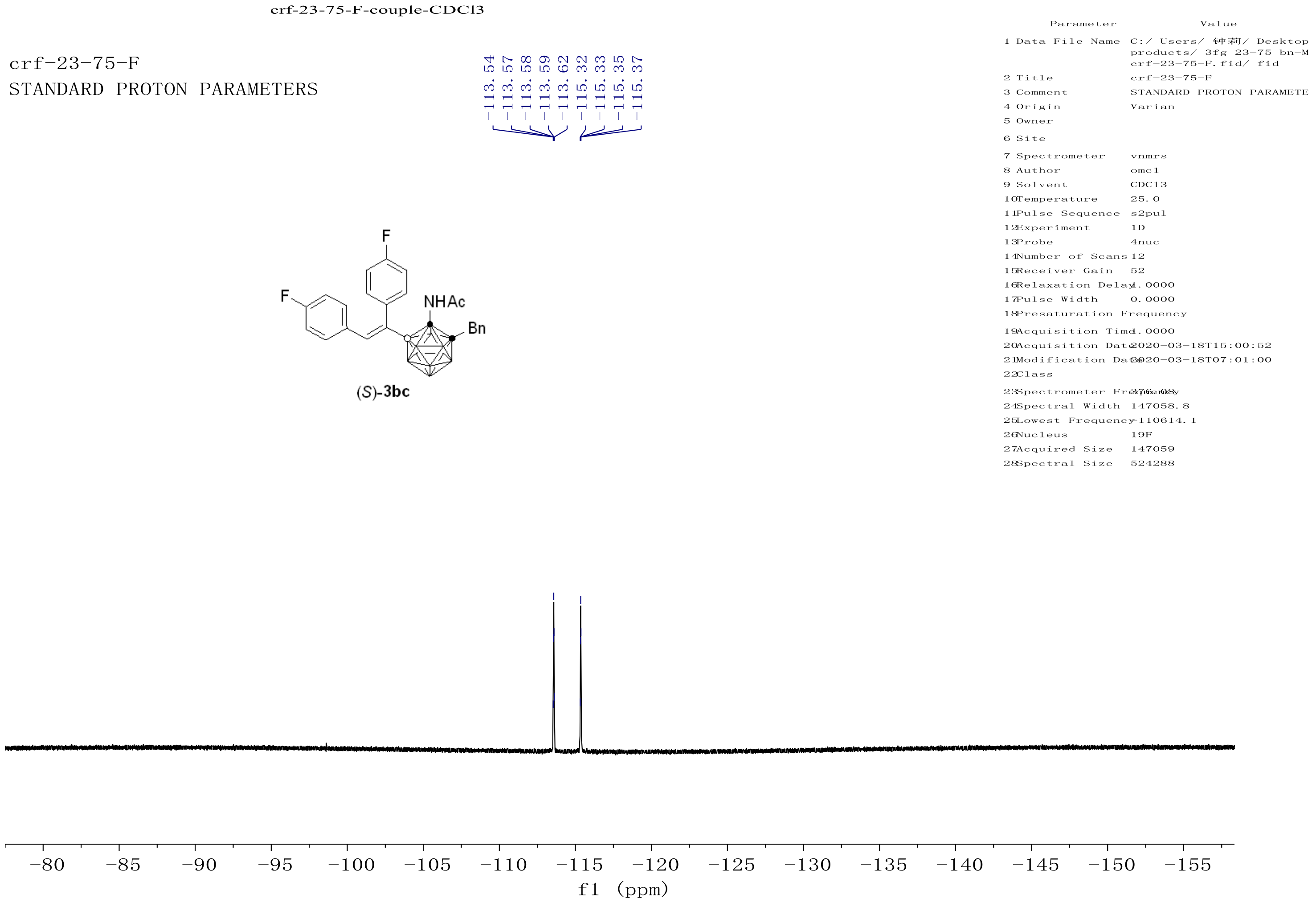

Supplementary Figure 82. ^{1}H NMR of (*S*)-**3bd**.

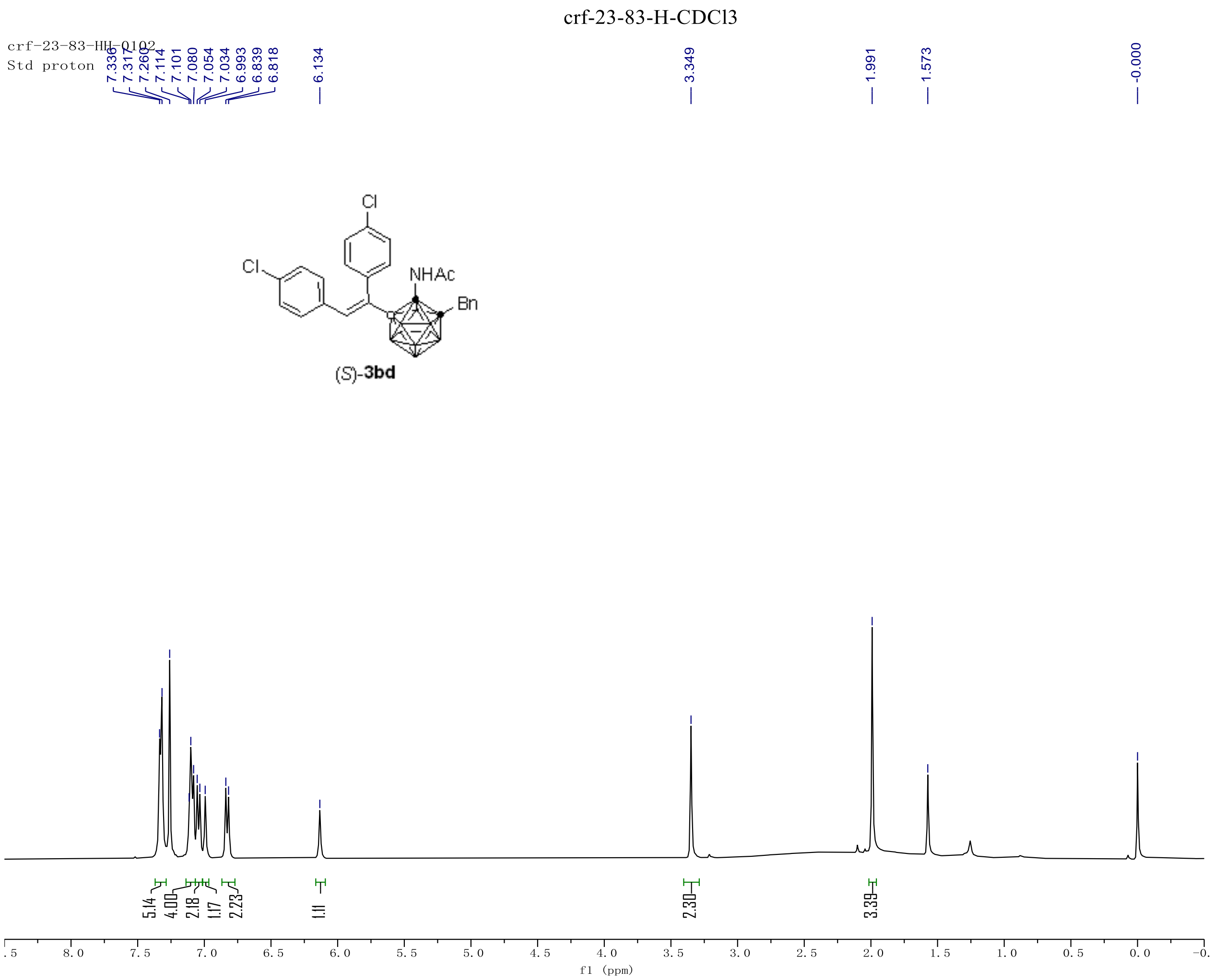

	Parameter	Value
1	Data File Name	D:/ nmr/ asymmetric alkenylation/ products/ 23-83BnMe-Cl/ crf-23-83-HH-0102.fid/ fid
2	Title	crf-23-83-HH-0102
3	Comment	Std proton
4	Origin	Varian
5	Owner	
6	Site	
7	Instrument	vnmrs
8	Author	omcl
9	Solvent	CDCl3
10	Temperature	30.0
11	Pulse Sequence	s2pul
12	Experiment	1D
13	Probe	ATB
14	Number of Scans	4
15	Receiver Gain	38
16	Relaxation Delay	1.0000
17	Pulse Width	11.5000
18	Presaturation Frequency	
19	Acquisition Time	3.0000
20	Acquisition Date	2020-01-02T15:40:59
21	Modification Date	2020-01-02T07:41:00
22	Class	
23	Spectrometer Frequency	400.03
24	Spectral Width	7225.4
25	Lowest Frequency	-804.9
26	Nucleus	1H
27	Acquired Size	21676
28	Spectral Size	65536
29	Digital Resolution	0.11

Supplementary Figure 83. $^{13}C\{^{1}H\}$ NMR of (*S*)-**3bd**.

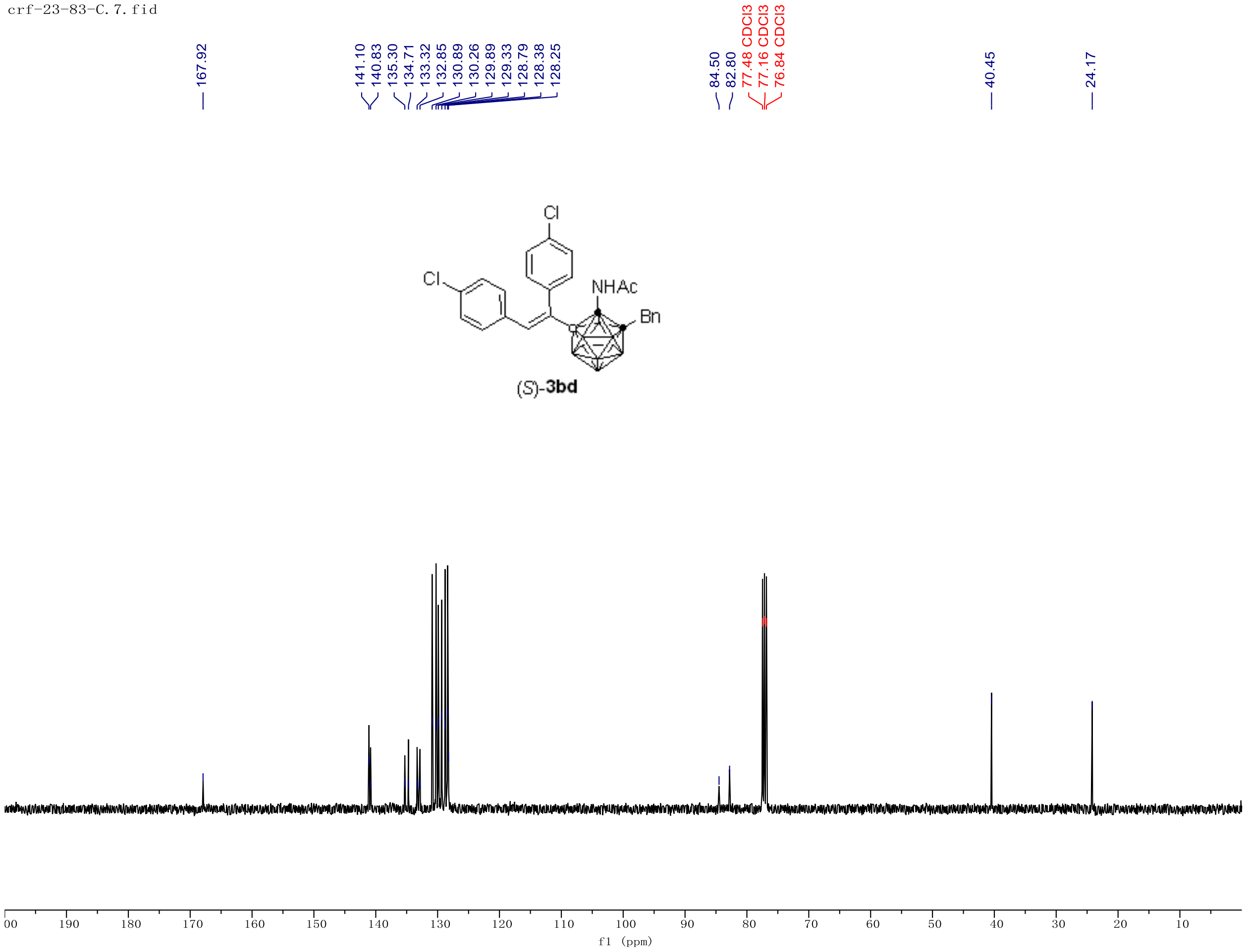

	Parameter	Value
1	Data File Name	D:/ nmr/ asymmetric alkenylation/ products/ 23-83/ crf-23-83-C/ 7/ fid
2	Title	crf-23-83-C.7.fid
3	Comment	
4	Origin	Bruker BioSpin GmbH
5	Owner	nmr
6	Site	
7	Instrument	spect
8	Author	
9	Solvent	CDCl3
10	Temperature	294.8
11	Pulse Sequence	zgpg30
12	Experiment	1D
13	Probe	Z116098_0640 (PA BBO 400SI BBF-H-D-05 Z SP)
14	Number of Scans	154
15	Receiver Gain	195.5
16	Relaxation Delay	1.0000
17	Pulse Width	10.0000
18	Presaturation Frequency	
19	Acquisition Time	1.2999
20	Acquisition Date	2019-12-30T17:22:28
21	Modification Date	2019-12-30T17:22:29
22	Class	
23	Spectrometer Frequency	100.64
24	Spectral Width	25252.5
25	Lowest Frequency	-1549.6
26	Nucleus	13C
27	Acquired Size	32827
28	Spectral Size	131072
29	Digital Resolution	0.19

Supplementary Figure 84. $^{11}B\{^1H\}$ NMR of (*S*)-**3bd**.

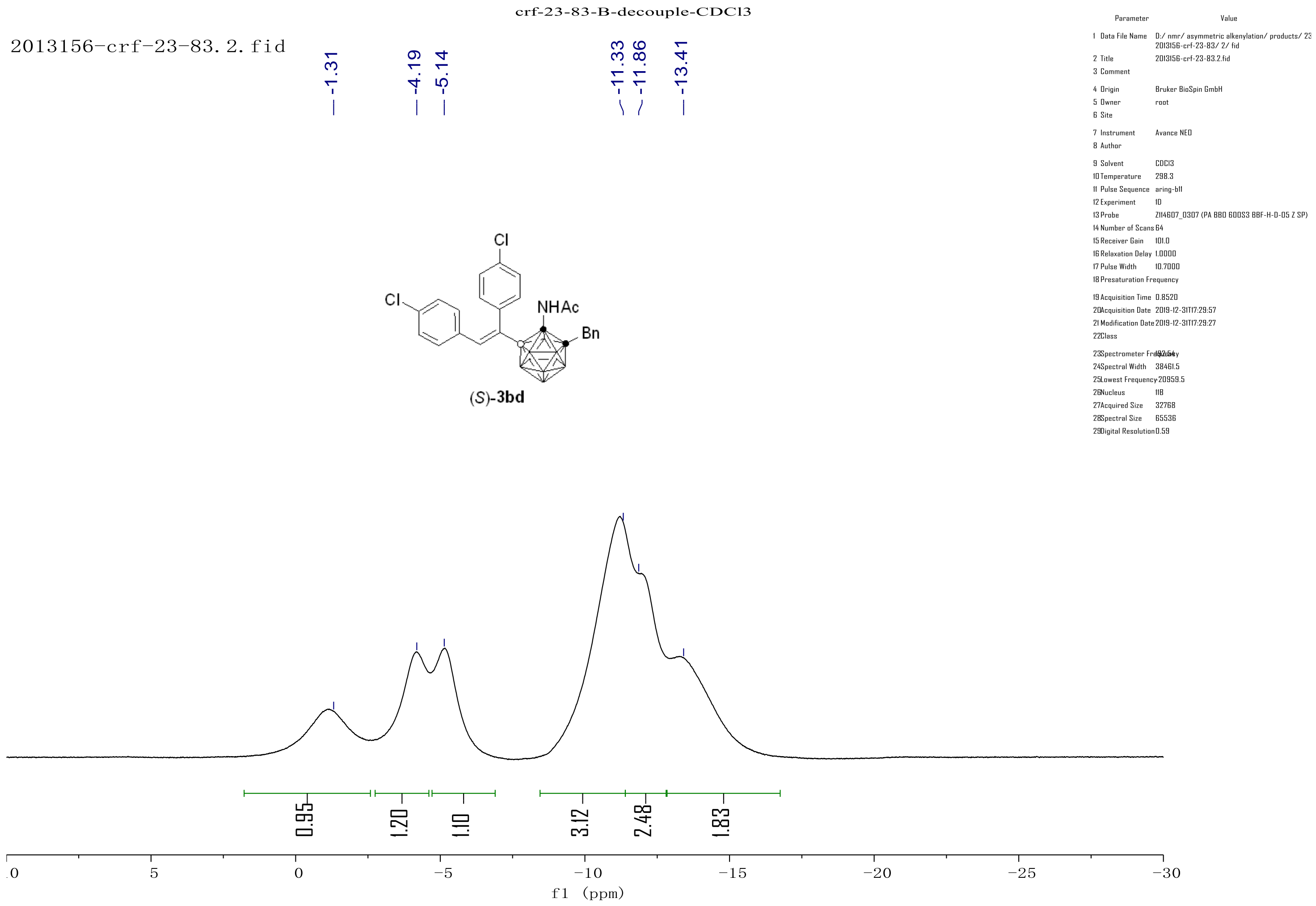

Supplementary Figure 85. ^{11}B NMR of (*S*)-**3bd**.

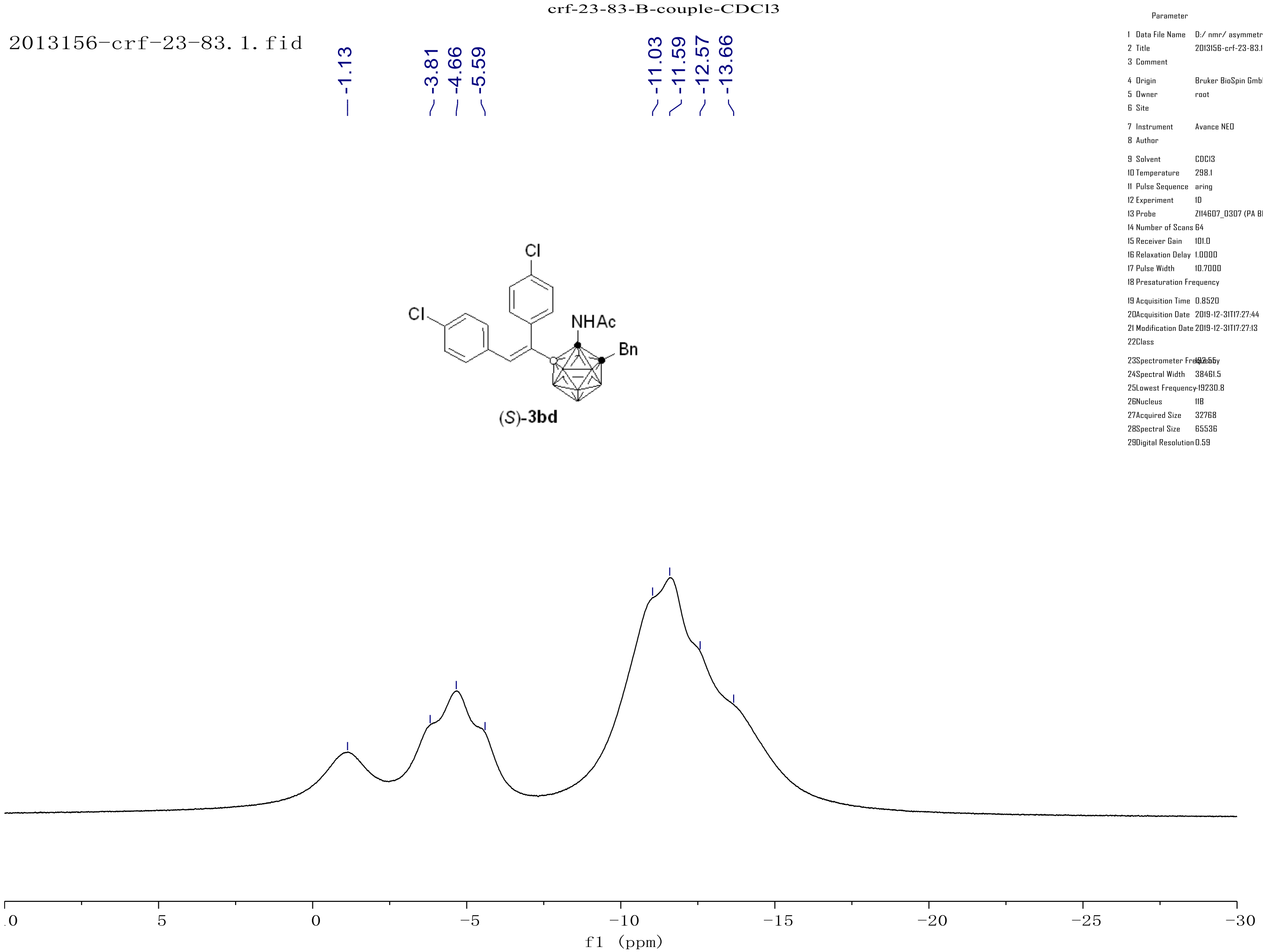

	Parameter	Value
1	Data File Name	D:/ nmr/ asymmetric alkenylation/ products/ 23-83BnMe-(
2	Title	2013156-crf-23-83.1.fid
3	Comment	
4	Origin	Bruker BioSpin GmbH
5	Owner	root
6	Site	
7	Instrument	Avance NEO
8	Author	
9	Solvent	CDCl3
10	Temperature	298.1
11	Pulse Sequence	aring
12	Experiment	1D
13	Probe	Z114607_0307 (PA BBO 600S3 BBF-H-D-05 Z SP)
14	Number of Scans	64
15	Receiver Gain	101.0
16	Relaxation Delay	1.0000
17	Pulse Width	10.7000
18	Presaturation Frequency	
19	Acquisition Time	0.8520
20	Acquisition Date	2019-12-31T17:27:44
21	Modification Date	2019-12-31T17:27:13
22	Class	
23	Spectrometer Frequency	192.55
24	Spectral Width	38461.5
25	Lowest Frequency	-19230.8
26	Nucleus	11B
27	Acquired Size	32768
28	Spectral Size	65536
29	Digital Resolution	0.59

Supplementary Figure 86. [11]H NMR of (*S*)-**3be**.

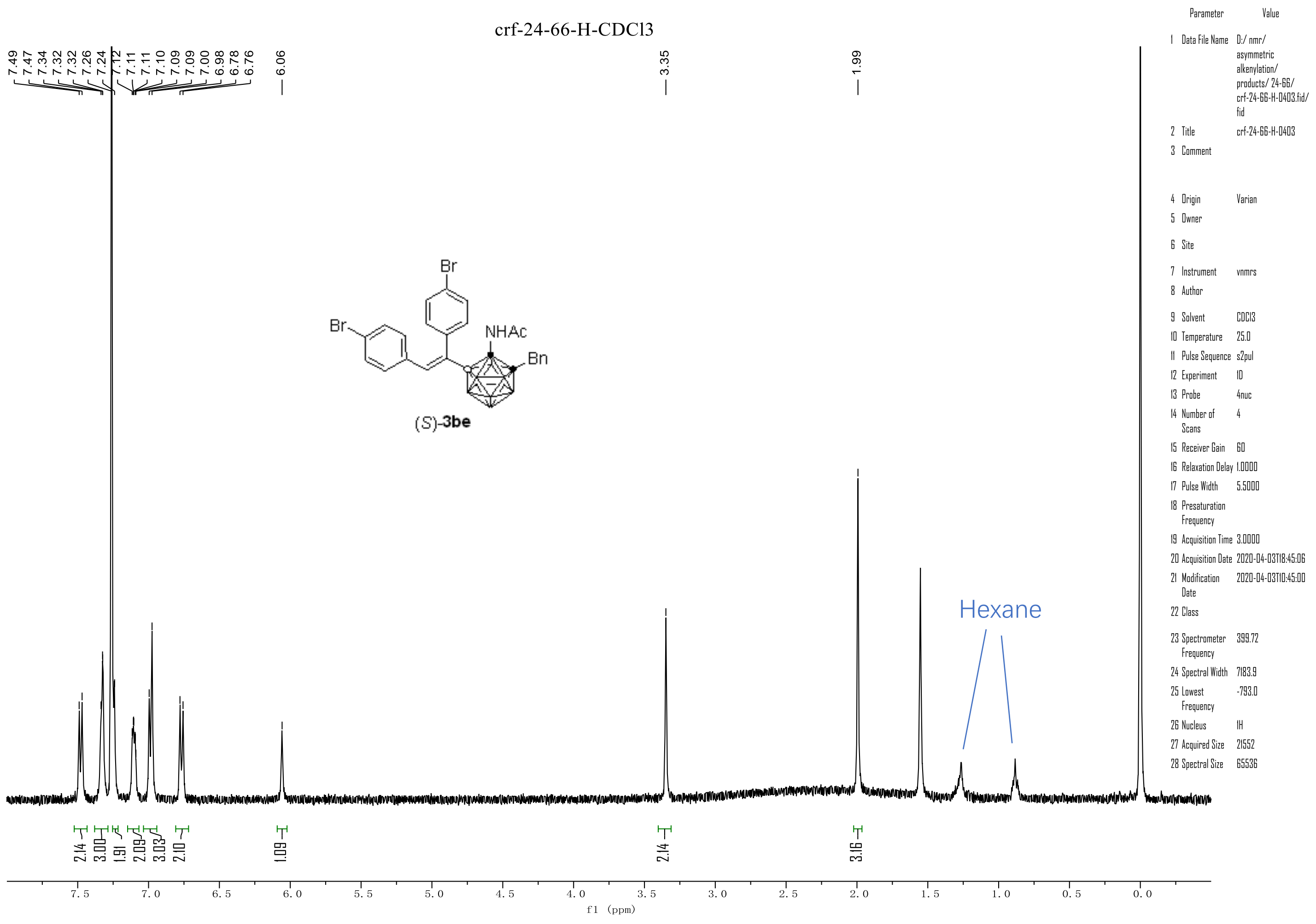

Supplementary Figure 87. $^{13}C\{^{1}H\}$ NMR of (*S*)-**3be**.

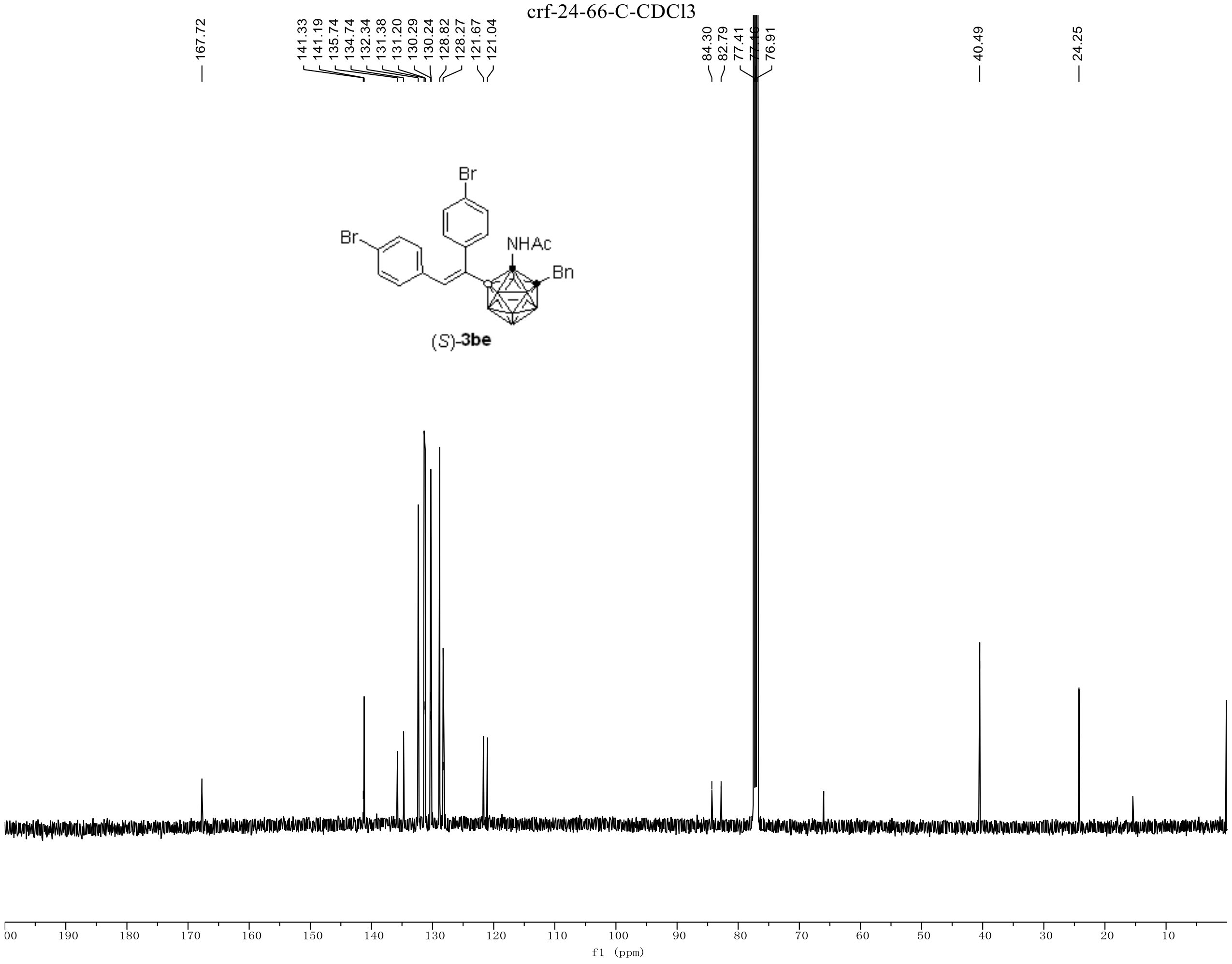

	Parameter	Value
1	Data File Name	D:/ nmr/ asymmetric alkenylation/ products/ 24-66/ 2013156c-crf-24-66/ 2013156c-crf-24-66/ 1/ fid
2	Title	2013156c-crf-24-66.1.fid
3	Comment	
4	Origin	Bruker BioSpin GmbH
5	Owner	nmrsu
6	Site	
7	Instrument	Avance
8	Author	
9	Solvent	CDCl3
10	Temperature	298.2
11	Pulse Sequence	zgpg30
12	Experiment	1D
13	Probe	Z150355_0001 (CP BBO 500S1 BB-H&F-D-05 Z)
14	Number of Scans	82
15	Receiver Gain	101.0
16	Relaxation Delay	2.0000
17	Pulse Width	10.0000
18	Presaturation Frequency	
19	Acquisition Time	0.8847
20	Acquisition Date	2020-04-07T13:19:17
21	Modification Date	2020-04-07T13:18:57
22	Class	
23	Spectrometer Frequency	125.78
24	Spectral Width	37037.0
25	Lowest Frequency	-8454.0
26	Nucleus	13C
27	Acquired Size	32768
28	Spectral Size	65536

Supplementary Figure 88. $^{11}B\{^{1}H\}$ NMR of (*S*)-**3be**.

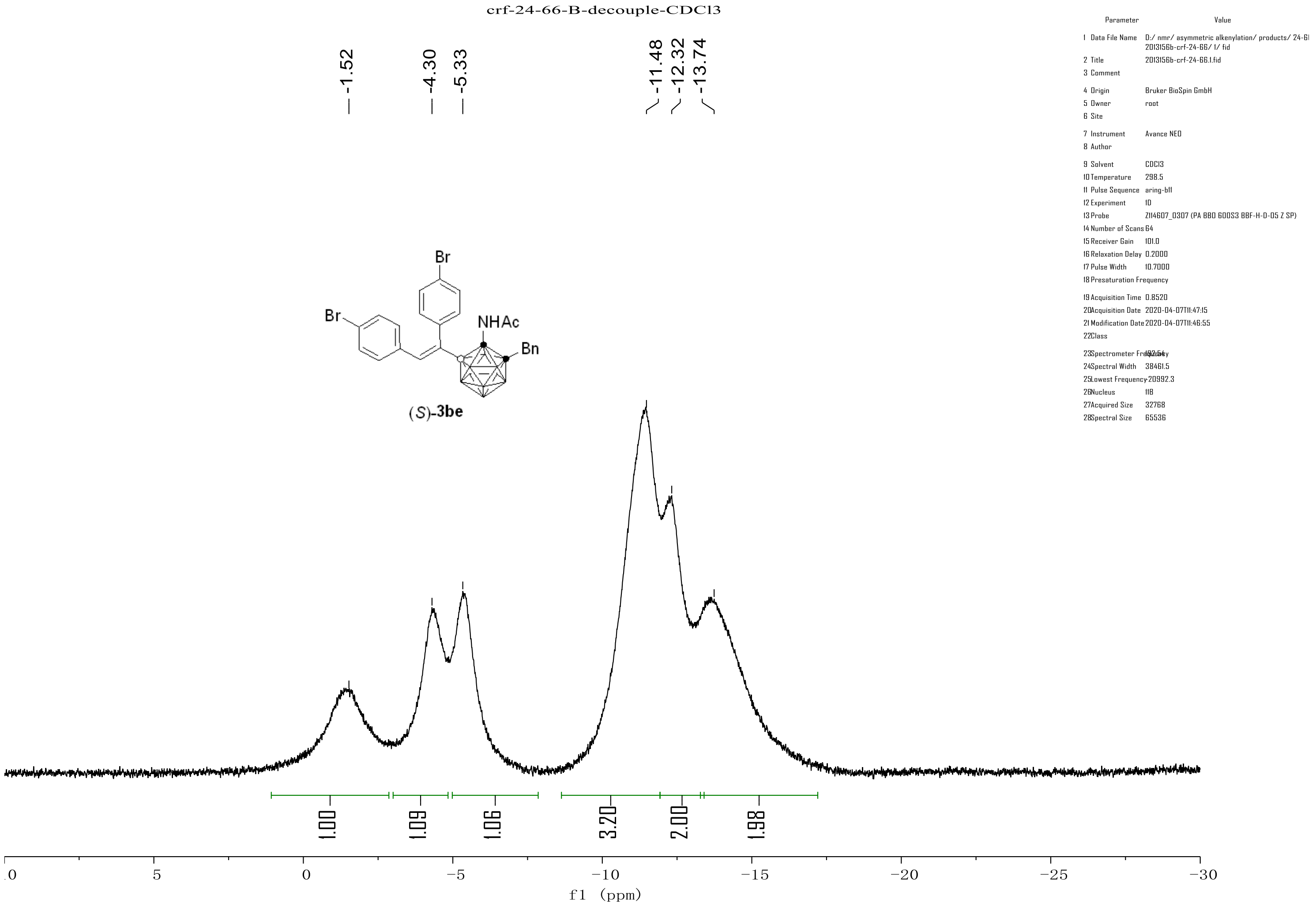

Supplementary Figure 89. ^{11}B NMR of (*S*)-**3be**.

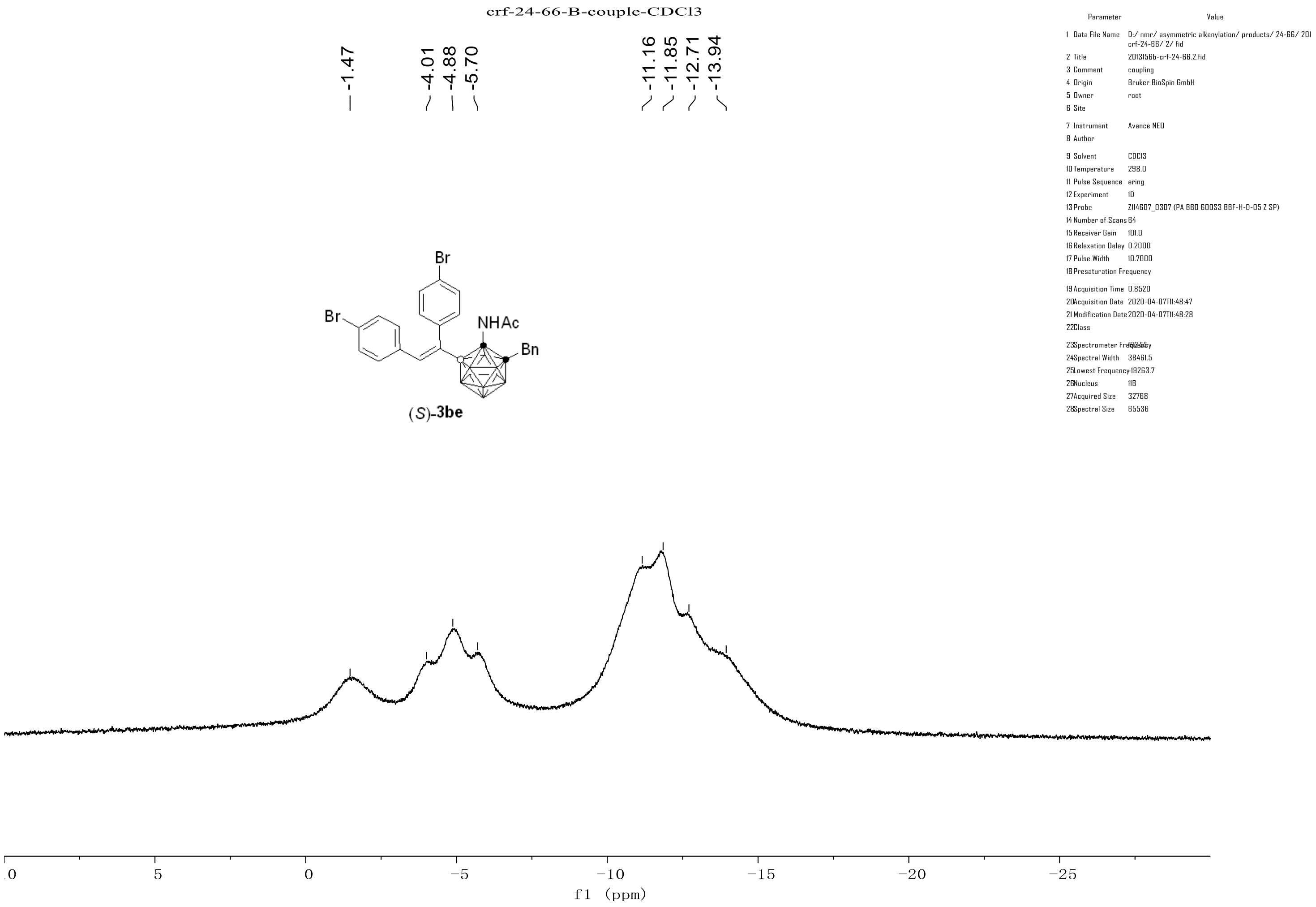

Supplementary Figure 90. ^{1}H NMR of (*S*)-**3bf**.

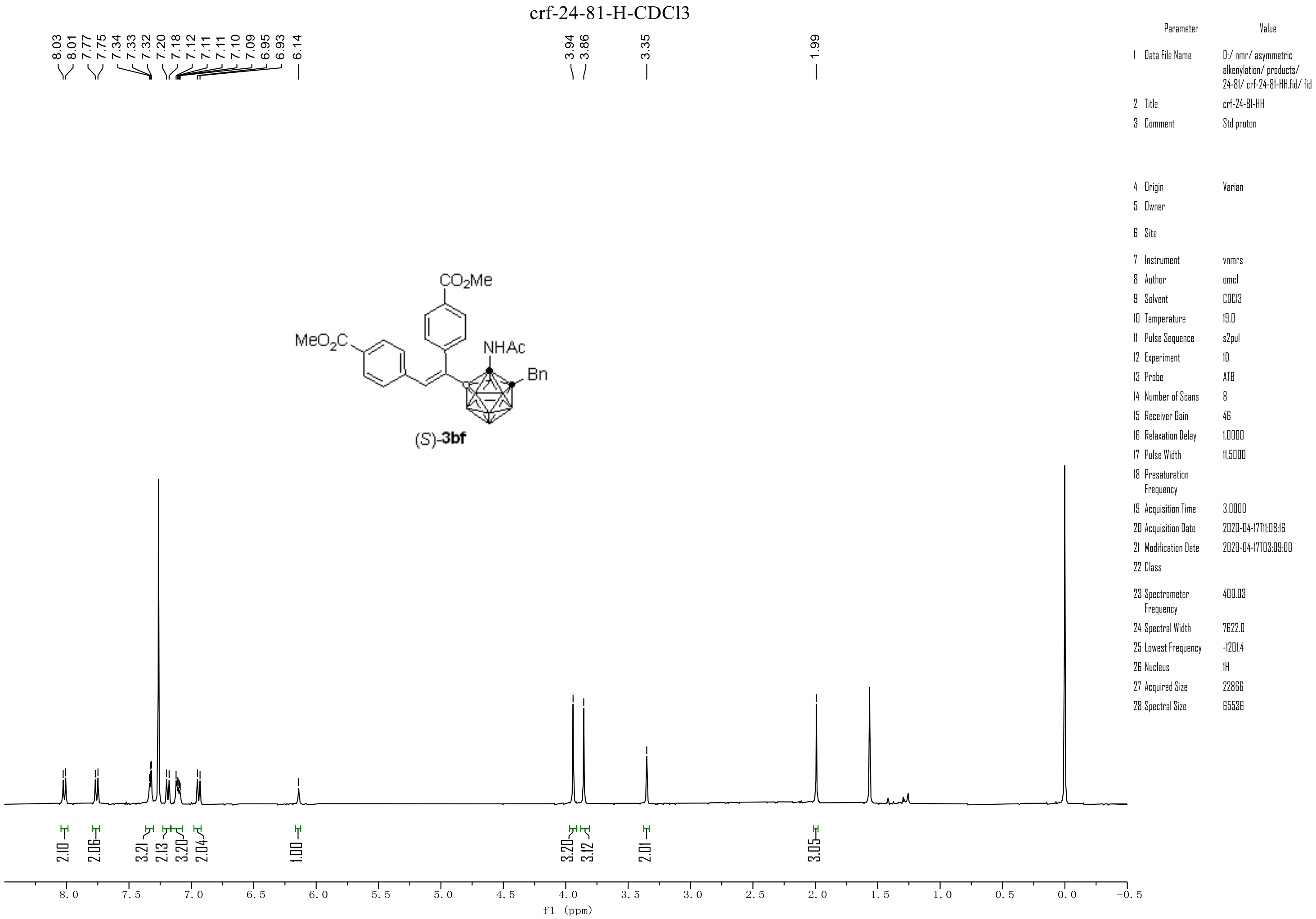

	Parameter	Value
1	Data File Name	D:/ nmr/ asymmetric alkenylation/ products/ 24-81/ crf-24-81-HH.fid/ fid
2	Title	crf-24-81-HH
3	Comment	Std proton
4	Origin	Varian
5	Owner	
6	Site	
7	Instrument	vnmrs
8	Author	omcl
9	Solvent	CDCl3
10	Temperature	19.0
11	Pulse Sequence	s2pul
12	Experiment	1D
13	Probe	ATB
14	Number of Scans	8
15	Receiver Gain	46
16	Relaxation Delay	1.0000
17	Pulse Width	11.5000
18	Presaturation Frequency	
19	Acquisition Time	3.0000
20	Acquisition Date	2020-04-17T11:08:16
21	Modification Date	2020-04-17T03:09:00
22	Class	
23	Spectrometer Frequency	400.03
24	Spectral Width	7622.0
25	Lowest Frequency	-1201.4
26	Nucleus	1H
27	Acquired Size	22866
28	Spectral Size	65536

Supplementary Figure 91. $^{13}C\{^1H\}$ NMR of (*S*)-**3bf**.

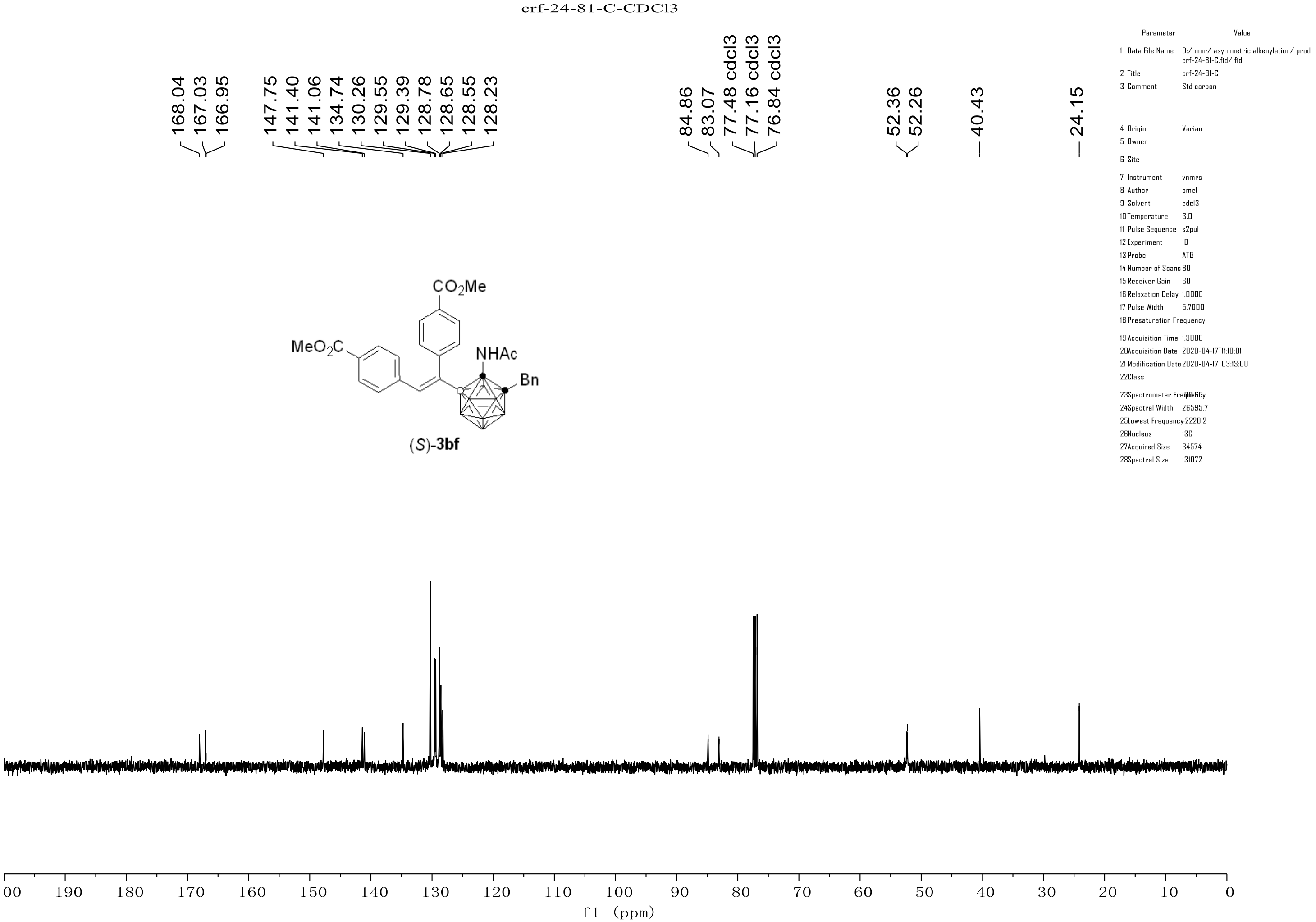

Supplementary Figure 92. $^{11}B\{^1H\}$ NMR of (*S*)-**3bf**.

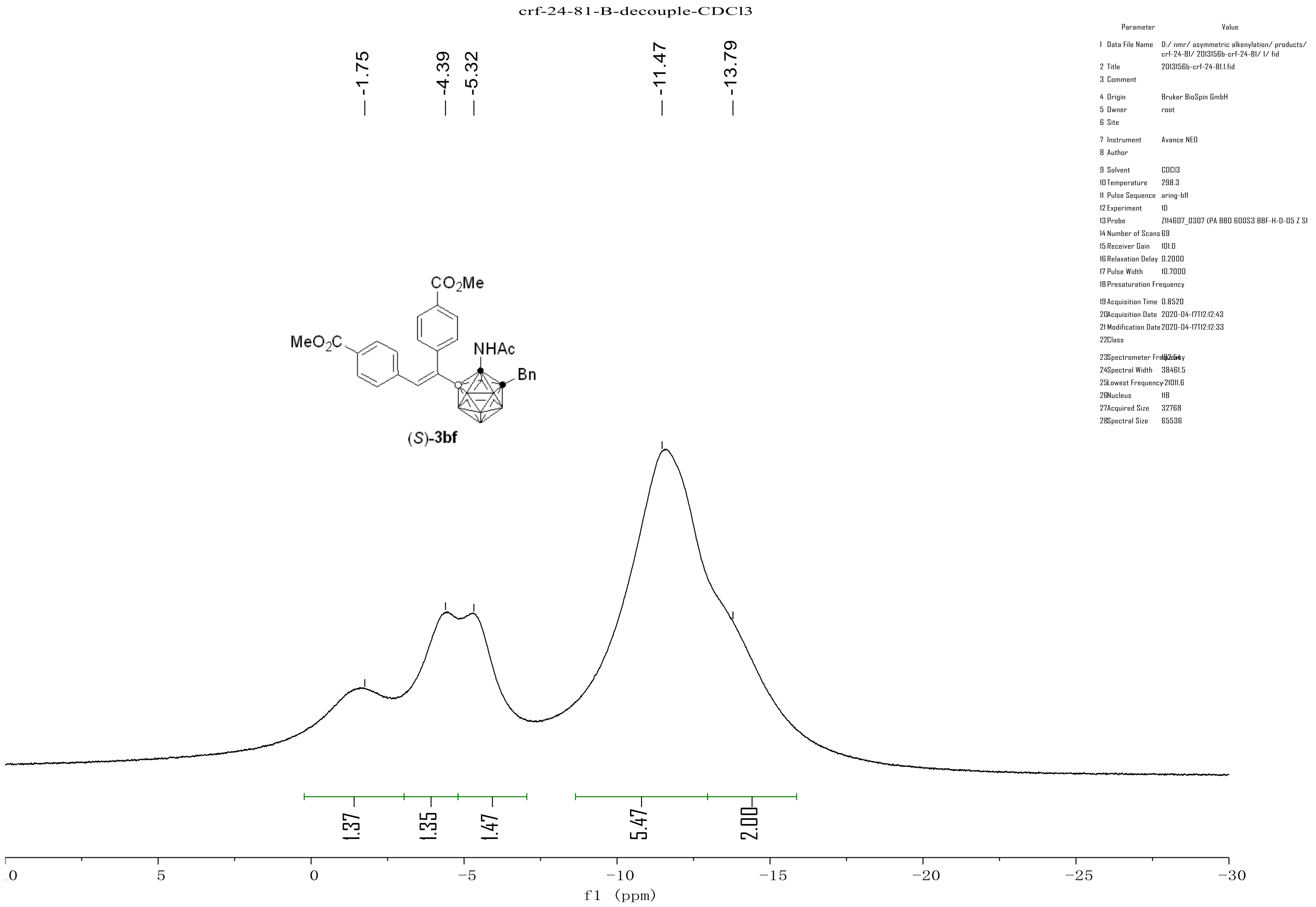

Supplementary Figure 93. ^{11}B NMR of (*S*)-**3bf**.

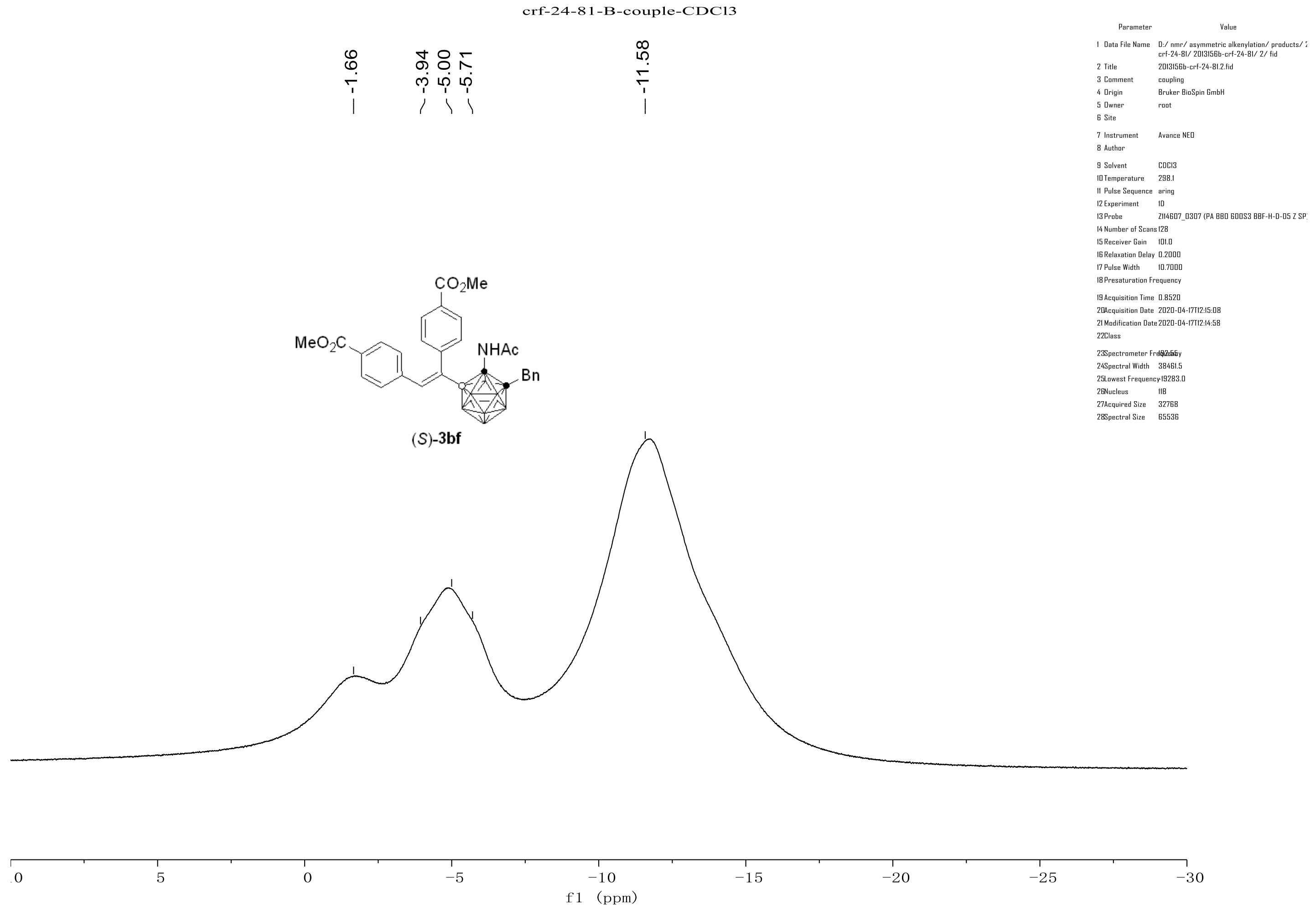

Supplementary Figure 94. ^{1}H NMR of (*S*)-**3bg**.

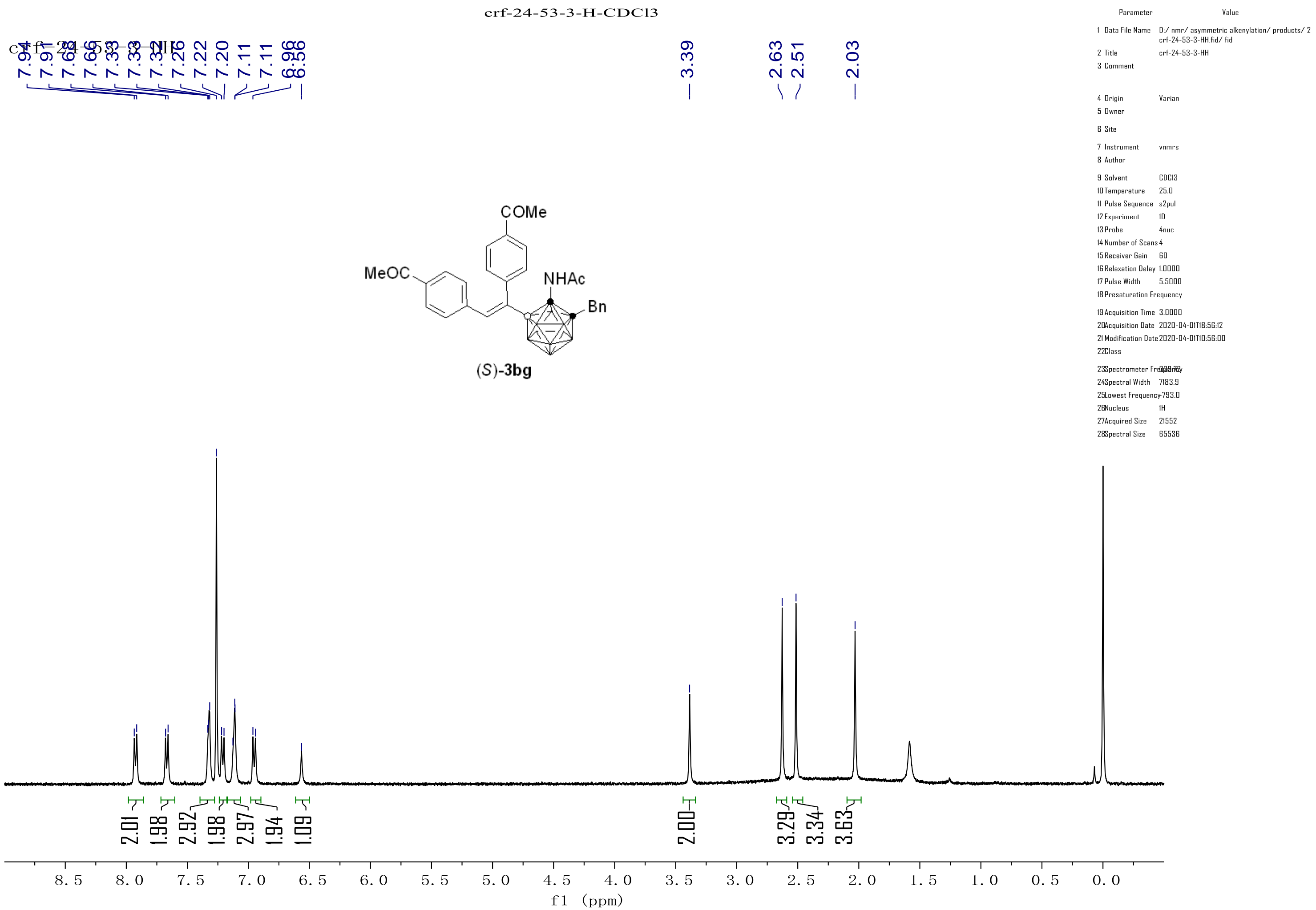

Supplementary Figure 95. $^{13}C\{^{1}H\}$ NMR of (*S*)-**3bg**.

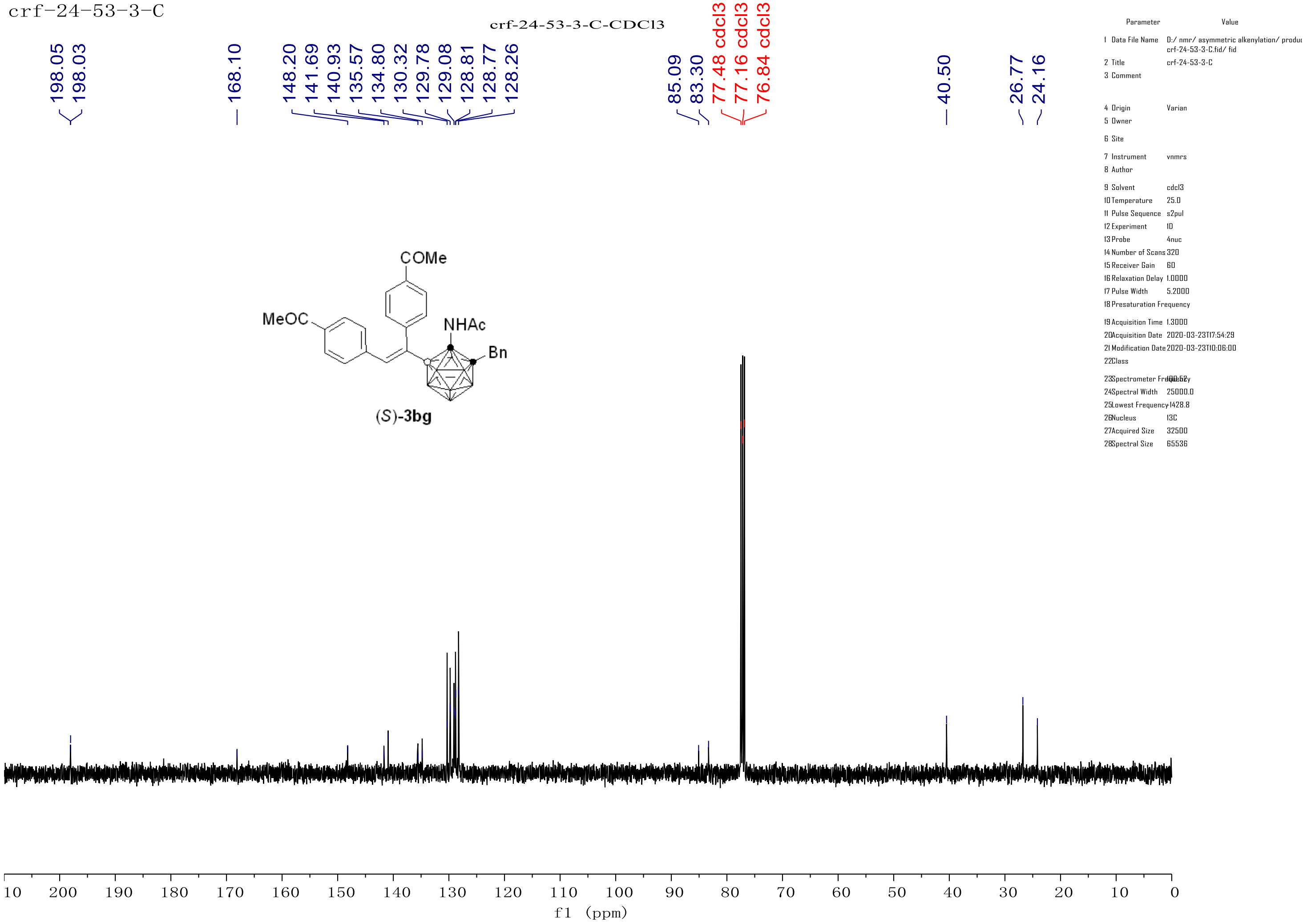

Supplementary Figure 96. $^{11}B\{^{1}H\}$ NMR of (*S*)-**3bg**.

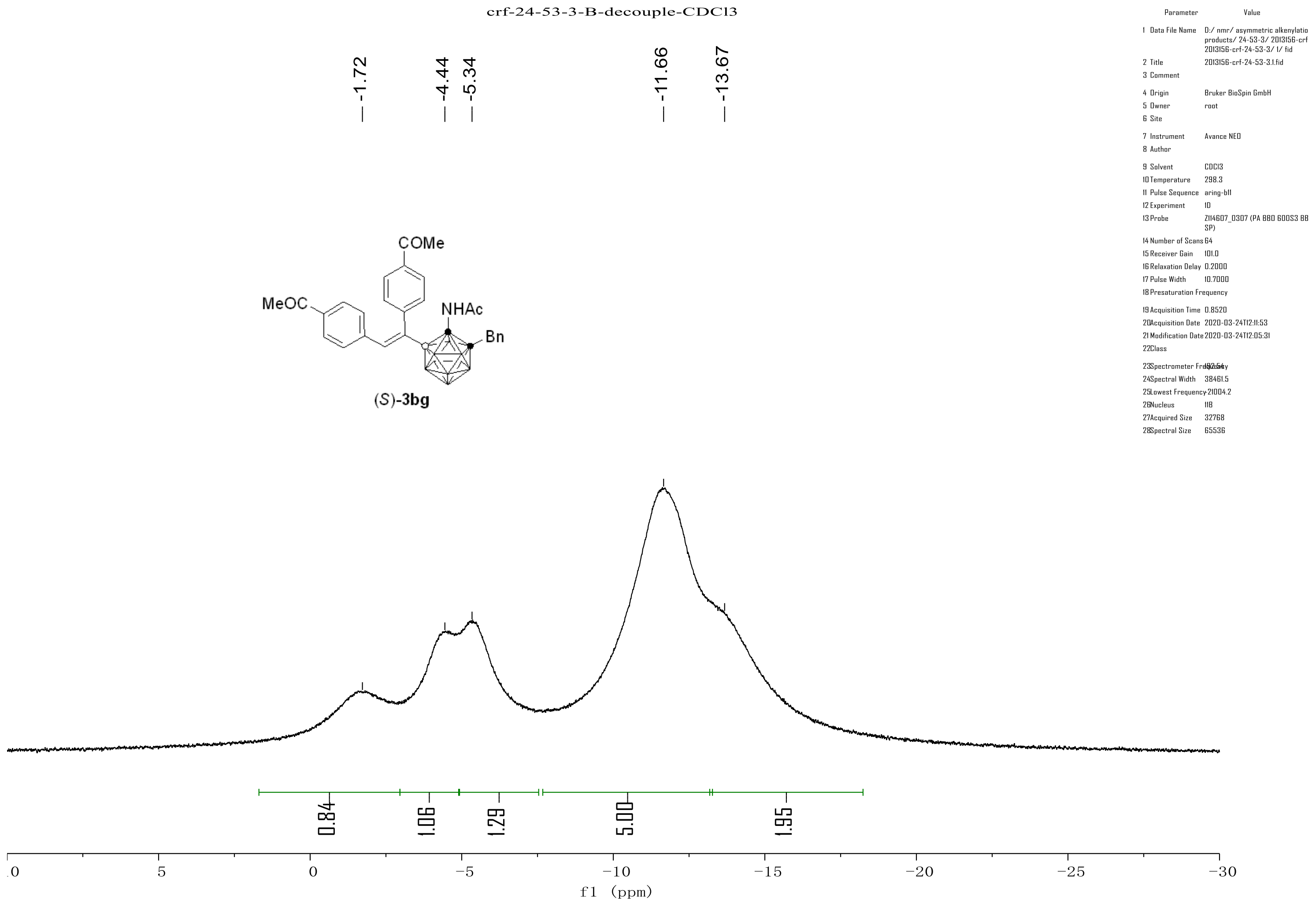

Supplementary Figure 97. ^{11}B NMR of (*S*)-**3bg**.

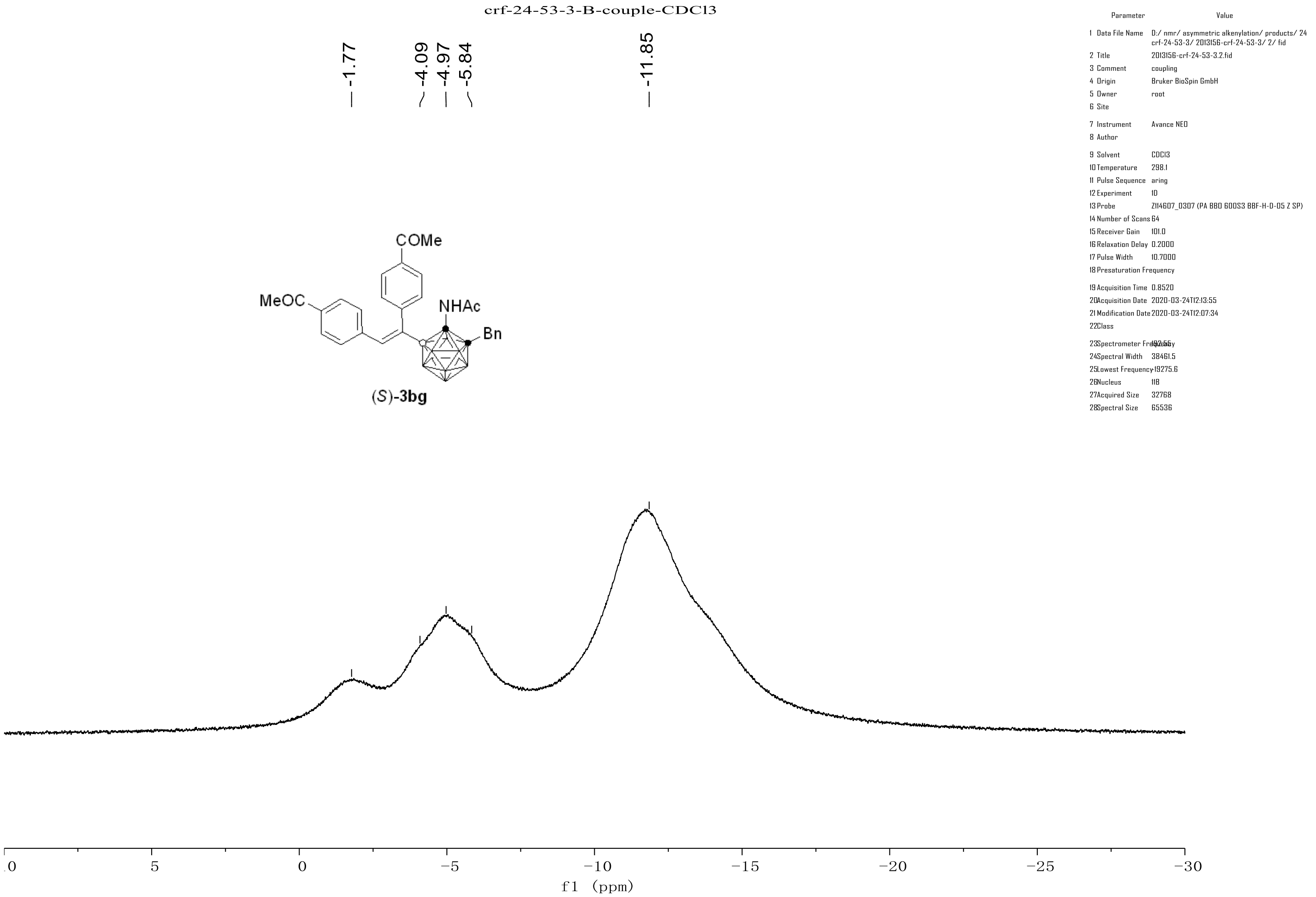

Supplementary Figure 98. ^{1}H NMR of (*S*)-**3bh**.

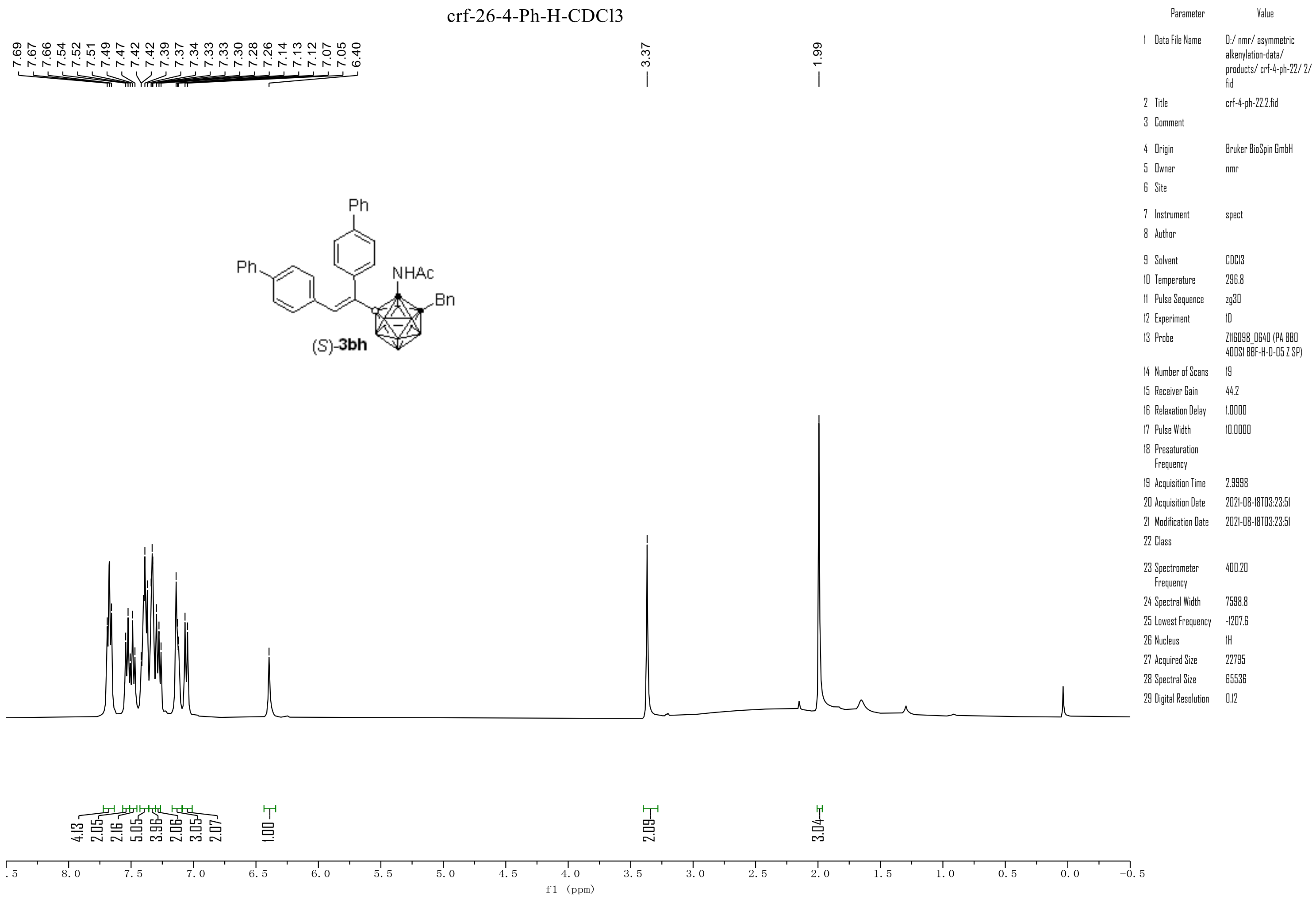

Supplementary Figure 99. $^{13}C\{^1H\}$ NMR of (*S*)-**3bh**.

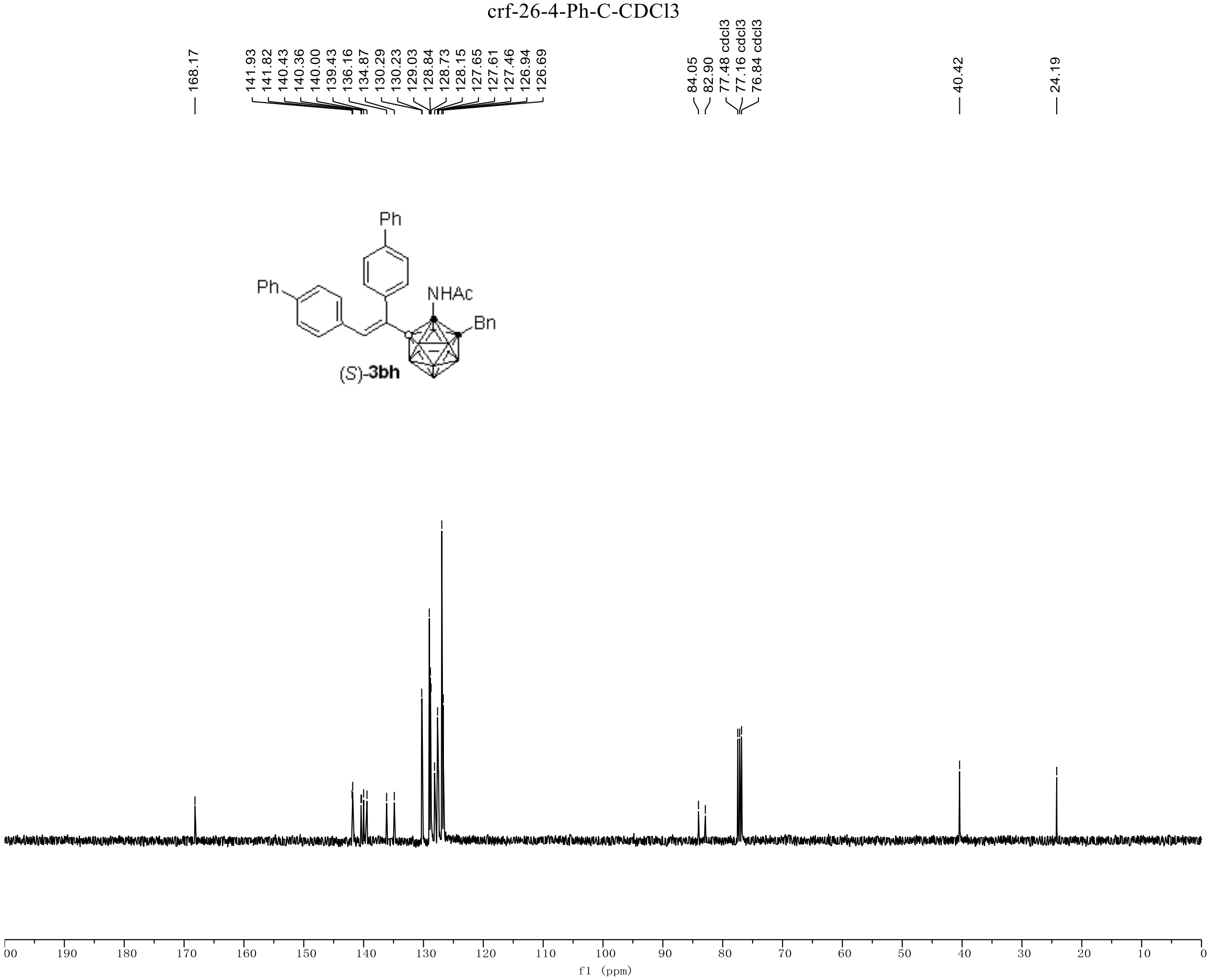

	Parameter	Value
1	Data File Name	D:/ nmr/ asymmetric alkenylation-data/ products/ zhf-4-ph-c2.fid/ fid
2	Title	zhf-4-ph-c2
3	Comment	Std carbon
4	Origin	Varian
5	Owner	
6	Site	
7	Instrument	vnmrs
8	Author	omc1
9	Solvent	cdcl3
10	Temperature	30.0
11	Pulse Sequence	s2pul
12	Experiment	1D
13	Probe	ATB
14	Number of Scans	80
15	Receiver Gain	30
16	Relaxation Delay	1.0000
17	Pulse Width	5.7000
18	Presaturation Frequency	
19	Acquisition Time	1.3000
20	Acquisition Date	2021-08-24T12:23:09
21	Modification Date	2021-08-23T16:26:00
22	Class	
23	Spectrometer Frequency	100.60
24	Spectral Width	26595.7
25	Lowest Frequency	-2224.5
26	Nucleus	13C
27	Acquired Size	34574
28	Spectral Size	131072
29	Digital Resolution	0.20

Supplementary Figure 100. $^{11}B\{^{1}H\}$ NMR of (*S*)-**3bh**.

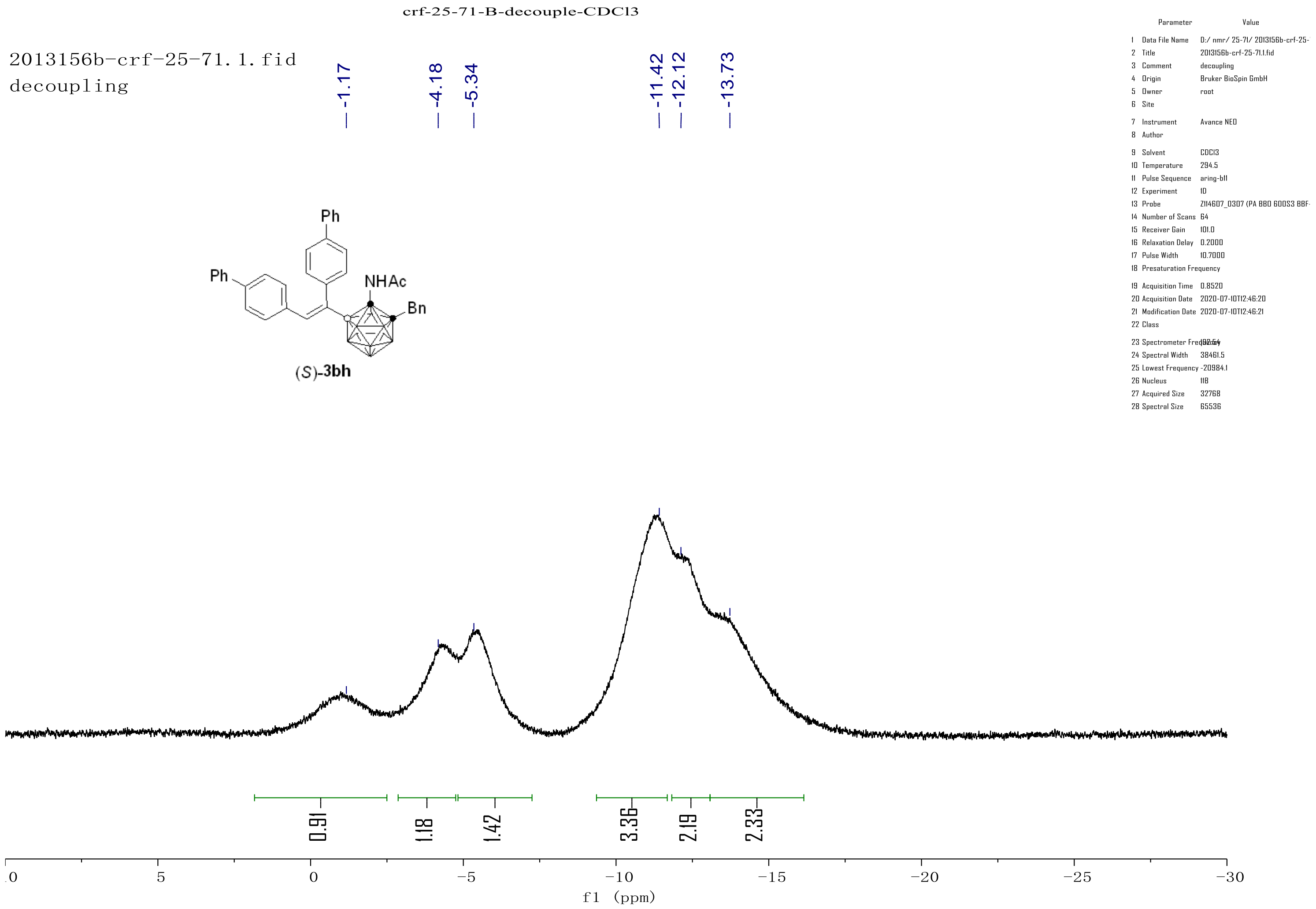

Supplementary Figure 101. ^{11}B NMR of (*S*)-**3bh**.

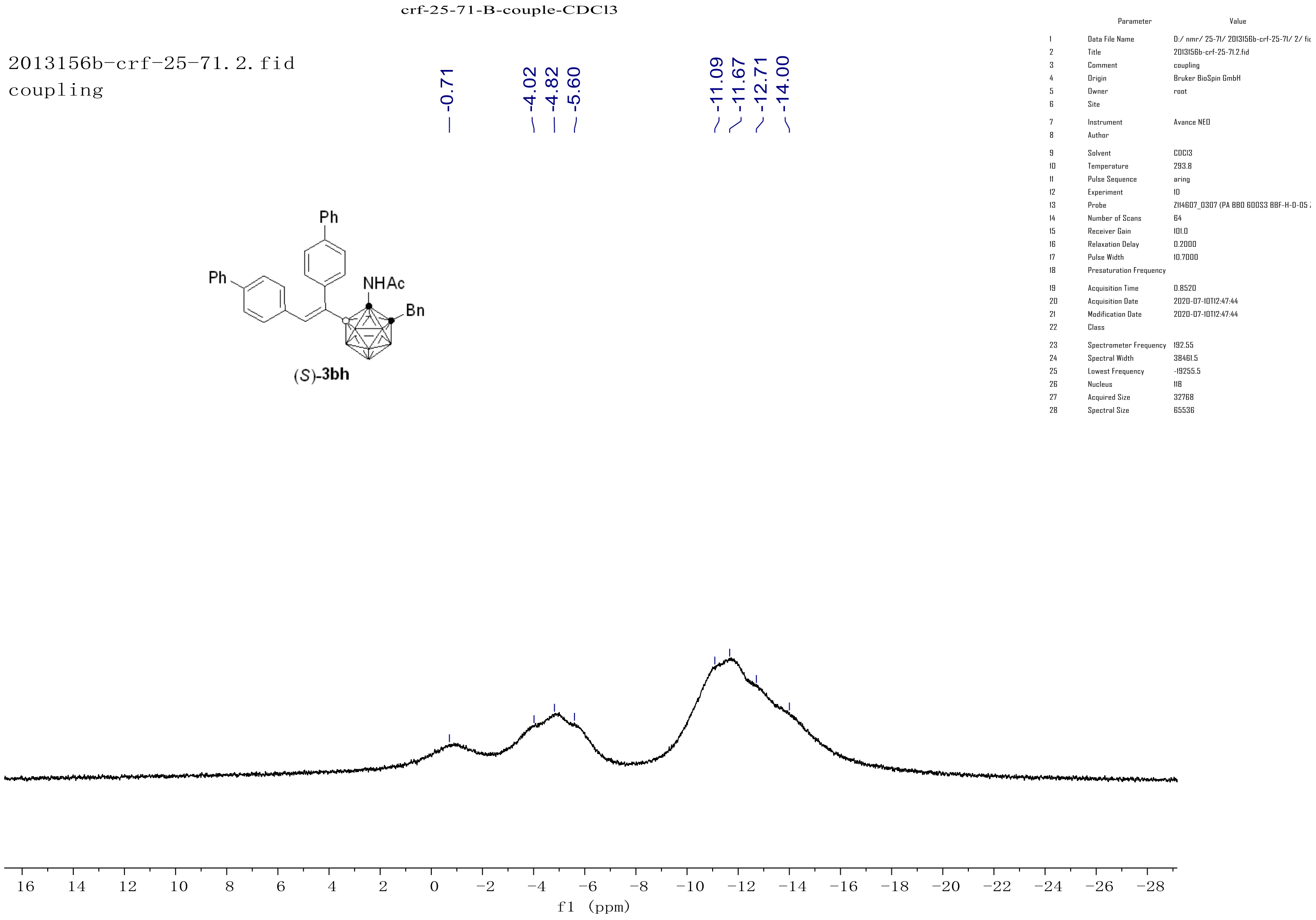

Supplementary Figure 102. ^{1}H NMR of (*S*)-**3bi**.

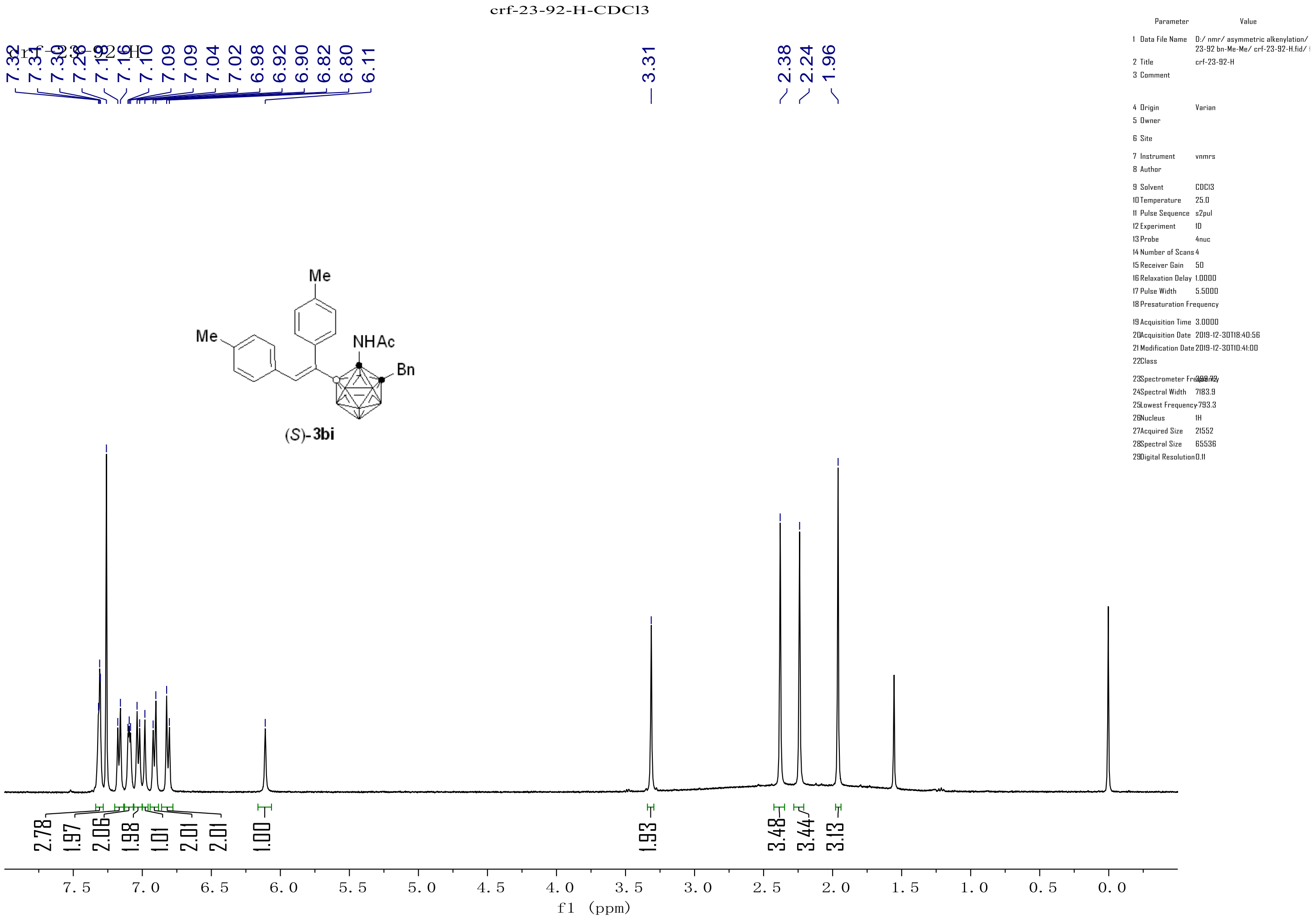

Supplementary Figure 103. $^{13}C\{^{1}H\}$ NMR of (*S*)-**3bi**.

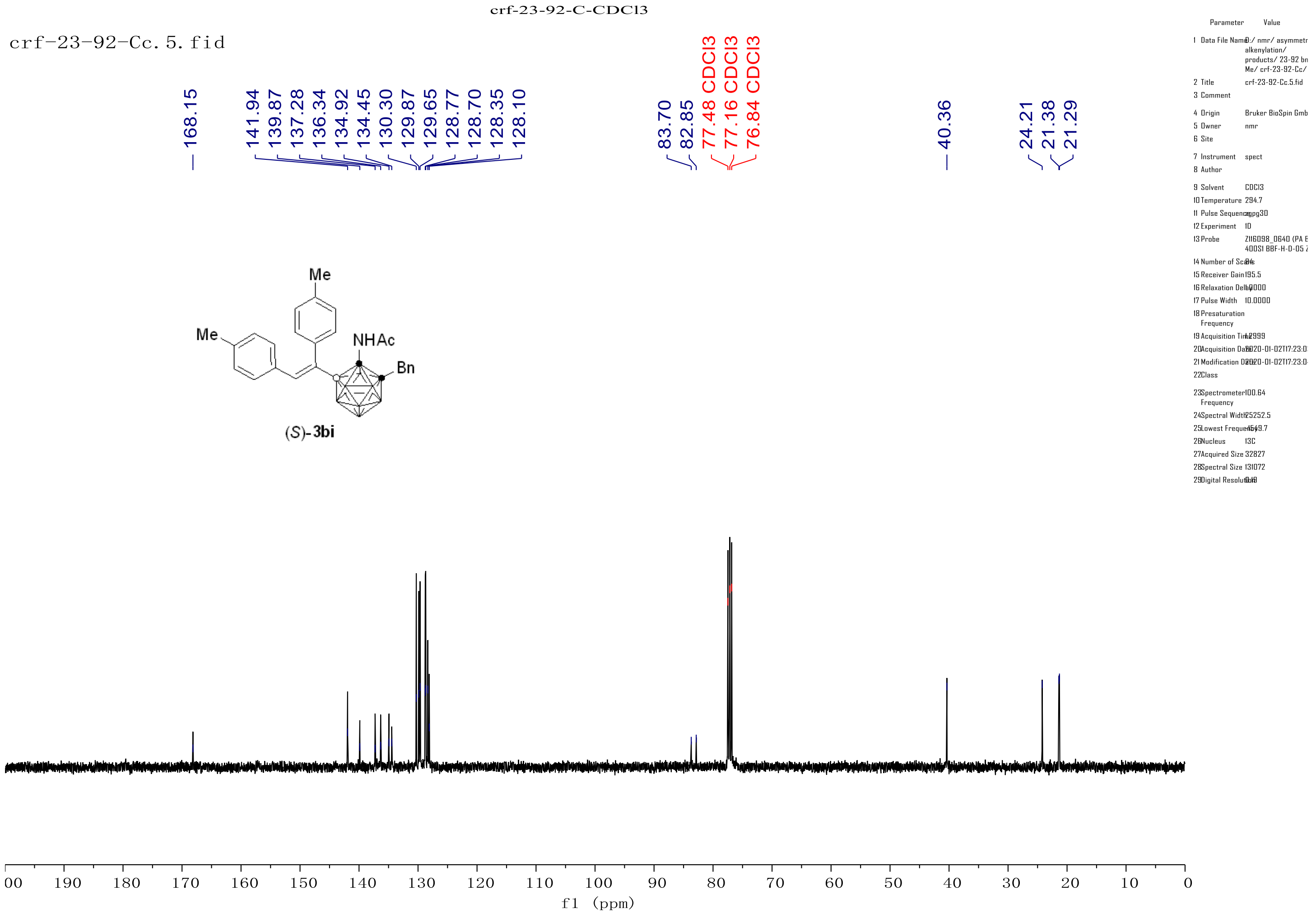

Supplementary Figure 104. $^{11}B\{^{1}H\}$ NMR of (*S*)-**3bi**.

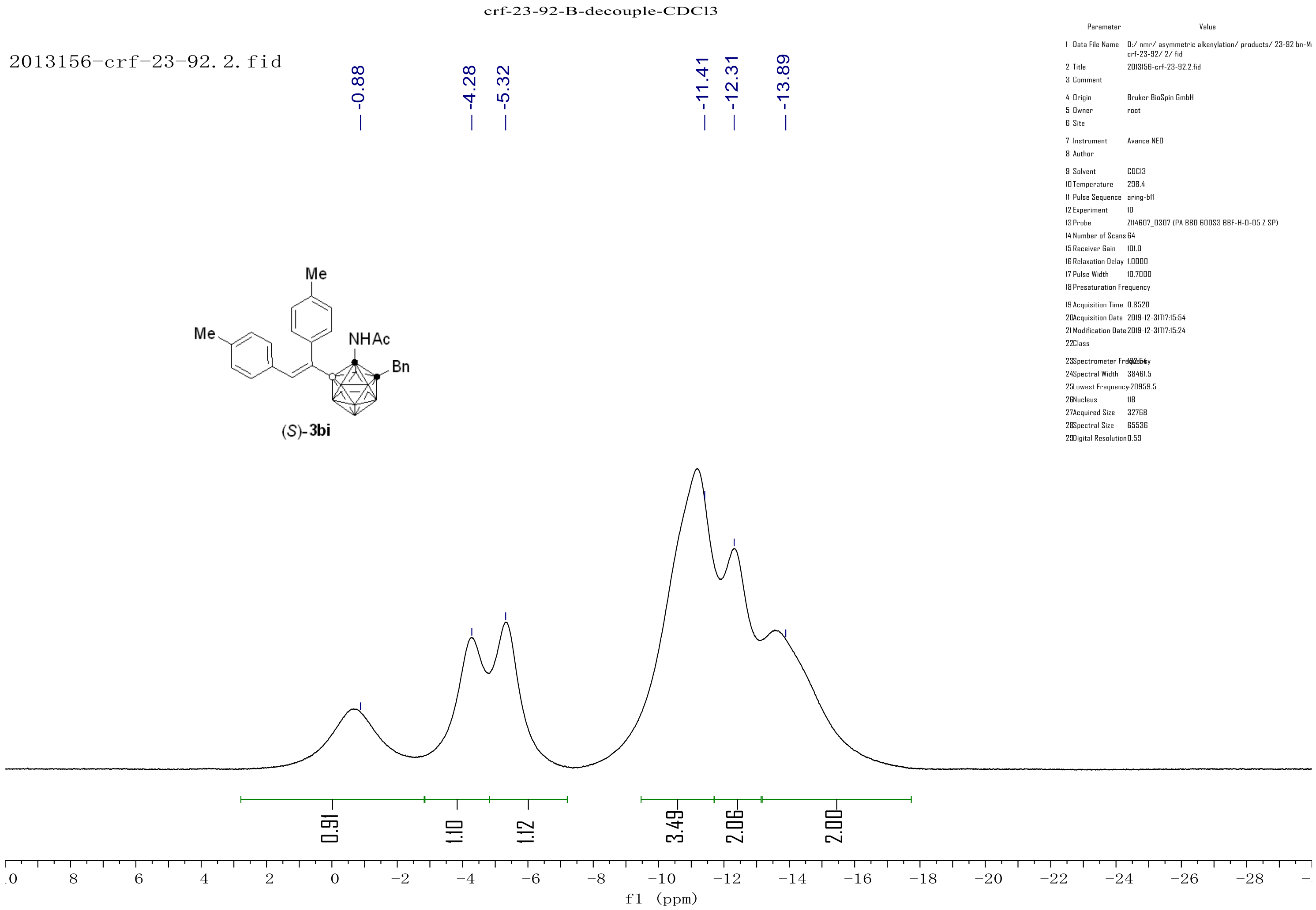

Supplementary Figure 105. ^{11}B NMR of (*S*)-**3bi**.

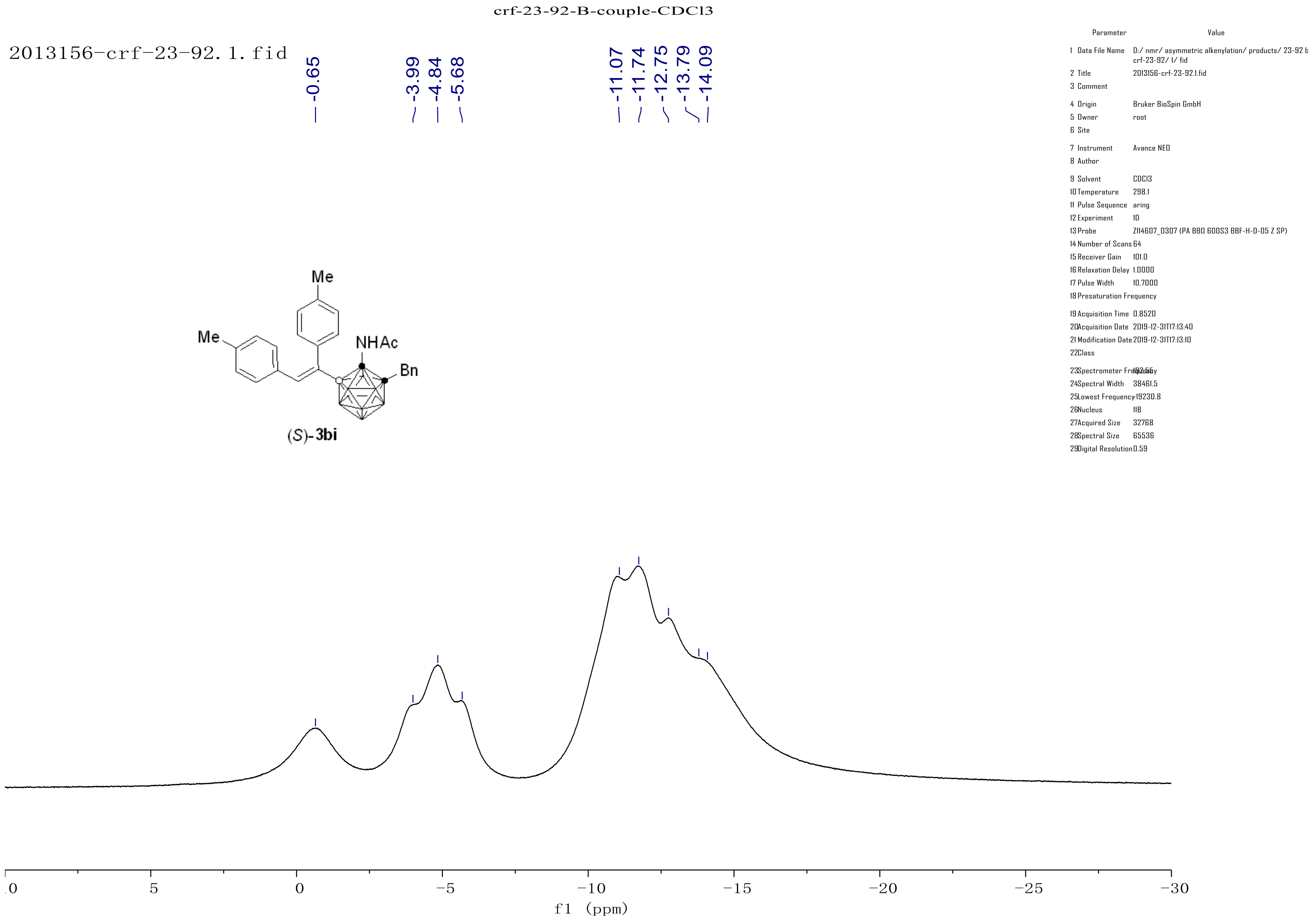

Supplementary Figure 106. ^{1}H NMR of (*S*)-**3bj**.

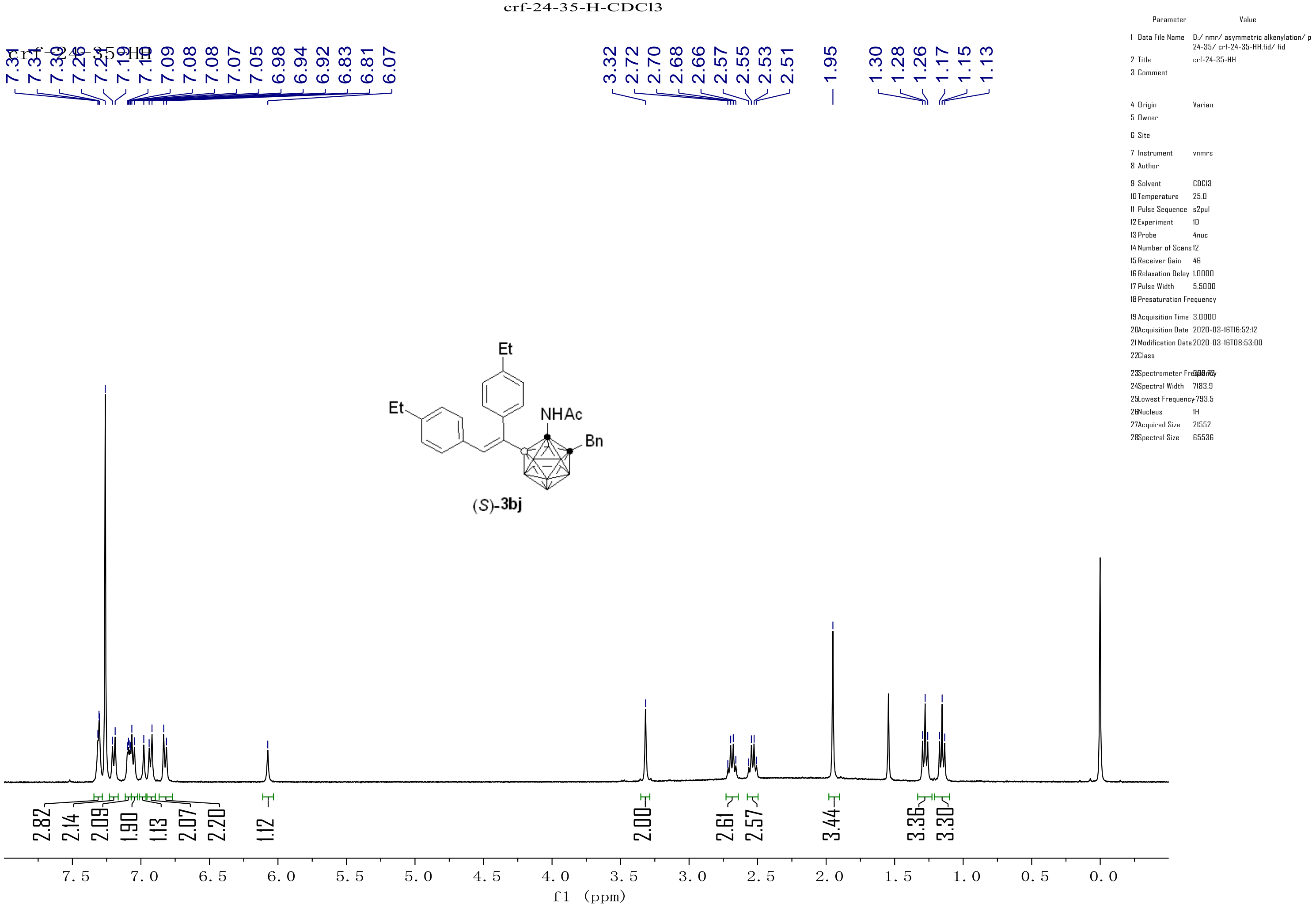

Supplementary Figure 107. $^{13}C\{^1H\}$ NMR of (*S*)-**3bj**.

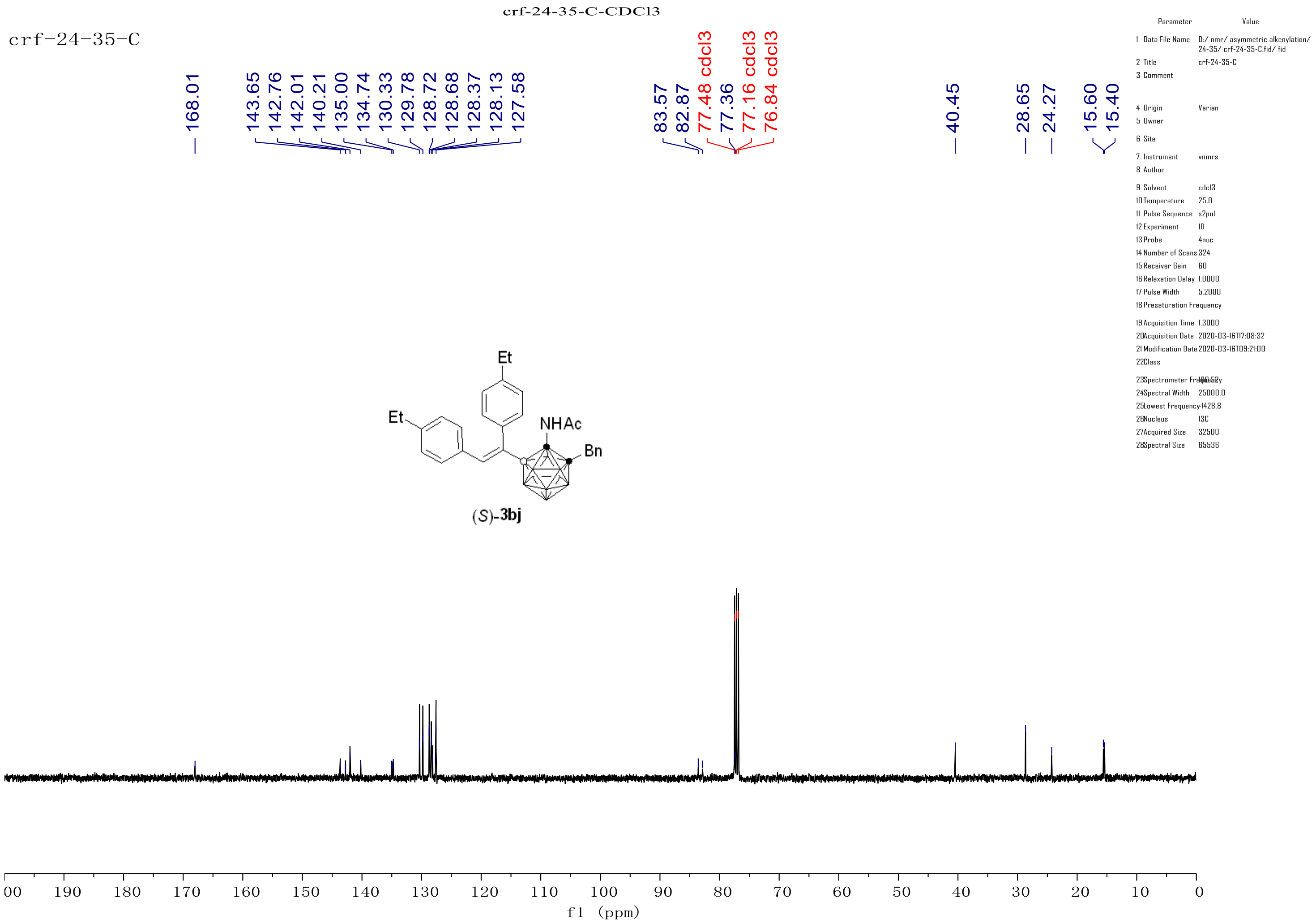

Supplementary Figure 108. $^{11}B\{^{1}H\}$ NMR of (*S*)-**3bj**.

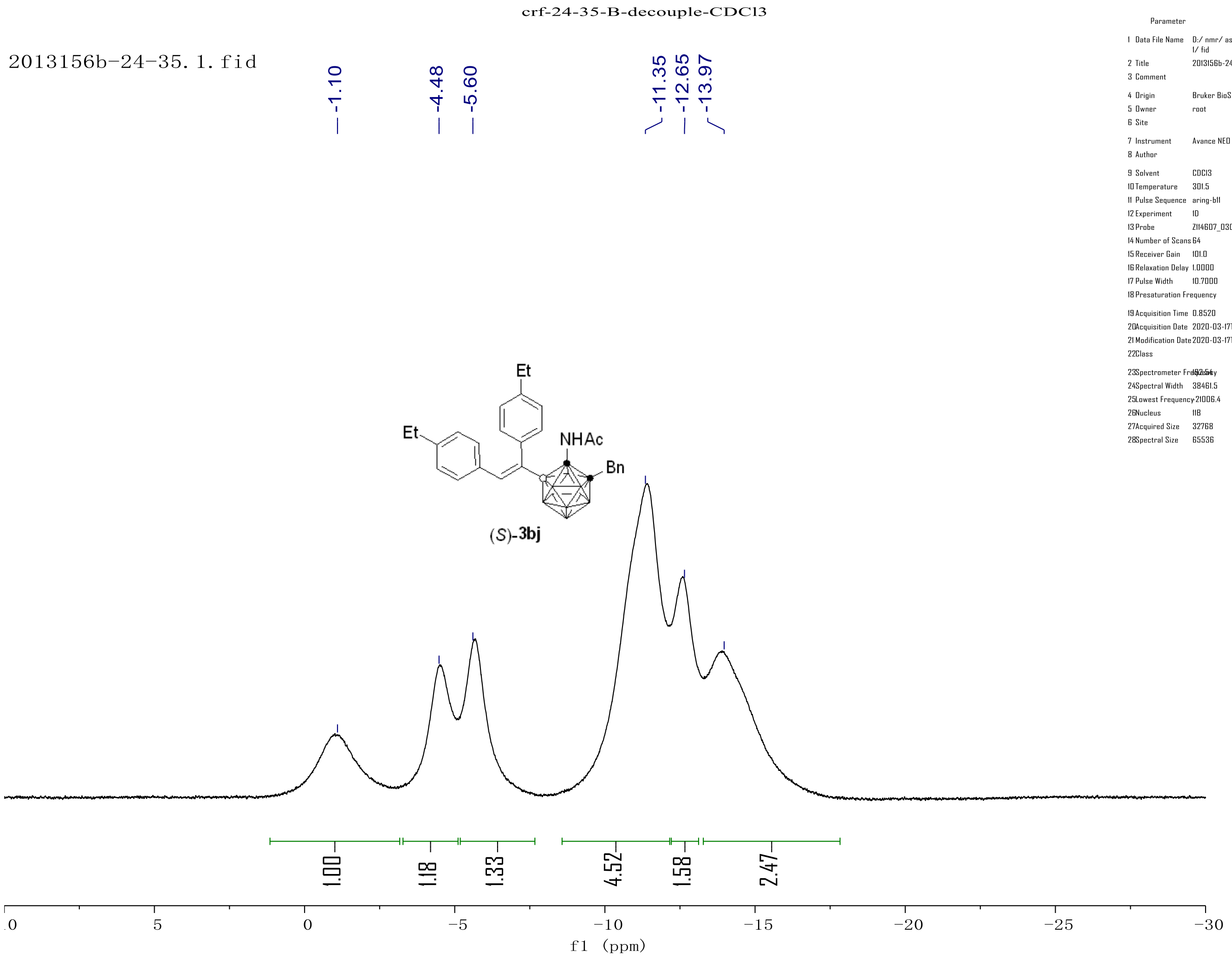

Parameter	Value
1 Data File Name	D:/ nmr/ asymmetric alkenylation/ products/ 24-1/ fid
2 Title	2013156b-24-35.1.fid
3 Comment	
4 Origin	Bruker BioSpin GmbH
5 Owner	root
6 Site	
7 Instrument	Avance NEO
8 Author	
9 Solvent	CDCl3
10 Temperature	301.5
11 Pulse Sequence	aring-b11
12 Experiment	1D
13 Probe	Z114607_0307 (PA BBO 600S3 BBF-H-D-05 Z SP)
14 Number of Scans	64
15 Receiver Gain	101.0
16 Relaxation Delay	1.0000
17 Pulse Width	10.7000
18 Presaturation Frequency	
19 Acquisition Time	0.8520
20 Acquisition Date	2020-03-17T12:03:18
21 Modification Date	2020-03-17T11:57:32
22 Class	
23 Spectrometer Frequency	192.54
24 Spectral Width	38461.5
25 Lowest Frequency	-21006.4
26 Nucleus	11B
27 Acquired Size	32768
28 Spectral Size	65536

Supplementary Figure 109. ^{11}B NMR of (*S*)-**3bj**.

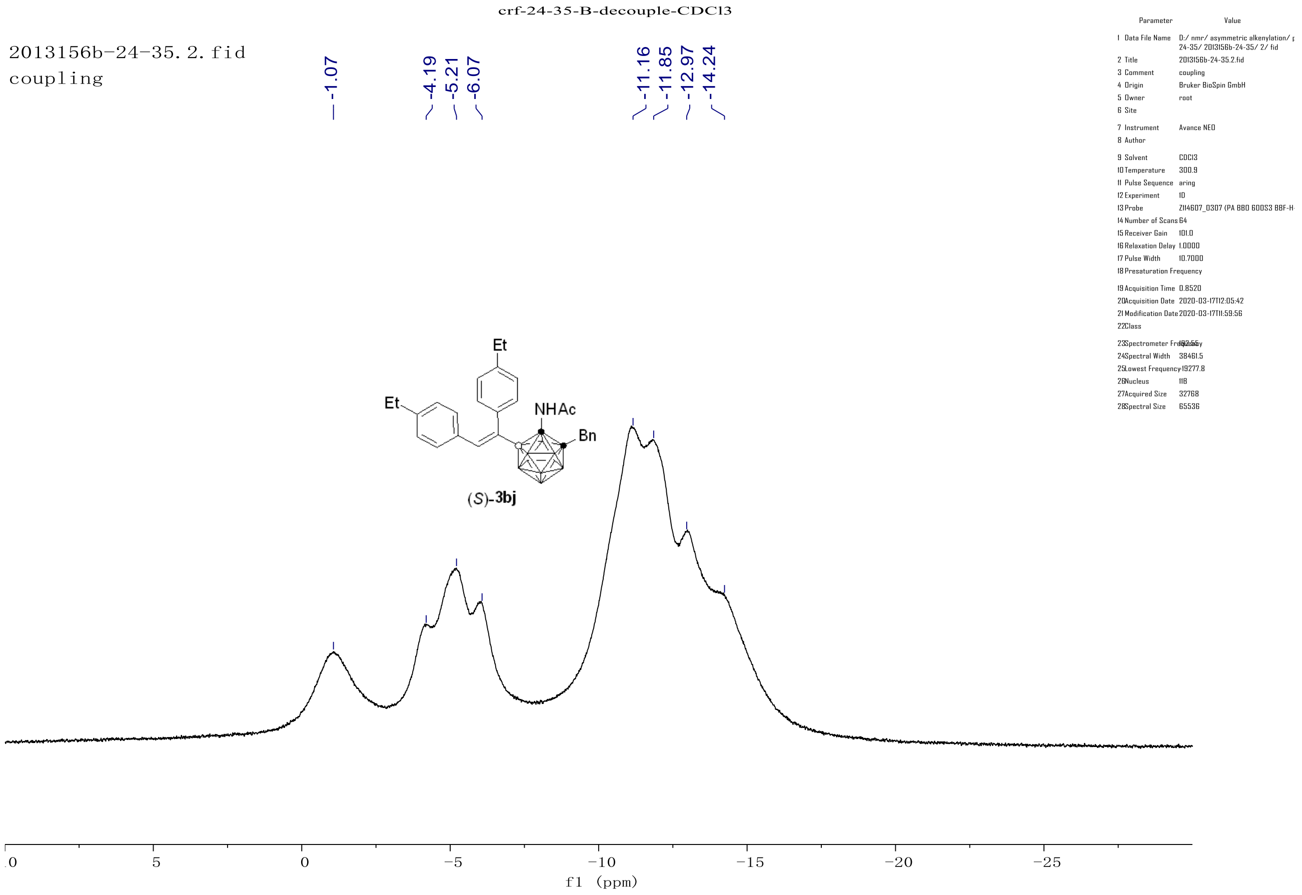

Supplementary Figure 110. ^{1}H NMR of (*S*)-**3bk**.

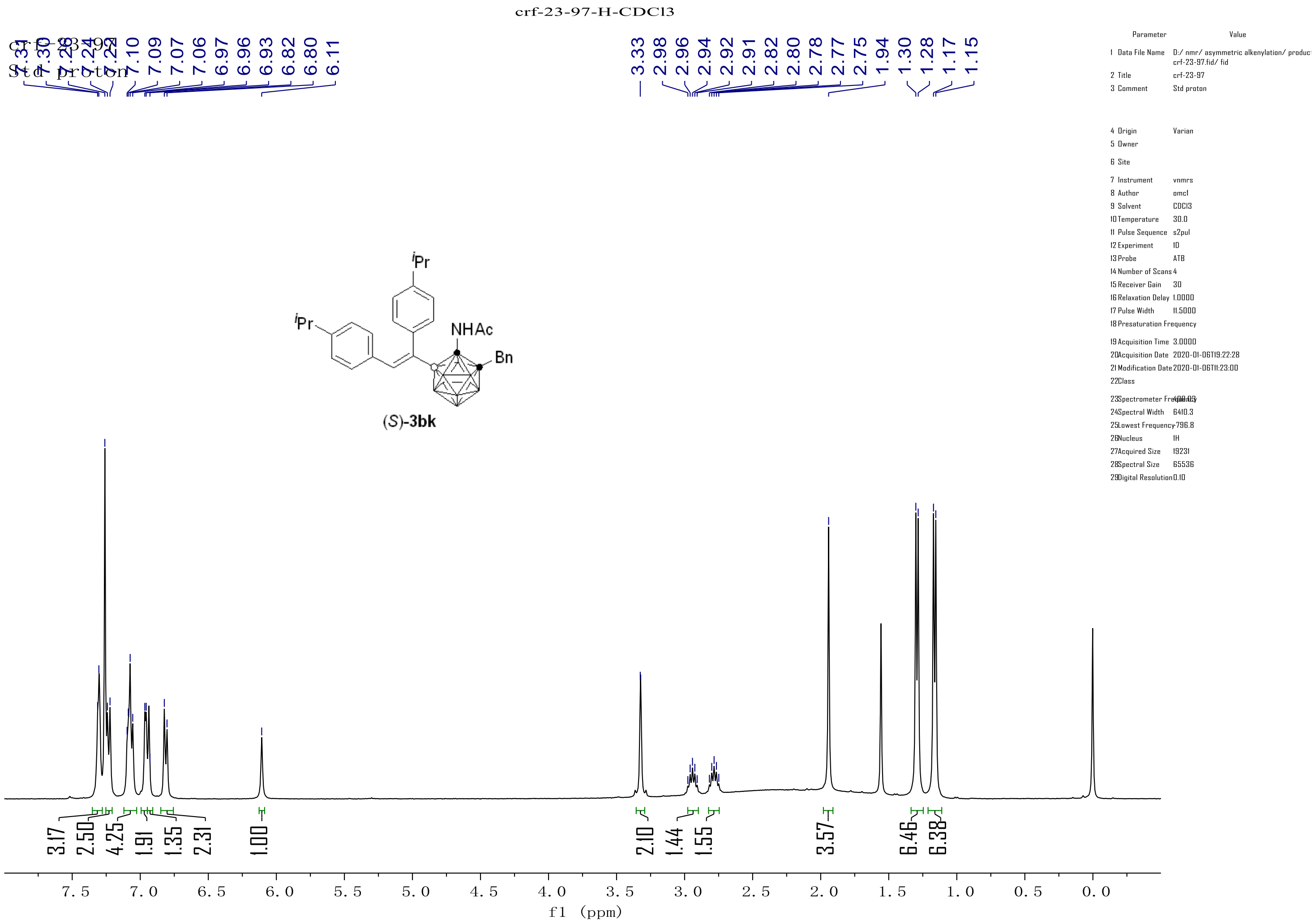

Supplementary Figure 111. $^{13}C\{^{1}H\}$ NMR of (*S*)-**3bk**.

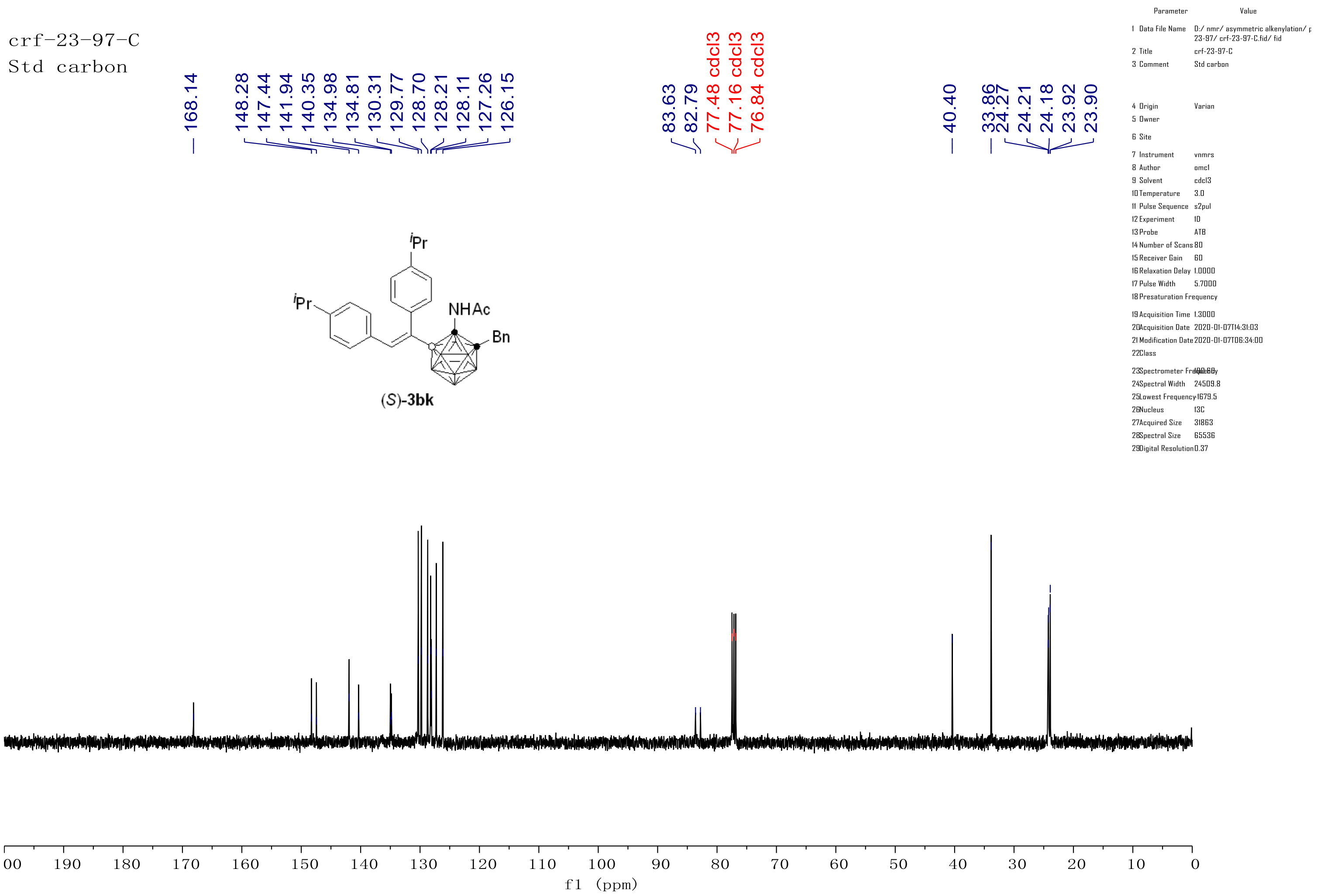

Supplementary Figure 112. $^{11}B\{^{1}H\}$ NMR of (*S*)-**3bk**.

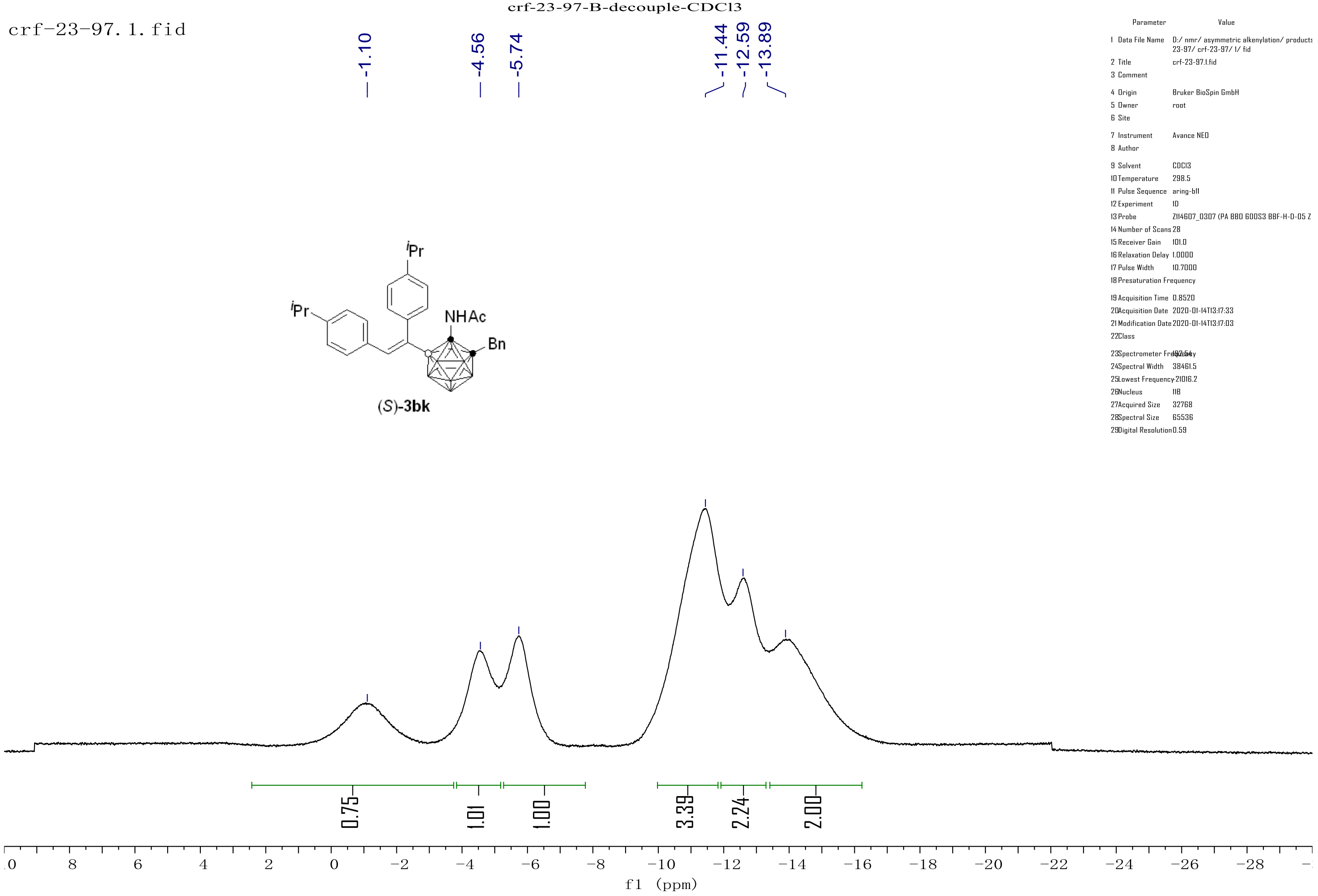

Parameter	Value
1 Data File Name	D:/ nmr/ asymmetric alkenylation/ products 23-97/ crf-23-97/ 1/ fid
2 Title	crf-23-97.1.fid
3 Comment	
4 Origin	Bruker BioSpin GmbH
5 Owner	root
6 Site	
7 Instrument	Avance NEO
8 Author	
9 Solvent	CDCl3
10 Temperature	298.5
11 Pulse Sequence	aring-b11
12 Experiment	1D
13 Probe	Z114607_0307 (PA BBO 600S3 BBF-H-D-05 Z
14 Number of Scans	28
15 Receiver Gain	101.0
16 Relaxation Delay	1.0000
17 Pulse Width	10.7000
18 Presaturation Frequency	
19 Acquisition Time	0.8520
20 Acquisition Date	2020-01-14T13:17:33
21 Modification Date	2020-01-14T13:17:03
22 Class	
23 Spectrometer Frequency	192.54
24 Spectral Width	38461.5
25 Lowest Frequency	-21016.2
26 Nucleus	11B
27 Acquired Size	32768
28 Spectral Size	65536
29 Digital Resolution	0.59

Supplementary Figure 113. ^{11}B NMR of (*S*)-**3bk**.

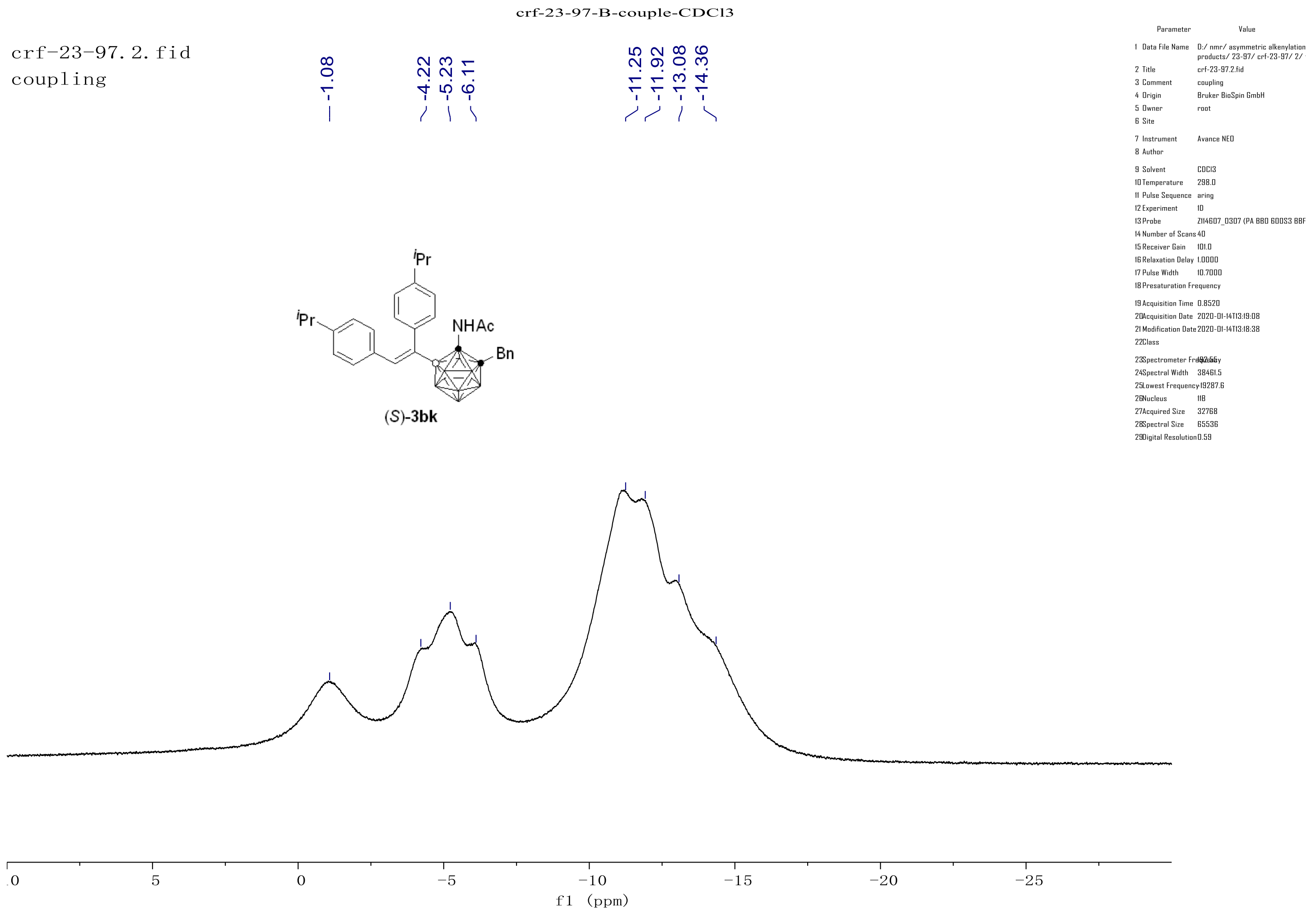

Supplementary Figure 114. ^{1}H NMR of (*S*)-**3bl**.

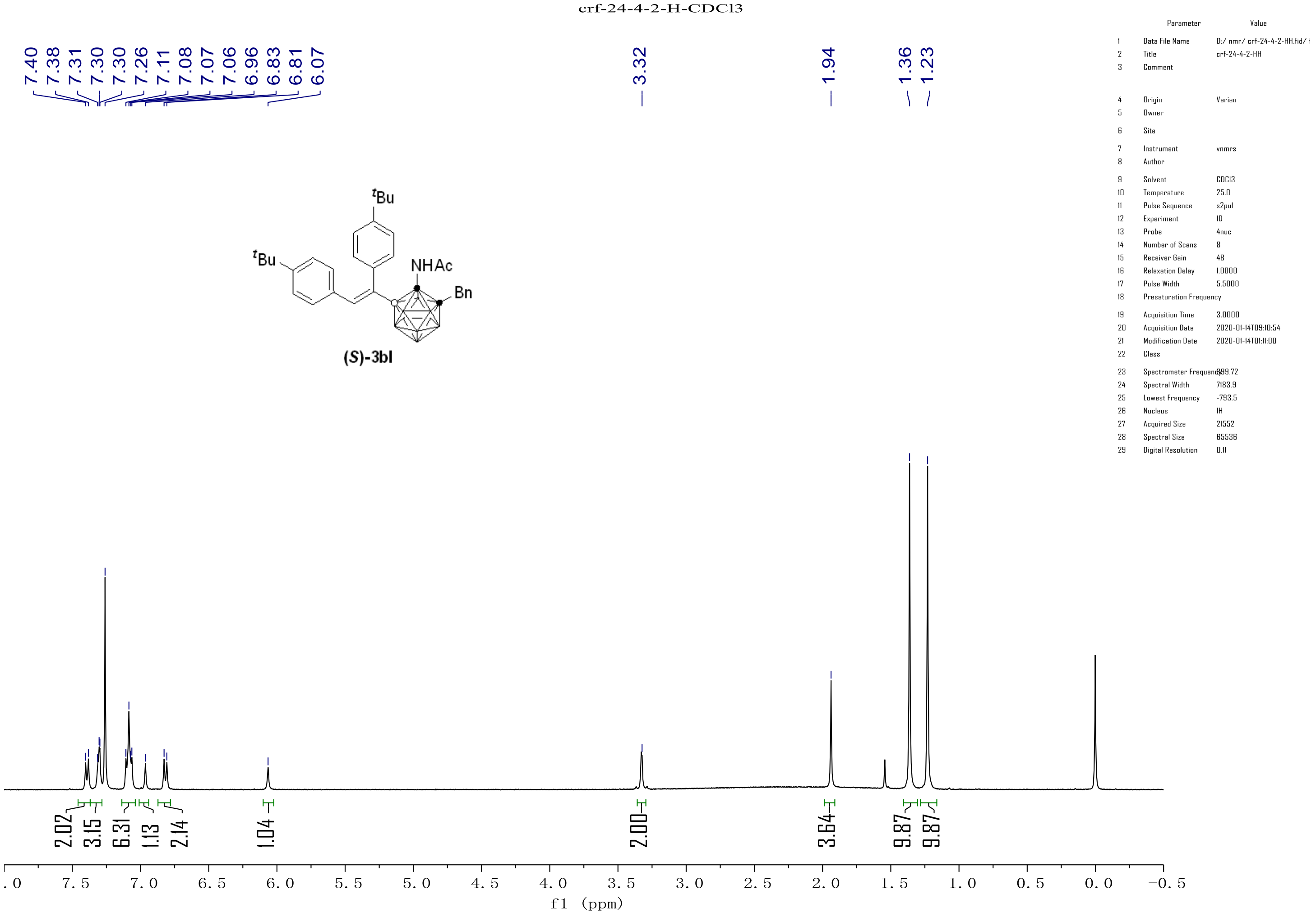

Supplementary Figure 115. $^{13}C\{^1H\}$ NMR of (*S*)-**3bl**.

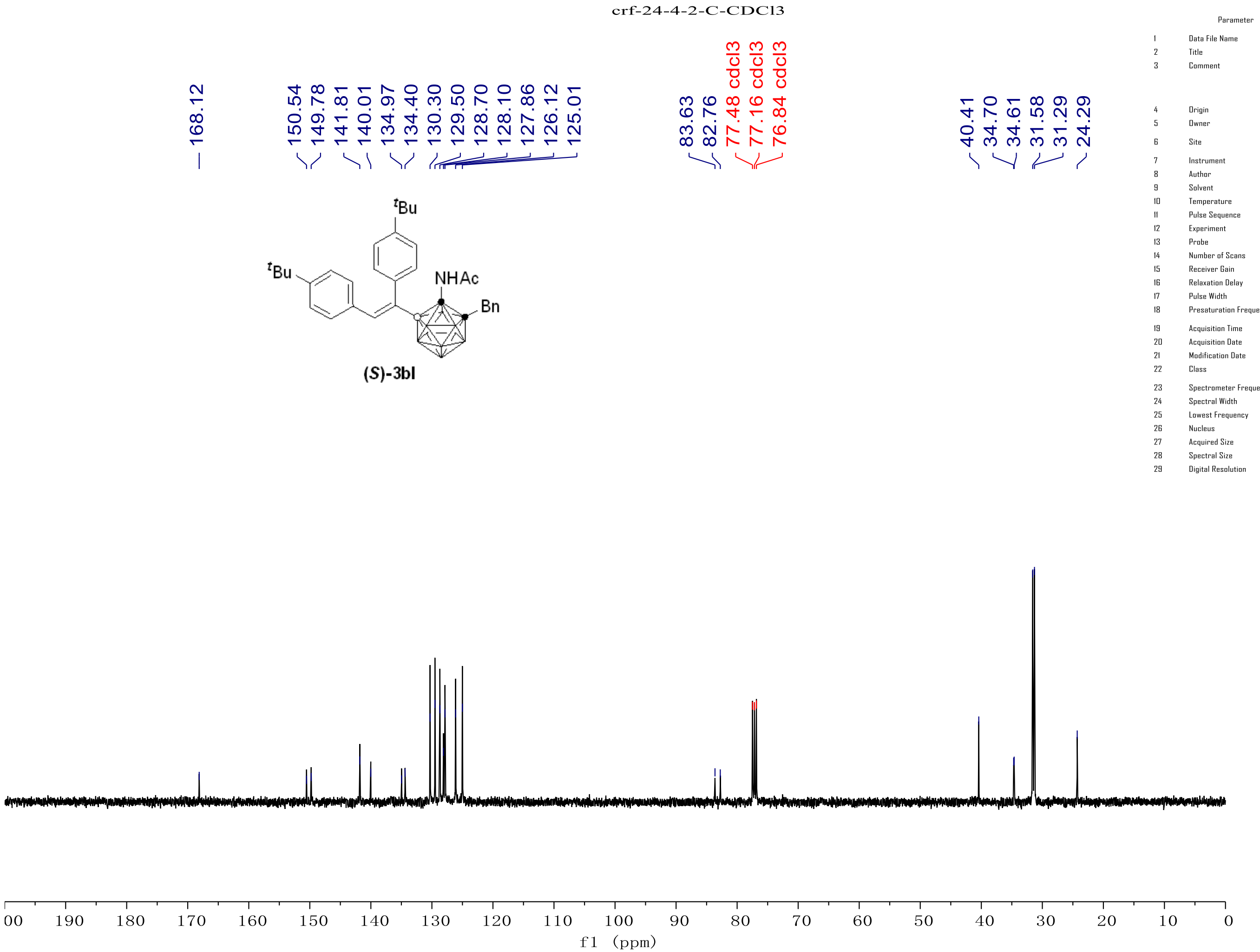

	Parameter	Value
1	Data File Name	D:/ nmr/ crf-24-4-2-C.fid/ fid
2	Title	crf-24-4-2-C
3	Comment	Std carbon
4	Origin	Varian
5	Owner	
6	Site	
7	Instrument	vnmrs
8	Author	omcl
9	Solvent	cdcl3
10	Temperature	3.0
11	Pulse Sequence	s2pul
12	Experiment	1D
13	Probe	ATB
14	Number of Scans	108
15	Receiver Gain	28
16	Relaxation Delay	1.0000
17	Pulse Width	5.7000
18	Presaturation Frequency	
19	Acquisition Time	1.3000
20	Acquisition Date	2020-01-14T10:56:09
21	Modification Date	2020-01-14T03:00:00
22	Class	
23	Spectrometer Frequency	100.60
24	Spectral Width	24509.8
25	Lowest Frequency	-1680.3
26	Nucleus	13C
27	Acquired Size	31863
28	Spectral Size	65536
29	Digital Resolution	0.37

Supplementary Figure 116. $^{11}B\{^{1}H\}$ NMR of (*S*)-**3bl**.

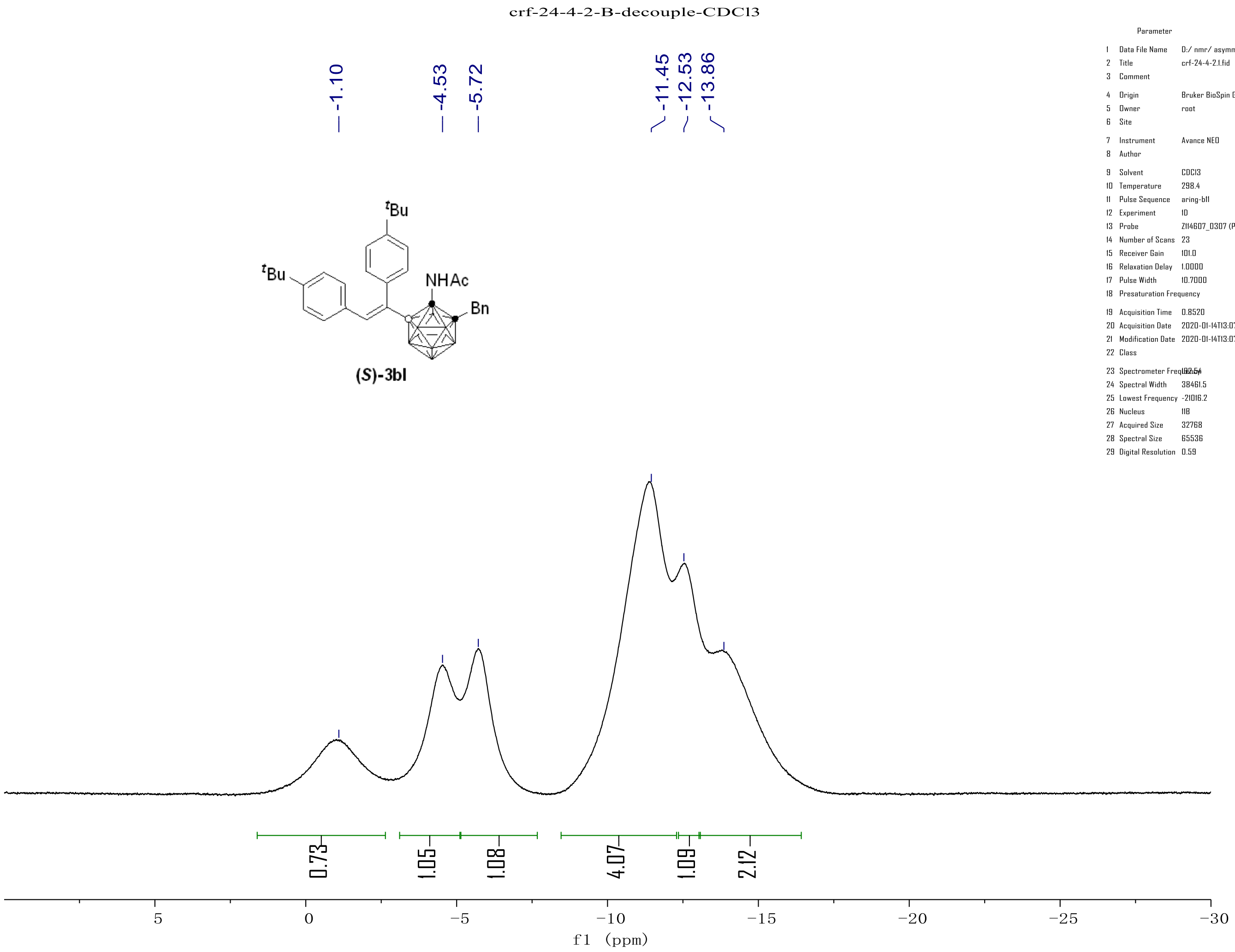

(*S*)-**3bl**

	Parameter	Value
1	Data File Name	D:/ nmr/ asymmetric alkenylation/ products/ 24-4
2	Title	crf-24-4-2.1.fid
3	Comment	
4	Origin	Bruker BioSpin GmbH
5	Owner	root
6	Site	
7	Instrument	Avance NEO
8	Author	
9	Solvent	CDCl3
10	Temperature	298.4
11	Pulse Sequence	aring-b11
12	Experiment	1D
13	Probe	Z114607_0307 (PA BBO 600S3 BBF-H-D-05 Z SP)
14	Number of Scans	23
15	Receiver Gain	101.0
16	Relaxation Delay	1.0000
17	Pulse Width	10.7000
18	Presaturation Frequency	
19	Acquisition Time	0.8520
20	Acquisition Date	2020-01-14T13:07:31
21	Modification Date	2020-01-14T13:07:00
22	Class	
23	Spectrometer Frequency	192.54
24	Spectral Width	38461.5
25	Lowest Frequency	-21016.2
26	Nucleus	11B
27	Acquired Size	32768
28	Spectral Size	65536
29	Digital Resolution	0.59

Supplementary Figure 117. ^{11}B NMR of (*S*)-**3bl**.

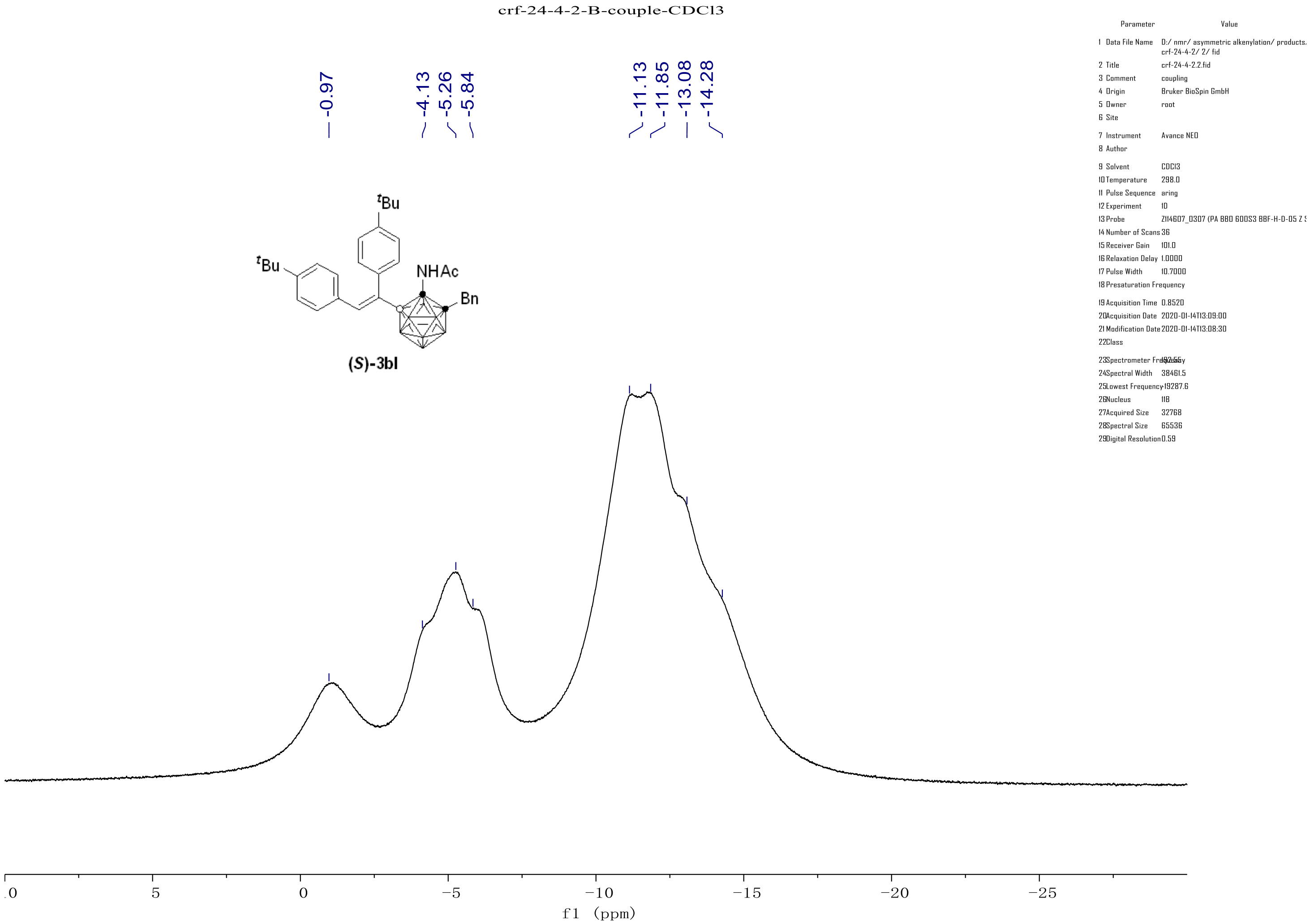

Supplementary Figure 118. ^{1}H NMR of (*S*)-**3bm**

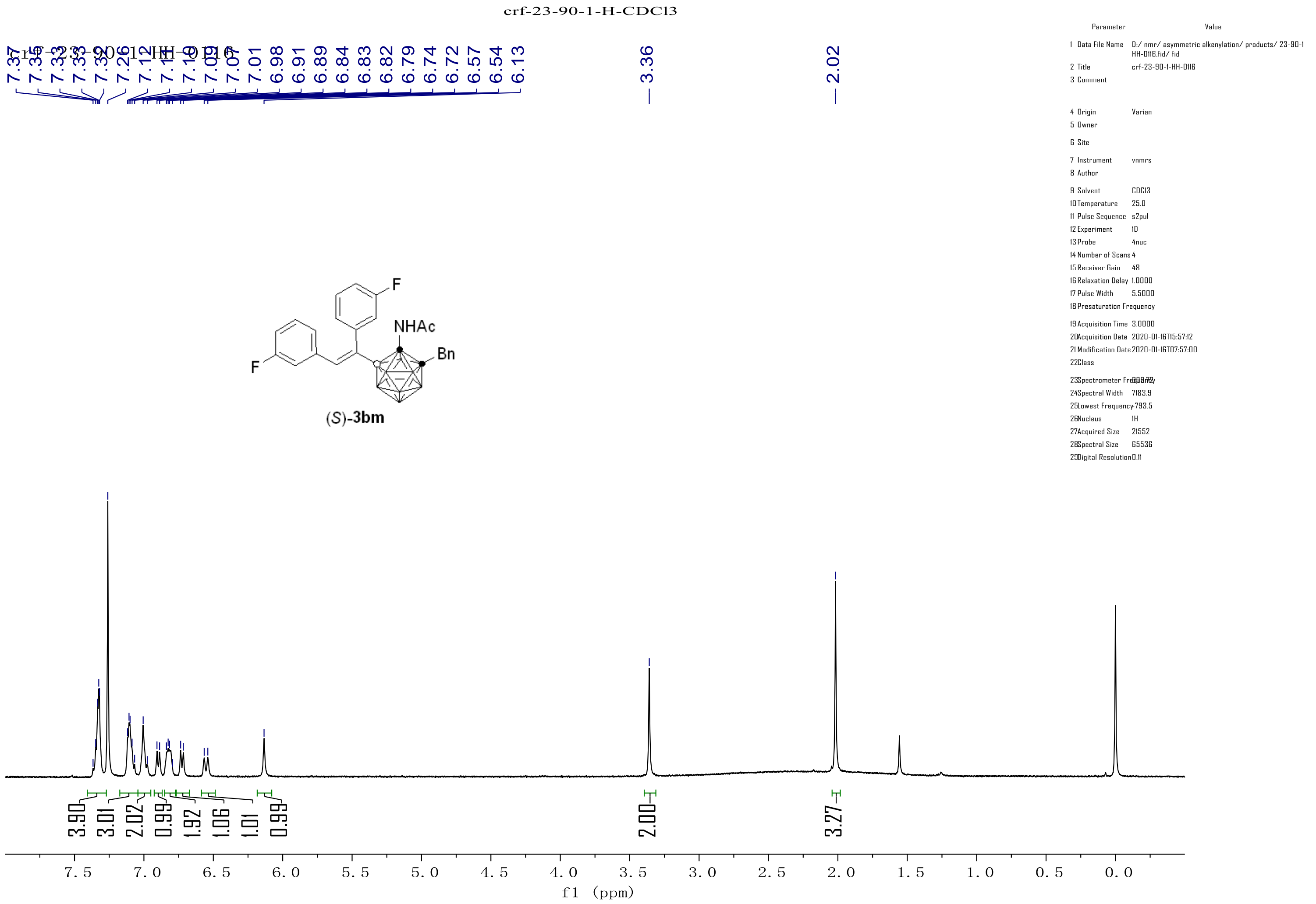

Supplementary Figure 119. $^{13}C\{^{1}H\}$ NMR of (*S*)-**3bm**.

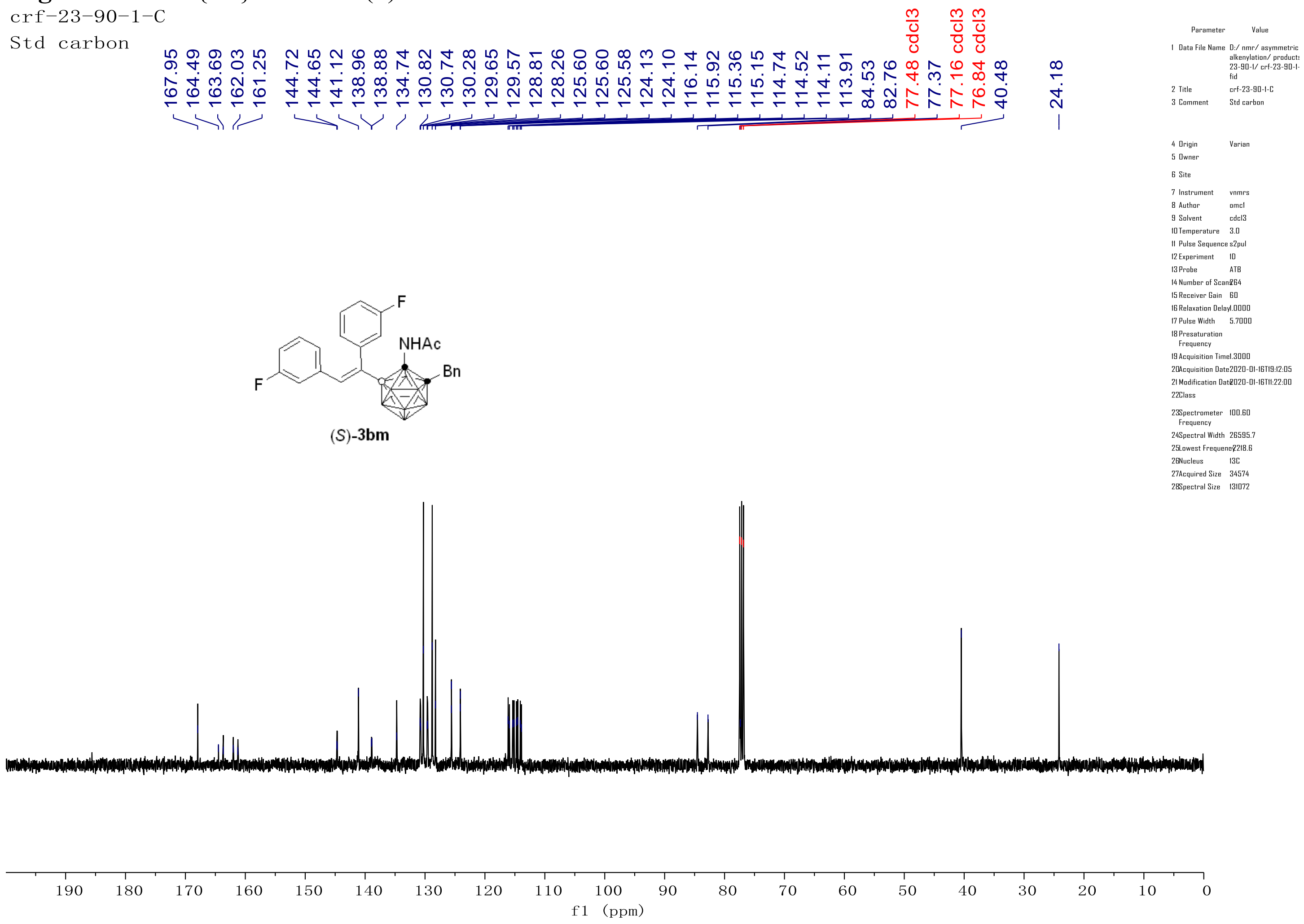

Supplementary Figure 120. $^{11}B\{^{1}H\}$ NMR of (*S*)-**3bm**.

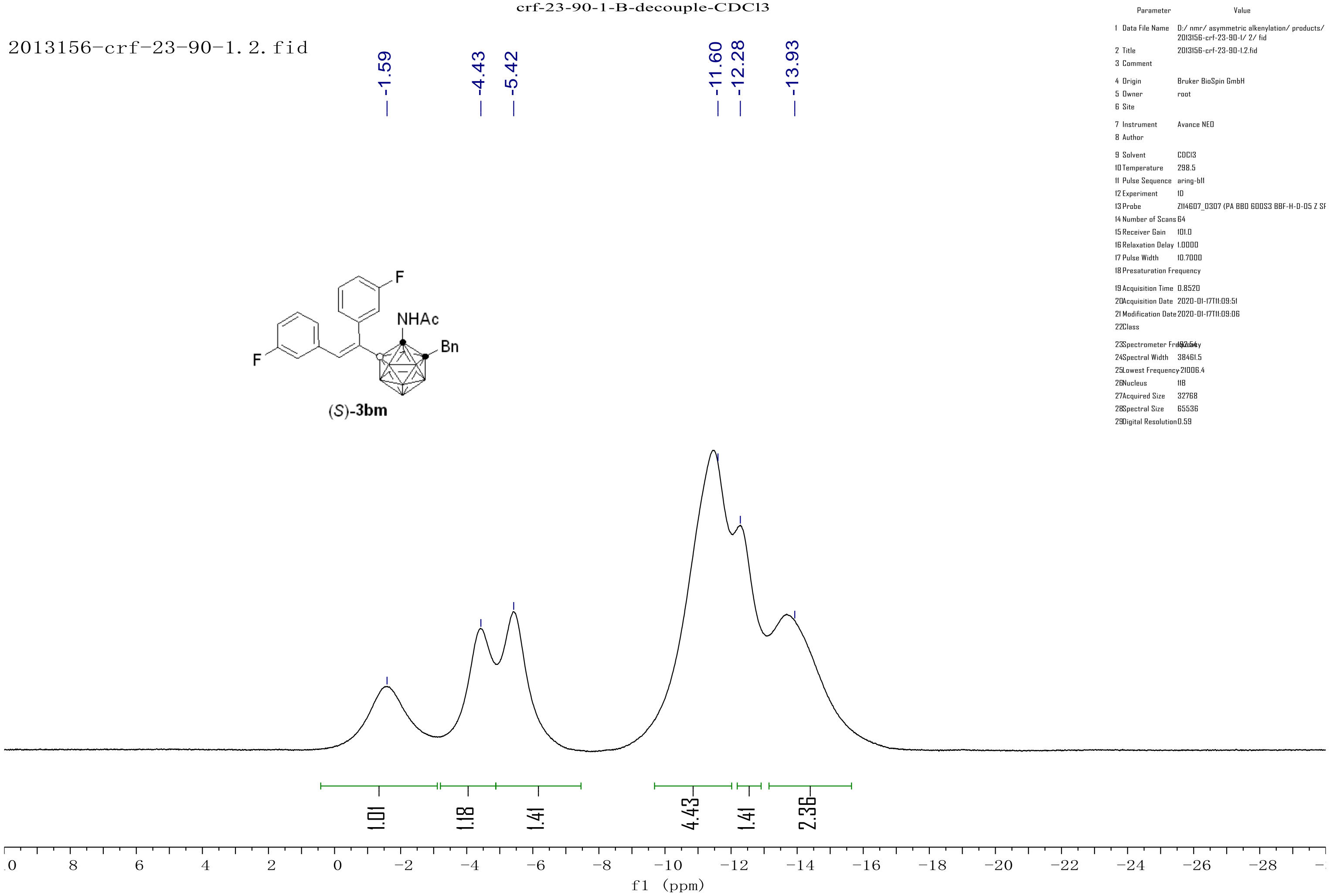

Parameter	Value
1 Data File Name	D:/ nmr/ asymmetric alkenylation/ products/ 2013156-crf-23-90-1/ 2/ fid
2 Title	2013156-crf-23-90-1.2.fid
3 Comment	
4 Origin	Bruker BioSpin GmbH
5 Owner	root
6 Site	
7 Instrument	Avance NEO
8 Author	
9 Solvent	CDCl3
10 Temperature	298.5
11 Pulse Sequence	aring-b11
12 Experiment	1D
13 Probe	Z114607_0307 (PA BBO 600S3 BBF-H-D-05 Z SF
14 Number of Scans	64
15 Receiver Gain	101.0
16 Relaxation Delay	1.0000
17 Pulse Width	10.7000
18 Presaturation Frequency	
19 Acquisition Time	0.8520
20 Acquisition Date	2020-01-17T11:09:51
21 Modification Date	2020-01-17T11:09:06
22 Class	
23 Spectrometer Frequency	192.54
24 Spectral Width	38461.5
25 Lowest Frequency	-21006.4
26 Nucleus	11B
27 Acquired Size	32768
28 Spectral Size	65536
29 Digital Resolution	0.59

Supplementary Figure 121. ^{11}B NMR of (*S*)-**3bm**.

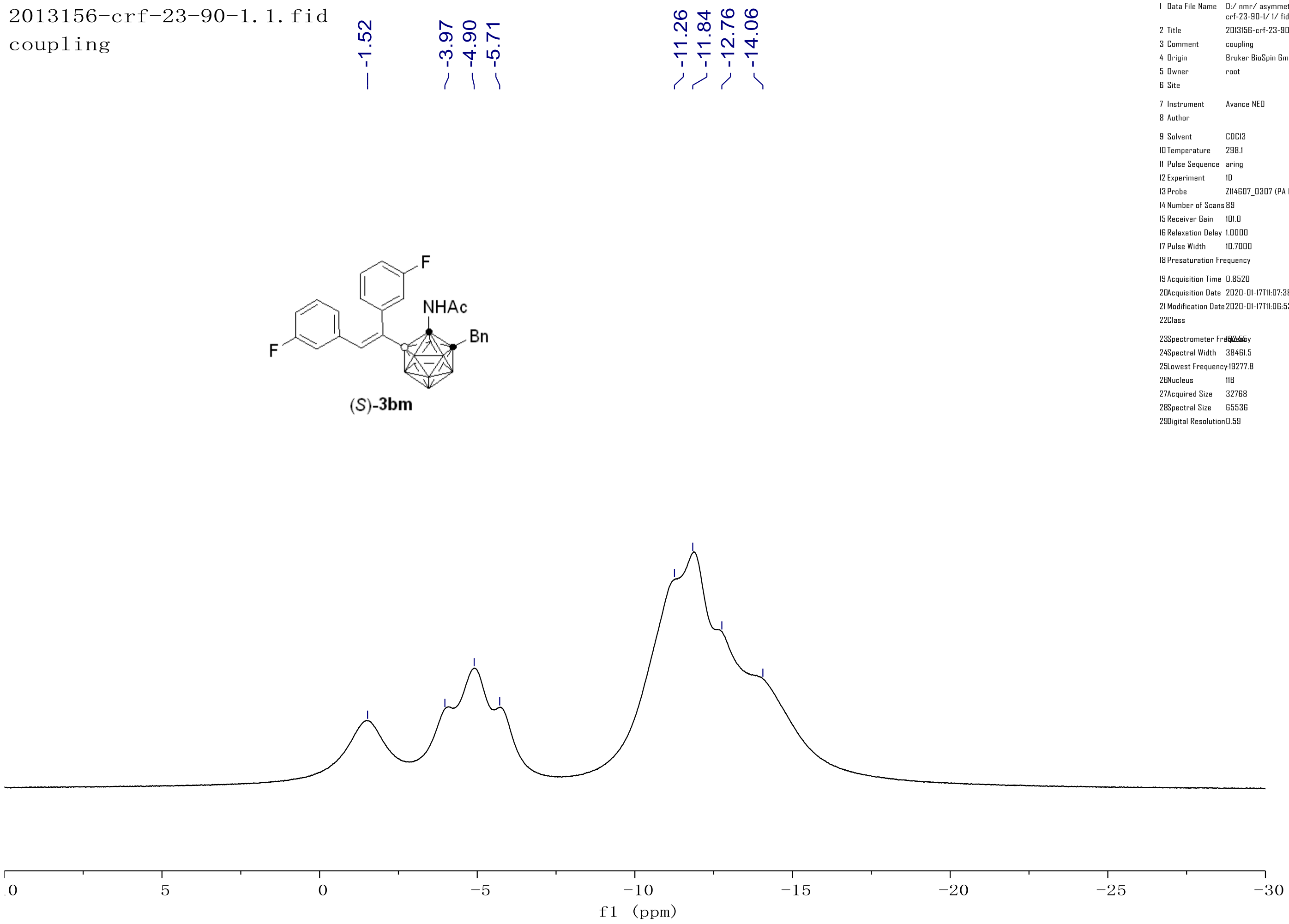

(*S*)-**3bm**

	Parameter	Value
1	Data File Name	D:/ nmr/ asymmetric alkenylation/ products/ 2 crf-23-90-1/ 1/ fid
2	Title	2013156-crf-23-90-1.1.fid
3	Comment	coupling
4	Origin	Bruker BioSpin GmbH
5	Owner	root
6	Site	
7	Instrument	Avance NEO
8	Author	
9	Solvent	CDCl3
10	Temperature	298.1
11	Pulse Sequence	aring
12	Experiment	1D
13	Probe	Z114607_0307 (PA BBO 600S3 BBF-H-D-05 Z SP)
14	Number of Scans	89
15	Receiver Gain	101.0
16	Relaxation Delay	1.0000
17	Pulse Width	10.7000
18	Presaturation Frequency	
19	Acquisition Time	0.8520
20	Acquisition Date	2020-01-17T11:07:38
21	Modification Date	2020-01-17T11:06:53
22	Class	
23	Spectrometer Frequency	192.55
24	Spectral Width	38461.5
25	Lowest Frequency	-19277.8
26	Nucleus	11B
27	Acquired Size	32768
28	Spectral Size	65536
29	Digital Resolution	0.59

Supplementary Figure 122. ^{19}F NMR of (*S*)-**3bm**.

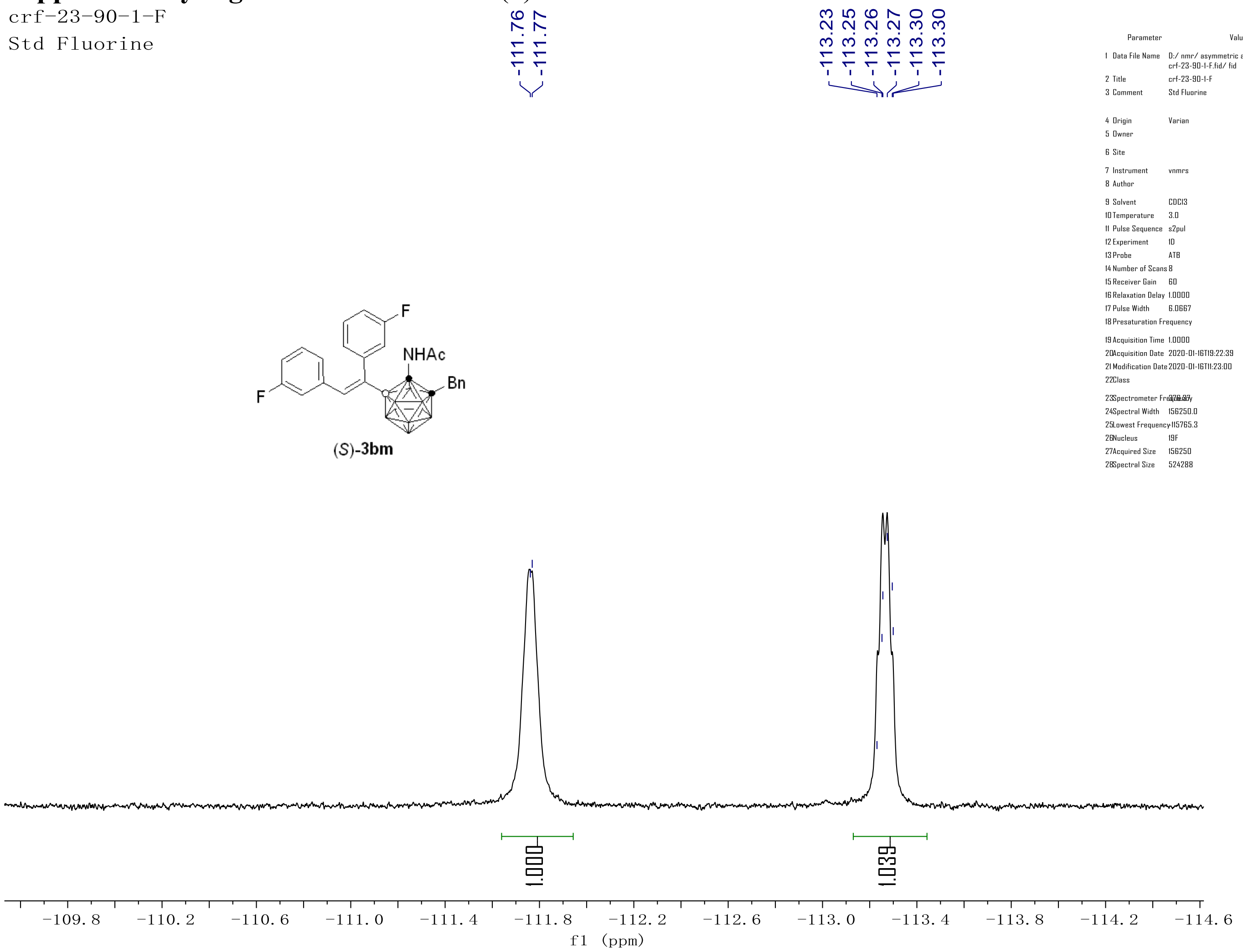

(*S*)-**3bm**

	Parameter	Value
1	Data File Name	D:/ nmr/ asymmetric alkenylation/ products/ crf-23-90-1-F.fid/ fid
2	Title	crf-23-90-1-F
3	Comment	Std Fluorine
4	Origin	Varian
5	Owner	
6	Site	
7	Instrument	vnmrs
8	Author	
9	Solvent	CDCl3
10	Temperature	3.0
11	Pulse Sequence	s2pul
12	Experiment	1D
13	Probe	ATB
14	Number of Scans	8
15	Receiver Gain	60
16	Relaxation Delay	1.0000
17	Pulse Width	6.0667
18	Presaturation Frequency	
19	Acquisition Time	1.0000
20	Acquisition Date	2020-01-16T19:22:39
21	Modification Date	2020-01-16T11:23:00
22	Class	
23	Spectrometer Frequency	376.37
24	Spectral Width	156250.0
25	Lowest Frequency	-115765.3
26	Nucleus	19F
27	Acquired Size	156250
28	Spectral Size	524288

Supplementary Figure 123. ^{1}H NMR of (*S*)-**3bn**.

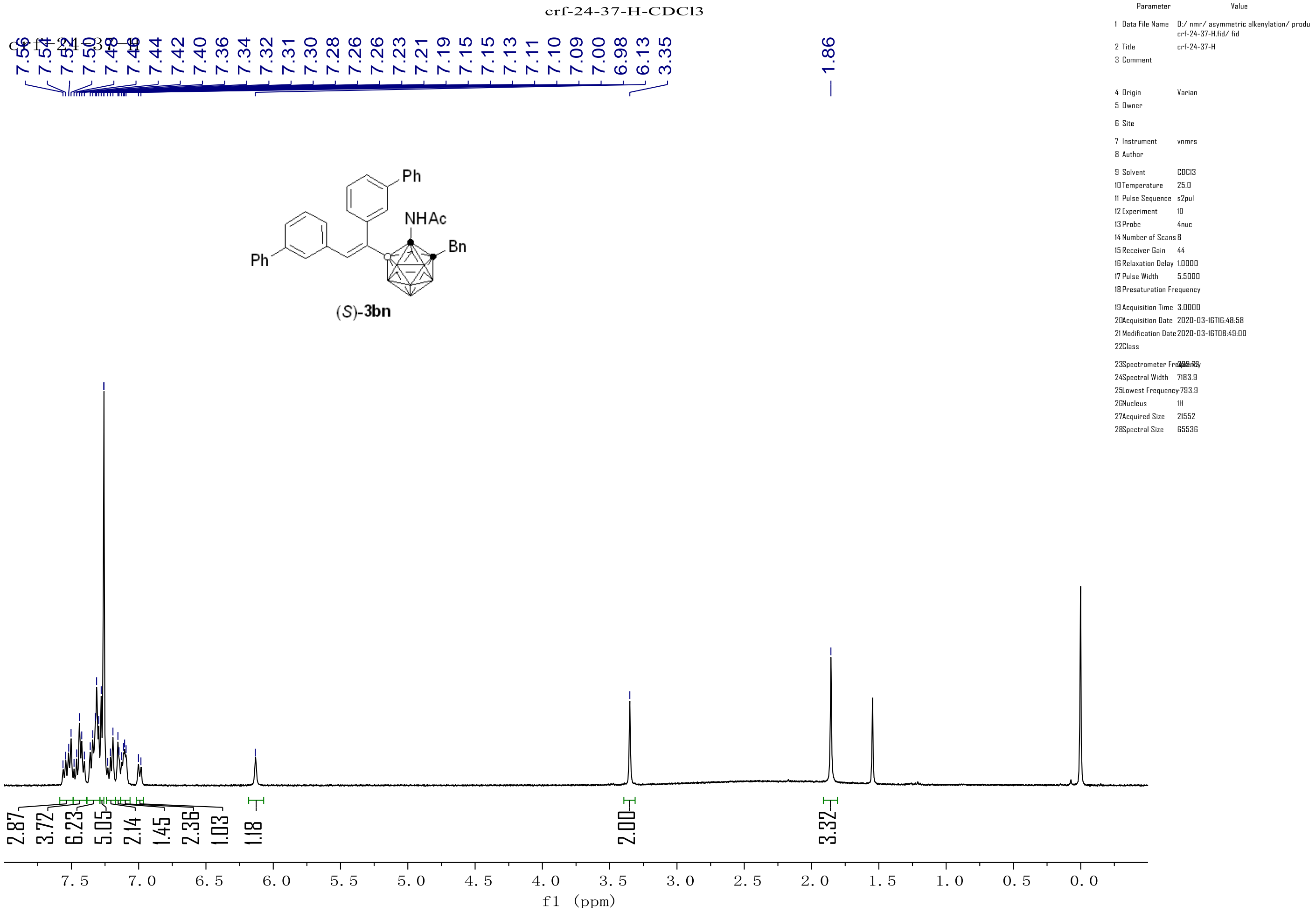

Supplementary Figure 124. $^{13}C\{^{1}H\}$ NMR of (*S*)-**3bn**.

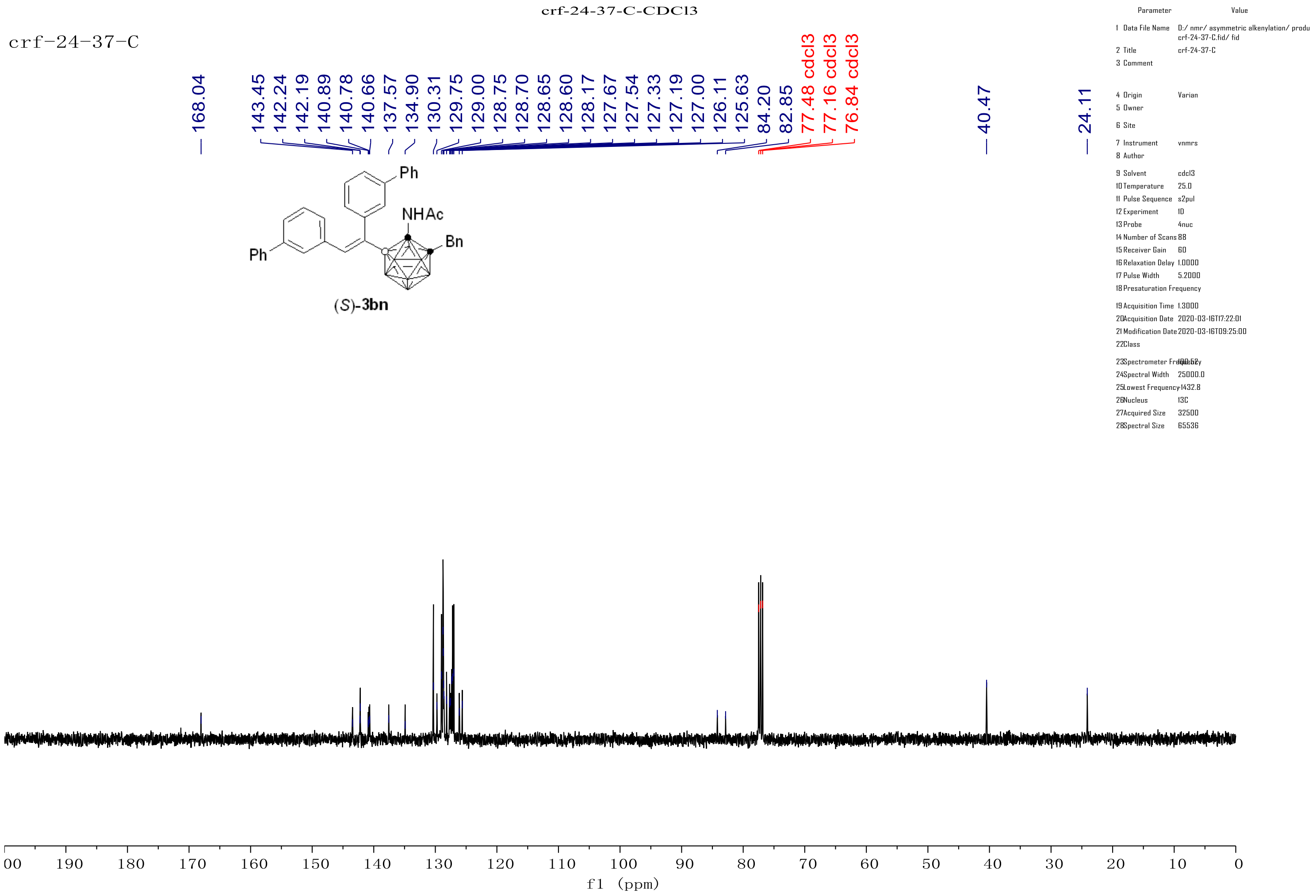

Supplementary Figure 125. $^{11}B\{^{1}H\}$ NMR of (*S*)-**3bn**.

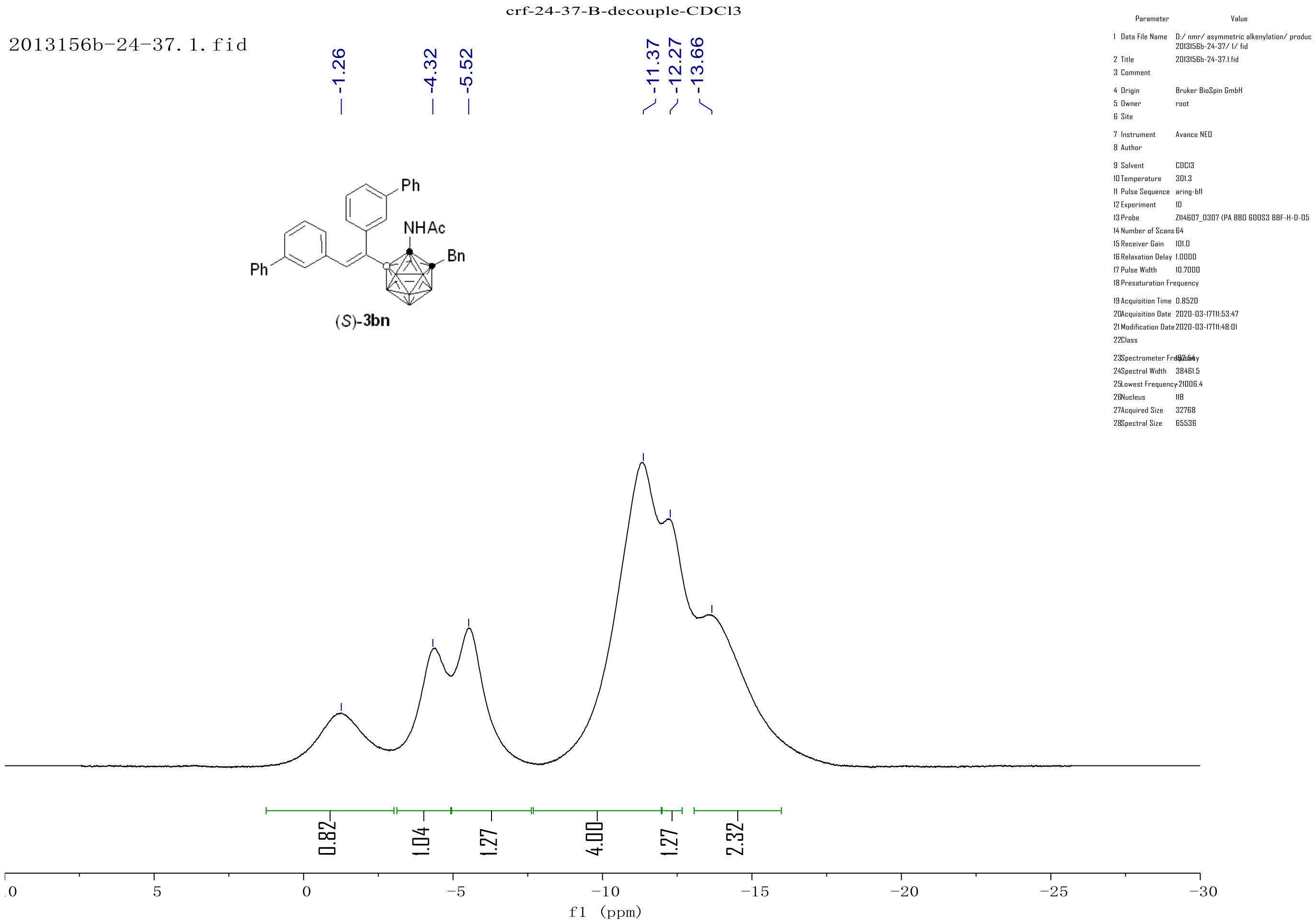

Supplementary Figure 126. ^{11}B NMR of (*S*)-**3bn**.

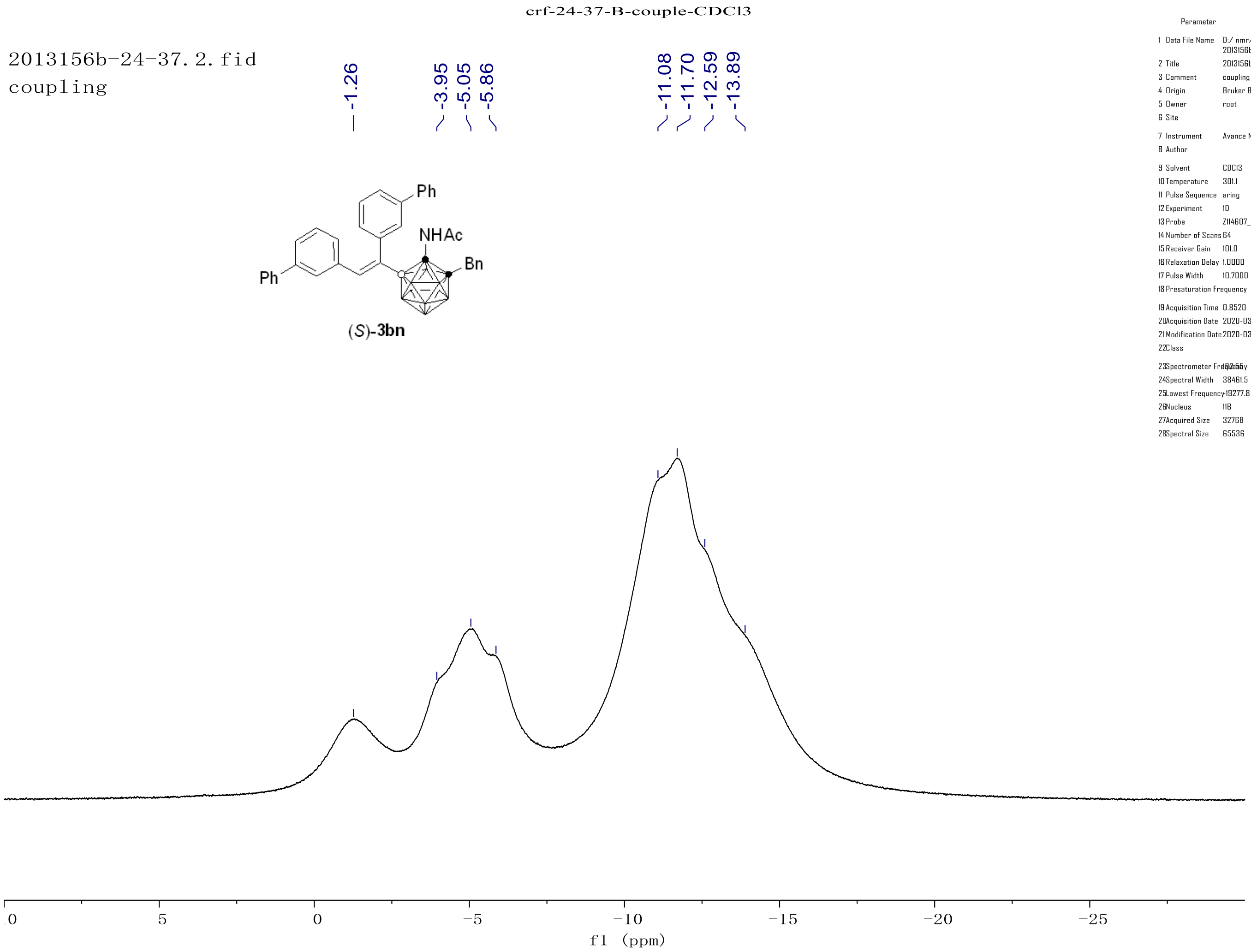

Parameter	Value
1 Data File Name	D:/ nmr/ asymmetric alkenylation/ product 2013156b-24-37/ 2/ fid
2 Title	2013156b-24-37.2.fid
3 Comment	coupling
4 Origin	Bruker BioSpin GmbH
5 Owner	root
6 Site	
7 Instrument	Avance NEO
8 Author	
9 Solvent	CDCl3
10 Temperature	301.1
11 Pulse Sequence	aring
12 Experiment	1D
13 Probe	Z114607_0307 (PA BBO 600S3 BBF-H-D-05 Z
14 Number of Scans	64
15 Receiver Gain	101.0
16 Relaxation Delay	1.0000
17 Pulse Width	10.7000
18 Presaturation Frequency	
19 Acquisition Time	0.8520
20 Acquisition Date	2020-03-17T11:56:33
21 Modification Date	2020-03-17T11:50:47
22 Class	
23 Spectrometer Frequency	192.55
24 Spectral Width	38461.5
25 Lowest Frequency	-19277.8
26 Nucleus	11B
27 Acquired Size	32768
28 Spectral Size	65536

Supplementary Figure 127. ^{1}H NMR of (*S*)-**3bo**.

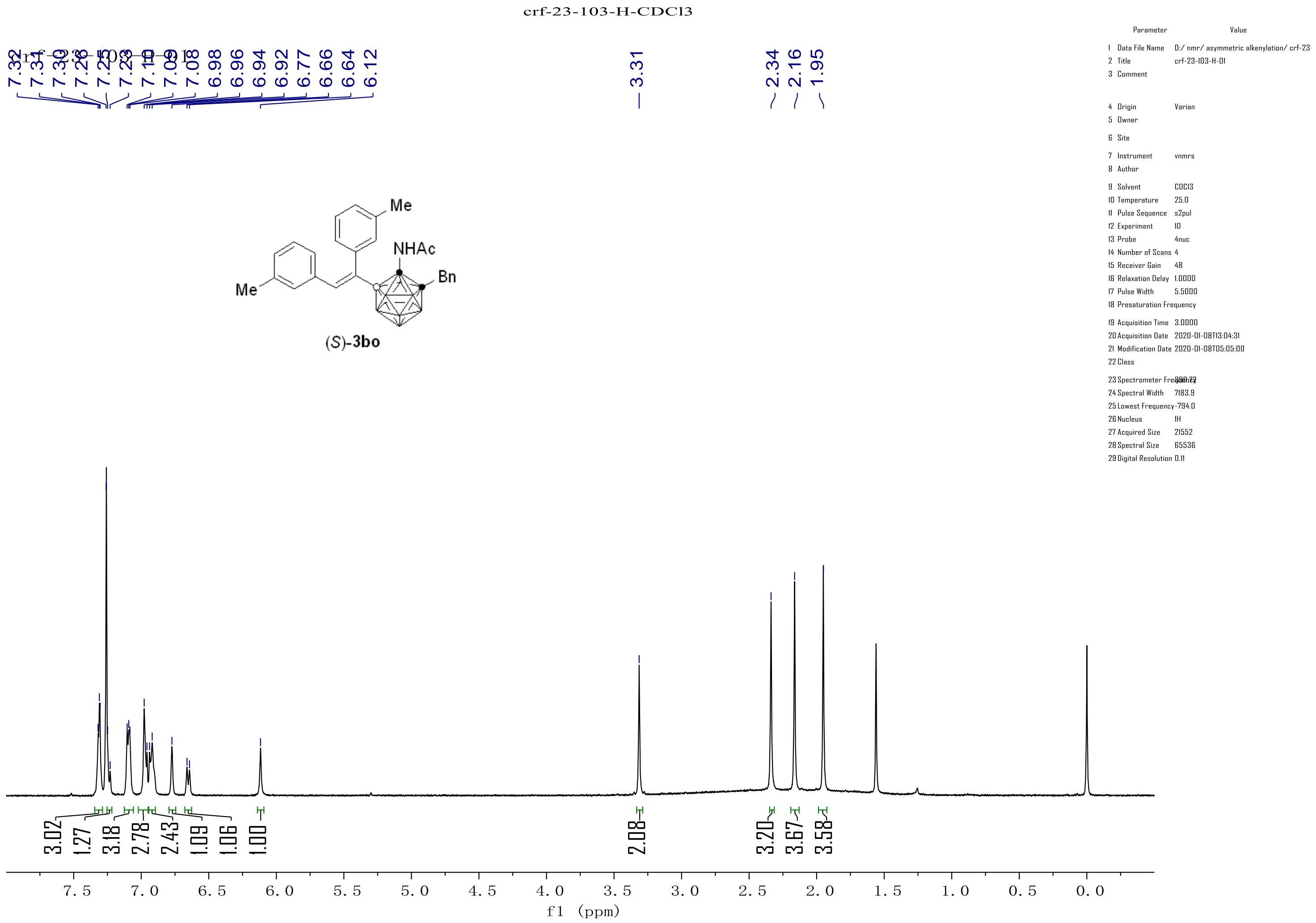

Supplementary Figure 128. $^{13}C\{^{1}H\}$ NMR of (*S*)-**3bo**.

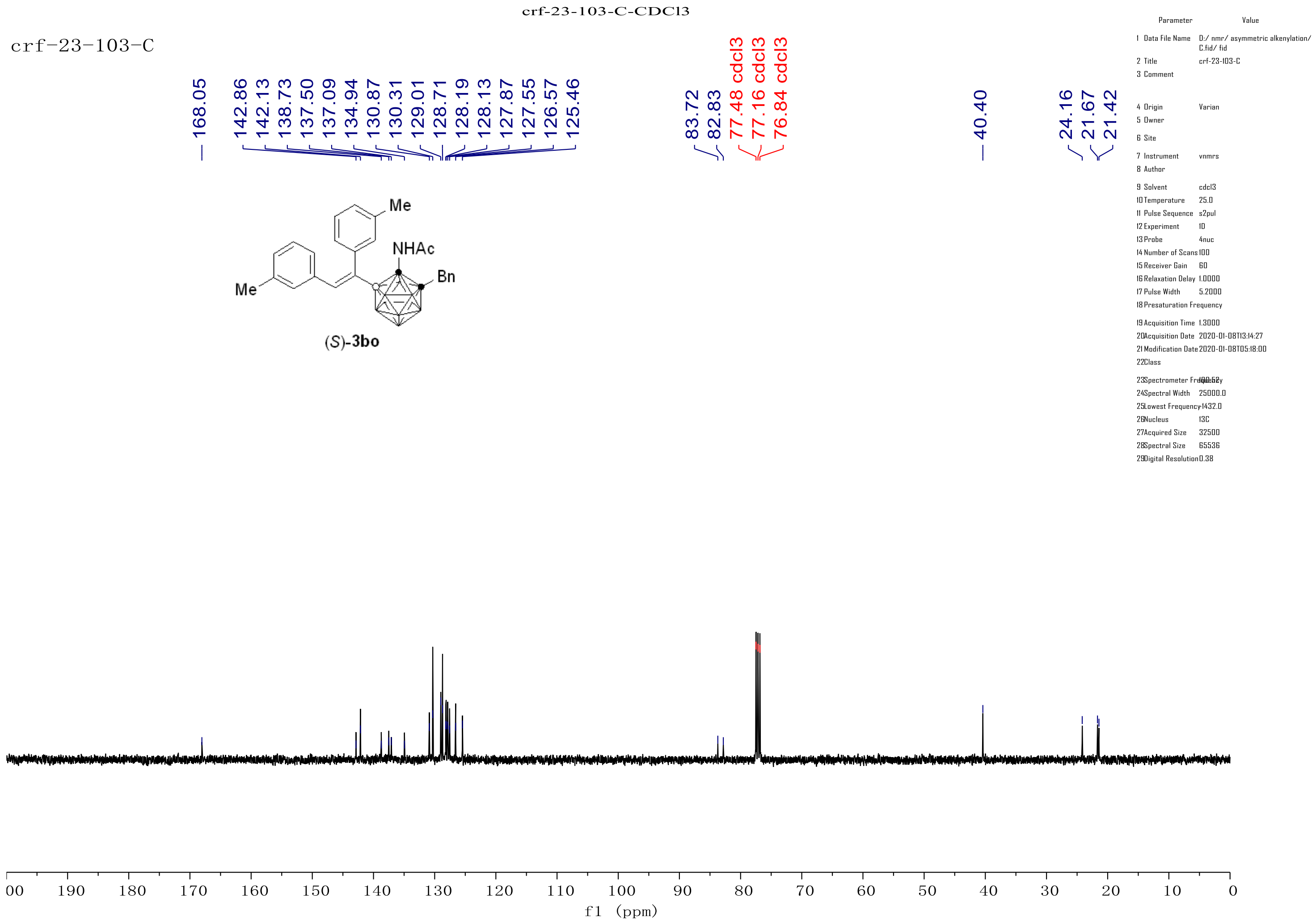

Supplementary Figure 129. $^{11}B\{^{1}H\}$ NMR of (*S*)-**3bo**.

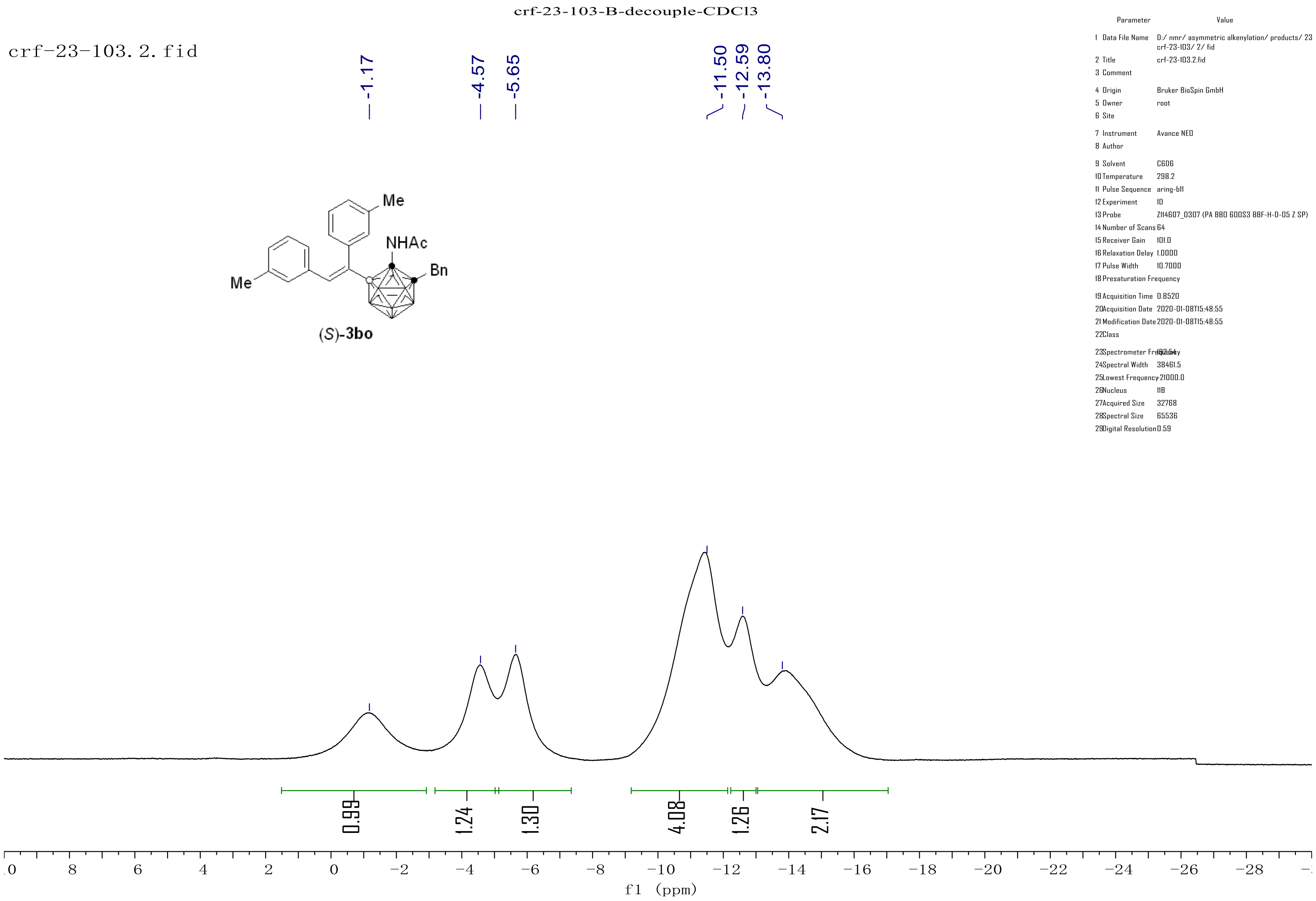

Parameter	Value
1 Data File Name	D:/ nmr/ asymmetric alkenylation/ products/ 23 crf-23-103/ 2/ fid
2 Title	crf-23-103.2.fid
3 Comment	
4 Origin	Bruker BioSpin GmbH
5 Owner	root
6 Site	
7 Instrument	Avance NEO
8 Author	
9 Solvent	C6D6
10 Temperature	298.2
11 Pulse Sequence	aring-b11
12 Experiment	1D
13 Probe	Z114607_0307 (PA BBO 600S3 BBF-H-D-05 Z SP)
14 Number of Scans	64
15 Receiver Gain	101.0
16 Relaxation Delay	1.0000
17 Pulse Width	10.7000
18 Presaturation Frequency	
19 Acquisition Time	0.8520
20 Acquisition Date	2020-01-08T15:48:55
21 Modification Date	2020-01-08T15:48:55
22 Class	
23 Spectrometer Frequency	192.54
24 Spectral Width	38461.5
25 Lowest Frequency	-21000.0
26 Nucleus	11B
27 Acquired Size	32768
28 Spectral Size	65536
29 Digital Resolution	0.59

Supplementary Figure 130. ^{11}B NMR of (*S*)-**3bo**.

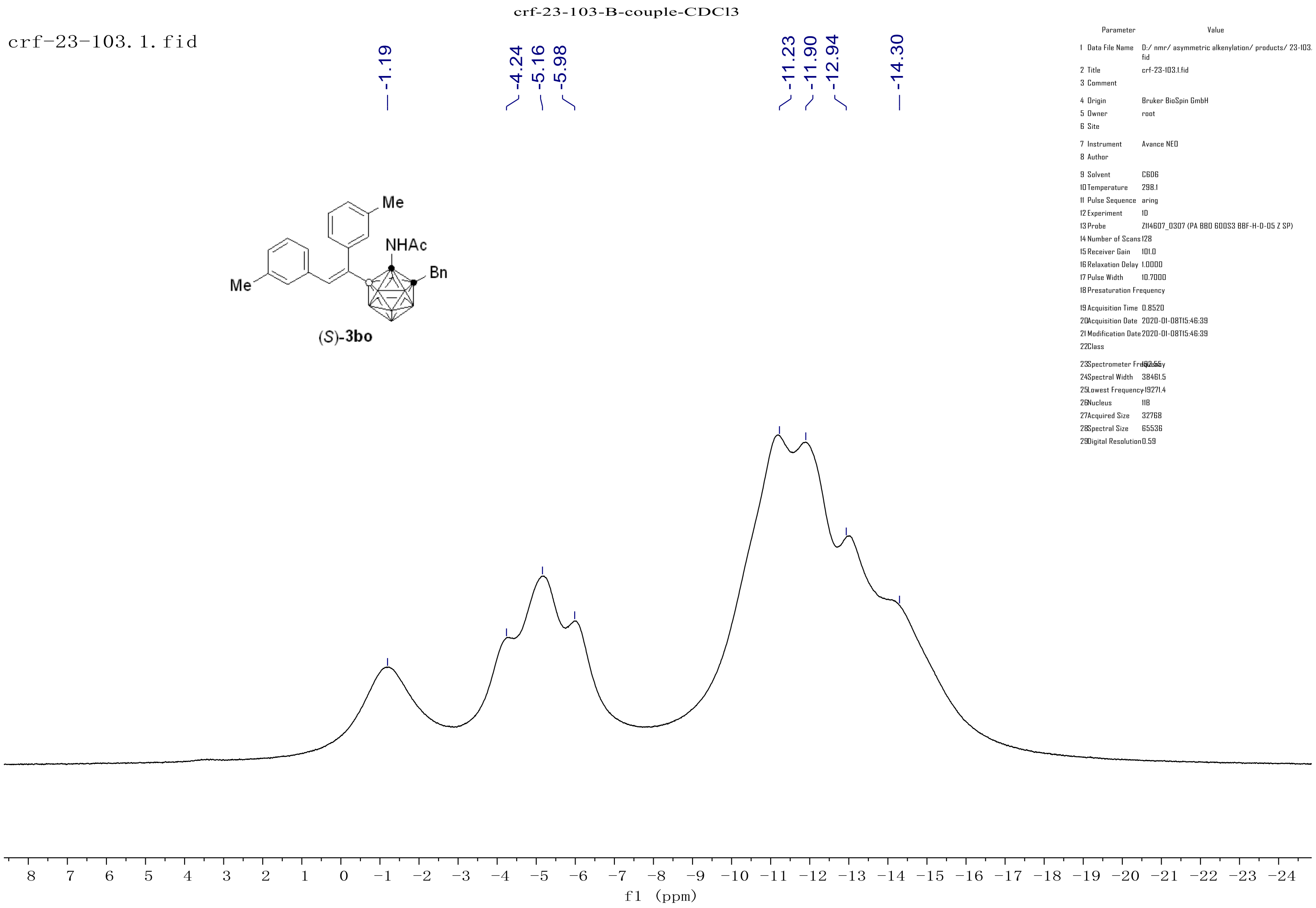

Supplementary Figure 131. ^{1}H NMR of (*S*)-**3bp**.

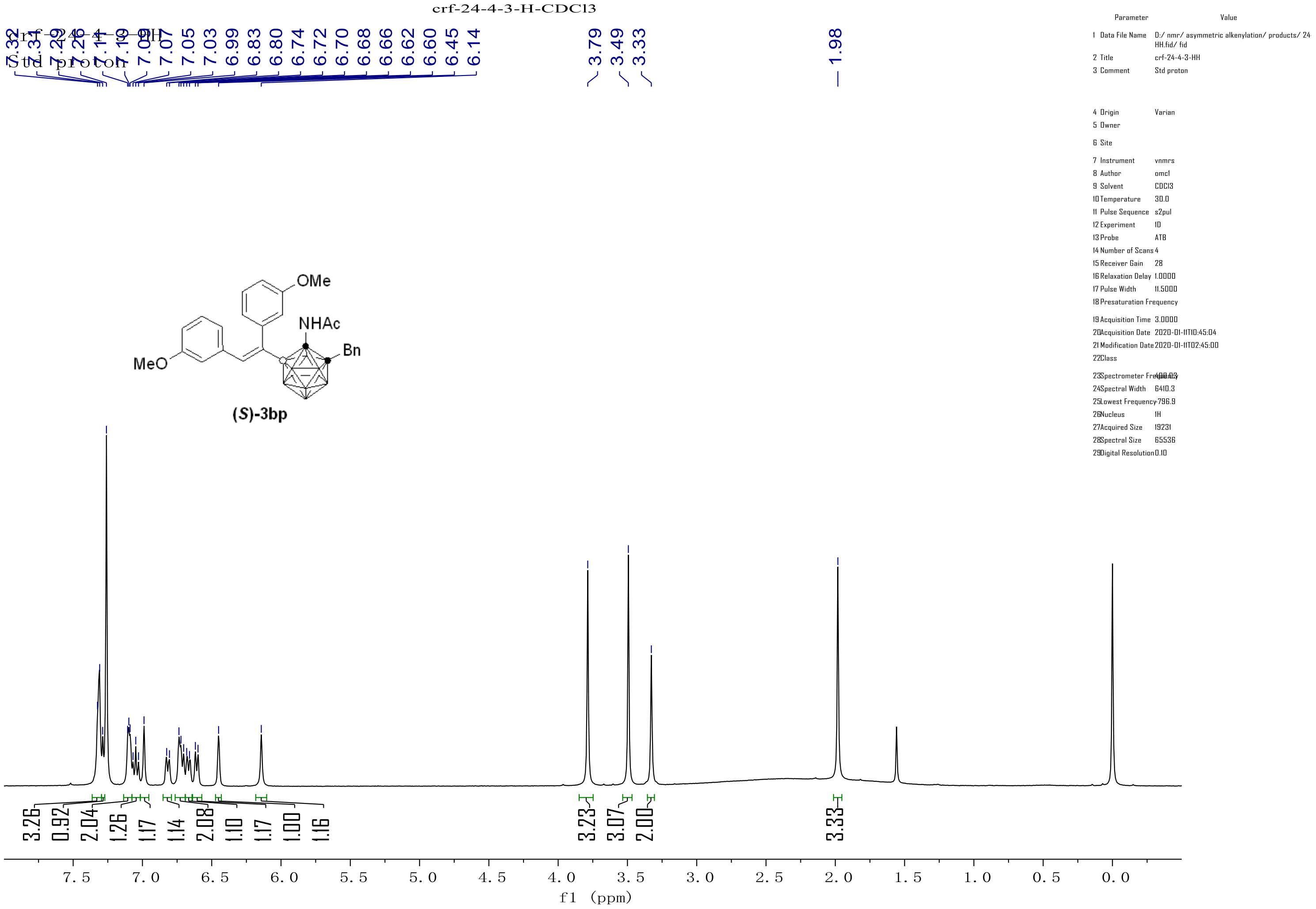

Supplementary Figure 132. $^{13}C\{^{1}H\}$ NMR of (*S*)-**3bp**.

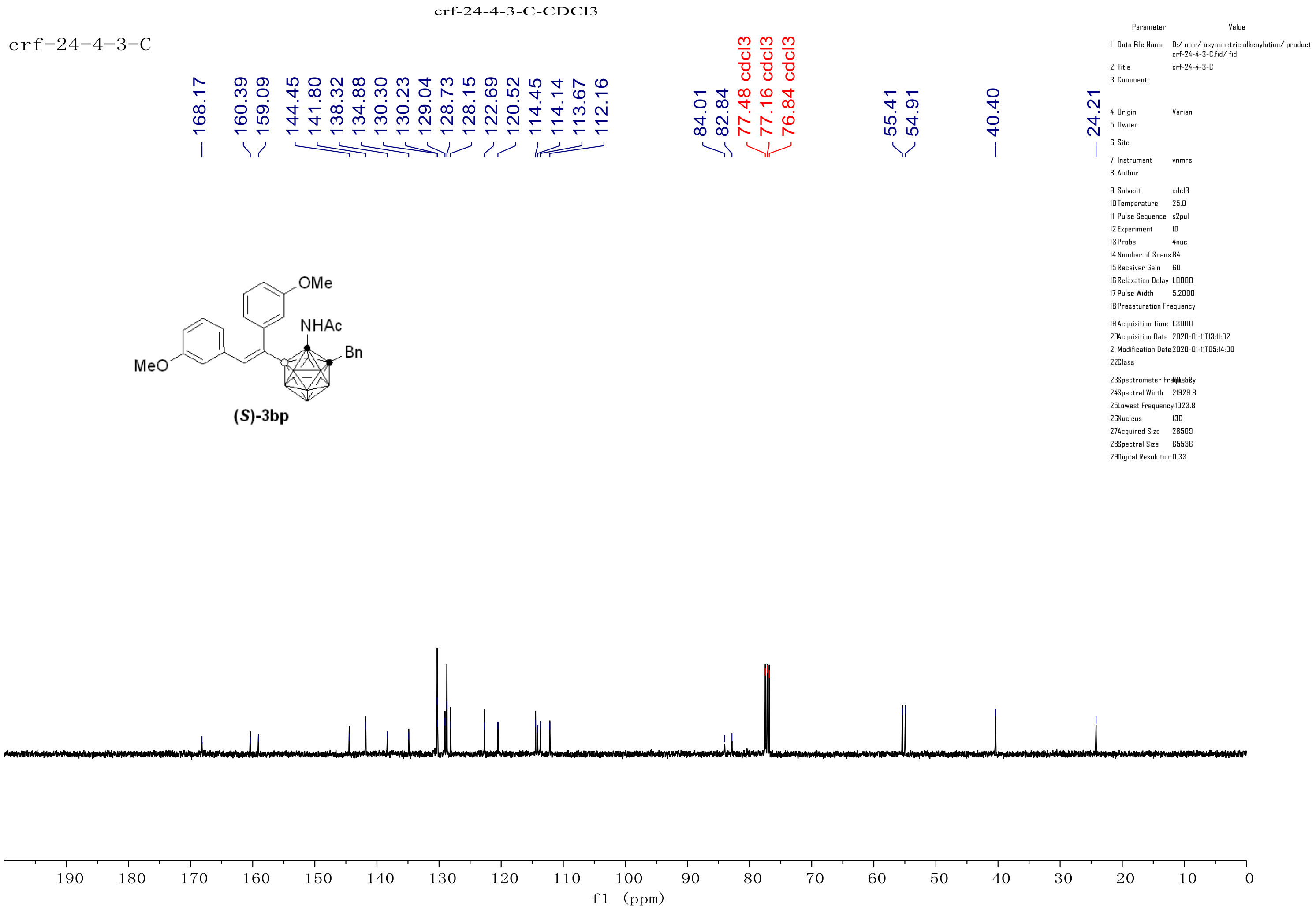

Supplementary Figure 133. $^{11}B\{^{1}H\}$ NMR of (*S*)-**3bp**.

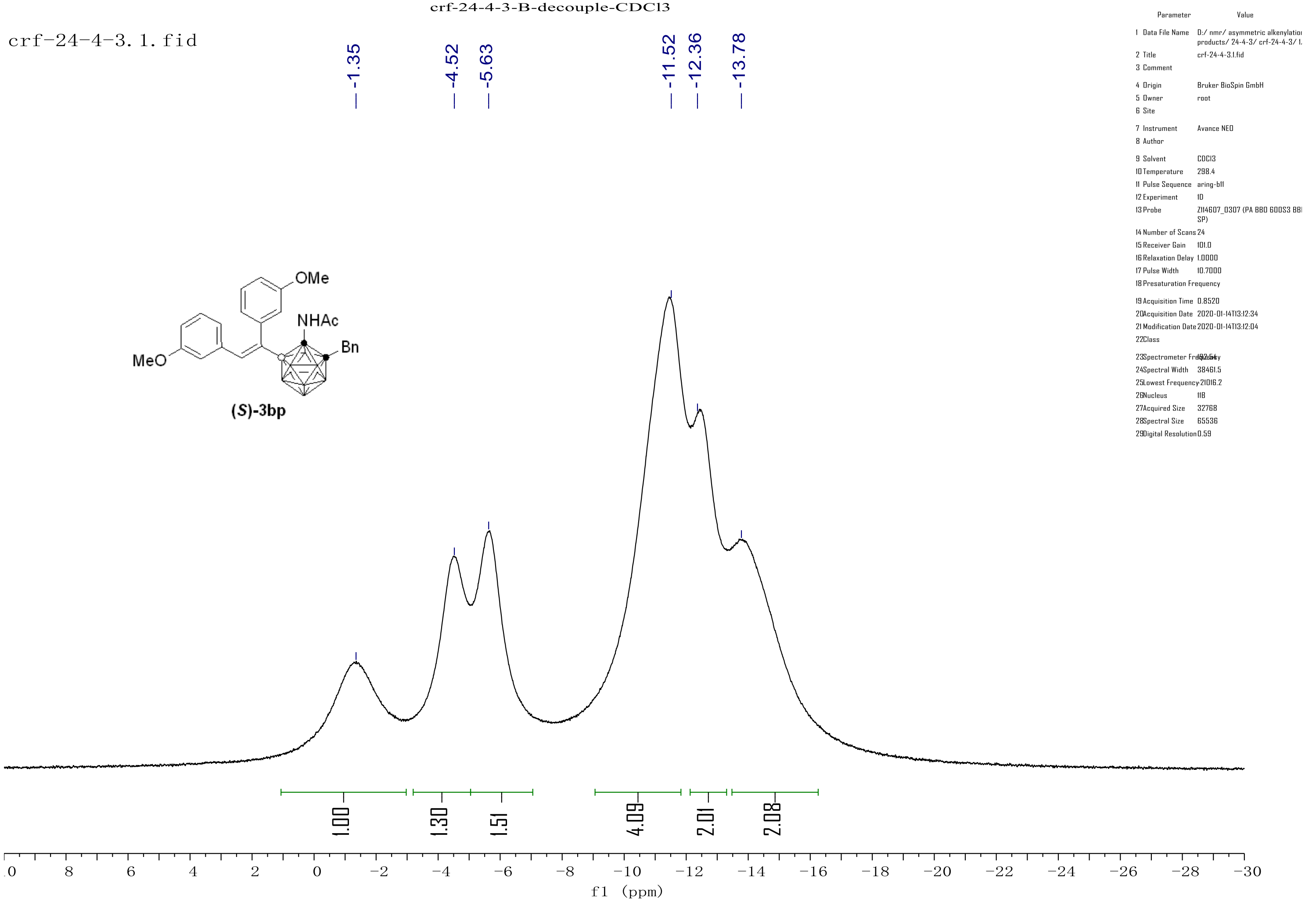

Supplementary Figure 134. ^{11}B NMR of (*S*)-**3bp**.

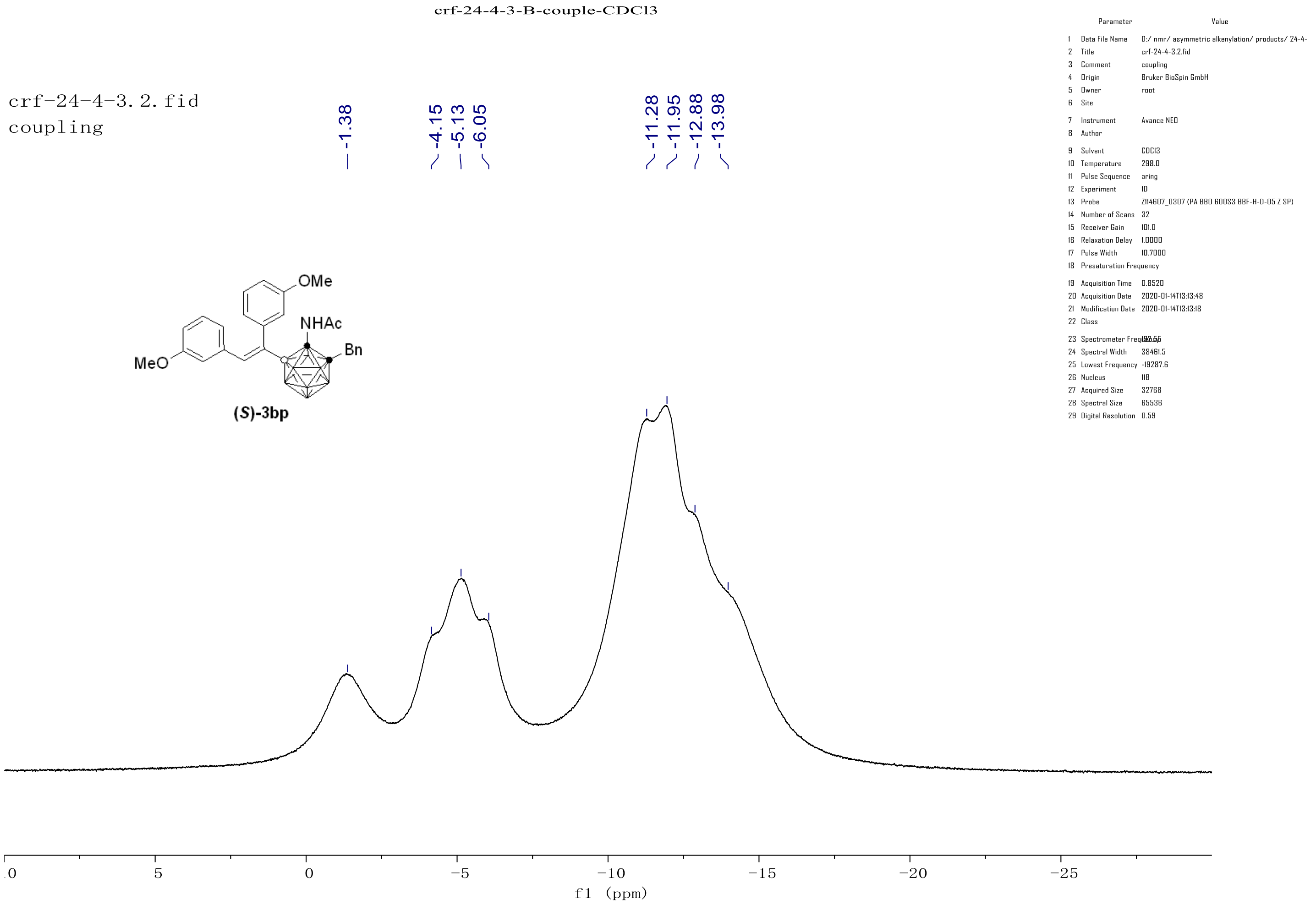

Supplementary Figure 135. ^{1}H NMR of (*S*)-**3bq**.

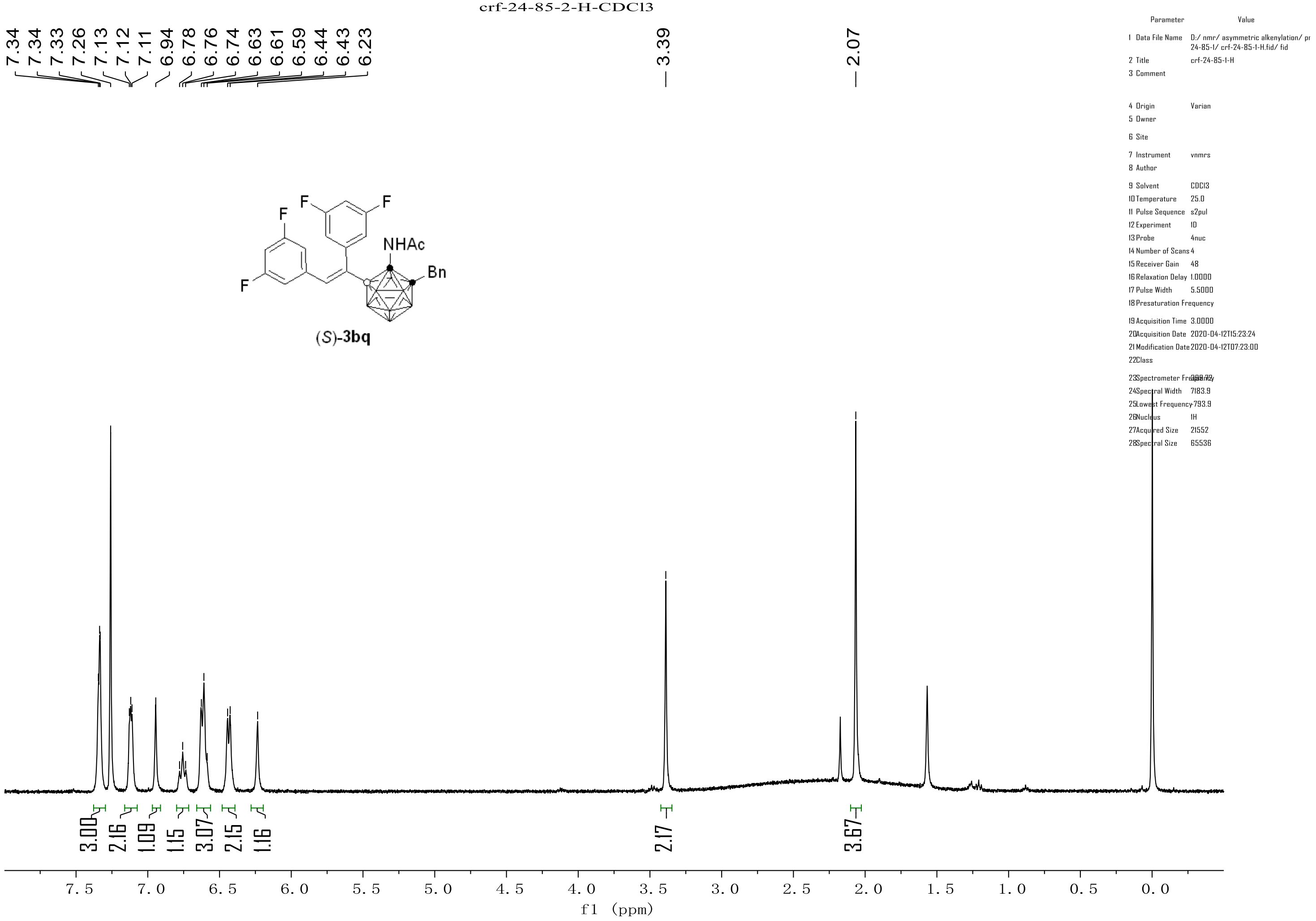

Supplementary Figure 136. $^{13}C\{^{1}H\}$ NMR of (*S*)-**3bq**.

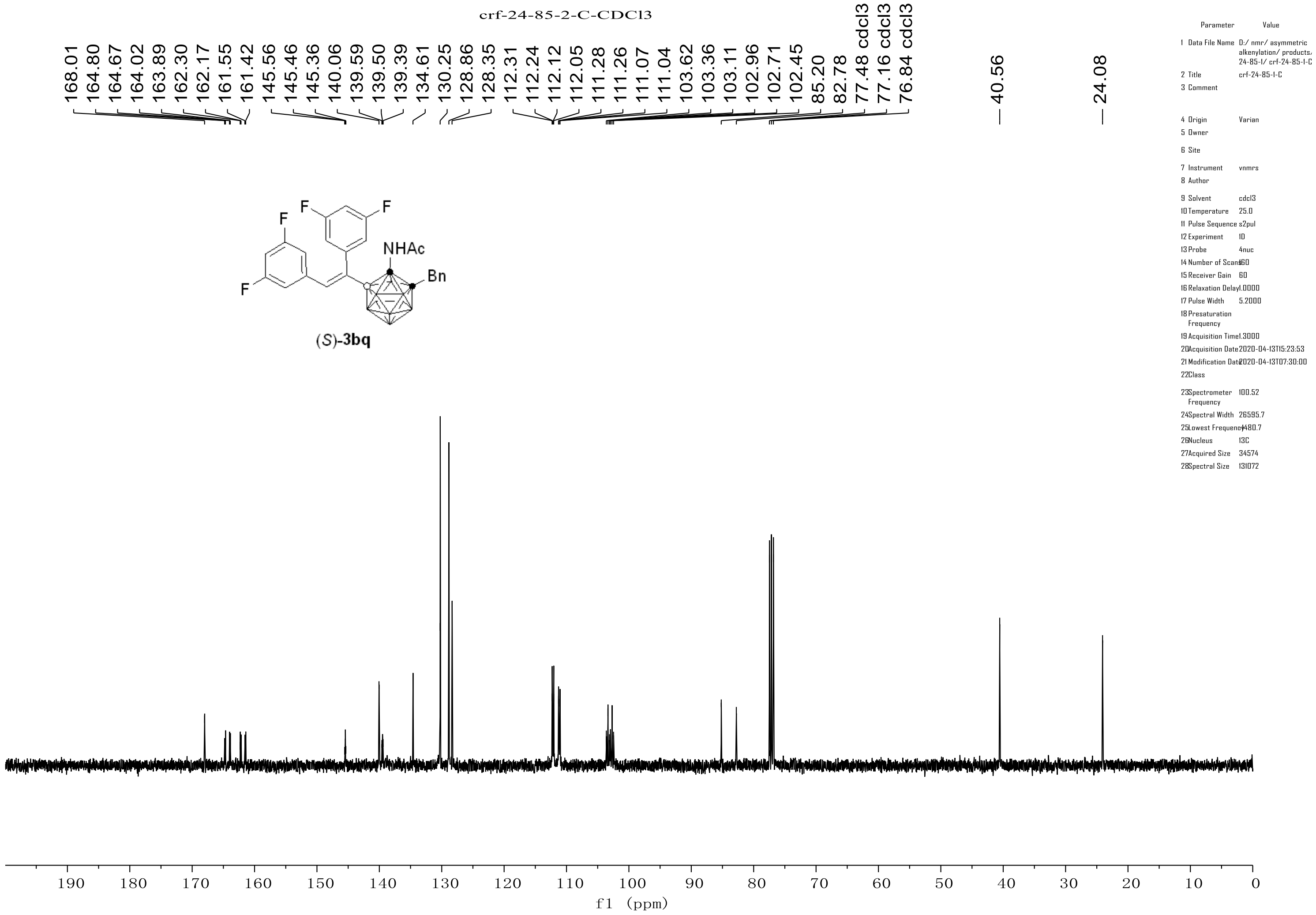

Supplementary Figure 137. $^{11}B\{^{1}H\}$ NMR of (*S*)-**3bq**.

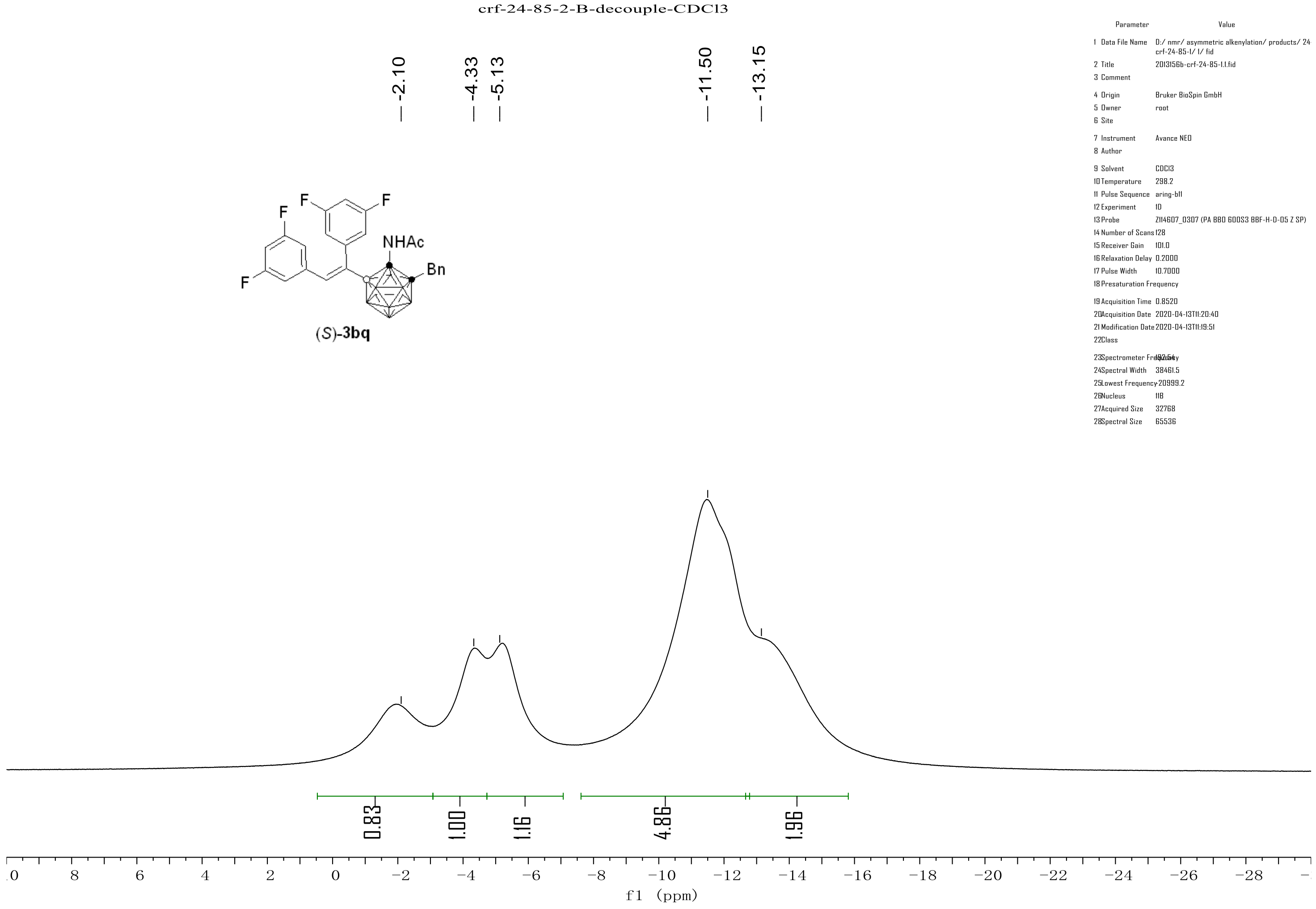

Supplementary Figure 138. ^{11}B NMR of (*S*)-**3bq**.

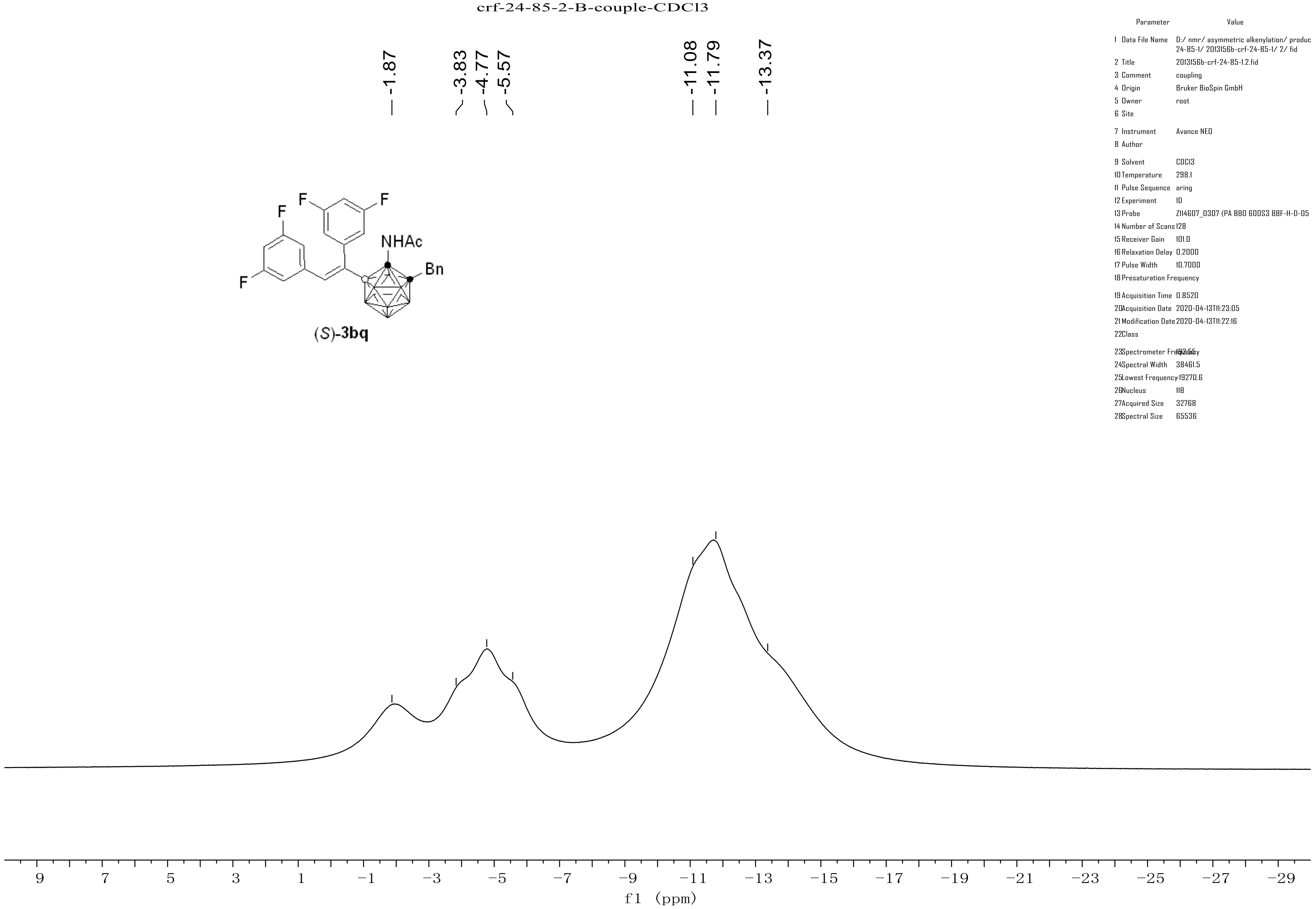

Supplementary Figure 139. ^{19}F NMR of (*S*)-**3bq**.

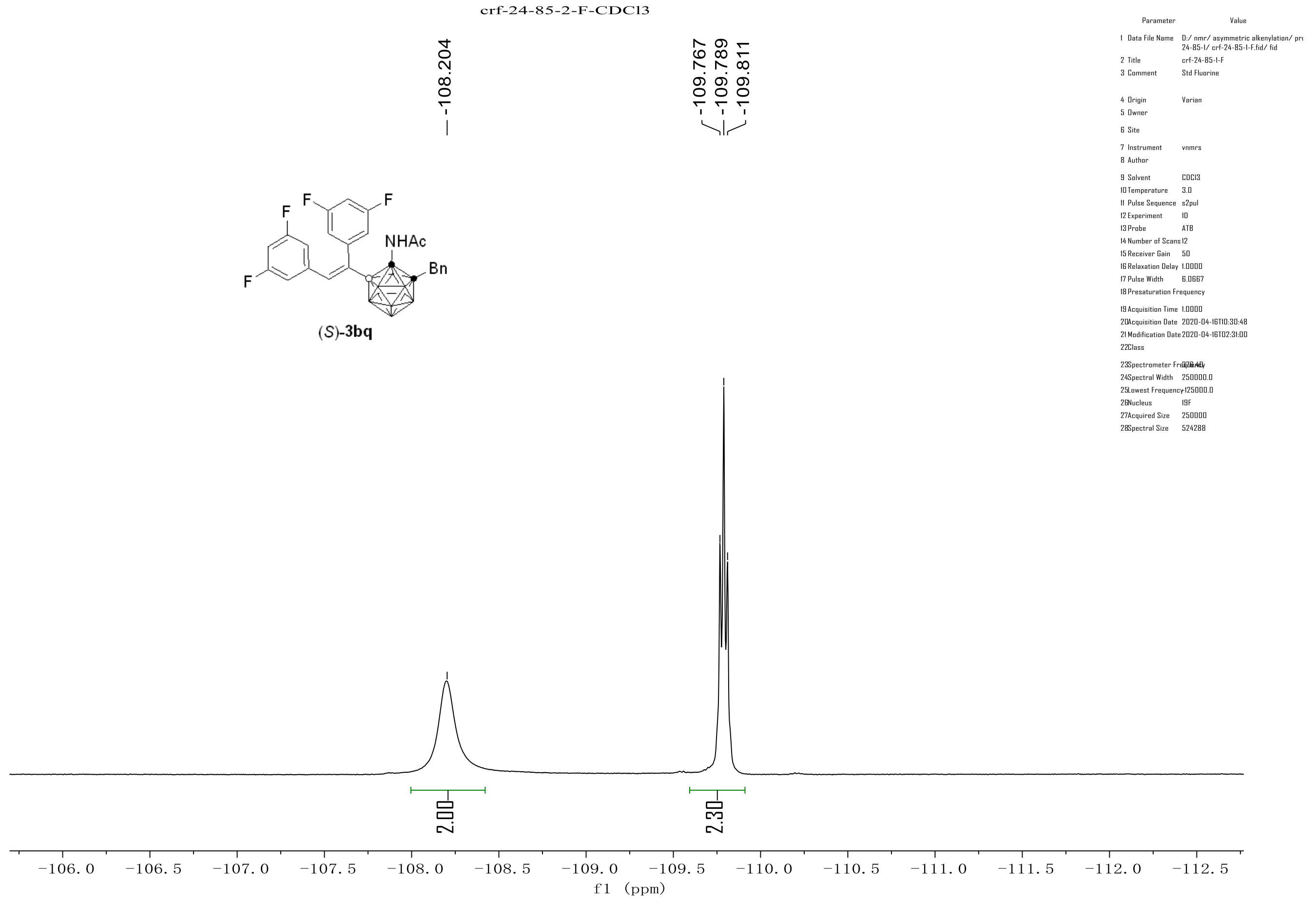

Supplementary Figure 140. ^{1}H NMR of (*S*)-**3br**.

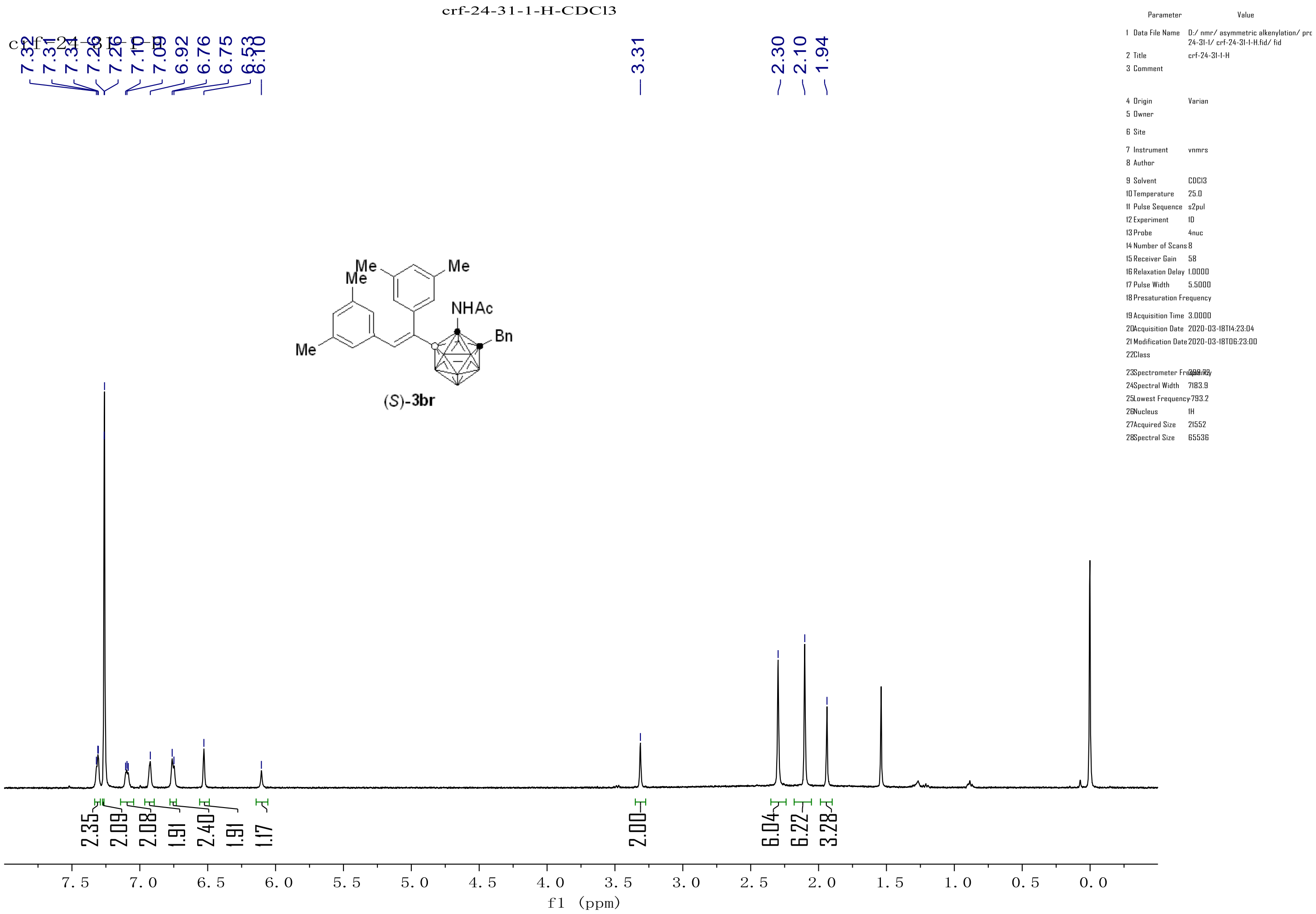

Supplementary Figure 141. $^{13}C\{^{1}H\}$ NMR of (*S*)-**3br**.

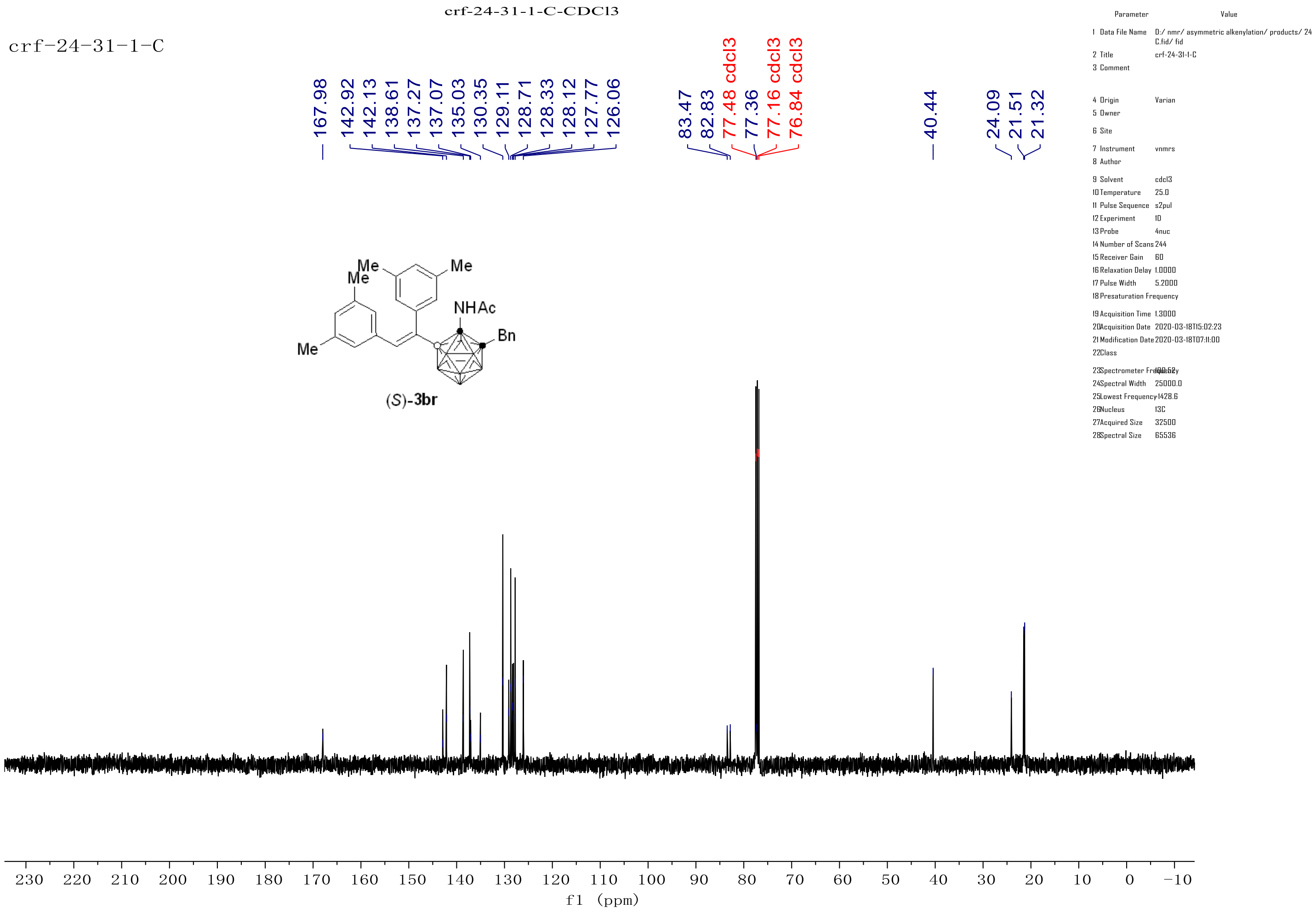

Supplementary Figure 142. $^{11}B\{^1H\}$ NMR of (*S*)-**3br**.

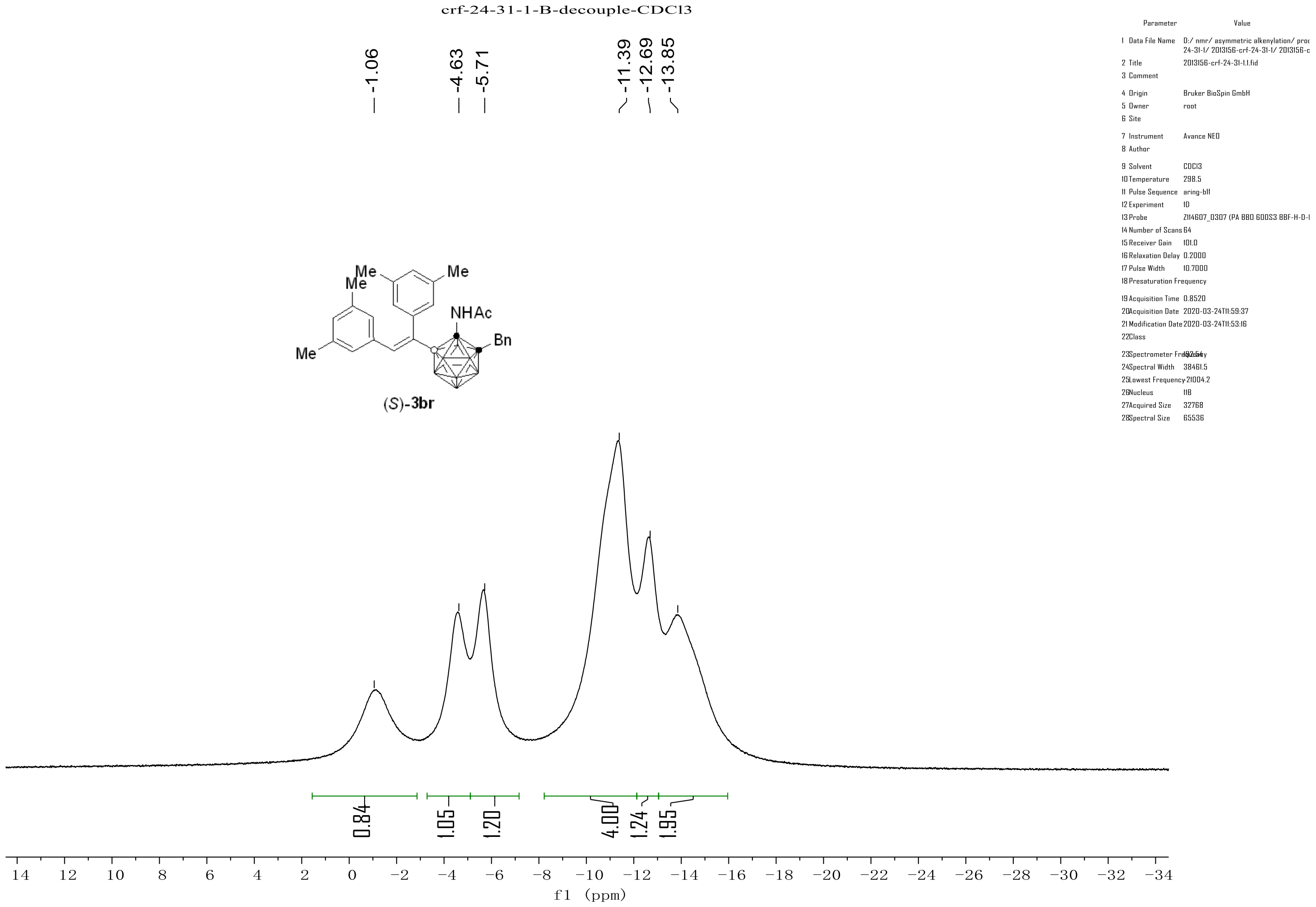

Supplementary Figure 143. ^{11}B NMR of (*S*)-**3br**.

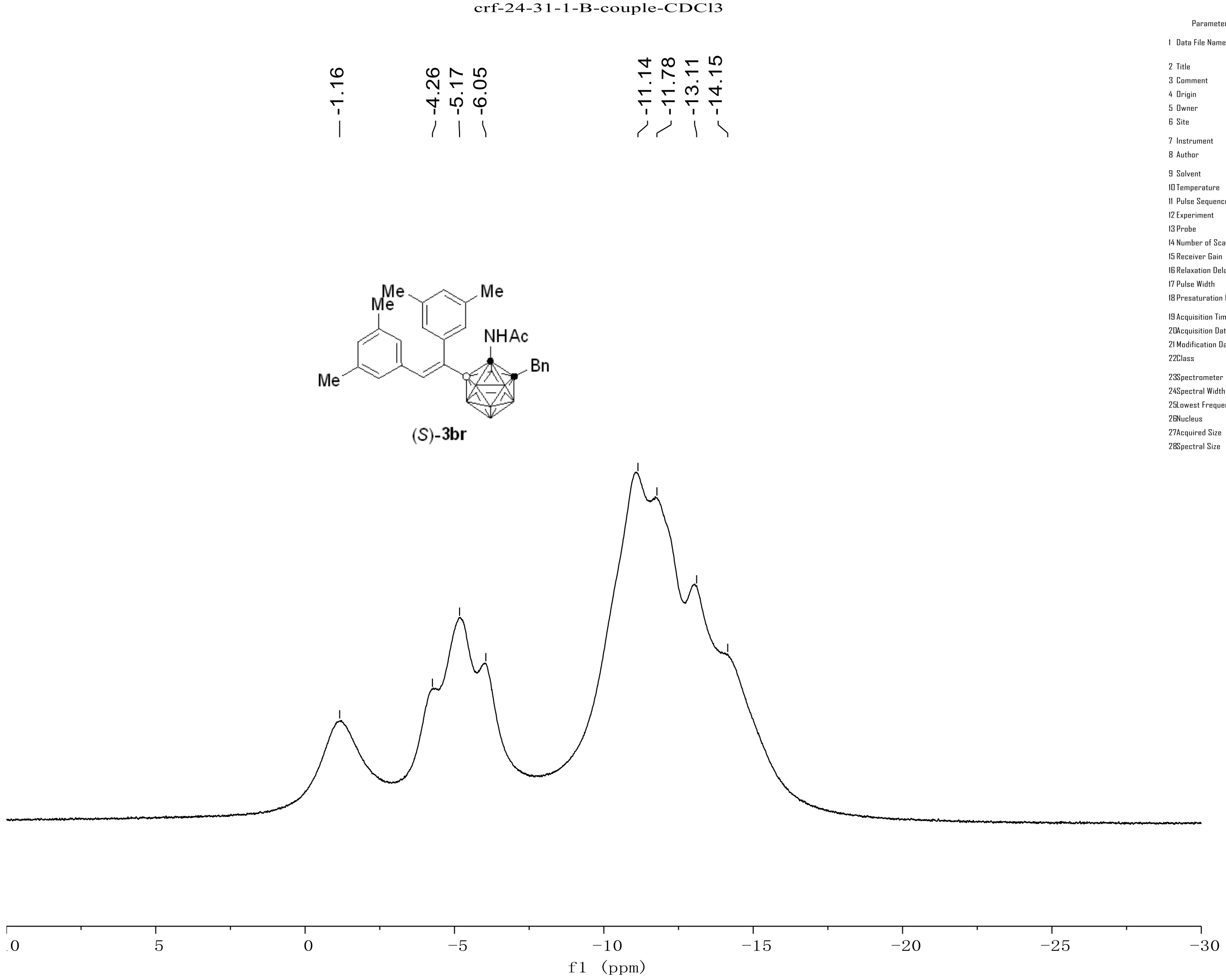

	Parameter	Value
1	Data File Name	D:/ nmr/ asymmetric alkenylation/ products/ 2 crf-24-31-1/ 2013156-crf-24-31-1/ 2/ fid
2	Title	2013156-crf-24-31-1.2.fid
3	Comment	coupling
4	Origin	Bruker BioSpin GmbH
5	Owner	root
6	Site	
7	Instrument	Avance NEO
8	Author	
9	Solvent	CDCl3
10	Temperature	298.1
11	Pulse Sequence	aring
12	Experiment	1D
13	Probe	Z114607_0307 (PA BBO 600S3 BBF-H-D-05 Z SP)
14	Number of Scans	64
15	Receiver Gain	101.0
16	Relaxation Delay	1.0000
17	Pulse Width	10.7000
18	Presaturation Frequency	
19	Acquisition Time	0.8520
20	Acquisition Date	2020-03-24T12:02:10
21	Modification Date	2020-03-24T11:55:49
22	Class	
23	Spectrometer Frequency	192.55
24	Spectral Width	38461.5
25	Lowest Frequency	-19275.6
26	Nucleus	11B
27	Acquired Size	32768
28	Spectral Size	65536

Supplementary Figure 144. ^{1}H NMR of (*S*)-**3bs**.

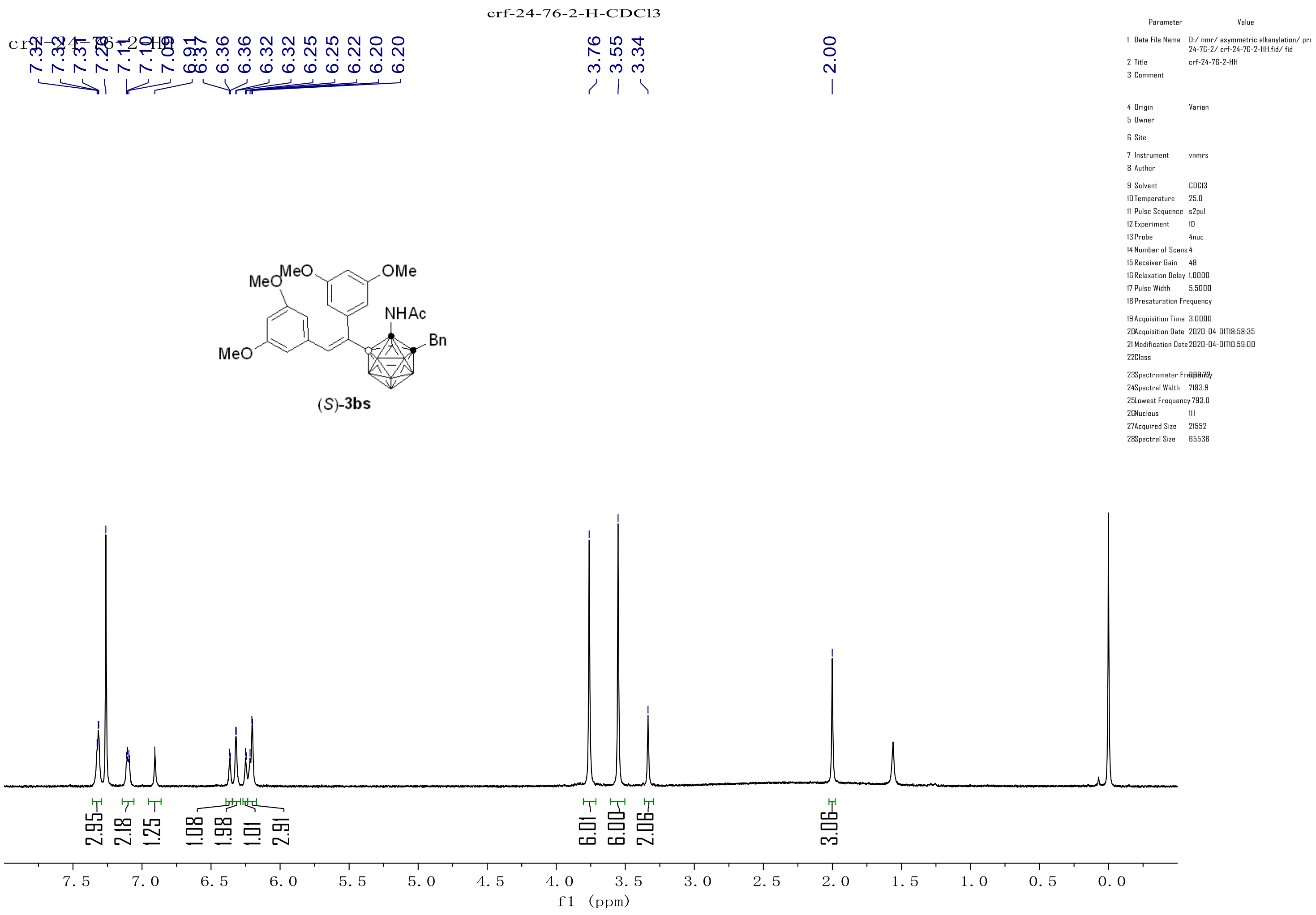

Supplementary Figure 145. $^{13}C\{^{1}H\}$ NMR of (*S*)-**3bs**.

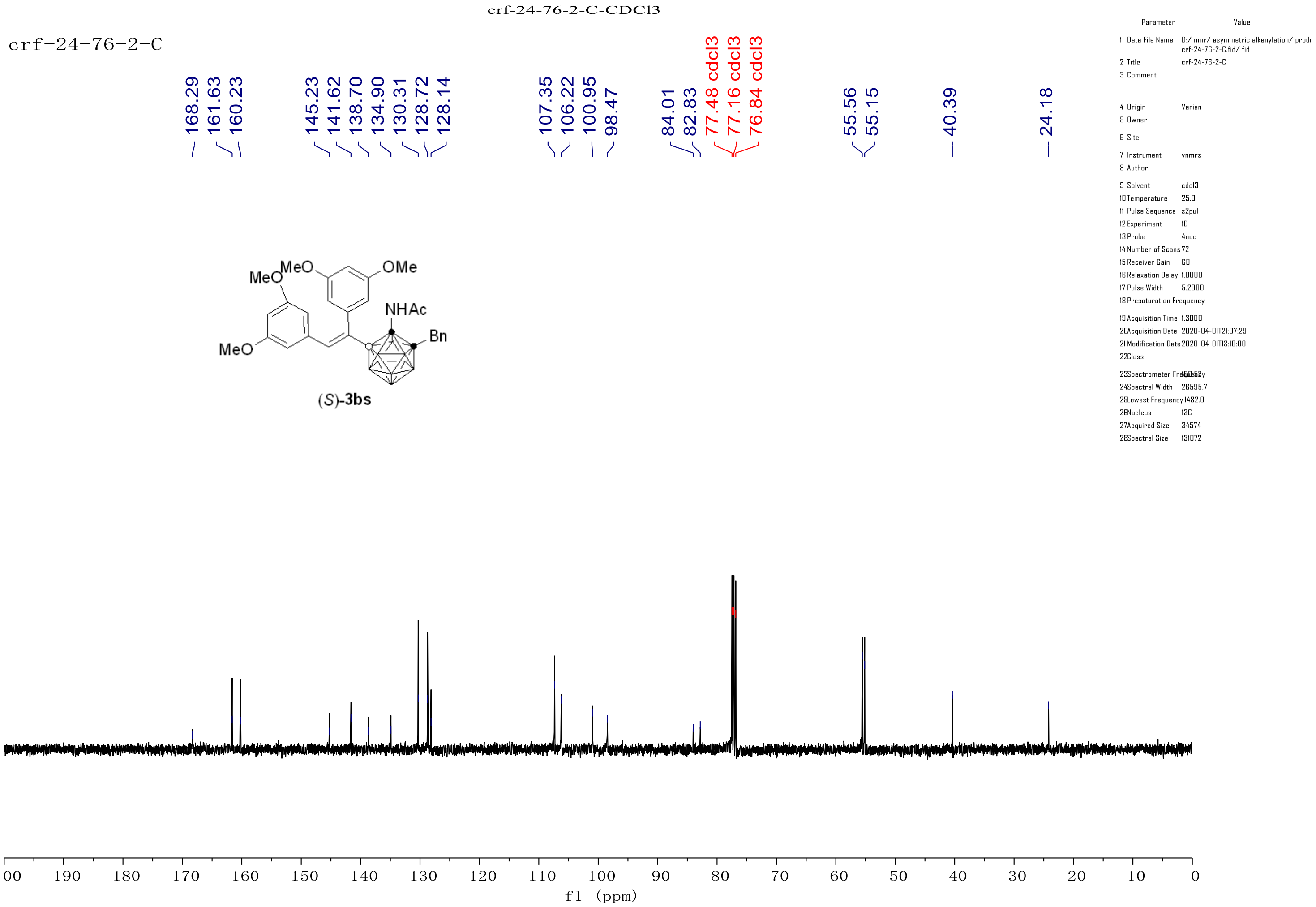

Supplementary Figure 146. $^{11}B\{^{1}H\}$ NMR of (*S*)-**3bs**.

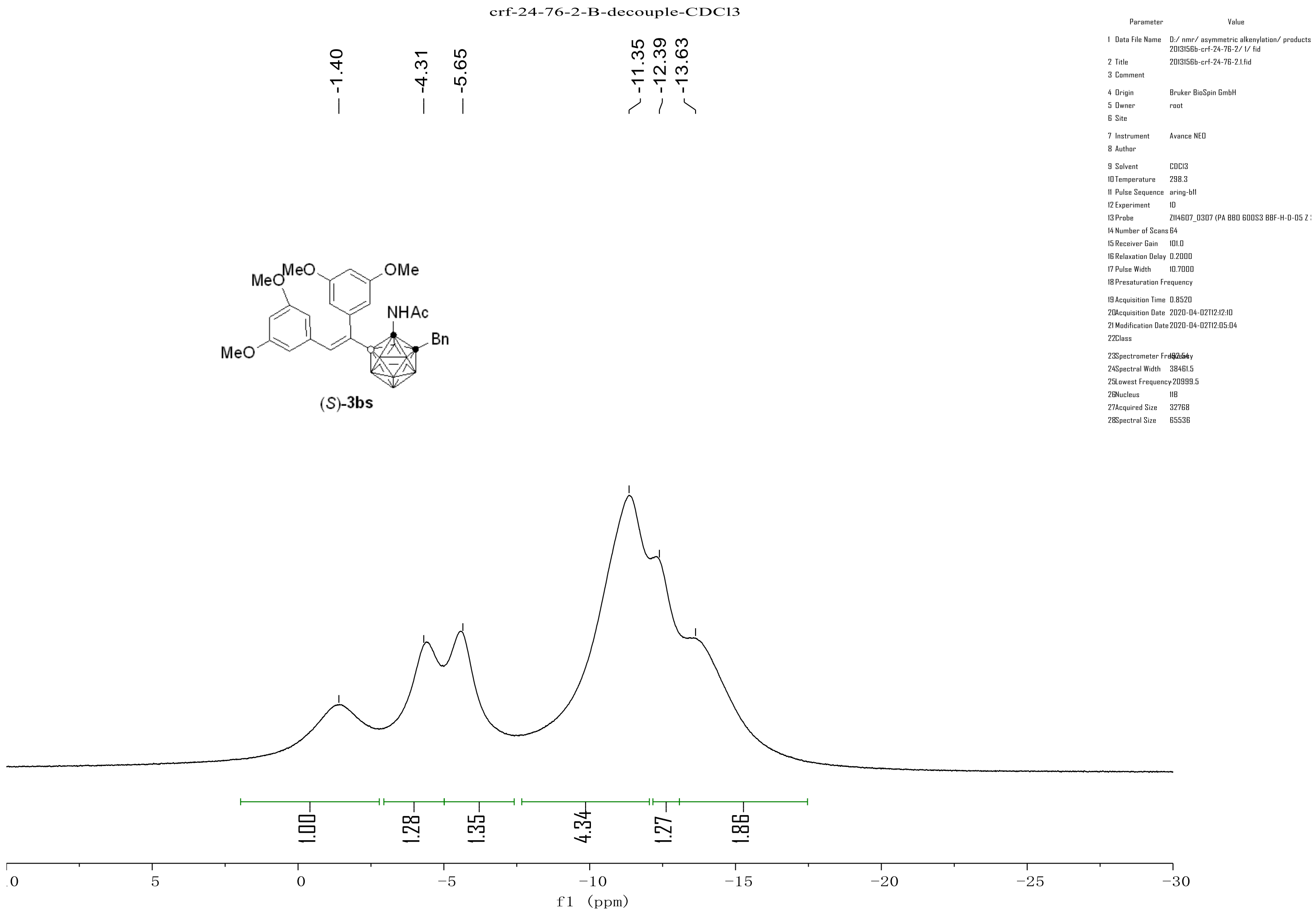

Supplementary Figure 147. ^{11}B NMR of (*S*)-**3bs**.

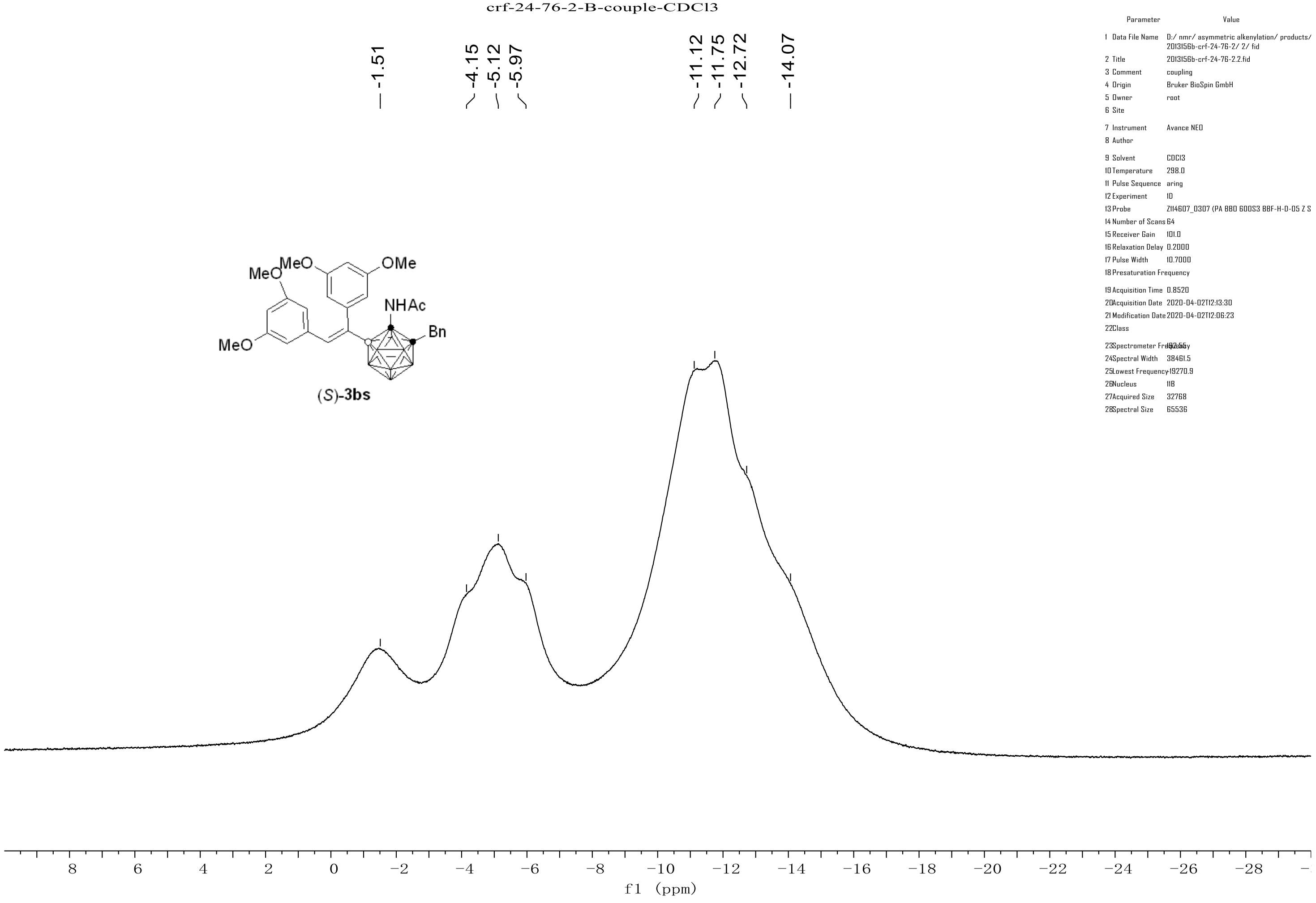

	Parameter	Value
1	Data File Name	D:/ nmr/ asymmetric alkenylation/ products/ 2013156b-crf-24-76-2/ 2/ fid
2	Title	2013156b-crf-24-76-2.2.fid
3	Comment	coupling
4	Origin	Bruker BioSpin GmbH
5	Owner	root
6	Site	
7	Instrument	Avance NEO
8	Author	
9	Solvent	CDCl3
10	Temperature	298.0
11	Pulse Sequence	aring
12	Experiment	1D
13	Probe	Z114607_0307 (PA BBO 600S3 BBF-H-D-05 Z S
14	Number of Scans	64
15	Receiver Gain	101.0
16	Relaxation Delay	0.2000
17	Pulse Width	10.7000
18	Presaturation Frequency	
19	Acquisition Time	0.8520
20	Acquisition Date	2020-04-02T12:13:30
21	Modification Date	2020-04-02T12:06:23
22	Class	
23	Spectrometer Frequency	192.55
24	Spectral Width	38461.5
25	Lowest Frequency	-19270.9
26	Nucleus	11B
27	Acquired Size	32768
28	Spectral Size	65536

Supplementary Figure 148. ^{1}H NMR of (*S*)-**3bt**.

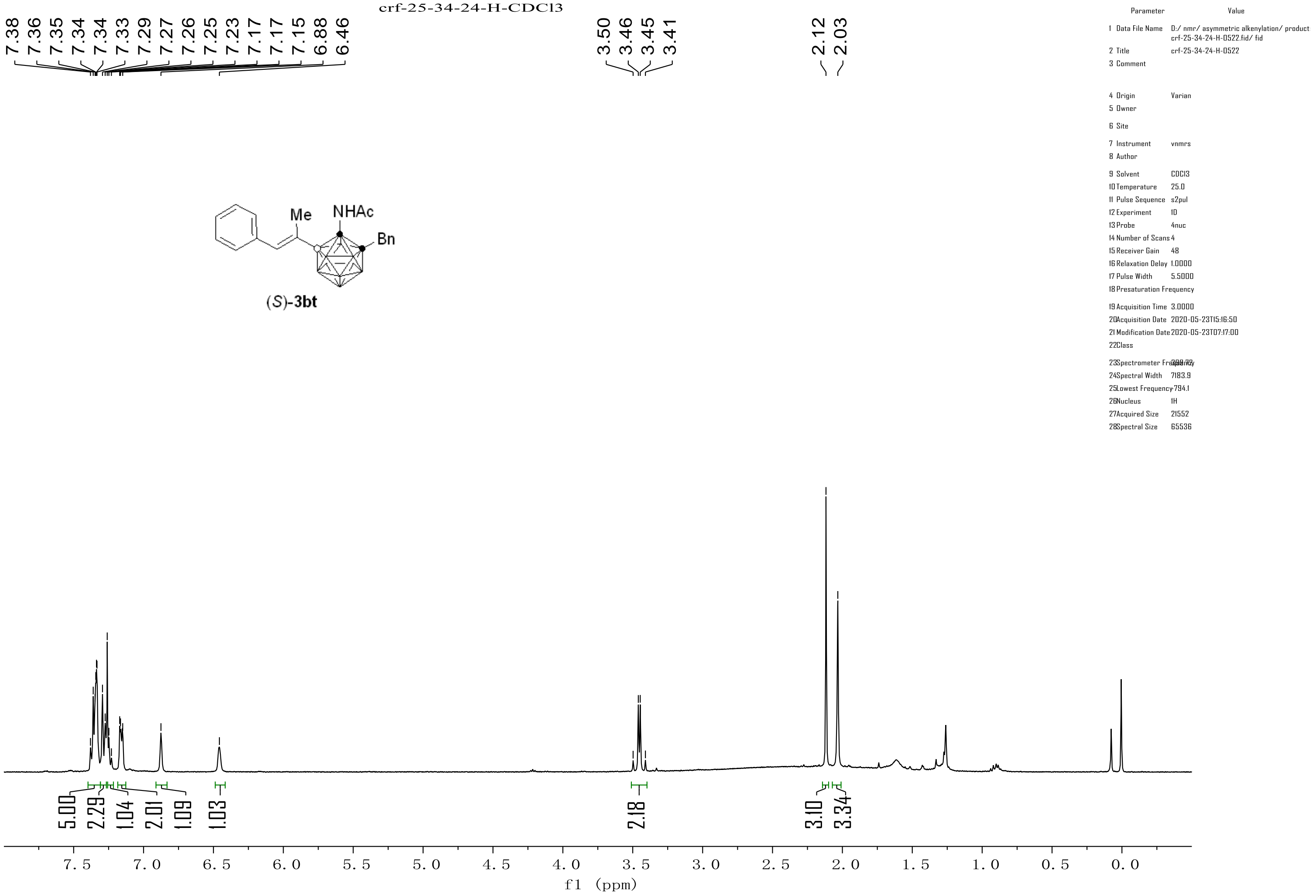

Supplementary Figure 149. $^{13}C\{^{1}H\}$ NMR of (*S*)-**3bt**.

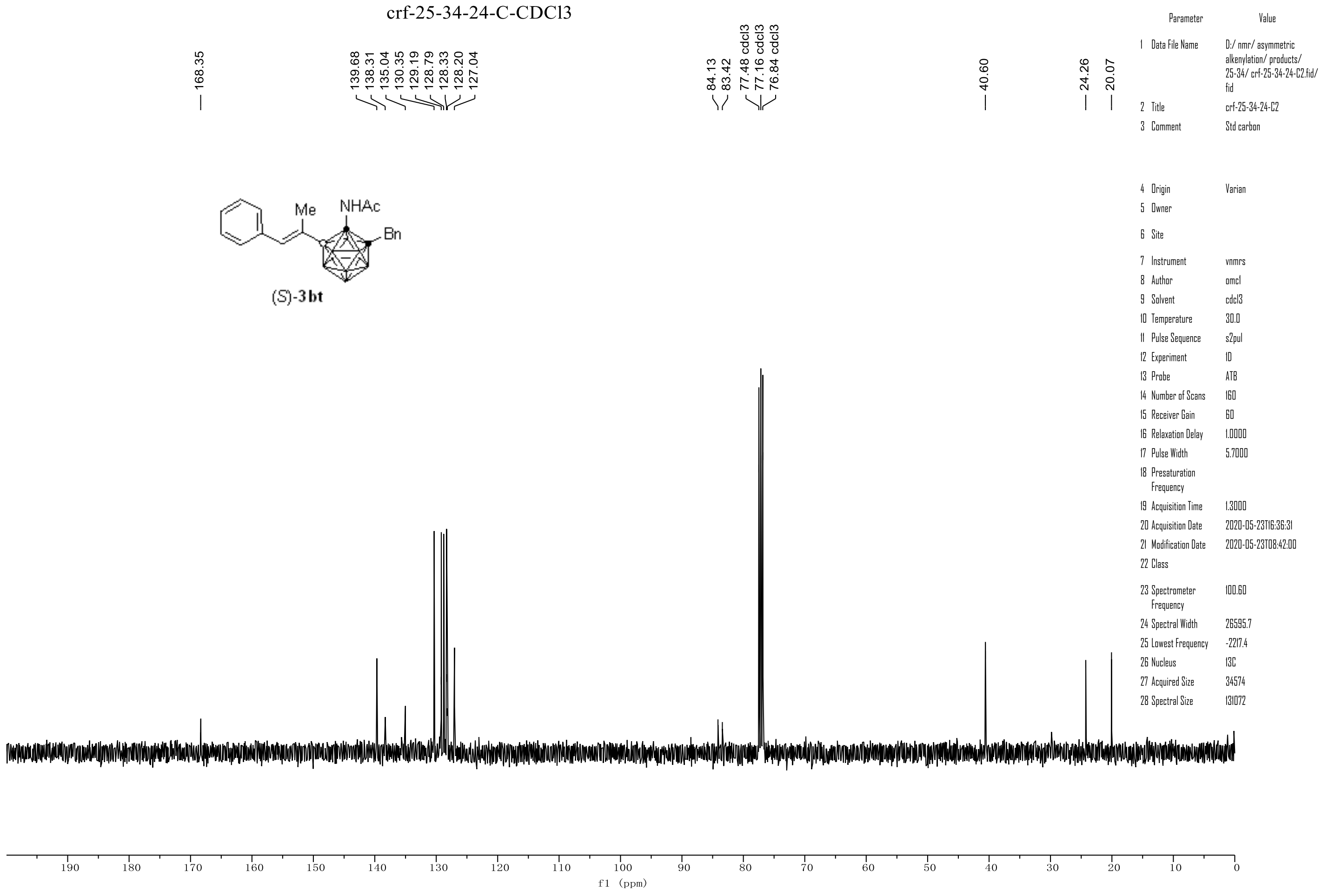

Supplementary Figure 150. $^{11}B\{^{1}H\}$ NMR of (*S*)-**3bt**.

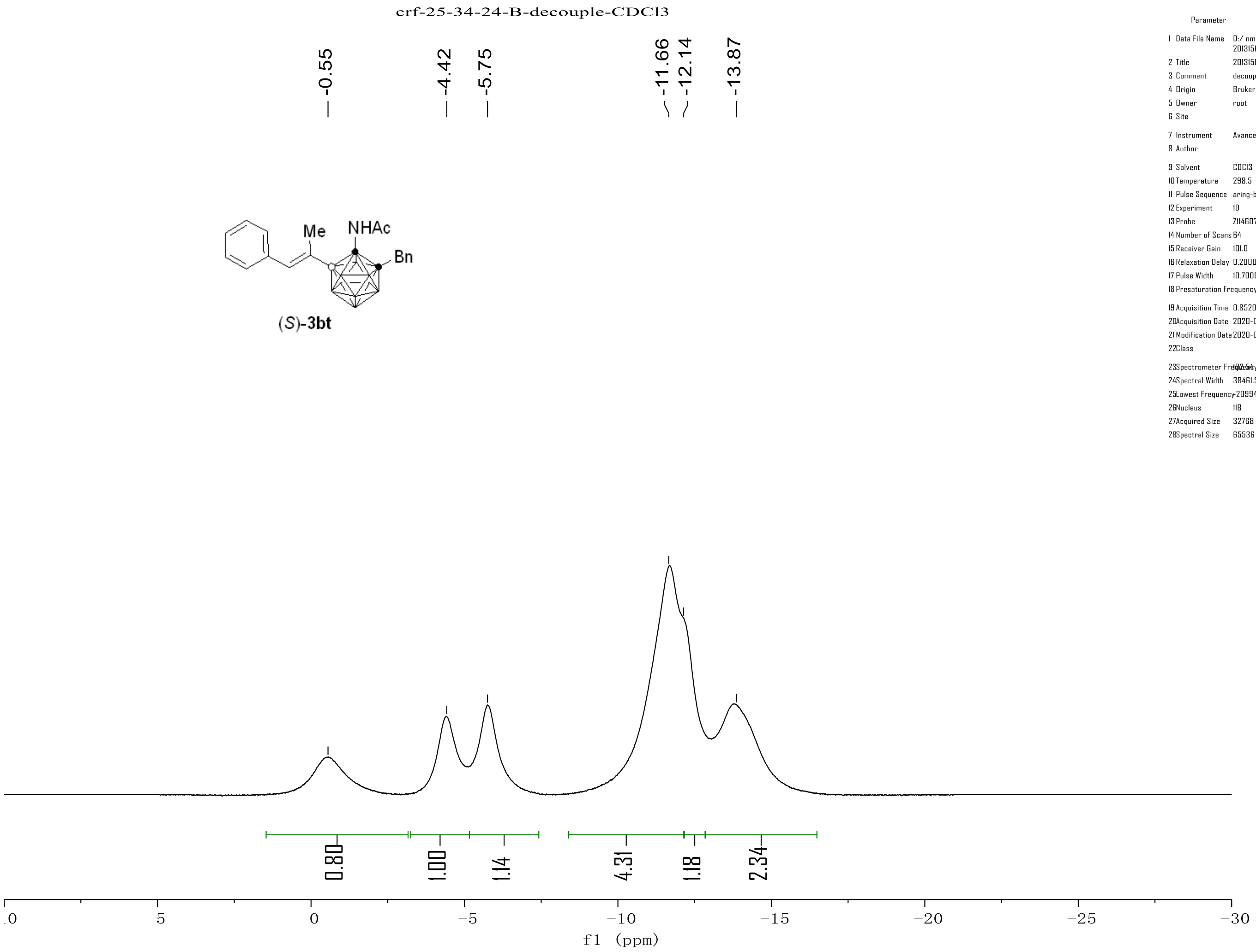

Parameter	Value
1 Data File Name	D:/ nmr/ asymmetric alkenylation/ prod 2013156-crf-25-34-24/ 1/ fid
2 Title	2013156-crf-25-34-24.1.fid
3 Comment	decoupling
4 Origin	Bruker BioSpin GmbH
5 Owner	root
6 Site	
7 Instrument	Avance NEO
8 Author	
9 Solvent	CDCl3
10 Temperature	298.5
11 Pulse Sequence	aring-b11
12 Experiment	1D
13 Probe	Z114607_0307 (PA BBO 600S3 BBF-H-D-0
14 Number of Scans	64
15 Receiver Gain	101.0
16 Relaxation Delay	0.2000
17 Pulse Width	10.7000
18 Presaturation Frequency	
19 Acquisition Time	0.8520
20 Acquisition Date	2020-05-25T12:52:24
21 Modification Date	2020-05-25T12:52:09
22 Class	
23 Spectrometer Frequency	192.54
24 Spectral Width	38461.5
25 Lowest Frequency	-20994.6
26 Nucleus	11B
27 Acquired Size	32768
28 Spectral Size	65536

Supplementary Figure 151. ^{11}B NMR of (*S*)-**3bt**.

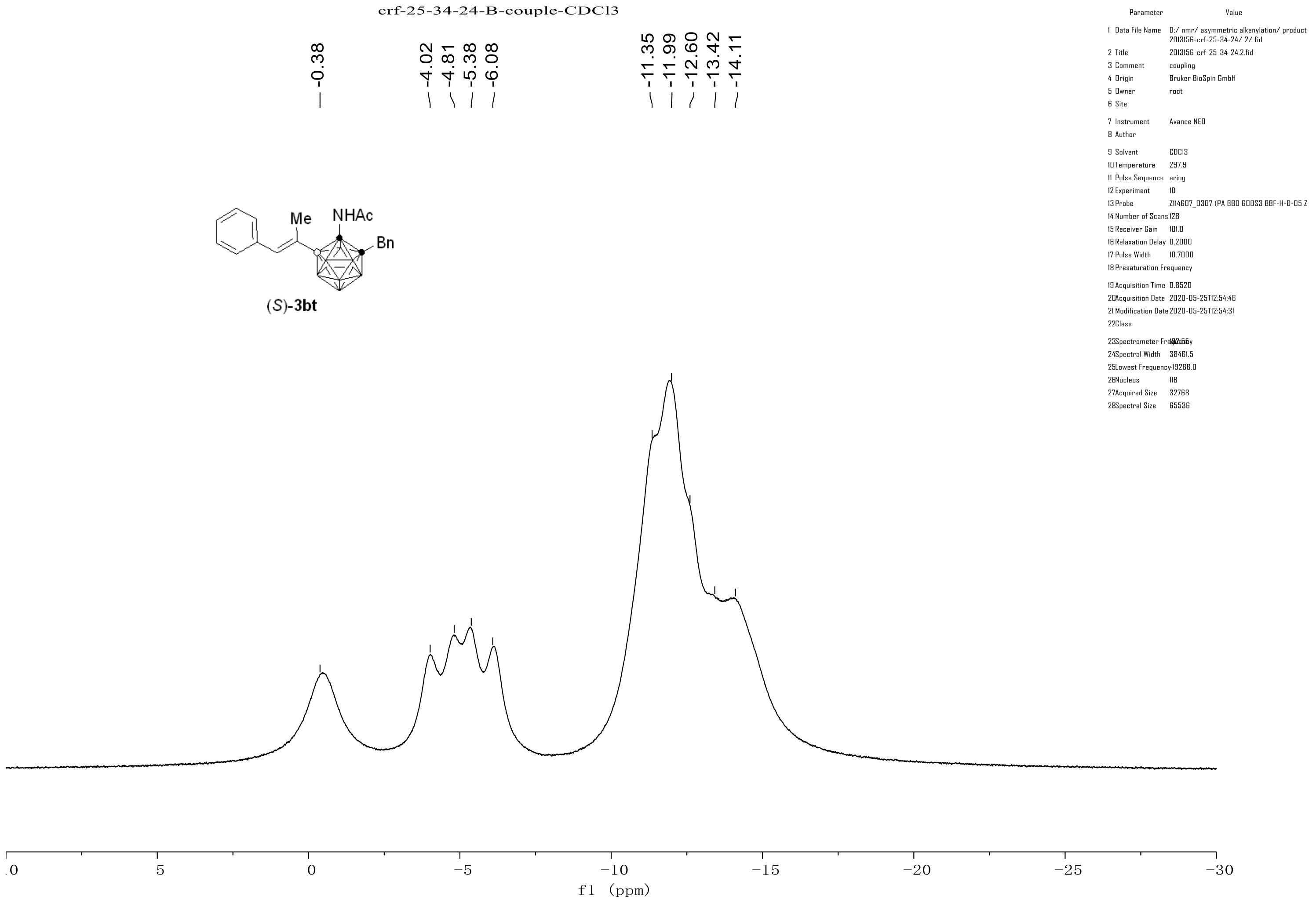

Supplementary Figure 152. NOESY NMR of (*S*)-**3bt**.

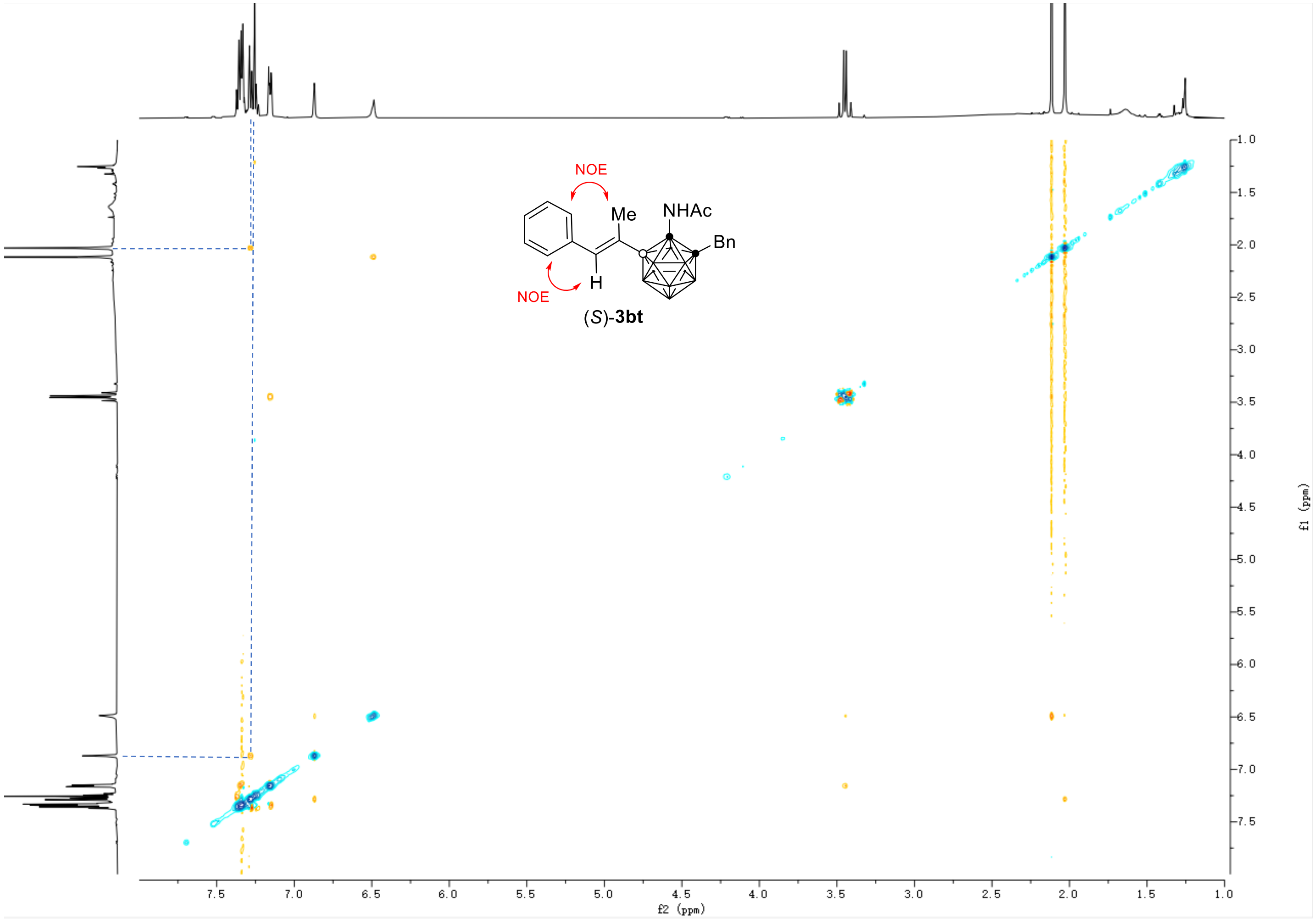

Supplementary Figure 153. ^{1}H NMR of (*S*)-**3bt'**.

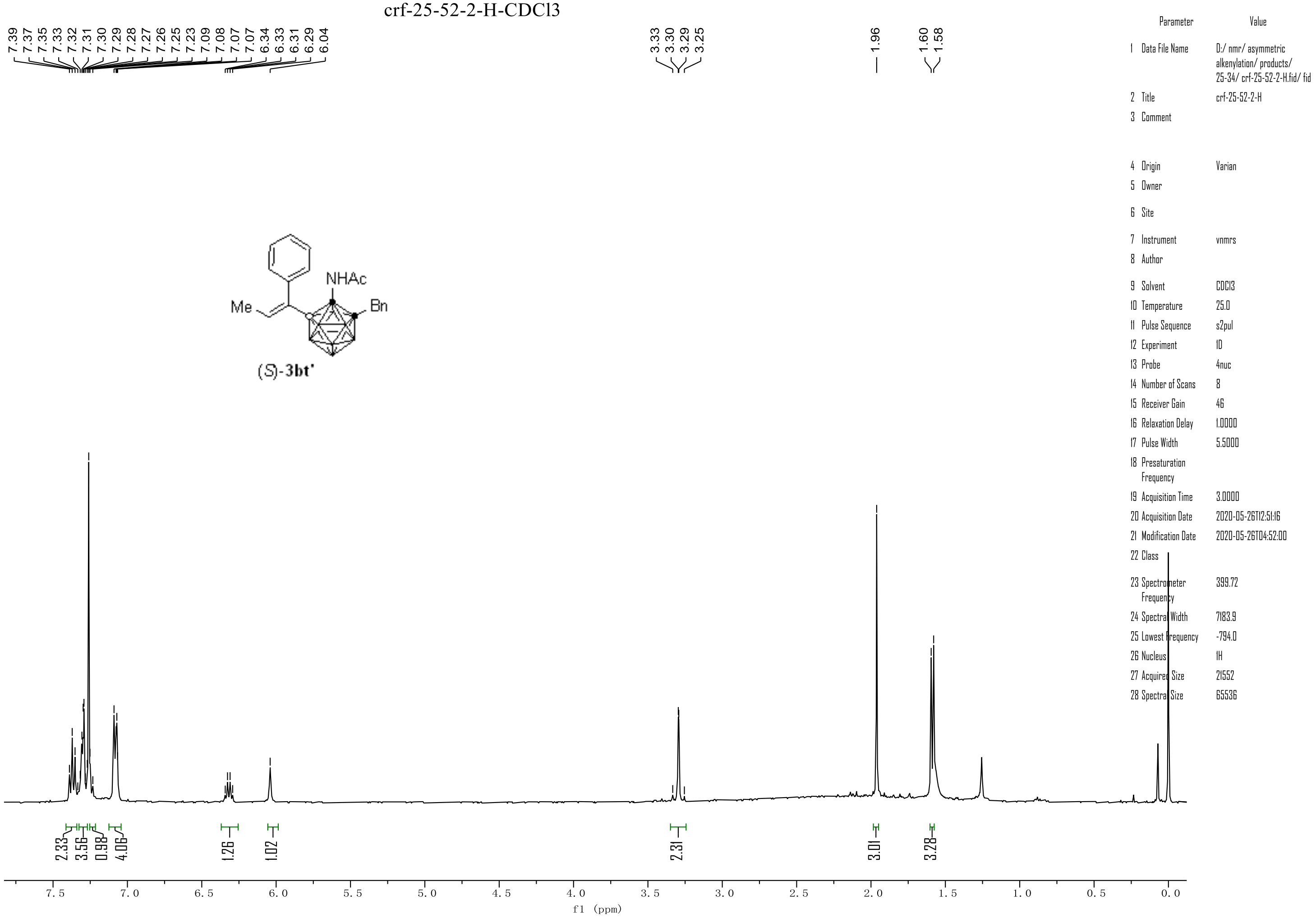

Supplementary Figure 154. $^{13}C\{^{1}H\}$ NMR of (*S*)-**3bt'**.

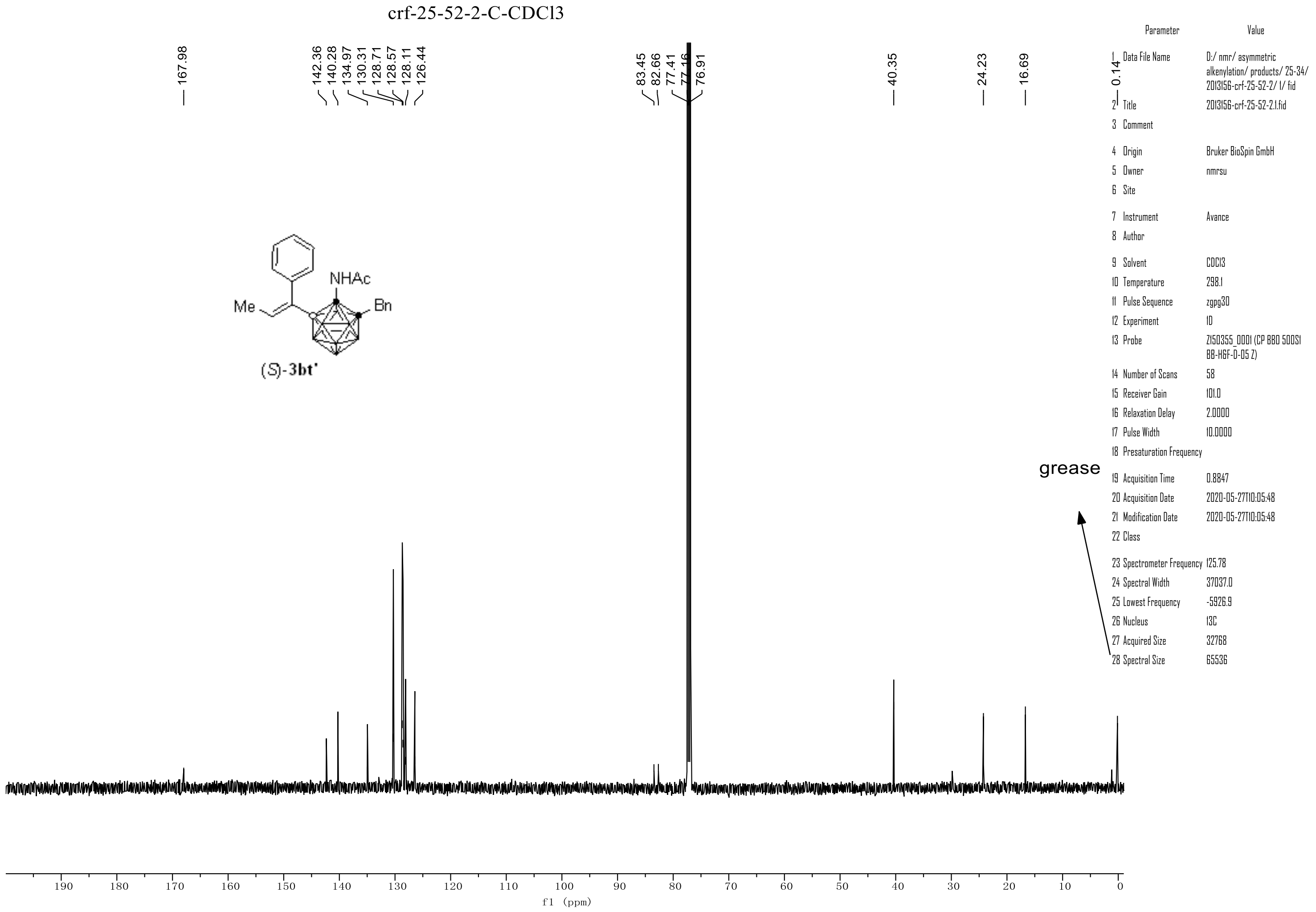

Supplementary Figure 155. $^{11}B\{^{1}H\}$ NMR of (*S*)-**3bt’**.

crf-25-52-2-B-decouple-CDCl3

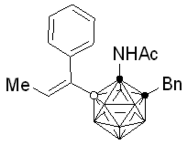

(*S*)-**3bt'**

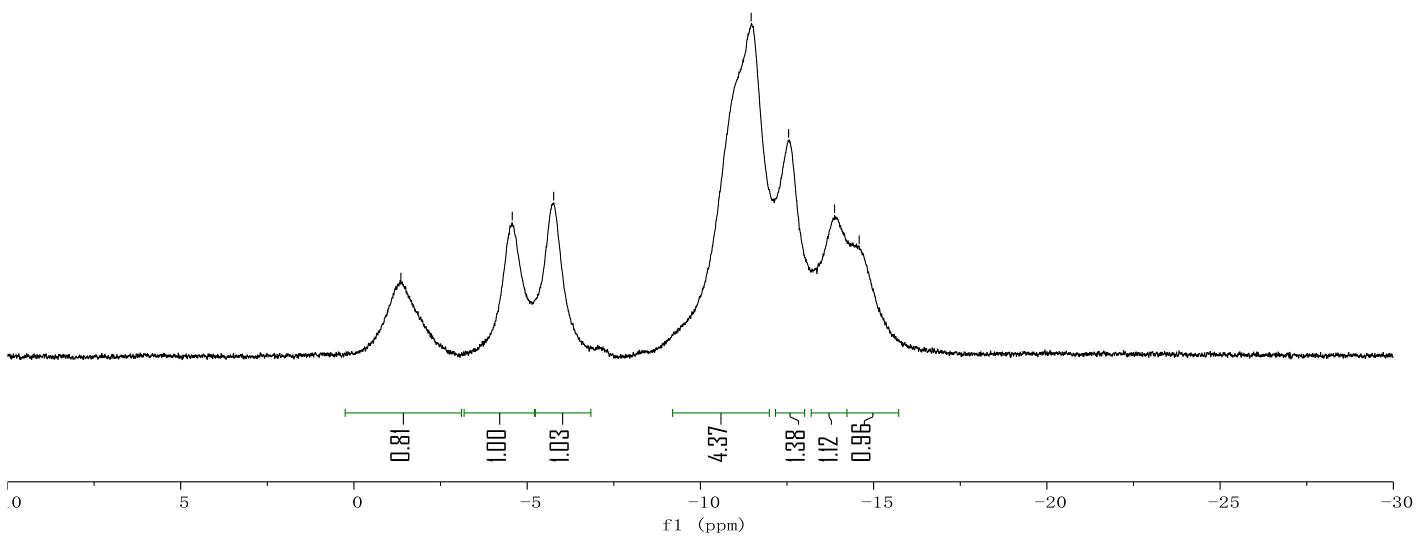

Parameter	Value
1 Data File Name	D:/ nmr/ asymmetric alkenylation/ products/ 25-crf-25-52-2/ 1/ fid
2 Title	2013156-crf-25-52-2.1.fid
3 Comment	decoupling
4 Origin	Bruker BioSpin GmbH
5 Owner	root
6 Site	
7 Instrument	Avance NEO
8 Author	
9 Solvent	CDCl3
10 Temperature	298.3
11 Pulse Sequence	aring-b11
12 Experiment	1D
13 Probe	Z114607_0307 (PA BBO 600S3 BBF-H-D-05 Z SP)
14 Number of Scans	64
15 Receiver Gain	101.0
16 Relaxation Delay	0.2000
17 Pulse Width	10.7000
18 Presaturation Frequency	
19 Acquisition Time	0.8520
20 Acquisition Date	2020-05-26T15:41:23
21 Modification Date	2020-05-26T15:41:03
22 Class	
23 Spectrometer Frequency	192.54
24 Spectral Width	38461.5
25 Lowest Frequency	-20994.6
26 Nucleus	11B
27 Acquired Size	32768
28 Spectral Size	65536

Supplementary Figure 156. ^{11}B NMR of (*S*)-**3bt'**.

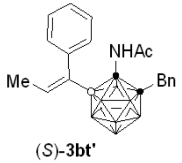

(*S*)-**3bt'**

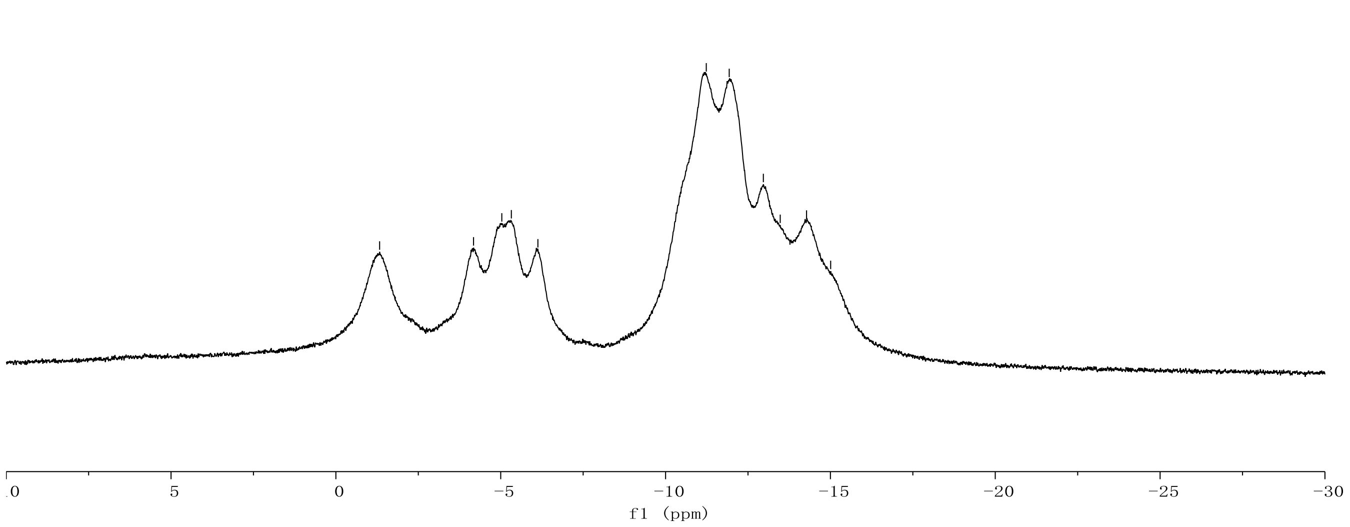

	Parameter	Value
1	Data File Name	D:/ nmr/ asymmetric alkenylation/ products/ 25-3 2/ fid
2	Title	2013156-crf-25-52-2.2.fid
3	Comment	coupling
4	Origin	Bruker BioSpin GmbH
5	Owner	root
6	Site	
7	Instrument	Avance NEO
8	Author	
9	Solvent	CDCl3
10	Temperature	298.0
11	Pulse Sequence	aring
12	Experiment	1D
13	Probe	Z114607_0307 (PA BBO 600S3 BBF-H-D-05 Z SP)
14	Number of Scans	110
15	Receiver Gain	101.0
16	Relaxation Delay	0.2000
17	Pulse Width	10.7000
18	Presaturation Frequency	
19	Acquisition Time	0.8520
20	Acquisition Date	2020-05-26T15:43:39
21	Modification Date	2020-05-26T15:43:19
22	Class	
23	Spectrometer Frequency	192.55
24	Spectral Width	38461.5
25	Lowest Frequency	-19266.0
26	Nucleus	11B
27	Acquired Size	32768
28	Spectral Size	65536

Supplementary Figure 157. NOESY NMR of (*S*)-**3bt'**.

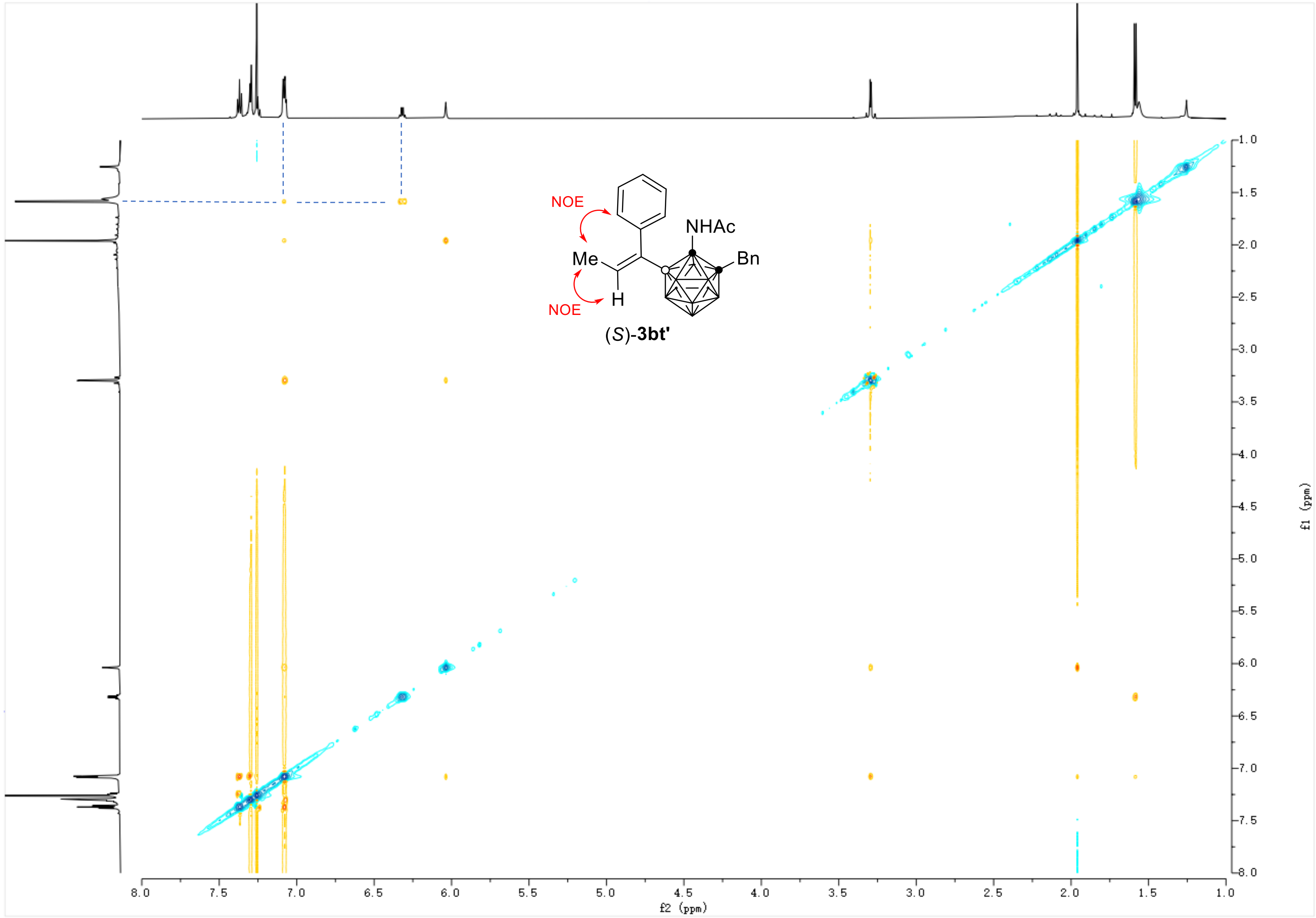

Supplementary Figure 158. ^{1}H NMR of (*S*)-**3ca**.

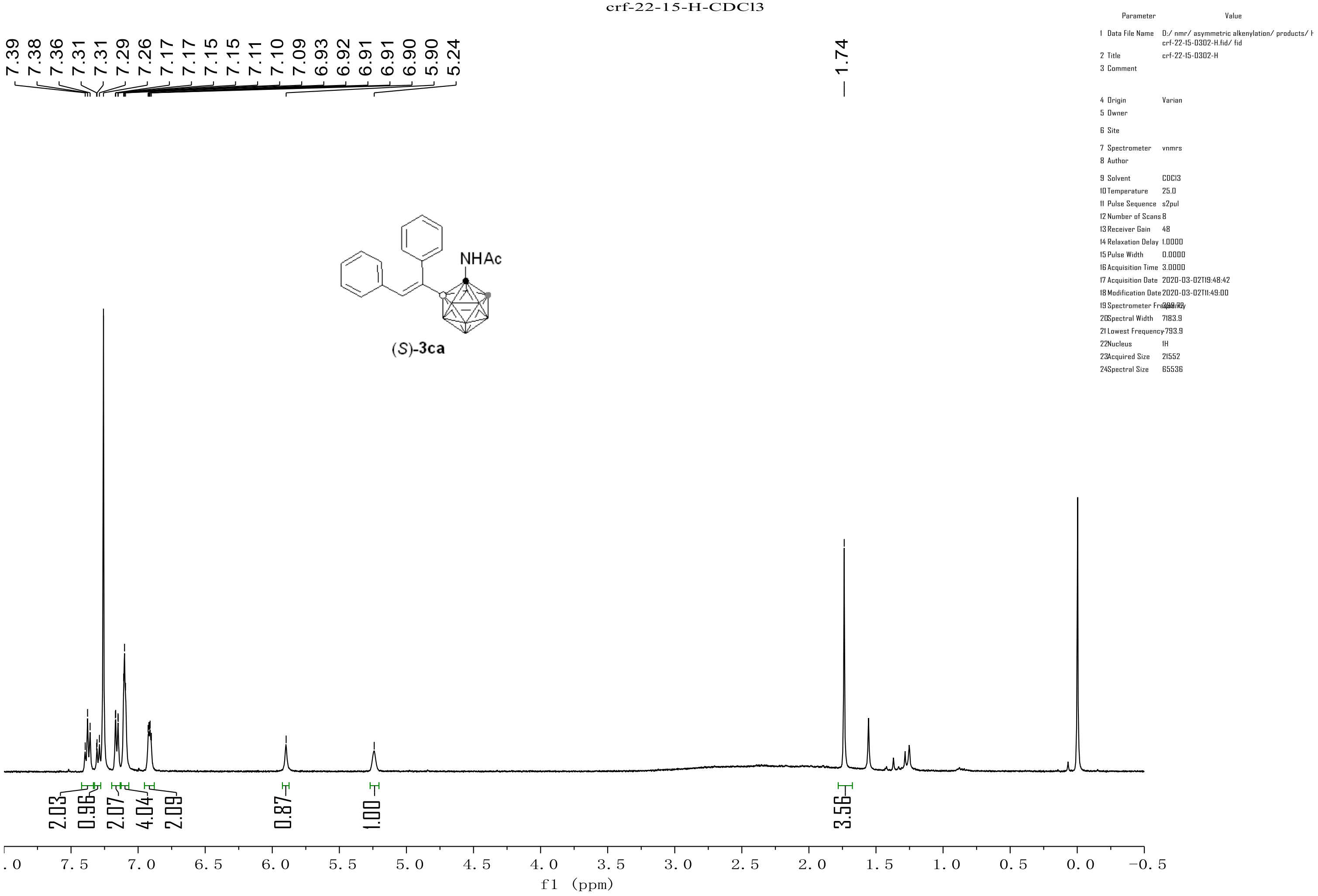

Supplementary Figure 159. $^{13}C\{^{1}H\}$ NMR of (*S*)-**3ca**.

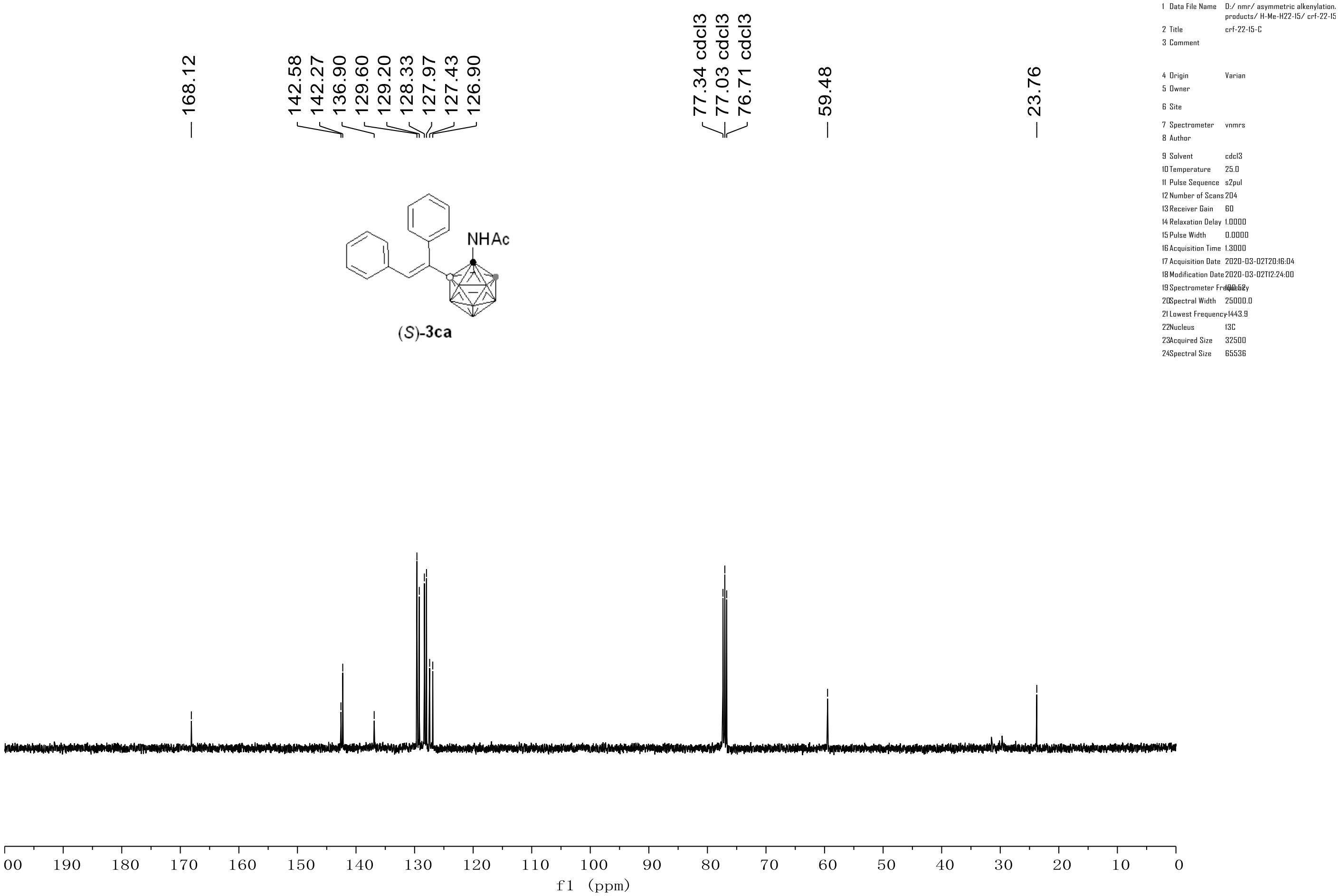

Parameter	Value
1 Data File Name	D:/ nmr/ asymmetric alkenylation. products/ H-Me-H22-15/ crf-22-15
2 Title	crf-22-15-C
3 Comment	
4 Origin	Varian
5 Owner	
6 Site	
7 Spectrometer	vnmrs
8 Author	
9 Solvent	cdcl3
10 Temperature	25.0
11 Pulse Sequence	s2pul
12 Number of Scans	204
13 Receiver Gain	60
14 Relaxation Delay	1.0000
15 Pulse Width	0.0000
16 Acquisition Time	1.3000
17 Acquisition Date	2020-03-02T20:16:04
18 Modification Date	2020-03-02T12:24:00
19 Spectrometer Frequency	100.52
20 Spectral Width	25000.0
21 Lowest Frequency	-1443.9
22 Nucleus	13C
23 Acquired Size	32500
24 Spectral Size	65536

Supplementary Figure 160. $^{11}B\{^{1}H\}$ NMR of (*S*)-**3ca**.

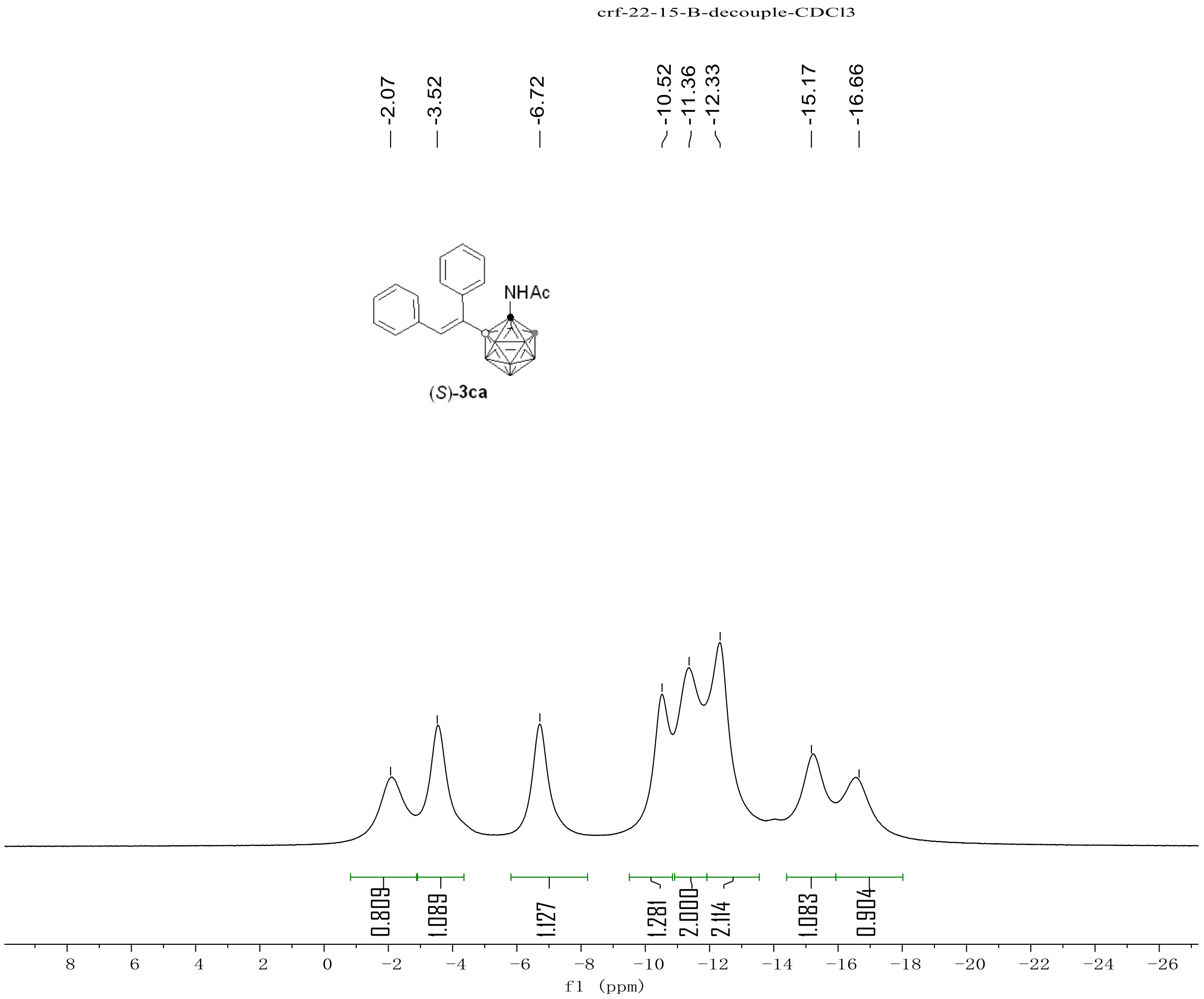

	Parameter	Value
1	Data File Name	D:/ nmr/ asymmetric alkenylation/ product 2013156-crf-22-15/ 2/ fid
2	Title	2013156-crf-22-15.2.fid
3	Comment	
4	Origin	Bruker BioSpin GmbH
5	Owner	root
6	Site	
7	Instrument	Avance NEO
8	Author	
9	Solvent	CDCl3
10	Temperature	301.3
11	Pulse Sequence	aring-b11
12	Experiment	1D
13	Probe	Z114607_0307 (PA BBO 600S3 BBF-H-D-05 Z
14	Number of Scans	64
15	Receiver Gain	101.0
16	Relaxation Delay	1.0000
17	Pulse Width	10.7000
18	Presaturation Frequency	
19	Acquisition Time	0.8520
20	Acquisition Date	2020-03-03T10:32:19
21	Modification Date	2020-03-03T10:27:43
22	Class	
23	Spectrometer Frequency	192.54
24	Spectral Width	38461.5
25	Lowest Frequency	-21006.4
26	Nucleus	11B
27	Acquired Size	32768
28	Spectral Size	65536

Supplementary Figure 161. ^{11}B NMR of (*S*)-**3ca**.

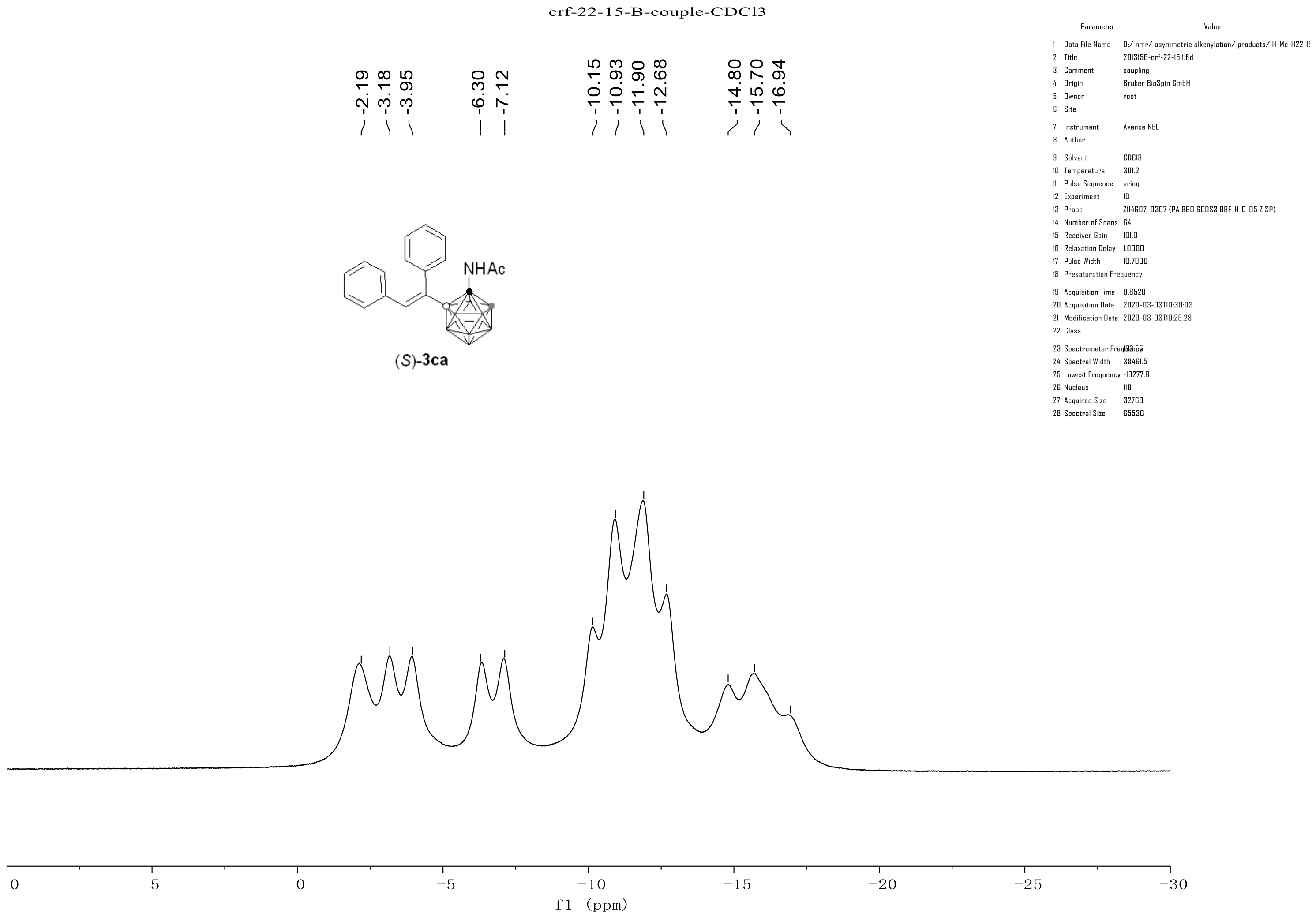

Supplementary Figure 162. ^{1}H NMR of (*S*)-**3aa**.

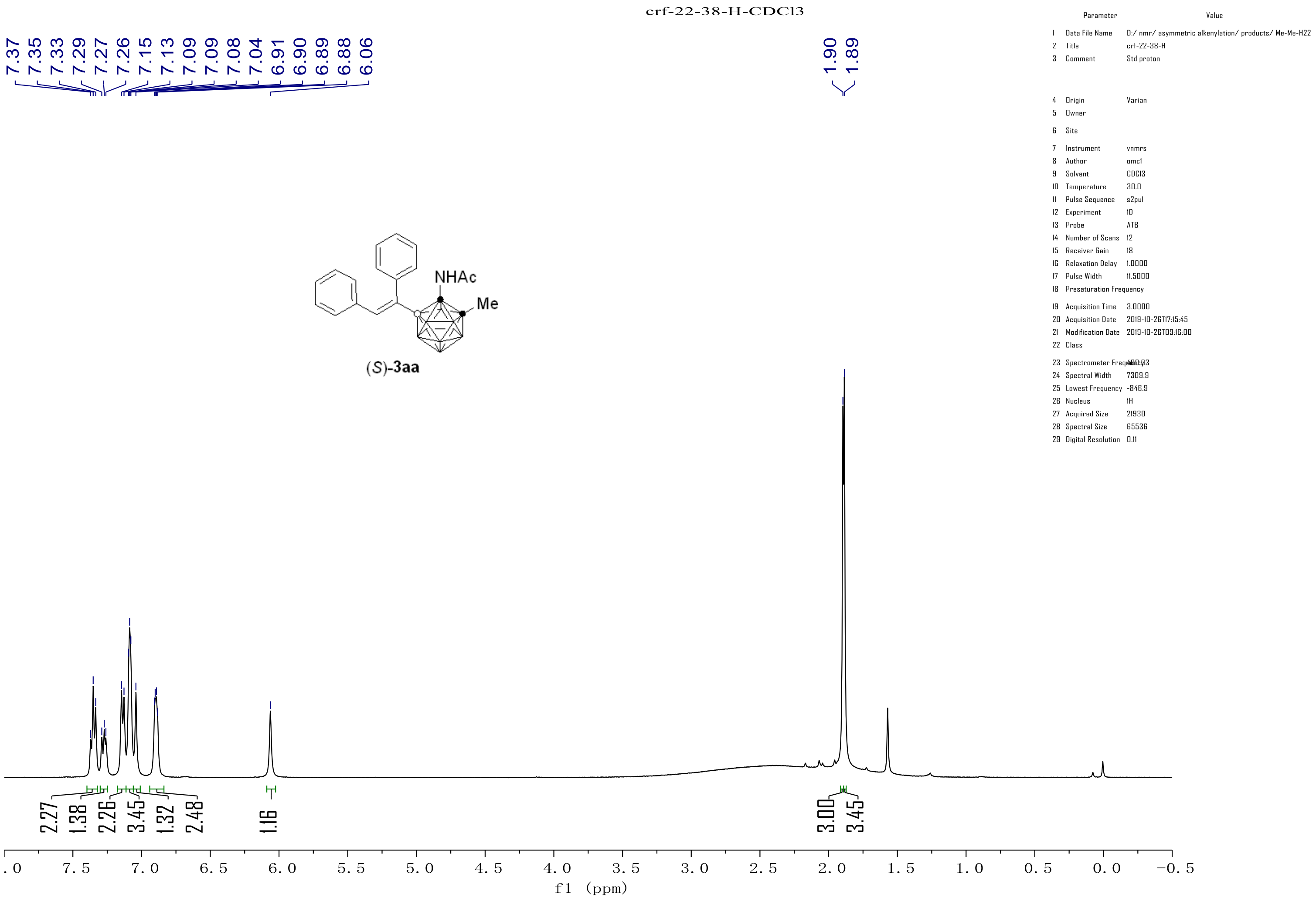

Supplementary Figure 163. $^{13}C\{^1H\}$ NMR of (*S*)-**3aa**.

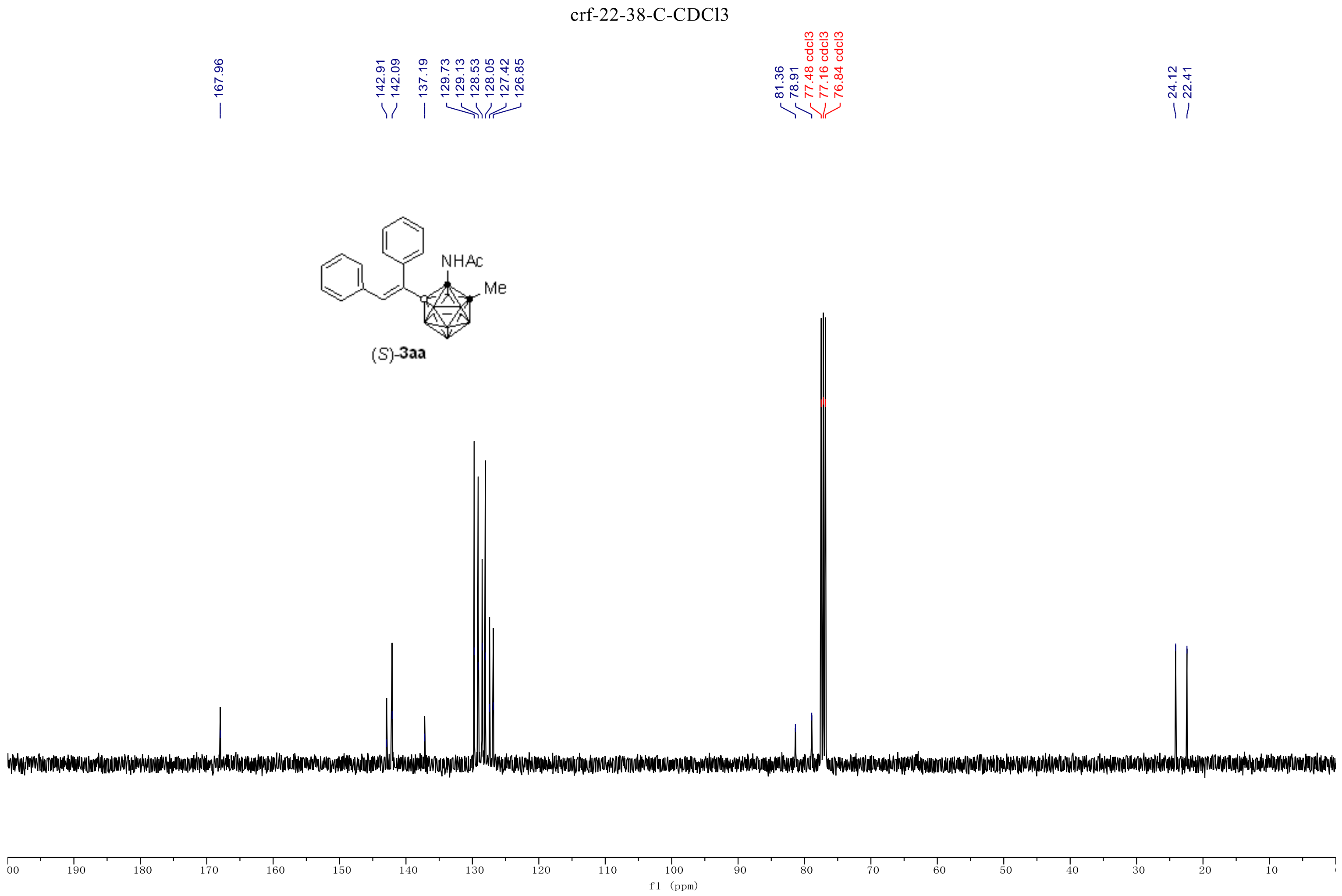

Supplementary Figure 164. $^{11}B\{^{1}H\}$ NMR of (*S*)-**3aa**.

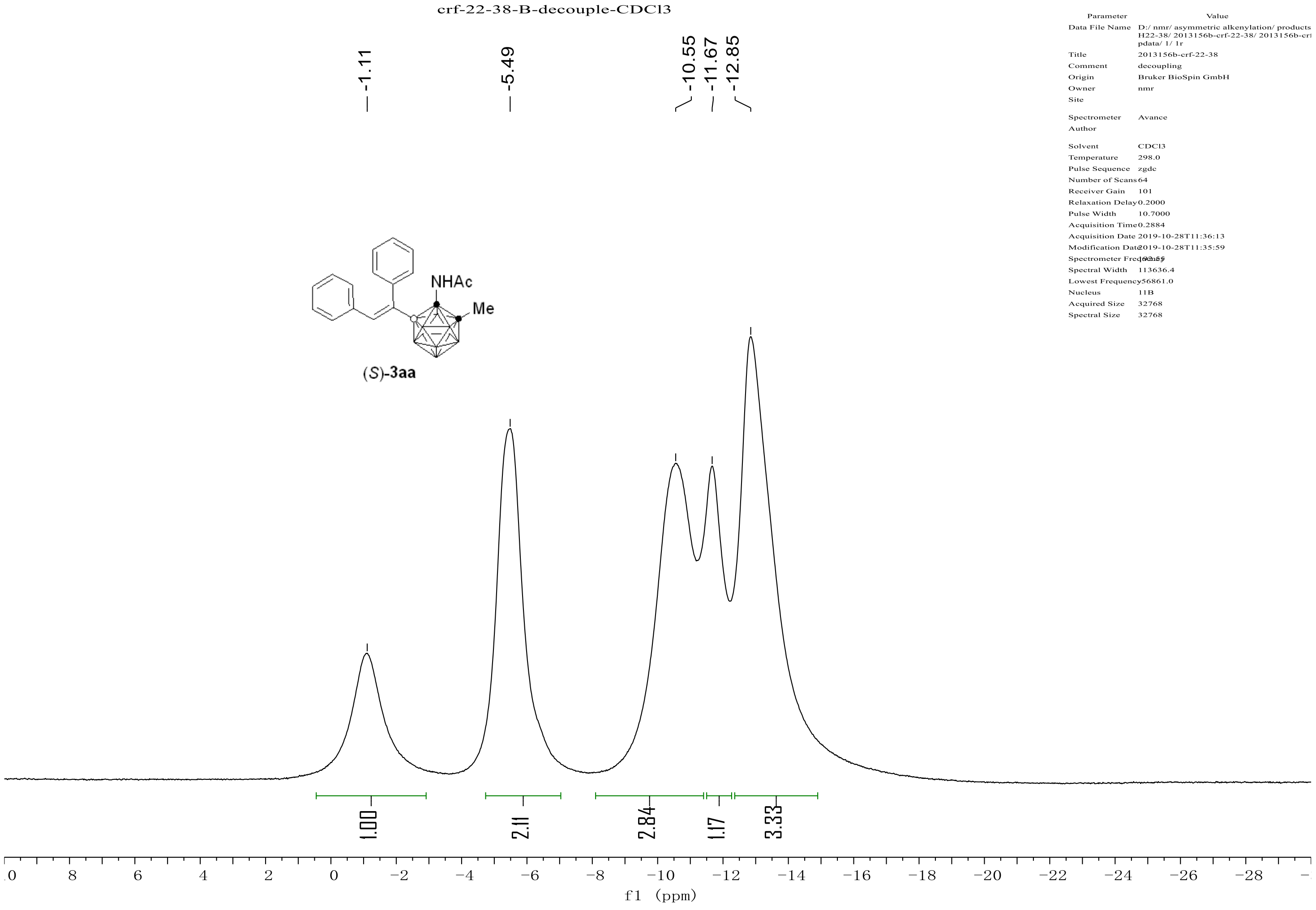

Supplementary Figure 165. ^{11}B NMR of (*S*)-**3aa**.

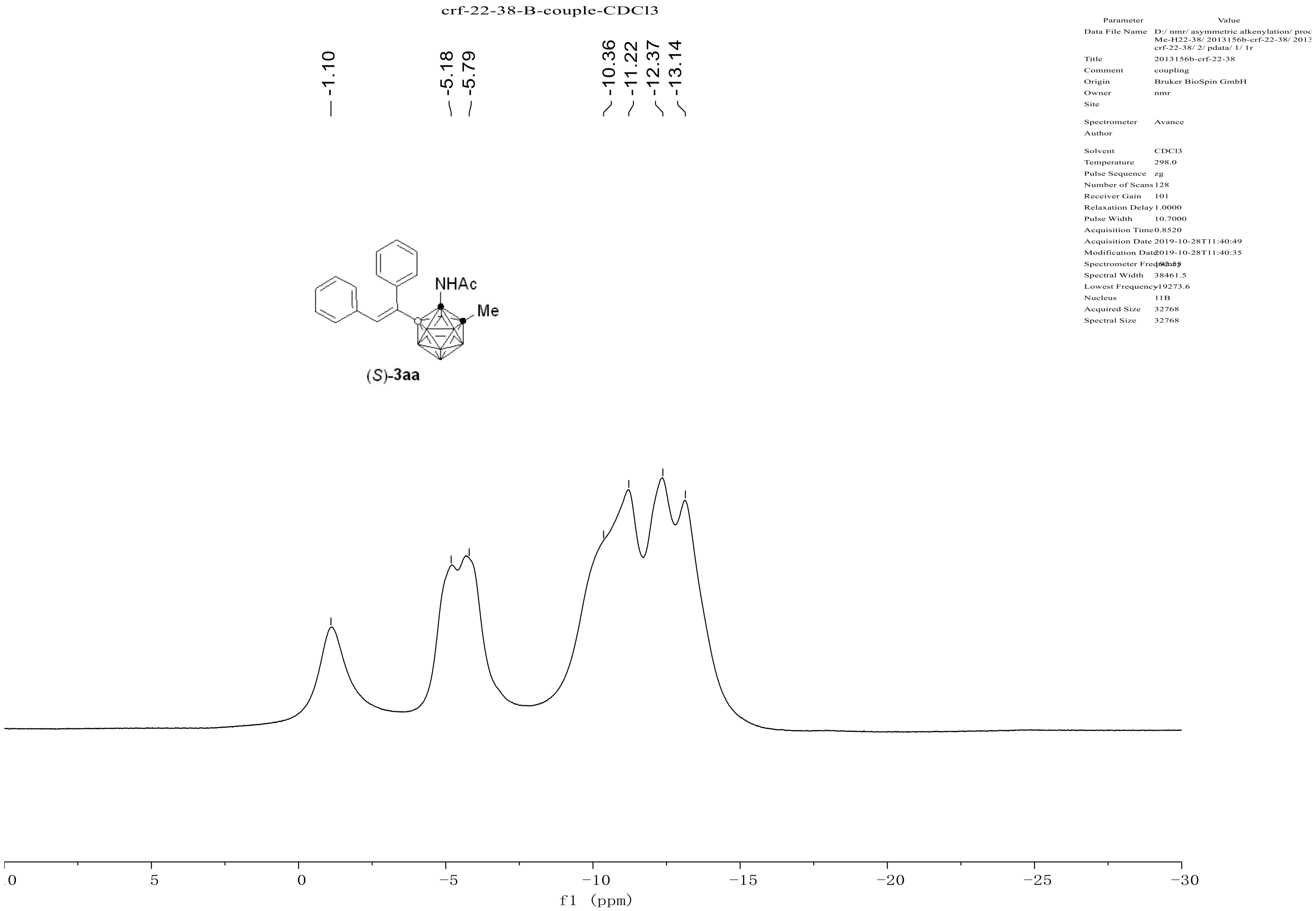

Supplementary Figure 166. ^{1}H NMR of (*S*)-**3da**.

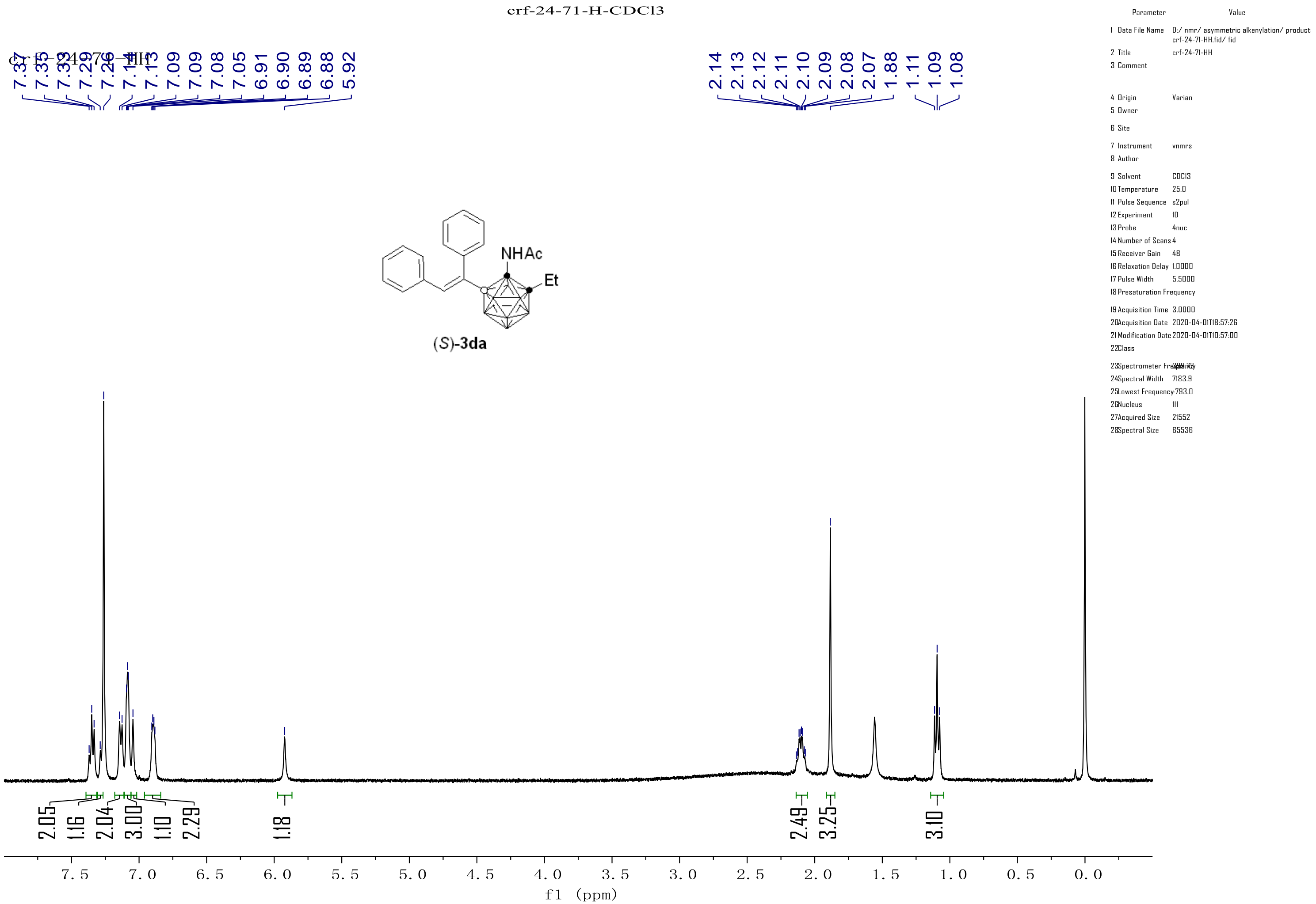

Supplementary Figure 167. $^{13}C\{^{1}H\}$ NMR of (*S*)-**3da**.

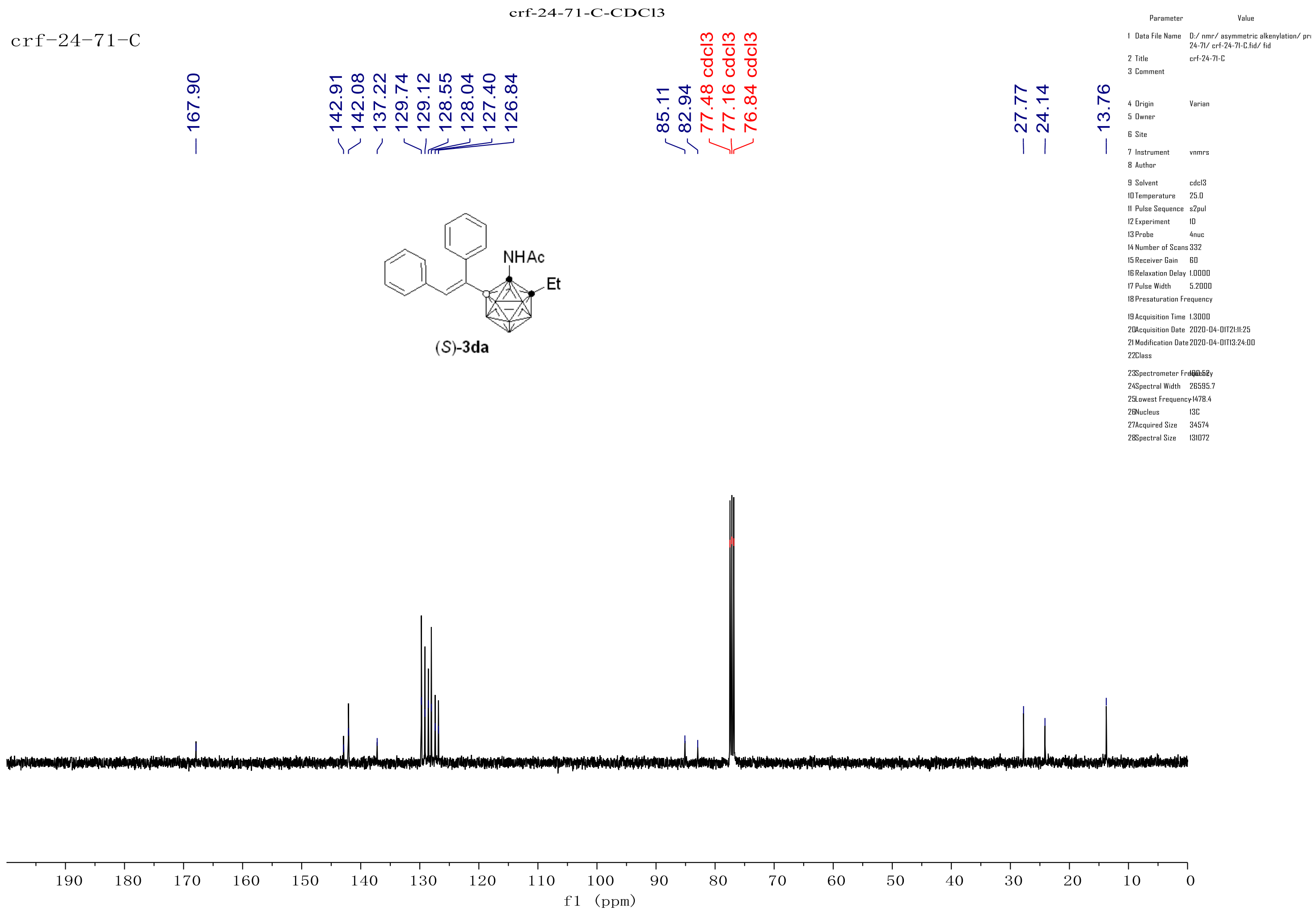

Supplementary Figure 168. $^{11}B\{^{1}H\}$ NMR of (*S*)-**3da**.

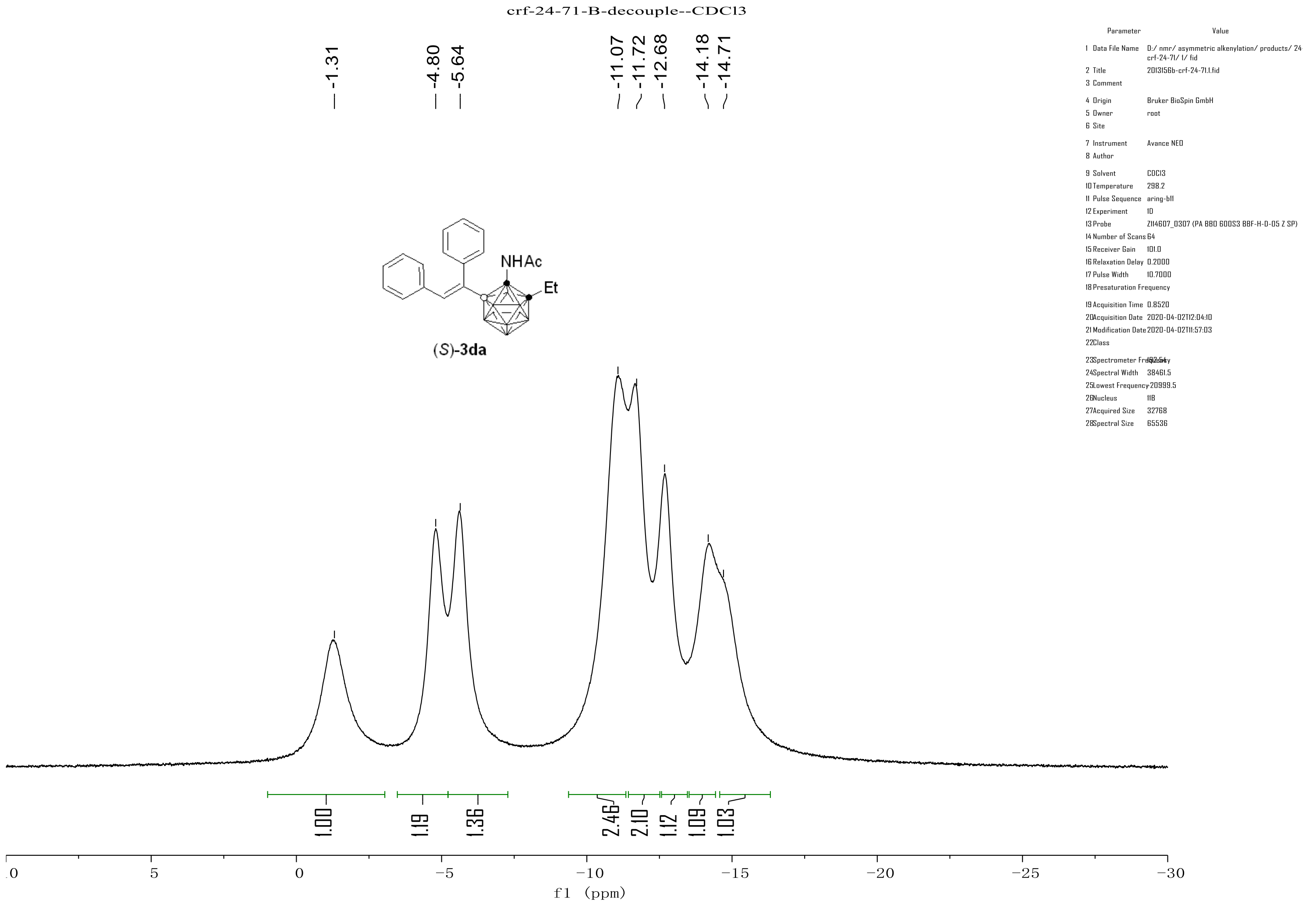

Supplementary Figure 169. ^{11}B NMR of (*S*)-**3da**.

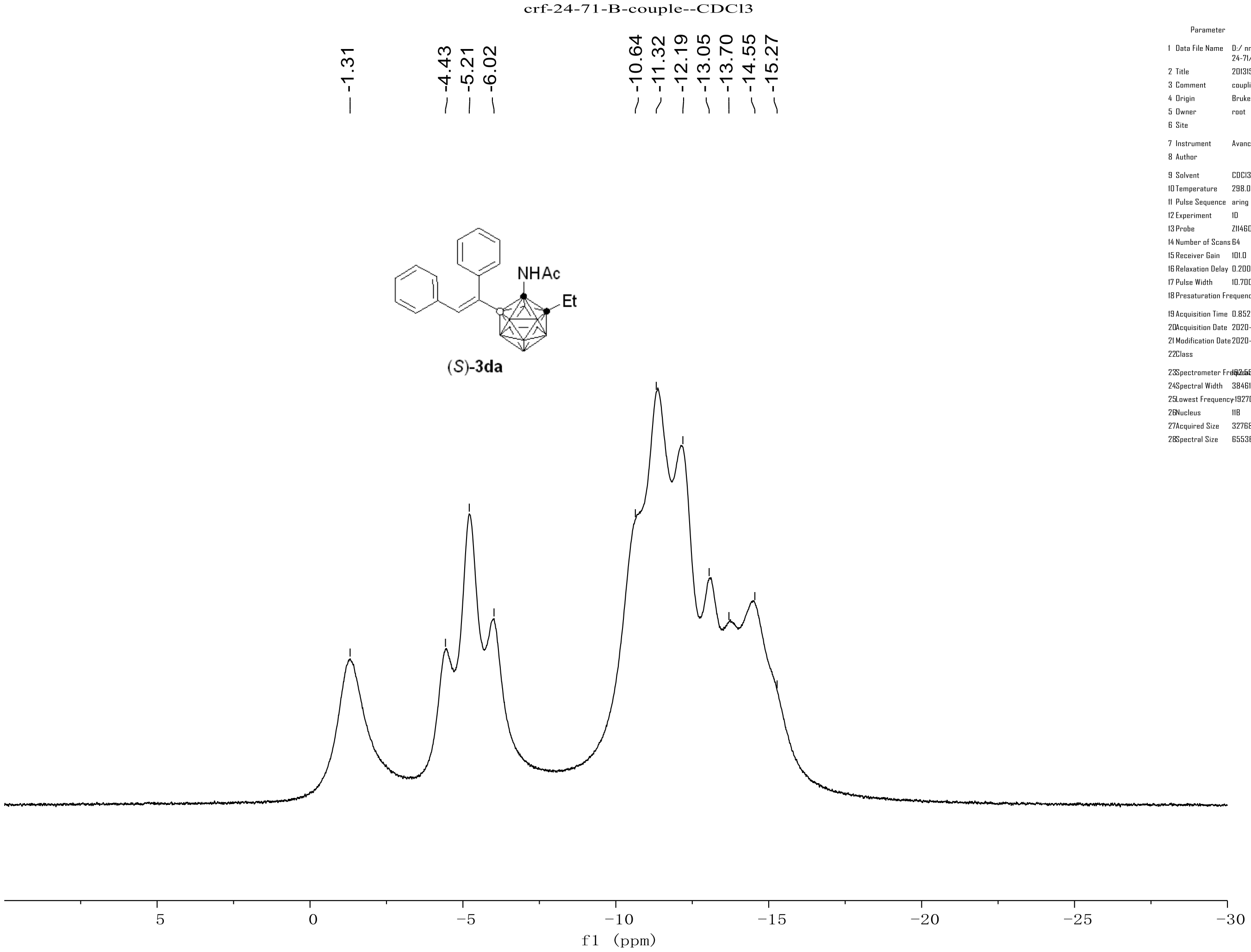

	Parameter	Value
1	Data File Name	D:/ nmr/ asymmetric alkenylation/ p 24-71/ 2013156b-crf-24-71/ 2/ fid
2	Title	2013156b-crf-24-71.2.fid
3	Comment	coupling
4	Origin	Bruker BioSpin GmbH
5	Owner	root
6	Site	
7	Instrument	Avance NEO
8	Author	
9	Solvent	CDCl3
10	Temperature	298.0
11	Pulse Sequence	aring
12	Experiment	1D
13	Probe	Z114607_0307 (PA BBO 600S3 BBF-H-
14	Number of Scans	64
15	Receiver Gain	101.0
16	Relaxation Delay	0.2000
17	Pulse Width	10.7000
18	Presaturation Frequency	
19	Acquisition Time	0.8520
20	Acquisition Date	2020-04-02T12:05:30
21	Modification Date	2020-04-02T11:58:24
22	Class	
23	Spectrometer Frequency	192.55
24	Spectral Width	38461.5
25	Lowest Frequency	-19270.9
26	Nucleus	11B
27	Acquired Size	32768
28	Spectral Size	65536

Supplementary Figure 170. ^{1}H NMR of (*S*)-**3ea**.

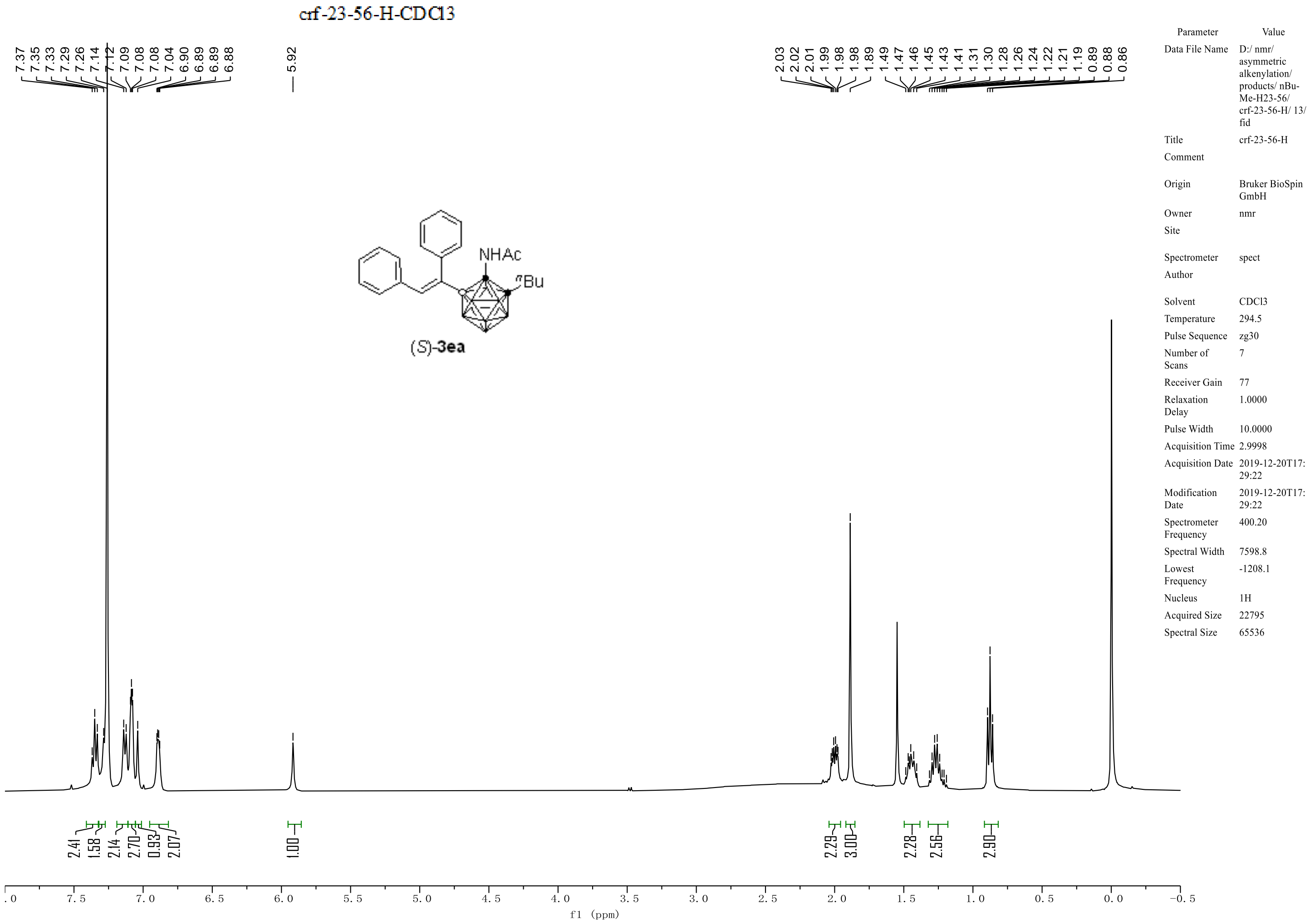

Parameter	Value
Data File Name	D:/ nmr/ asymmetric alkenylation/ products/ nBu-Me-H23-56/ crf-23-56-H/ 13/ fid
Title	crf-23-56-H
Comment	
Origin	Bruker BioSpin GmbH
Owner	nmr
Site	
Spectrometer	spect
Author	
Solvent	CDCl3
Temperature	294.5
Pulse Sequence	zg30
Number of Scans	7
Receiver Gain	77
Relaxation Delay	1.0000
Pulse Width	10.0000
Acquisition Time	2.9998
Acquisition Date	2019-12-20T17:29:22
Modification Date	2019-12-20T17:29:22
Spectrometer Frequency	400.20
Spectral Width	7598.8
Lowest Frequency	-1208.1
Nucleus	1H
Acquired Size	22795
Spectral Size	65536

Supplementary Figure 171. $^{13}C\{^{1}H\}$ NMR of (*S*)-**3ea**.

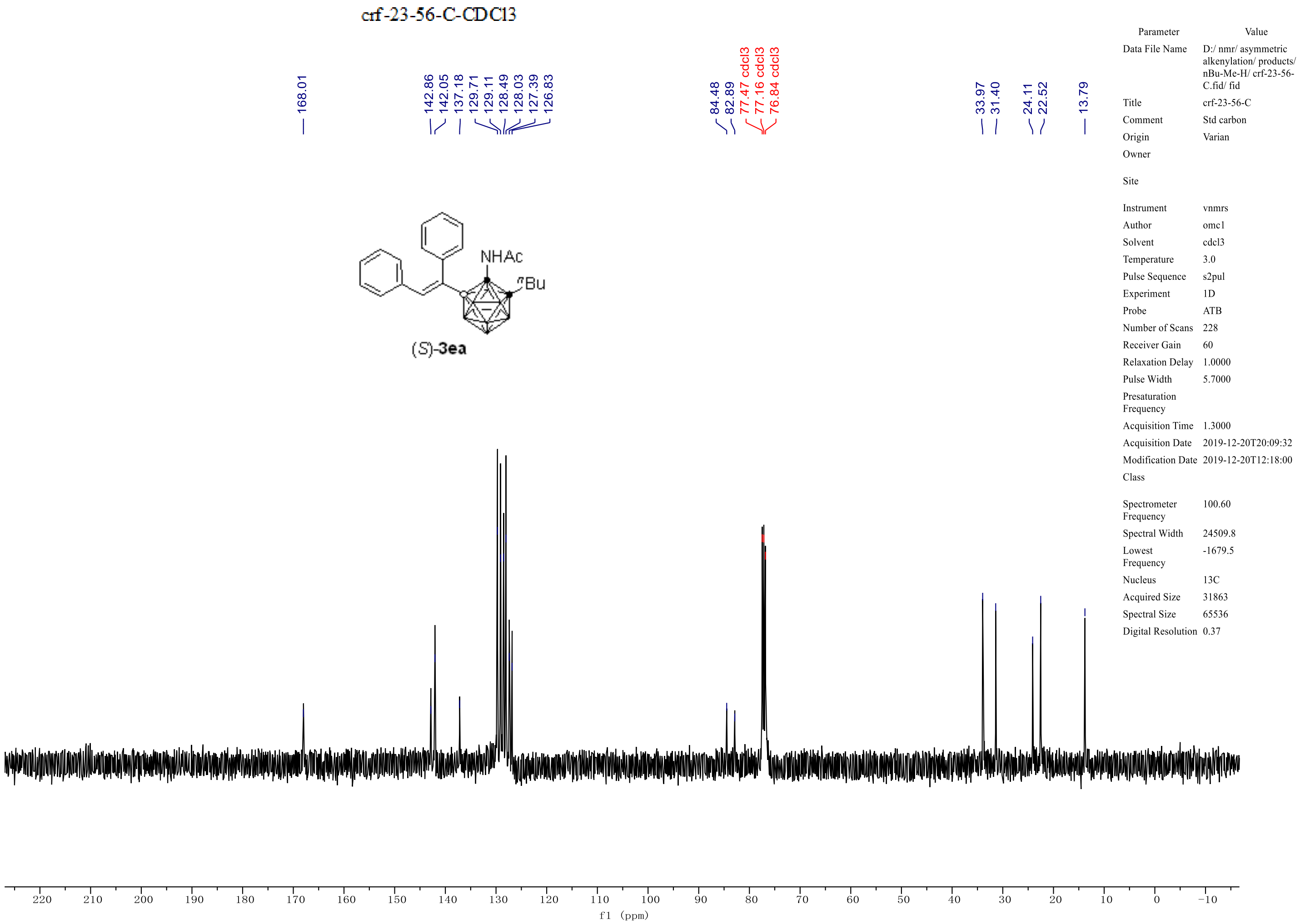

Parameter	Value
Data File Name	D:/ nmr/ asymmetric alkenylation/ products/ nBu-Me-H/ crf-23-56-C.fid/ fid
Title	crf-23-56-C
Comment	Std carbon
Origin	Varian
Owner	
Site	
Instrument	vnmrs
Author	omc1
Solvent	cdcl3
Temperature	3.0
Pulse Sequence	s2pul
Experiment	1D
Probe	ATB
Number of Scans	228
Receiver Gain	60
Relaxation Delay	1.0000
Pulse Width	5.7000
Presaturation Frequency	
Acquisition Time	1.3000
Acquisition Date	2019-12-20T20:09:32
Modification Date	2019-12-20T12:18:00
Class	
Spectrometer Frequency	100.60
Spectral Width	24509.8
Lowest Frequency	-1679.5
Nucleus	13C
Acquired Size	31863
Spectral Size	65536
Digital Resolution	0.37

Supplementary Figure 172. $^{11}B\{^{1}H\}$ NMR of (*S*)-**3ea**.

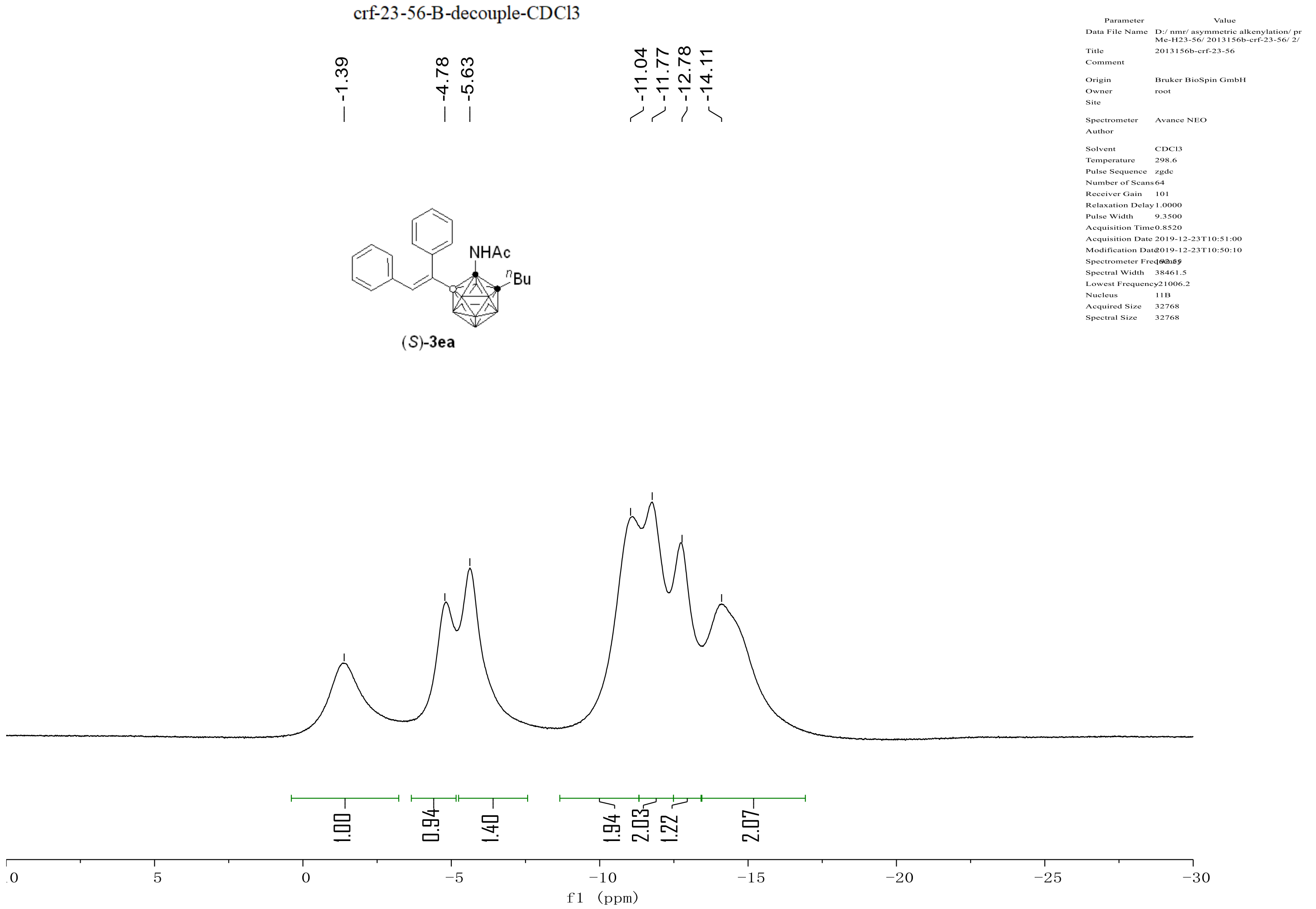

Supplementary Figure 173. ^{11}B NMR of (*S*)-**3ea**.

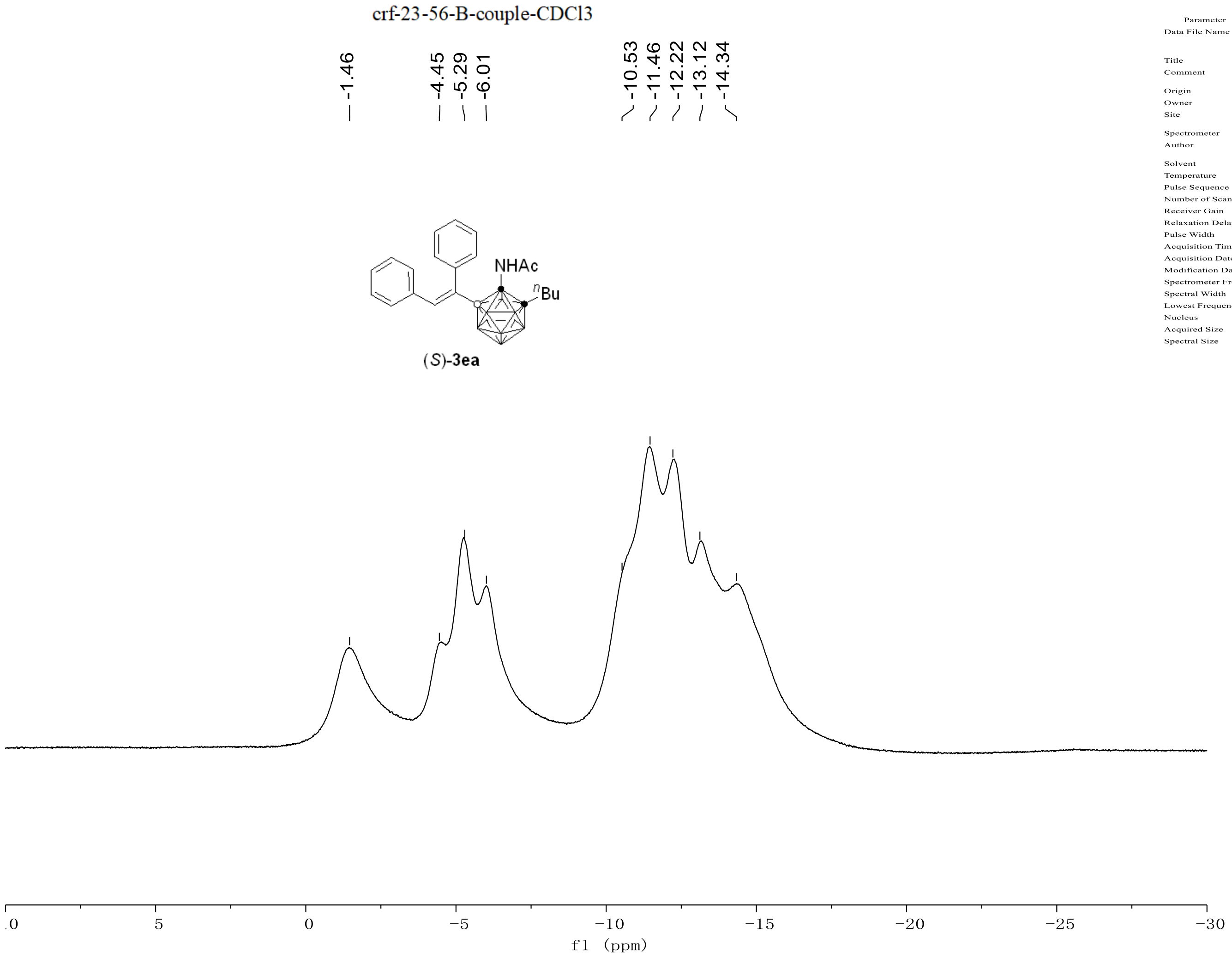

Parameter	Value
Data File Name	D:/ nmr/ asymmetric alkenylatio products/ nBu-Me-H23-56/ 2013 crf-23-56/ 1/ pdata/ 1/ 1r
Title	2013156b-crf-23-56
Comment	
Origin	Bruker BioSpin GmbH
Owner	root
Site	
Spectrometer	Avance NEO
Author	
Solvent	CDCl3
Temperature	297.9
Pulse Sequence	zg
Number of Scans	64
Receiver Gain	101
Relaxation Delay	1.0000
Pulse Width	9.3500
Acquisition Time	0.8520
Acquisition Date	2019-12-23T10:53:43
Modification Date	2019-12-23T10:52:53
Spectrometer Frequency	192.55
Spectral Width	38461.5
Lowest Frequency	-19277.6
Nucleus	11B
Acquired Size	32768
Spectral Size	32768

Supplementary Figure 174. ^{1}H NMR of (*S*)-**3fa**.

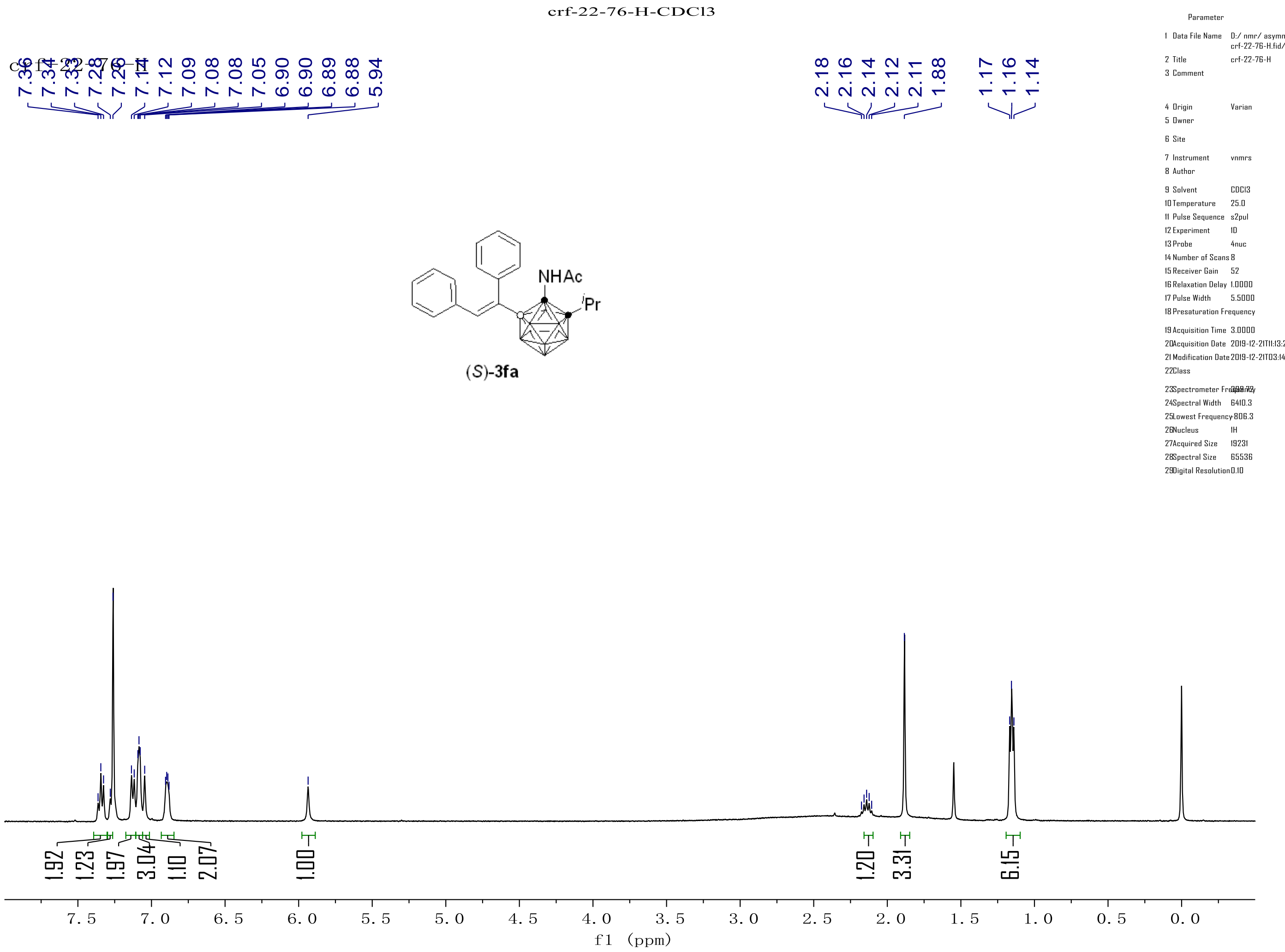

Parameter	Value
1 Data File Name	D:/ nmr/ asymmetric alkenylation/ products/ crf-22-76-H.fid/ fid
2 Title	crf-22-76-H
3 Comment	
4 Origin	Varian
5 Owner	
6 Site	
7 Instrument	vnmrs
8 Author	
9 Solvent	CDCl3
10 Temperature	25.0
11 Pulse Sequence	s2pul
12 Experiment	1D
13 Probe	4nuc
14 Number of Scans	8
15 Receiver Gain	52
16 Relaxation Delay	1.0000
17 Pulse Width	5.5000
18 Presaturation Frequency	
19 Acquisition Time	3.0000
20 Acquisition Date	2019-12-21T11:13:24
21 Modification Date	2019-12-21T03:14:00
22 Class	
23 Spectrometer Frequency	[illegible]
24 Spectral Width	6410.3
25 Lowest Frequency	-806.3
26 Nucleus	1H
27 Acquired Size	19231
28 Spectral Size	65536
29 Digital Resolution	0.10

Supplementary Figure 175. $^{13}C\{^{1}H\}$ NMR of (*S*)-**3fa**.

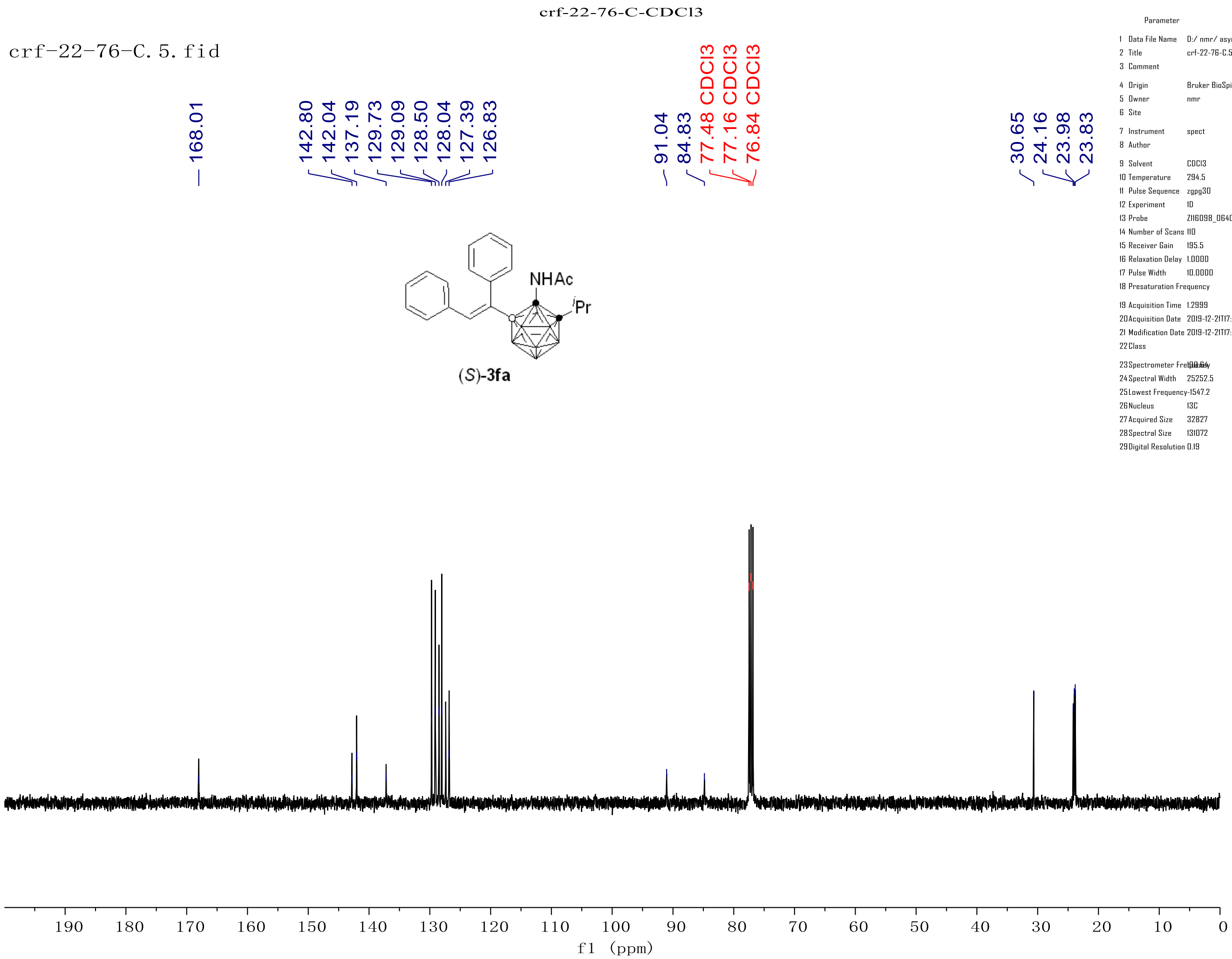

	Parameter	Value
1	Data File Name	D:/ nmr/ asymmetric alkenylation/ products/ iPr-Me
2	Title	crf-22-76-C.5.fid
3	Comment	
4	Origin	Bruker BioSpin GmbH
5	Owner	nmr
6	Site	
7	Instrument	spect
8	Author	
9	Solvent	CDCl3
10	Temperature	294.5
11	Pulse Sequence	zgpg30
12	Experiment	1D
13	Probe	Z116098_0640 (PA BBO 400S1 BBF-H-D-05 Z SP)
14	Number of Scans	110
15	Receiver Gain	195.5
16	Relaxation Delay	1.0000
17	Pulse Width	10.0000
18	Presaturation Frequency	
19	Acquisition Time	1.2999
20	Acquisition Date	2019-12-21T17:29:43
21	Modification Date	2019-12-21T17:29:44
22	Class	
23	Spectrometer Frequency	100.64
24	Spectral Width	25252.5
25	Lowest Frequency	-1547.2
26	Nucleus	13C
27	Acquired Size	32827
28	Spectral Size	131072
29	Digital Resolution	0.19

Supplementary Figure 176. $^{11}B\{^1H\}$ NMR of (*S*)-**3fa**.

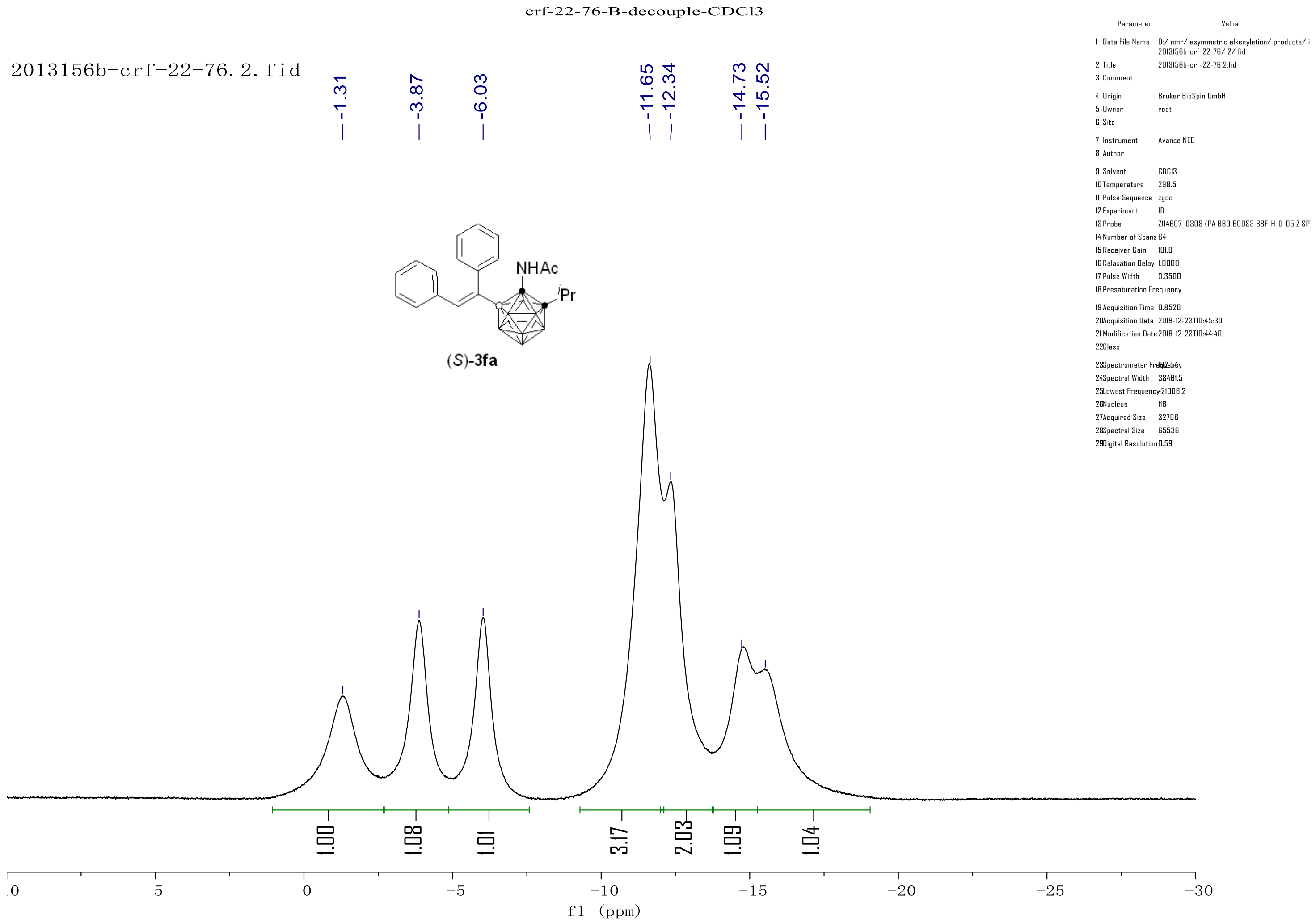

Supplementary Figure 177. ^{11}B NMR of (*S*)-**3fa**.

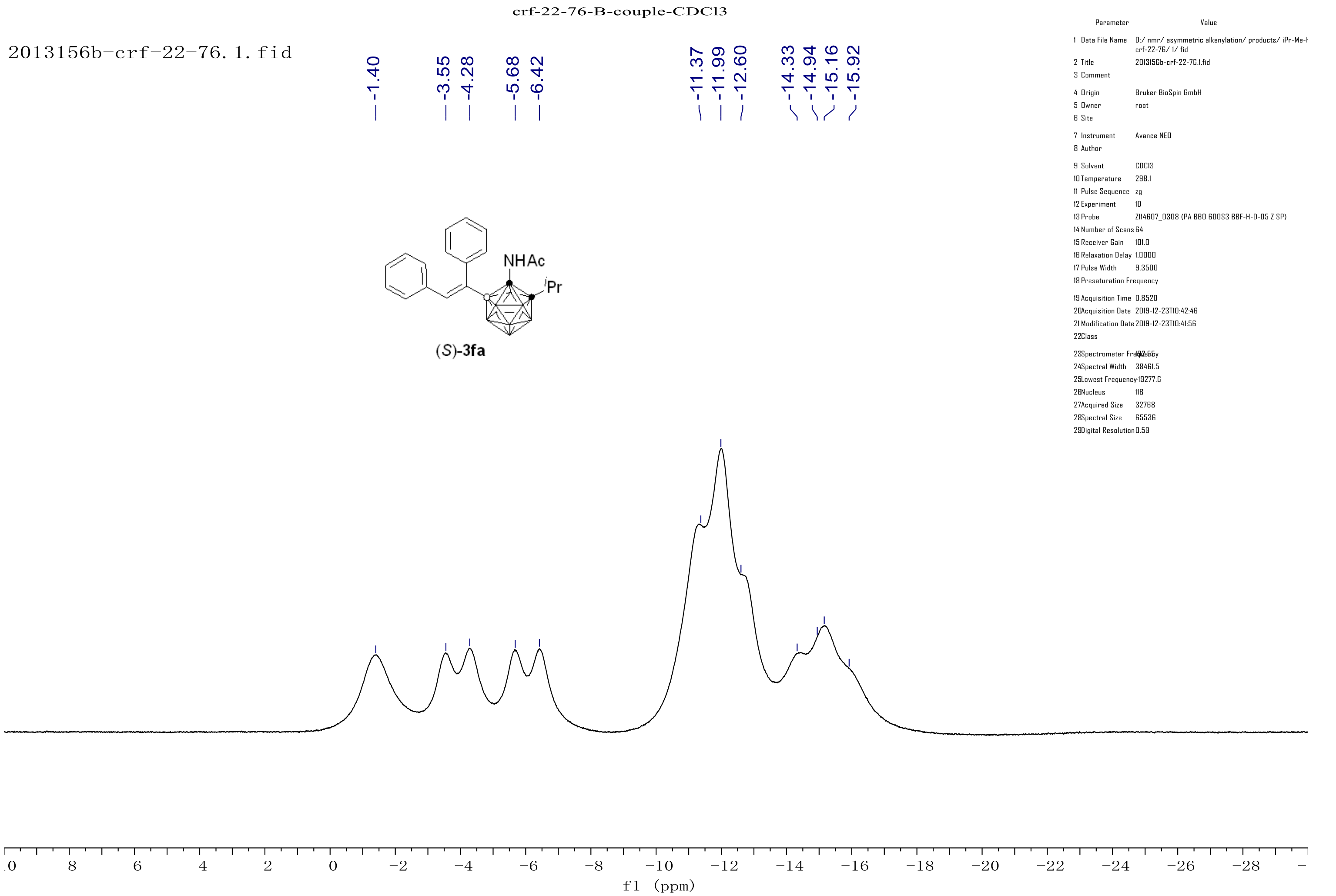

Supplementary Figure 178. ¹H NMR of (*S*)-**3ga**.

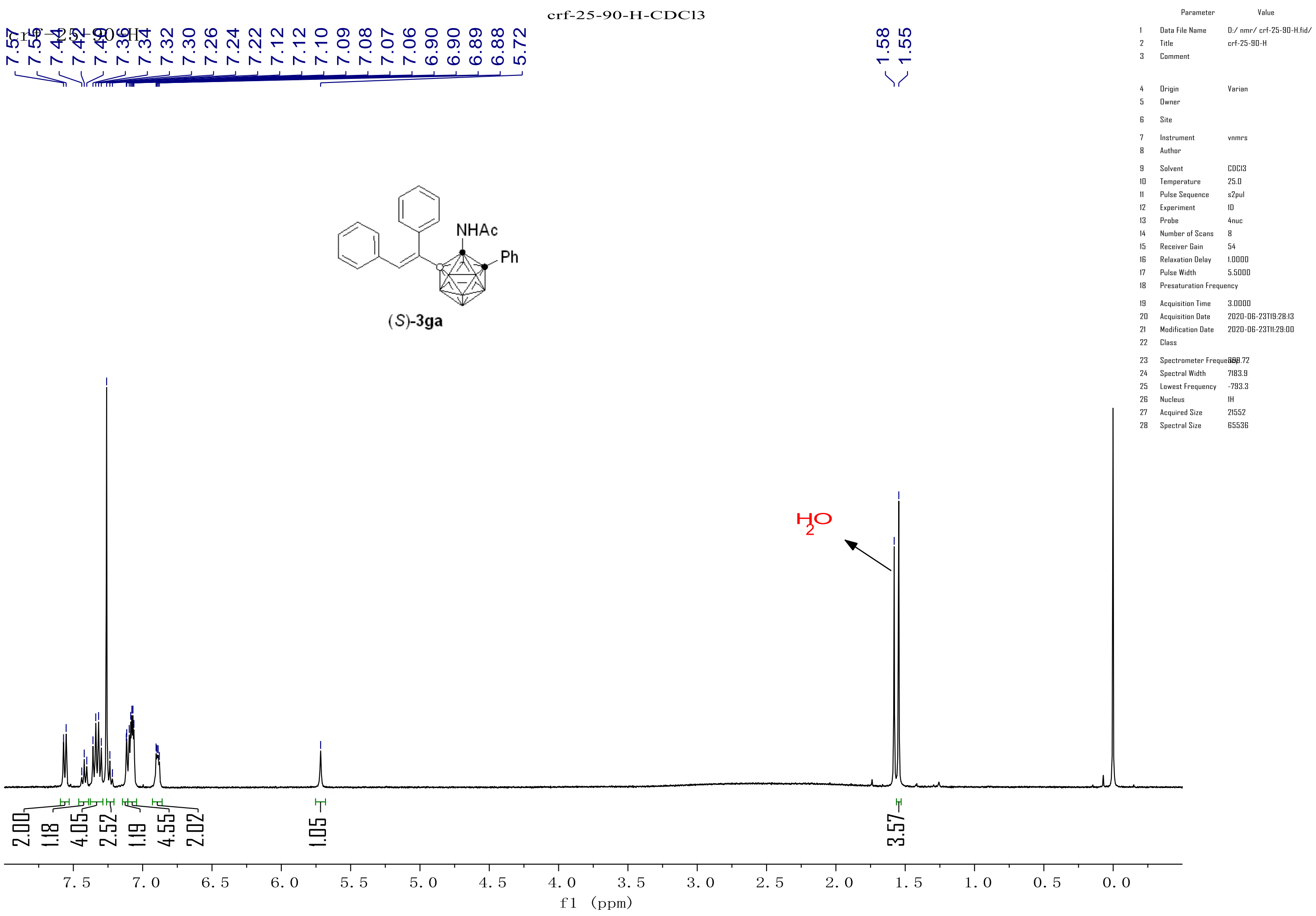

Supplementary Figure 179. $^{13}C\{^{1}H\}$ NMR of (*S*)-**3ga**.

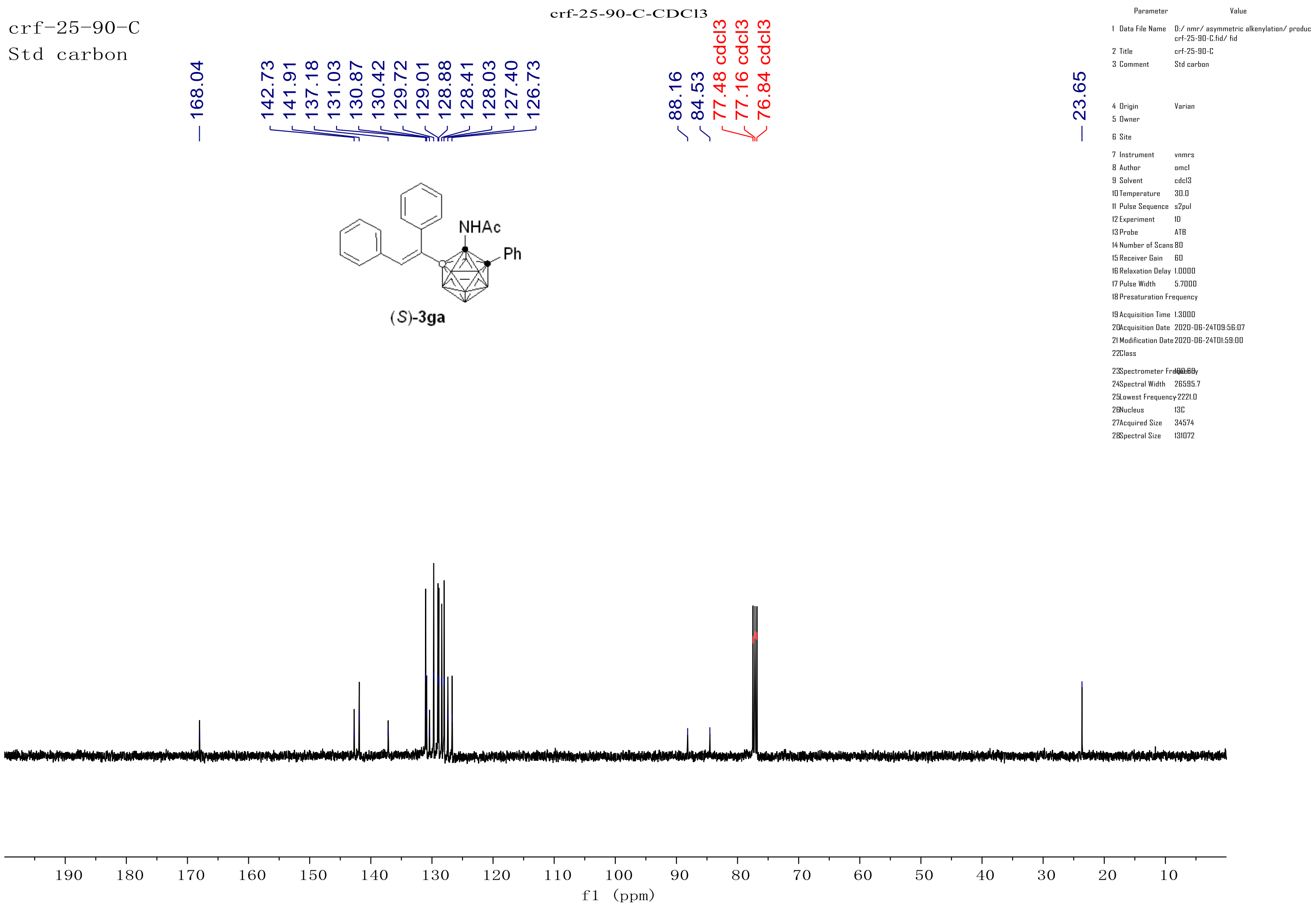

Supplementary Figure 180. $^{11}B\{^{1}H\}$ NMR of (*S*)-**3ga**.

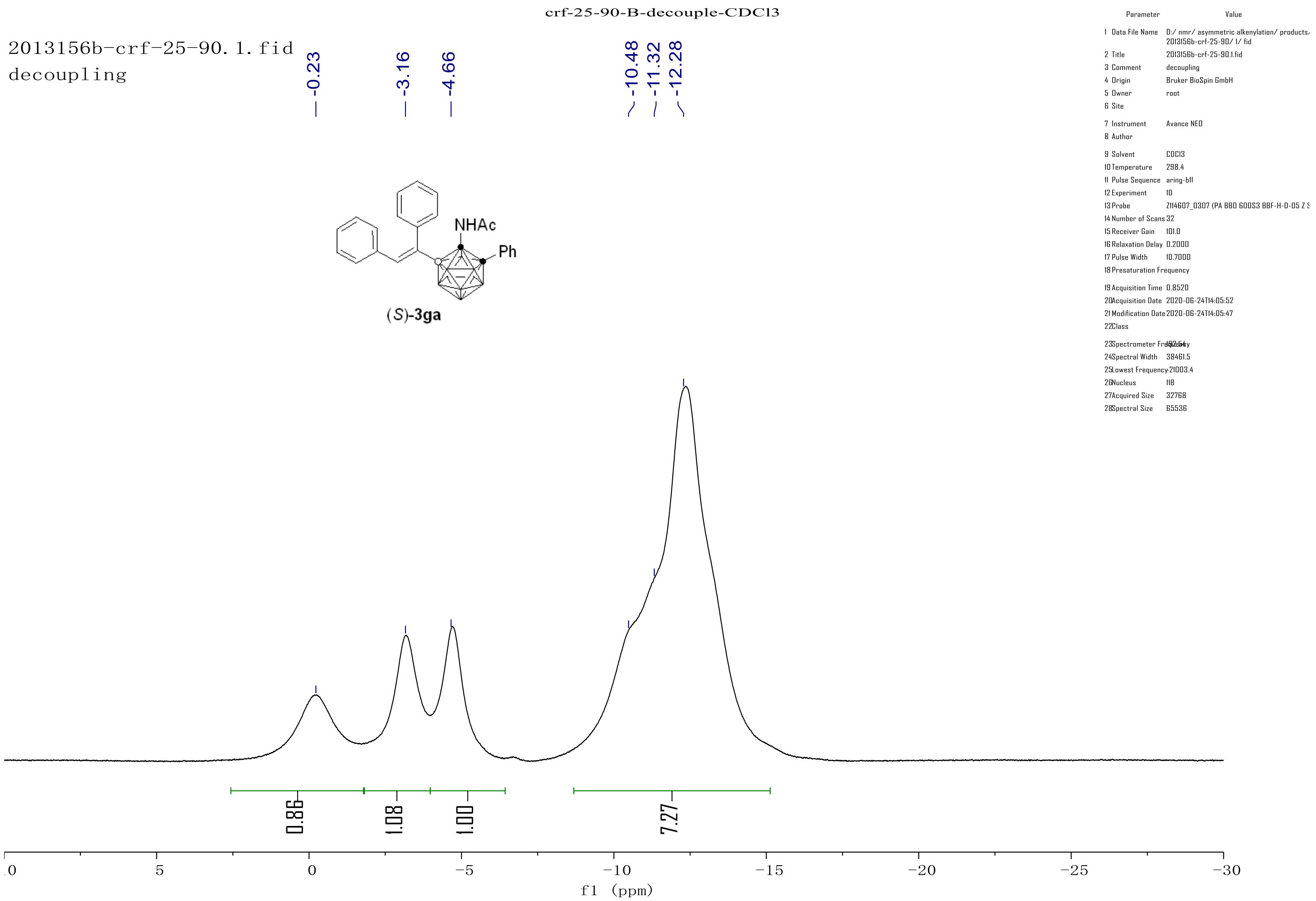

Supplementary Figure 181. ^{11}B NMR of (*S*)-**3ga**.

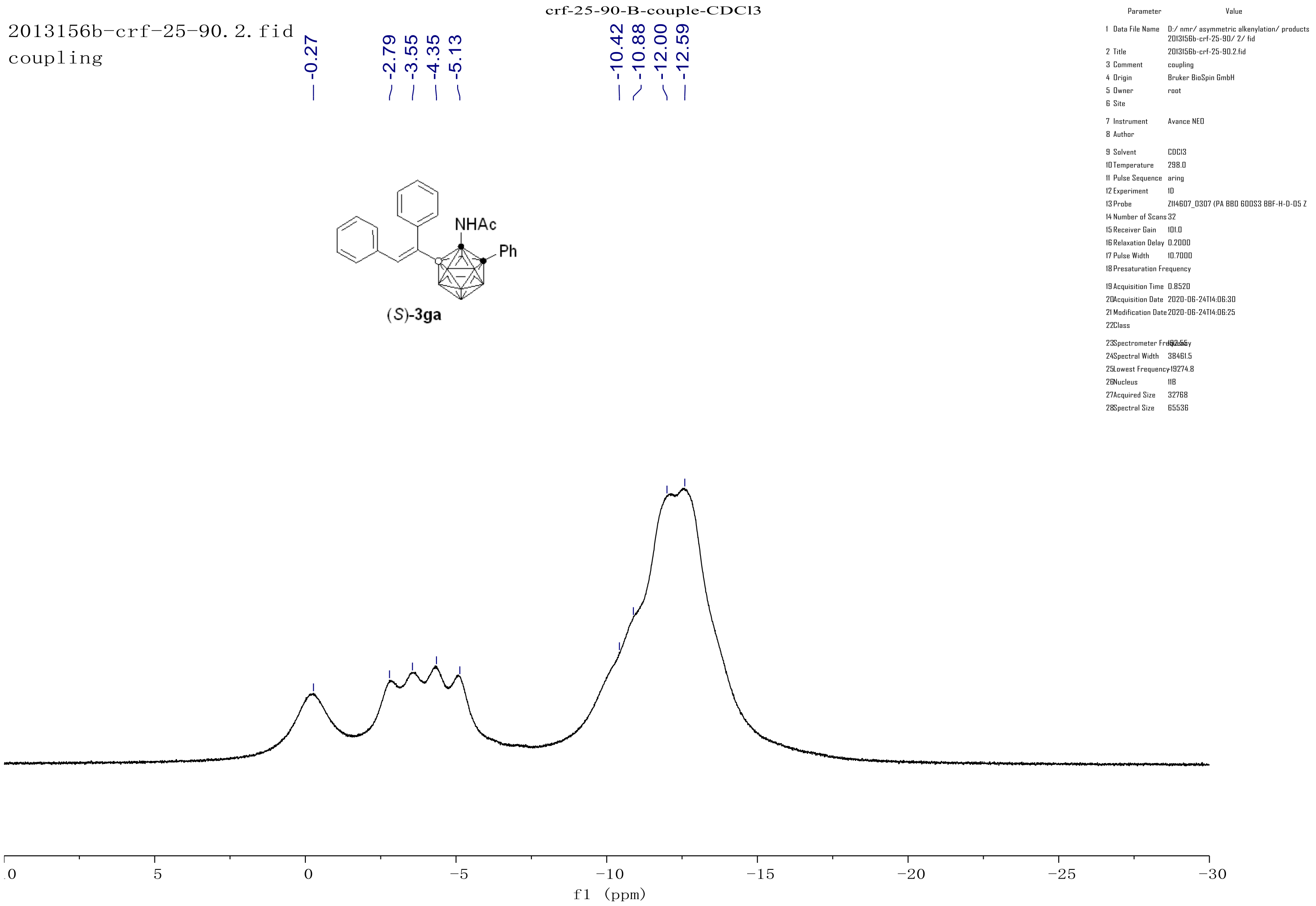

Supplementary Figure 182. ^{1}H NMR of (*S*)-**3ha**.

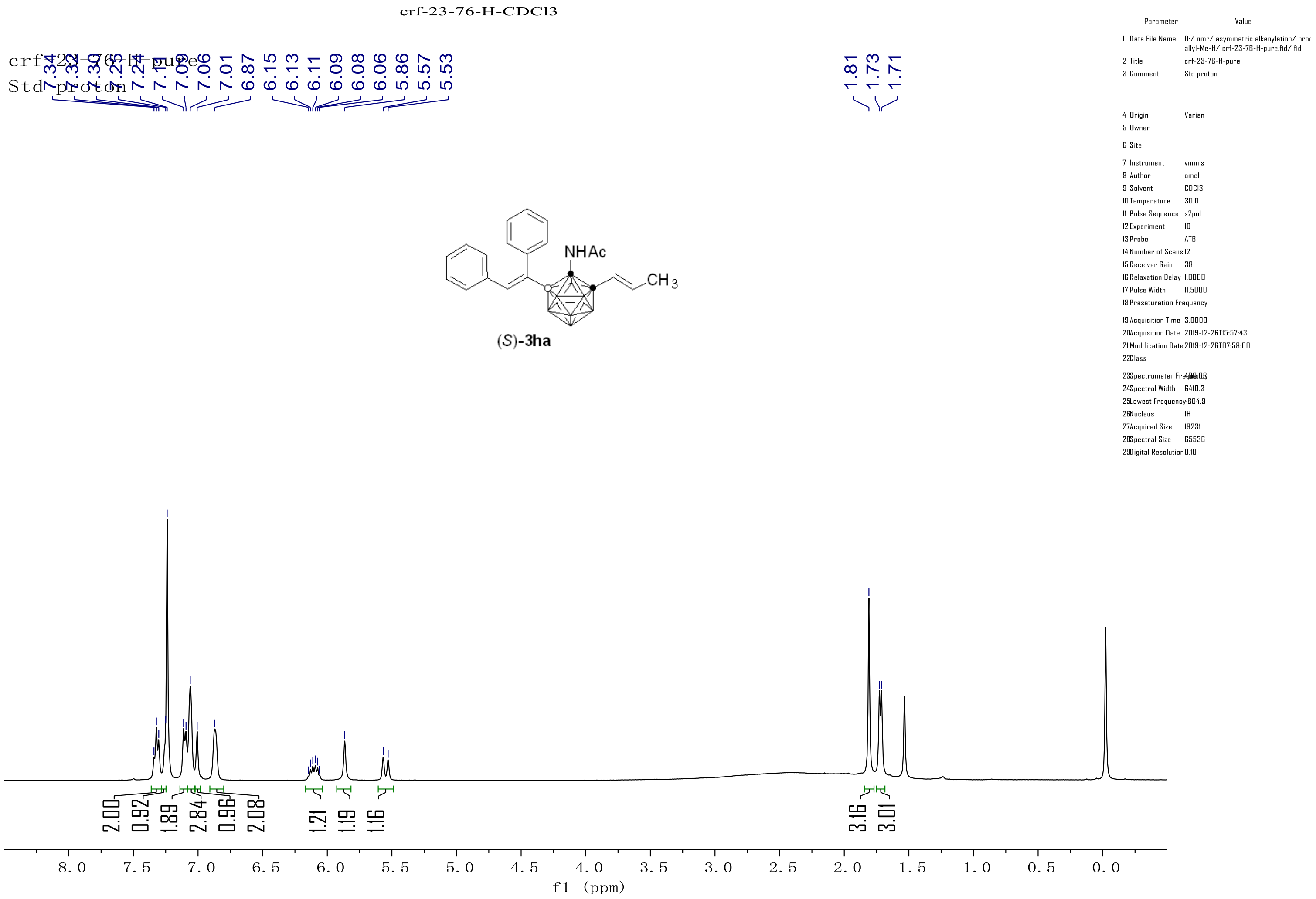

Supplementary Figure 183. $^{13}C\{^{1}H\}$ NMR of (*S*)-**3ha**.

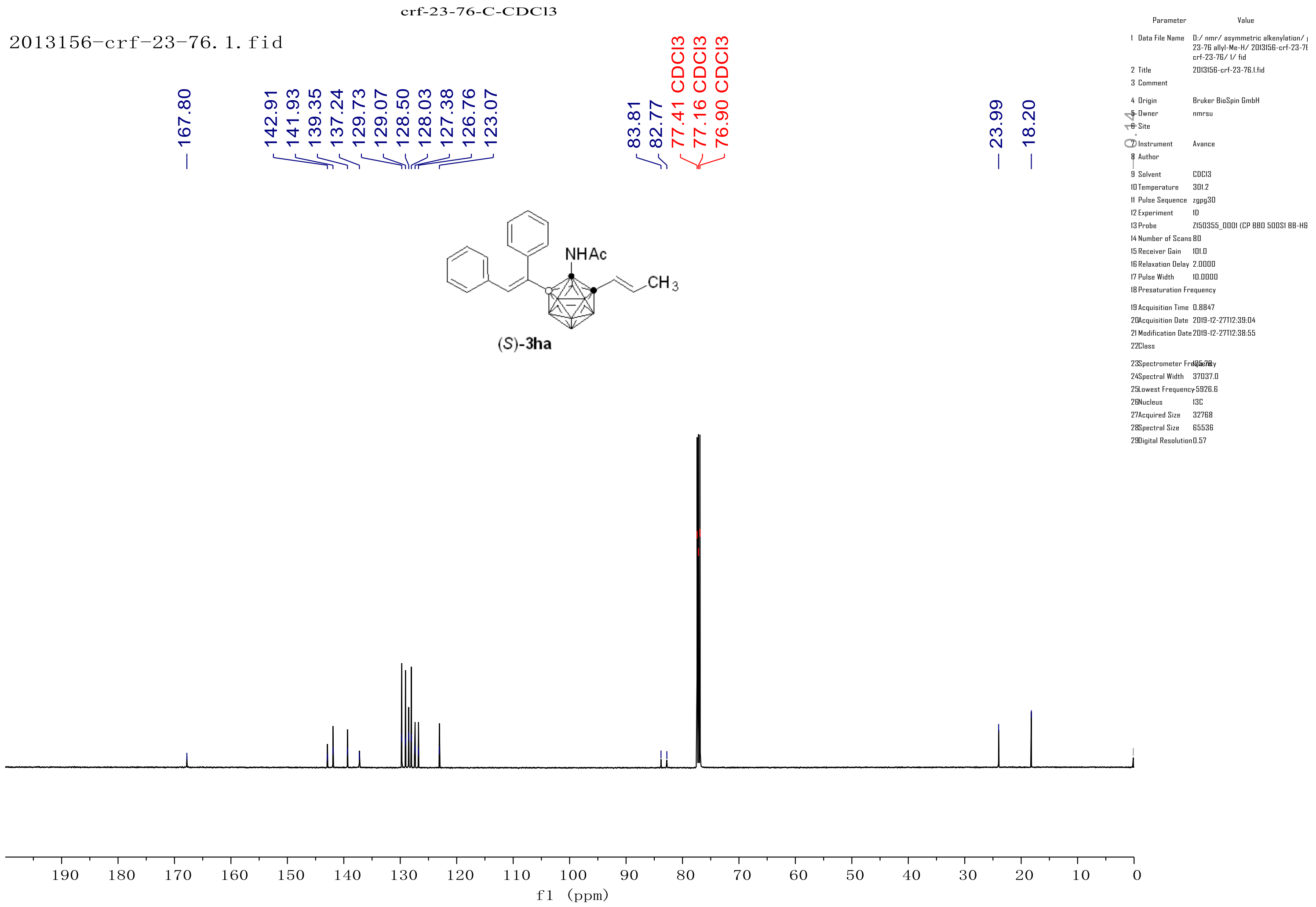

Supplementary Figure 184. $^{11}B\{^{1}H\}$ NMR of (*S*)-**3ha**.

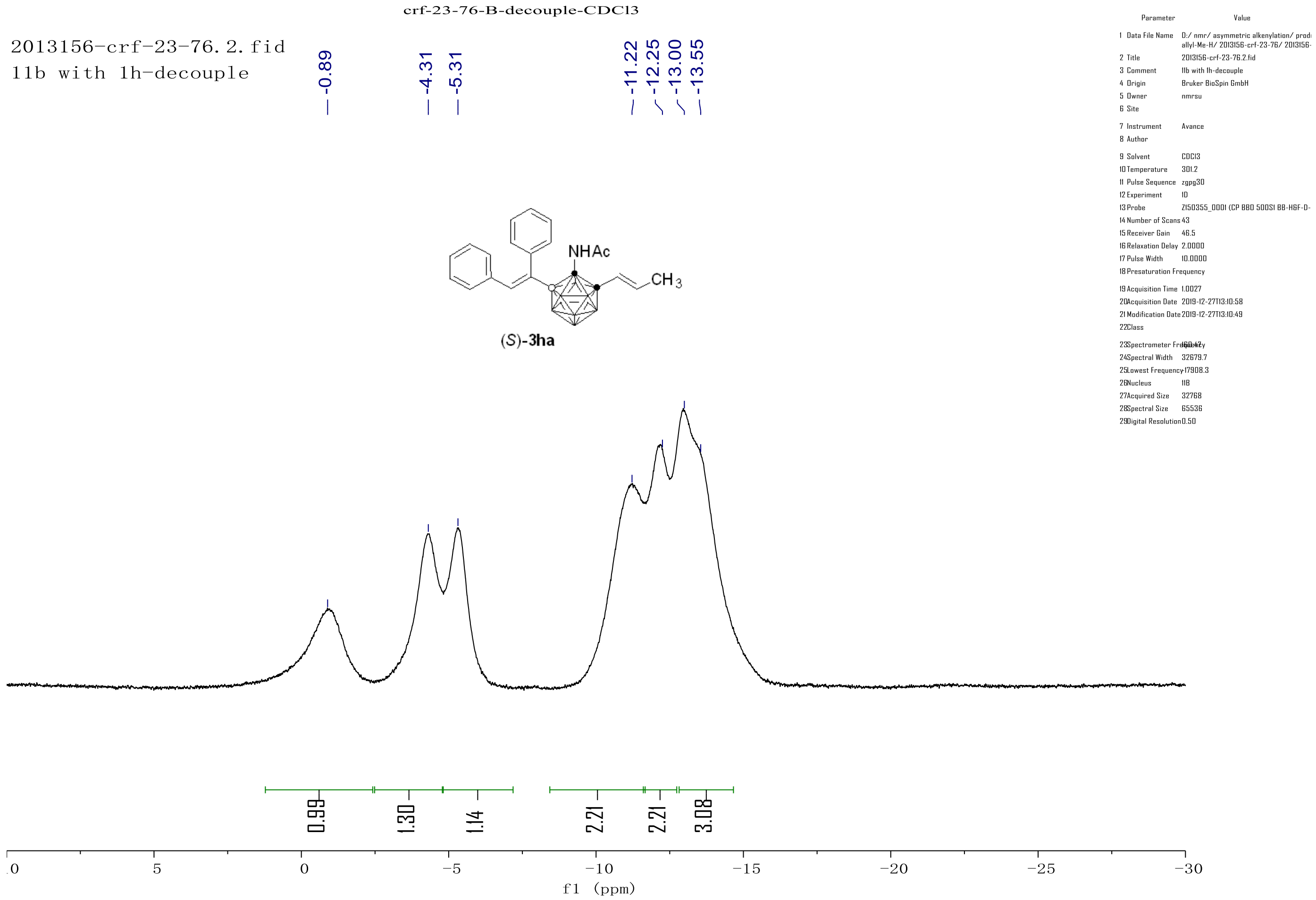

	Parameter	Value
1	Data File Name	D:/ nmr/ asymmetric alkenylation/ prod allyl-Me-H/ 2013156-crf-23-76/ 2013156-
2	Title	2013156-crf-23-76.2.fid
3	Comment	11b with 1h-decouple
4	Origin	Bruker BioSpin GmbH
5	Owner	nmrsu
6	Site	
7	Instrument	Avance
8	Author	
9	Solvent	CDCl3
10	Temperature	301.2
11	Pulse Sequence	zgpg30
12	Experiment	1D
13	Probe	Z150355_0001 (CP BBO 500S1 BB-H&F-D-
14	Number of Scans	43
15	Receiver Gain	46.5
16	Relaxation Delay	2.0000
17	Pulse Width	10.0000
18	Presaturation Frequency	
19	Acquisition Time	1.0027
20	Acquisition Date	2019-12-27T13:10:58
21	Modification Date	2019-12-27T13:10:49
22	Class	
23	Spectrometer Frequency	160.47
24	Spectral Width	32679.7
25	Lowest Frequency	-17908.3
26	Nucleus	11B
27	Acquired Size	32768
28	Spectral Size	65536
29	Digital Resolution	0.50

Supplementary Figure 185. ^{11}B NMR of (*S*)-**3ha**.

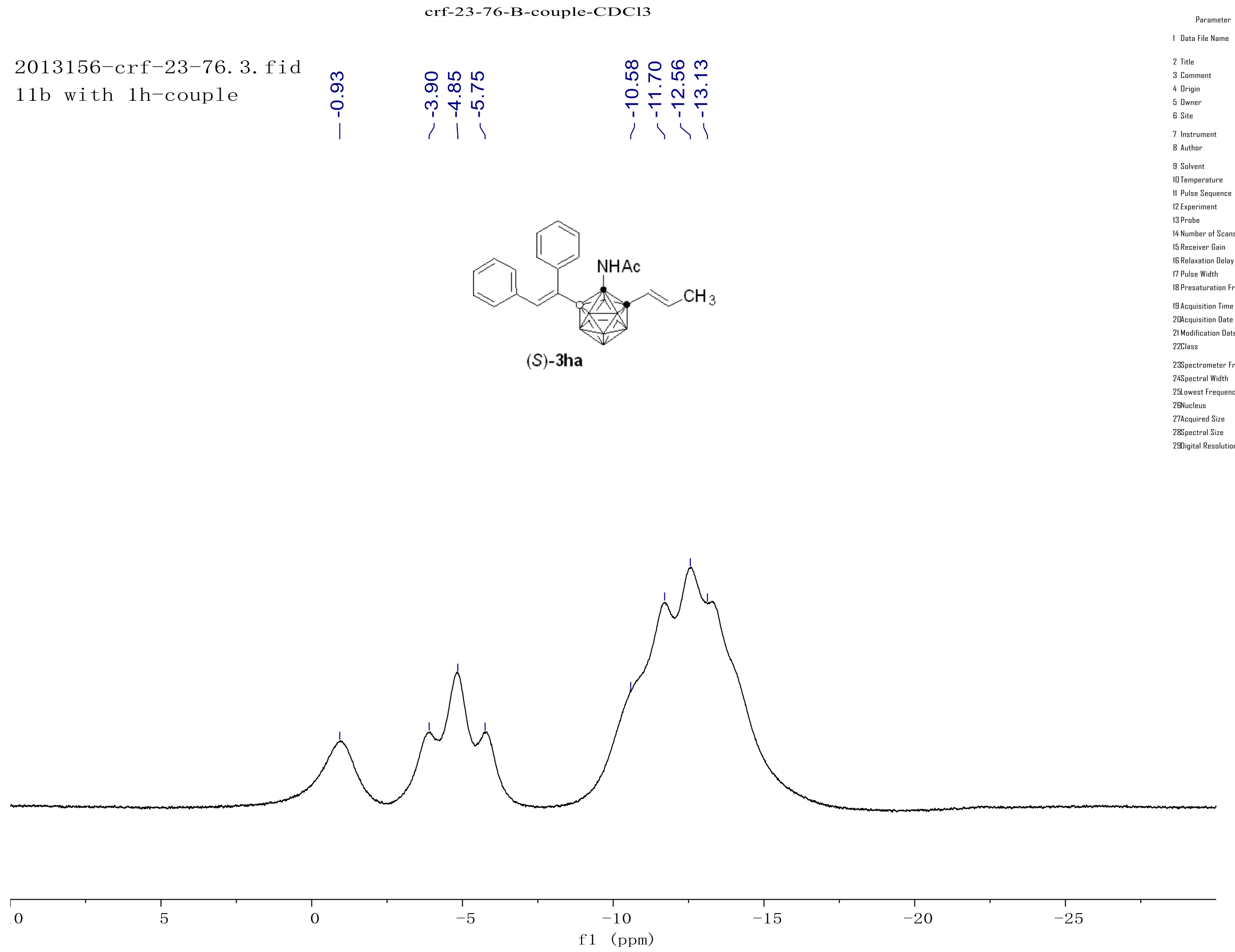

	Parameter	Value
1	Data File Name	D:/ nmr/ asymmetric alkenylation/ prod allyl-Me-H/ 2013156-crf-23-76/ 2013156-
2	Title	2013156-crf-23-76.2.fid
3	Comment	11b with 1h-decouple
4	Origin	Bruker BioSpin GmbH
5	Owner	nmrsu
6	Site	
7	Instrument	Avance
8	Author	
9	Solvent	CDCl3
10	Temperature	301.2
11	Pulse Sequence	zgpg30
12	Experiment	1D
13	Probe	Z150355_0001 (CP BBO 500S1 BB-H&F-D-
14	Number of Scans	43
15	Receiver Gain	46.5
16	Relaxation Delay	2.0000
17	Pulse Width	10.0000
18	Presaturation Frequency	
19	Acquisition Time	1.0027
20	Acquisition Date	2019-12-27T13:10:58
21	Modification Date	2019-12-27T13:10:49
22	Class	
23	Spectrometer Frequency	160.47
24	Spectral Width	32679.7
25	Lowest Frequency	-17908.3
26	Nucleus	11B
27	Acquired Size	32768
28	Spectral Size	65536
29	Digital Resolution	0.50

Supplementary Figure 186. ^{1}H NMR of (*S*)-**3ia**.

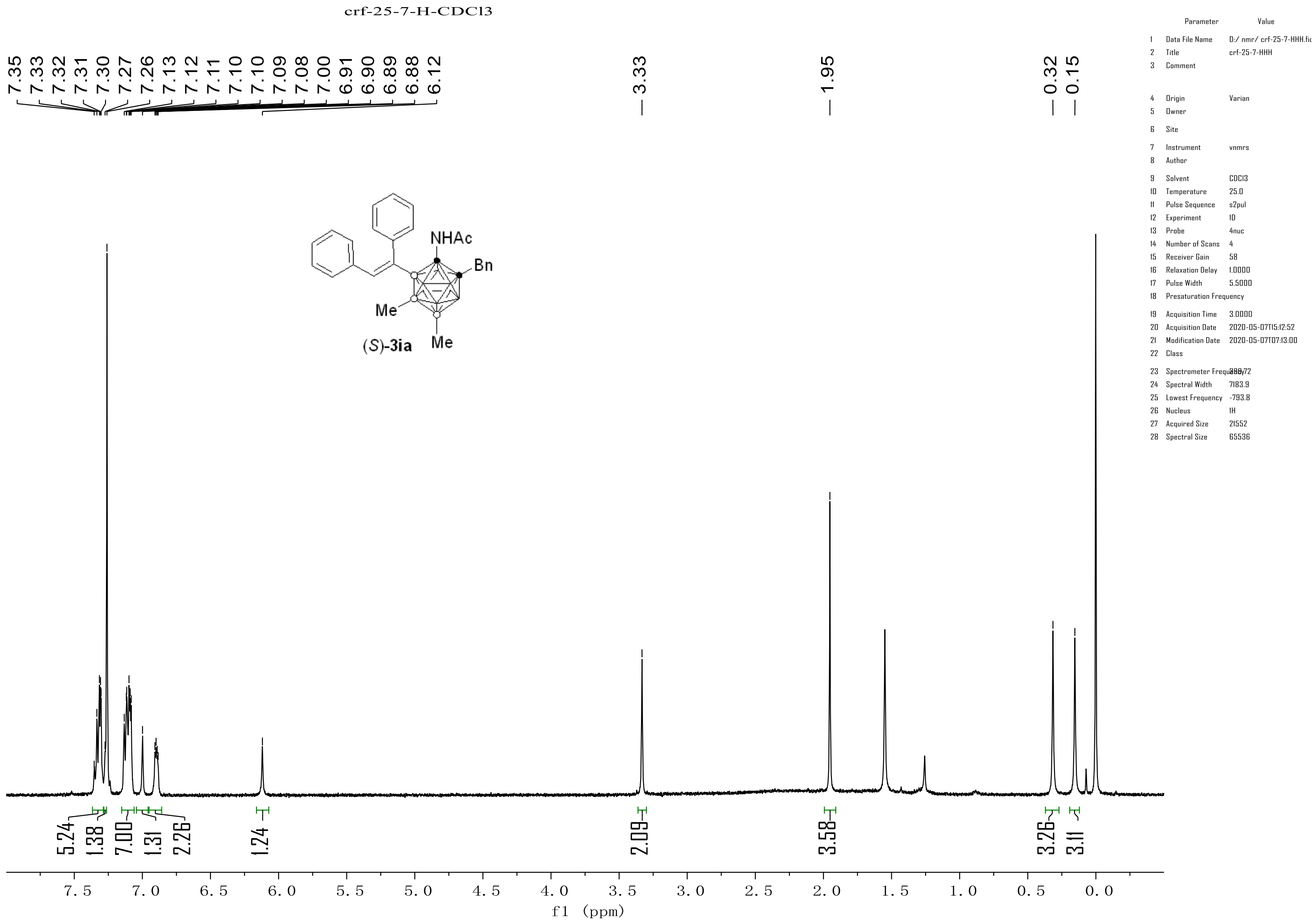

Supplementary Figure 187. $^{13}C\{^{1}H\}$ NMR of (*S*)-**3ia**.

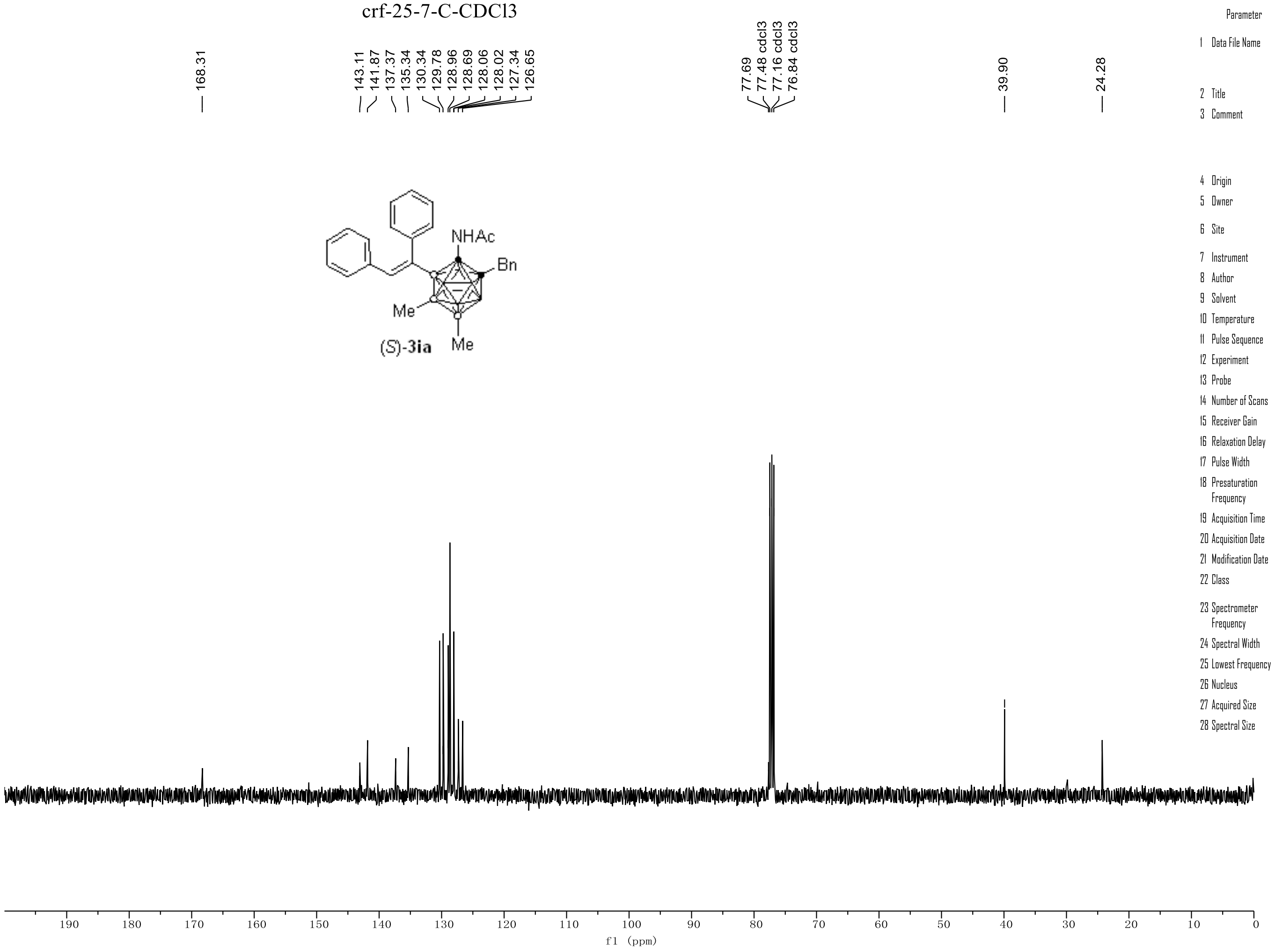

	Parameter	Value
1	Data File Name	D:/ nmr/ asymmetric alkenylation/ products/ 25-7/ crf-25-7-C.fid/ fid
2	Title	crf-25-7-C
3	Comment	Std carbon
4	Origin	Varian
5	Owner	
6	Site	
7	Instrument	vnmrs
8	Author	omcl
9	Solvent	cdcl3
10	Temperature	30.0
11	Pulse Sequence	s2pul
12	Experiment	1D
13	Probe	ATB
14	Number of Scans	248
15	Receiver Gain	60
16	Relaxation Delay	1.0000
17	Pulse Width	5.7000
18	Presaturation Frequency	
19	Acquisition Time	1.3000
20	Acquisition Date	2020-05-07T19:16:31
21	Modification Date	2020-05-07T11:26:00
22	Class	
23	Spectrometer Frequency	100.60
24	Spectral Width	26595.7
25	Lowest Frequency	-2217.1
26	Nucleus	13C
27	Acquired Size	34574
28	Spectral Size	131072

Supplementary Figure 188. $^{11}B\{^{1}H\}$ NMR of (*S*)-**3ia**.

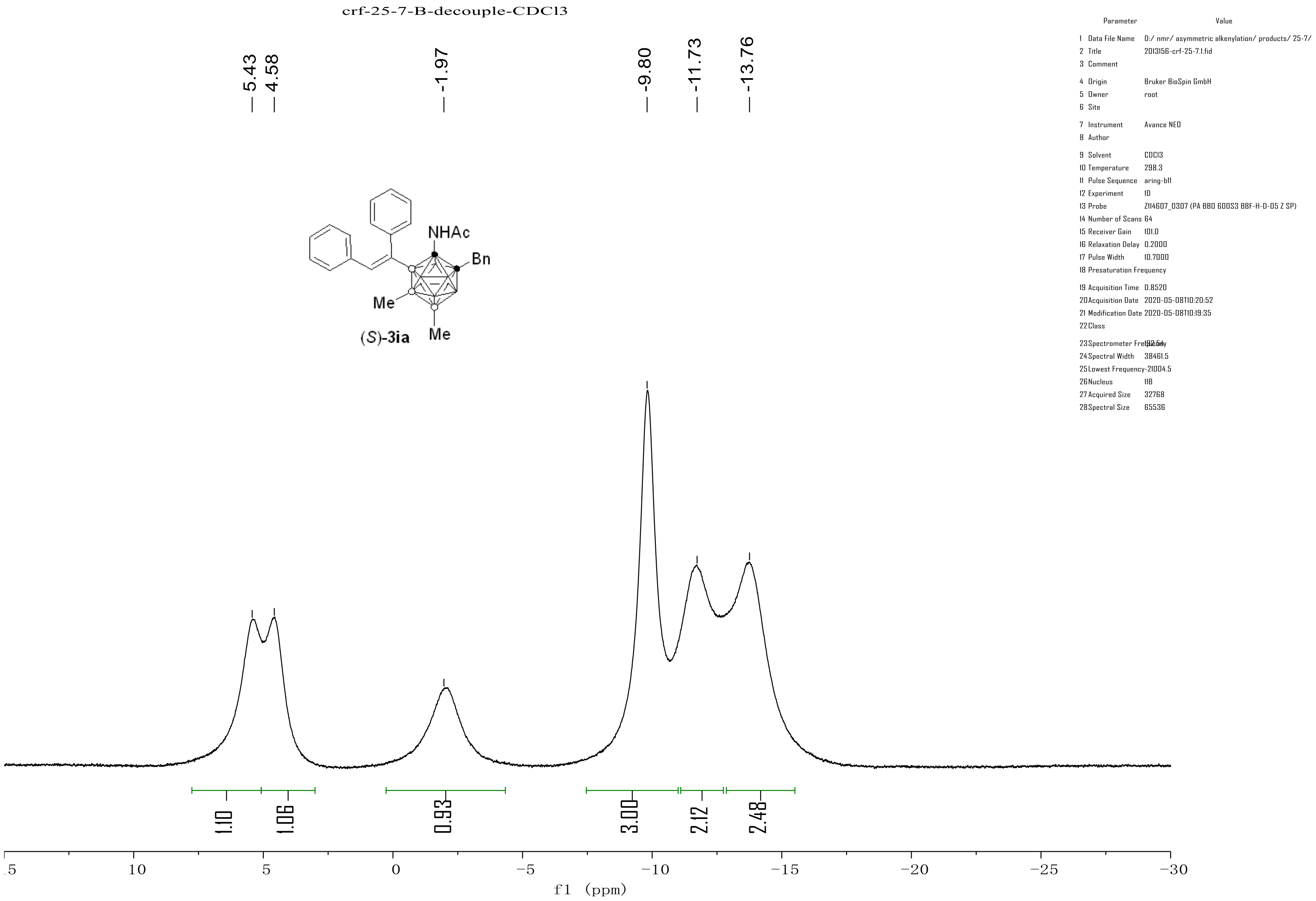

Supplementary Figure 189. ^{11}B NMR of (*S*)-**3ia**.

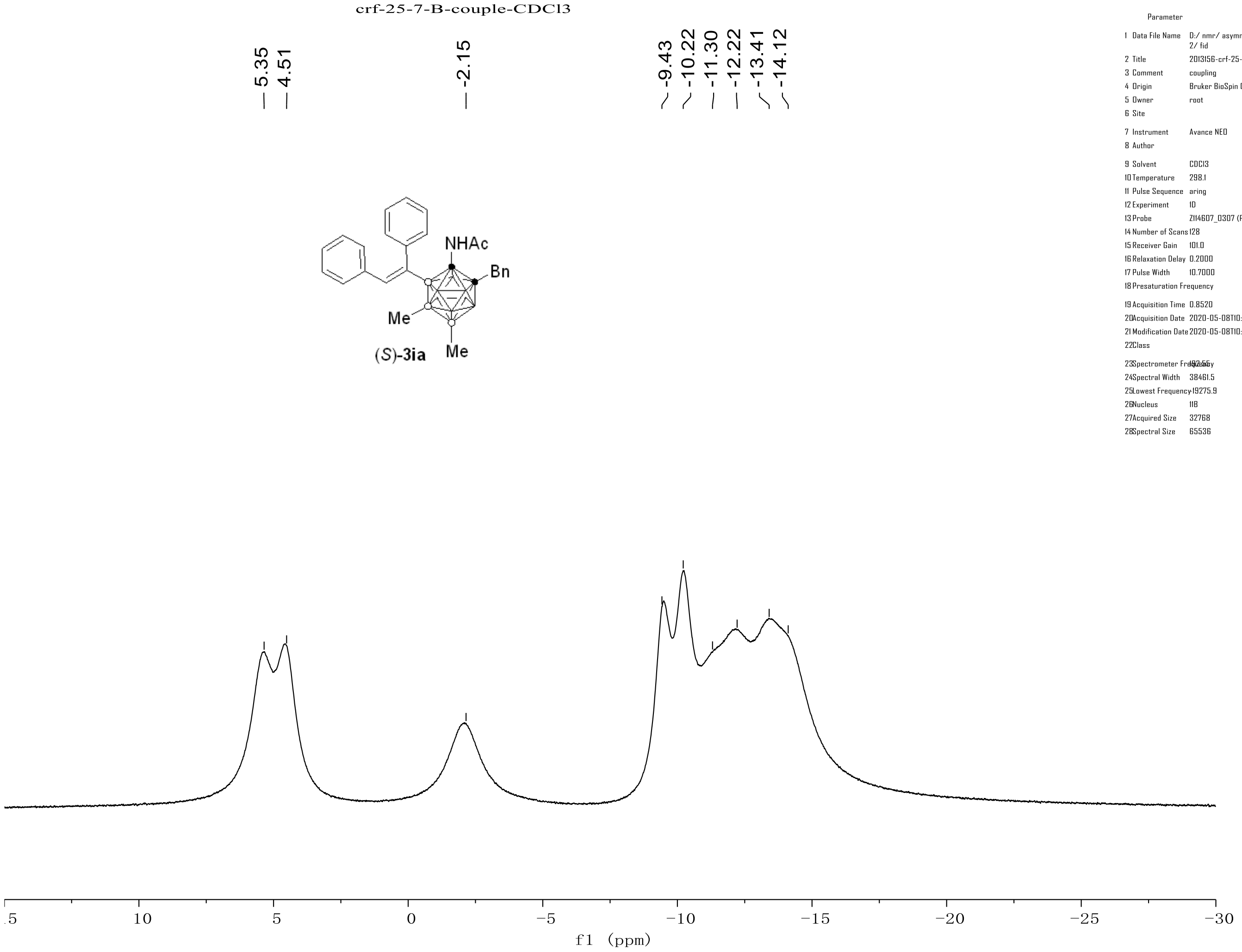

	Parameter	Value
1	Data File Name	D:/ nmr/ asymmetric alkenylation/ products/ 25-2/ fid
2	Title	2013156-crf-25-7.2.fid
3	Comment	coupling
4	Origin	Bruker BioSpin GmbH
5	Owner	root
6	Site	
7	Instrument	Avance NEO
8	Author	
9	Solvent	CDCl3
10	Temperature	298.1
11	Pulse Sequence	aring
12	Experiment	1D
13	Probe	Z114607_0307 (PA BBO 600S3 BBF-H-D-05 Z SP)
14	Number of Scans	128
15	Receiver Gain	101.0
16	Relaxation Delay	0.2000
17	Pulse Width	10.7000
18	Presaturation Frequency	
19	Acquisition Time	0.8520
20	Acquisition Date	2020-05-08T10:23:17
21	Modification Date	2020-05-08T10:21:59
22	Class	
23	Spectrometer Frequency	192.55
24	Spectral Width	38461.5
25	Lowest Frequency	-19275.9
26	Nucleus	11B
27	Acquired Size	32768
28	Spectral Size	65536

Supplementary Figure 190. ^{1}H NMR of (*S*)-**3ja**.

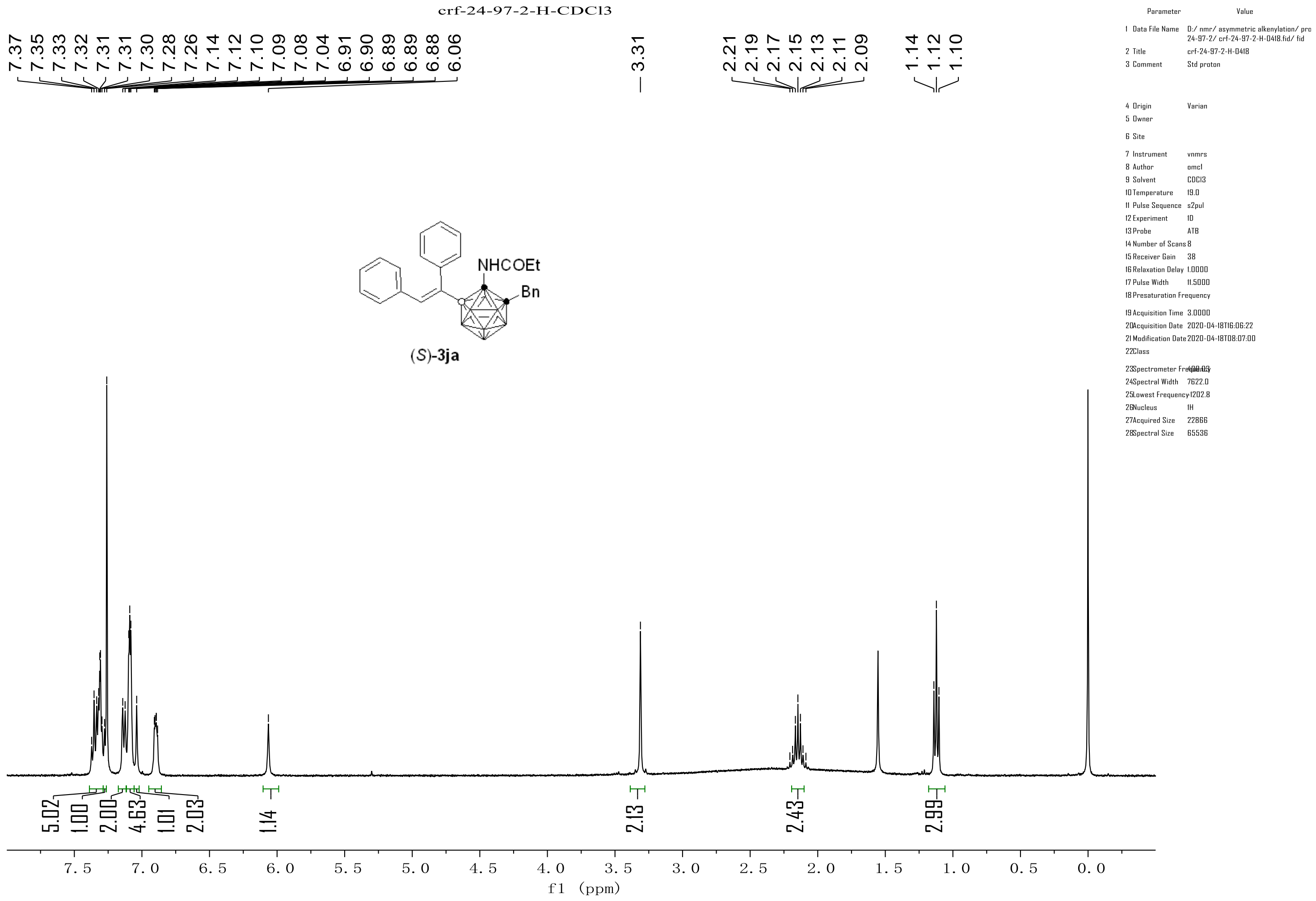

Supplementary Figure 191. $^{13}C\{^1H\}$ NMR of (*S*)-**3ja**.

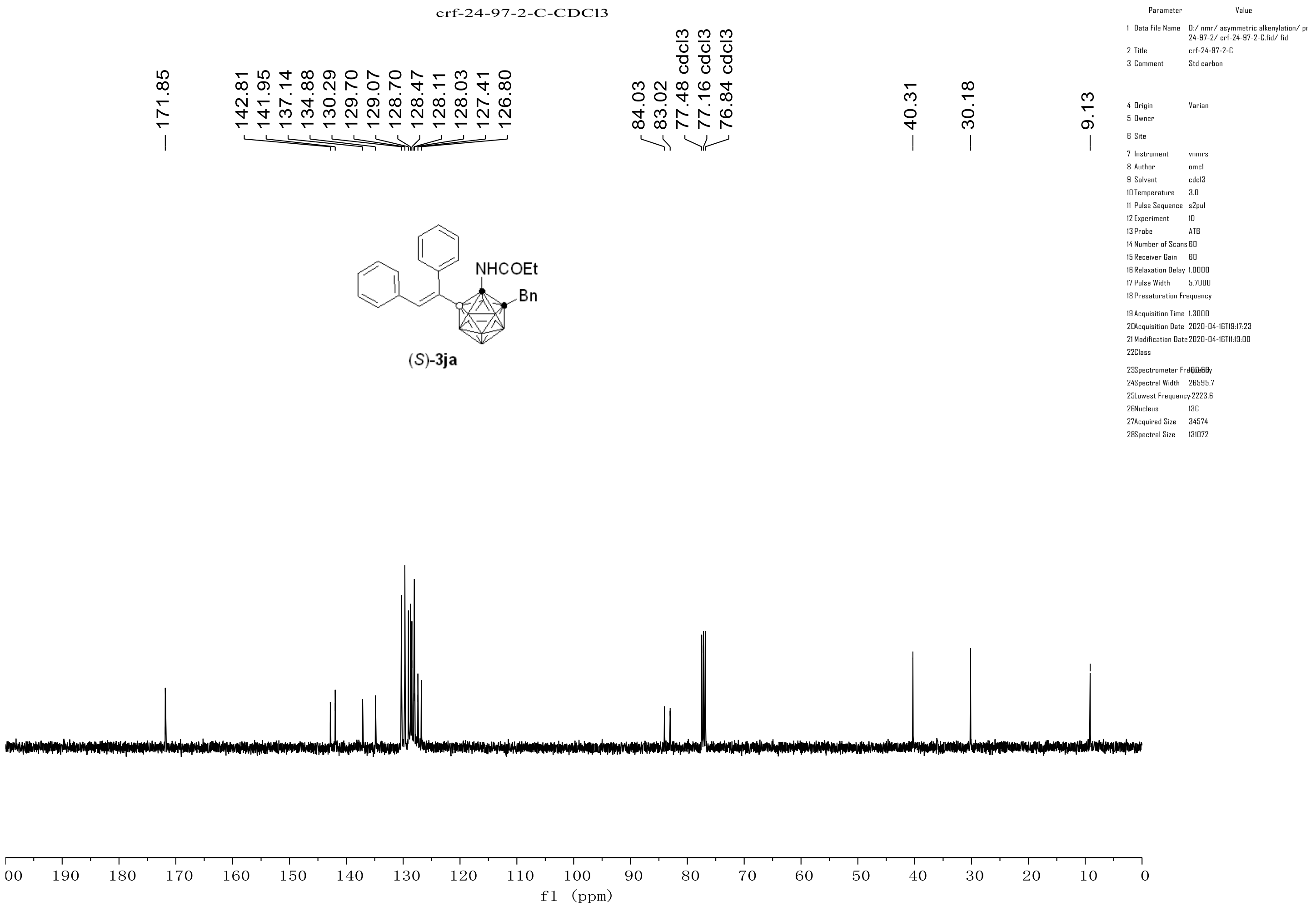

Supplementary Figure 192. $^{11}B\{^1H\}$ NMR of (*S*)-**3ja**.

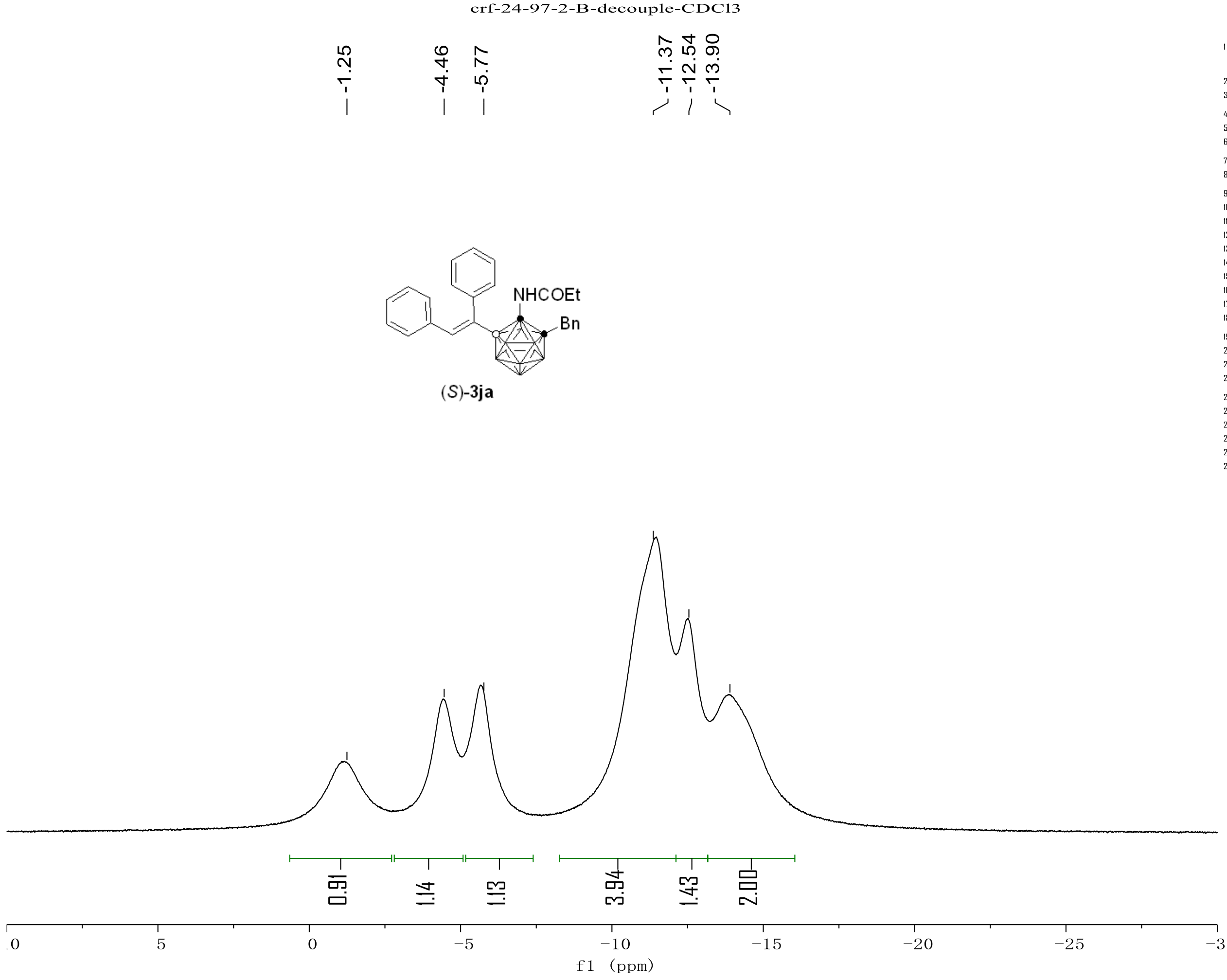

	Parameter	Value
1	Data File Name	D:/ nmr/ asymmetric alkenylation/ products/ 24-97-2/ 2013156b-24-97-2/ 1/ fid
2	Title	2013156b-24-97-2.1.fid
3	Comment	
4	Origin	Bruker BioSpin GmbH
5	Owner	root
6	Site	
7	Instrument	Avance NEO
8	Author	
9	Solvent	CDCl3
10	Temperature	298.4
11	Pulse Sequence	aring-b11
12	Experiment	1D
13	Probe	Z114607_0307 (PA BBO 600S3 BBF-
14	Number of Scans	35
15	Receiver Gain	101.0
16	Relaxation Delay	0.2000
17	Pulse Width	10.7000
18	Presaturation Frequency	
19	Acquisition Time	0.8520
20	Acquisition Date	2020-04-16T15:37:39
21	Modification Date	2020-04-16T15:37:34
22	Class	
23	Spectrometer Frequency	192.54
24	Spectral Width	38461.5
25	Lowest Frequency	-21011.6
26	Nucleus	11B
27	Acquired Size	32768
28	Spectral Size	65536

Supplementary Figure 193. ^{11}B NMR of (*S*)-**3ja**.

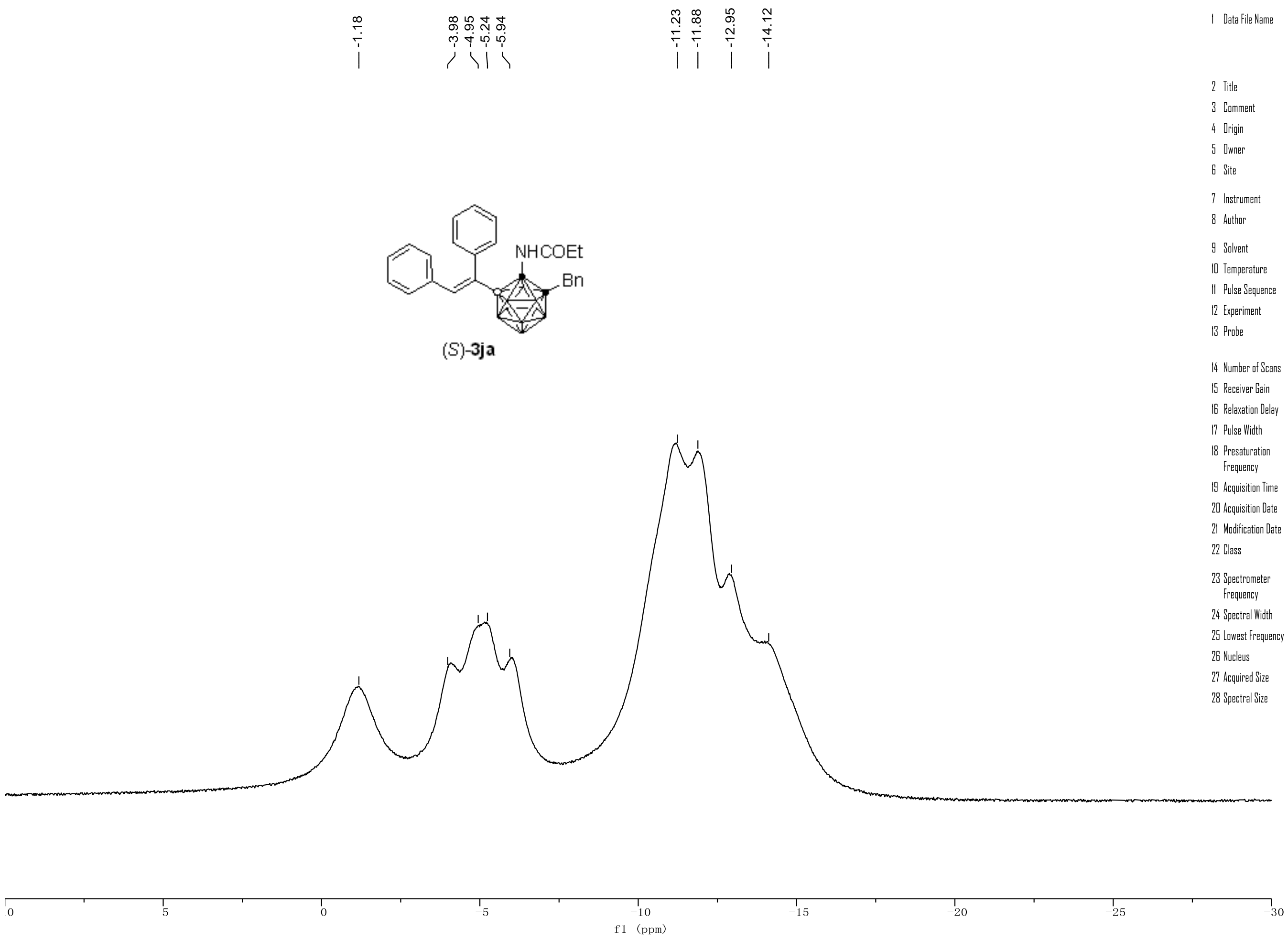

	Parameter	Value
1	Data File Name	D:/ nmr/ asymmetric alkenylation/ products/ 24-97-2/ 2013156b-24-97-2/ 2013156b-24-97-2/ 2/ fid
2	Title	2013156b-24-97-2.2.fid
3	Comment	coupling
4	Origin	Bruker BioSpin GmbH
5	Owner	root
6	Site	
7	Instrument	Avance NEO
8	Author	
9	Solvent	CDCl3
10	Temperature	298.0
11	Pulse Sequence	aring
12	Experiment	1D
13	Probe	Z114607_0307 (PA BBO 600S3 BBF-H-D-05 Z SP)
14	Number of Scans	34
15	Receiver Gain	101.0
16	Relaxation Delay	0.2000
17	Pulse Width	10.7000
18	Presaturation Frequency	
19	Acquisition Time	0.8520
20	Acquisition Date	2020-04-16T15:38:19
21	Modification Date	2020-04-16T15:38:14
22	Class	
23	Spectrometer Frequency	192.55
24	Spectral Width	38461.5
25	Lowest Frequency	-19283.0
26	Nucleus	11B
27	Acquired Size	32768
28	Spectral Size	65536

Supplementary Figure 194. ^{1}H NMR of (*S*)-**3ka**.

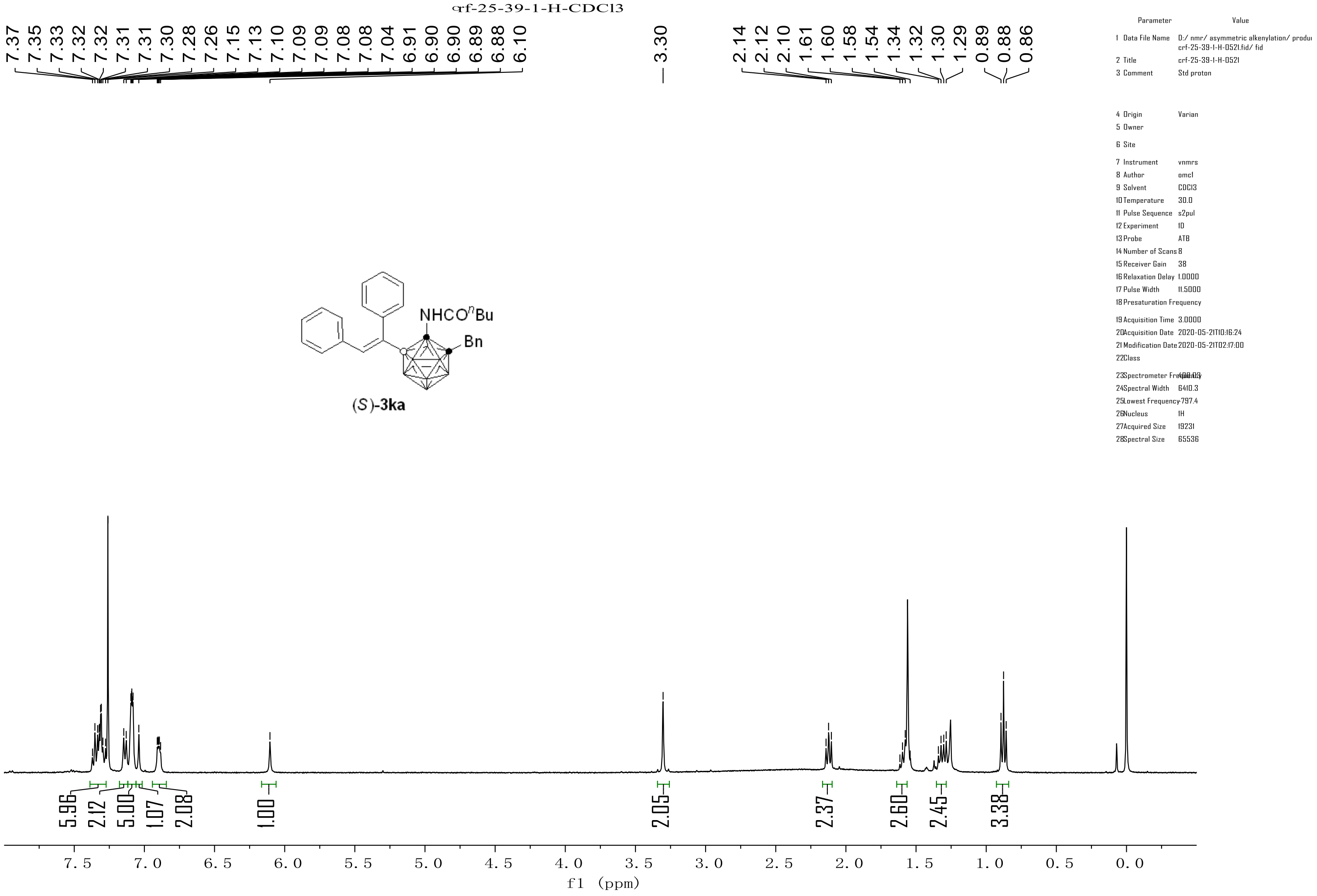

Supplementary Figure 195. $^{13}C\{^{1}H\}$ NMR of (*S*)-**3ka**.

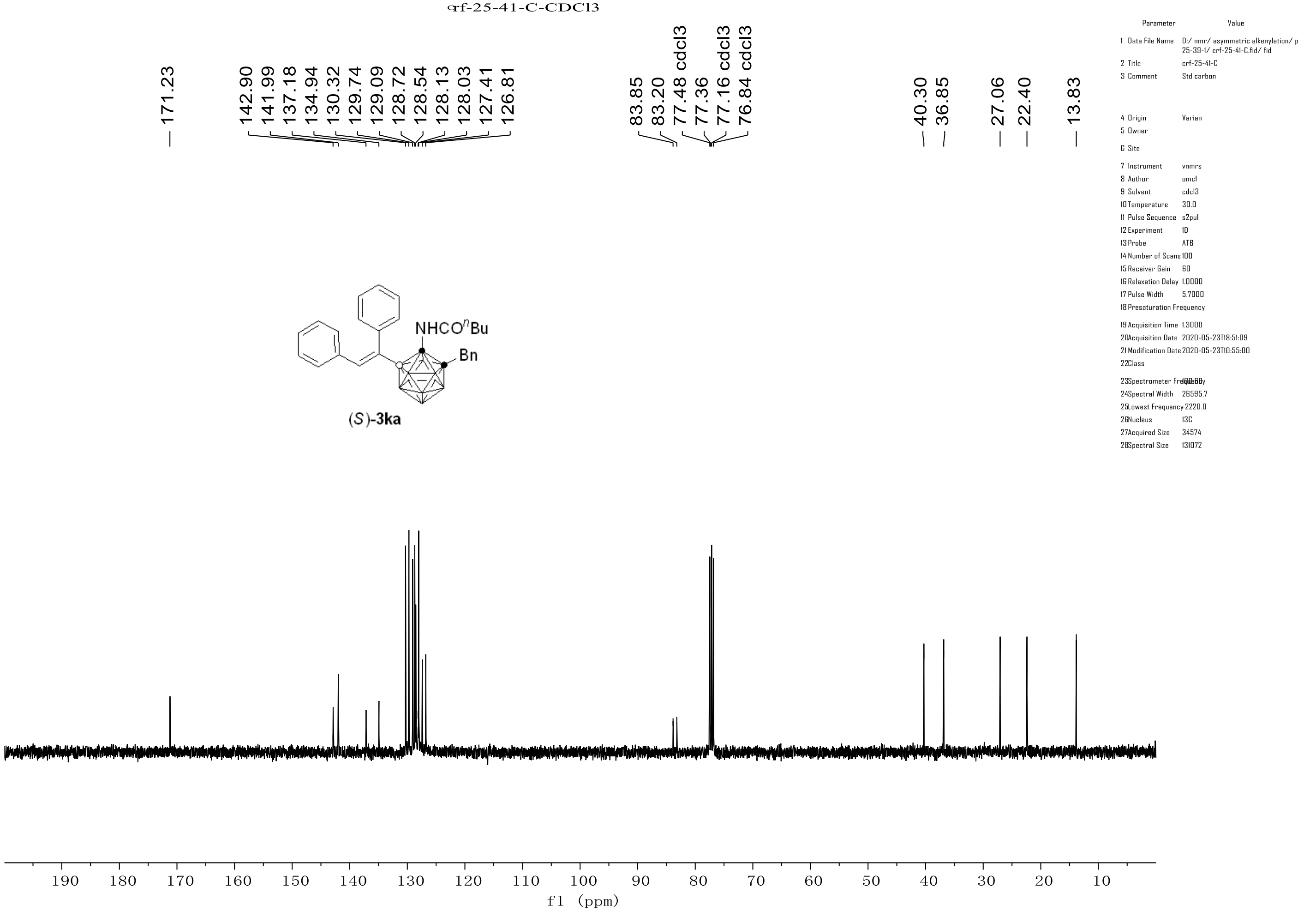

Supplementary Figure 196. $^{11}B\{^{1}H\}$ NMR of (*S*)-**3ka**.

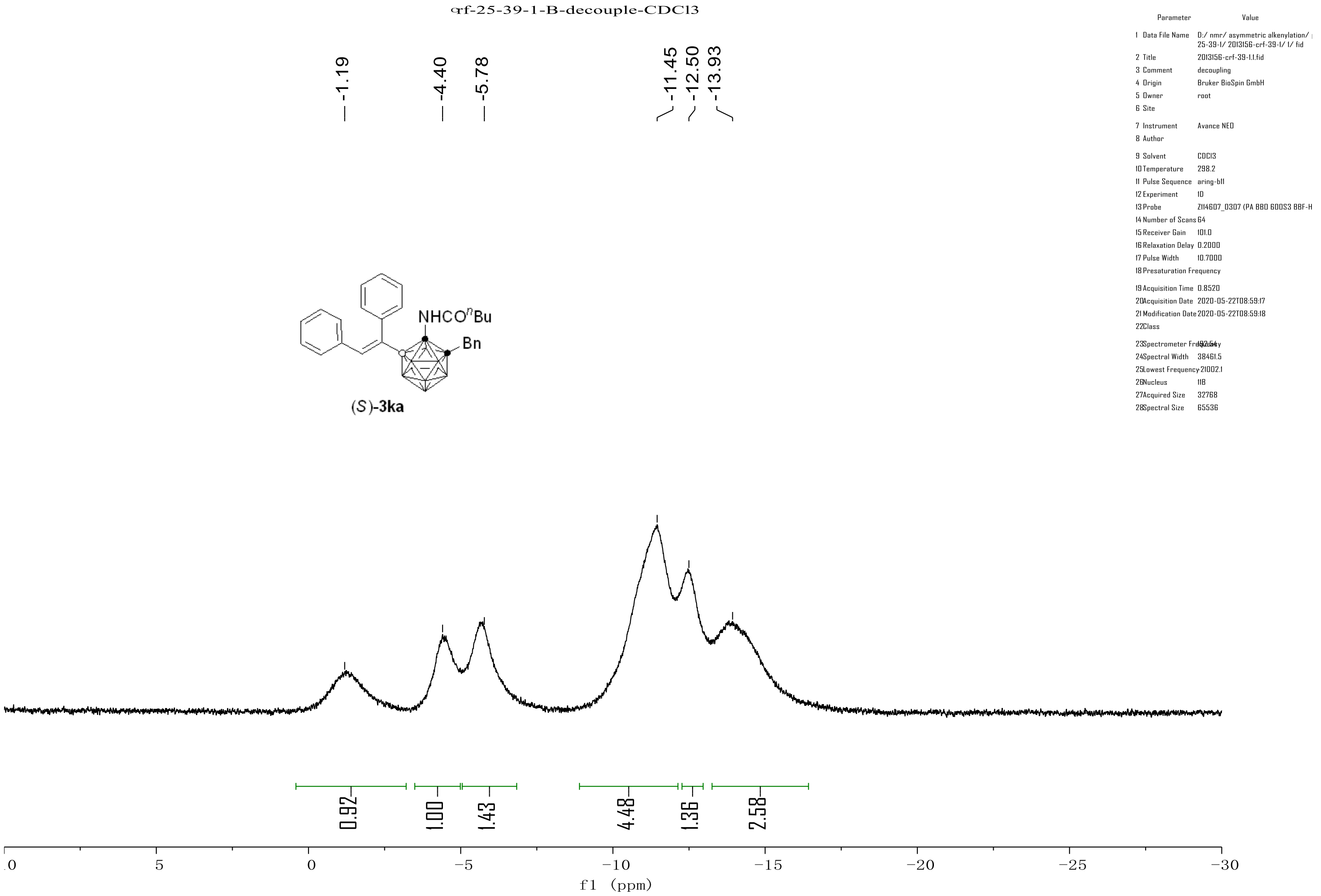

Supplementary Figure 197. ^{11}B NMR of (*S*)-**3ka**.

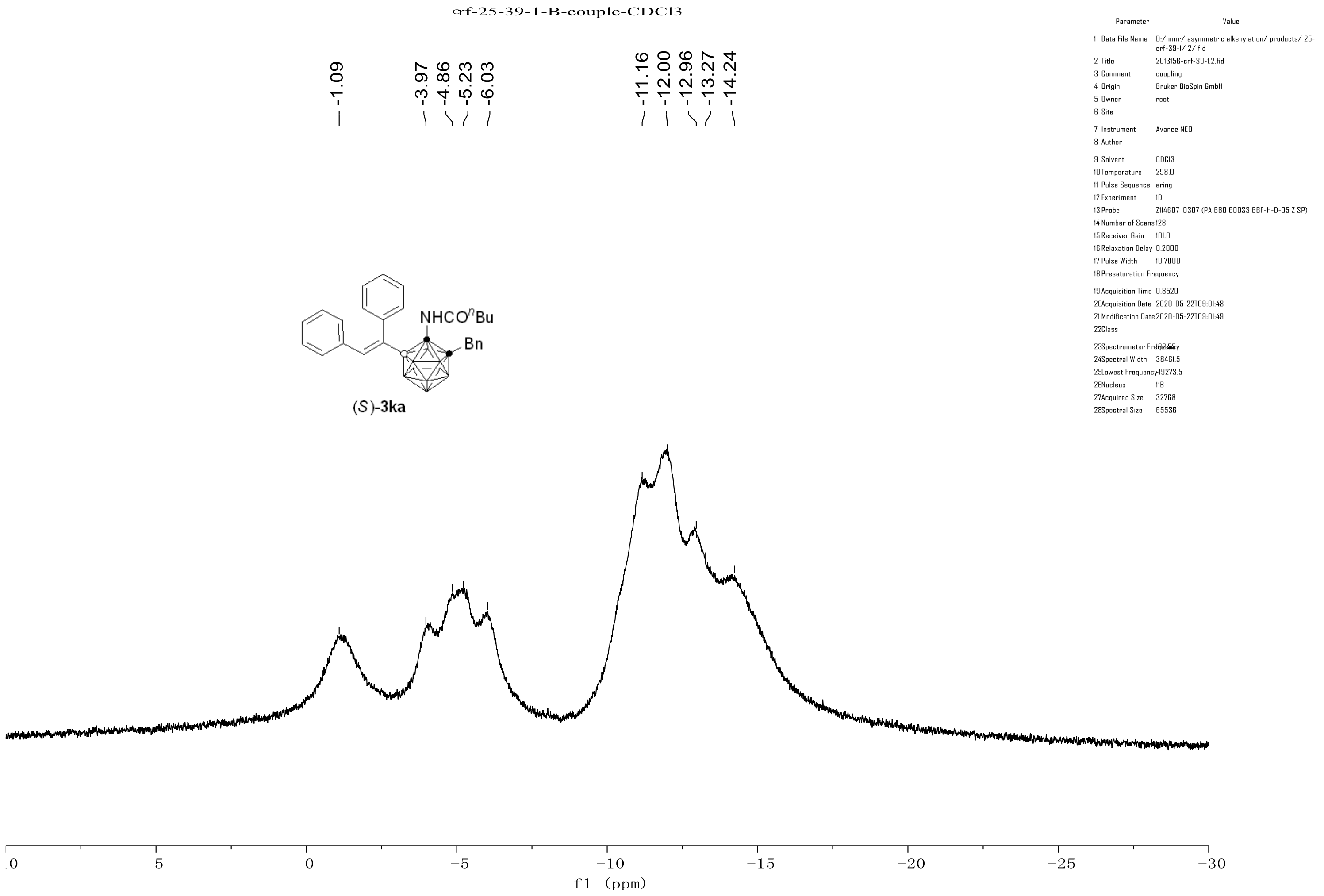

Supplementary Figure 198. ^{1}H NMR of (*S*)-**3la**.

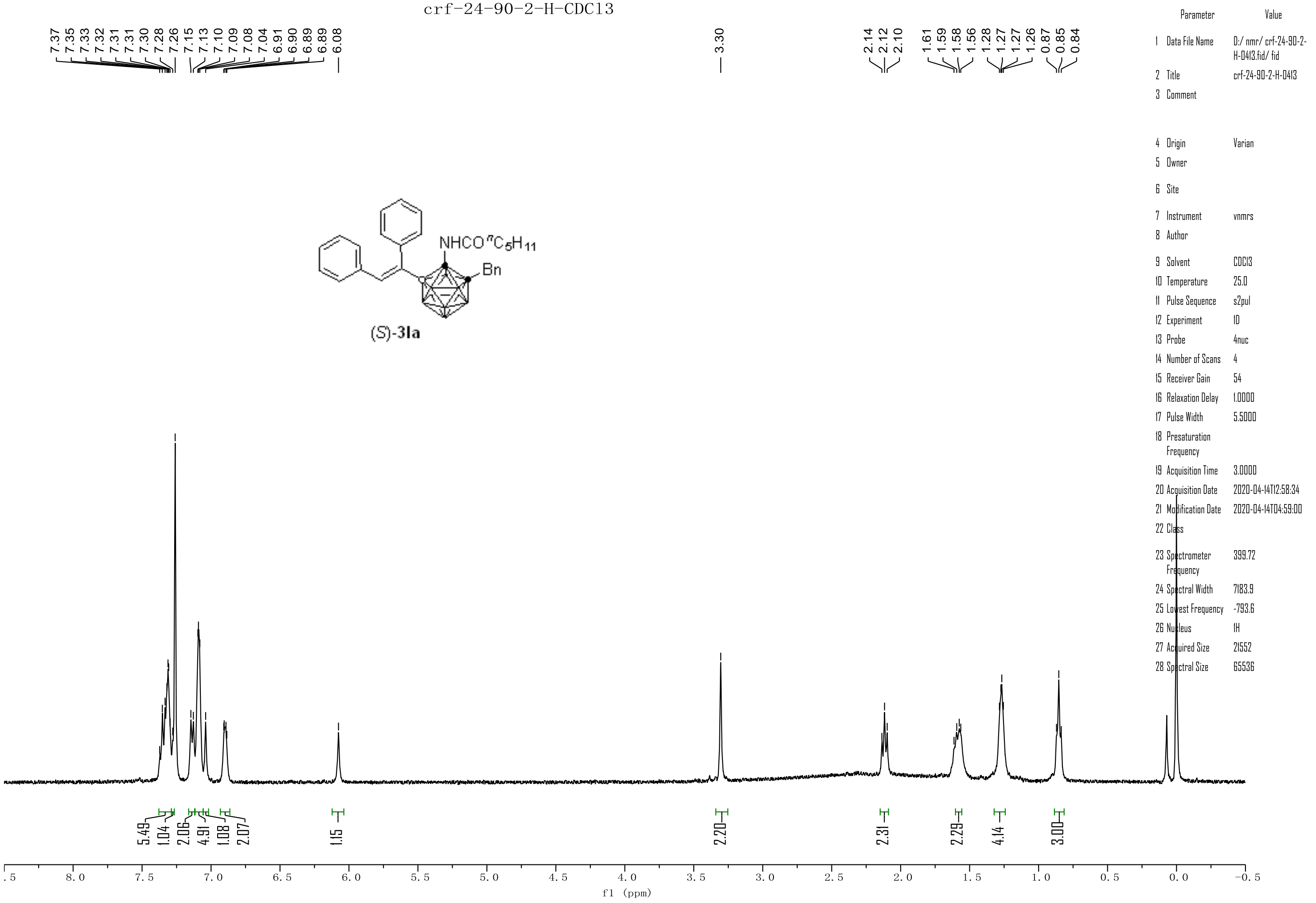

Supplementary Figure 199. $^{13}C\{^{1}H\}$ NMR of (*S*)-**3la**.

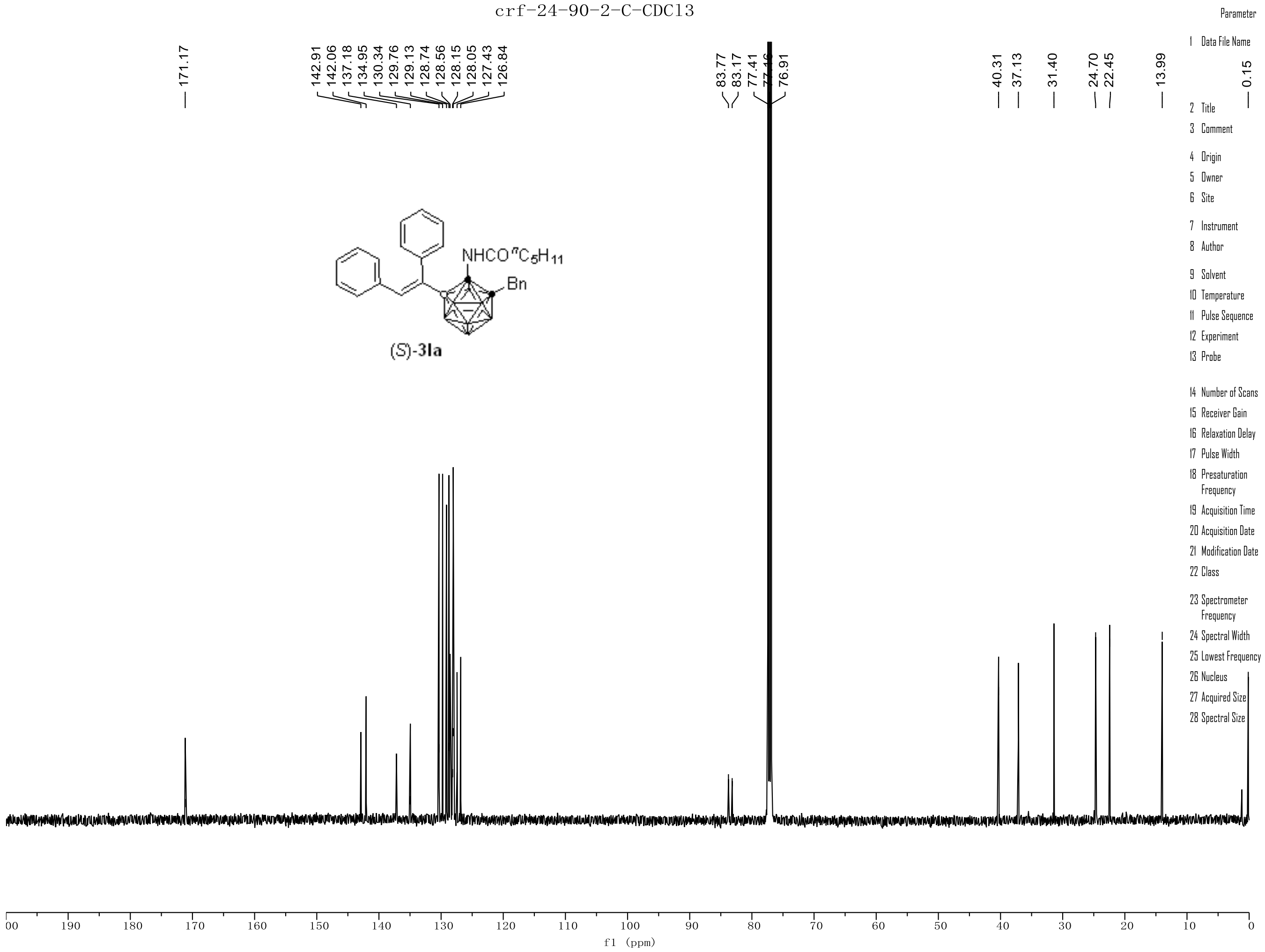

	Parameter	Value
1	Data File Name	D:/ nmr/ asymmetric alkenylation/ products/ 24-90-2/ crf-c-24-90-2-c/ crf-c-24-90-2-c/ 1/ fid
2	Title	crf-c-24-90-2-c.1.fid
3	Comment	
4	Origin	Bruker BioSpin GmbH
5	Owner	nmrsu
6	Site	
7	Instrument	Avance
8	Author	
9	Solvent	CDCl3
10	Temperature	298.1
11	Pulse Sequence	zgpg30
12	Experiment	1D
13	Probe	Z150355_0001 (CP BBO 500S1 BB-H&F-D-05 Z)
14	Number of Scans	194
15	Receiver Gain	101.0
16	Relaxation Delay	2.0000
17	Pulse Width	10.0000
18	Presaturation Frequency	
19	Acquisition Time	0.8847
20	Acquisition Date	2020-04-15T10:50:10
21	Modification Date	2020-04-15T10:49:08
22	Class	
23	Spectrometer Frequency	125.78
24	Spectral Width	37037.0
25	Lowest Frequency	-5926.6
26	Nucleus	13C
27	Acquired Size	32768
28	Spectral Size	65536

Supplementary Figure 200. $^{11}B\{^{1}H\}$ NMR of (*S*)-**3la**.

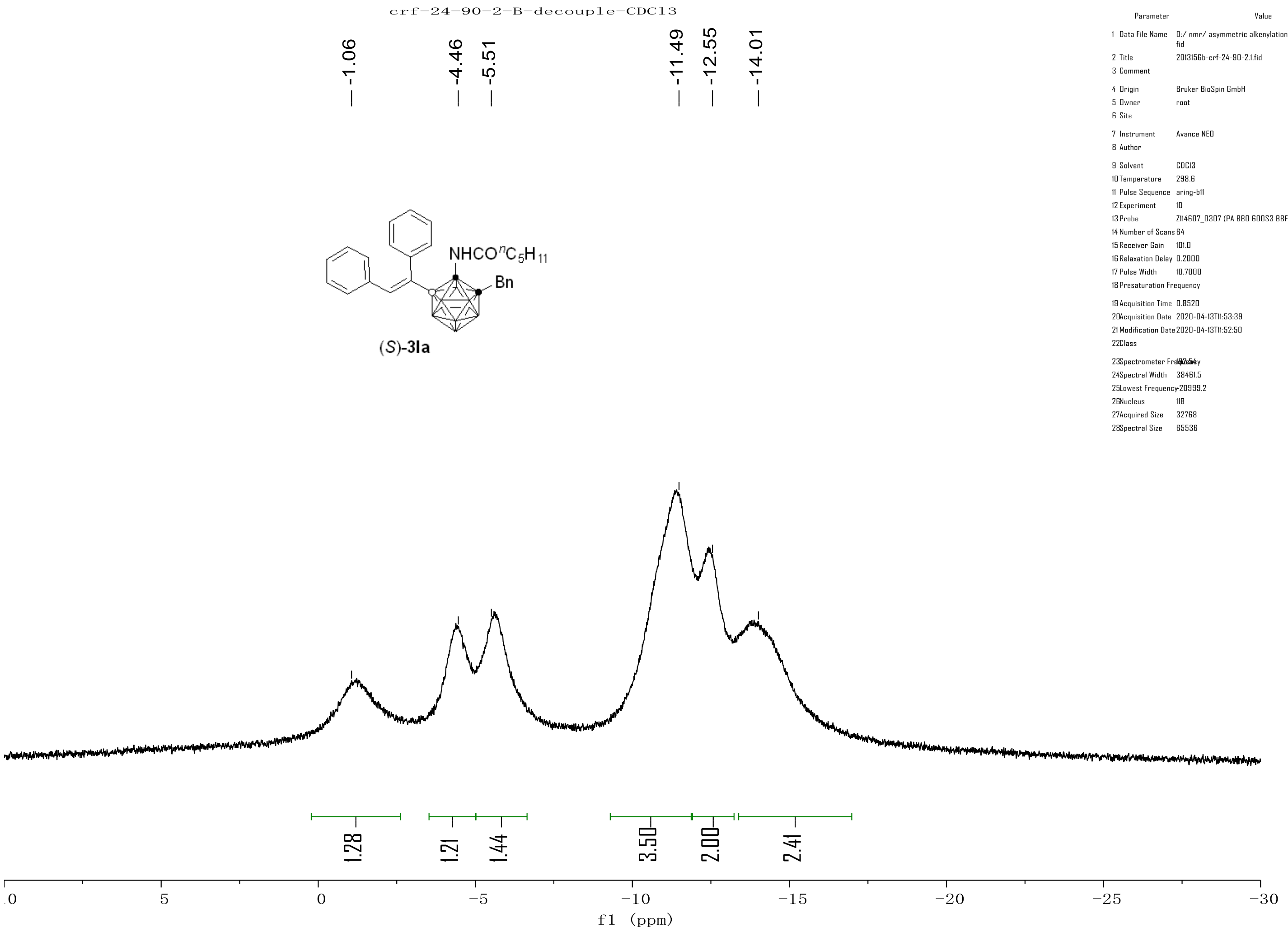

Parameter	Value
1 Data File Name	D:/ nmr/ asymmetric alkenylation/ products/ 24-90-2/ fid
2 Title	20131566-crf-24-90-2.1.fid
3 Comment	
4 Origin	Bruker BioSpin GmbH
5 Owner	root
6 Site	
7 Instrument	Avance NEO
8 Author	
9 Solvent	CDCl3
10 Temperature	298.6
11 Pulse Sequence	zgig-b11
12 Experiment	1D
13 Probe	Z114607_0307 (PA BBO 600S3 BBF-H-D-05 Z SP)
14 Number of Scans	64
15 Receiver Gain	101.0
16 Relaxation Delay	0.2000
17 Pulse Width	10.7000
18 Presaturation Frequency	
19 Acquisition Time	0.8520
20 Acquisition Date	2020-04-13T11:53:39
21 Modification Date	2020-04-13T11:52:50
22 Class	
23 Spectrometer Frequency	192.54
24 Spectral Width	38461.5
25 Lowest Frequency	-20999.2
26 Nucleus	11B
27 Acquired Size	32768
28 Spectral Size	65536

Supplementary Figure 201. ^{11}B NMR of (*S*)-**3la**.

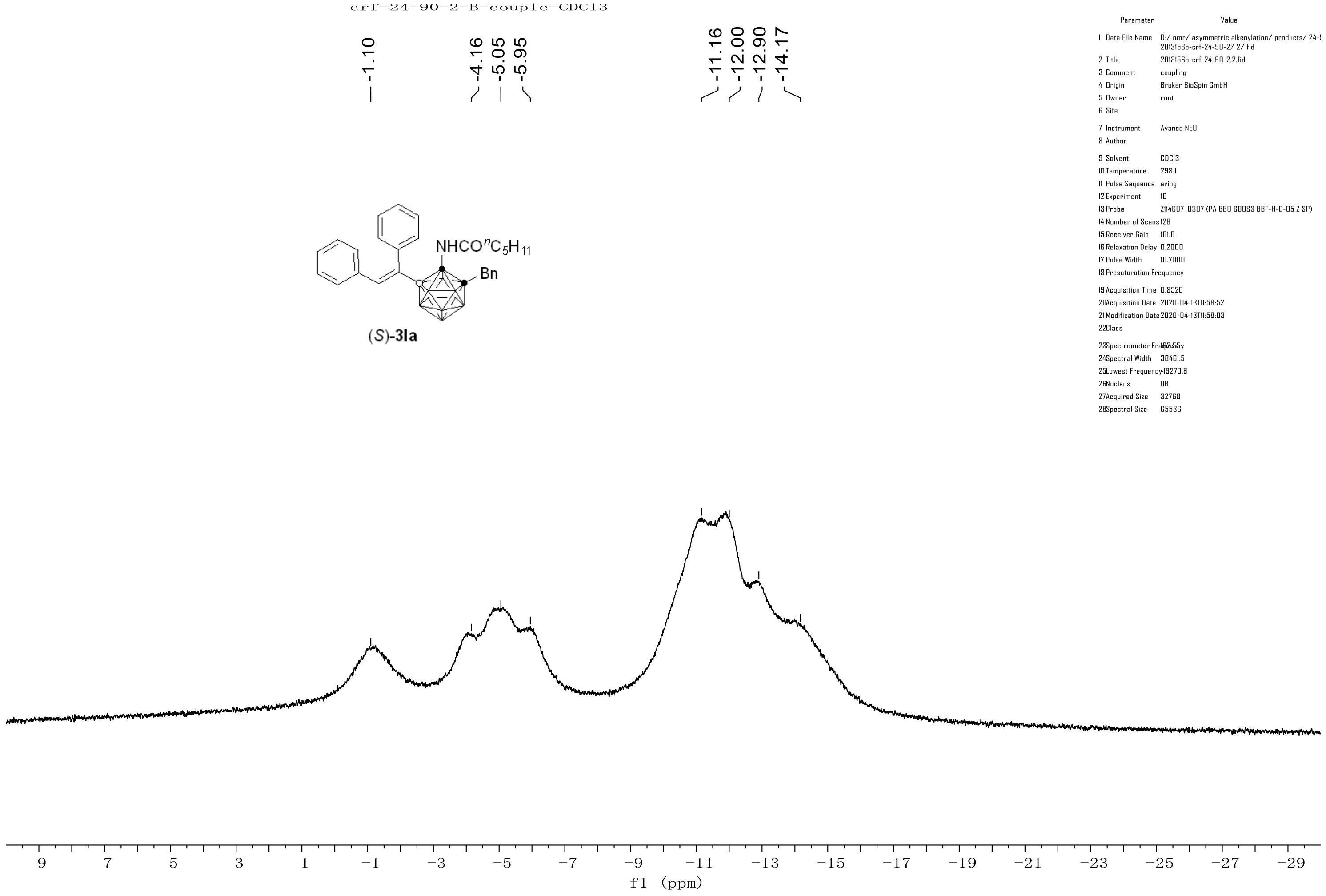

Supplementary Figure 202. ^{1}H NMR of (*S*)-**3ma**.

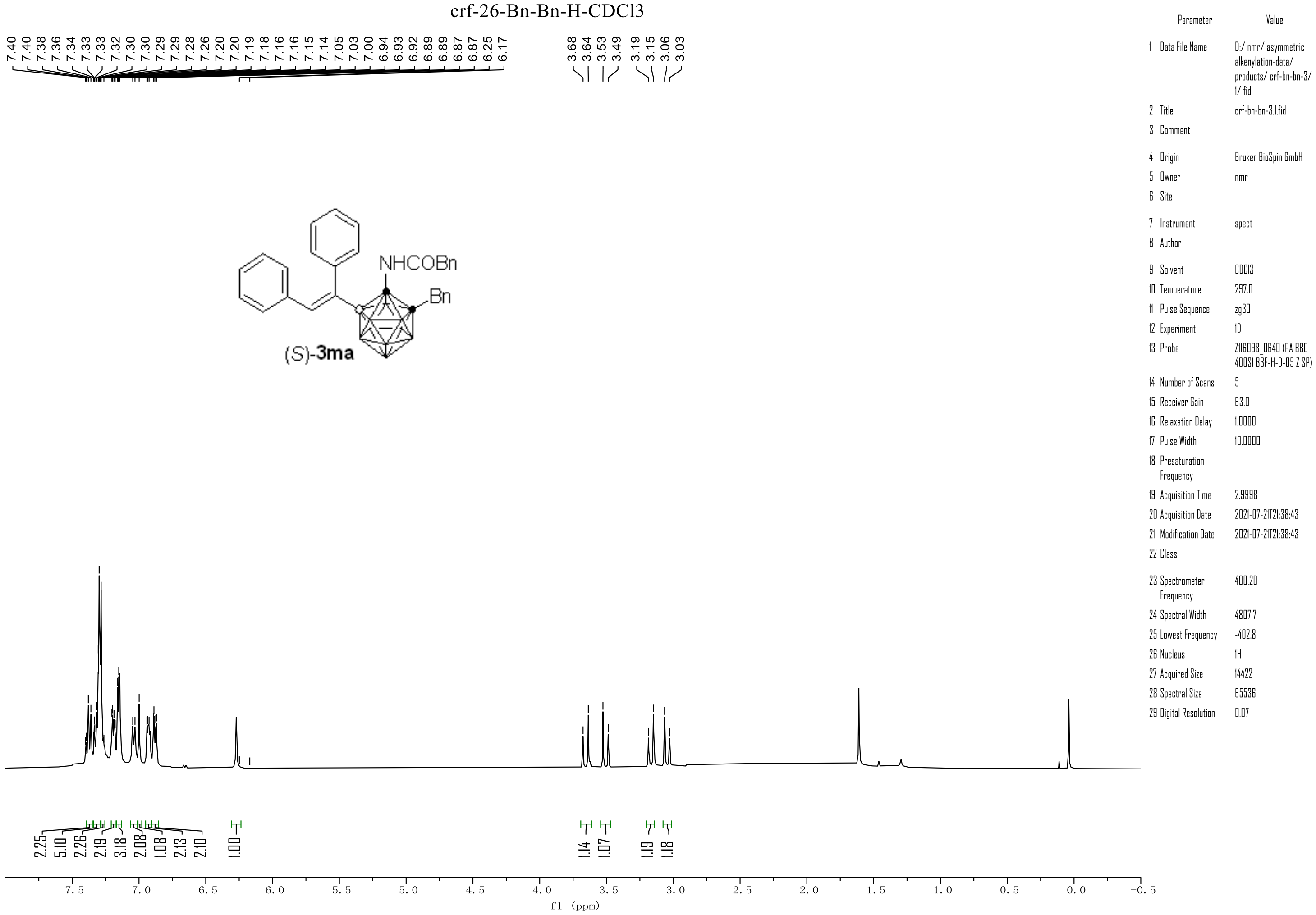

	Parameter	Value
1	Data File Name	D:/ nmr/ asymmetric alkenylation-data/ products/ crf-bn-bn-3/ 1/ fid
2	Title	crf-bn-bn-3.1.fid
3	Comment	
4	Origin	Bruker BioSpin GmbH
5	Owner	nmr
6	Site	
7	Instrument	spect
8	Author	
9	Solvent	CDCl3
10	Temperature	297.0
11	Pulse Sequence	zg30
12	Experiment	1D
13	Probe	Z116098_0640 (PA BBO 400S1 BBF-H-D-05 Z SP)
14	Number of Scans	5
15	Receiver Gain	63.0
16	Relaxation Delay	1.0000
17	Pulse Width	10.0000
18	Presaturation Frequency	
19	Acquisition Time	2.9998
20	Acquisition Date	2021-07-21T21:38:43
21	Modification Date	2021-07-21T21:38:43
22	Class	
23	Spectrometer Frequency	400.20
24	Spectral Width	4807.7
25	Lowest Frequency	-402.8
26	Nucleus	1H
27	Acquired Size	14422
28	Spectral Size	65536
29	Digital Resolution	0.07

Supplementary Figure 203. $^{13}C\{^{1}H\}$ NMR of (*S*)-**3ma**.

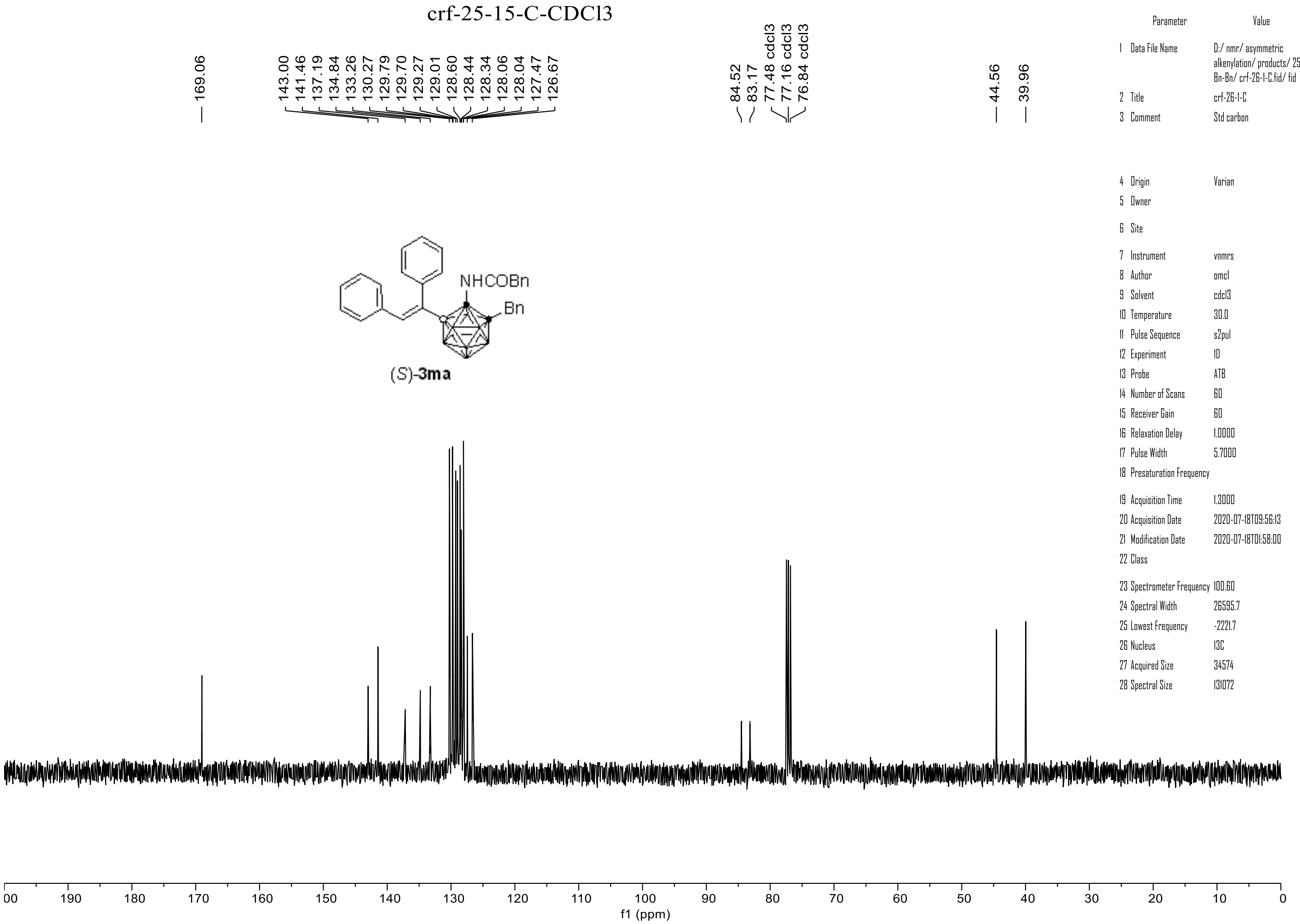

	Parameter	Value
1	Data File Name	D:/ nmr/ asymmetric alkenylation/ products/ 25-15 Bn-Bn/ crf-26-1-C.fid/ fid
2	Title	crf-26-1-C
3	Comment	Std carbon
4	Origin	Varian
5	Owner	
6	Site	
7	Instrument	vnmrs
8	Author	omcl
9	Solvent	cdcl3
10	Temperature	30.0
11	Pulse Sequence	s2pul
12	Experiment	1D
13	Probe	ATB
14	Number of Scans	60
15	Receiver Gain	60
16	Relaxation Delay	1.0000
17	Pulse Width	5.7000
18	Presaturation Frequency	
19	Acquisition Time	1.3000
20	Acquisition Date	2020-07-18T09:56:13
21	Modification Date	2020-07-18T01:58:00
22	Class	
23	Spectrometer Frequency	100.60
24	Spectral Width	26595.7
25	Lowest Frequency	-2221.7
26	Nucleus	13C
27	Acquired Size	34574
28	Spectral Size	131072

Supplementary Figure 204. $^{11}B\{^{1}H\}$ NMR of (*S*)-**3ma**.

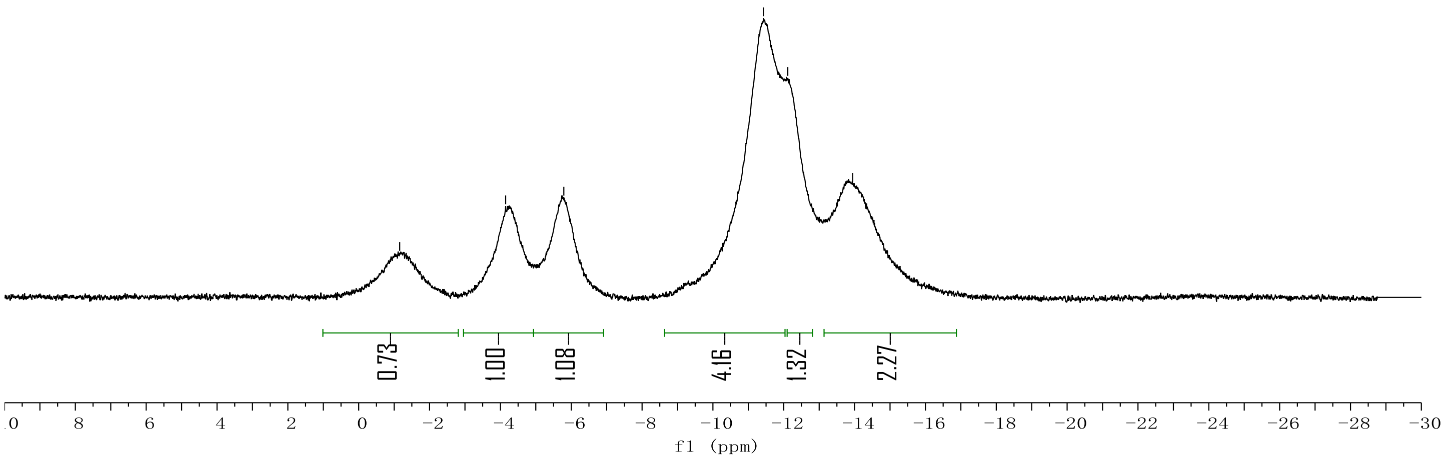

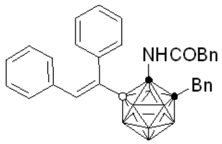

(*S*)-**3ma**

Parameter	Value
1 Data File Name	D:/ nmr/ asymmetric alkenylation/ products/ 25-1/ fid
2 Title	2013156-crf-25-15.1.fid
3 Comment	
4 Origin	Bruker BioSpin GmbH
5 Owner	root
6 Site	
7 Instrument	Avance NEO
8 Author	
9 Solvent	CDCl3
10 Temperature	298.3
11 Pulse Sequence	aring-b11
12 Experiment	1D
13 Probe	Z114607_0307 (PA BBO 600S3 BBF-H-D-05 Z SP)
14 Number of Scans	64
15 Receiver Gain	101.0
16 Relaxation Delay	0.2000
17 Pulse Width	10.7000
18 Presaturation Frequency	
19 Acquisition Time	0.8520
20 Acquisition Date	2020-05-08T10:37:11
21 Modification Date	2020-05-08T10:35:53
22 Class	
23 Spectrometer Frequency	192.54
24 Spectral Width	38461.5
25 Lowest Frequency	-21004.5
26 Nucleus	11B
27 Acquired Size	32768
28 Spectral Size	65536

Supplementary Figure 205. ^{11}B NMR of (*S*)-**3ma**.

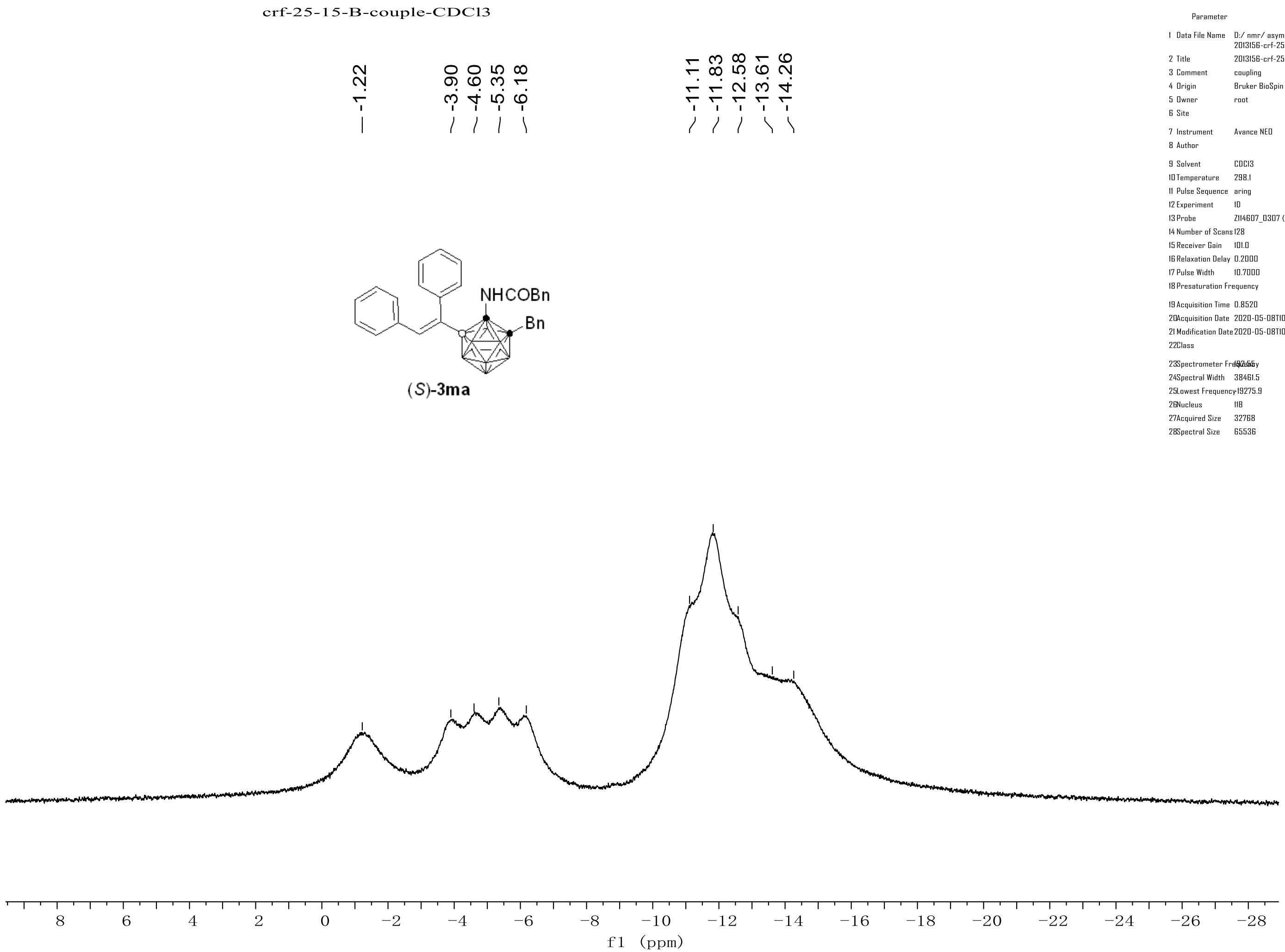

	Parameter	Value
1	Data File Name	D:/ nmr/ asymmetric alkenylation/ produc 2013156-crf-25-15/ 2/ fid
2	Title	2013156-crf-25-15.2.fid
3	Comment	coupling
4	Origin	Bruker BioSpin GmbH
5	Owner	root
6	Site	
7	Instrument	Avance NEO
8	Author	
9	Solvent	CDCl3
10	Temperature	298.1
11	Pulse Sequence	aring
12	Experiment	1D
13	Probe	Z114607_0307 (PA BBO 600S3 BBF-H-D-05
14	Number of Scans	128
15	Receiver Gain	101.0
16	Relaxation Delay	0.2000
17	Pulse Width	10.7000
18	Presaturation Frequency	
19	Acquisition Time	0.8520
20	Acquisition Date	2020-05-08T10:39:36
21	Modification Date	2020-05-08T10:38:18
22	Class	
23	Spectrometer Frequency	192.55
24	Spectral Width	38461.5
25	Lowest Frequency	-19275.9
26	Nucleus	11B
27	Acquired Size	32768
28	Spectral Size	65536

Supplementary Figure 206. ^{1}H NMR of (*S*)-**3na**.

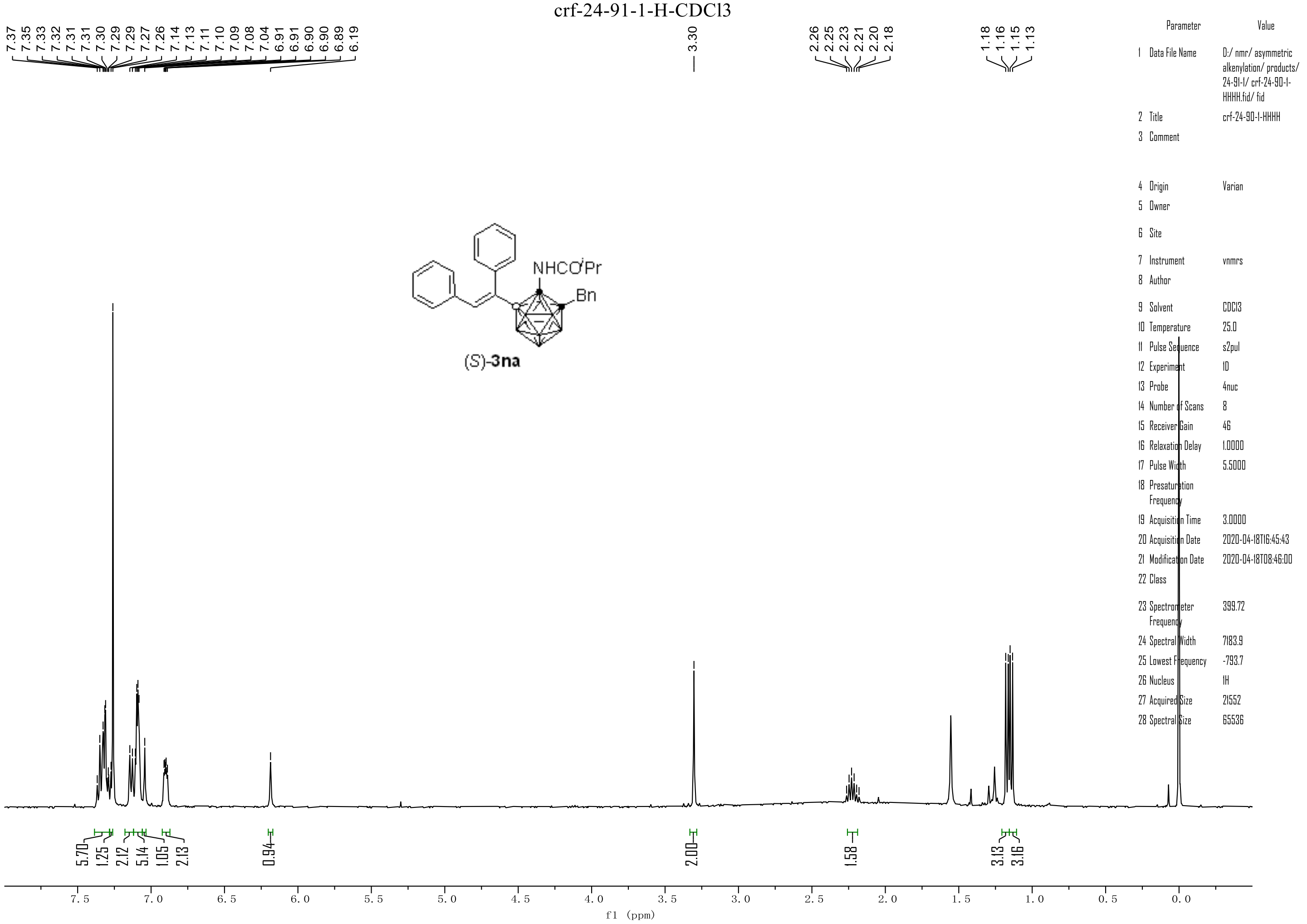

Supplementary Figure 207. $^{13}C\{^{1}H\}$ NMR of (*S*)-**3na**.

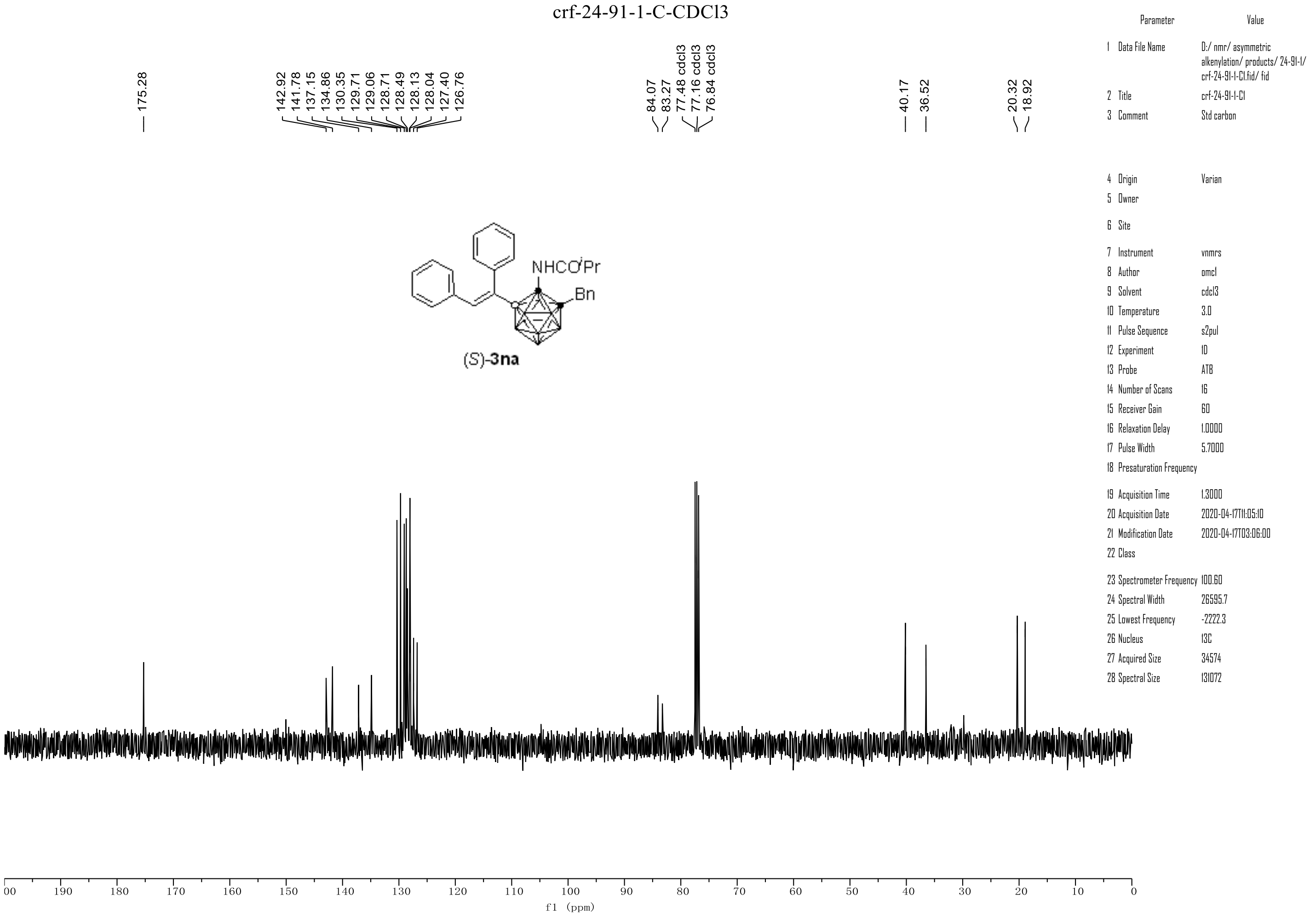

Supplementary Figure 208. $^{11}B\{^1H\}$ NMR of (*S*)-**3na**.

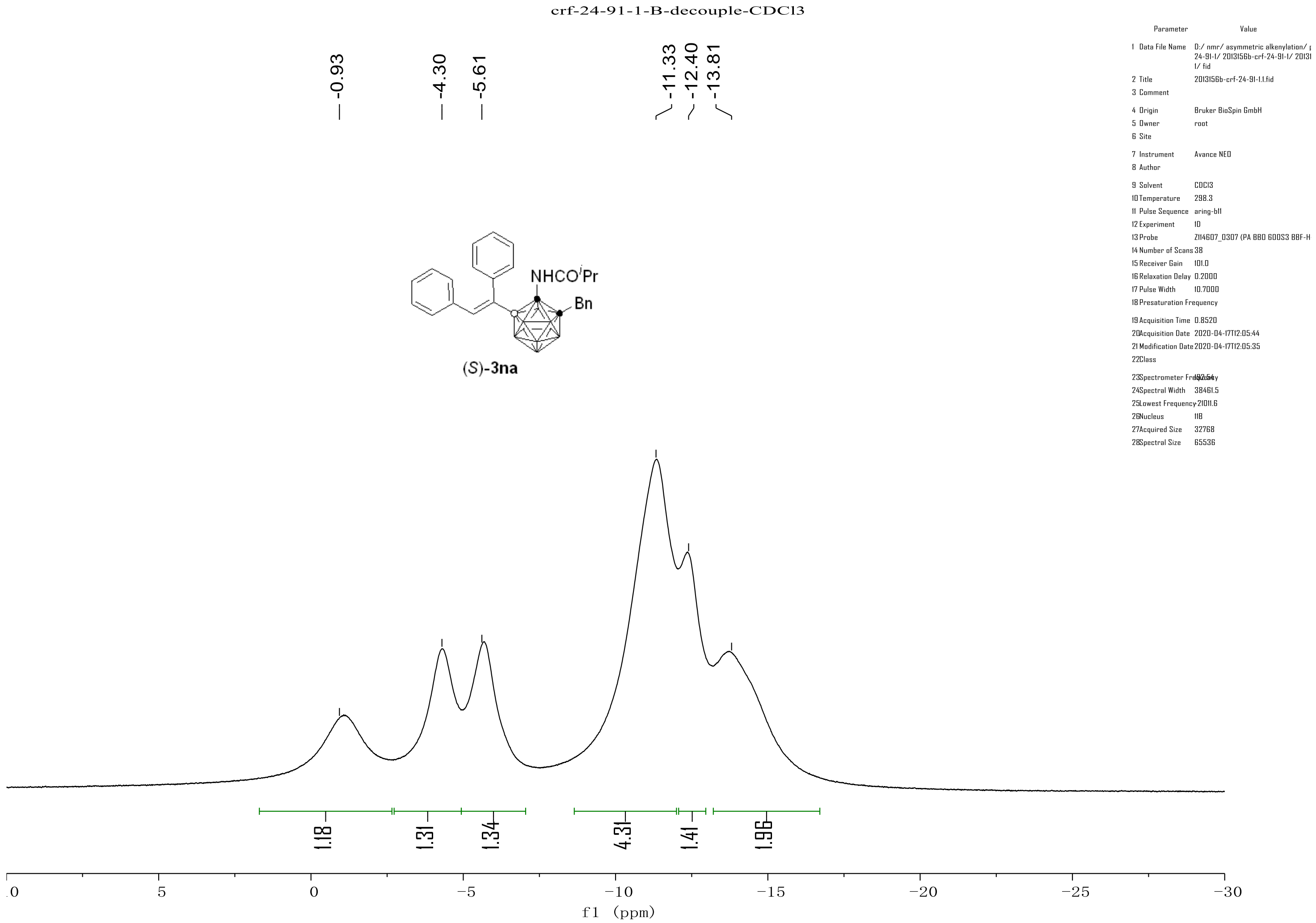

Supplementary Figure 209. ^{11}B NMR of (*S*)-**3na**.

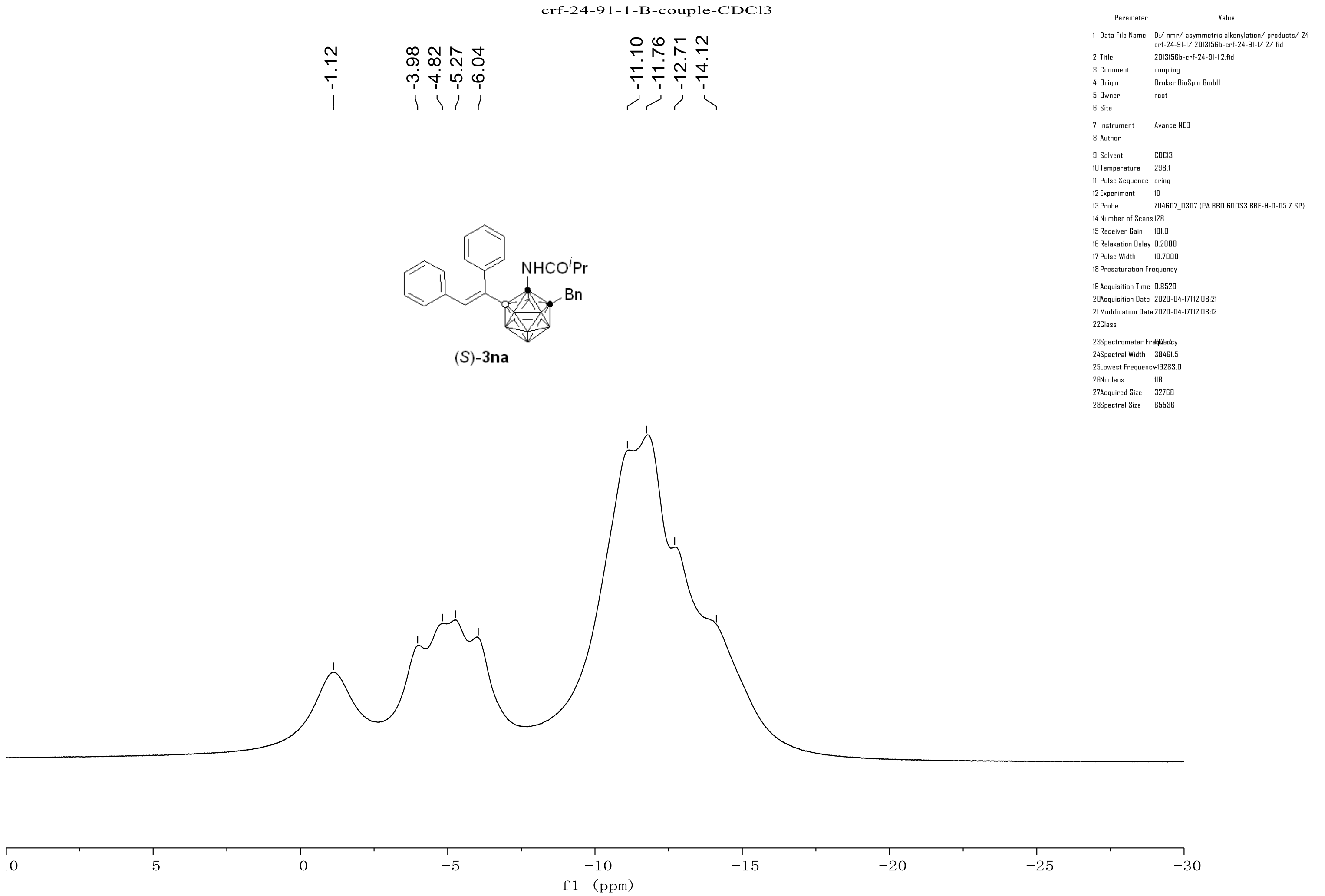

Supplementary Figure 210. ^{1}H NMR of (*S*)-**3oa**.

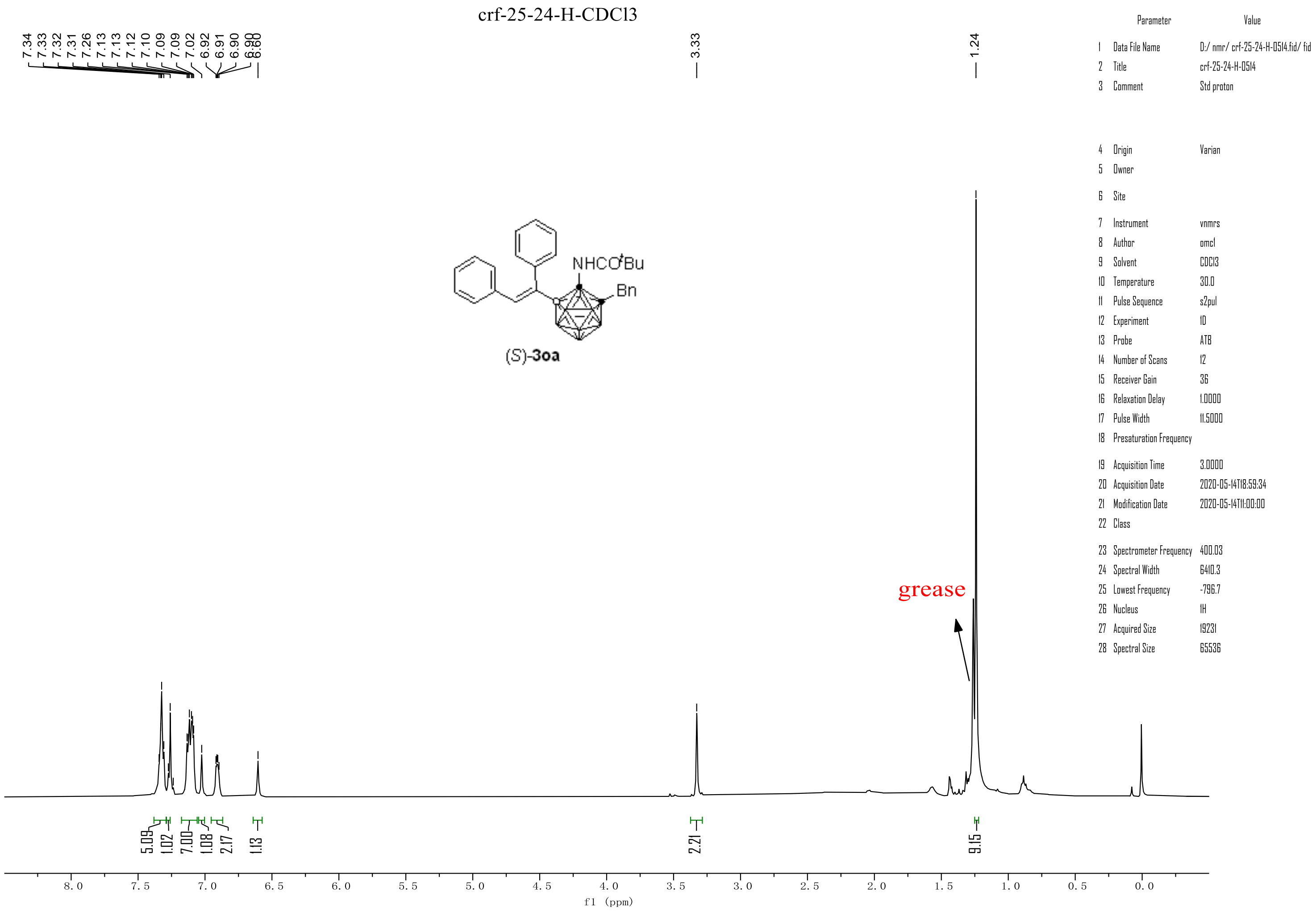

Supplementary Figure 211. $^{13}C\{^1H\}$ NMR of (*S*)-**3oa**.

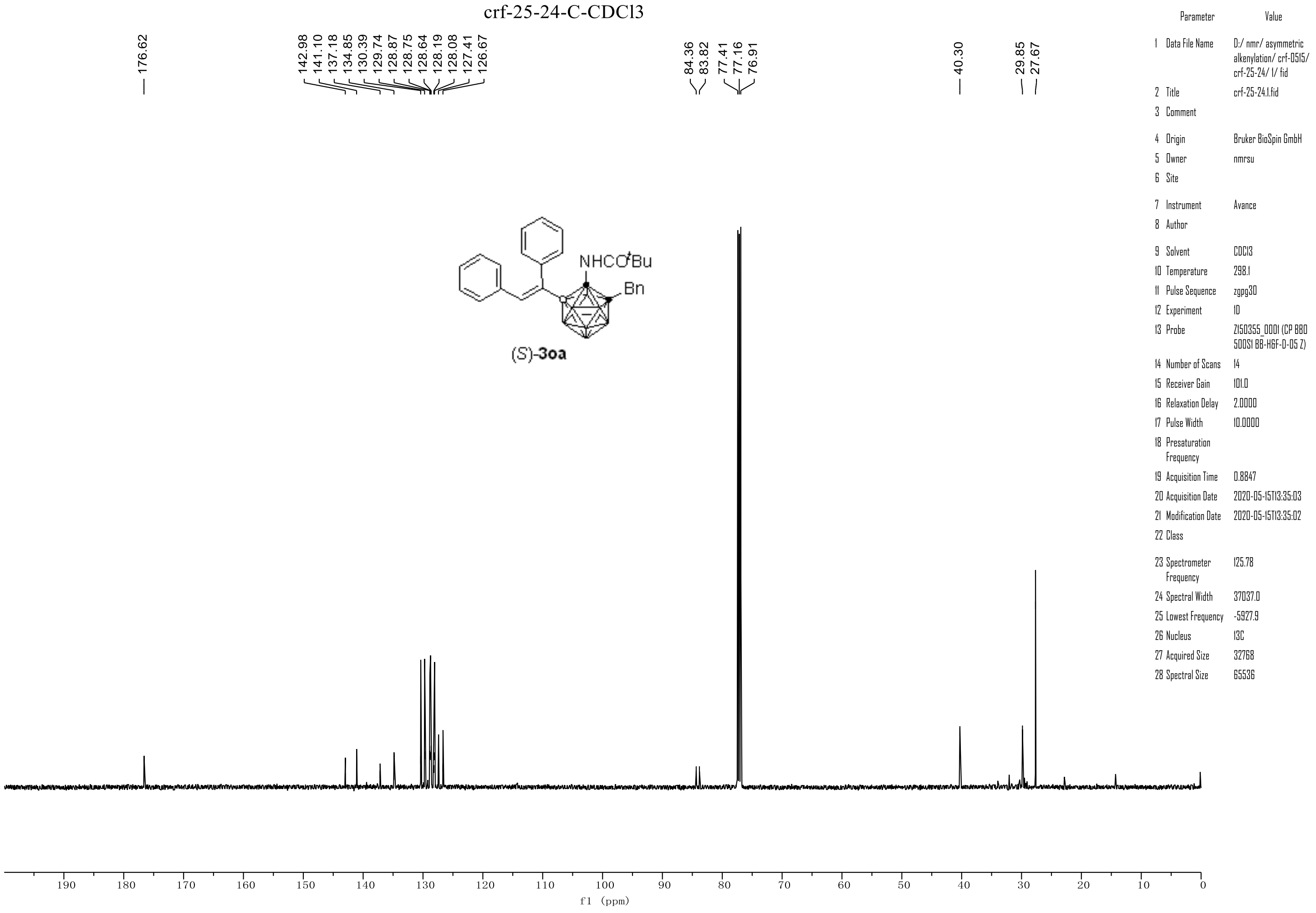

Supplementary Figure 212. $^{11}B\{^1H\}$ NMR of (*S*)-**3oa**.

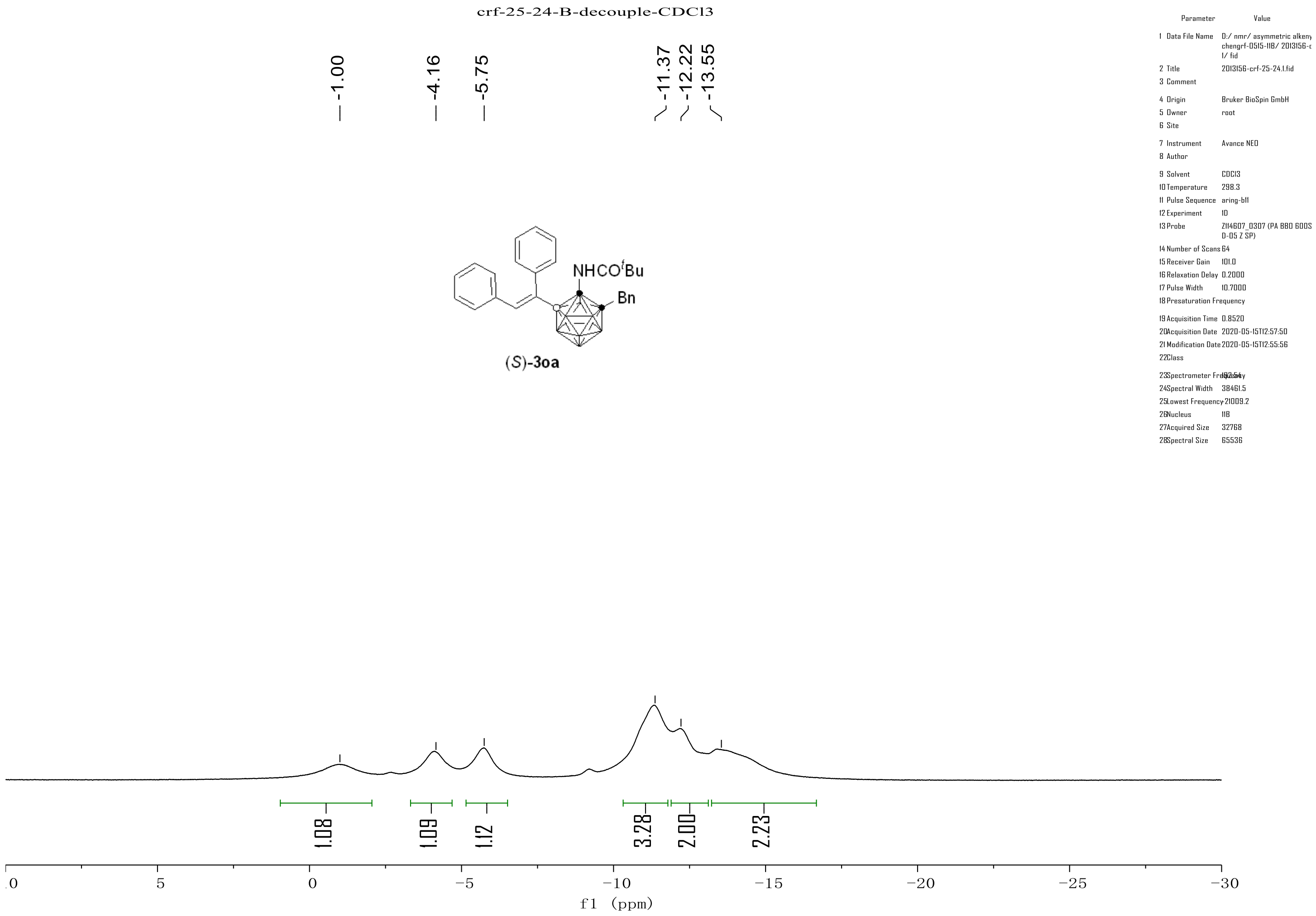

	Parameter	Value
1	Data File Name	D:/ nmr/ asymmetric alkeny chengrf-0515-11B/ 2013156-c 1/ fid
2	Title	2013156-crf-25-24.1.fid
3	Comment	
4	Origin	Bruker BioSpin GmbH
5	Owner	root
6	Site	
7	Instrument	Avance NEO
8	Author	
9	Solvent	CDCl3
10	Temperature	298.3
11	Pulse Sequence	aring-b11
12	Experiment	1D
13	Probe	Z114607_0307 (PA BBO 600S D-05 Z SP)
14	Number of Scans	64
15	Receiver Gain	101.0
16	Relaxation Delay	0.2000
17	Pulse Width	10.7000
18	Presaturation Frequency	
19	Acquisition Time	0.8520
20	Acquisition Date	2020-05-15T12:57:50
21	Modification Date	2020-05-15T12:55:56
22	Class	
23	Spectrometer Frequency	192.54
24	Spectral Width	38461.5
25	Lowest Frequency	-21009.2
26	Nucleus	11B
27	Acquired Size	32768
28	Spectral Size	65536

Supplementary Figure 213. ^{11}B NMR of (*S*)-**3oa**.

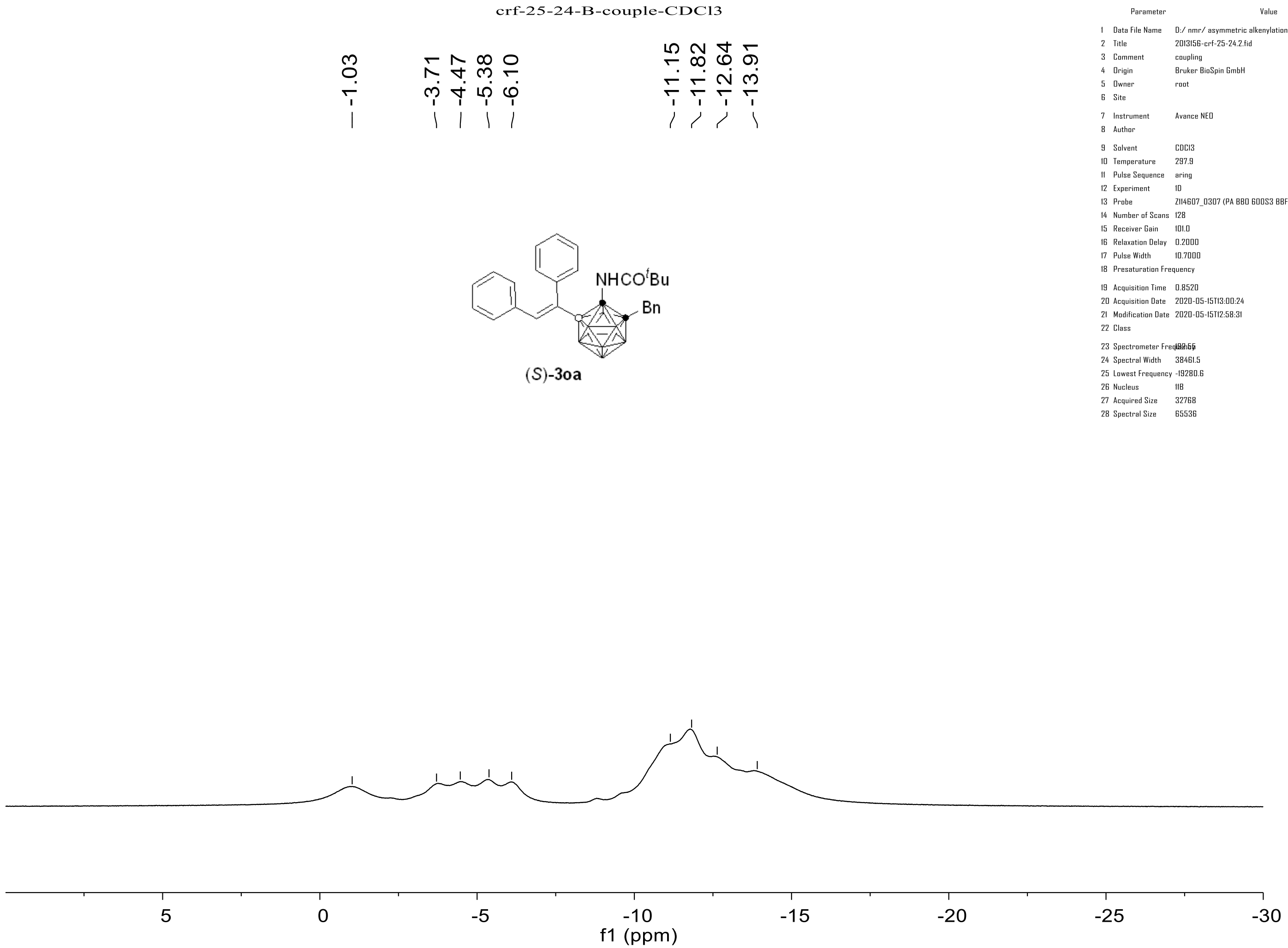

	Parameter	Value
1	Data File Name	D:/ nmr/ asymmetric alkenylation/ products/ 25-24 Bn-tBı
2	Title	2013156-crf-25-24.2.fid
3	Comment	coupling
4	Origin	Bruker BioSpin GmbH
5	Owner	root
6	Site	
7	Instrument	Avance NEO
8	Author	
9	Solvent	CDCl3
10	Temperature	297.9
11	Pulse Sequence	aring
12	Experiment	1D
13	Probe	Z114607_0307 (PA BBO 600S3 BBF-H-D-05 Z SP)
14	Number of Scans	128
15	Receiver Gain	101.0
16	Relaxation Delay	0.2000
17	Pulse Width	10.7000
18	Presaturation Frequency	
19	Acquisition Time	0.8520
20	Acquisition Date	2020-05-15T13:00:24
21	Modification Date	2020-05-15T12:58:31
22	Class	
23	Spectrometer Frequency	192.55
24	Spectral Width	38461.5
25	Lowest Frequency	-19280.6
26	Nucleus	11B
27	Acquired Size	32768
28	Spectral Size	65536

Supplementary Figure 214. ^{1}H NMR of (*S*)-**3pa**.

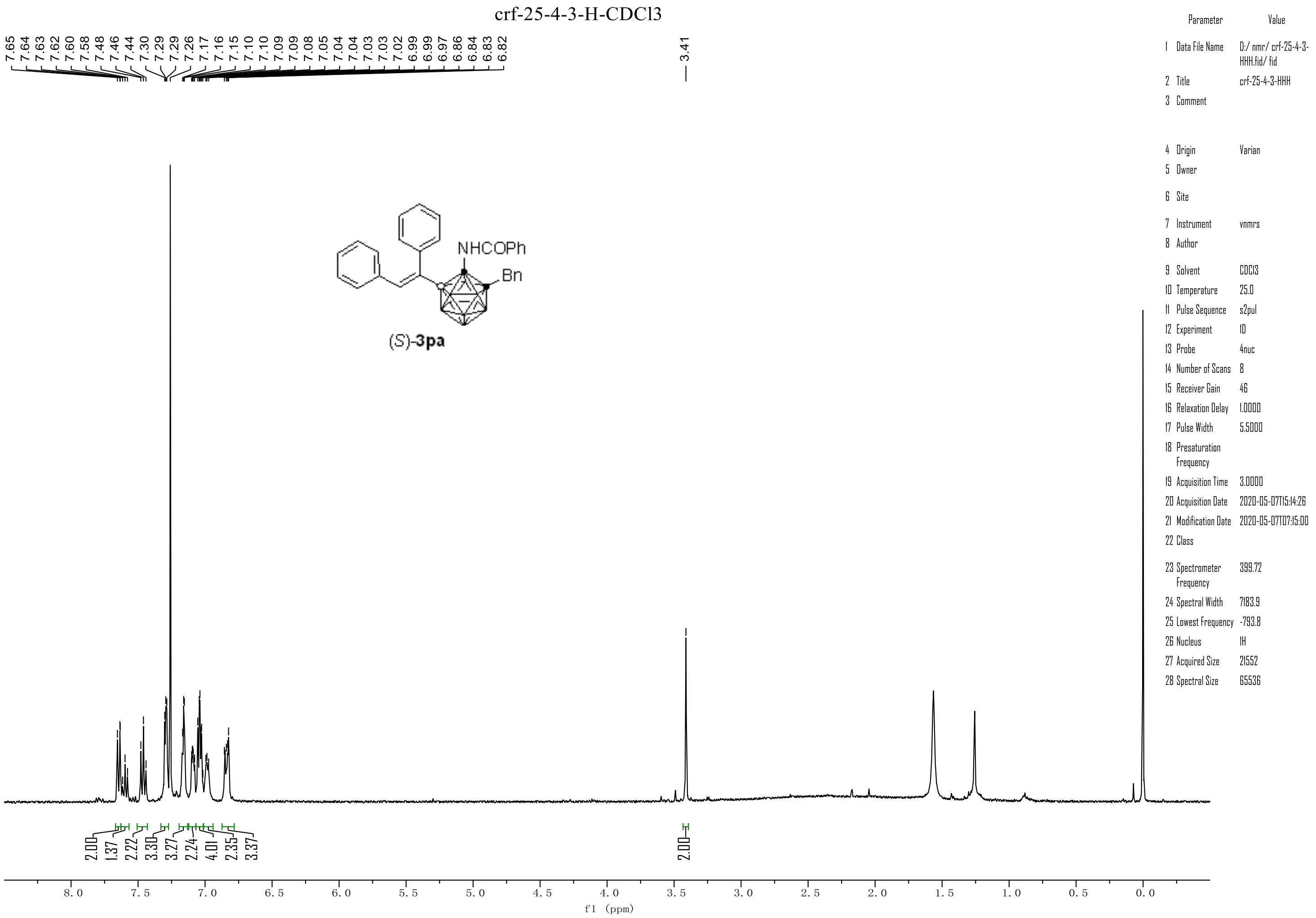

Supplementary Figure 215. $^{13}C\{^1H\}$ NMR of (*S*)-**3pa**.

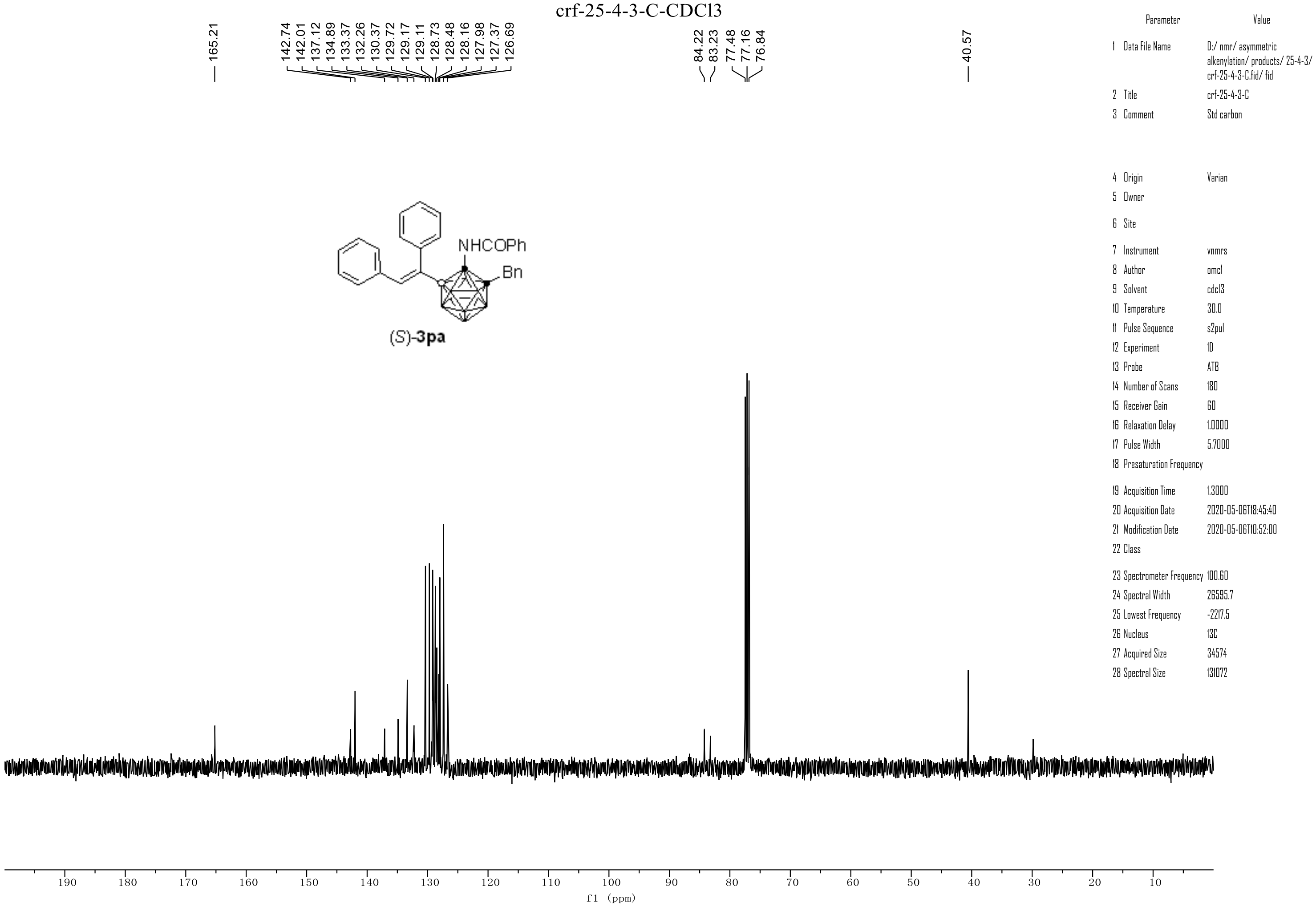

Parameter	Value
1 Data File Name	D:/ nmr/ asymmetric alkenylation/ products/ 25-4-3/ crf-25-4-3-C.fid/ fid
2 Title	crf-25-4-3-C
3 Comment	Std carbon
4 Origin	Varian
5 Owner	
6 Site	
7 Instrument	vnmrs
8 Author	omcl
9 Solvent	cdcl3
10 Temperature	30.0
11 Pulse Sequence	s2pul
12 Experiment	1D
13 Probe	ATB
14 Number of Scans	180
15 Receiver Gain	60
16 Relaxation Delay	1.0000
17 Pulse Width	5.7000
18 Presaturation Frequency	
19 Acquisition Time	1.3000
20 Acquisition Date	2020-05-06T18:45:40
21 Modification Date	2020-05-06T10:52:00
22 Class	
23 Spectrometer Frequency	100.60
24 Spectral Width	26595.7
25 Lowest Frequency	-2217.5
26 Nucleus	13C
27 Acquired Size	34574
28 Spectral Size	131072

Supplementary Figure 216. $^{11}B\{^{1}H\}$ NMR of (*S*)-**3pa**.

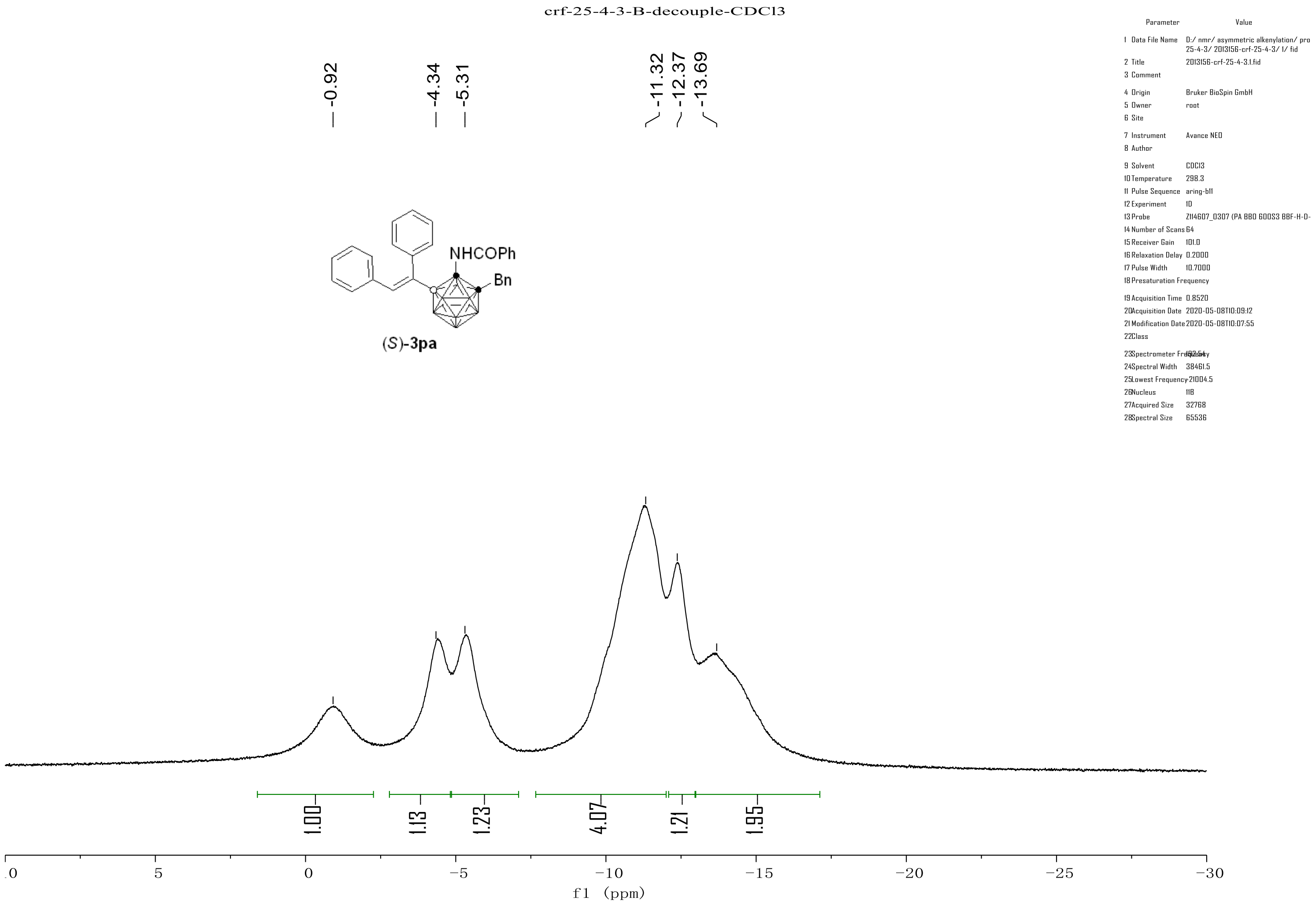

Supplementary Figure 217. ^{11}B NMR of (*S*)-**3pa**.

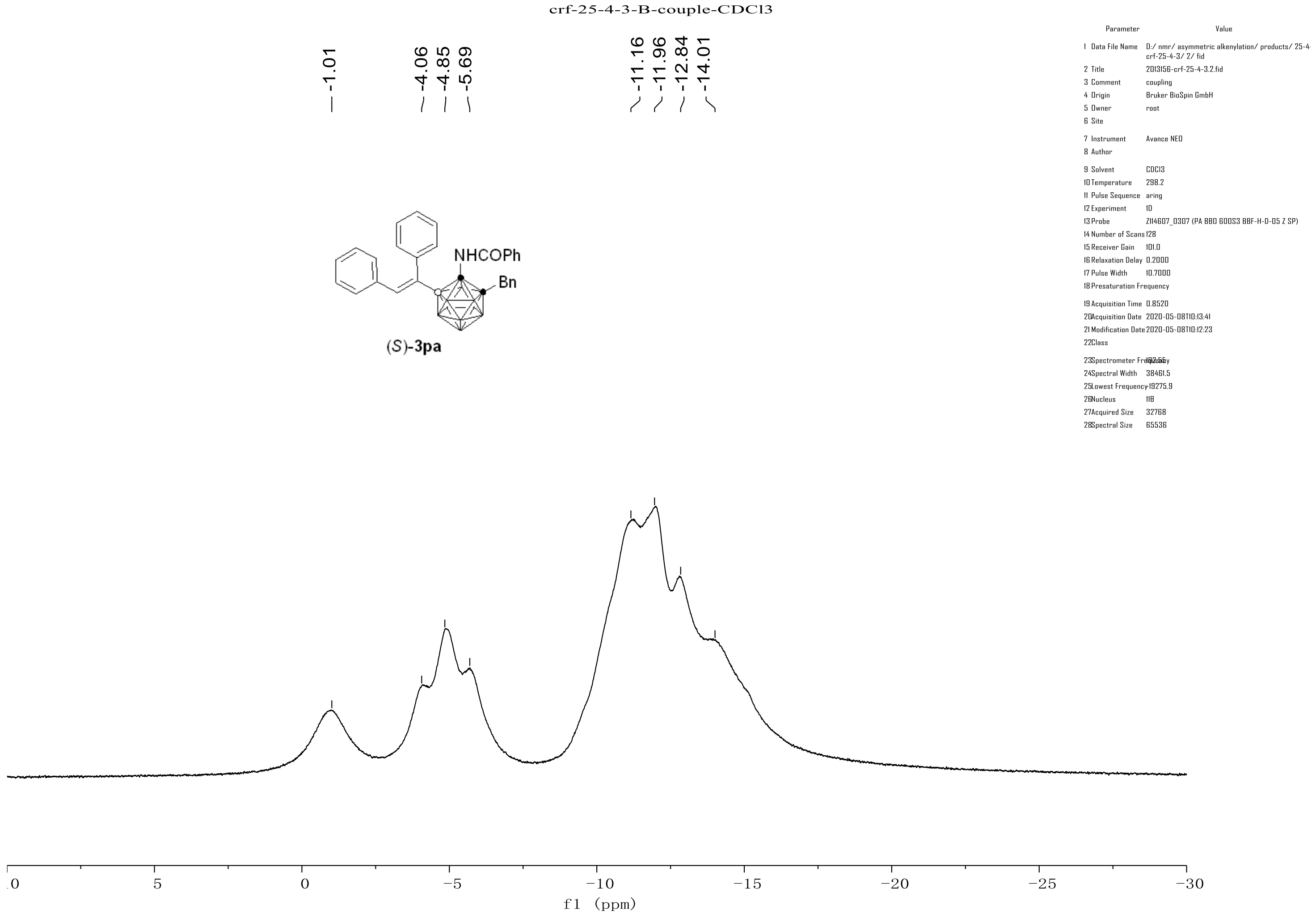

Supplementary Figure 218. ^{1}H NMR of (*S*)-**3qa**.

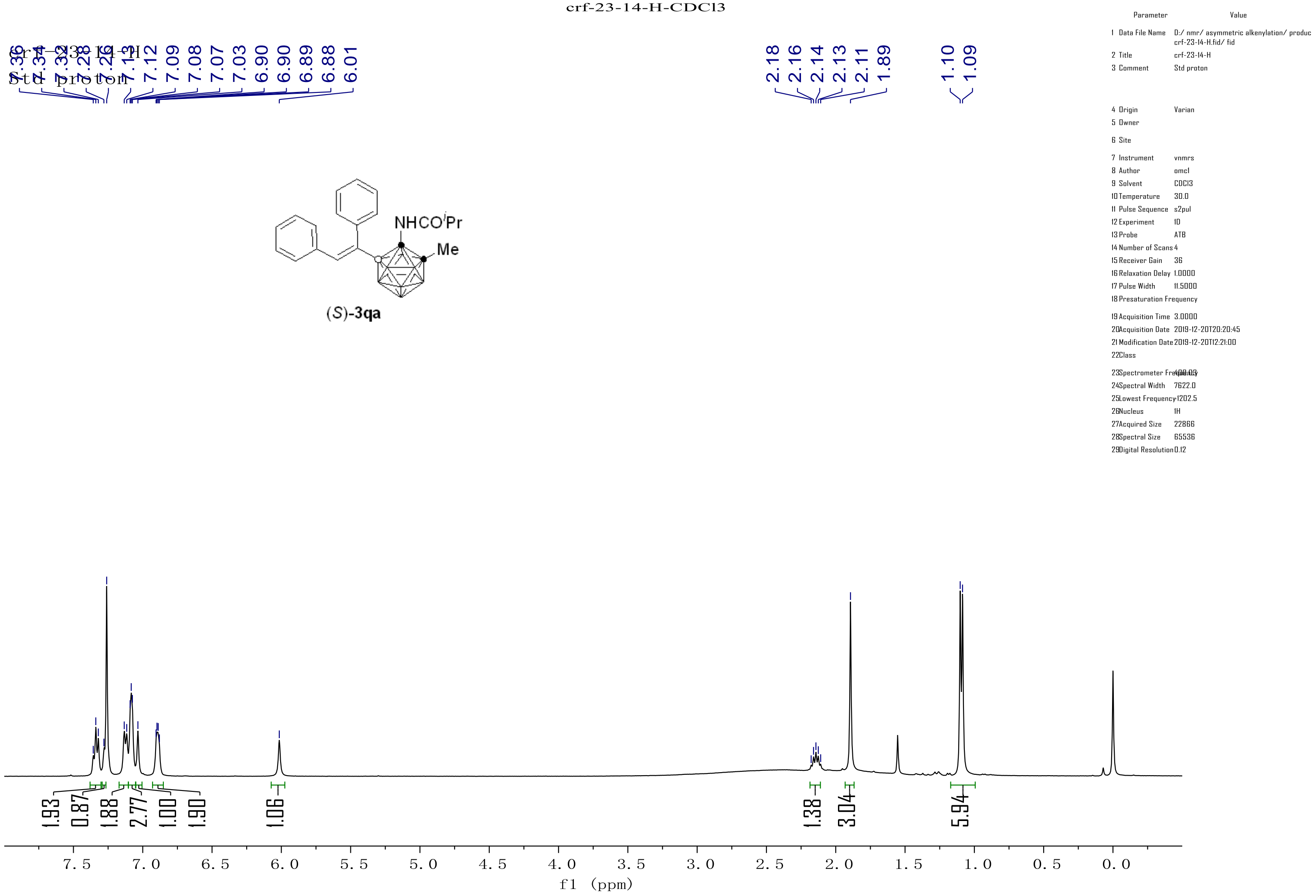

Supplementary Figure 219. $^{13}C\{^{1}H\}$ NMR of (*S*)-**3qa**.

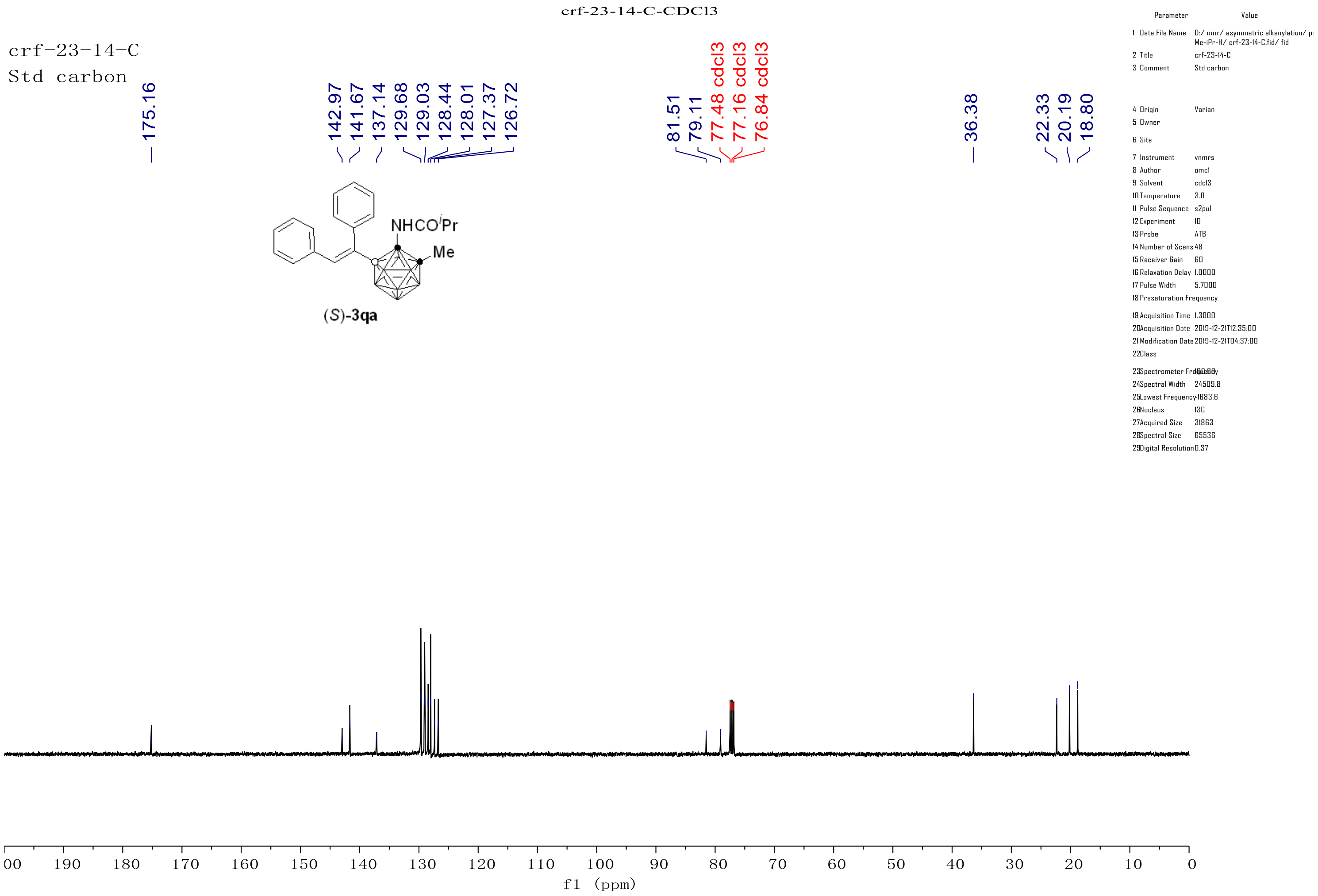

Supplementary Figure 220. $^{11}B\{^{1}H\}$ NMR of (*S*)-**3qa**.

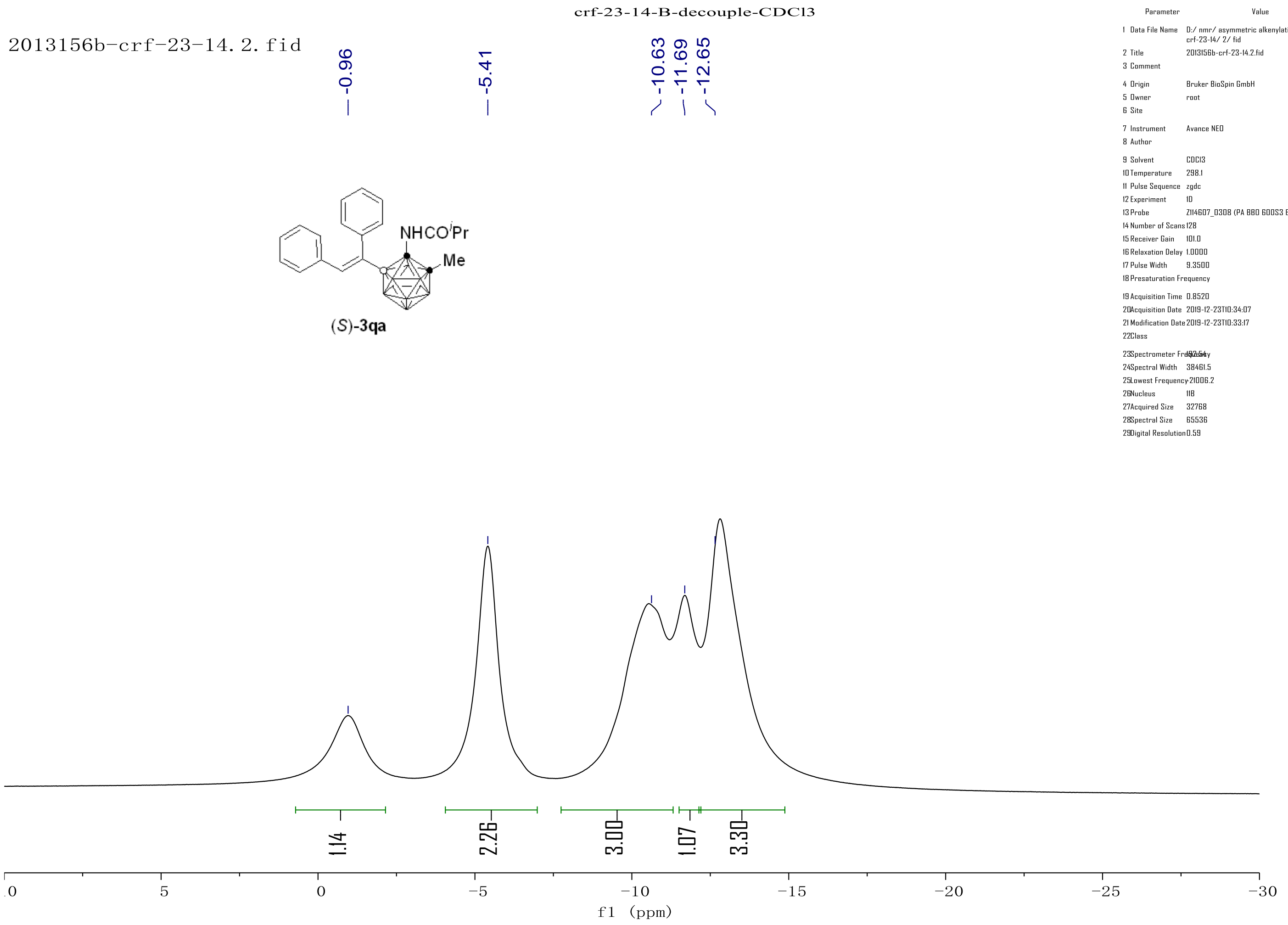

	Parameter	Value
1	Data File Name	D:/ nmr/ asymmetric alkenylation/ products/ M crf-23-14/ 2/ fid
2	Title	2013156b-crf-23-14.2.fid
3	Comment	
4	Origin	Bruker BioSpin GmbH
5	Owner	root
6	Site	
7	Instrument	Avance NEO
8	Author	
9	Solvent	CDCl3
10	Temperature	298.1
11	Pulse Sequence	zgdc
12	Experiment	1D
13	Probe	Z114607_0308 (PA BBO 600S3 BBF-H-D-05 Z SP)
14	Number of Scans	128
15	Receiver Gain	101.0
16	Relaxation Delay	1.0000
17	Pulse Width	9.3500
18	Presaturation Frequency	
19	Acquisition Time	0.8520
20	Acquisition Date	2019-12-23T10:34:07
21	Modification Date	2019-12-23T10:33:17
22	Class	
23	Spectrometer Frequency	192.54
24	Spectral Width	38461.5
25	Lowest Frequency	-21006.2
26	Nucleus	11B
27	Acquired Size	32768
28	Spectral Size	65536
29	Digital Resolution	0.59

Supplementary Figure 221. ^{11}B NMR of (*S*)-**3qa**.

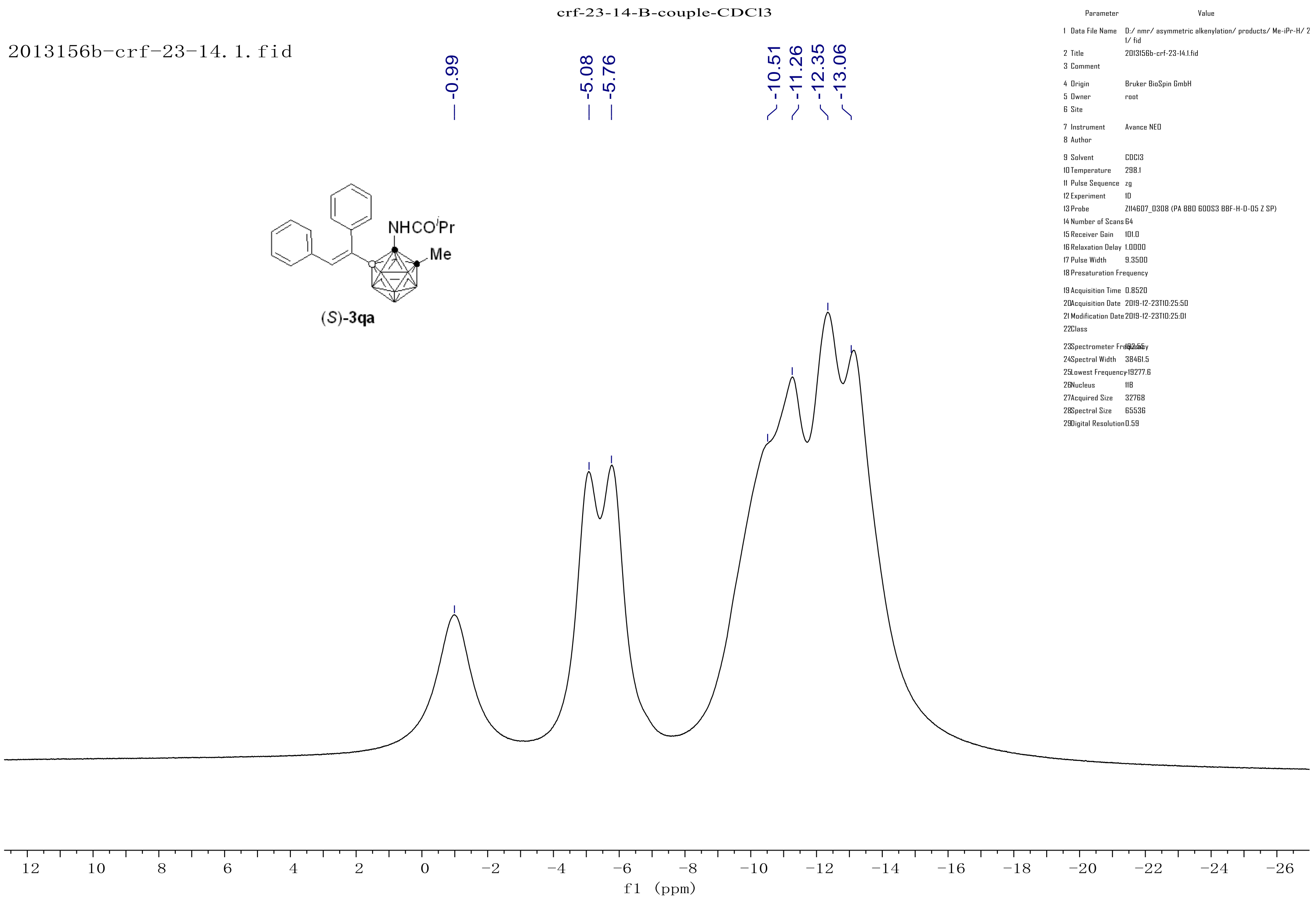

Supplementary Figure 222. ^{1}H NMR of (*S*)-**3ra**.

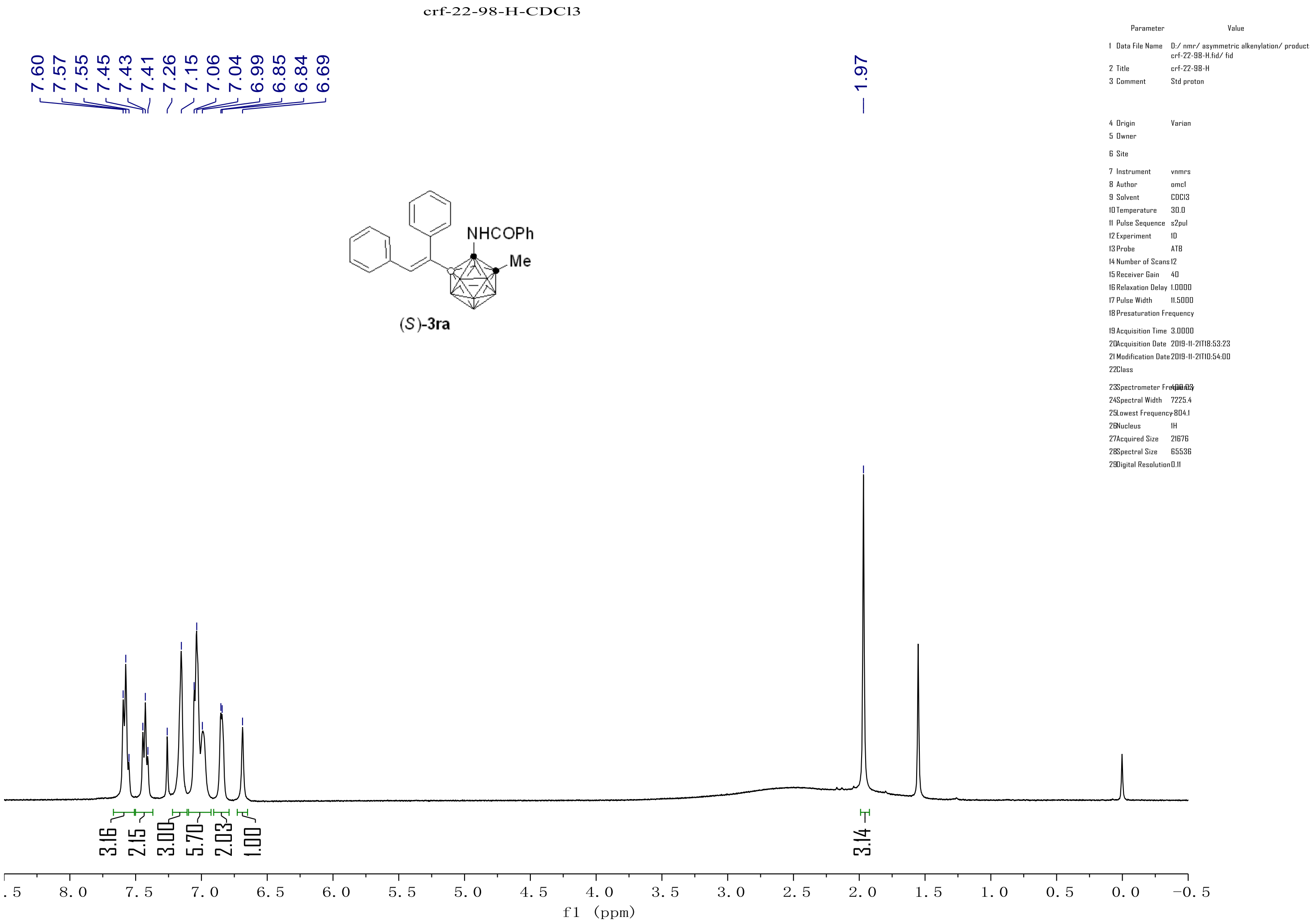

Supplementary Figure 223. $^{13}C\{^{1}H\}$ NMR of (*S*)-**3ra**.

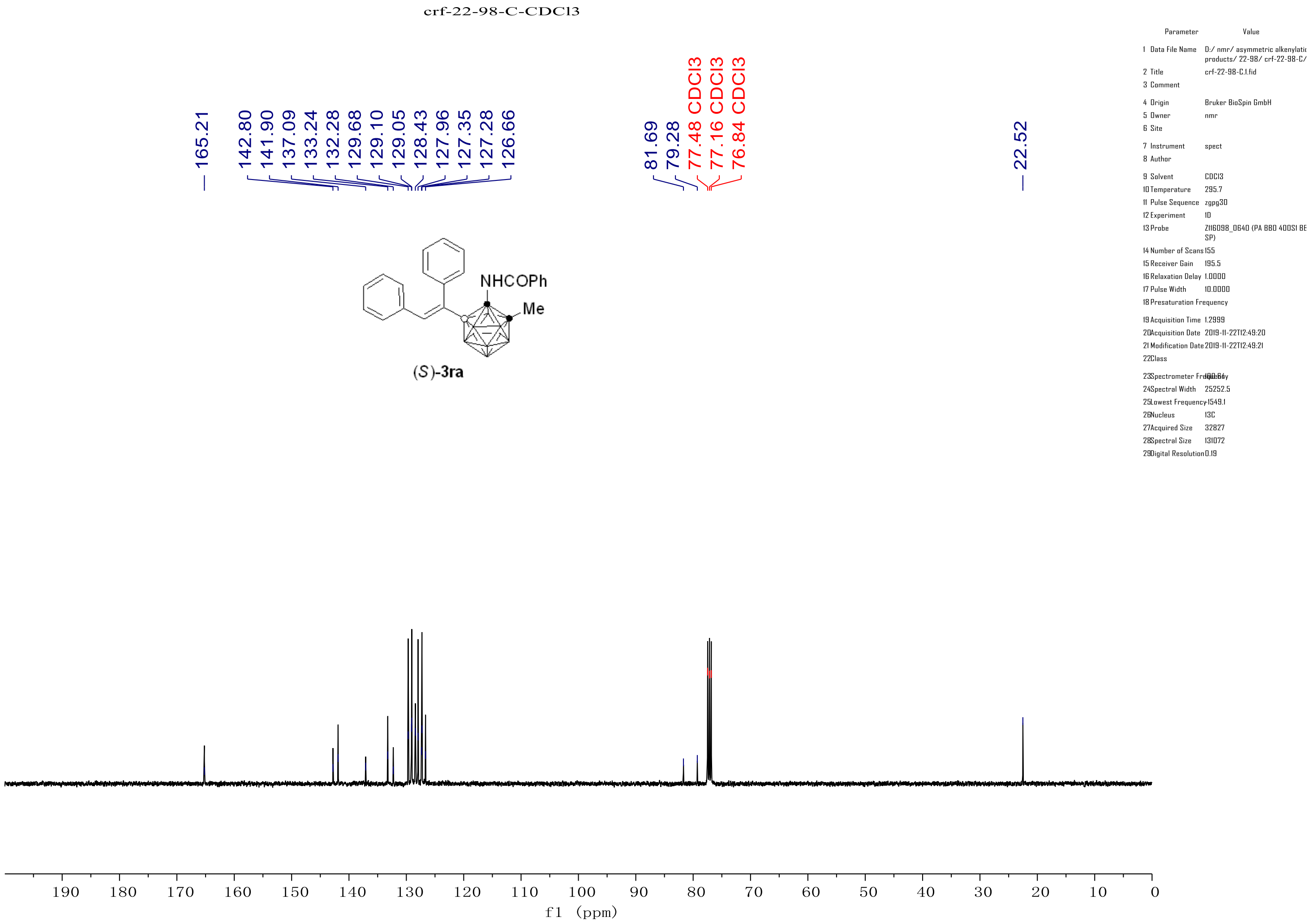

Supplementary Figure 224. $^{11}B\{^{1}H\}$ NMR of (*S*)-**3ra**.

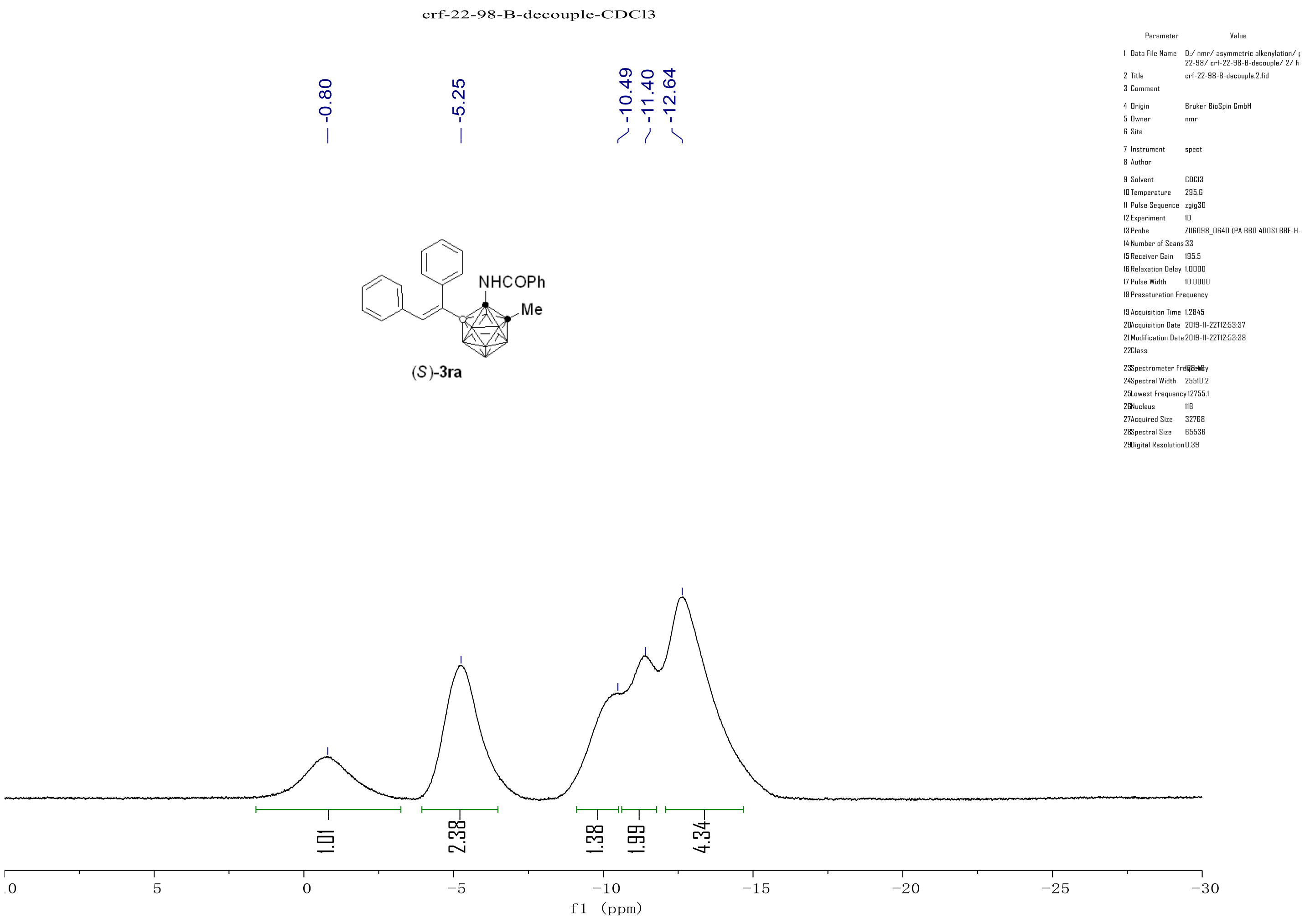

	Parameter	Value
1	Data File Name	D:/ nmr/ asymmetric alkenylation/ [illegible] 22-98/ crf-22-98-B-decouple/ 2/ fi[illegible]
2	Title	crf-22-98-B-decouple.2.fid
3	Comment	
4	Origin	Bruker BioSpin GmbH
5	Owner	nmr
6	Site	
7	Instrument	spect
8	Author	
9	Solvent	CDCl3
10	Temperature	295.6
11	Pulse Sequence	zgig30
12	Experiment	1D
13	Probe	Z116098_0640 (PA BBO 400S1 BBF-H-
14	Number of Scans	33
15	Receiver Gain	195.5
16	Relaxation Delay	1.0000
17	Pulse Width	10.0000
18	Presaturation Frequency	
19	Acquisition Time	1.2845
20	Acquisition Date	2019-11-22T12:53:37
21	Modification Date	2019-11-22T12:53:38
22	Class	
23	Spectrometer Frequency	128.40
24	Spectral Width	25510.2
25	Lowest Frequency	-12755.1
26	Nucleus	11B
27	Acquired Size	32768
28	Spectral Size	65536
29	Digital Resolution	0.39

Supplementary Figure 225. ^{11}B NMR of (*S*)-**3ra**.

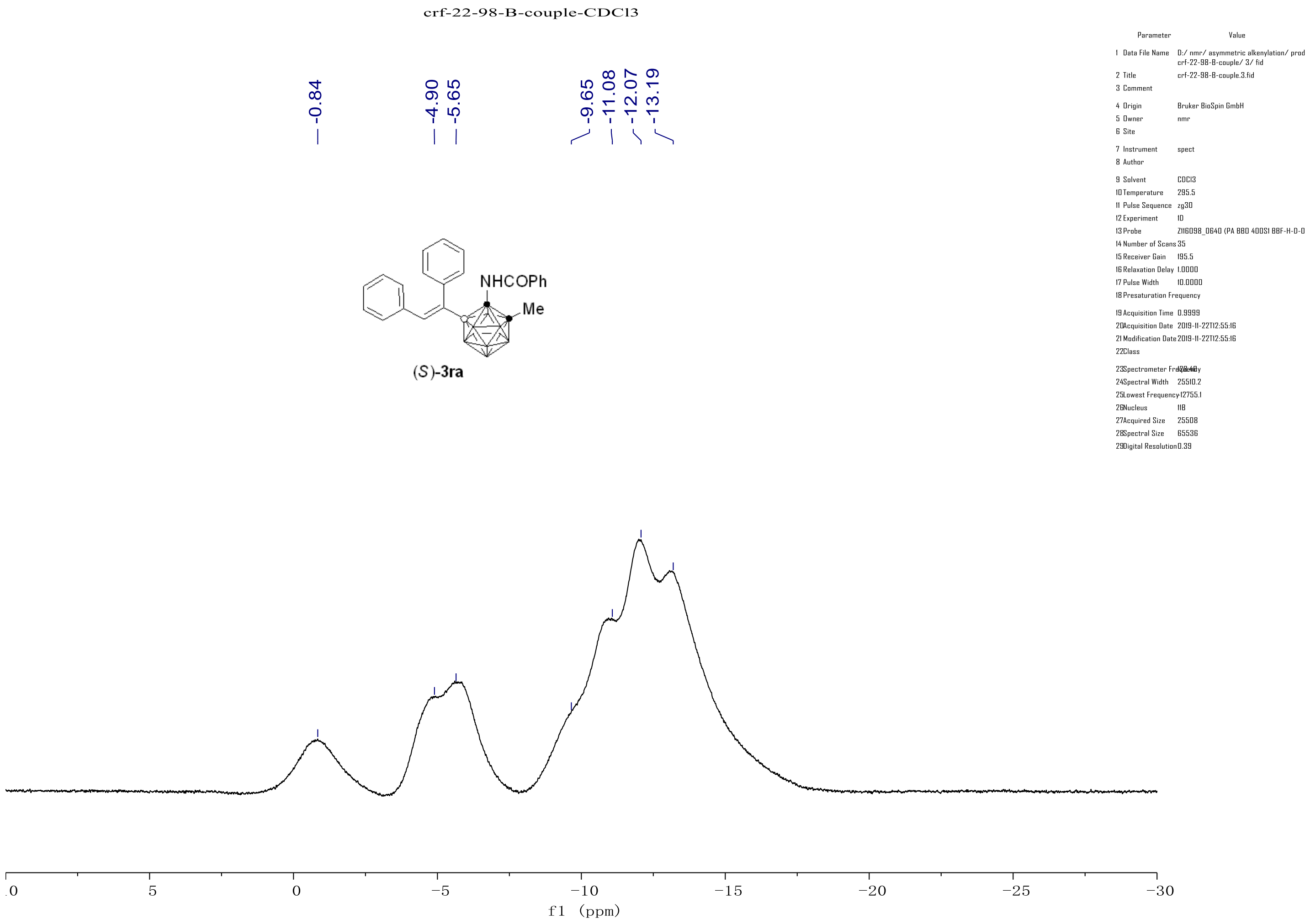

Parameter	Value
1 Data File Name	D:/ nmr/ asymmetric alkenylation/ prod crf-22-98-B-couple/ 3/ fid
2 Title	crf-22-98-B-couple.3.fid
3 Comment	
4 Origin	Bruker BioSpin GmbH
5 Owner	nmr
6 Site	
7 Instrument	spect
8 Author	
9 Solvent	CDCl3
10 Temperature	295.5
11 Pulse Sequence	zg30
12 Experiment	1D
13 Probe	Z116098_0640 (PA BBO 400S1 BBF-H-D-0
14 Number of Scans	35
15 Receiver Gain	195.5
16 Relaxation Delay	1.0000
17 Pulse Width	10.0000
18 Presaturation Frequency	
19 Acquisition Time	0.9999
20 Acquisition Date	2019-11-22T12:55:16
21 Modification Date	2019-11-22T12:55:16
22 Class	
23 Spectrometer Frequency	128.40
24 Spectral Width	25510.2
25 Lowest Frequency	-12755.1
26 Nucleus	11B
27 Acquired Size	25508
28 Spectral Size	65536
29 Digital Resolution	0.39

Supplementary Figure 226. ^{1}H NMR of (*S*)-**4ba**.

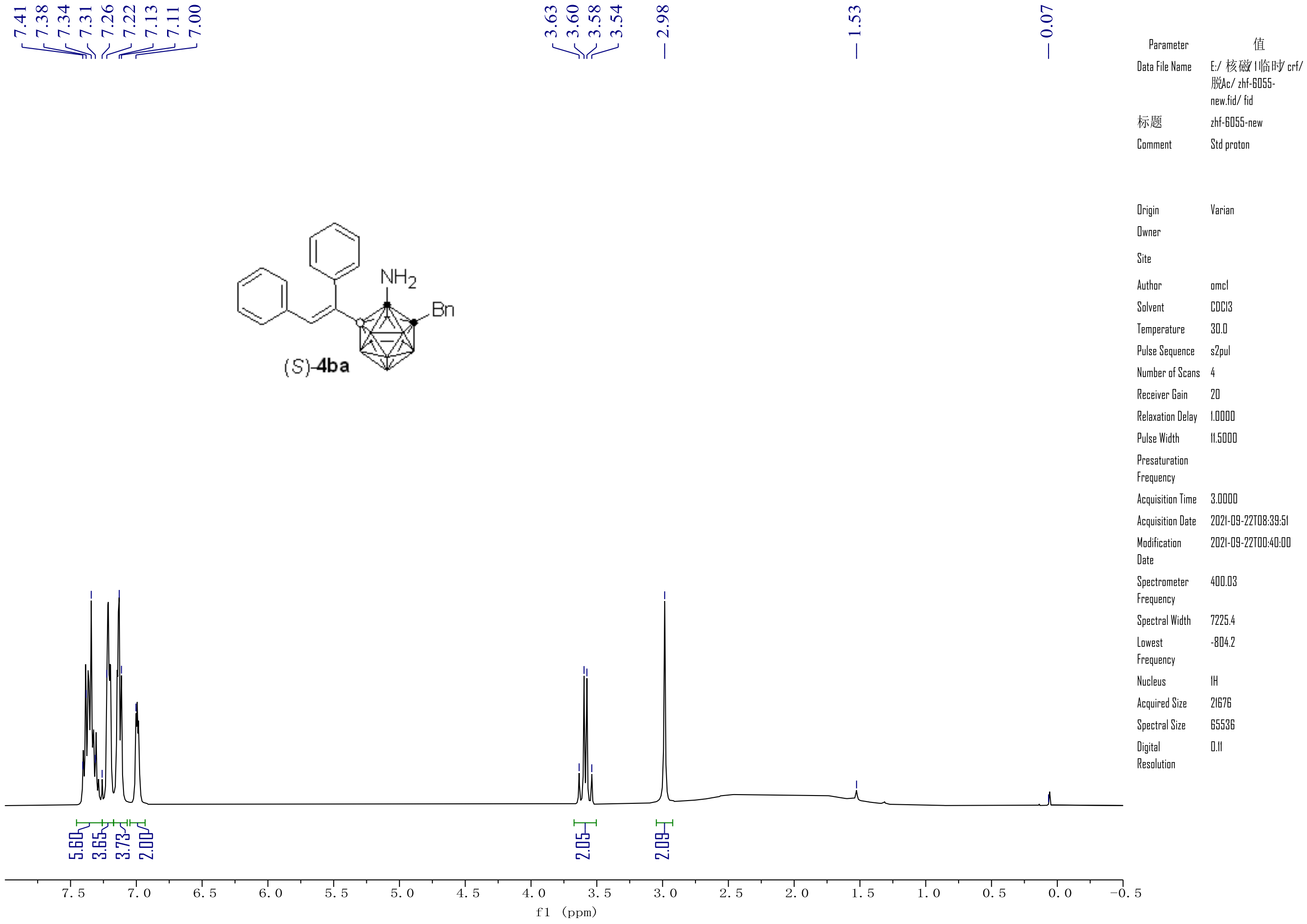

Parameter	值
Data File Name	E:/ 核磁 \临时 crf/ 脱Ac/ zhf-6055-new.fid/ fid
标题	zhf-6055-new
Comment	Std proton
Origin	Varian
Owner	
Site	
Author	omcl
Solvent	CDCl3
Temperature	30.0
Pulse Sequence	s2pul
Number of Scans	4
Receiver Gain	20
Relaxation Delay	1.0000
Pulse Width	11.5000
Presaturation Frequency	
Acquisition Time	3.0000
Acquisition Date	2021-09-22T08:39:51
Modification Date	2021-09-22T00:40:00
Spectrometer Frequency	400.03
Spectral Width	7225.4
Lowest Frequency	-804.2
Nucleus	1H
Acquired Size	21676
Spectral Size	65536
Digital Resolution	0.11

Supplementary Figure 227. $^{13}C\{^{1}H\}$ NMR of (*S*)-**4ba**.

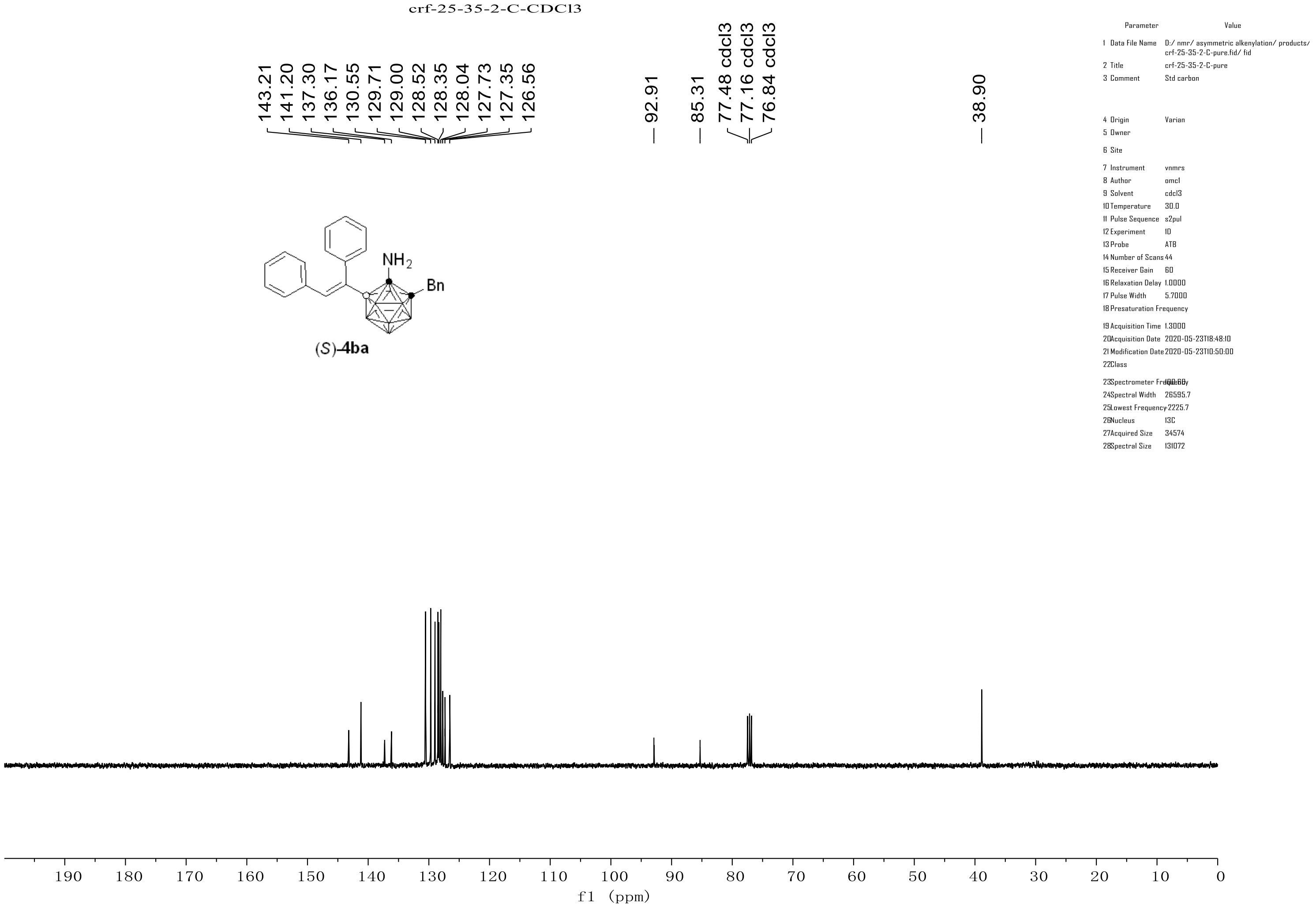

Supplementary Figure 228. $^{11}B\{^{1}H\}$ NMR of (*S*)-**4ba**.

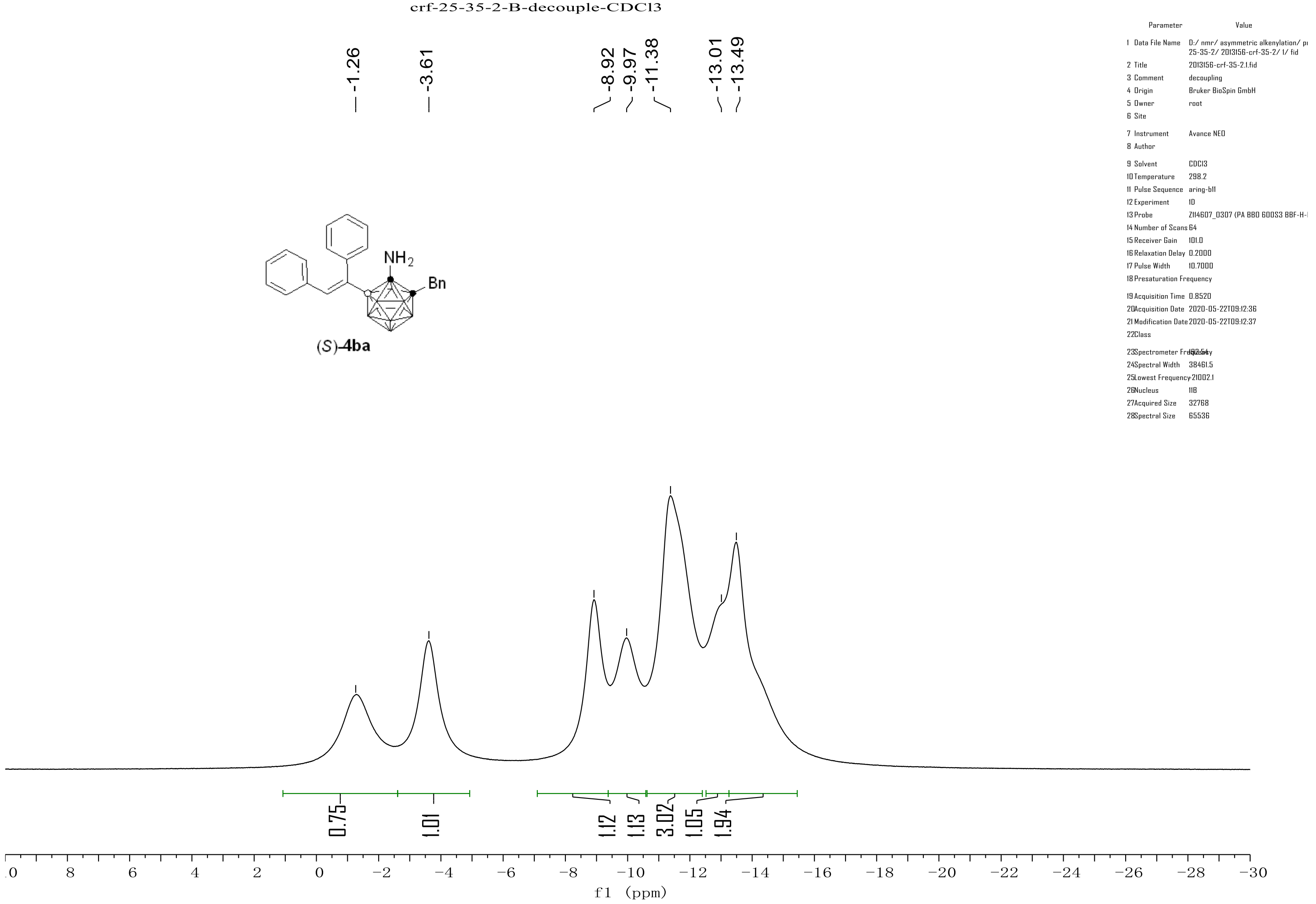

Supplementary Figure 229. ^{11}B NMR of (*S*)-**4ba**.

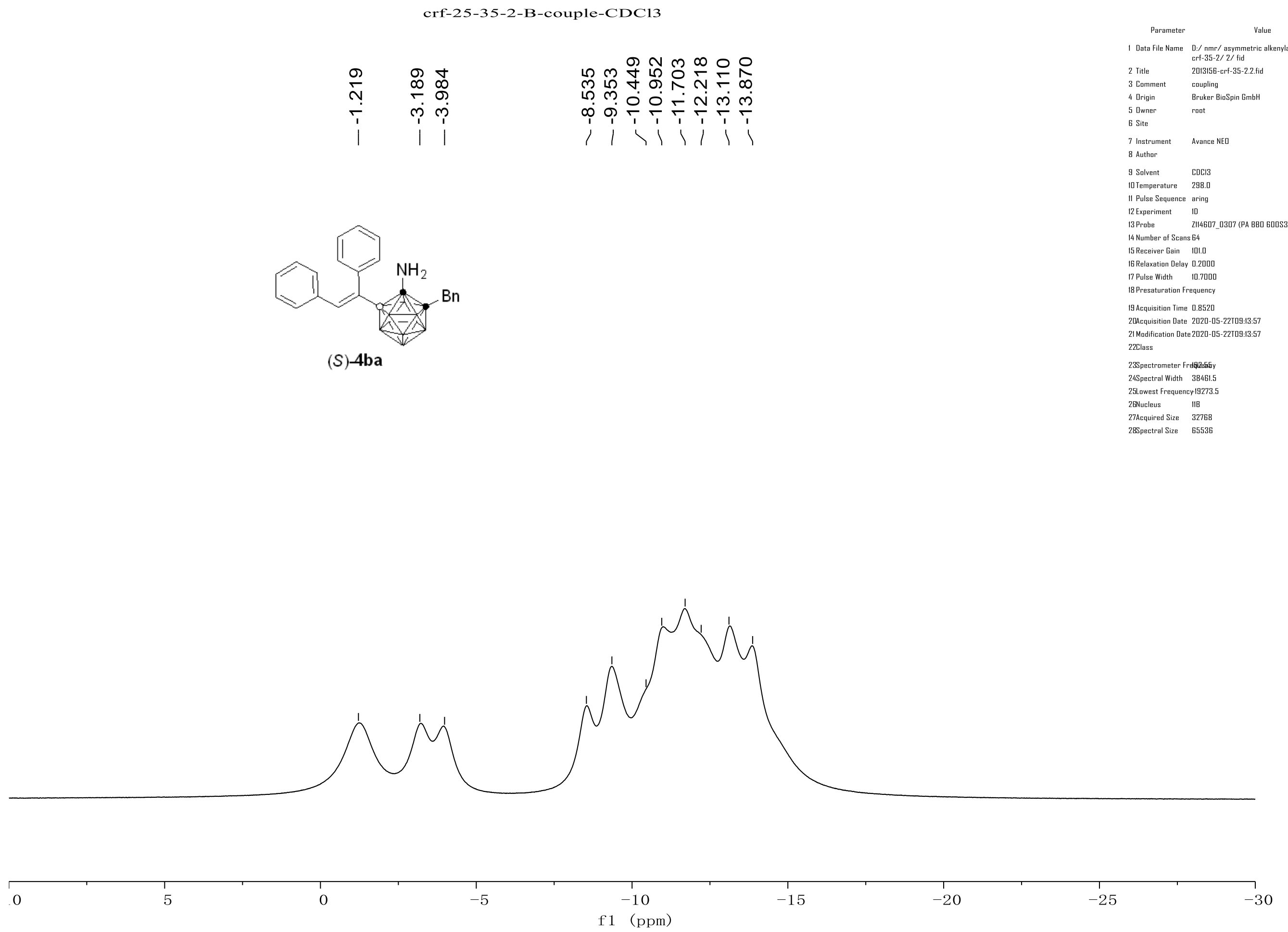

	Parameter	Value
1	Data File Name	D:/ nmr/ asymmetric alkenylation/ products/ crf-35-2/ 2/ fid
2	Title	2013156-crf-35-2.2.fid
3	Comment	coupling
4	Origin	Bruker BioSpin GmbH
5	Owner	root
6	Site	
7	Instrument	Avance NEO
8	Author	
9	Solvent	CDCl3
10	Temperature	298.0
11	Pulse Sequence	aring
12	Experiment	1D
13	Probe	Z114607_0307 (PA BBO 600S3 BBF-H-D-05 Z SP
14	Number of Scans	64
15	Receiver Gain	101.0
16	Relaxation Delay	0.2000
17	Pulse Width	10.7000
18	Presaturation Frequency	
19	Acquisition Time	0.8520
20	Acquisition Date	2020-05-22T09:13:57
21	Modification Date	2020-05-22T09:13:57
22	Class	
23	Spectrometer Frequency	192.55
24	Spectral Width	38461.5
25	Lowest Frequency	-19273.5
26	Nucleus	11B
27	Acquired Size	32768
28	Spectral Size	65536

Supplementary Figure 230. Chiral HPLC analysis of (*rac*)-**3ba**.

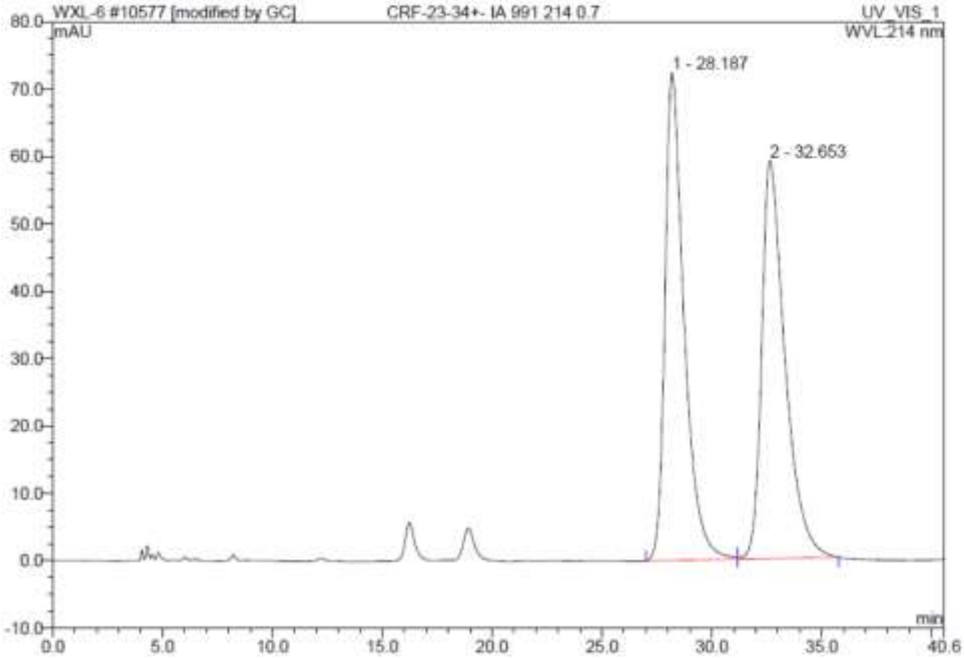

No.	Ret.Time min	Peak Name	Height mAU	Area mAU*min	Rel.Area %	Amount	Type
1	28.19	n.a.	72.391	73.053	50.13	n.a.	BM
2	32.65	n.a.	59.095	72.662	49.87	n.a.	MB
Total:			131.486	145.715	100.00	0.000	

Supplementary Figure 231. Chiral HPLC analysis of (*S*)-**3ba** from asymmetric reaction (Method **A**).

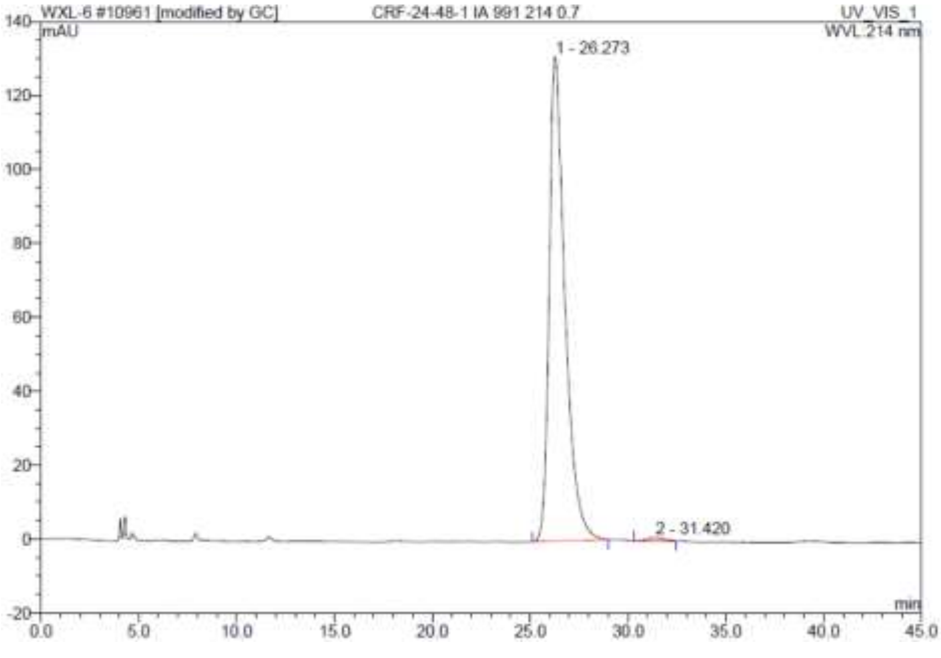

No.	Ret.Time min	Peak Name	Height mAU	Area mAU*min	Rel.Area %	Amount	Type
1	26.27	n.a.	131.077	122.104	99.40	n.a.	BMB
2	31.42	n.a.	0.835	0.736	0.60	n.a.	BMB*
Total:			131.912	122.840	100.00	0.000	

Supplementary Figure 232. Chiral HPLC analysis of (*R*)-**3ba** from asymmetric reaction (Method **A**).

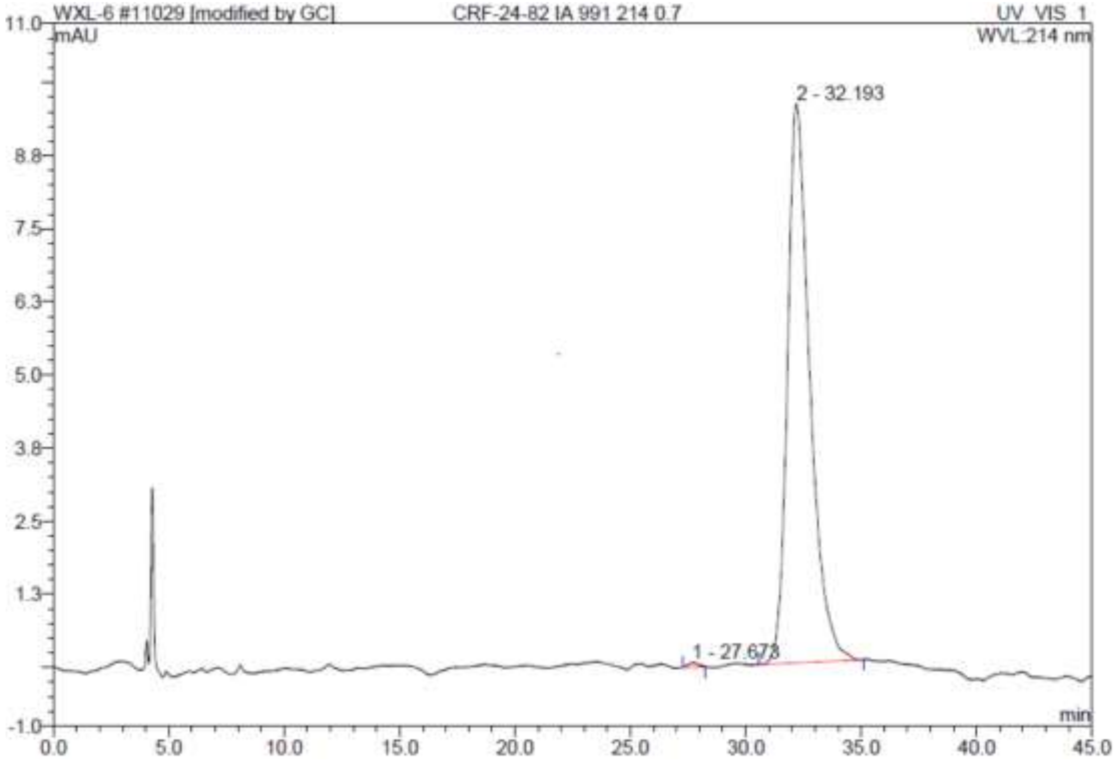

No.	Ret.Time min	Peak Name	Height mAU	Area mAU*min	Rel.Area %	Amount	Type
1	27.67	n.a.	0.086	0.045	0.42	n.a.	BMB*
2	32.19	n.a.	9.558	10.788	99.58	n.a.	BMB*
Total:			9.644	10.833	100.00	0.000	

Supplementary Figure 233. Chiral HPLC analysis of (*rac*)-**3bb**.

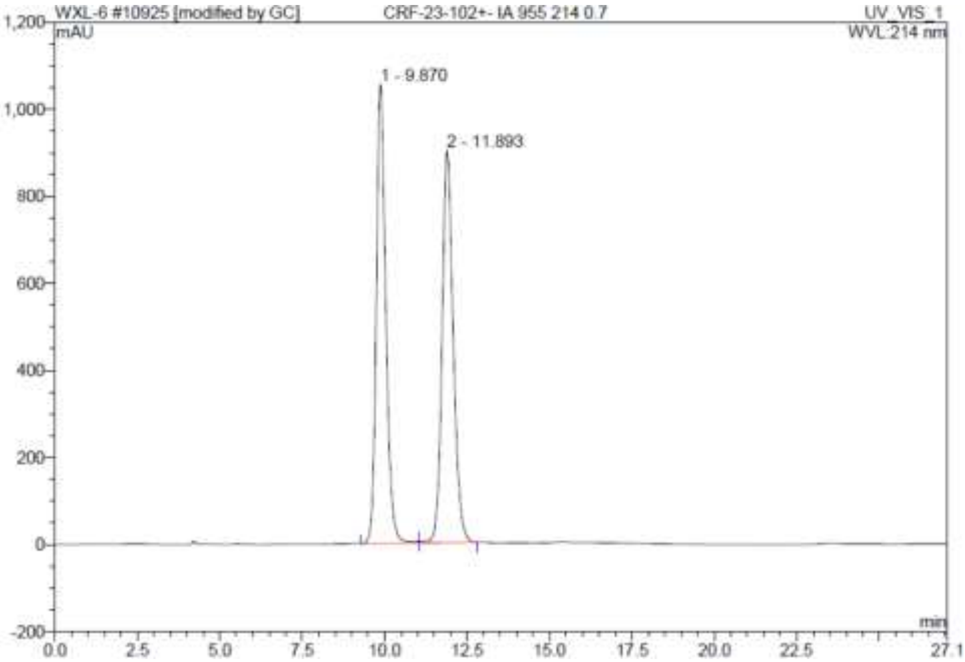

No.	Ret.Time min	Peak Name	Height mAU	Area mAU*min	Rel.Area %	Amount	Type
1	9.87	n.a.	1054.899	352.346	49.88	n.a.	BM
2	11.89	n.a.	900.692	354.097	50.12	n.a.	MB
Total:			1955.592	706.443	100.00	0.000	

Supplementary Figure 234. Chiral HPLC analysis of (*S*)-**3bb** from asymmetric reaction (Method **A**).

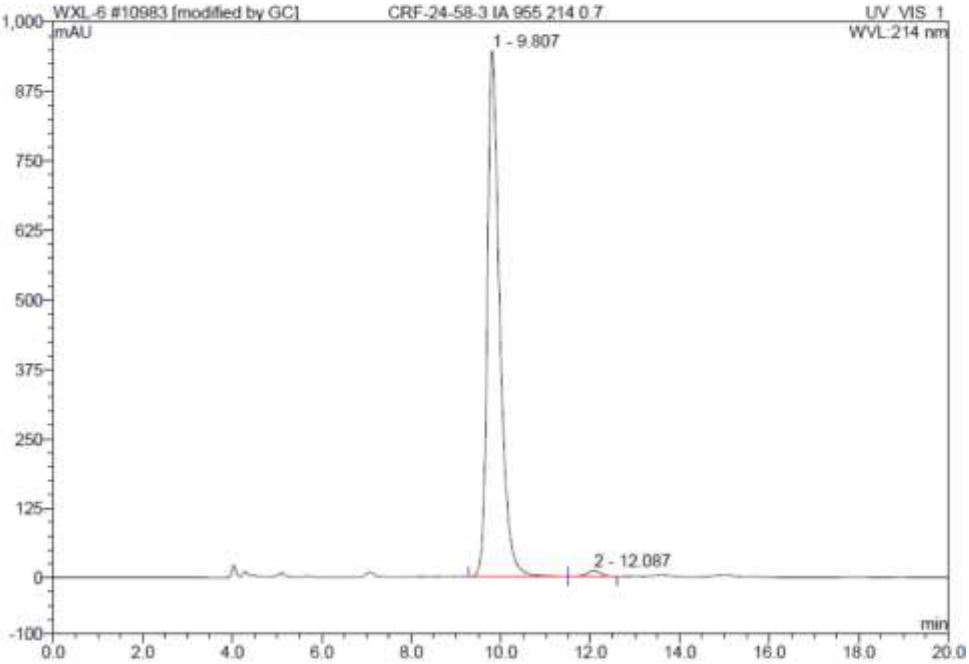

No.	Ret.Time min	Peak Name	Height mAU	Area mAU*min	Rel.Area %	Amount	Type
1	9.81	n.a.	946.568	305.494	98.61	n.a.	BM
2	12.09	n.a.	11.134	4.317	1.39	n.a.	MB
Total:			957.702	309.811	100.00	0.000	

Supplementary Figure 235. Chiral HPLC analysis of (*rac*)-**3bc**.

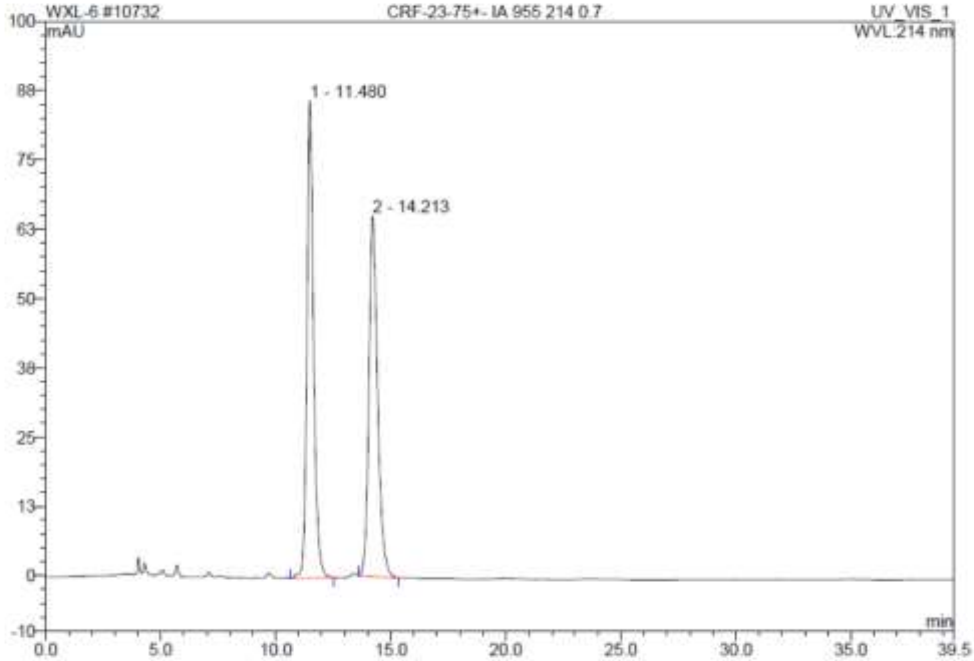

No.	Ret.Time min	Peak Name	Height mAU	Area mAU*min	Rel.Area %	Amount	Type
1	11.48	n.a.	86.096	29.131	50.59	n.a.	BMB
2	14.21	n.a.	65.001	28.447	49.41	n.a.	BMB
Total:			151.097	57.578	100.00	0.000	

Supplementary Figure 236. Chiral HPLC analysis of (*S*)-**3bc** from asymmetric reaction (Method **A**).

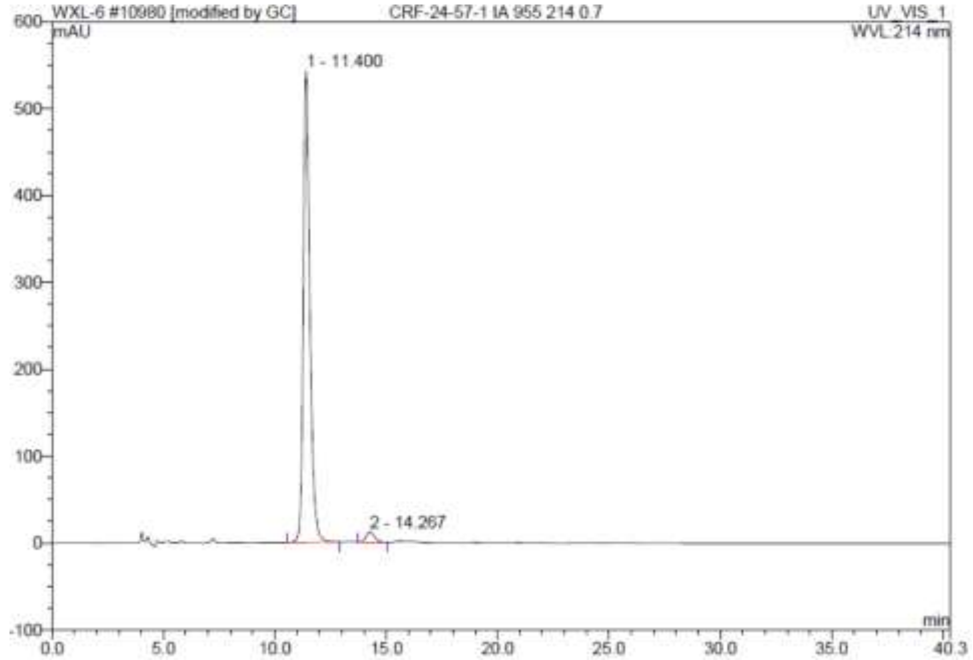

No.	Ret.Time min	Peak Name	Height mAU	Area mAU*min	Rel.Area %	Amount	Type
1	11.40	n.a.	542.604	185.002	97.31	n.a.	BMB
2	14.27	n.a.	11.928	5.116	2.69	n.a.	BMB
Total:			554.533	190.118	100.00	0.000	

Supplementary Figure 237. Chiral HPLC analysis of (*rac*)-**3bd**.

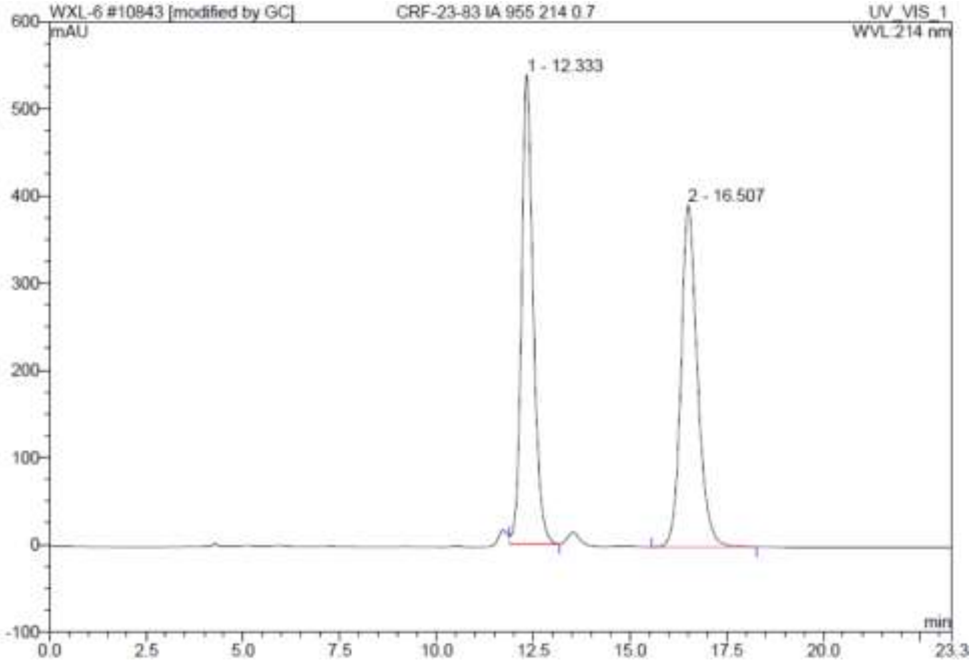

No.	Ret.Time min	Peak Name	Height mAU	Area mAU*min	Rel.Area %	Amount	Type
1	12.33	n.a.	538.587	188.421	49.32	n.a.	MB*
2	16.51	n.a.	392.278	193.585	50.68	n.a.	BMB
Total:			930.866	382.006	100.00	0.000	

Supplementary Figure 238. Chiral HPLC analysis of (*S*)-**3bd** from asymmetric reaction (Method **A**).

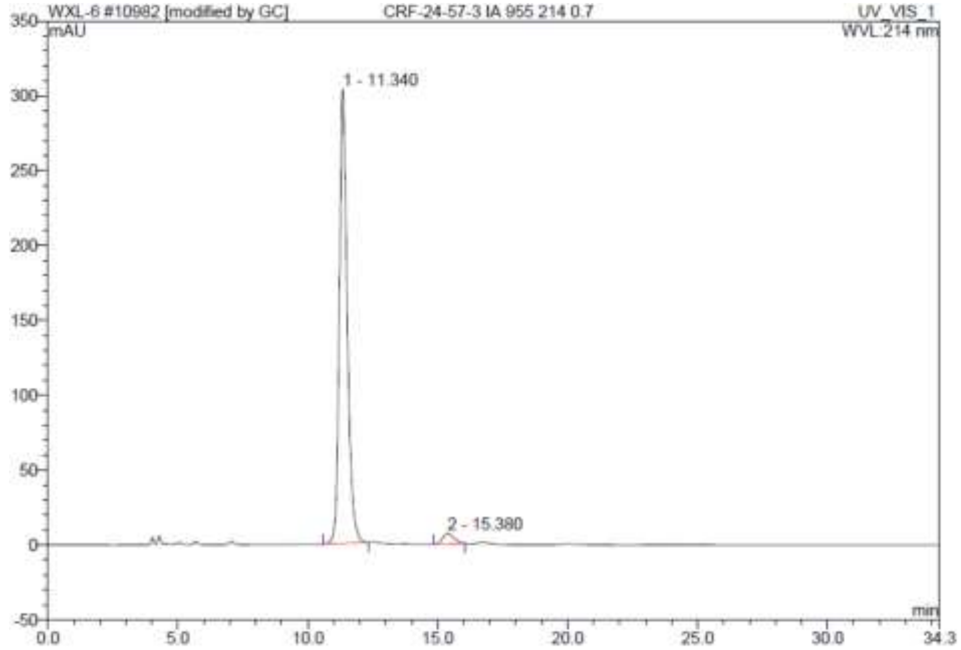

No.	Ret.Time min	Peak Name	Height mAU	Area mAU*min	Rel.Area %	Amount	Type
1	11.34	n.a.	303.511	106.990	96.76	n.a.	BMB
2	15.38	n.a.	7.123	3.587	3.24	n.a.	BMB*
Total:			310.634	110.578	100.00	0.000	

Supplementary Figure 239. Chiral HPLC analysis of (*rac*)-**3be**.

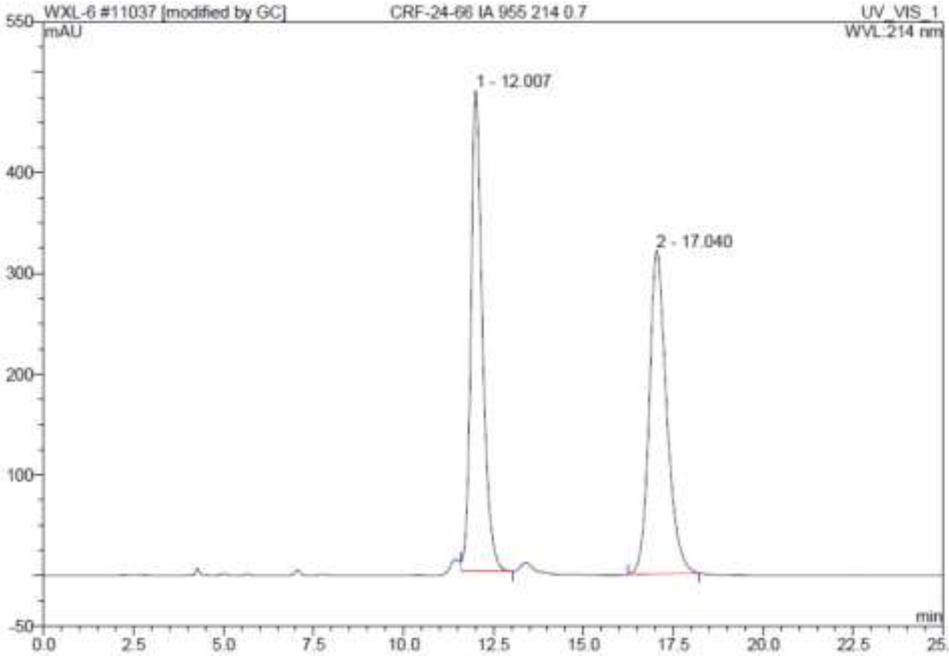

No.	Ret.Time min	Peak Name	Height mAU	Area mAU*min	Rel.Area %	Amount	Type
1	12.01	n.a.	477.470	178.620	49.70	n.a.	MB*
2	17.04	n.a.	321.203	180.782	50.30	n.a.	BMB*
Total:			798.672	359.402	100.00	0.000	

Supplementary Figure 240. Chiral HPLC analysis of (*S*)-**3be** from asymmetric reaction (Method **A**).

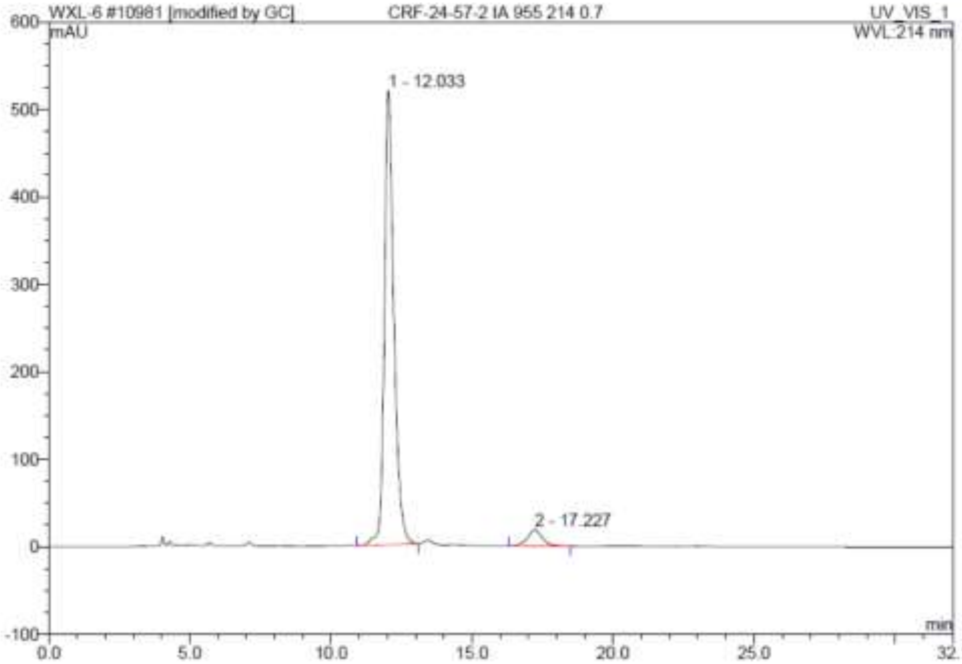

No.	Ret.Time min	Peak Name	Height mAU	Area mAU*min	Rel.Area %	Amount	Type
1	12.03	n.a.	519.983	203.054	94.68	n.a.	BMB*
2	17.23	n.a.	18.526	11.415	5.32	n.a.	BMB
Total:			538.509	214.469	100.00	0.000	

Supplementary Figure 241.Chiral HPLC analysis of (*rac*)-**3bf**.

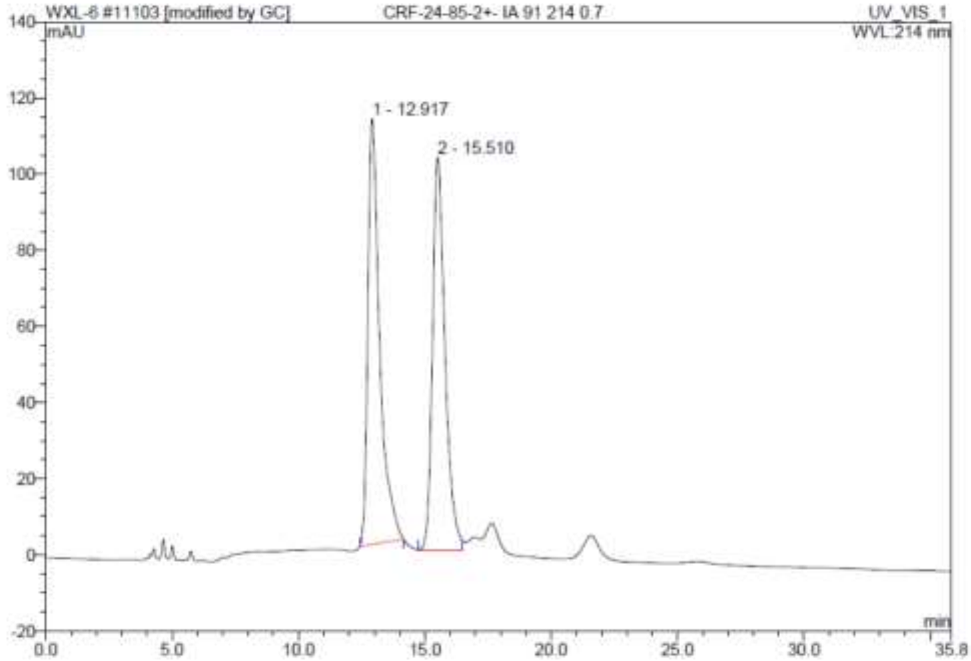

No.	Ret.Time min	Peak Name	Height mAU	Area mAU*min	Rel.Area %	Amount	Type
1	12.92	n.a.	111.951	60.546	50.68	n.a.	BMB*
2	15.51	n.a.	103.114	58.911	49.32	n.a.	BM *
Total:			215.065	119.458	100.00	0.000	

Supplementary Figure 242.Chiral HPLC analysis of (*S*)-**3bf** from asymmetric reaction (Method **A**).

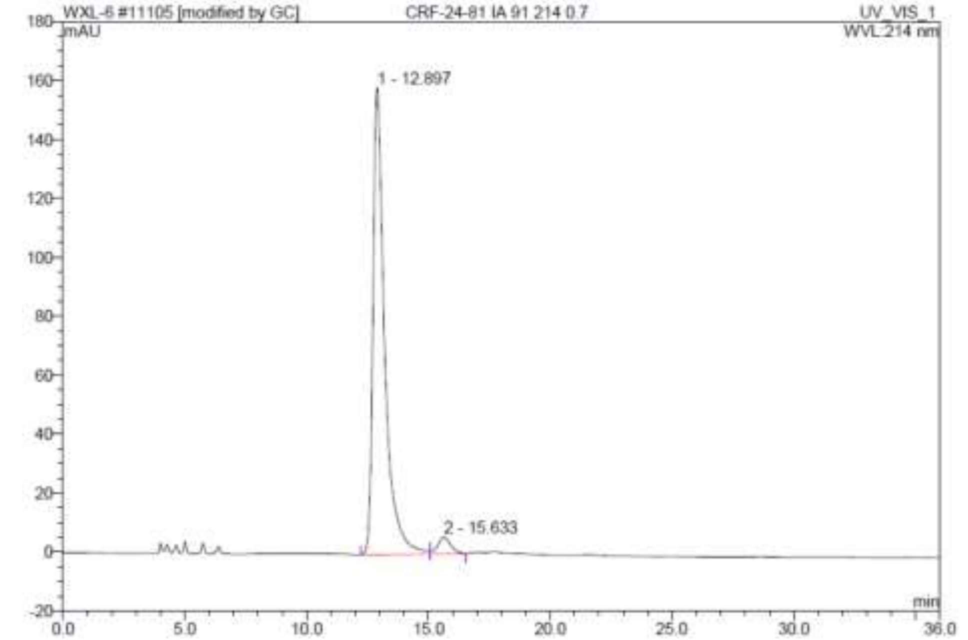

No.	Ret.Time min	Peak Name	Height mAU	Area mAU*min	Rel.Area %	Amount	Type
1	12.90	n.a.	158.611	88.186	96.17	n.a.	BM
2	15.63	n.a.	5.694	3.509	3.83	n.a.	MB
Total:			164.305	91.695	100.00	0.000	

Supplementary Figure 243. Chiral HPLC analysis of (*rac*)-**3bg**.

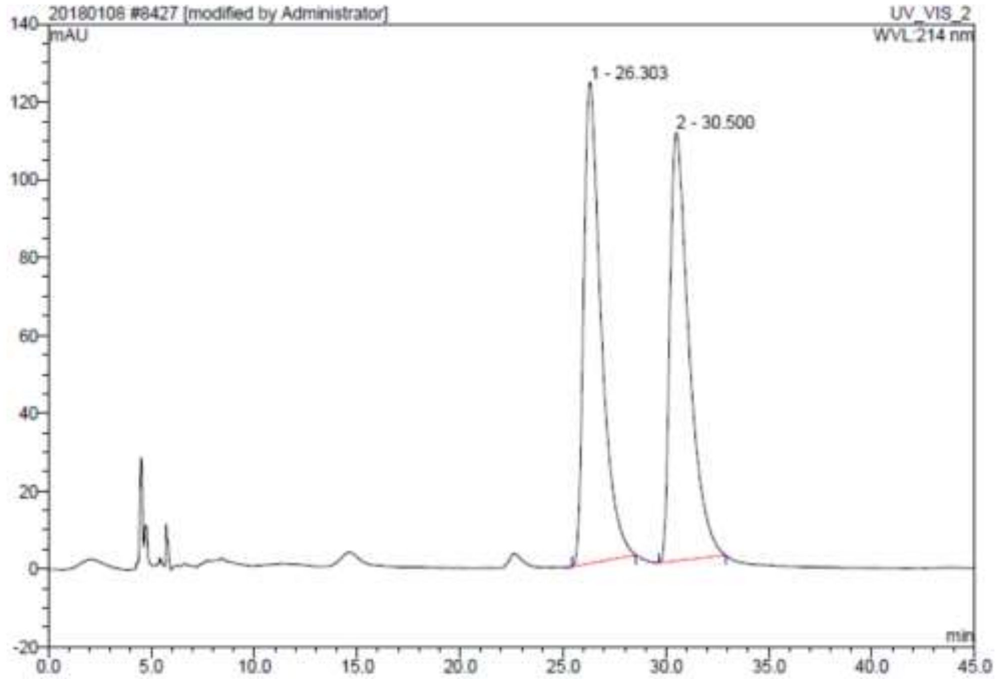

No.	Ret.Time min	Peak Name	Height mAU	Area mAU*min	Rel.Area %	Amount	Type
1	26.30	n.a.	123.604	121.764	50.67	n.a.	BMB
2	30.50	n.a.	110.079	118.566	49.33	n.a.	BMB
Total:			233.683	240.330	100.00	0.000	

Supplementary Figure 244. Chiral HPLC analysis of (*S*)-**3bg** from asymmetric reaction (Method **A**).

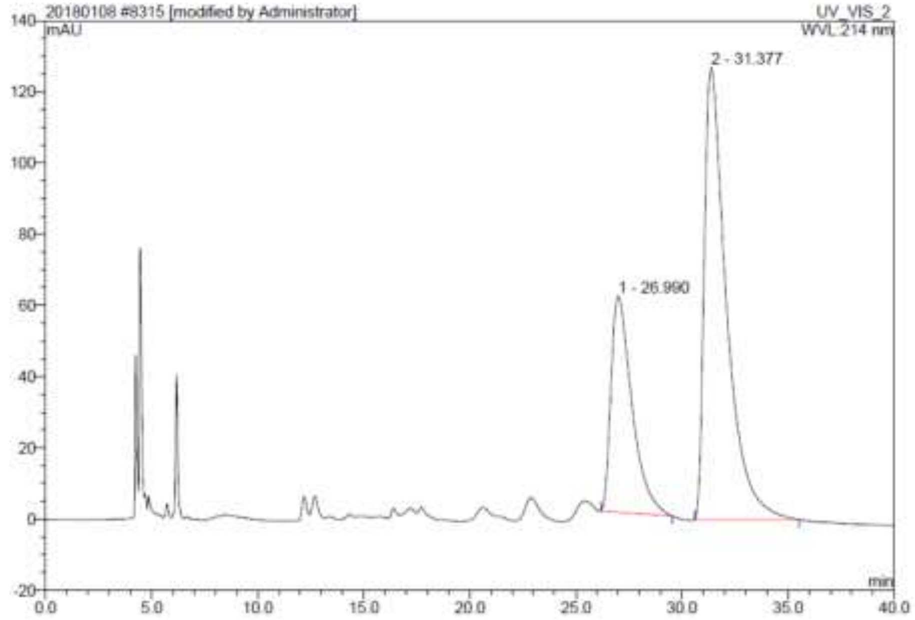

No.	Ret.Time min	Peak Name	Height mAU	Area mAU*min	Rel.Area %	Amount	Type
1	26.99	n.a.	60.695	69.467	31.09	n.a.	BMB*
2	31.38	n.a.	126.961	154.005	68.91	n.a.	BMB*
Total:			187.656	223.472	100.00	0.000	

Supplementary Figure 245. Chiral HPLC analysis of (*rac*)-**3bh**.

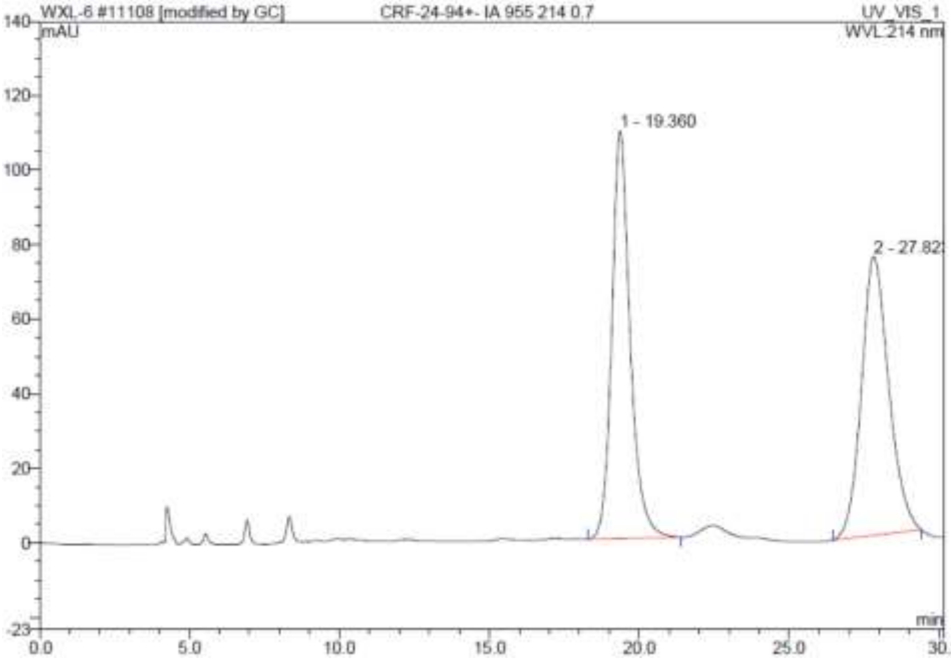

No.	Ret.Time min	Peak Name	Height mAU	Area mAU*min	Rel.Area %	Amount	Type
1	19.36	n.a.	109.256	77.440	49.46	n.a.	BMB*
2	27.82	n.a.	74.698	79.125	50.54	n.a.	BMB*
Total:			183.954	156.564	100.00	0.000	

Supplementary Figure 246.Chiral HPLC analysis of (*S*)-**3bh** from asymmetric reaction (Method **D**).

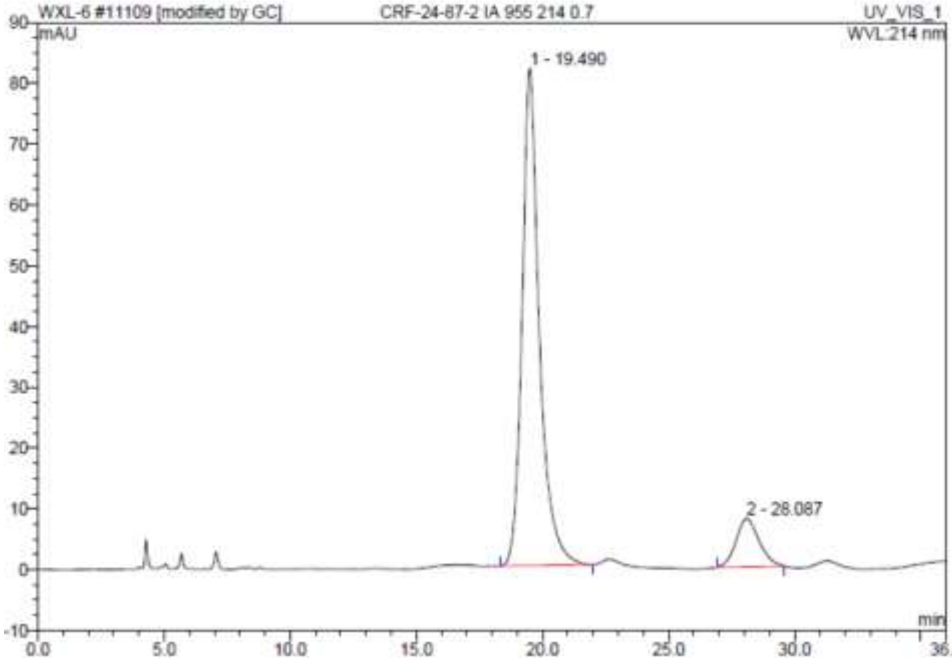

No.	Ret.Time min	Peak Name	Height mAU	Area mAU*min	Rel.Area %	Amount	Type
1	19.49	n.a.	81.834	64.751	88.53	n.a.	BMB*
2	28.09	n.a.	7.937	8.387	11.47	n.a.	BMB
Total:			89.770	73.138	100.00	0.000	

Supplementary Figure 247. Chiral HPLC analysis of (*rac*)-**3bi**.

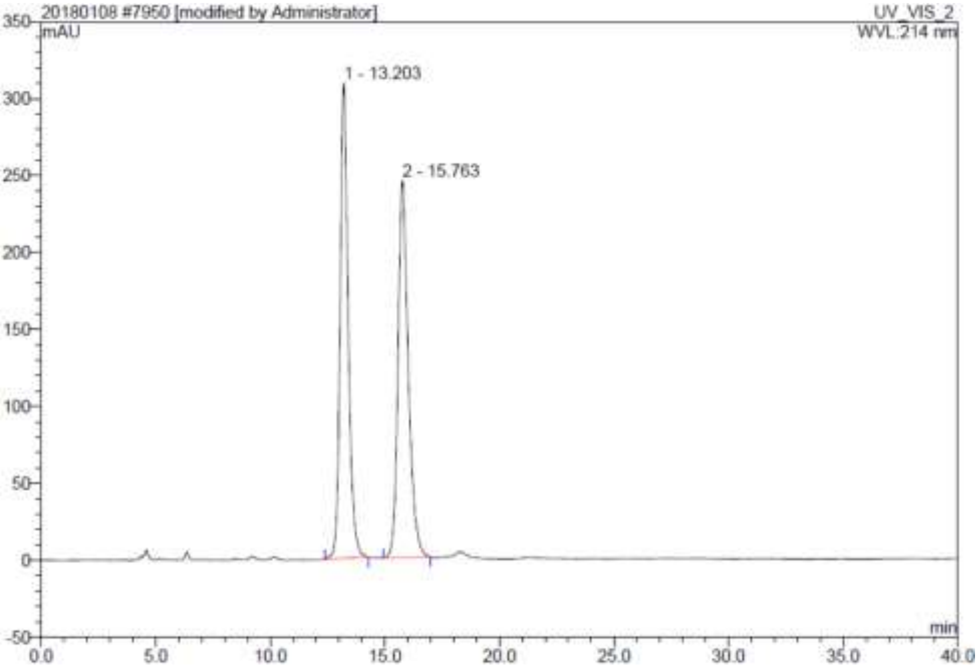

No.	Ret.Time min	Peak Name	Height mAU	Area mAU*min	Rel.Area %	Amount	Type
1	13.20	n.a.	308.869	125.924	49.73	n.a.	BMB
2	15.76	n.a.	245.072	127.268	50.27	n.a.	BMB
Total:			553.941	253.192	100.00	0.000	

Supplementary Figure 248. Chiral HPLC analysis of (*S*)-**3bi** from asymmetric reaction (Method **B**).

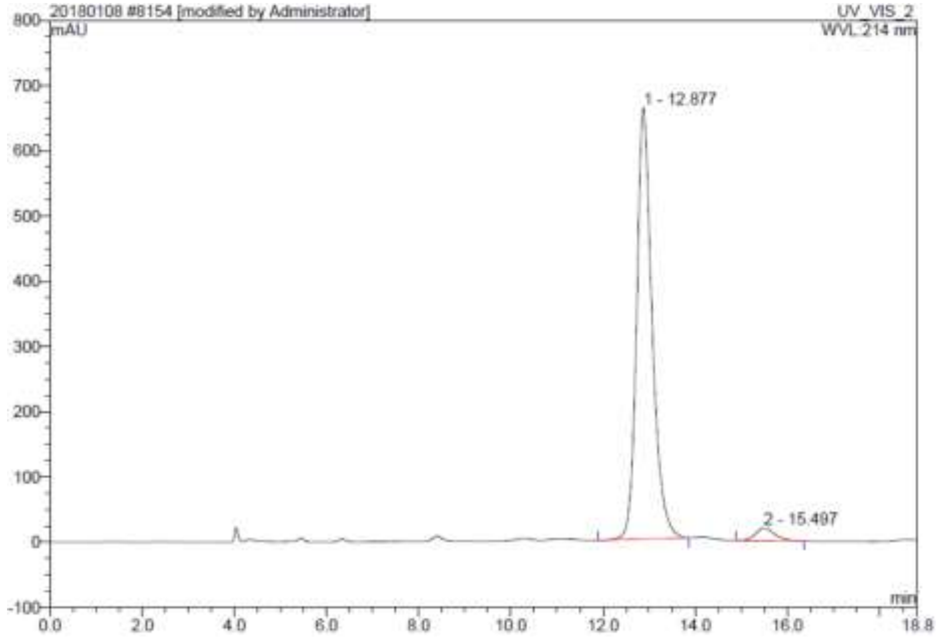

No.	Ret.Time min	Peak Name	Height mAU	Area mAU*min	Rel.Area %	Amount	Type
1	12.88	n.a.	662.132	258.186	96.47	n.a.	BMB*
2	15.50	n.a.	19.506	9.439	3.53	n.a.	BMB*
Total:			681.638	267.625	100.00	0.000	

Supplementary Figure 249. Chiral HPLC analysis of (*S*)-**3bi** from asymmetric reaction (Method **C**).

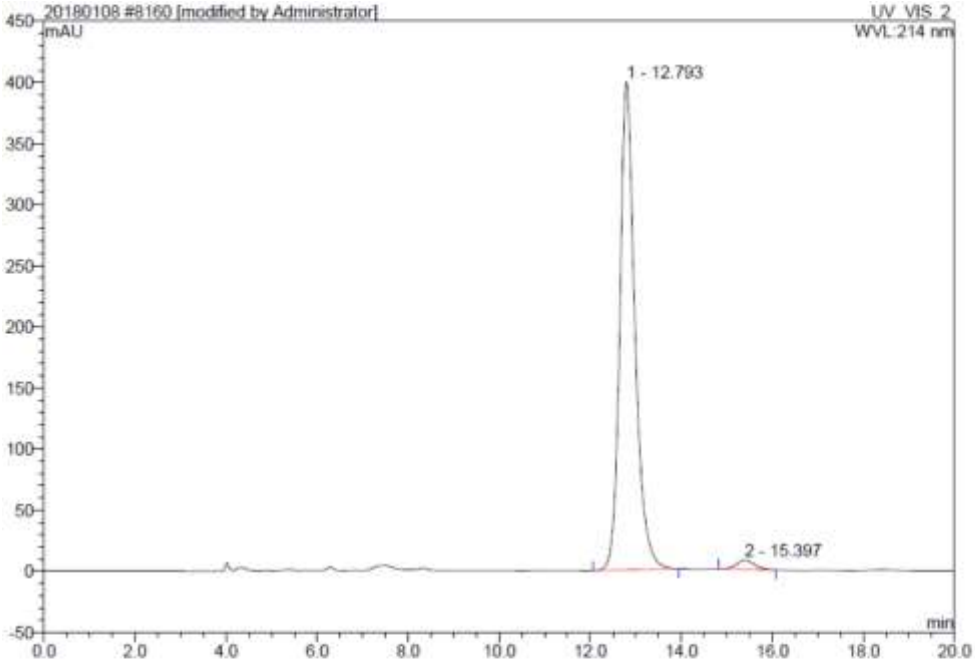

No.	Ret.Time min	Peak Name	Height mAU	Area mAU*min	Rel.Area %	Amount	Type
1	12.79	n.a.	399.371	153.660	97.76	n.a.	BMB
2	15.40	n.a.	7.660	3.519	2.24	n.a.	BMB
Total:			407.031	157.179	100.00	0.000	

Supplementary Figure 250. Chiral HPLC analysis of (*rac*)-**3bj**.

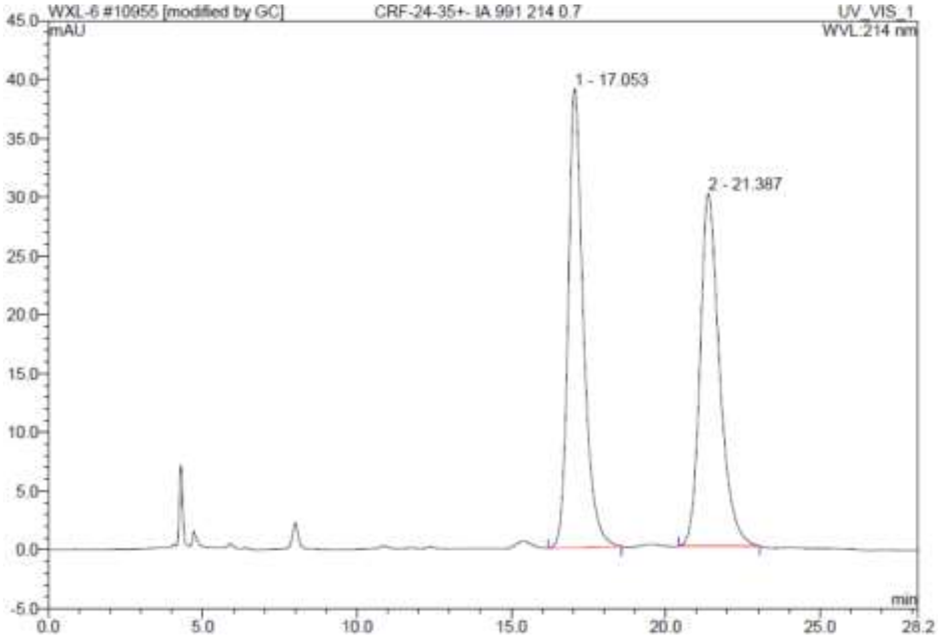

No.	Ret.Time min	Peak Name	Height mAU	Area mAU*min	Rel.Area %	Amount	Type
1	17.05	n.a.	39.045	22.405	50.42	n.a.	BMB
2	21.39	n.a.	29.995	22.031	49.58	n.a.	BMB
Total:			69.039	44.436	100.00	0.000	

Supplementary Figure 251. Chiral HPLC analysis of (*S*)-**3bj** from asymmetric reaction (Method **B**).

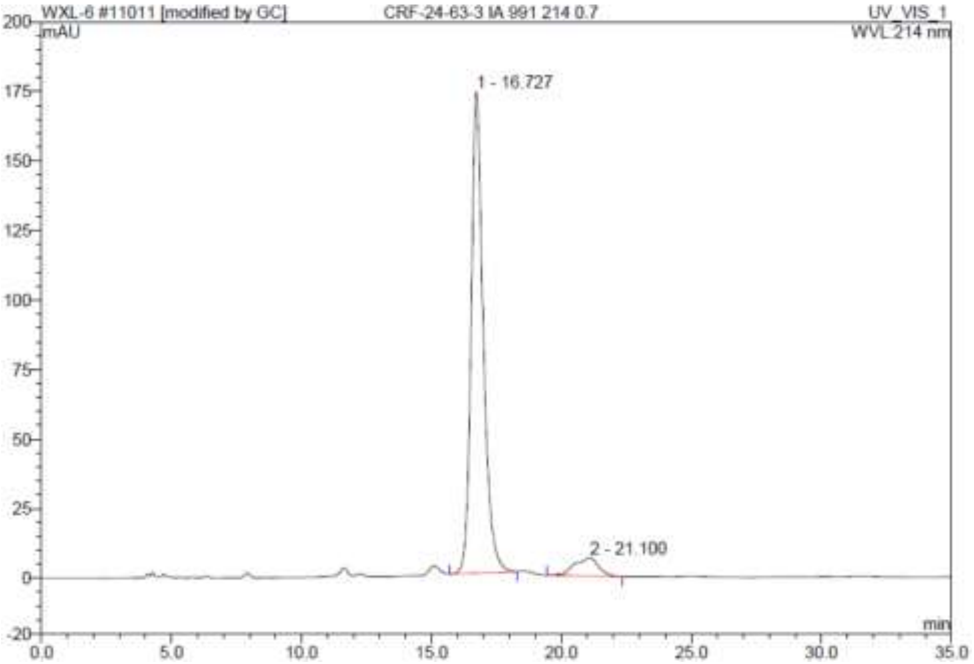

No.	Ret.Time min	Peak Name	Height mAU	Area mAU*min	Rel.Area %	Amount	Type
1	16.73	n.a.	173.146	93.933	93.07	n.a.	BMB*
2	21.10	n.a.	6.573	6.991	6.93	n.a.	BMB
Total:			179.719	100.924	100.00	0.000	

Supplementary Figure 252. Chiral HPLC analysis of (*S*)-**3bj** from asymmetric reaction (Method **C**).

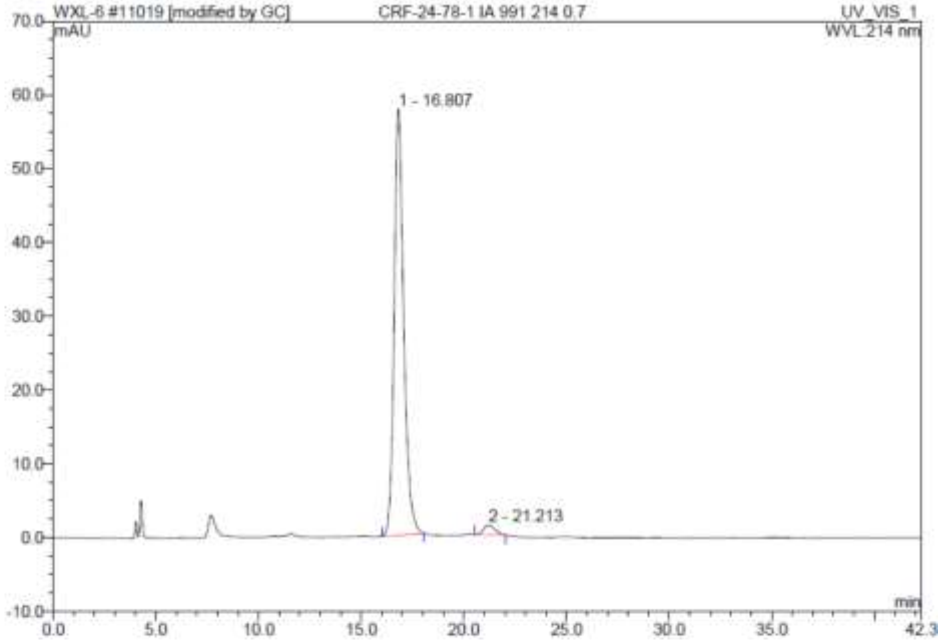

No.	Ret.Time min	Peak Name	Height mAU	Area mAU*min	Rel.Area %	Amount	Type
1	16.81	n.a.	57.797	31.117	97.38	n.a.	BMB
2	21.21	n.a.	1.318	0.837	2.62	n.a.	BMB
Total:			59.115	31.954	100.00	0.000	

Supplementary Figure 253. Chiral HPLC analysis of (*rac*)-**3bk**.

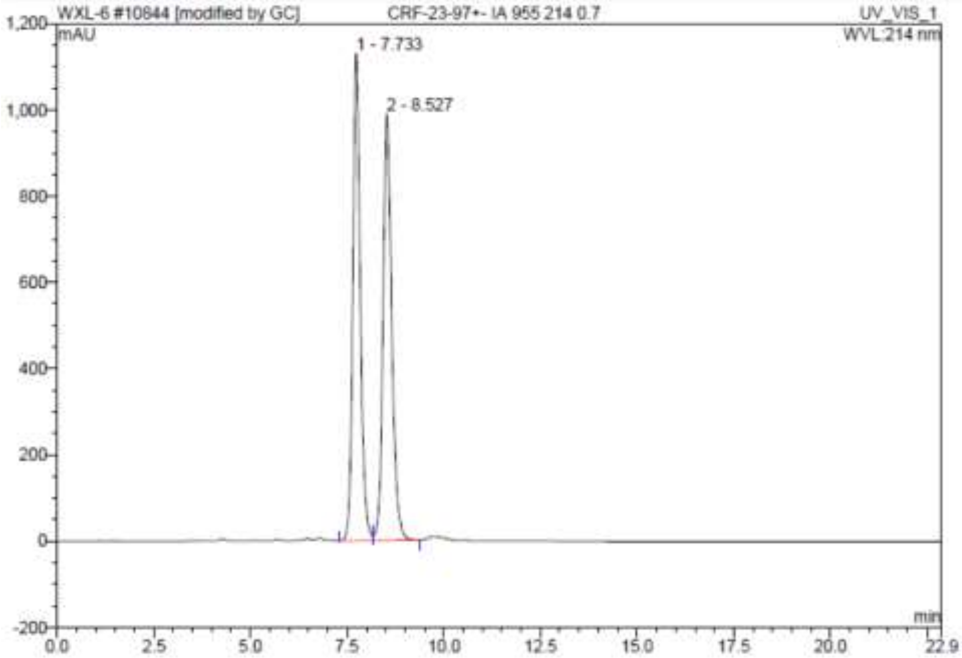

No.	Ret.Time min	Peak Name	Height mAU	Area mAU*min	Rel.Area %	Amount	Type
1	7.73	n.a.	1130.626	253.344	49.48	n.a.	BM *
2	8.53	n.a.	989.626	258.623	50.52	n.a.	MB*
Total:			2120.252	511.966	100.00	0.000	

Supplementary Figure 254. Chiral HPLC analysis of (*S*)-**3bk** from asymmetric reaction (Method **B**).

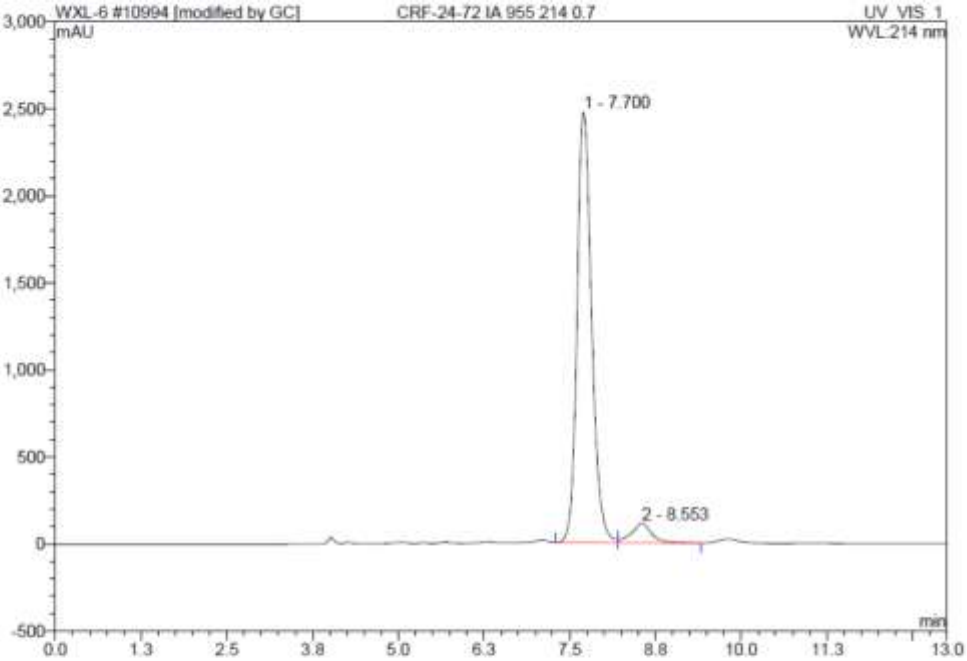

No.	Ret.Time min	Peak Name	Height mAU	Area mAU*min	Rel.Area %	Amount	Type
1	7.70	n.a.	2475.577	609.503	94.08	n.a.	BM *
2	8.55	n.a.	112.750	38.329	5.92	n.a.	MB*
Total:			2588.327	647.832	100.00	0.000	

Supplementary Figure 255. Chiral HPLC analysis of (*S*)-**3bk** from asymmetric reaction (Method **C**).

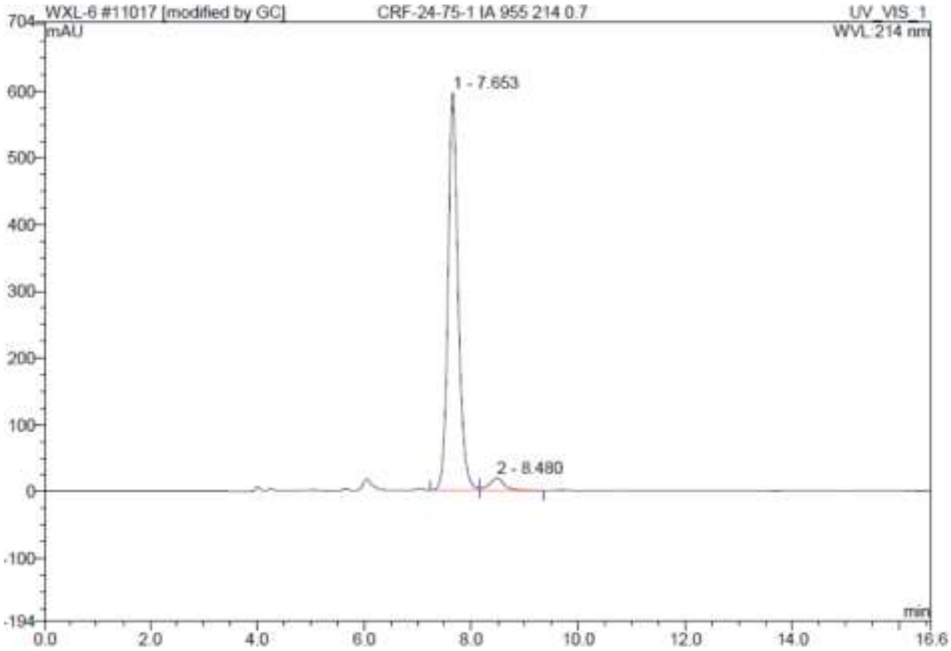

No.	Ret.Time min	Peak Name	Height mAU	Area mAU*min	Rel.Area %	Amount	Type
1	7.65	n.a.	595.724	130.570	95.37	n.a.	BM *
2	8.48	n.a.	19.039	6.345	4.63	n.a.	MB*
Total:			614.763	136.914	100.00	0.000	

Supplementary Figure 256. Chiral HPLC analysis of (*rac*)-**3bl**.

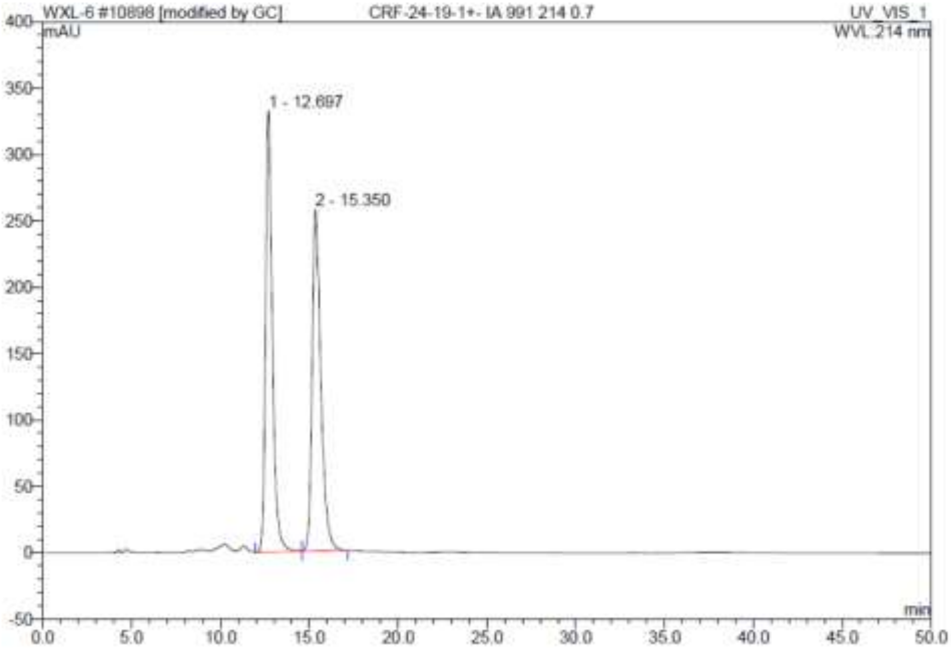

No.	Ret.Time min	Peak Name	Height mAU	Area mAU*min	Rel.Area %	Amount	Type
1	12.70	n.a.	332.423	147.270	50.13	n.a.	BM
2	15.35	n.a.	257.353	146.493	49.87	n.a.	MB
Total:			589.776	293.762	100.00	0.000	

Supplementary Figure 257. Chiral HPLC analysis of (*S*)-**3bl** from asymmetric reaction (Method **B**).

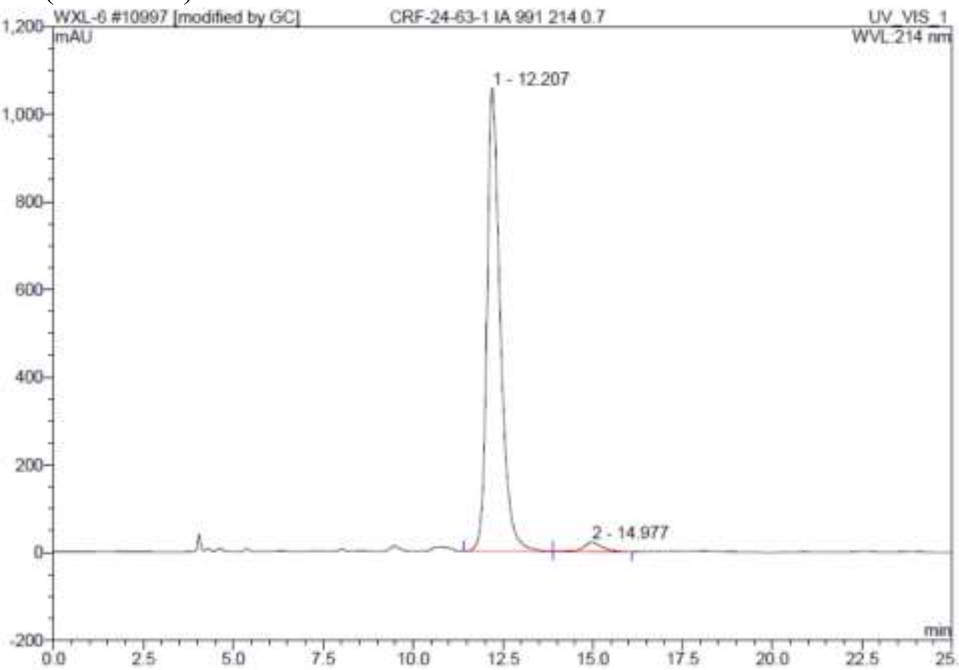

No.	Ret.Time min	Peak Name	Height mAU	Area mAU*min	Rel.Area %	Amount	Type
1	12.21	n.a.	1057.706	456.373	97.10	n.a.	BM
2	14.98	n.a.	21.740	13.653	2.90	n.a.	MB
Total:			1079.445	470.026	100.00	0.000	

Supplementary Figure 258. Chiral HPLC analysis of (*S*)-**3bl** from asymmetric reaction (Method **C**).

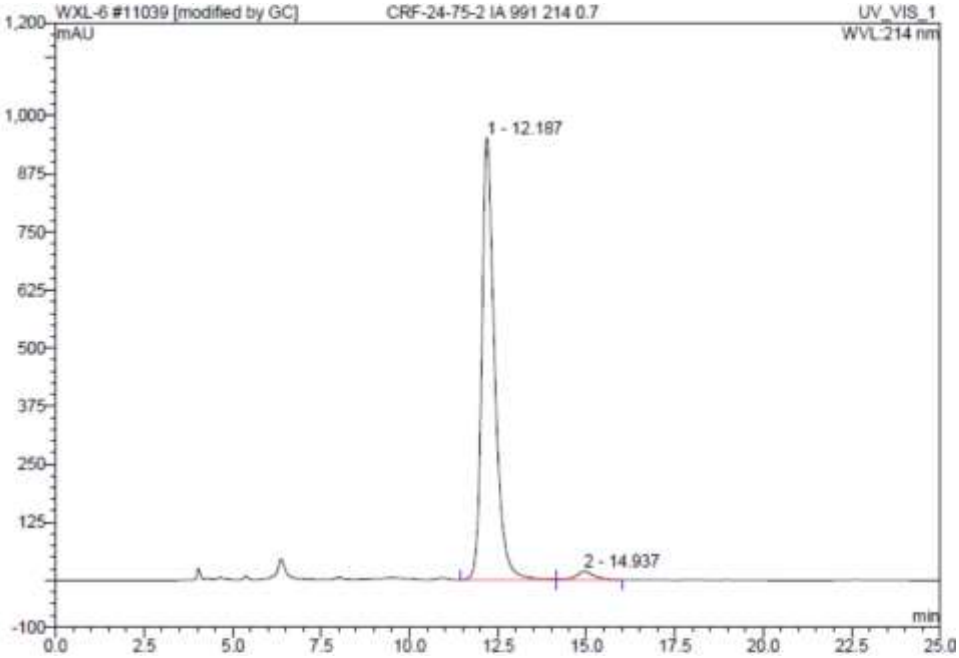

No.	Ret.Time min	Peak Name	Height mAU	Area mAU*min	Rel.Area %	Amount	Type
1	12.19	n.a.	950.950	400.563	97.23	n.a.	BM
2	14.94	n.a.	18.733	11.391	2.77	n.a.	MB
Total:			969.683	411.954	100.00	0.000	

Supplementary Figure 259. Chiral HPLC analysis of (*rac*)-**3bm**.

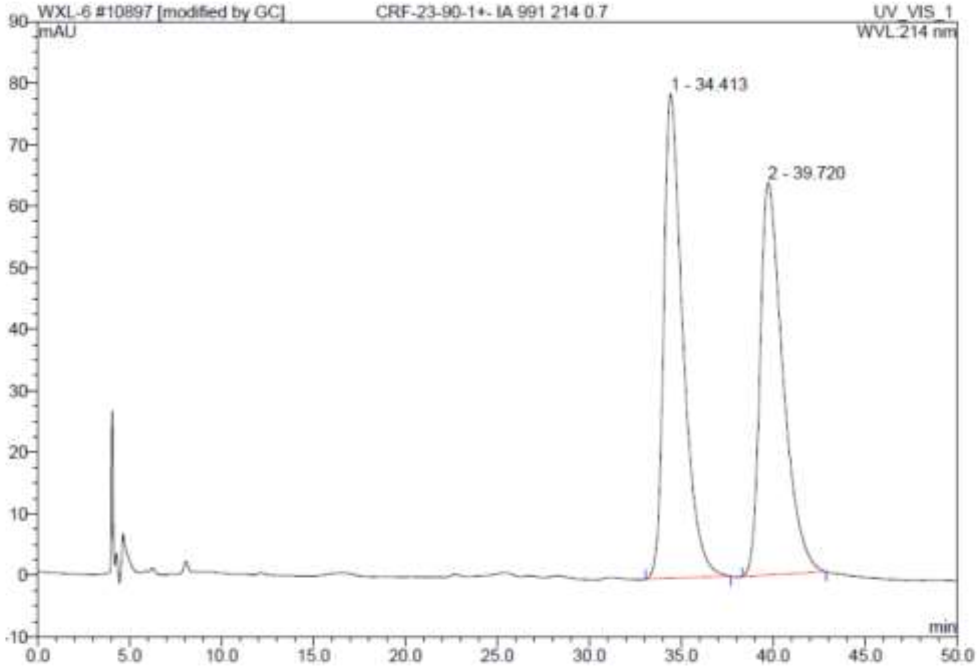

No.	Ret.Time min	Peak Name	Height mAU	Area mAU*min	Rel.Area %	Amount	Type
1	34.41	n.a.	78.807	95.469	50.86	n.a.	BMB*
2	39.72	n.a.	63.762	92.230	49.14	n.a.	BMB
Total:			142.569	187.699	100.00	0.000	

Supplementary Figure 260. Chiral HPLC analysis of (*S*)-**3bm** from asymmetric reaction (Method **A**).

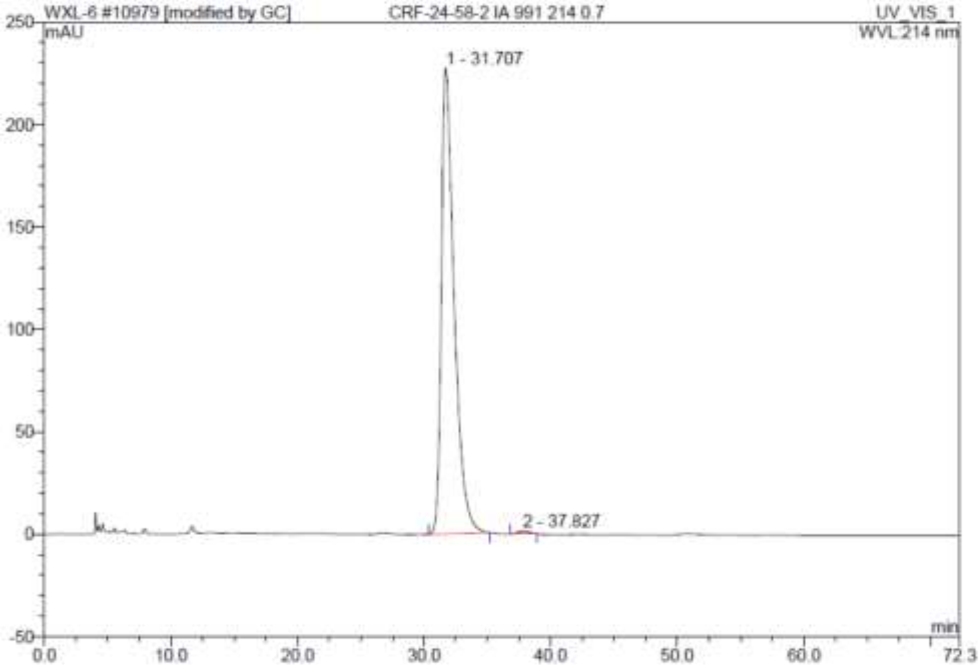

No.	Ret.Time min	Peak Name	Height mAU	Area mAU*min	Rel.Area %	Amount	Type
1	31.71	n.a.	227.529	266.750	99.40	n.a.	BMB
2	37.83	n.a.	1.523	1.605	0.60	n.a.	BMB
Total:			229.053	268.355	100.00	0.000	

Supplementary Figure 261. Chiral HPLC analysis of (*rac*)-**3bn**.

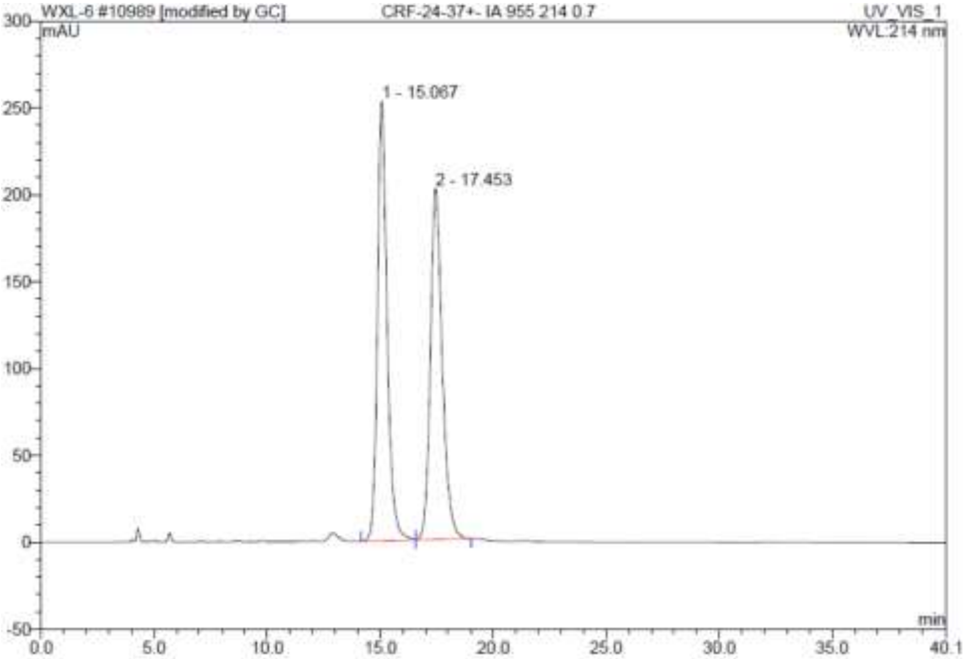

No.	Ret.Time min	Peak Name	Height mAU	Area mAU*min	Rel.Area %	Amount	Type
1	15.07	n.a.	253.040	125.165	50.51	n.a.	BM
2	17.45	n.a.	201.892	122.629	49.49	n.a.	MB
Total:			454.932	247.794	100.00	0.000	

Supplementary Figure 262. Chiral HPLC analysis of (*S*)-**3bn** from asymmetric reaction (Method **B**).

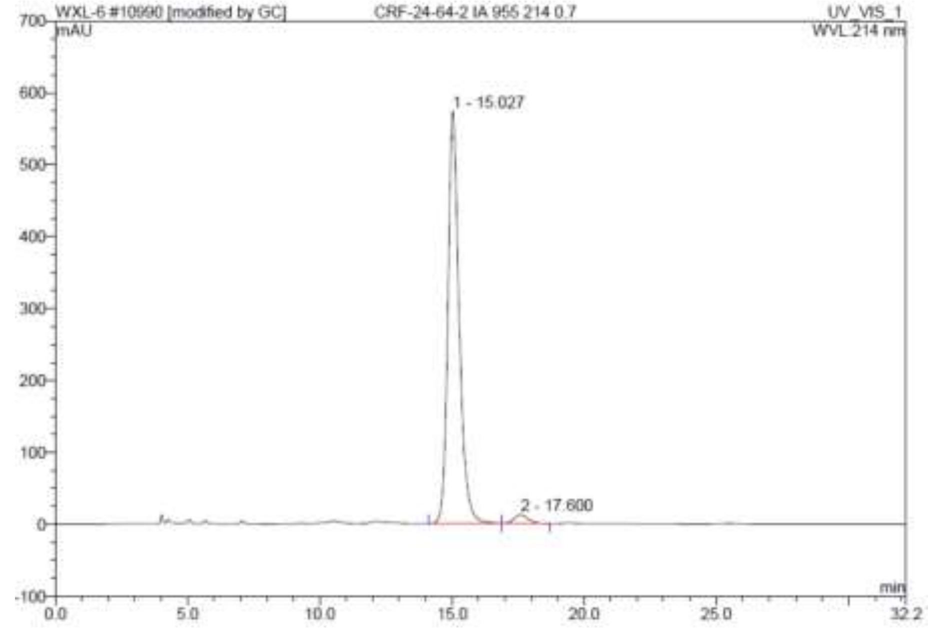

No.	Ret.Time min	Peak Name	Height mAU	Area mAU*min	Rel.Area %	Amount	Type
1	15.03	n.a.	574.247	283.991	97.24	n.a.	BM
2	17.60	n.a.	12.591	8.075	2.76	n.a.	MB
Total:			586.838	292.066	100.00	0.000	

Supplementary Figure 263. Chiral HPLC analysis of (*S*)-**3bn** from asymmetric reaction (Method **C**).

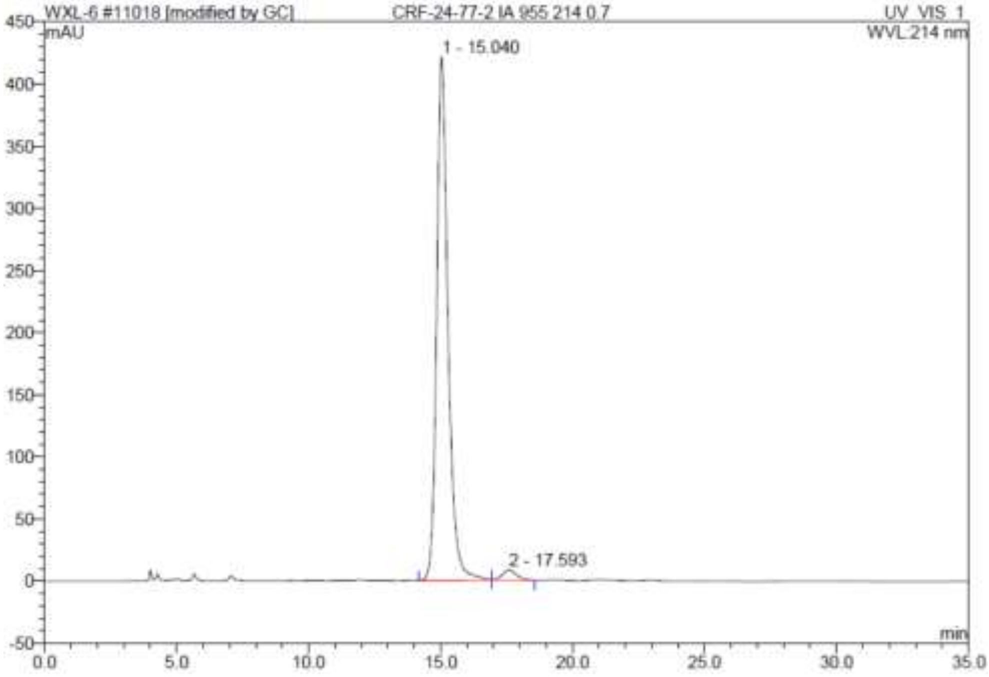

No.	Ret.Time min	Peak Name	Height mAU	Area mAU*min	Rel.Area %	Amount	Type
1	15.04	n.a.	421.565	210.145	97.48	n.a.	BM
2	17.59	n.a.	8.222	5.429	2.52	n.a.	MB
Total:			429.787	215.574	100.00	0.000	

Supplementary Figure 264. Chiral HPLC analysis of (*rac*)-**3bo**.

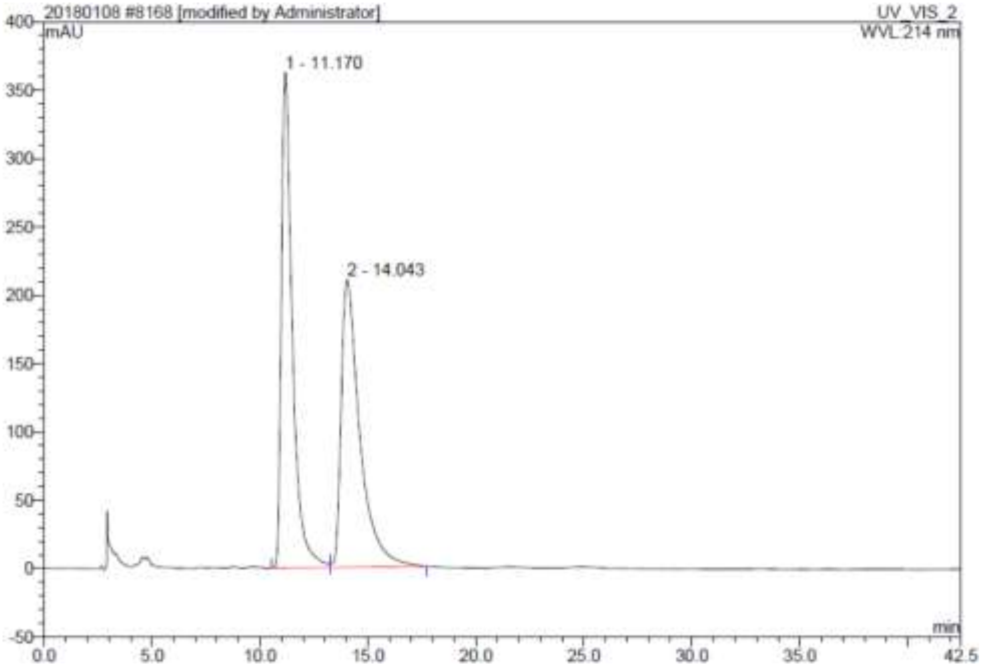

No.	Ret.Time min	Peak Name	Height mAU	Area mAU*min	Rel.Area %	Amount	Type
1	11.17	n.a.	362.672	215.437	50.56	n.a.	BM
2	14.04	n.a.	210.392	210.648	49.44	n.a.	MB
Total:			573.063	426.084	100.00	0.000	

Supplementary Figure 265. Chiral HPLC analysis of (*S*)-**3bo** from asymmetric reaction (Method **B**).

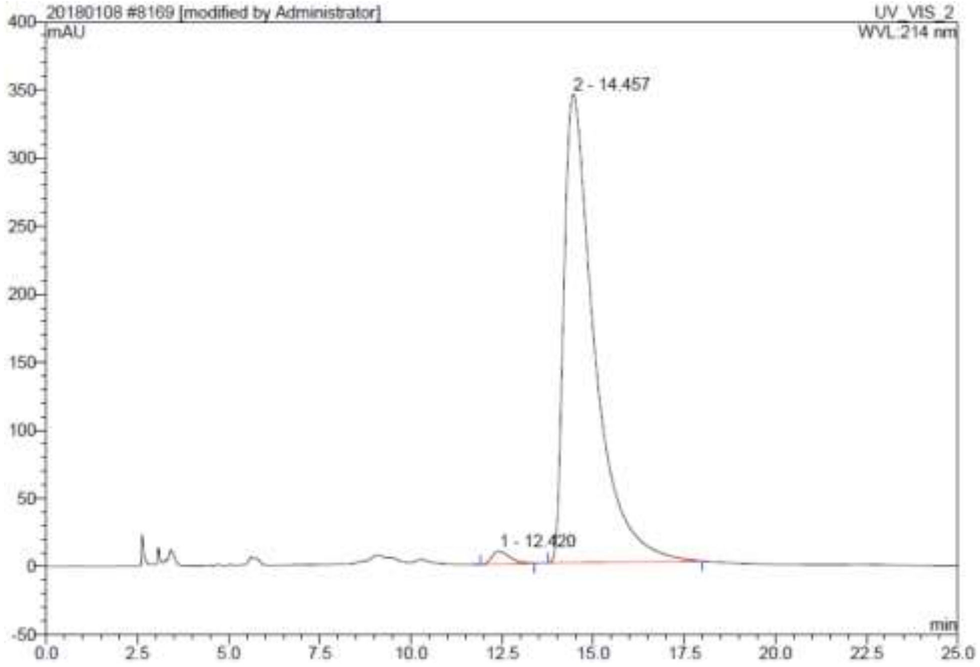

No.	Ret.Time min	Peak Name	Height mAU	Area mAU*min	Rel.Area %	Amount	Type
1	12.42	n.a.	9.598	5.417	1.60	n.a.	BMB
2	14.46	n.a.	344.171	333.019	98.40	n.a.	BMB
Total:			353.769	338.436	100.00	0.000	

Supplementary Figure 266. Chiral HPLC analysis of (*S*)-**3bo** from asymmetric reaction (Method **C**).

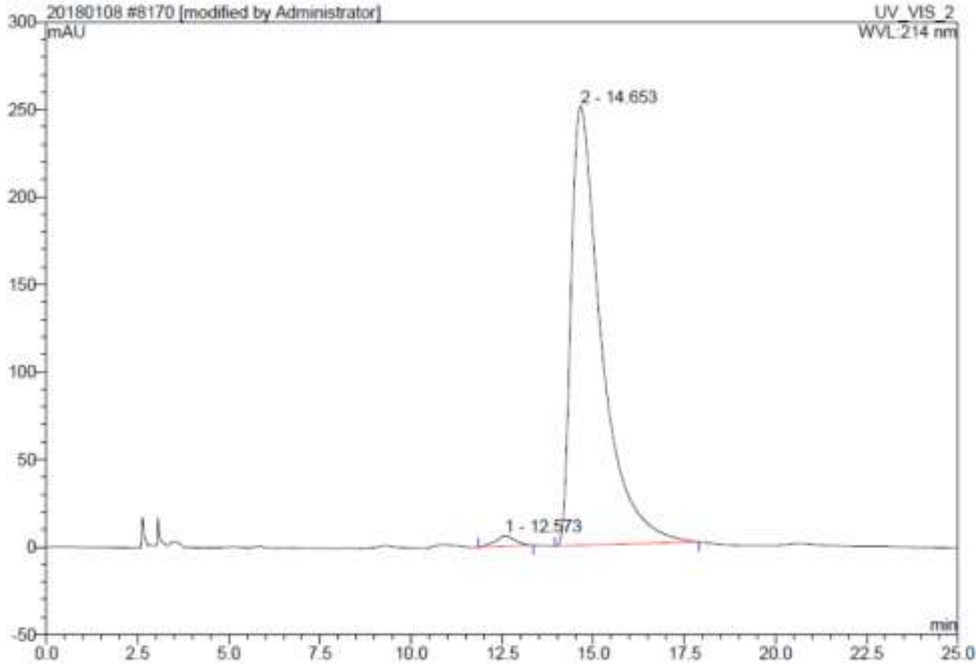

No.	Ret.Time min	Peak Name	Height mAU	Area mAU*min	Rel.Area %	Amount	Type
1	12.57	n.a.	5.993	3.761	1.53	n.a.	BMB
2	14.65	n.a.	251.077	242.348	98.47	n.a.	BMB
Total:			257.069	246.109	100.00	0.000	

Supplementary Figure 267. Chiral HPLC analysis of (*rac*)-**3bp**.

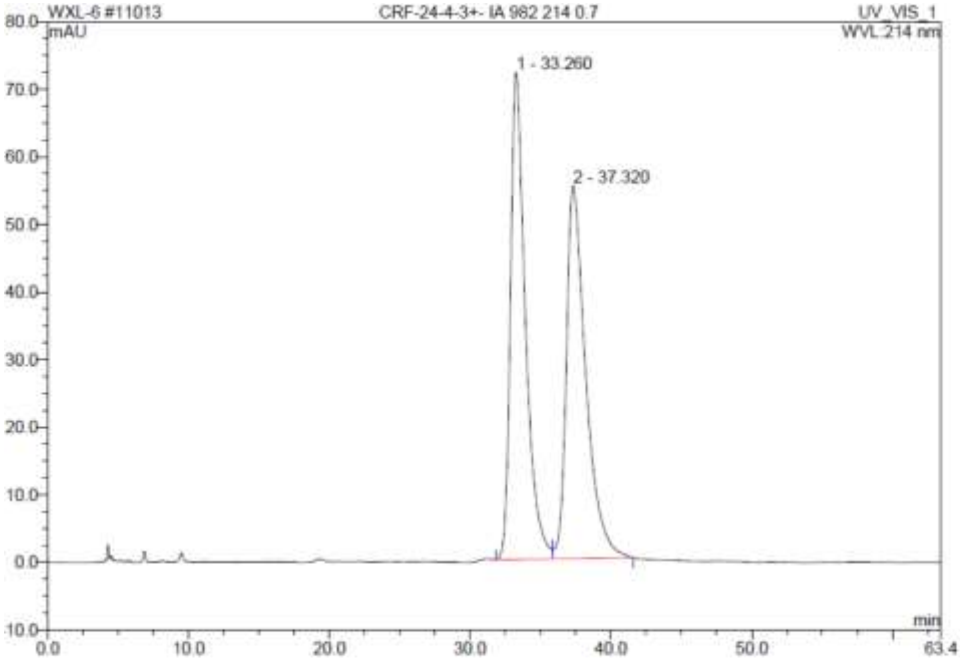

No.	Ret.Time min	Peak Name	Height mAU	Area mAU*min	Rel.Area %	Amount	Type
1	33.26	n.a.	72.096	88.607	50.17	n.a.	BM
2	37.32	n.a.	55.179	88.014	49.83	n.a.	MB
Total:			127.275	176.621	100.00	0.000	

Supplementary Figure 268. Chiral HPLC analysis of (*S*)-**3bp** from asymmetric reaction (Method **B**).

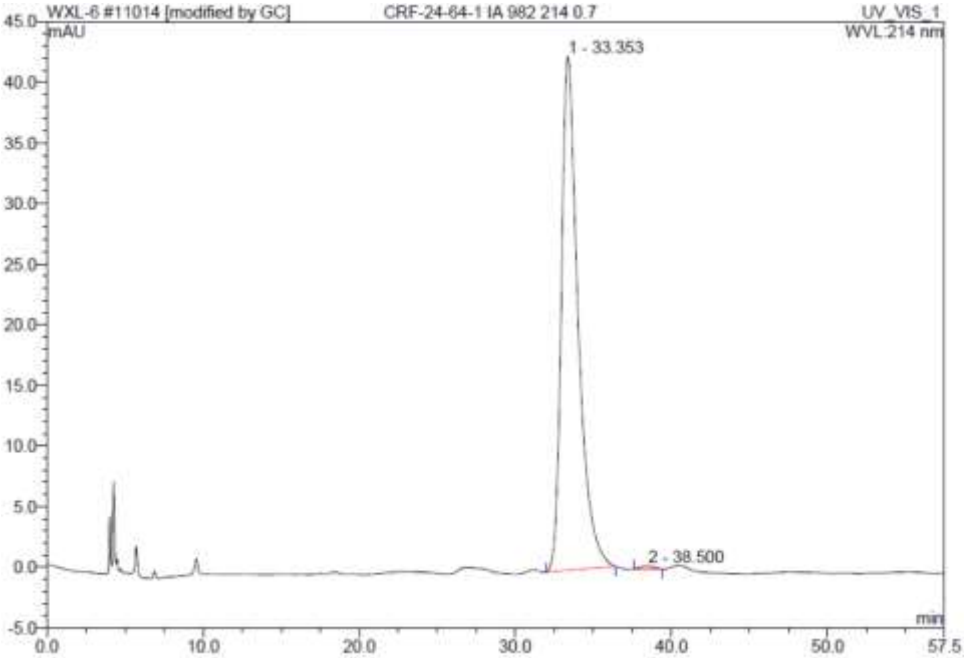

No.	Ret.Time min	Peak Name	Height mAU	Area mAU*min	Rel.Area %	Amount	Type
1	33.35	n.a.	42.416	52.662	99.53	n.a.	BMB
2	38.50	n.a.	0.246	0.246	0.47	n.a.	BMB*
Total:			42.661	52.908	100.00	0.000	

Supplementary Figure 269. Chiral HPLC analysis of (*S*)-**3bp** from asymmetric reaction (Method **C**).

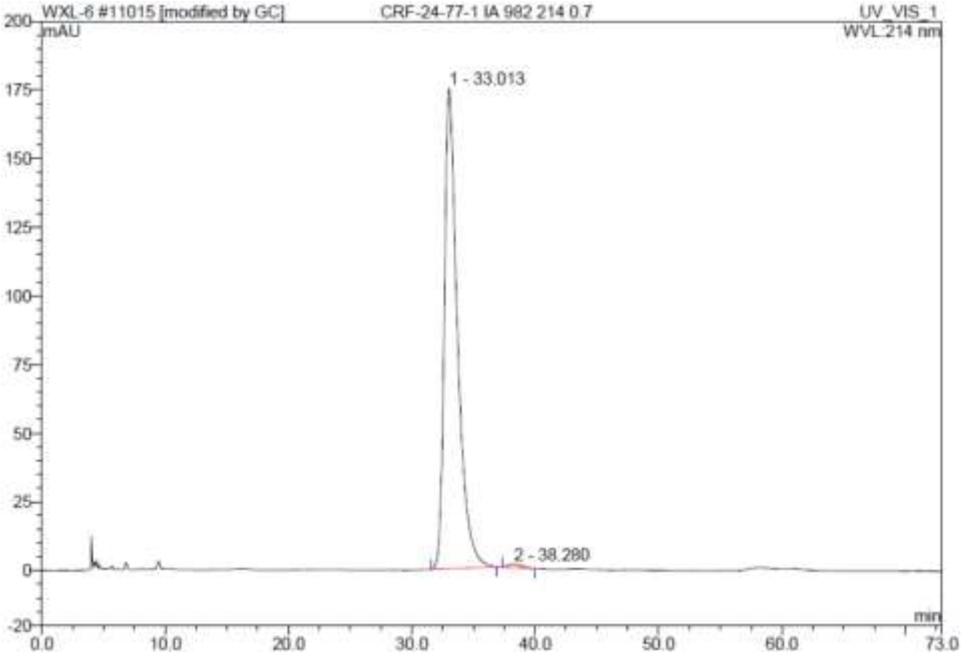

No.	Ret.Time min	Peak Name	Height mAU	Area mAU*min	Rel.Area %	Amount	Type
1	33.01	n.a.	175.029	216.832	99.24	n.a.	BMB
2	38.28	n.a.	1.286	1.656	0.76	n.a.	BMB*
Total:			176.315	218.488	100.00	0.000	

Supplementary Figure 270. Chiral HPLC analysis of (*rac*)-**3bq**.

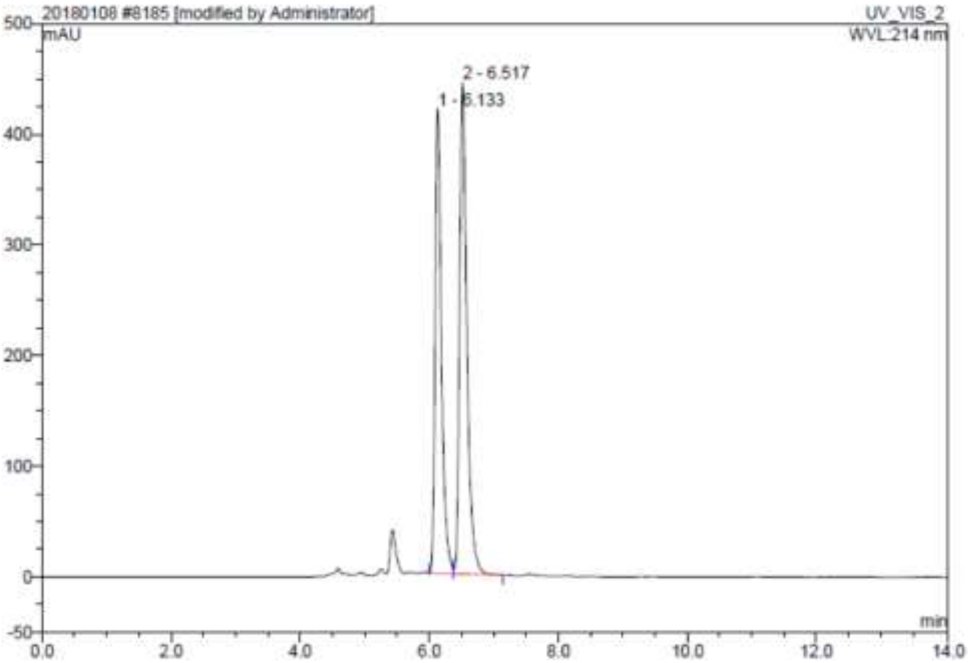

No.	Ret.Time min	Peak Name	Height mAU	Area mAU*min	Rel.Area %	Amount	Type
1	6.13	n.a.	420.068	48.125	45.28	n.a.	BM
2	6.52	n.a.	444.351	58.154	54.72	n.a.	MB
Total:			864.420	106.279	100.00	0.000	

Supplementary Figure 271. Chiral HPLC analysis of (*S*)-**3bq** from asymmetric reaction (Method **A**).

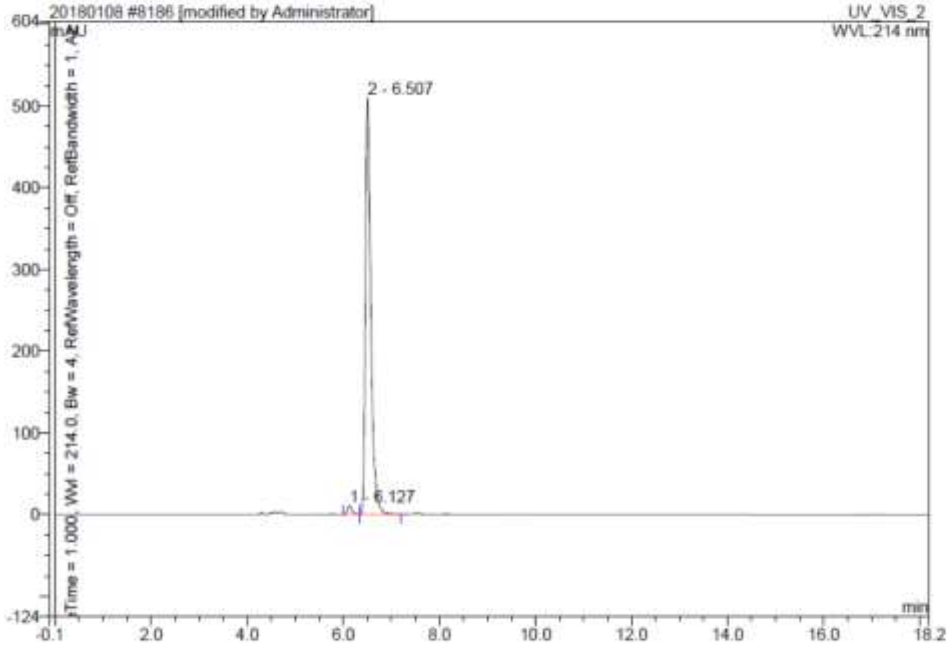

No.	Ret.Time min	Peak Name	Height mAU	Area mAU*min	Rel.Area %	Amount	Type
1	6.13	n.a.	10.605	1.205	1.77	n.a.	BM
2	6.51	n.a.	510.310	66.828	98.23	n.a.	MB
Total:			520.915	68.032	100.00	0.000	

Supplementary Figure 272. Chiral HPLC analysis of (*rac*)-**3br**.

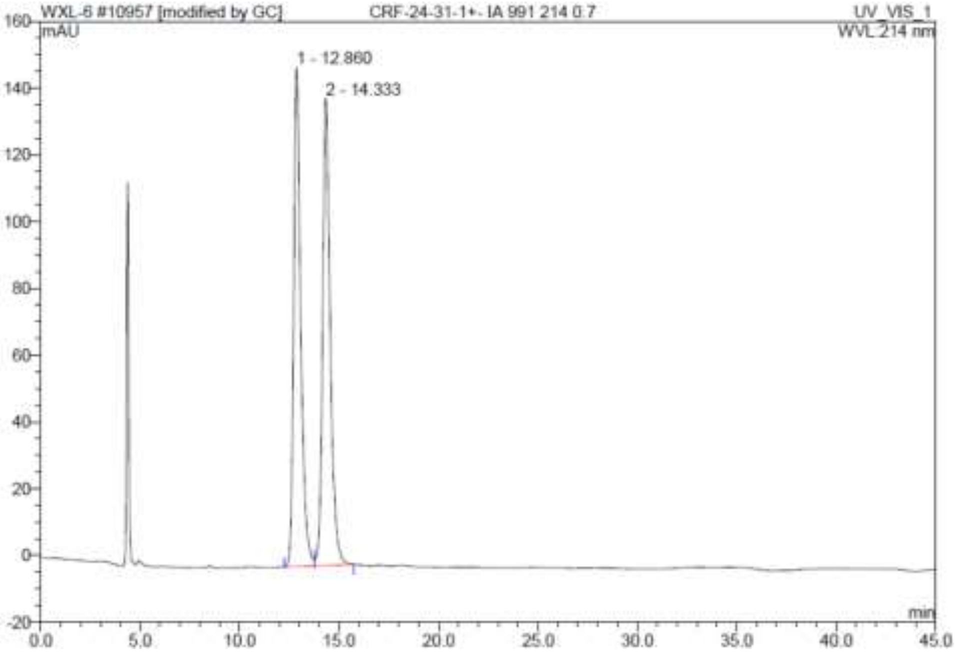

No.	Ret.Time min	Peak Name	Height mAU	Area mAU*min	Rel.Area %	Amount	Type
1	12.86	n.a.	149.405	61.549	49.75	n.a.	BM
2	14.33	n.a.	139.944	62.169	50.25	n.a.	MB
Total:			289.349	123.718	100.00	0.000	

Supplementary Figure 273. Chiral HPLC analysis of (*S*)-**3br** from asymmetric reaction (Method **B**).

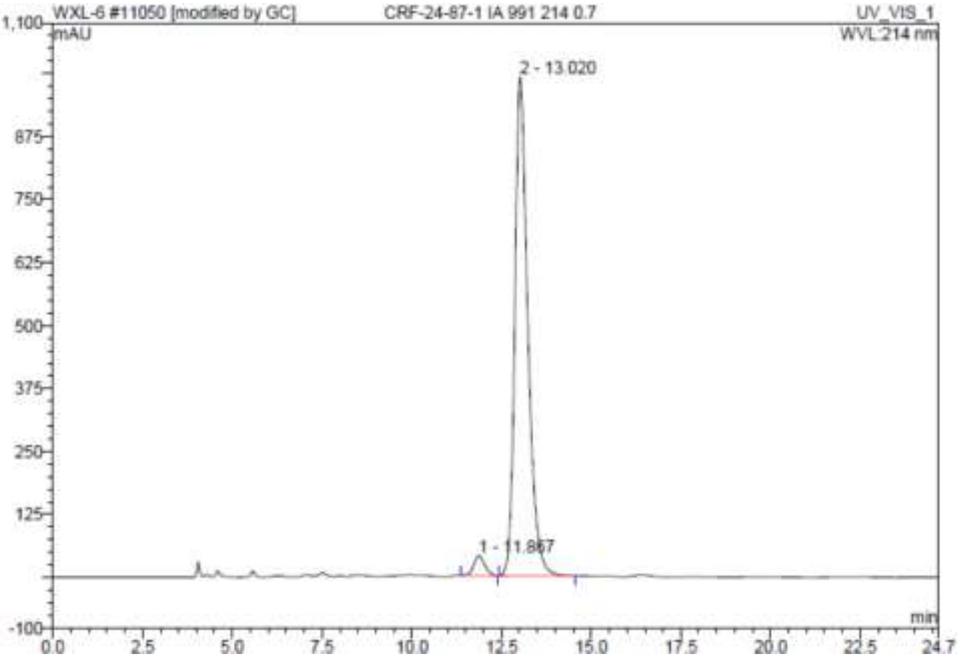

No.	Ret.Time min	Peak Name	Height mAU	Area mAU*min	Rel.Area %	Amount	Type
1	11.87	n.a.	38.975	13.693	3.20	n.a.	BMB
2	13.02	n.a.	989.098	414.368	96.80	n.a.	BMB
Total:			1028.073	428.061	100.00	0.000	

Supplementary Figure 274. Chiral HPLC analysis of (*S*)-**3br** from asymmetric reaction (Method **C**).

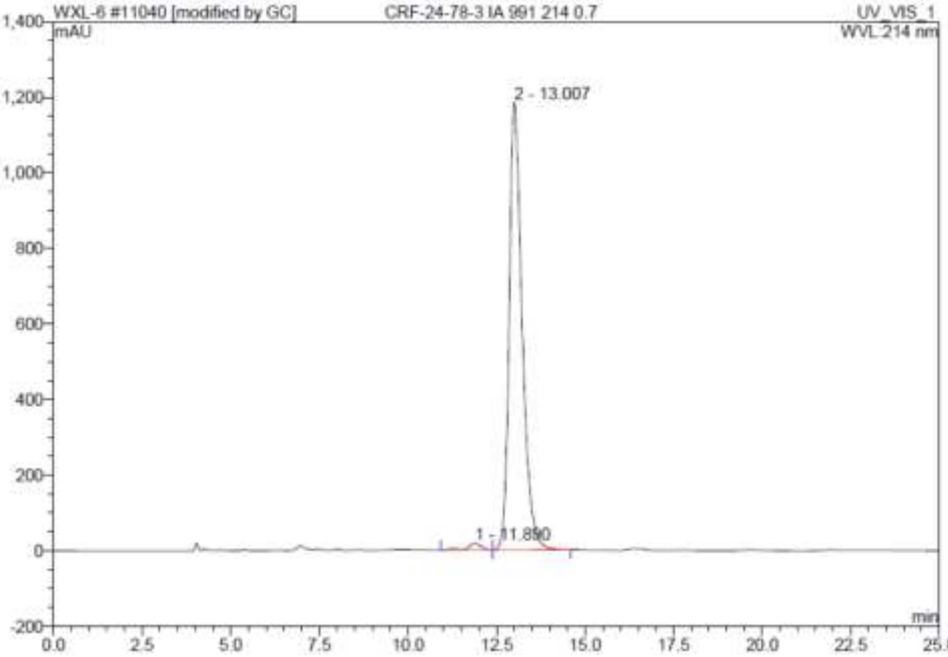

No.	Ret.Time min	Peak Name	Height mAU	Area mAU*min	Rel.Area %	Amount	Type
1	11.89	n.a.	18.473	8.040	1.58	n.a.	BM *
2	13.01	n.a.	1184.693	501.255	98.42	n.a.	MB
Total:			1203.166	509.296	100.00	0.000	

Supplementary Figure 275. Chiral HPLC analysis of (*rac*)-**3bs**.

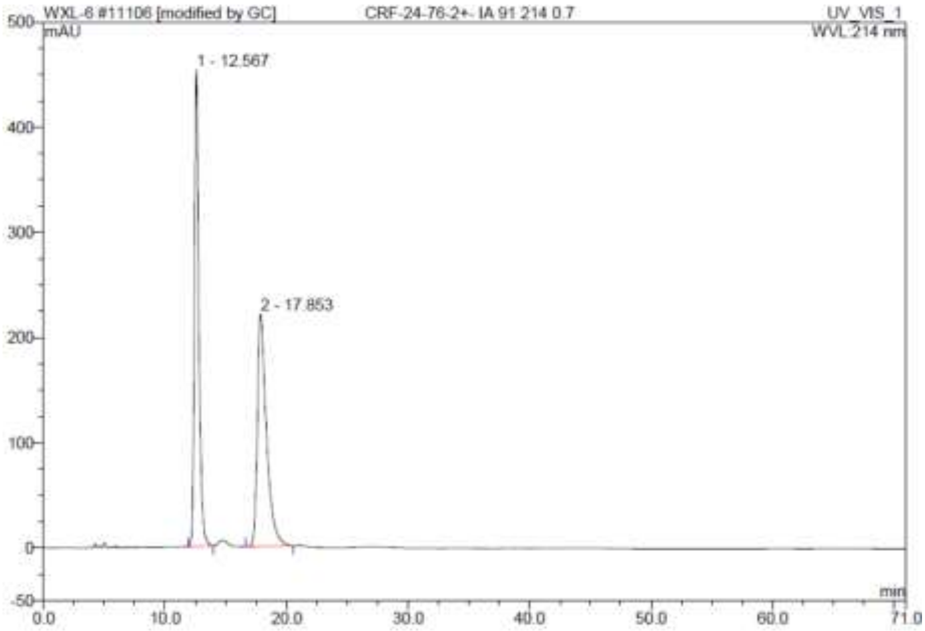

No.	Ret.Time min	Peak Name	Height mAU	Area mAU*min	Rel.Area %	Amount	Type
1	12.57	n.a.	453.424	191.953	49.98	n.a.	BMB*
2	17.85	n.a.	220.905	192.109	50.02	n.a.	BMB
Total:			674.329	384.061	100.00	0.000	

Supplementary Figure 276. Chiral HPLC analysis of (*S*)-**3bs** from asymmetric reaction (Method **C**).

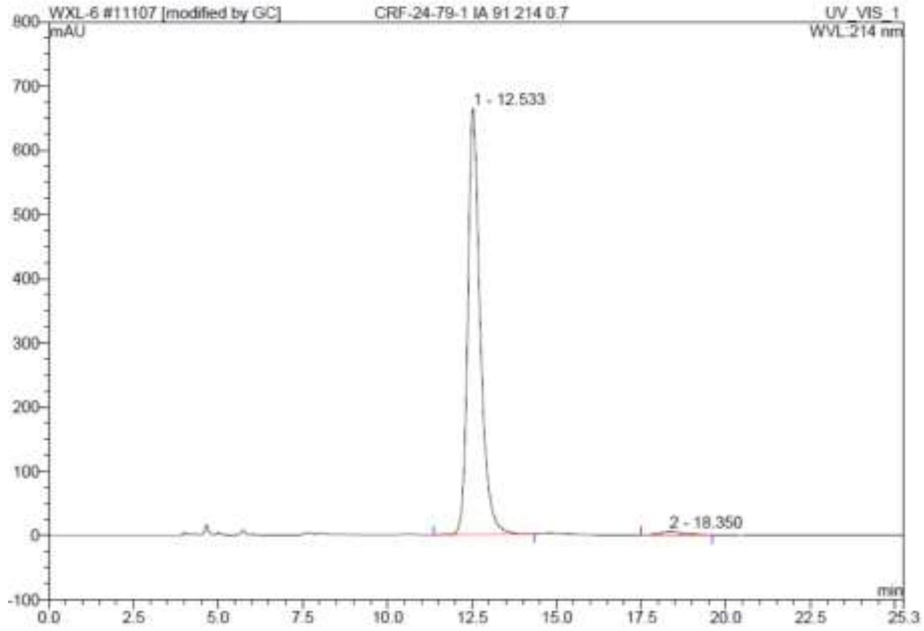

No.	Ret.Time min	Peak Name	Height mAU	Area mAU*min	Rel.Area %	Amount	Type
1	12.53	n.a.	664.184	283.029	98.43	n.a.	BMB*
2	18.35	n.a.	5.027	4.513	1.57	n.a.	BMB
Total:			669.211	287.542	100.00	0.000	

Supplementary Figure 277. Chiral HPLC analysis of (*rac*)-**3bt**.

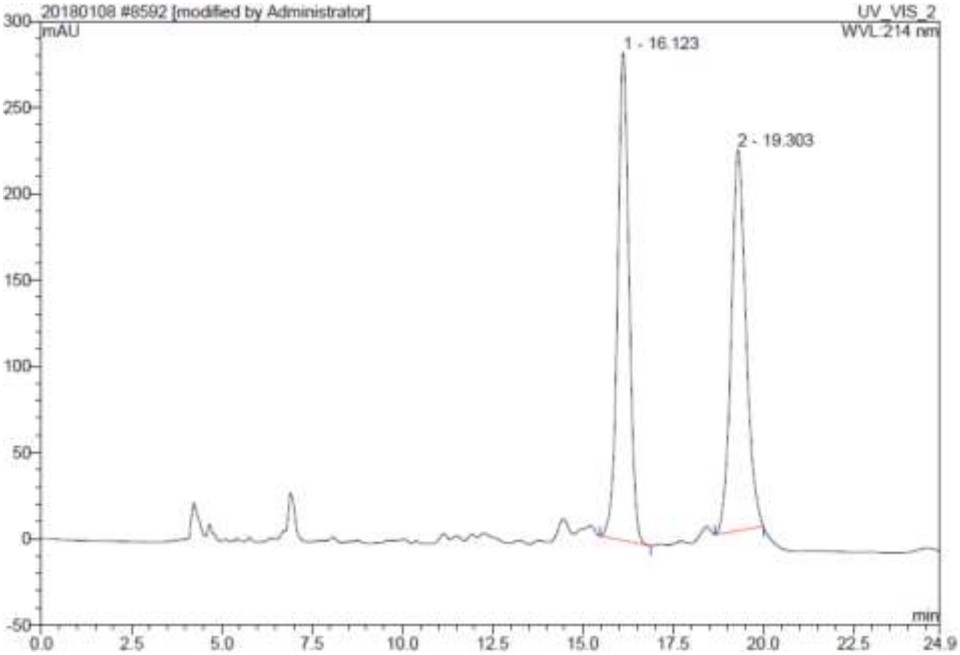

No.	Ret.Time min	Peak Name	Height mAU	Area mAU*min	Rel.Area %	Amount	Type
1	16.12	n.a.	282.693	104.238	49.89	n.a.	BMB*
2	19.30	n.a.	220.718	104.690	50.11	n.a.	BMB*
Total:			503.412	208.927	100.00	0.000	

Supplementary Figure 278. Chiral HPLC analysis of (*S*)-**3bt** from asymmetric reaction (Method **B**).

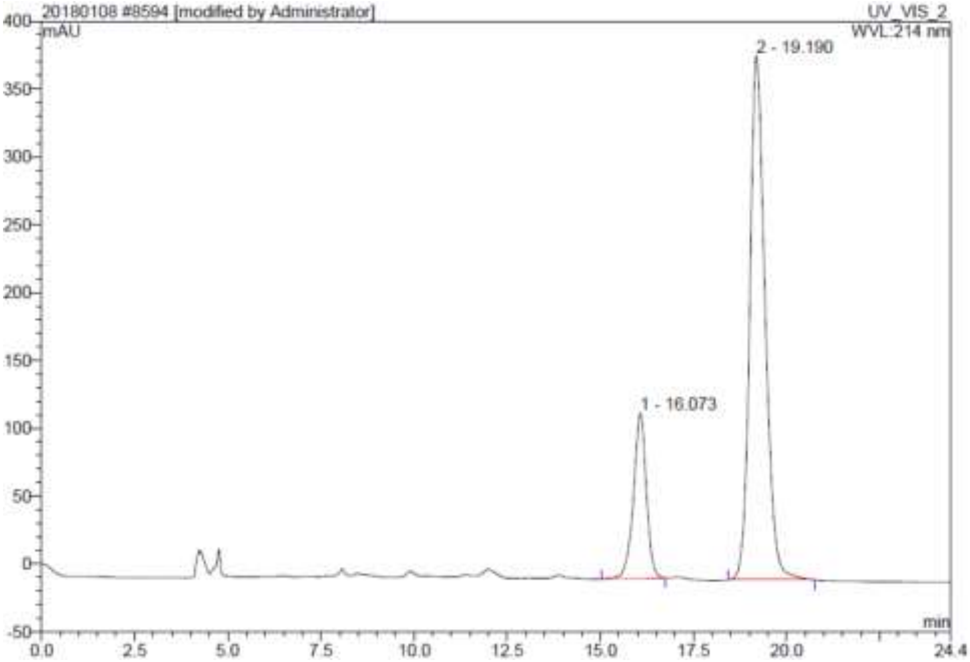

No.	Ret.Time min	Peak Name	Height mAU	Area mAU*min	Rel.Area %	Amount	Type
1	16.07	n.a.	121.885	48.901	20.96	n.a.	BMB
2	19.19	n.a.	386.136	184.439	79.04	n.a.	BMB
Total:			508.021	233.341	100.00	0.000	

Supplementary Figure 279. Chiral HPLC analysis of (*rac*)-**3bt'**.

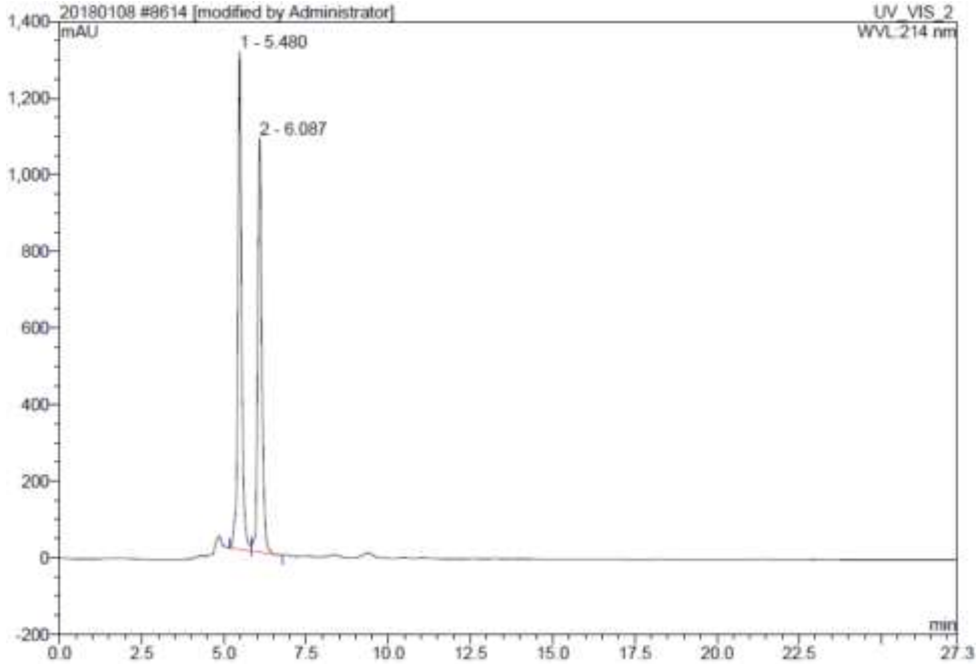

No.	Ret.Time min	Peak Name	Height mAU	Area mAU*min	Rel.Area %	Amount	Type
1	5.48	n.a.	1252.682	152.046	50.18	n.a.	BMB*
2	6.09	n.a.	1063.574	150.936	49.82	n.a.	BMB*
Total:			2316.256	302.982	100.00	0.000	

Supplementary Figure 280. Chiral HPLC analysis of (*S*)-**3bt'** from asymmetric reaction (Method **B**).

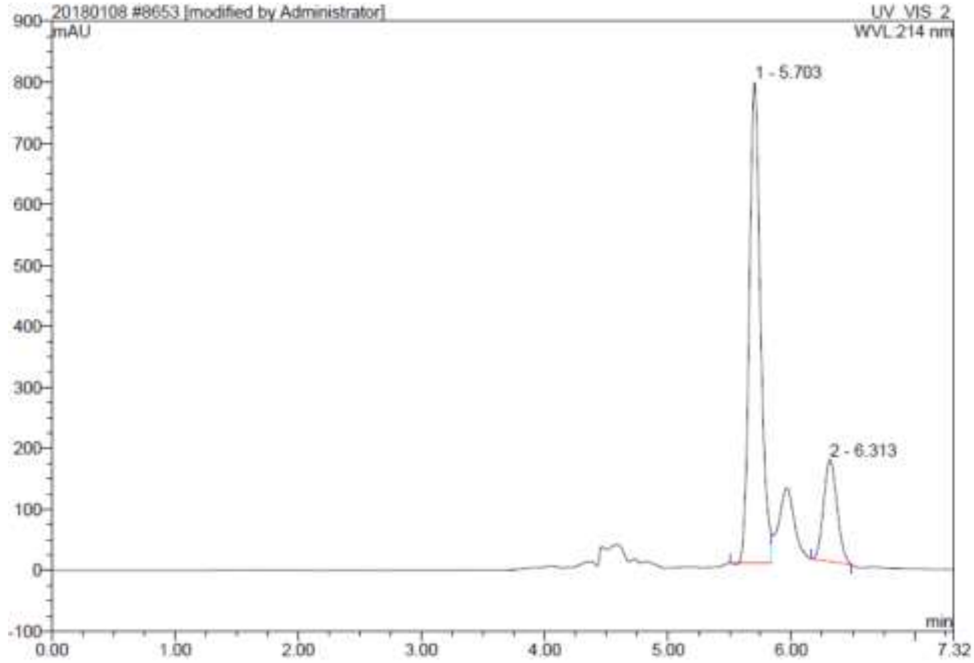

No.	Ret.Time min	Peak Name	Height mAU	Area mAU*min	Rel.Area %	Amount	Type
1	5.70	n.a.	788.589	82.741	79.76	n.a.	BM *
2	6.31	n.a.	167.408	20.996	20.24	n.a.	BMB*
Total:			955.997	103.737	100.00	0.000	

Supplementary Figure 281. Chiral HPLC analysis of (*rac*)-**3ca**.

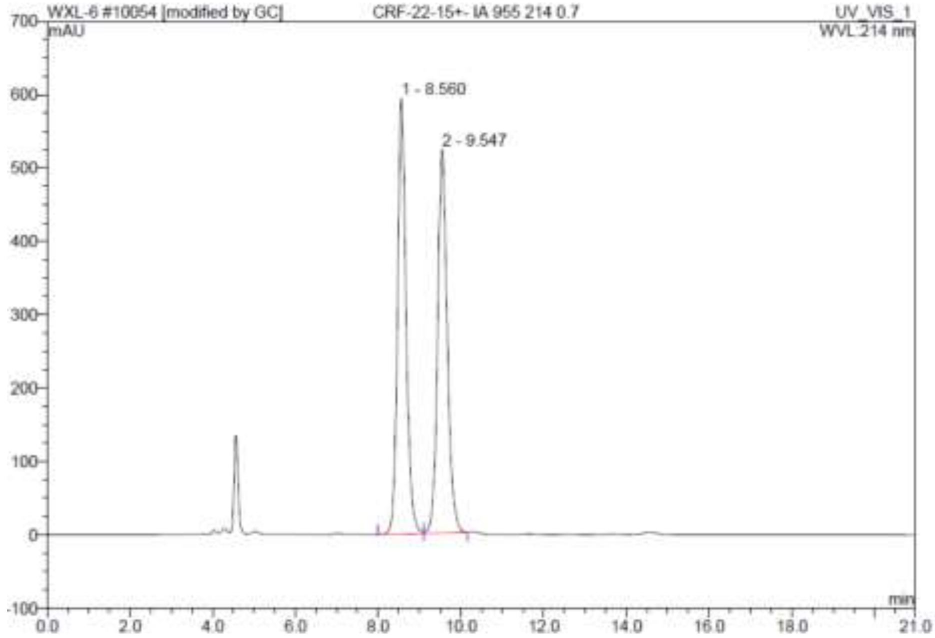

No.	Ret.Time min	Peak Name	Height mAU	Area mAU*min	Rel.Area %	Amount	Type
1	8.56	n.a.	593.778	142.168	50.15	n.a.	BM *
2	9.55	n.a.	523.046	141.299	49.85	n.a.	MB*
Total:			1116.824	283.467	100.00	0.000	

Supplementary Figure 282. Chiral HPLC analysis of (*S*)-**3ca** from asymmetric reaction (Method **B**).

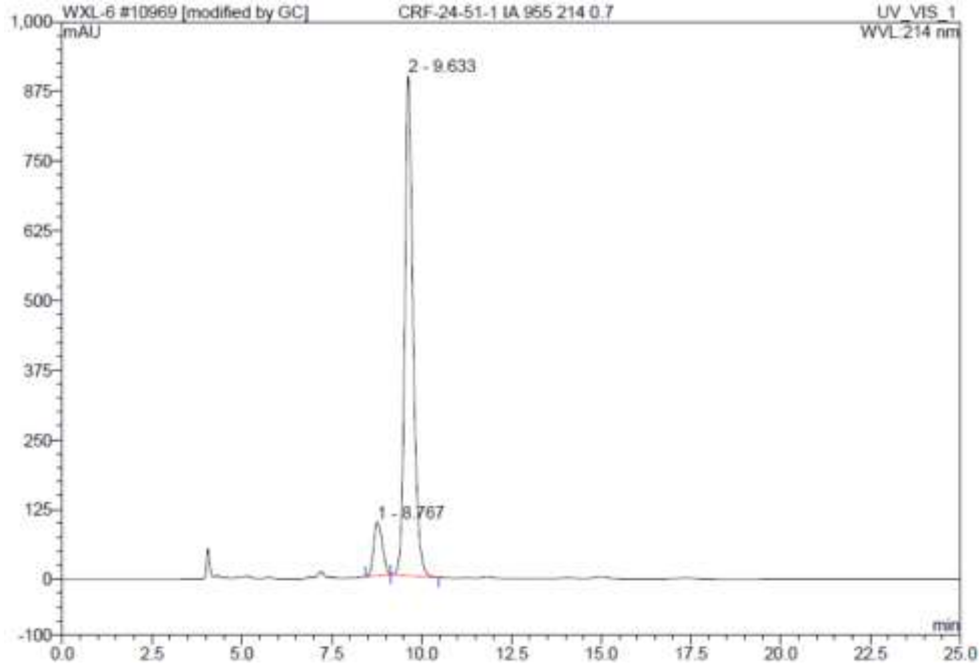

No.	Ret.Time min	Peak Name	Height mAU	Area mAU*min	Rel.Area %	Amount	Type
1	8.77	n.a.	95.778	26.706	9.94	n.a.	BMb*
2	9.63	n.a.	898.044	242.036	90.06	n.a.	bMB*
Total:			993.822	268.742	100.00	0.000	

Supplementary Figure 283. Chiral HPLC analysis of (*rac*)-**3aa**.

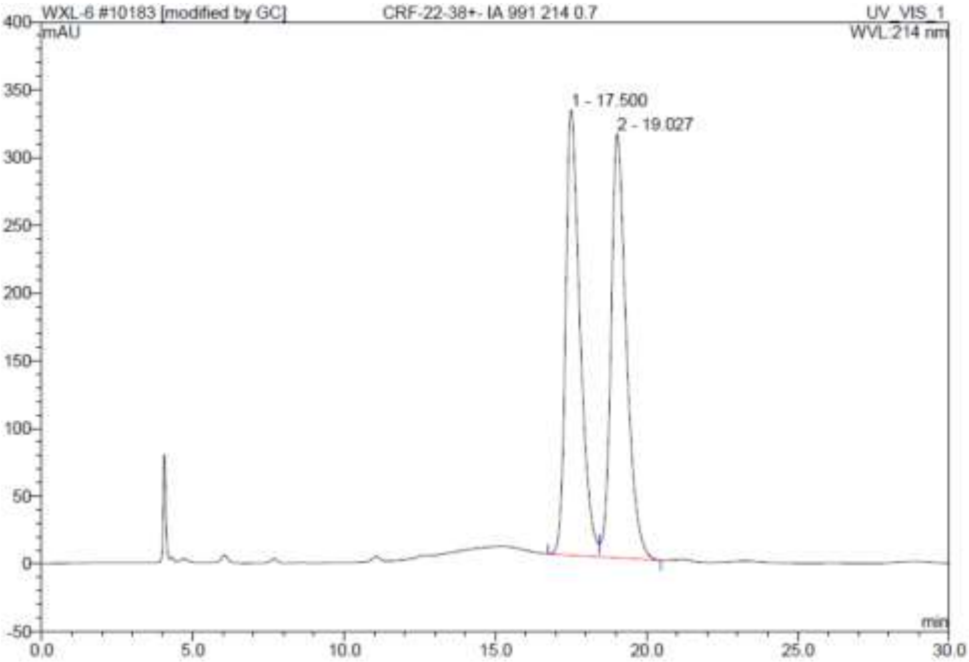

No.	Ret.Time min	Peak Name	Height mAU	Area mAU*min	Rel.Area %	Amount	Type
1	17.50	n.a.	328.797	181.125	49.60	n.a.	BM *
2	19.03	n.a.	313.292	184.063	50.40	n.a.	MB
Total:			642.089	365.188	100.00	0.000	

Supplementary Figure 284. Chiral HPLC analysis of (*S*)-**3aa** from asymmetric reaction (Method **A**).

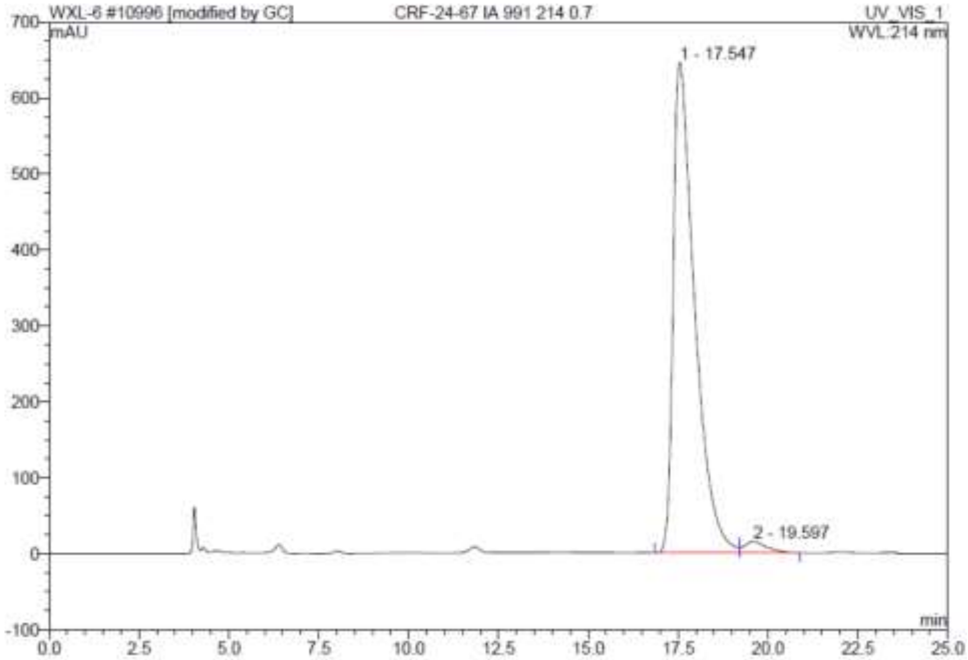

No.	Ret.Time min	Peak Name	Height mAU	Area mAU*min	Rel.Area %	Amount	Type
1	17.55	n.a.	645.869	446.161	97.60	n.a.	BM
2	19.60	n.a.	14.894	10.955	2.40	n.a.	MB
Total:			660.762	457.116	100.00	0.000	

Supplementary Figure 285. Chiral HPLC analysis of (*rac*)-**3da**.

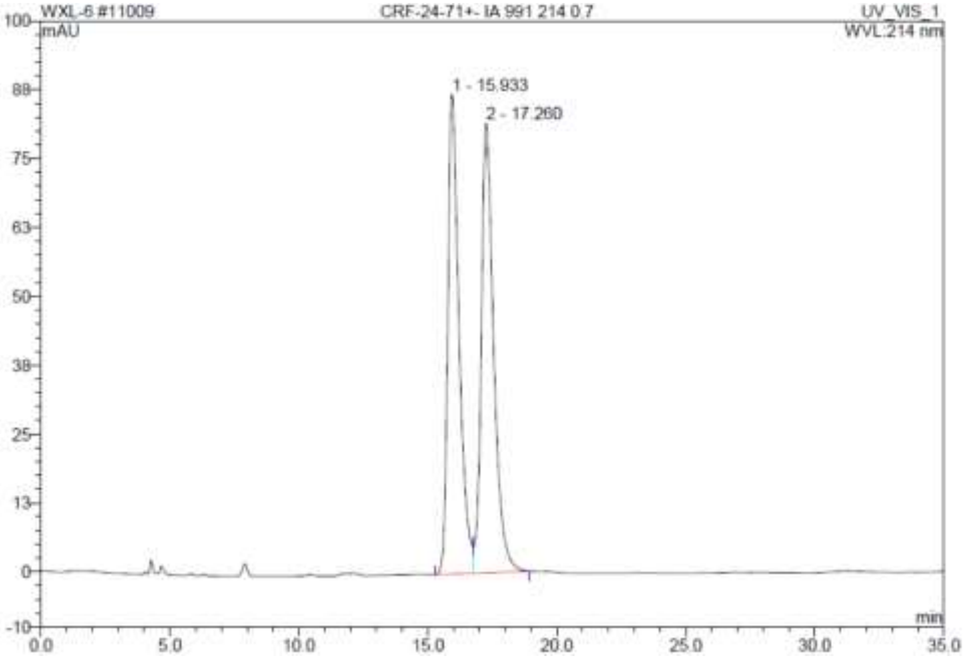

No.	Ret.Time min	Peak Name	Height mAU	Area mAU*min	Rel.Area %	Amount	Type
1	15.93	n.a.	87.055	44.152	49.23	n.a.	BM
2	17.26	n.a.	81.676	45.530	50.77	n.a.	MB
Total:			168.731	89.682	100.00	0.000	

Supplementary Figure 286. Chiral HPLC analysis of (*S*)-**3da** from asymmetric reaction (Method **A**).

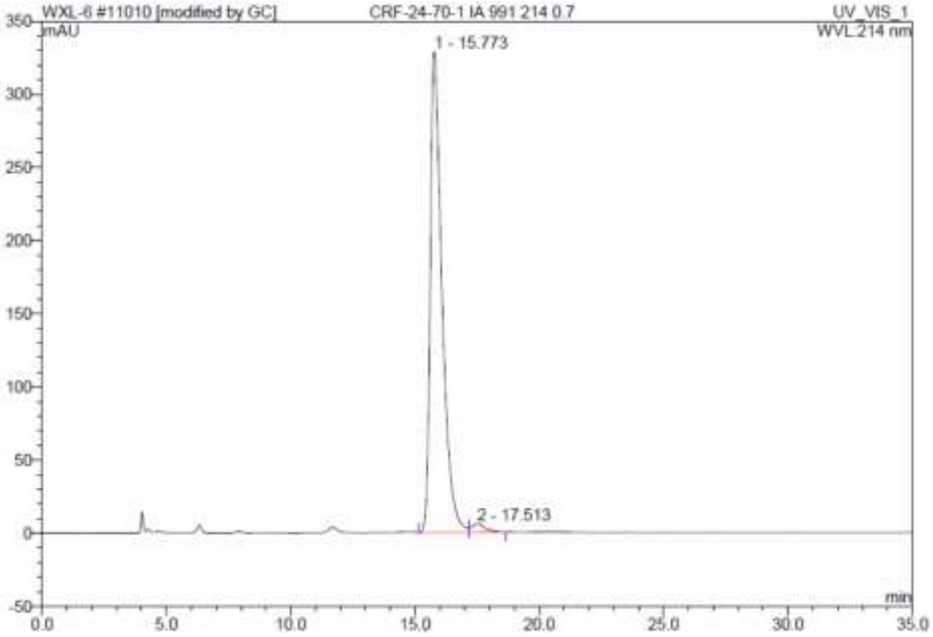

No.	Ret.Time min	Peak Name	Height mAU	Area mAU*min	Rel.Area %	Amount	Type
1	15.77	n.a.	328.610	178.912	97.96	n.a.	BM
2	17.51	n.a.	5.904	3.730	2.04	n.a.	MB
Total:			334.514	182.642	100.00	0.000	

Supplementary Figure 287. Chiral HPLC analysis of (*rac*)-**3ea**.

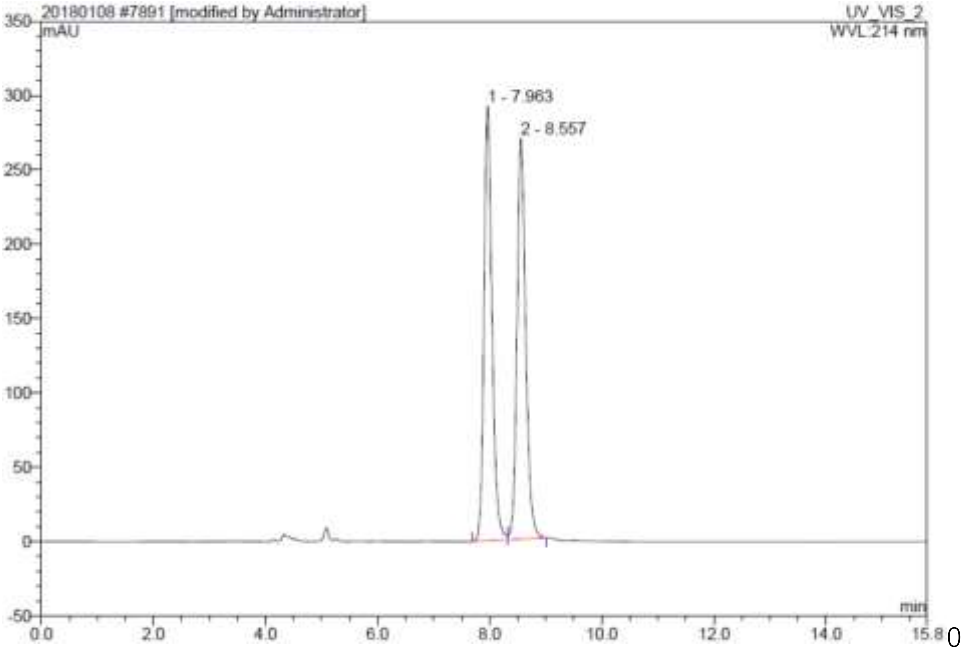

No.	Ret.Time min	Peak Name	Height mAU	Area mAU*min	Rel.Area %	Amount	Type
1	7.96	n.a.	292.569	48.730	50.11	n.a.	BM
2	8.56	n.a.	269.979	48.520	49.89	n.a.	MB
Total:			562.548	97.250	100.00	0.000	

Supplementary Figure 288. Chiral HPLC analysis of (*S*)-**3ea** from asymmetric reaction (Method **A**).

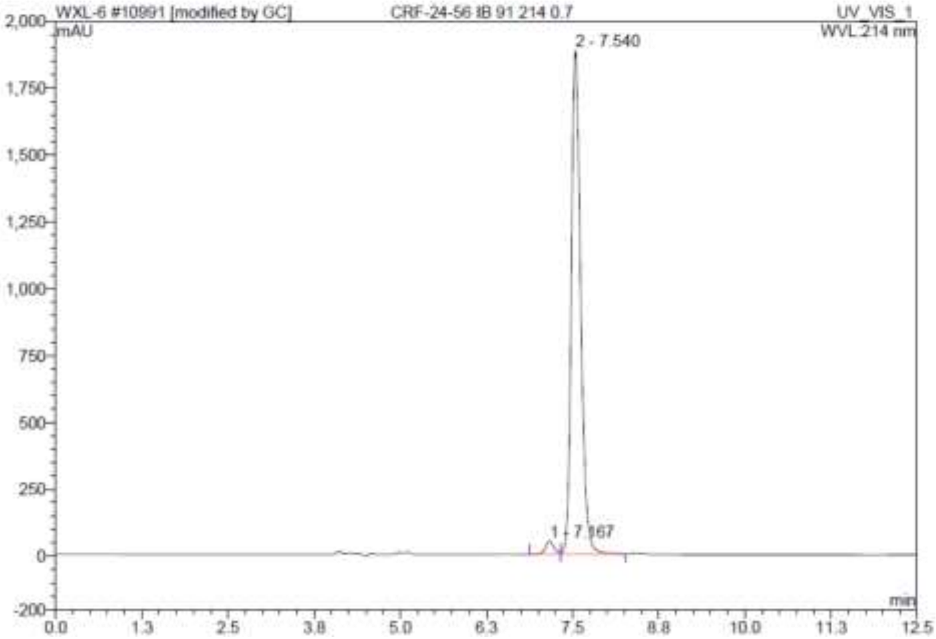

No.	Ret.Time min	Peak Name	Height mAU	Area mAU*min	Rel.Area %	Amount	Type
1	7.17	n.a.	48.796	6.908	2.28	n.a.	BM
2	7.54	n.a.	1885.334	296.037	97.72	n.a.	MB*
Total:			1934.129	302.945	100.00	0.000	

Supplementary Figure 289. Chiral HPLC analysis of (*rac*)-**3fa**.

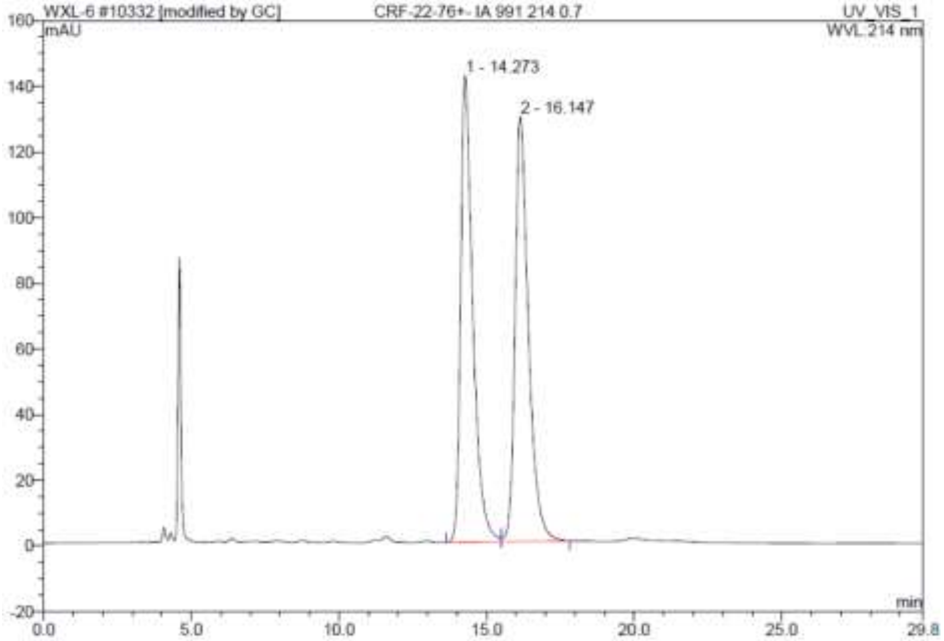

No.	Ret.Time min	Peak Name	Height mAU	Area mAU*min	Rel.Area %	Amount	Type
1	14.27	n.a.	142.123	68.051	50.00	n.a.	BM
2	16.15	n.a.	129.495	68.055	50.00	n.a.	MB
Total:			271.618	136.106	100.00	0.000	

Supplementary Figure 290. Chiral HPLC analysis of (*S*)-**3fa** from asymmetric reaction (Method **A**).

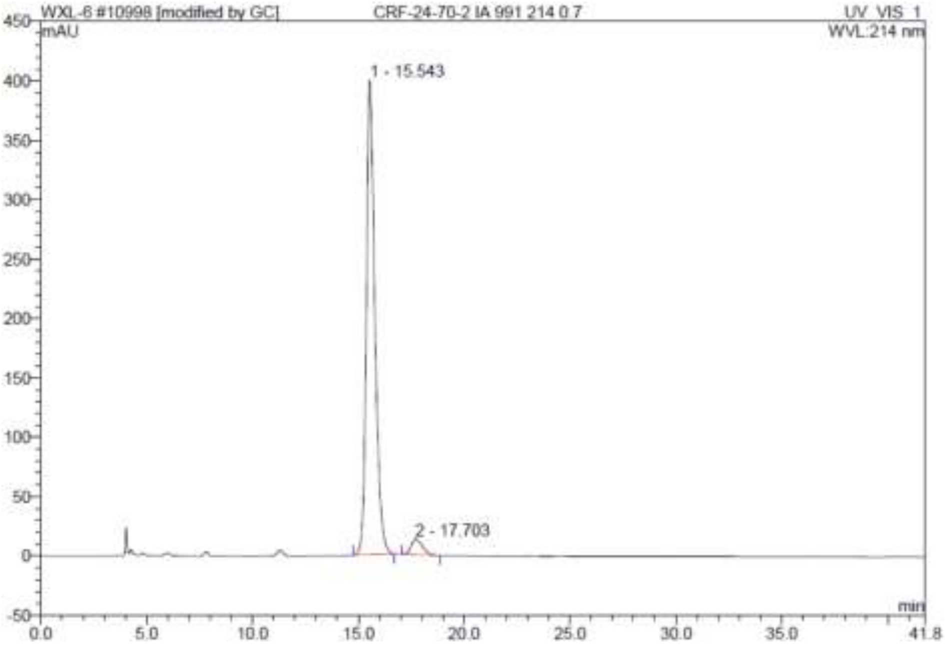

No.	Ret.Time min	Peak Name	Height mAU	Area mAU*min	Rel.Area %	Amount	Type
1	15.54	n.a.	399.650	185.481	95.81	n.a.	BMB
2	17.70	n.a.	13.095	8.107	4.19	n.a.	BMB
Total:			412.744	193.588	100.00	0.000	

Supplementary Figure 291. Chiral HPLC analysis of (*rac*)-**3ga**.

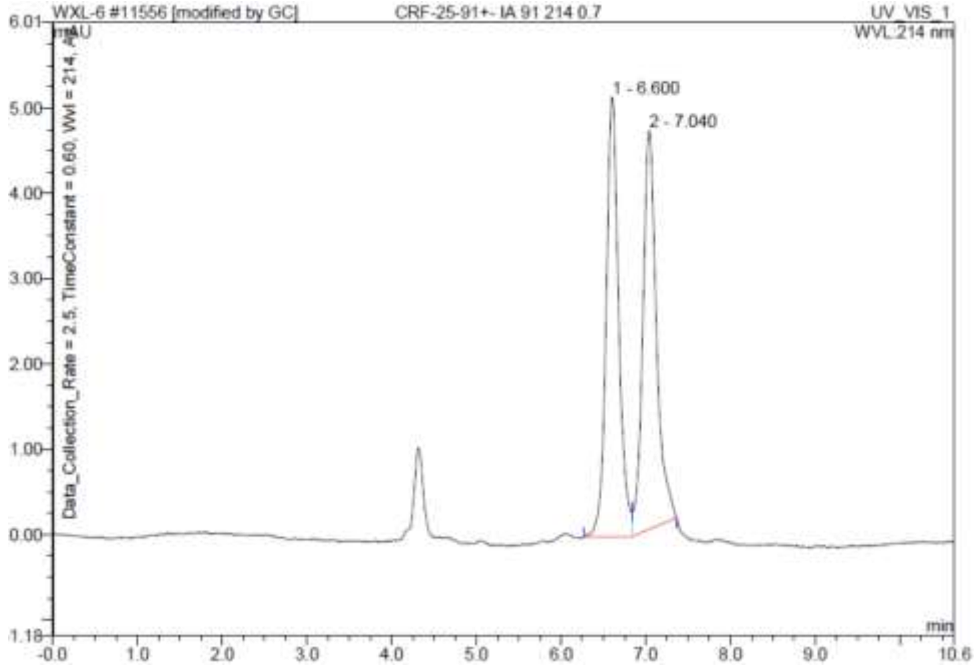

No.	Ret.Time min	Peak Name	Height mAU	Area mAU*min	Rel.Area %	Amount	Type
1	6.60	n.a.	5.156	0.886	50.26	n.a.	BM *
2	7.04	n.a.	4.678	0.877	49.74	n.a.	MB*
Total:			9.834	1.764	100.00	0.000	

Supplementary Figure 292. Chiral HPLC analysis of (*S*)-**3ga** from asymmetric reaction (Method **A**).

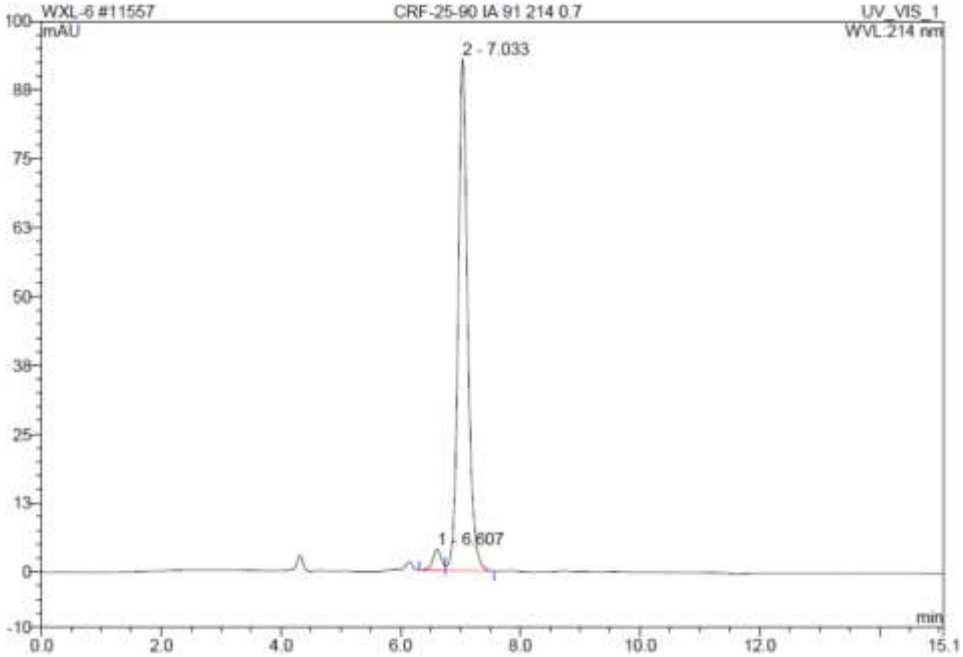

No.	Ret.Time min	Peak Name	Height mAU	Area mAU*min	Rel.Area %	Amount	Type
1	6.61	n.a.	3.916	0.660	3.69	n.a.	BM
2	7.03	n.a.	92.983	17.221	96.31	n.a.	MB
Total:			96.899	17.881	100.00	0.000	

Supplementary Figure 293. Chiral HPLC analysis of (*rac*)-**3ha**.

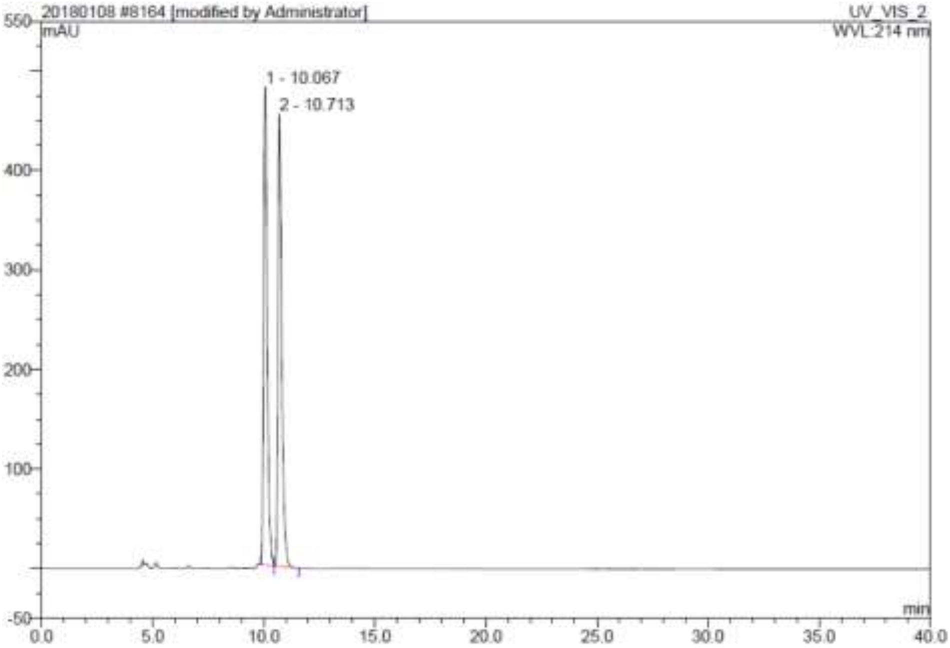

No.	Ret.Time min	Peak Name	Height mAU	Area mAU*min	Rel.Area %	Amount	Type
1	10.07	n.a.	480.670	88.094	49.82	n.a.	BM *
2	10.71	n.a.	455.037	88.719	50.18	n.a.	MB*
Total:			935.707	176.813	100.00	0.000	

Supplementary Figure 294. Chiral HPLC analysis of (*S*)-**3ha** from asymmetric reaction (Method **A**).

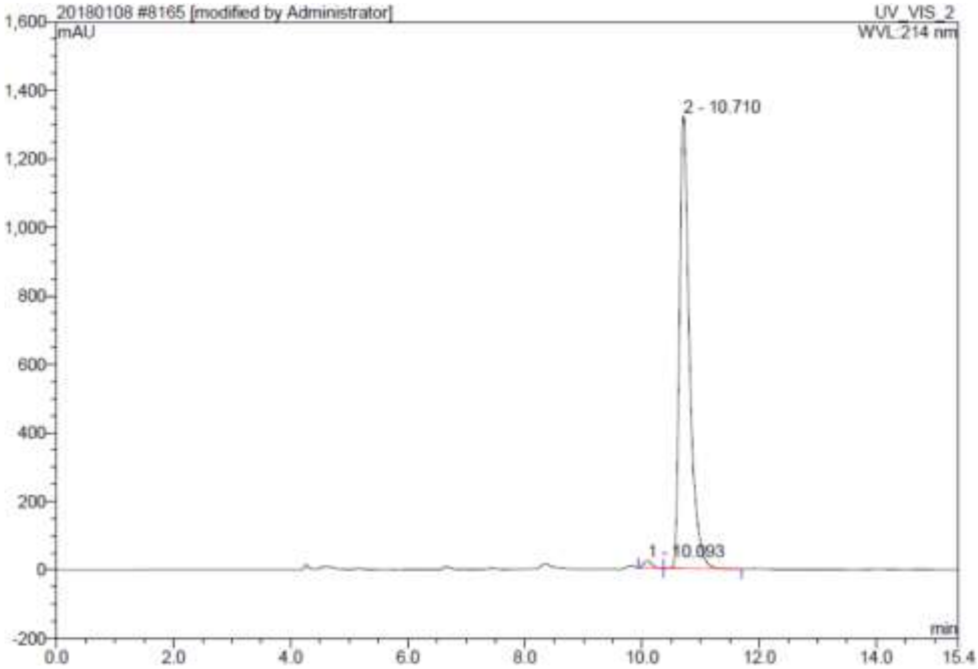

No.	Ret.Time min	Peak Name	Height mAU	Area mAU*min	Rel.Area %	Amount	Type
1	10.09	n.a.	21.285	3.196	1.20	n.a.	BM *
2	10.71	n.a.	1321.552	262.472	98.80	n.a.	MB*
Total:			1342.837	265.668	100.00	0.000	

Supplementary Figure 295. Chiral HPLC analysis of (*rac*)-**3ia**.

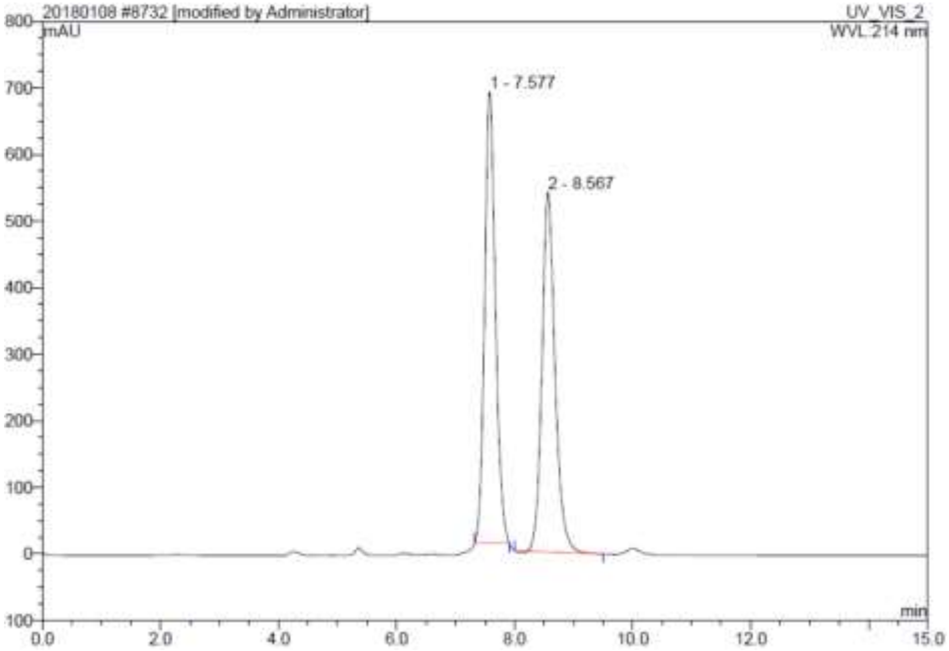

No.	Ret.Time min	Peak Name	Height mAU	Area mAU*min	Rel.Area %	Amount	Type
1	7.58	n.a.	677.929	142.898	50.86	n.a.	BMB*
2	8.57	n.a.	540.214	138.054	49.14	n.a.	BMB*
Total:			1218.143	280.952	100.00	0.000	

Supplementary Figure 296. Chiral HPLC analysis of (*S*)-**3ia** from asymmetric reaction (Method **A**).

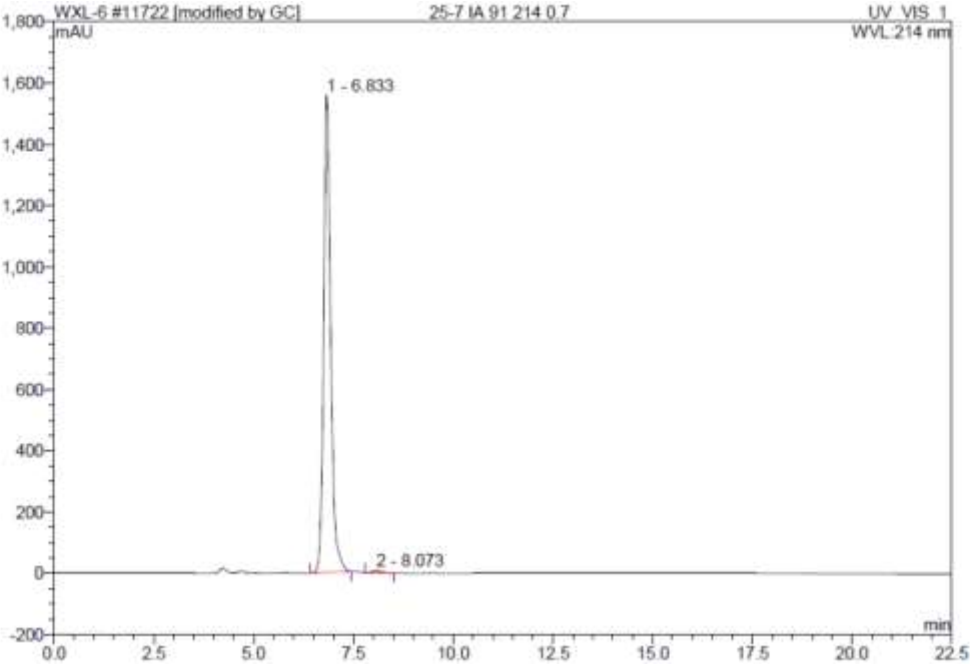

No.	Ret.Time min	Peak Name	Height mAU	Area mAU*min	Rel.Area %	Amount	Type
1	6.83	n.a.	1559.963	321.702	99.37	n.a.	BMB
2	8.07	n.a.	8.077	2.053	0.63	n.a.	BMB
Total:			1568.040	323.756	100.00	0.000	

Supplementary Figure 297. Chiral HPLC analysis of (*rac*)-**3ja**.

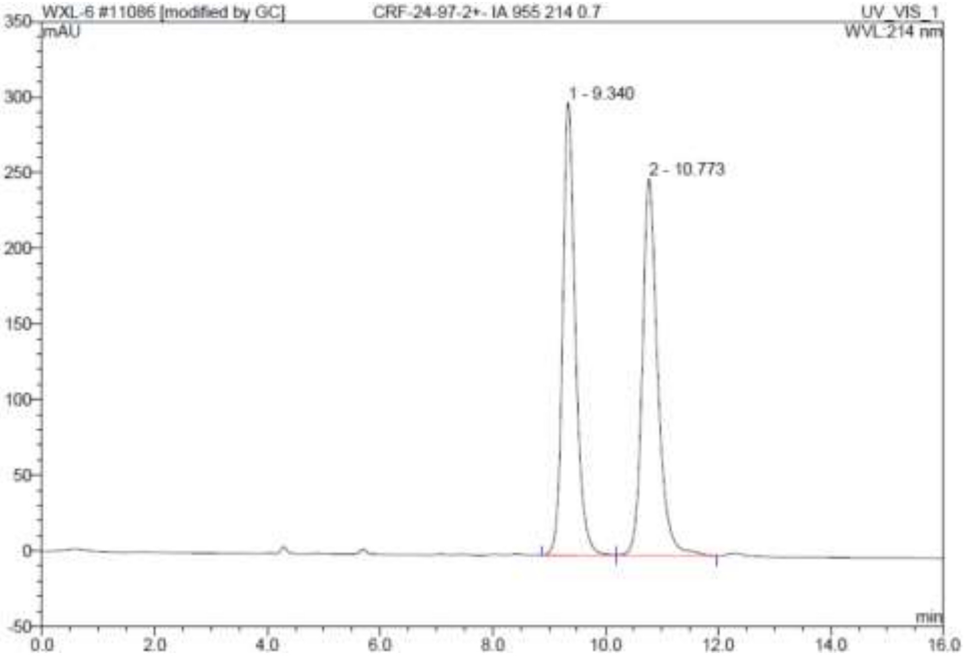

No.	Ret.Time min	Peak Name	Height mAU	Area mAU*min	Rel.Area %	Amount	Type
1	9.34	n.a.	299.499	78.451	49.83	n.a.	BM
2	10.77	n.a.	249.117	78.983	50.17	n.a.	MB*
Total:			548.616	157.434	100.00	0.000	

Supplementary Figure 298. Chiral HPLC analysis of (*S*)-**3ja** from asymmetric reaction (Method **A**).

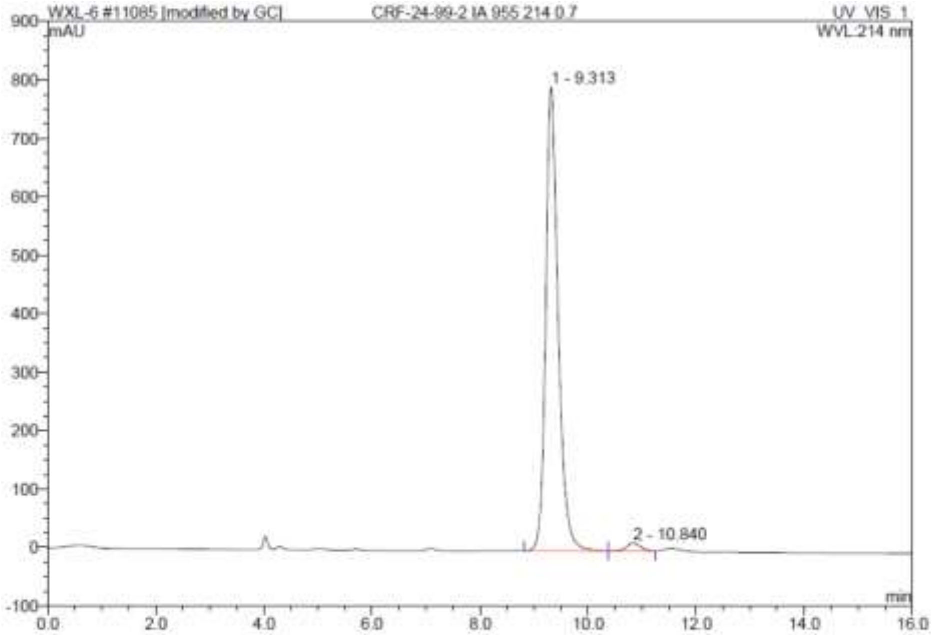

No.	Ret.Time min	Peak Name	Height mAU	Area mAU*min	Rel.Area %	Amount	Type
1	9.31	n.a.	794.573	211.289	98.15	n.a.	BMb
2	10.84	n.a.	13.472	3.973	1.85	n.a.	bMB*
Total:			808.044	215.263	100.00	0.000	

Supplementary Figure 299. Chiral HPLC analysis of (*rac*)-**3ka**.

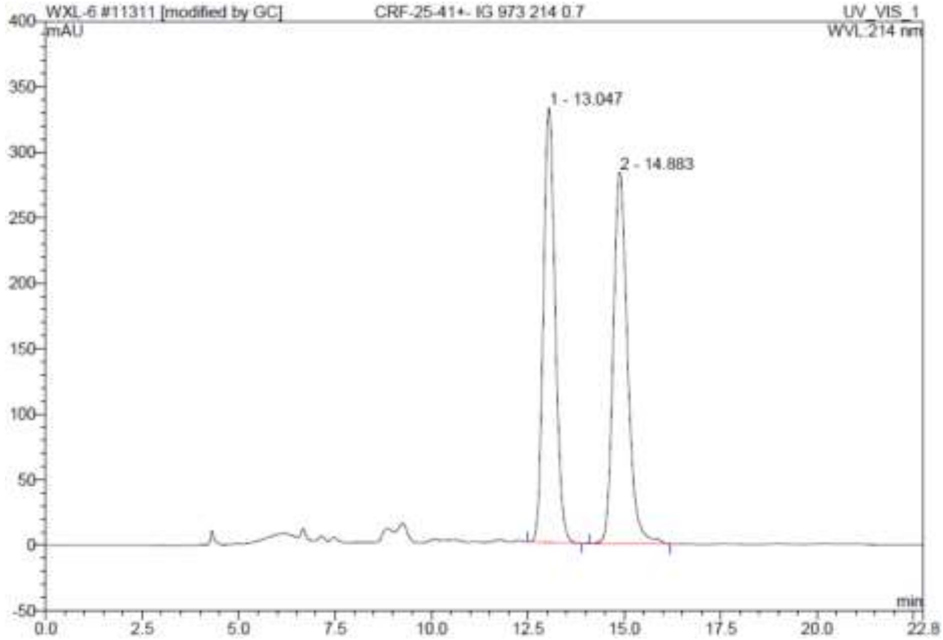

No.	Ret.Time min	Peak Name	Height mAU	Area mAU*min	Rel.Area %	Amount	Type
1	13.05	n.a.	332.071	117.454	49.07	n.a.	BMB
2	14.88	n.a.	283.395	121.922	50.93	n.a.	BMB
Total:			615.466	239.376	100.00	0.000	

Supplementary Figure 300. Chiral HPLC analysis of (*S*)-**3ka** from asymmetric reaction (Method **A**).

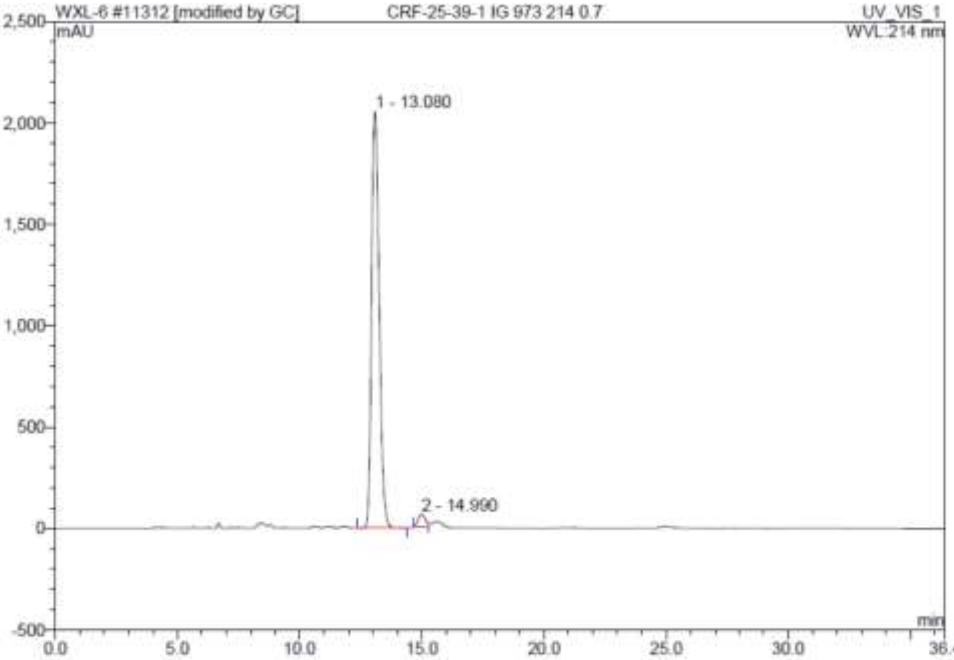

No.	Ret.Time min	Peak Name	Height mAU	Area mAU*min	Rel.Area %	Amount	Type
1	13.08	n.a.	2053.678	742.409	97.16	n.a.	BMB*
2	14.99	n.a.	61.259	21.664	2.84	n.a.	BM *
Total:			2114.937	764.073	100.00	0.000	

Supplementary Figure 301. Chiral HPLC analysis of (*rac*)-**3la**.

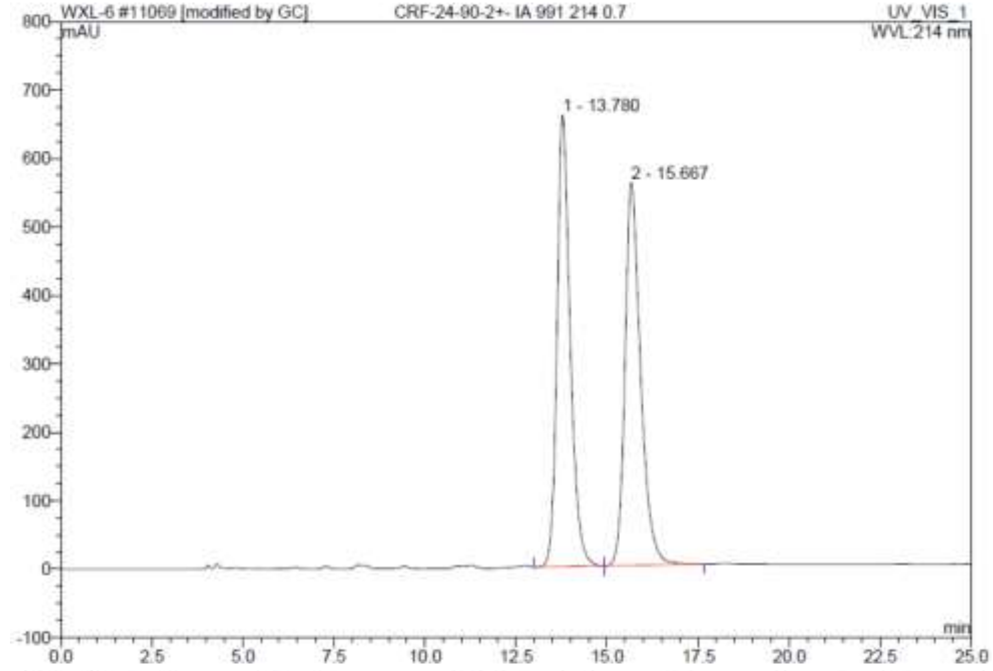

No.	Ret.Time min	Peak Name	Height mAU	Area mAU*min	Rel.Area %	Amount	Type
1	13.78	n.a.	660.037	276.403	49.74	n.a.	BM *
2	15.67	n.a.	559.254	279.288	50.26	n.a.	MB*
Total:			1219.291	555.691	100.00	0.000	

Supplementary Figure 302. Chiral HPLC analysis of (*S*)-**3la** from asymmetric reaction (Method **A**).

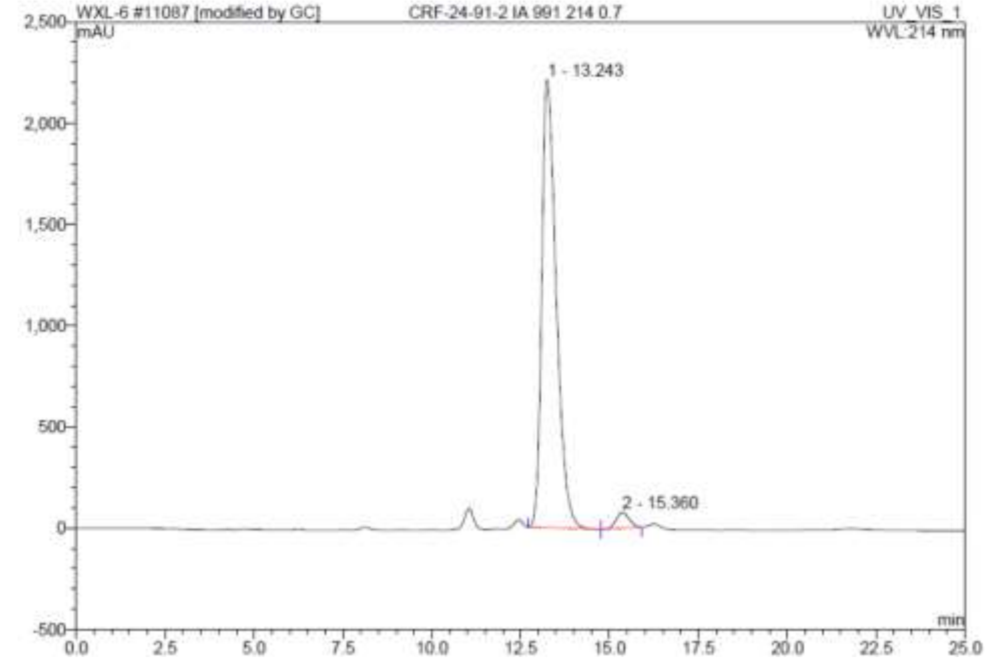

No.	Ret.Time min	Peak Name	Height mAU	Area mAU*min	Rel.Area %	Amount	Type
1	13.24	n.a.	2212.200	1044.271	96.96	n.a.	BMB*
2	15.36	n.a.	78.707	32.711	3.04	n.a.	BMB*
Total:			2290.907	1076.982	100.00	0.000	

Supplementary Figure 303. Chiral HPLC analysis of (*rac*)-**3ma**.

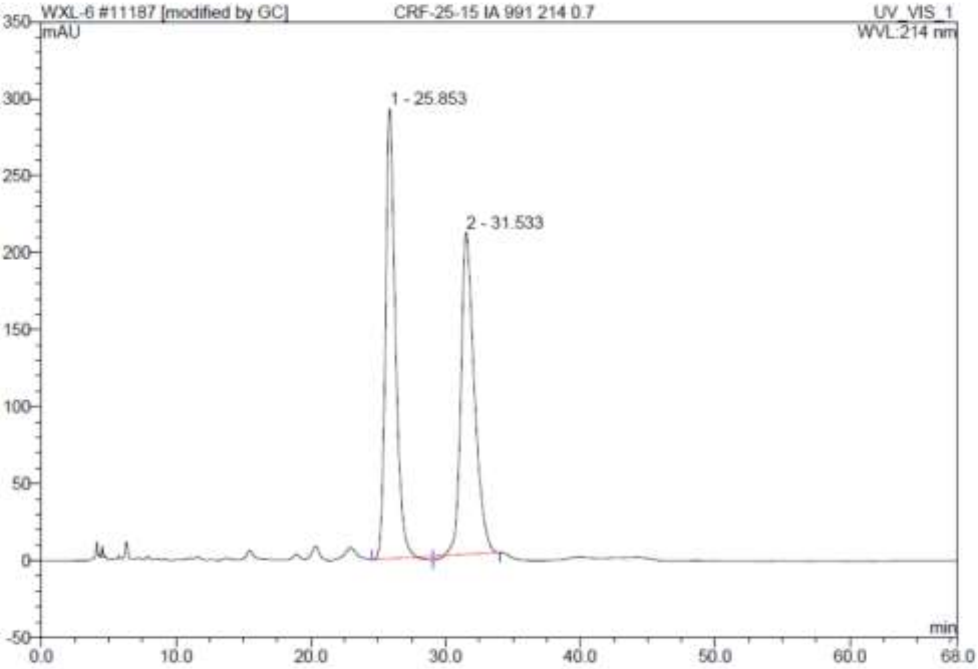

No.	Ret.Time min	Peak Name	Height mAU	Area mAU*min	Rel.Area %	Amount	Type
1	25.85	n.a.	292.564	247.663	50.77	n.a.	BM *
2	31.53	n.a.	209.309	240.163	49.23	n.a.	MB*
Total:			501.873	487.825	100.00	0.000	

Supplementary Figure 304. Chiral HPLC analysis of (*S*)-**3ma** from asymmetric reaction (Method **A**).

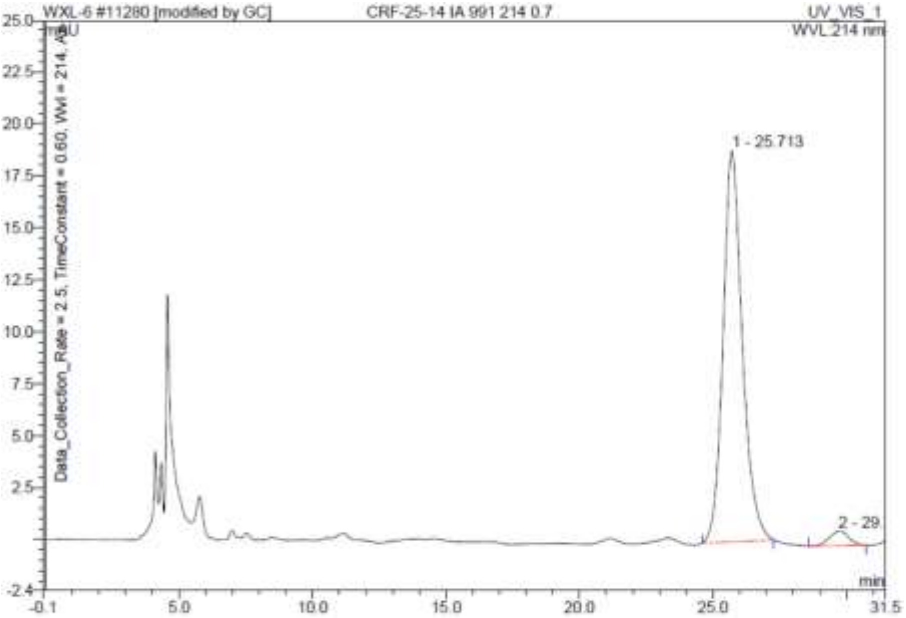

No.	Ret.Time min	Peak Name	Height mAU	Area mAU*min	Rel.Area %	Amount	Type
1	25.71	n.a.	18.815	15.715	96.41	n.a.	BMB
2	29.71	n.a.	0.705	0.586	3.59	n.a.	BMB*
Total:			19.519	16.300	100.00	0.000	

Supplementary Figure 305. Chiral HPLC analysis of (*rac*)-**3na**.

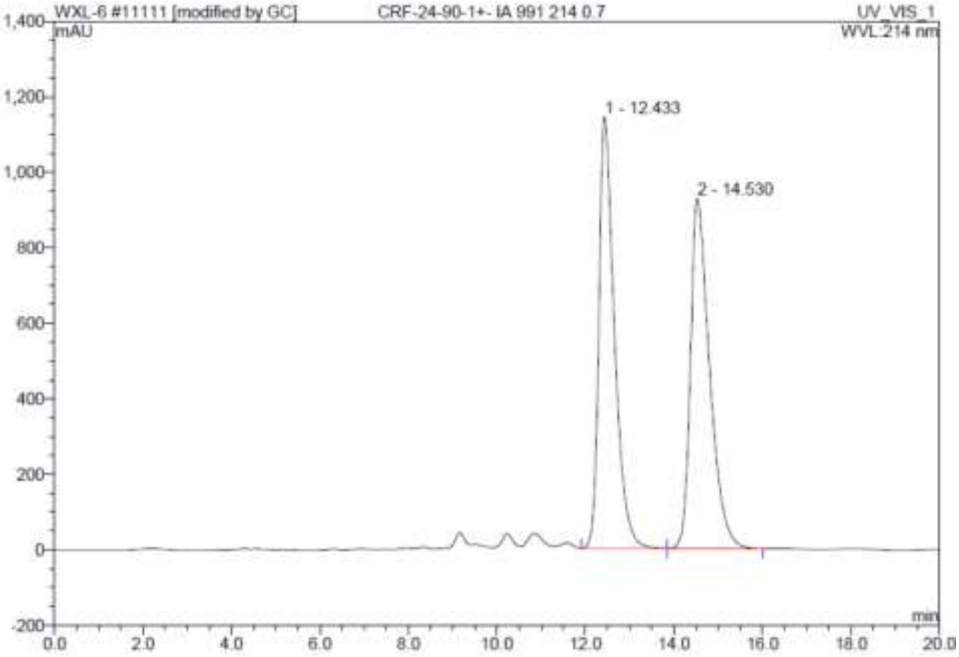

No.	Ret.Time min	Peak Name	Height mAU	Area mAU*min	Rel.Area %	Amount	Type
1	12.43	n.a.	1143.508	463.444	49.86	n.a.	BM *
2	14.53	n.a.	927.451	466.120	50.14	n.a.	MB*
Total:			2070.959	929.564	100.00	0.000	

Supplementary Figure 306. Chiral HPLC analysis of (*S*)-**3na** from asymmetric reaction (Method **A**).

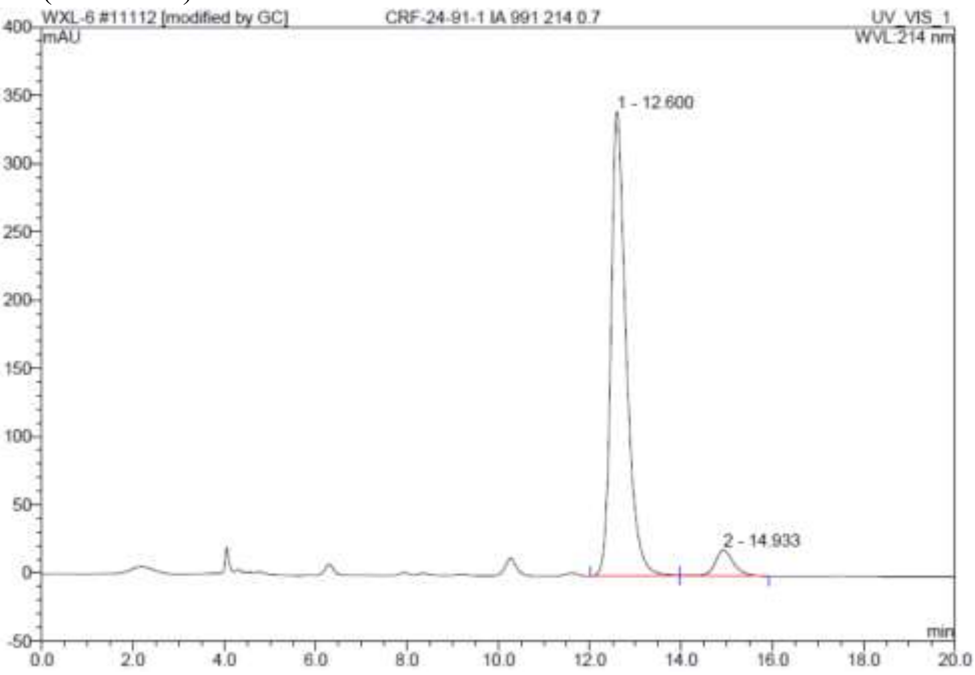

No.	Ret.Time min	Peak Name	Height mAU	Area mAU*min	Rel.Area %	Amount	Type
1	12.60	n.a.	340.655	131.992	93.32	n.a.	BM
2	14.93	n.a.	19.304	9.450	6.68	n.a.	MB
Total:			359.959	141.443	100.00	0.000	

Supplementary Figure 307. Chiral HPLC analysis of (*rac*)-**3oa**.

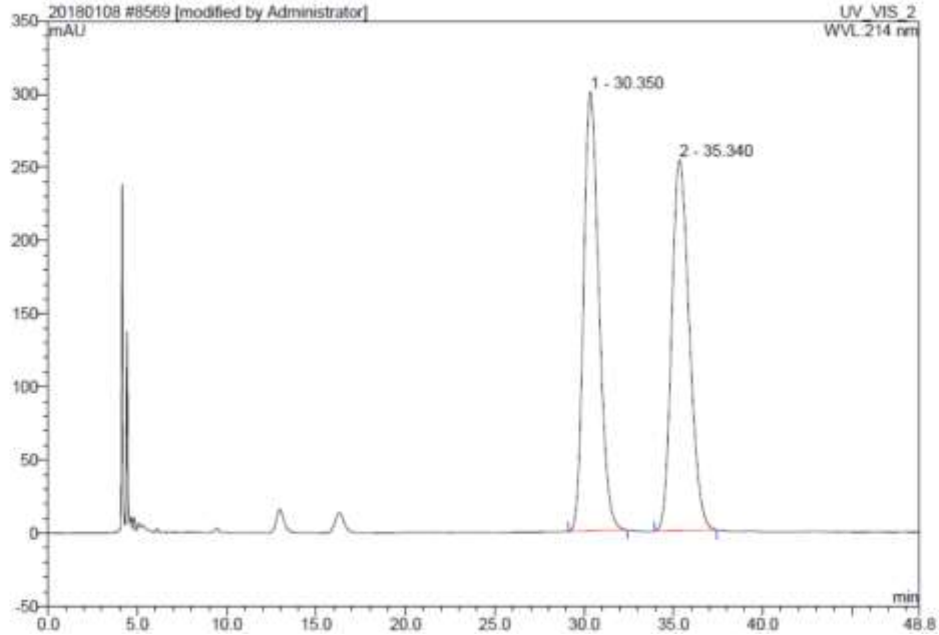

No.	Ret.Time min	Peak Name	Height mAU	Area mAU*min	Rel.Area %	Amount	Type
1	30.35	n.a.	300.260	297.543	50.51	n.a.	BMB
2	35.34	n.a.	253.620	291.518	49.49	n.a.	BMB
Total:			553.879	589.061	100.00	0.000	

Supplementary Figure 308. Chiral HPLC analysis of (*S*)-**3oa** from asymmetric reaction (Method **A**).

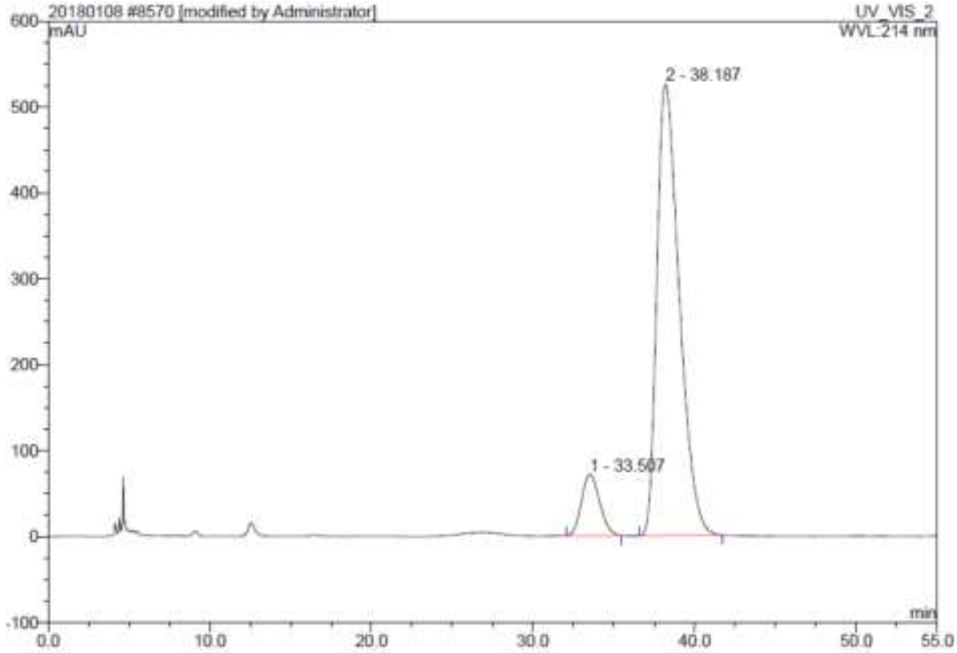

No.	Ret.Time min	Peak Name	Height mAU	Area mAU*min	Rel.Area %	Amount	Type
1	33.51	n.a.	71.401	92.696	9.91	n.a.	BMB
2	38.19	n.a.	525.356	842.994	90.09	n.a.	BMB
Total:			596.758	935.691	100.00	0.000	

Supplementary Figure 309. Chiral HPLC analysis of (*rac*)-**3pa**.

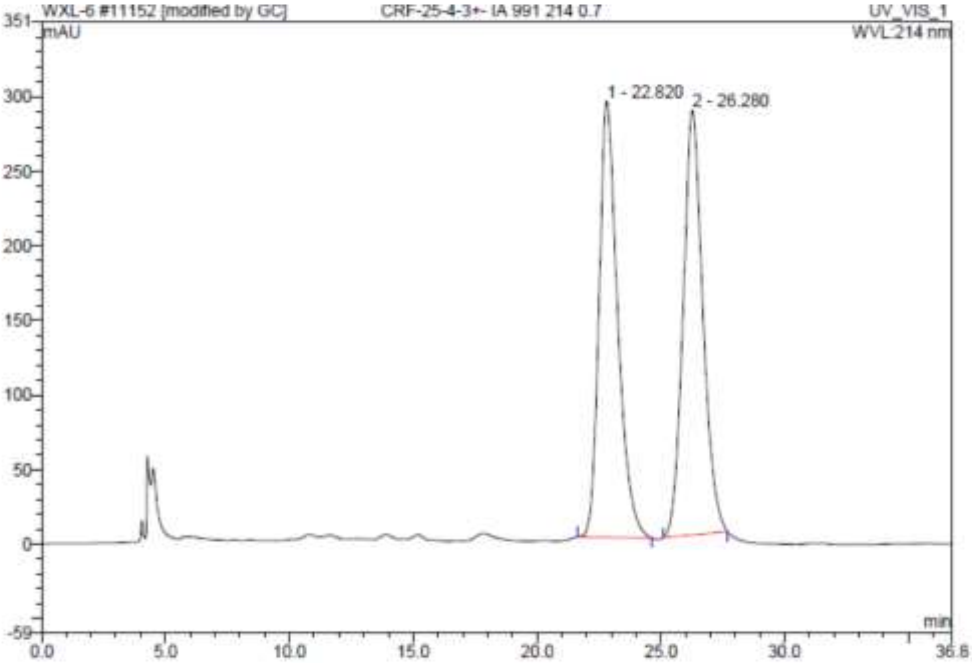

No.	Ret.Time min	Peak Name	Height mAU	Area mAU*min	Rel.Area %	Amount	Type
1	22.82	n.a.	292.752	254.591	49.19	n.a.	BMB*
2	26.28	n.a.	284.795	263.006	50.81	n.a.	BMB*
Total:			577.548	517.598	100.00	0.000	

Supplementary Figure 310. Chiral HPLC analysis of (*S*)-**3pa** from asymmetric reaction (Method **A**).

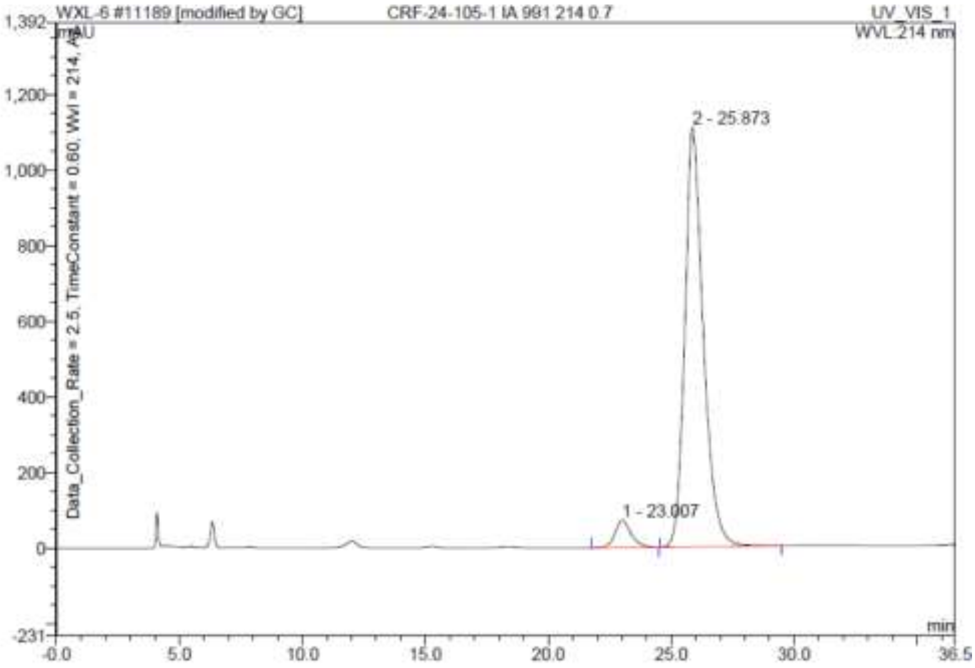

No.	Ret.Time min	Peak Name	Height mAU	Area mAU*min	Rel.Area %	Amount	Type
1	23.01	n.a.	72.704	54.808	5.36	n.a.	BMB
2	25.87	n.a.	1109.973	967.722	94.64	n.a.	BMB
Total:			1182.677	1022.530	100.00	0.000	

Supplementary Figure 311. Chiral HPLC analysis of (*rac*)-**3qa**.

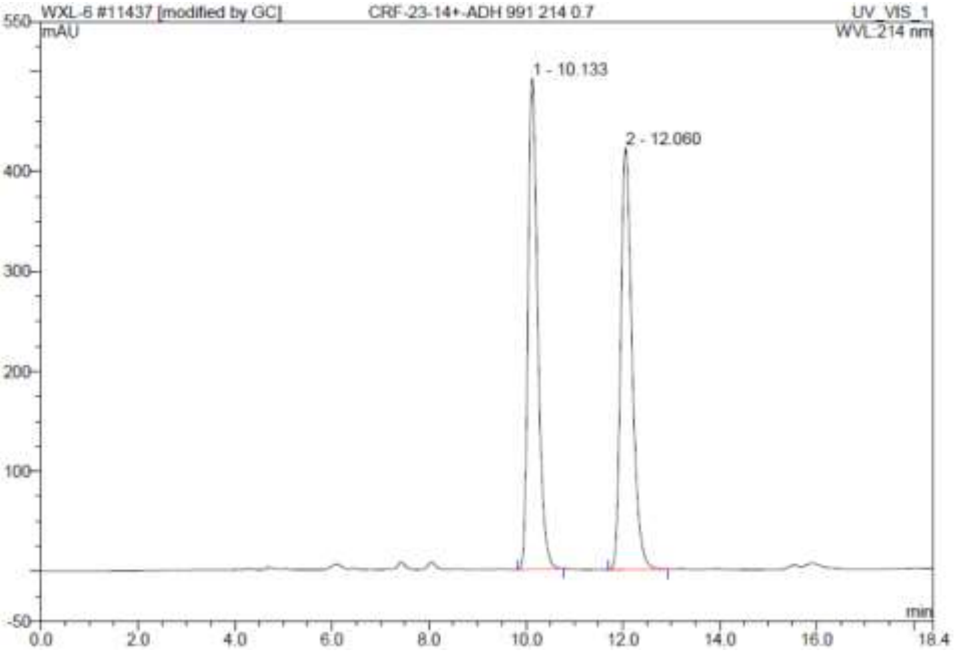

No.	Ret.Time min	Peak Name	Height mAU	Area mAU*min	Rel.Area %	Amount	Type
1	10.13	n.a.	491.380	113.662	49.84	n.a.	BMB*
2	12.06	n.a.	421.904	114.402	50.16	n.a.	BMB*
Total:			913.283	228.063	100.00	0.000	

Supplementary Figure 312. Chiral HPLC analysis of (*S*)-**3qa** from asymmetric reaction (Method **A**).

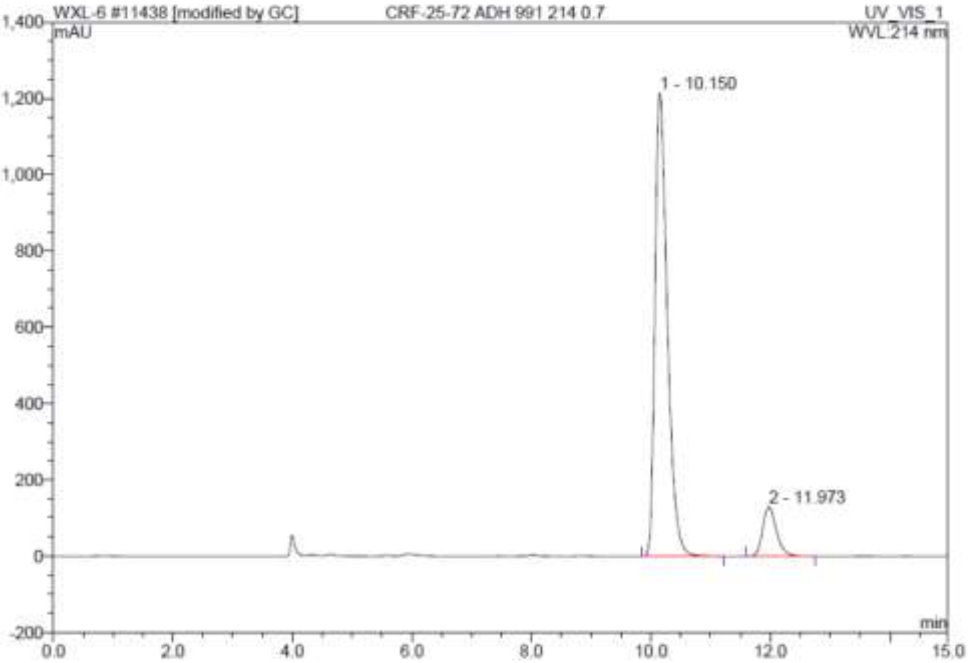

No.	Ret.Time min	Peak Name	Height mAU	Area mAU*min	Rel.Area %	Amount	Type
1	10.15	n.a.	1214.460	290.832	89.30	n.a.	BMB
2	11.97	n.a.	129.959	34.852	10.70	n.a.	BMB
Total:			1344.419	325.685	100.00	0.000	

Supplementary Figure 313. Chiral HPLC analysis of (*rac*)-**3ra**.

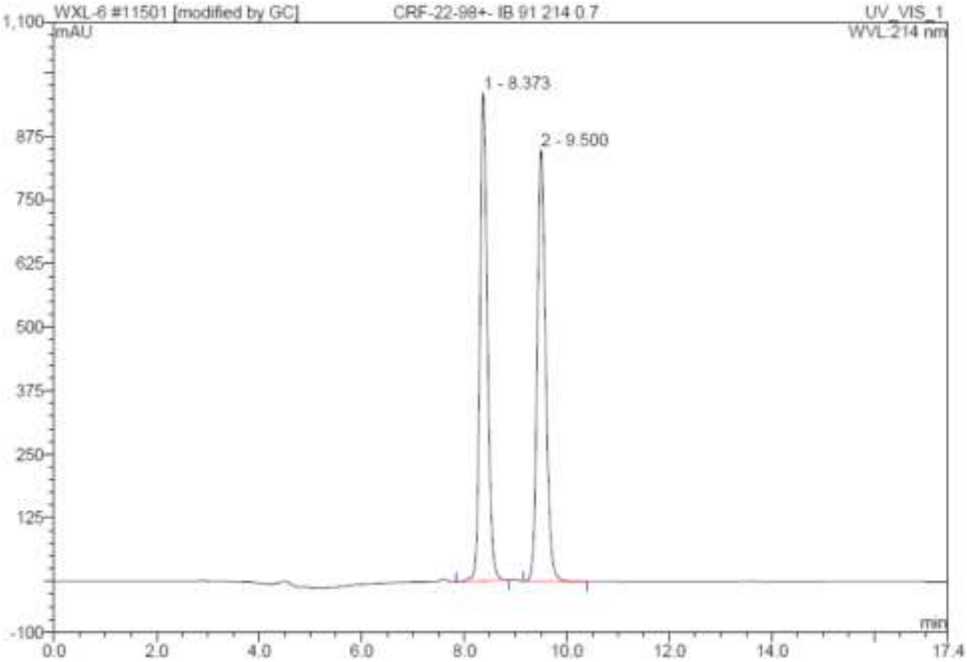

No.	Ret.Time min	Peak Name	Height mAU	Area mAU*min	Rel.Area %	Amount	Type
1	8.37	n.a.	959.970	163.190	50.08	n.a.	BMB*
2	9.50	n.a.	849.282	162.669	49.92	n.a.	BMB
Total:			1809.252	325.860	100.00	0.000	

Supplementary Figure 314. Chiral HPLC analysis of (*S*)-**3ra** from asymmetric reaction (Method **A**).

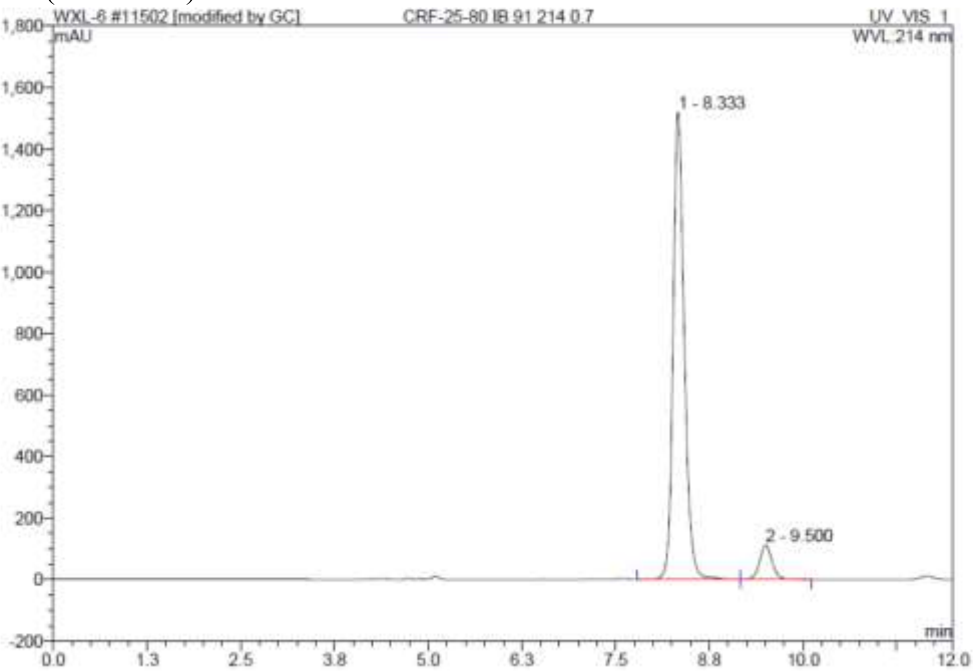

No.	Ret.Time min	Peak Name	Height mAU	Area mAU*min	Rel.Area %	Amount	Type
1	8.33	n.a.	1521.320	262.125	92.34	n.a.	BM
2	9.50	n.a.	111.383	21.736	7.66	n.a.	MB
Total:			1632.703	283.861	100.00	0.000	

Supplementary Figure 315. Chiral HPLC analysis of (*rac*)-**4ba**.

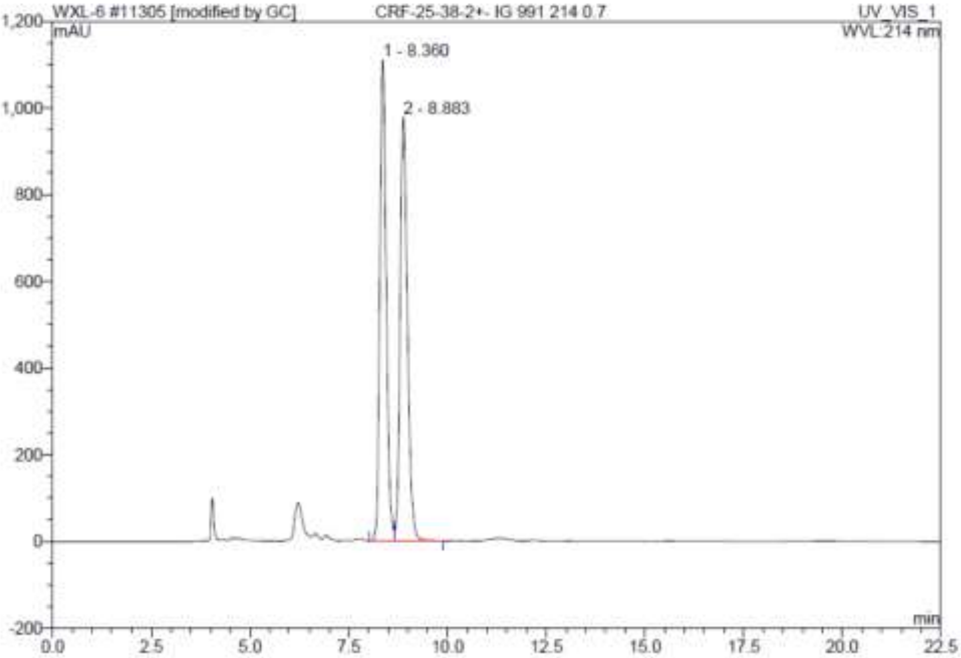

No.	Ret.Time min	Peak Name	Height mAU	Area mAU*min	Rel.Area %	Amount	Type
1	8.36	n.a.	1110.457	208.407	49.83	n.a.	BM
2	8.88	n.a.	977.679	209.802	50.17	n.a.	MB
Total:			2088.136	418.209	100.00	0.000	

Supplementary Figure 316. Chiral HPLC analysis of (*S*)-**4ba**.

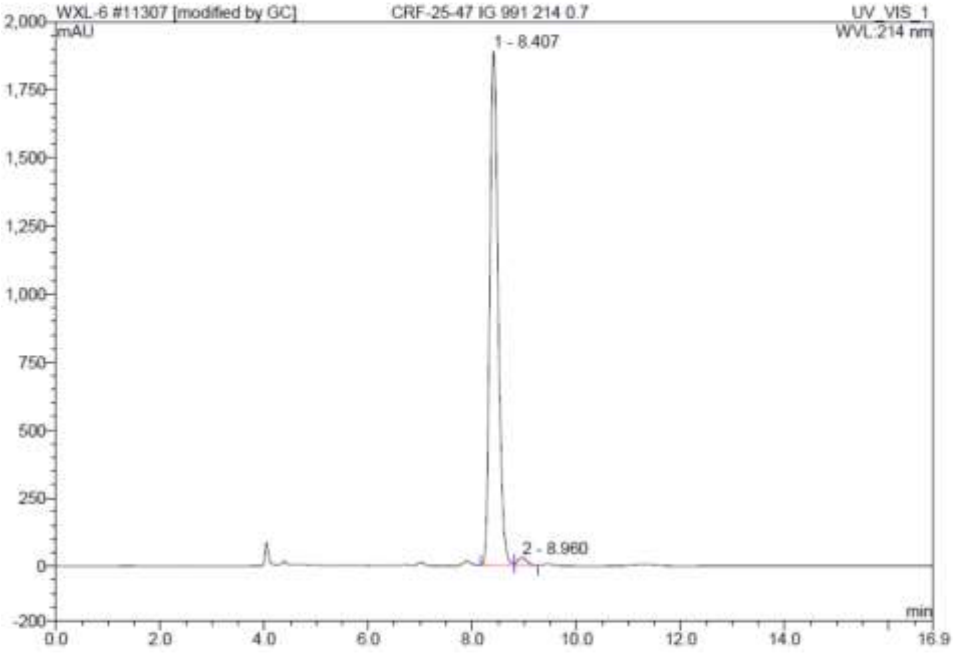

No.	Ret.Time min	Peak Name	Height mAU	Area mAU*min	Rel.Area %	Amount	Type
1	8.41	n.a.	1889.400	350.768	98.32	n.a.	BM *
2	8.96	n.a.	28.288	5.991	1.68	n.a.	MB*
Total:			1917.688	356.759	100.00	0.000	

Supplementary Figure 317. Chiral HPLC analysis of (*S*)-**3aa** in Table 1, Entry 9.

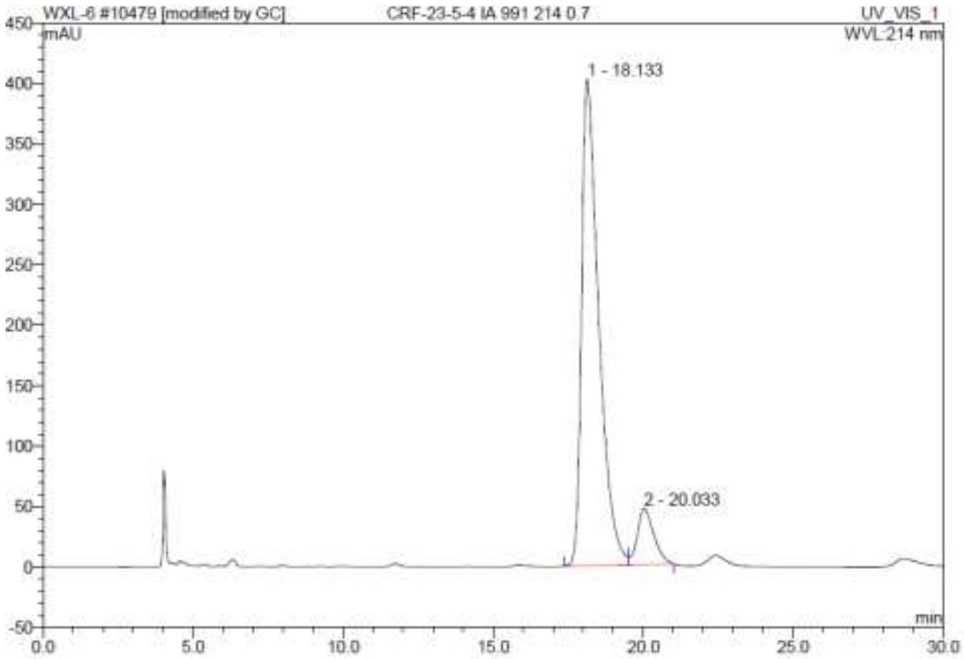

No.	Ret.Time min	Peak Name	Height mAU	Area mAU*min	Rel.Area %	Amount	Type
1	18.13	n.a.	402.232	266.418	89.98	n.a.	BM *
2	20.03	n.a.	46.324	29.668	10.02	n.a.	MB*
Total:			448.556	296.086	100.00	0.000	

Supplementary Figure 318. Chiral HPLC analysis of (*S*)-**3aa** in Table 1, Entry 10.

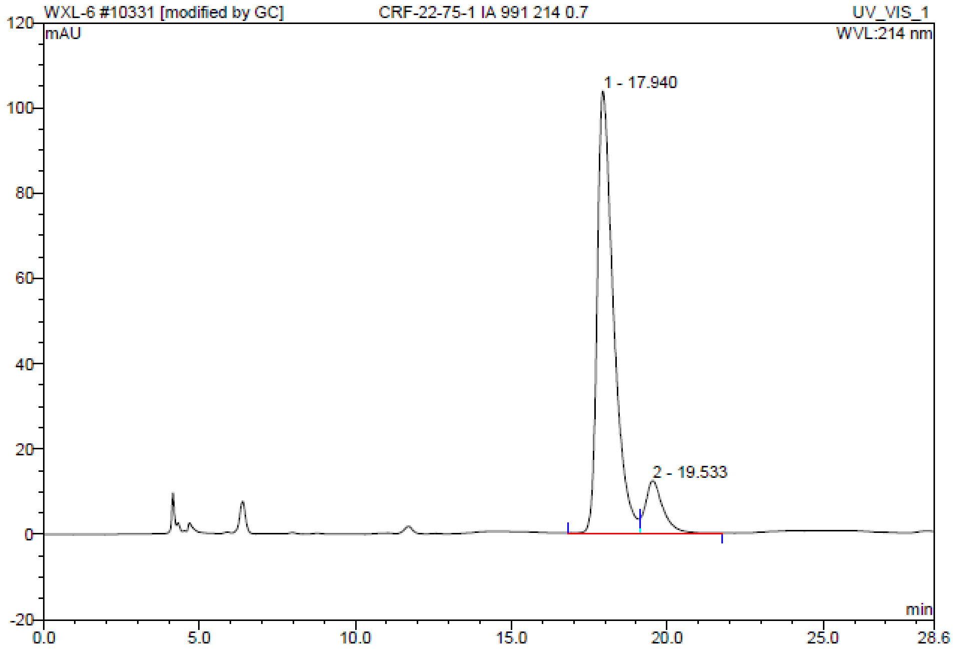

No.	Ret.Time min	Peak Name	Height mAU	Area mAU*min	Rel.Area %	Amount	Type
1	17.94	n.a.	103.669	62.670	88.50	n.a.	BM *
2	19.53	n.a.	12.343	8.143	11.50	n.a.	MB*
Total:			116.012	70.813	100.00	0.000	

Supplementary Figure 319. Chiral HPLC analysis of (*S*)-**3aa** in Table 1, Entry 11.

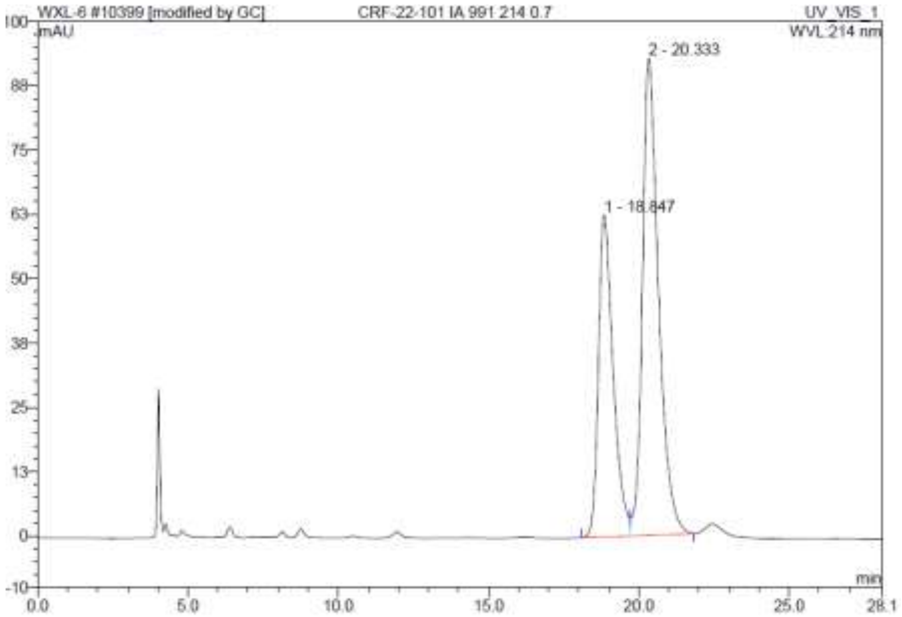

No.	Ret.Time min	Peak Name	Height mAU	Area mAU*min	Rel.Area %	Amount	Type
1	18.85	n.a.	62.522	35.479	38.08	n.a.	BM
2	20.33	n.a.	92.608	57.684	61.92	n.a.	MB*
Total:			155.129	93.164	100.00	0.000	

Supplementary Figure 320. Chiral HPLC analysis of (*S*)-**3aa** in Table 1, Entry 12.

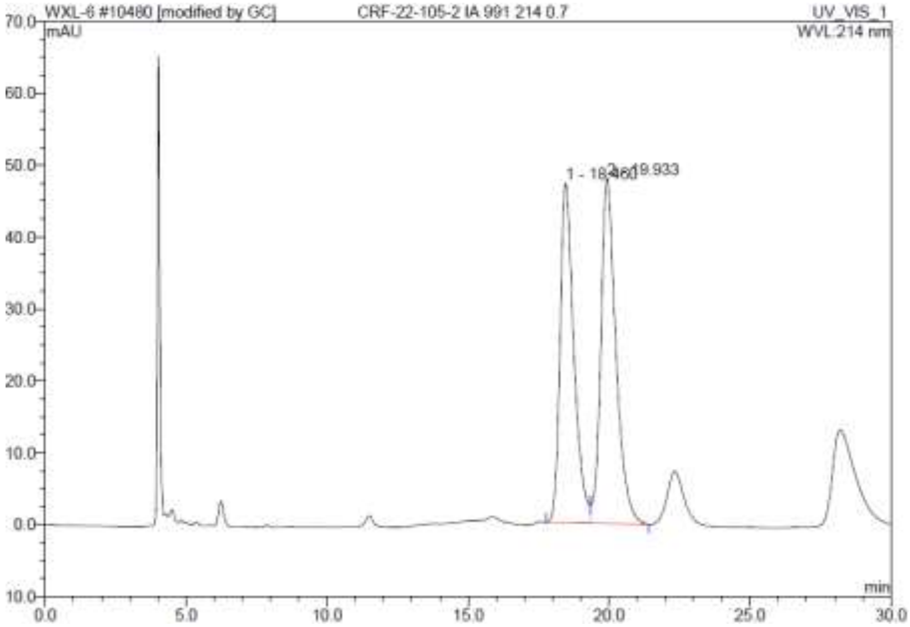

No.	Ret.Time min	Peak Name	Height mAU	Area mAU*min	Rel.Area %	Amount	Type
1	18.46	n.a.	47.232	26.658	47.21	n.a.	BM
2	19.93	n.a.	47.933	29.811	52.79	n.a.	MB
Total:			95.165	56.469	100.00	0.000	

Supplementary Figure 321. Chiral HPLC analysis of (*S*)-**3aa** in Table 1, Entry 13.

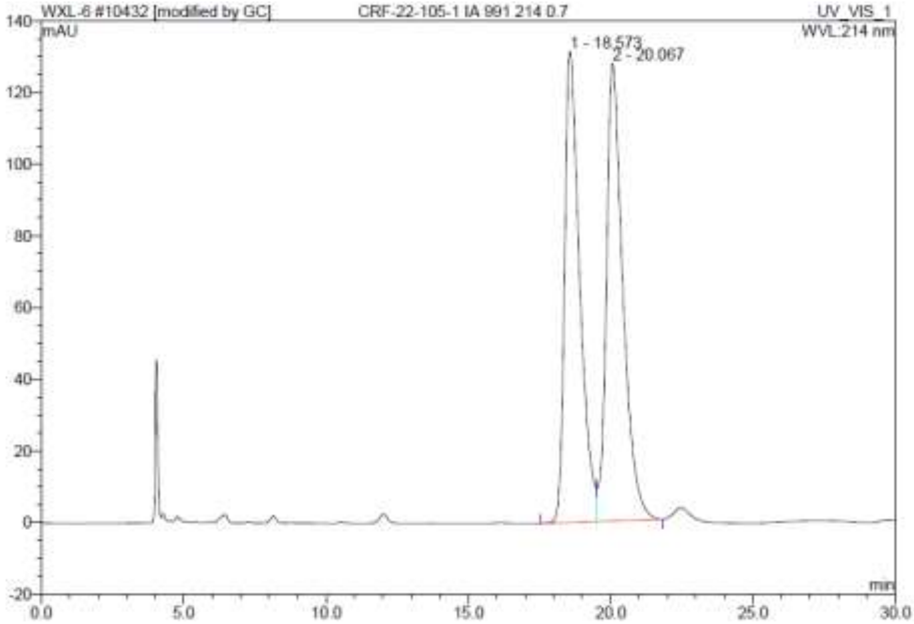

No.	Ret.Time min		Peak Name	Height mAU	Area mAU*min	Rel.Area %	Amount	Type
1	18.57	n.a.		131.500	81.016	48.55	n.a.	BM *
2	20.07	n.a.		127.742	85.862	51.45	n.a.	MB*
Total:				259.242	166.879	100.00	0.000	

Supplementary Figure 322. Chiral HPLC analysis of (*S*)-**3aa** in Table 1, Entry 14.

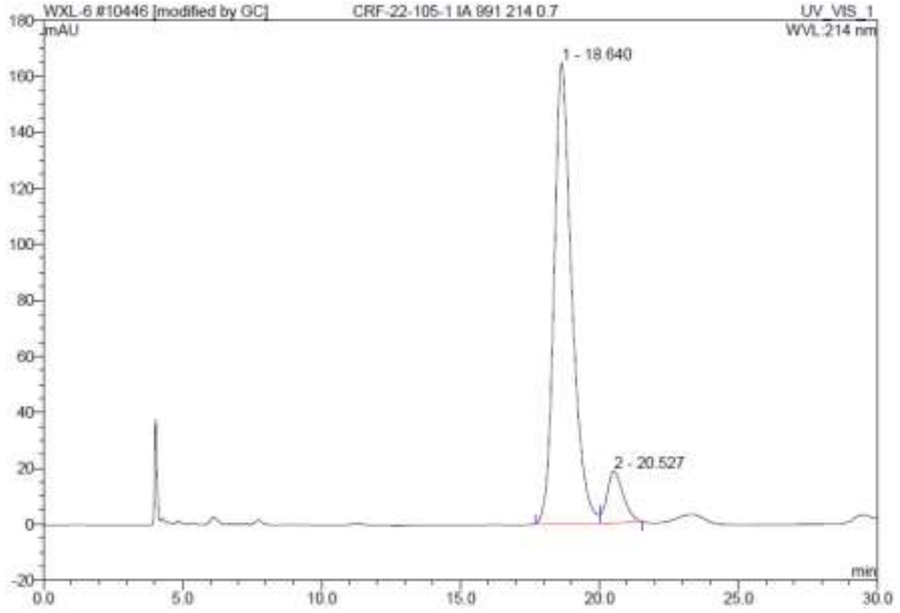

No.	Ret.Time min		Peak Name	Height mAU	Area mAU*min	Rel.Area %	Amount	Type
1	18.64	n.a.		164.516	122.298	90.69	n.a.	BM *
2	20.53	n.a.		18.624	12.550	9.31	n.a.	MB*
Total:				183.140	134.848	100.00	0.000	

Supplementary Figure 323. Chiral HPLC analysis of (*S*)-**3aa** in Table 1, Entry 15.

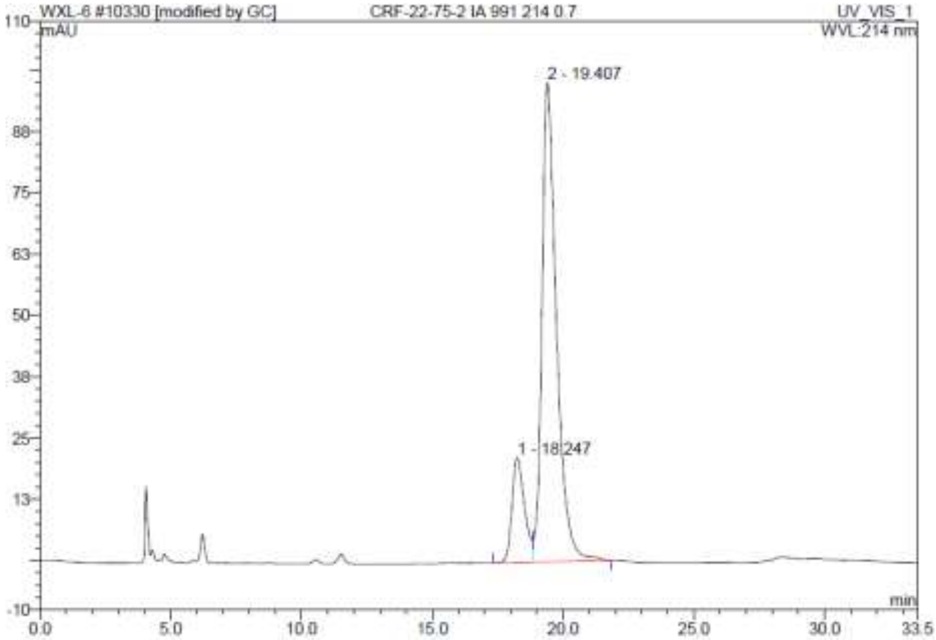

No.	Ret.Time min	Peak Name	Height mAU	Area mAU*min	Rel.Area %	Amount	Type
1	18.25	n.a.	21.288	11.690	15.68	n.a.	BM *
2	19.41	n.a.	97.615	62.875	84.32	n.a.	MB*
Total:			118.903	74.566	100.00	0.000	

Supplementary Figure 324. Chiral HPLC analysis of (*S*)-**3aa** in Table 1, Entry 16.

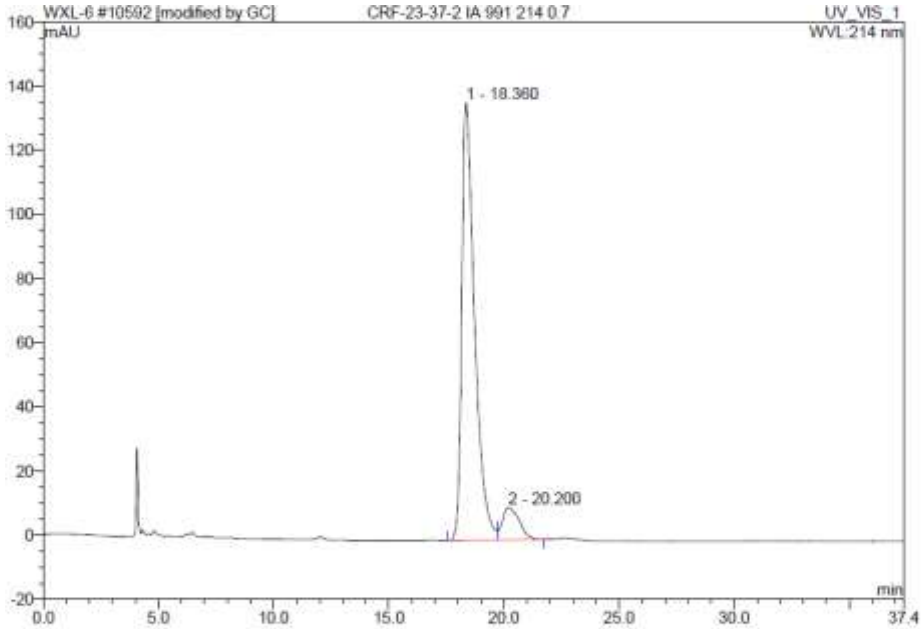

No.	Ret.Time min	Peak Name	Height mAU	Area mAU*min	Rel.Area %	Amount	Type
1	18.36	n.a.	136.352	87.875	91.08	n.a.	BM *
2	20.20	n.a.	10.071	8.611	8.92	n.a.	MB*
Total:			146.423	96.486	100.00	0.000	

Supplementary Figure 325. Chiral HPLC analysis of (*S*)-**3ba** in Table 1, Entry 17.

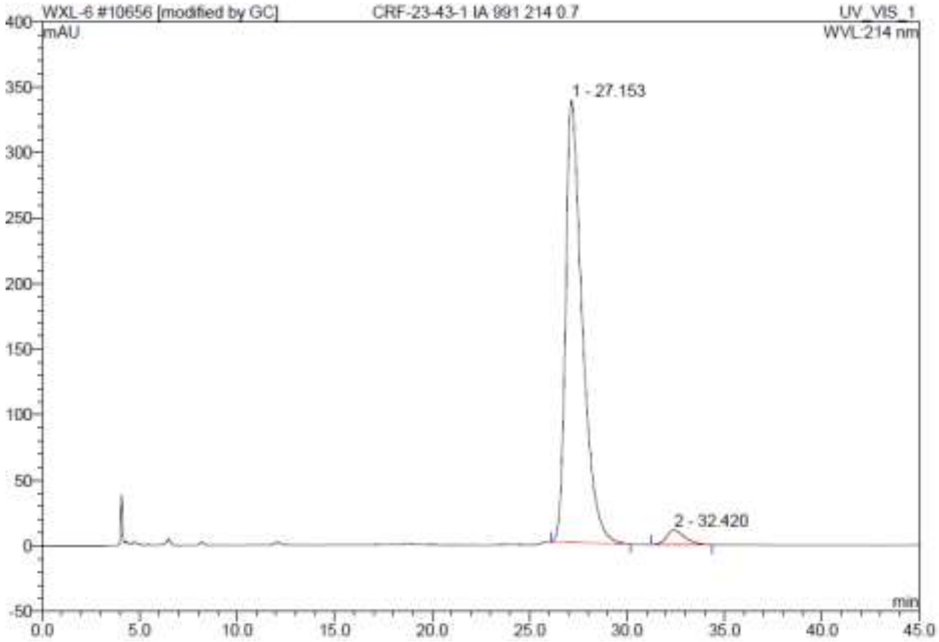

No.	Ret.Time min	Peak Name	Height mAU	Area mAU*min	Rel.Area %	Amount	Type
1	27.15	n.a.	337.720	334.004	96.48	n.a.	BMB
2	32.42	n.a.	11.097	12.188	3.52	n.a.	BMB
Total:			348.817	346.191	100.00	0.000	

Supplementary Figure 326. Chiral HPLC analysis of (*S*)-**3ba** in Table 2, Entry 1.

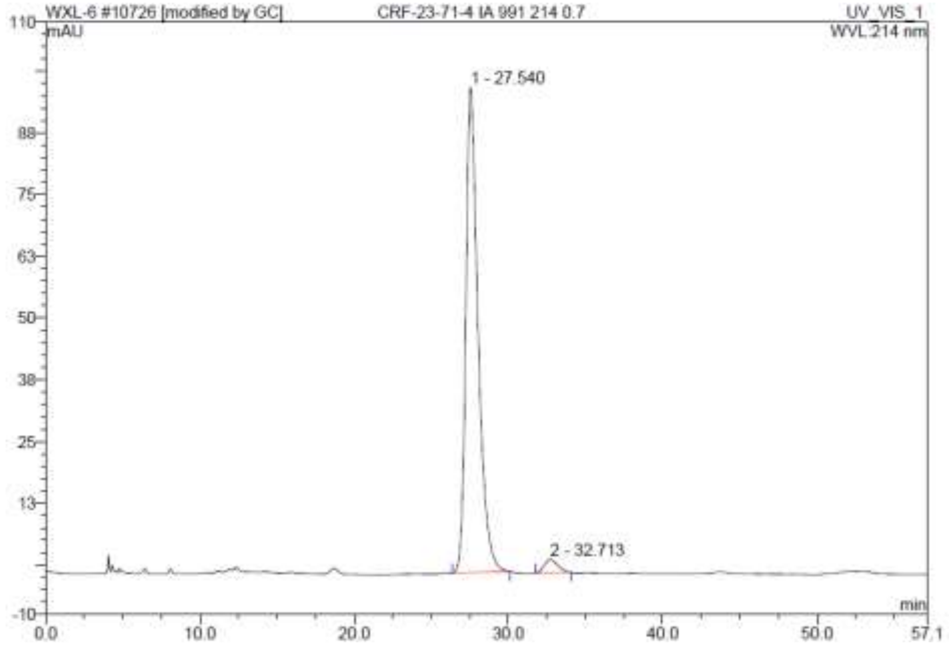

No.	Ret.Time min	Peak Name	Height mAU	Area mAU*min	Rel.Area %	Amount	Type
1	27.54	n.a.	98.328	90.328	97.10	n.a.	BMB
2	32.71	n.a.	2.686	2.695	2.90	n.a.	BMB
Total:			101.013	93.023	100.00	0.000	

Supplementary Figure 327. Chiral HPLC analysis of (*S*)-**3ba** in Table 2, Entry 4.

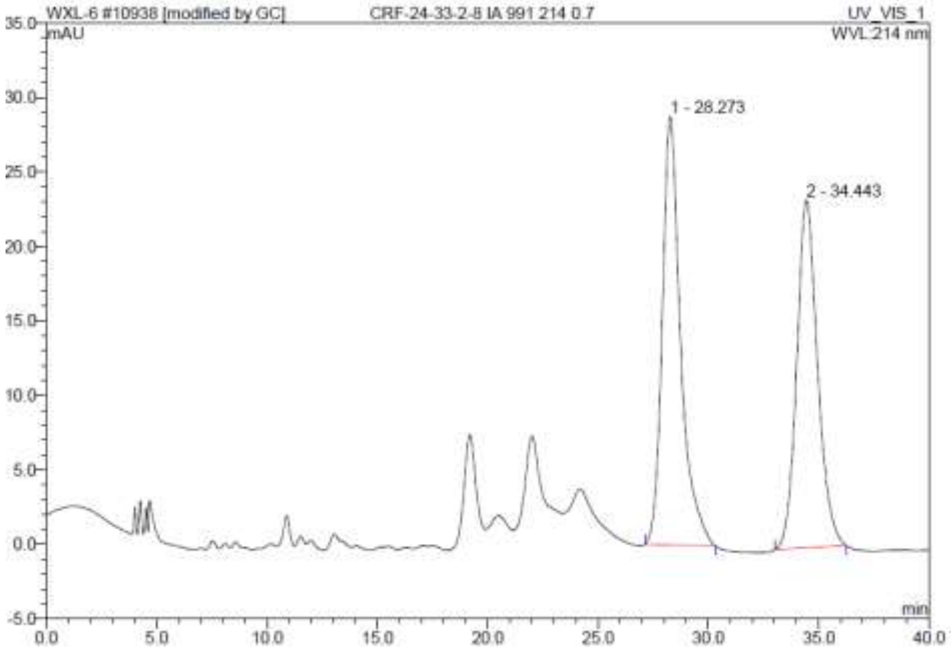

No.	Ret.Time min	Peak Name	Height mAU	Area mAU*min	Rel.Area %	Amount	Type
1	28.27	n.a.	28.743	27.669	52.09	n.a.	BMB
2	34.44	n.a.	23.342	25.448	47.91	n.a.	BMB
Total:			52.085	53.117	100.00	0.000	

Supplementary Figure 328. Chiral HPLC analysis of (*S*)-**3ba** in Table 2, Entry 5.

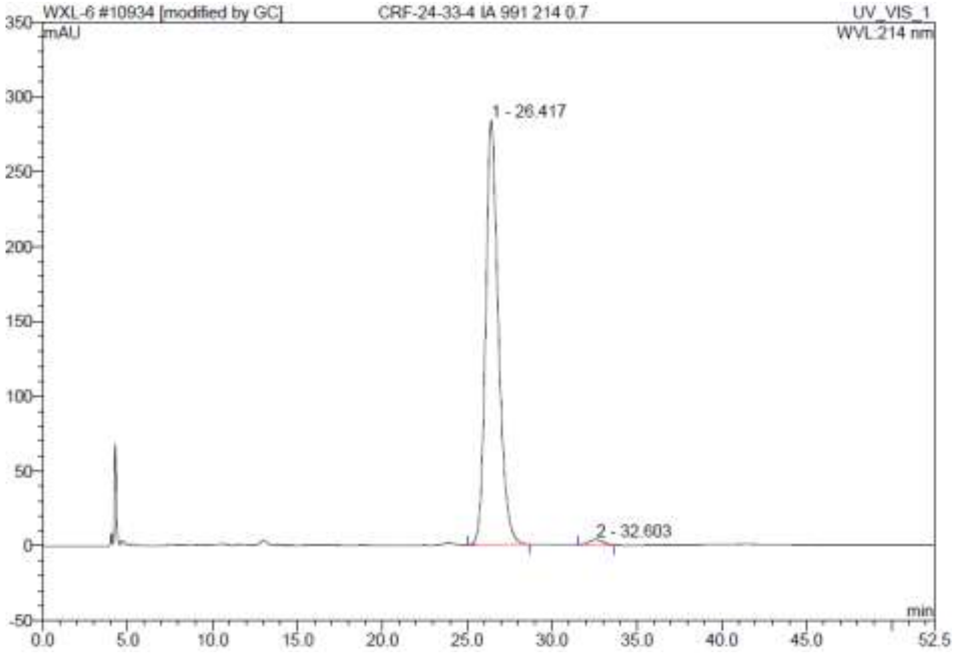

No.	Ret.Time min	Peak Name	Height mAU	Area mAU*min	Rel.Area %	Amount	Type
1	26.42	n.a.	283.846	248.116	98.63	n.a.	BMB
2	32.60	n.a.	3.505	3.437	1.37	n.a.	BMB
Total:			287.350	251.552	100.00	0.000	

Supplementary Figure 329. Chiral HPLC analysis of (*S*)-**3ba** in Table 2, Entry 6.

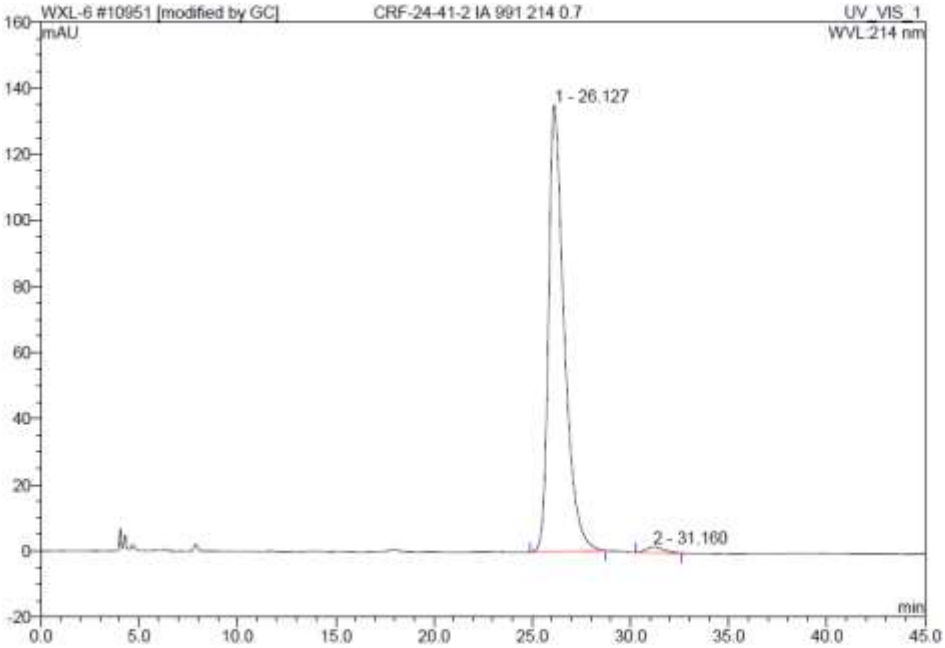

No.	Ret.Time min	Peak Name	Height mAU	Area mAU*min	Rel.Area %	Amount	Type
1	26.13	n.a.	135.082	124.632	98.55	n.a.	BMB
2	31.16	n.a.	1.727	1.834	1.45	n.a.	BMB
Total:			136.809	126.466	100.00	0.000	

Supplementary Figure 330. Chiral HPLC analysis of (*S*)-**3ba** in Table 2, Entry 7.

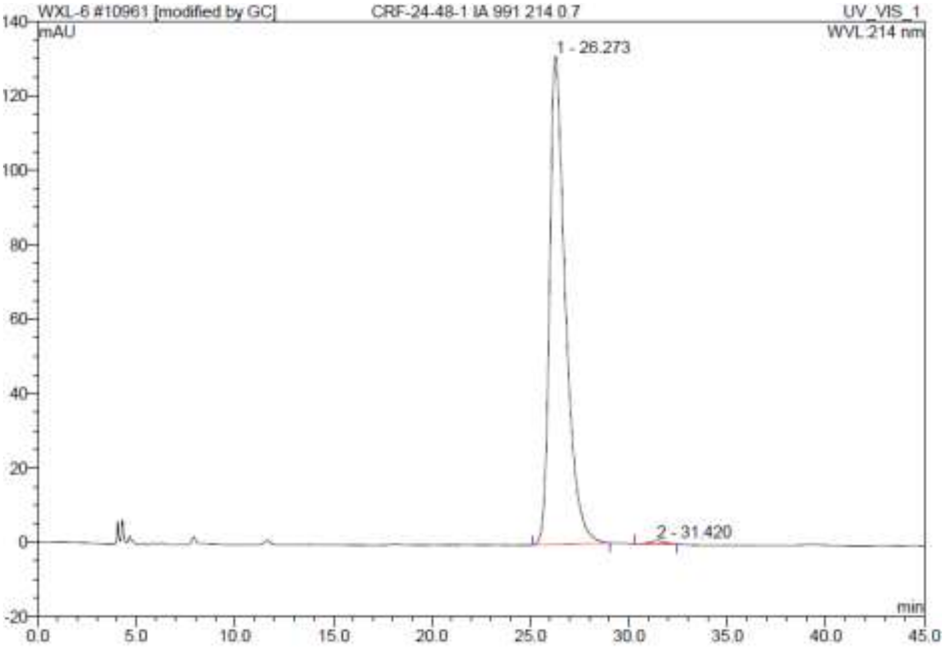

No.	Ret.Time min	Peak Name	Height mAU	Area mAU*min	Rel.Area %	Amount	Type
1	26.27	n.a.	131.077	122.104	99.40	n.a.	BMB
2	31.42	n.a.	0.835	0.736	0.60	n.a.	BMB*
Total:			131.912	122.840	100.00	0.000	

Supplementary Figure 331. Chiral HPLC analysis of (*S*)-**3ba** in Table 2, Entry 9.

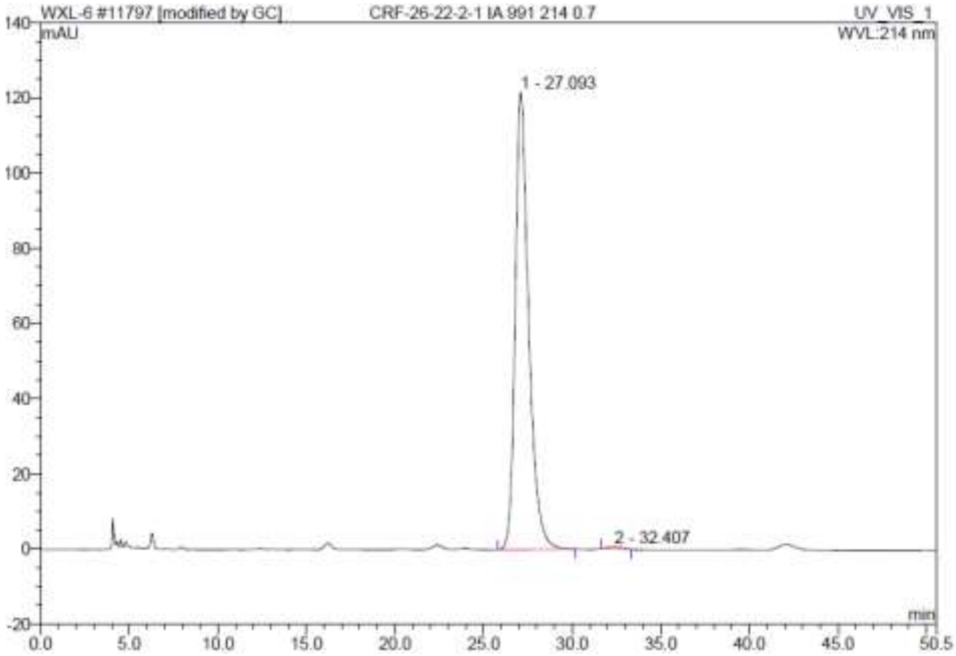

No.	Ret.Time min	Peak Name	Height mAU	Area mAU*min	Rel.Area %	Amount	Type
1	27.09	n.a.	121.583	107.016	99.50	n.a.	BMB*
2	32.41	n.a.	0.607	0.537	0.50	n.a.	BMB*
Total:			122.189	107.552	100.00	0.000	

Supplementary Figure 332. Chiral HPLC analysis of (*S*)-**3ba** in Table 2, Entry 10.

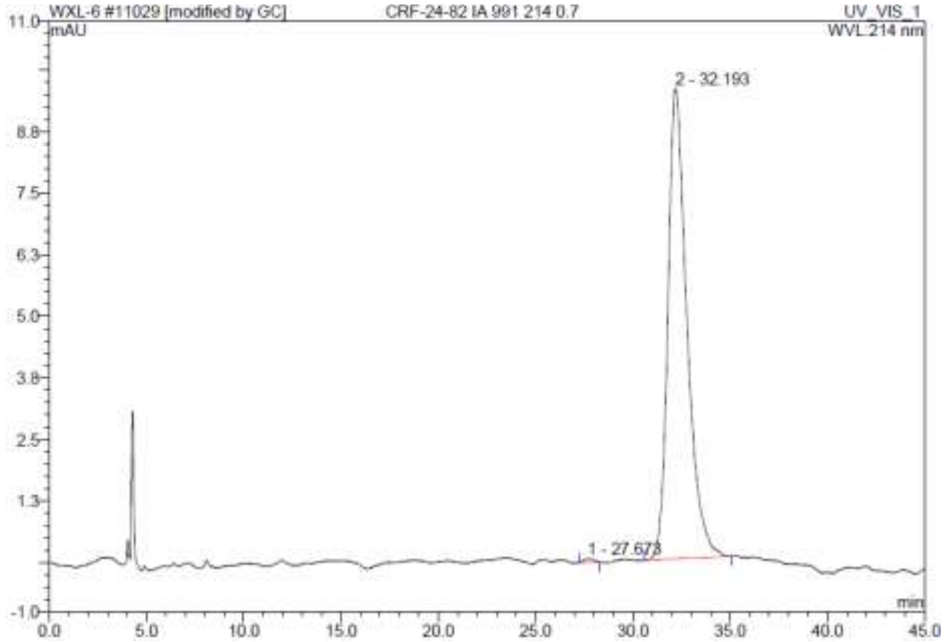

No.	Ret.Time min	Peak Name	Height mAU	Area mAU*min	Rel.Area %	Amount	Type
1	27.67	n.a.	0.086	0.045	0.42	n.a.	BMB*
2	32.19	n.a.	9.558	10.788	99.58	n.a.	BMB*
Total:			9.644	10.833	100.00	0.000	

Supplementary References

1. Gabbutt, C. D., Heron, B. M. & Instone, A. C. Control of the fading properties of photochromic 3,3-diaryl-3H-naphtho[2,1-b]pyrans. *Heterocycles* **60**, 843−855 (2003).
2. Yong, N., Wang, Y., Miao, J., Li, Y. & Zhang, Z. Synthesis and characterization of carboranyl schiff base compounds from 1-amino-*o*-carborane. *J. Organomet. Chem.* **798**, 182−188 (2015).
3. Lee, H., Onak, T., Jaballas, J. & Tran, U. Deuteration of *Closo*-1,2- and 1,7-$C_2B_{10}H_{12}$ Using $C_6D_6/AlCl_3$: Mechanistic Considerations. *Heteroatom Chem.* **9**, 95−102 (1998).
4. Sheldrick, G. M. SADABS: program for empirical absorption correction of area detector data. University of Göttingen, Germany (1996).
5. Sheldrick, G. M. SHELXTL 5.10 for Windows NT: structure determination software programs. Bruker Analytical X-ray Systems, Inc., Madison, Wisconsin, USA (1997).
6. Frisch, M. J. *et al.* Gaussian 09, Revision D.01; Gaussian, Inc., Pittsburgh, PA, (2009).
7. (a) Becke, A. D. Density-functional thermochemistry. III. The role of exact exchange. *J. Chem. Phys*. **98**, 5648−5652 (1993); (b) Lee, C., Yang, W. & Parr, R. G. Development of the colle-salvetti correlation-energy formula into a functional of the electron density. *Phys. Rev. B* **37**, 785−789 (1988).
8. (a) Grimme, S., Antony, J., Ehrlich, S. & Krieg, H. A consistent and accurate *ab initio* parametrization of density functional dispersion correction (DFT-D) for the 94 elements H-Pu. *J. Chem. Phys*. **132**, 154104 (2010). (b) Grimme, S. Semiempirical GGA-type density functional constructed with a long-range dispersion correction. *J. Comput. Chem*. **27**, 1787−1799 (2006).
9. (a) Hay, P. J. & Wadt, W. R. Ab initio effective core potentials for molecular calculations. Potentials for the transition metal atoms Sc to Hg. *J. Chem. Phys*. **82**, 270−283 (1985); (b) Wadt, W. R. & Hay, P. J. Ab initio effective core potentials for

molecular calculations. Potentials for main group elements Na to Bi. *J. Chem. Phys.* **82**, 284−298 (1985); (c) Hay, P. J. & Wadt, W. R. Ab initio effective core potentials for molecular calculations. Potentials for K to Au including the outermost core orbitals. *J. Chem. Phys.* **82**, 299−310 (1985).

10. Lu, T. & Chen, F. Multiwfn: a multifunctional wavefunction analyzer. *J. Comput. Chem.* **33**, 580–592 (2012).
11. Humphrey, W., Dalke, A. & Schulten, K. VMD: visual molecular dynamics. *J. Mol. Graphics* **14**, 33−38 (1996).
