# Supplementary material for: Ir-catalyzed enantioselective B−H alkenylation for asymmetric synthesis of chiral-at-cage o‑carboranes
Source: Nat Commun. 2021 Dec 8;12:7146. doi: 10.1038/s41467-021-27441-y (PMC8654863; doi:10.1038/s41467-021-27441-y)
Supplement: Supplementary file 2 — Description of Additional Supplementary Files [file 41467_2021_27441_MOESM2_ESM.pdf]

Description of Additional Supplementary Files

File name: Supplementary Data 1

Description: 1 Cartesian coordinates in .txt format for the optimized structure of TS-S 352

File name: Supplementary Data 2

Description: Cartesian coordinates in .txt format for the optimized structure of TS-R
